# Supplementary material for: Photoinduced ynamide structural reshuffling and functionalization
Source: Nat Commun. 2022 Apr 29;13:2345. doi: 10.1038/s41467-022-30001-7 (PMC9055057; doi:10.1038/s41467-022-30001-7)
Supplement: Supplementary file 2 — Supplementary Information [file 41467_2022_30001_MOESM2_ESM.pdf]

Supplementary Information

Photoinduced Ynamide Structural Reshuffling and Functionalization

Mohana Reddy Mutra[1] and Jeh-Jeng Wang[1,2]*

[1]Department of Medicinal and Applied Chemistry, Kaohsiung Medical University, No. 100, Shih-Chuan 1st Rd, Sanmin District, Kaohsiung City, 807 (Taiwan).

[2]Department of Medical Research, Kaohsiung Medical University Hospital, No. 100, Tzyou 1st Rd, Sanmin District, Kaohsiung City, 807 (Taiwan).

E-mail: jjwang@kmu.edu.tw

Table of Contents

1. **Supplementary Methods, Tables and Figures**

1.1. General information……………………………………………………………....................S3

1.2. Handling of reaction and blue LED technical details……………………………………...S4-S11

1.3. Reasons for very broad signal and no sharp peaks in compound (**3**) derivatives……………S12

1.4. Investigation and explanation of side product (**4**) formation………………………………...S13-S14

1.5. Preparation of starting Materials……………………………………………………………..S15-S27

1.6. Reaction optimization for compound **76**…………………………..………………………S28-29

1.7. Experimental procedures……………………………………………………………………..S29-S36

1.8. List of unsuccessful radical precursors..S37

1.9. Blue LED emission spectra and absorption spectra of-
compounds **2a**, **2ca**, **2da**, **2bd**, **2ea** and **1a**...S38-S57

1.10. Control studies for compounds **3**, **51** and **76**………………………………………………..S58-S70

1.11. Plausible mechanism for compound **76** formation..S71

1.12. Characterization data...S72-S107

1.13. Copies of ^{1}H, ^{13}C spectra…………………………………………………………S108-S235

2. **Supplementary Notes**

X-ray crystal data of compound **3**…………………………………………………..……....S236-S243

X-ray crystal data of compound **4**…………………………………………………..………S244-S247

X-ray crystal data of compound **14**…………………………………………………..……..S248-S251

X-ray crystal data of compound **50**…………………………………………………..……..S252-S255

X-ray crystal data of compound **51**…………………………………………………..……..S256-S259

X-ray crystal data of compound **76**…………………………………………………..……..S260-S263

X-ray crystal data of compound **94a**………………………………………………..……..S264-S267

X-ray crystal data of compound **95**…………………………………………………..……...S268-S271

X-ray crystal data of compound **97a**…………………….…………………………..……S272-S275

3. **Supplementary References**…………………………………………………….............S276

1. Supplementary Methods, Tables and Figures

1.1. General Information

^{1}H and ^{13}C NMR spectra were recorded on a 400 MHz and 600 MHz Varian Unity Plus or Varian Mercury plus spectrometer. The chemical shift (δ) values are reported in parts per million (ppm), and the coupling constants (J) are given in Hz. The spectra were recorded using $CDCl_3$, DMSO-d_6, and CD_3COCD_3 as a solvent. ^{1}H NMR chemical shifts are referenced to tetramethylsilane (TMS) (0 ppm). ^{13}CNMR was referenced to $CDCl_3$ (77.0 ppm), DMSO-d_6 (39.52), CD_3COCD_3 (29.84). The abbreviations used are as follows: s, singlet; d, doublet; t, triplet; q, quartet; dd, doublet of doublet; ddd, doublet of doublet; dt, doublet of triplets; td, a triplet of doublet; m, multiplet; brs, broad singlet and so on. Mass spectra and high-resolution mass spectra (HRMS) were measured using the LTQ Orbitrap XL (Thermo Fisher Scientific) Liquid chromatography-mass spectrometry at National Taiwan Normal University and National Sun Yat-sen University. All commercially available reagents were used without further purification unless noted otherwise. Commercially available reagents and solvents were obtained from Sigma-Aldrich, TCI, Acros, or Alfa Aesar. Melting points were determined on an EZ-Melt (Automated melting point apparatus). Absorption spectra were obtained on an UV-1900 Vis spectrophotometer (Shimadzu). All the synthesized products showed ^{1}HNMR spectra in agreement with the assigned structures. Reaction progress and product mixtures were routinely monitored by TLC using Merck TLC aluminum sheets (silica gel 60 F254). Column chromatography was carried out with 230-400 mesh silica gel 60 (Merck) using a mixture of hexane/ethyl acetate as the eluent.

1.1.1 Light Sources

Reactions were carried out using 40 W blue LED lamp (Kessil A160WE Controllable LED Aquarium Light) purchased from Kessil via Amazon (Taiwan). See the following links for more details. https://www.kessil.com/aquarium/saltwater_A160.php
https://www.kessil.com/support/downloadfiles/aquarium/A160WE_UserManual.pdf

40 W blue LED lamp (PR160L-456 nm) purchased from Fiberoptics & DiCon Lighting, Kaohsiung, Taiwan. See the links for more details. https://kessil.com/science/PR160L.php

1.1.2 Blue LED Emission Spectra

Emission spectra were measured using Ocean Optics USB 2000+ Spectrometer. Spectra were normalized to 1.0 at the emission maximum. These emission spectra were provided by Miss Angela Liou, Sales Specialist, DiCon Fiberoptics & DiCon Lighting, aliou@diconfiberoptics.com, Kaohsiung, Taiwan, +886 7 815-8055 Ext 485, DiCon Brands - Kessil | Fiilex | Cielux.

1.2. Handling of our photoinduced diversity radical transformation

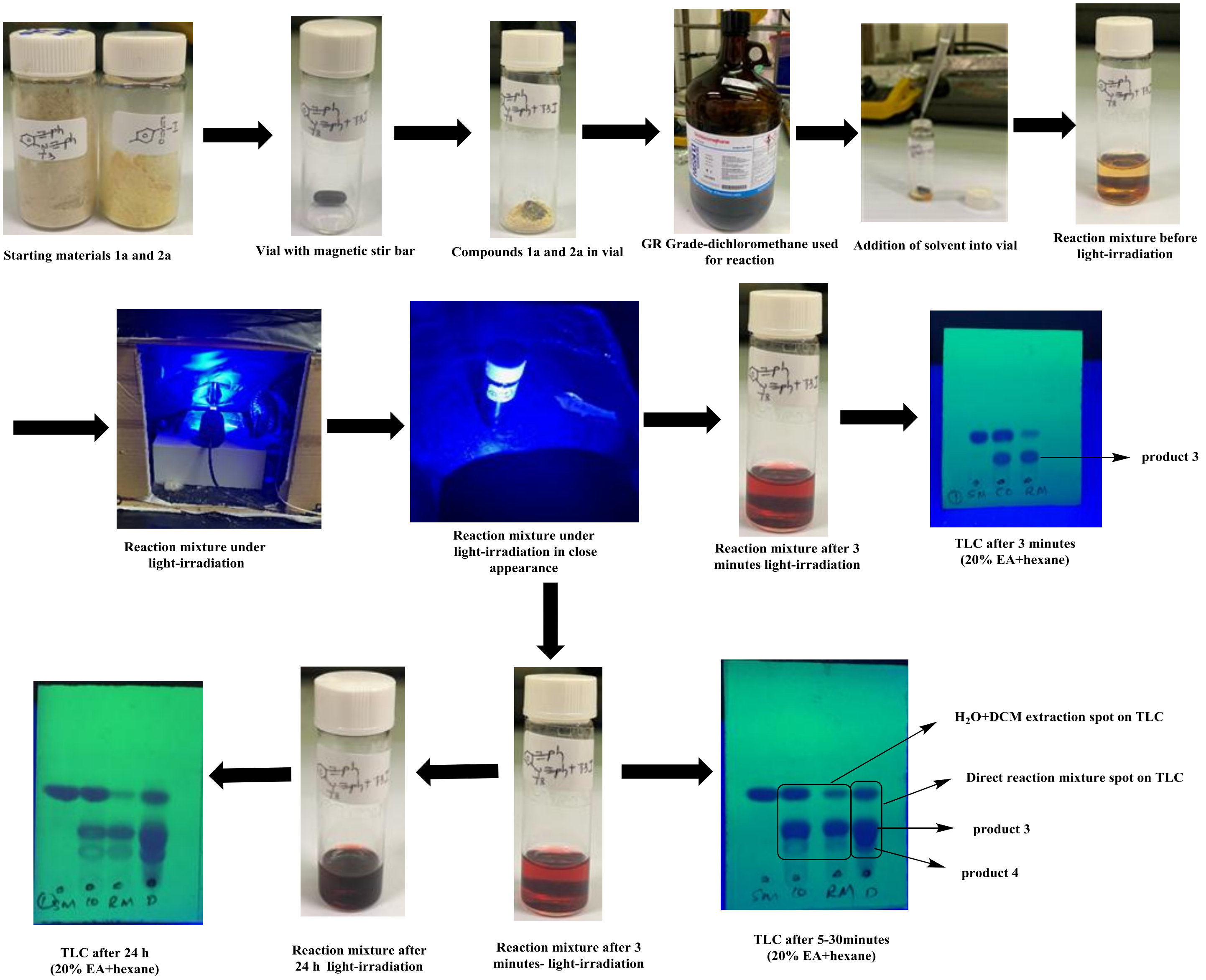

Supplementary Figure 1. Pictures represent the reaction setup under light-irradiation of blue LED source by using compounds **1a** and **2a** and the progress of the reaction monitored by thin-layer chromatography.

1.2.1. Handling of our photoinduced diversity radical transformation under N_2 atmosphere

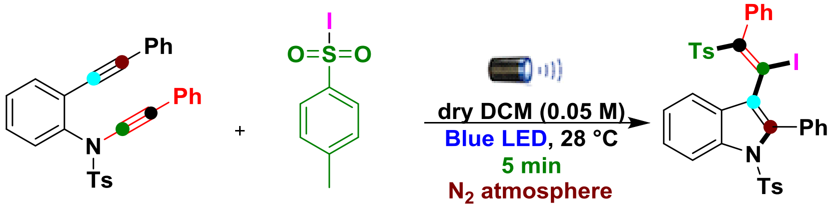

1a, 0.1 mmol **2a**, 0.11 mmol **3**, 81%

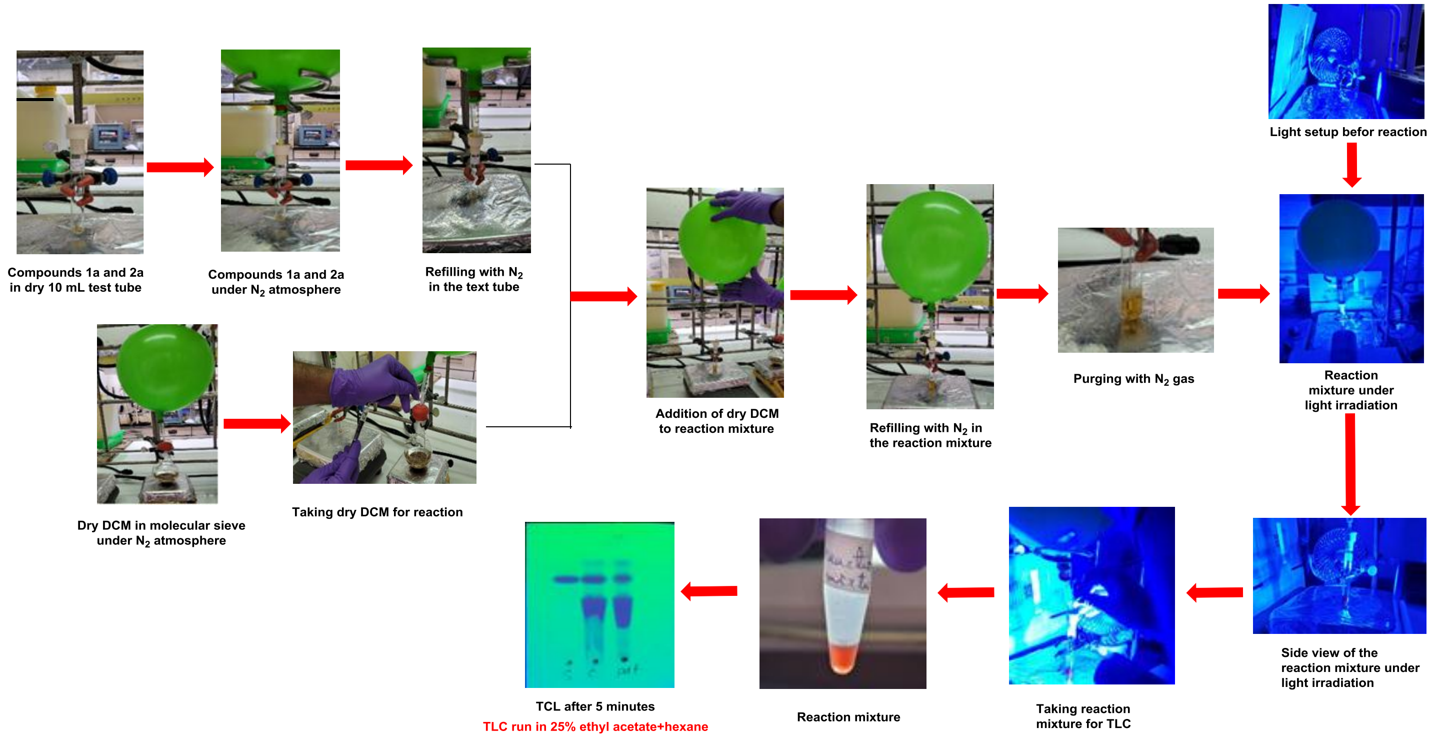

Supplementary Figure 2. Pictures represent the reaction setup of N_2 balloon under light-irradiation of 40 W blue LED source by using compounds **1a** and **2a** and the progress of the reaction monitored by thin-layer chromatography.

1.2.2. Handling of our diversity radical transformation in absence of light source under N_2 atmosphere

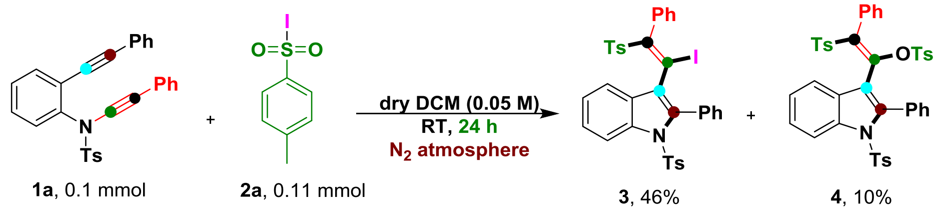

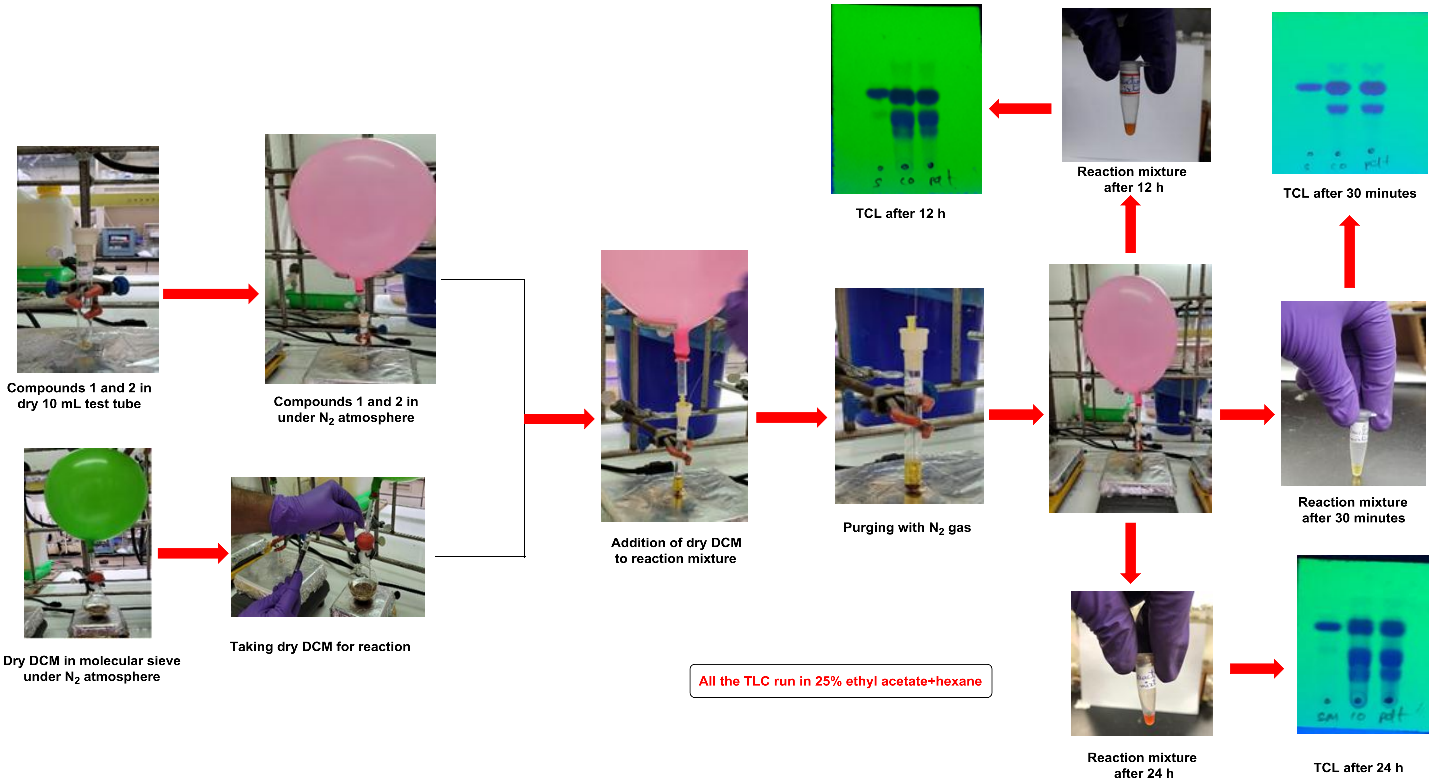

Supplementary Figure 3. Pictures represent the reaction setup of N_2 balloon at room temperature by using compounds **1a** and **2a** and the progress of the reaction monitored by thin-layer chromatography.

1.2.3. Handling of our diversity radical transformation with 40 W PR160L-456 nm blue LED

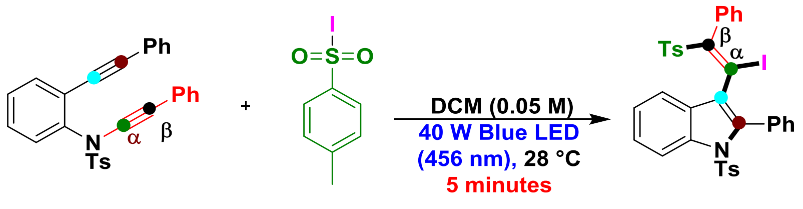

0.1 mmol, **1a** 0.11 mmol, **2a** **3,** 80%

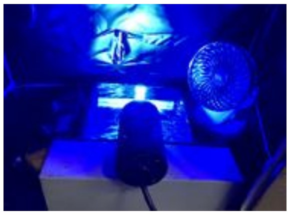

Reaction under PR160L-456 nm blue LED light

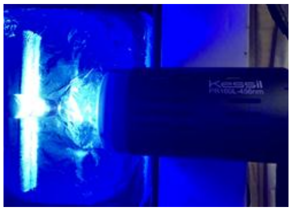

Upper view of the reaction

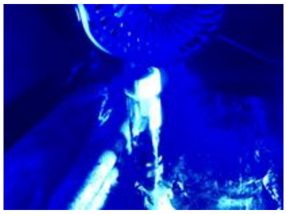

Side view of the reaction

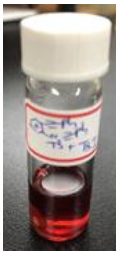

Reaction vial after 5 minutes

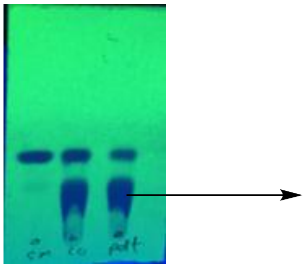

Compound **3**

25% EA+Hexane
Reaction mixture TLC after 5 minutes

Supplementary Figure 4. Pictures represent the reaction setup under light-irradiation of 40 W PR160L-456 nm source by using compounds **1a** and **2a** and the progress of the reaction monitored by thin-layer chromatography.

1.2.4. Investigation of the reaction temperature

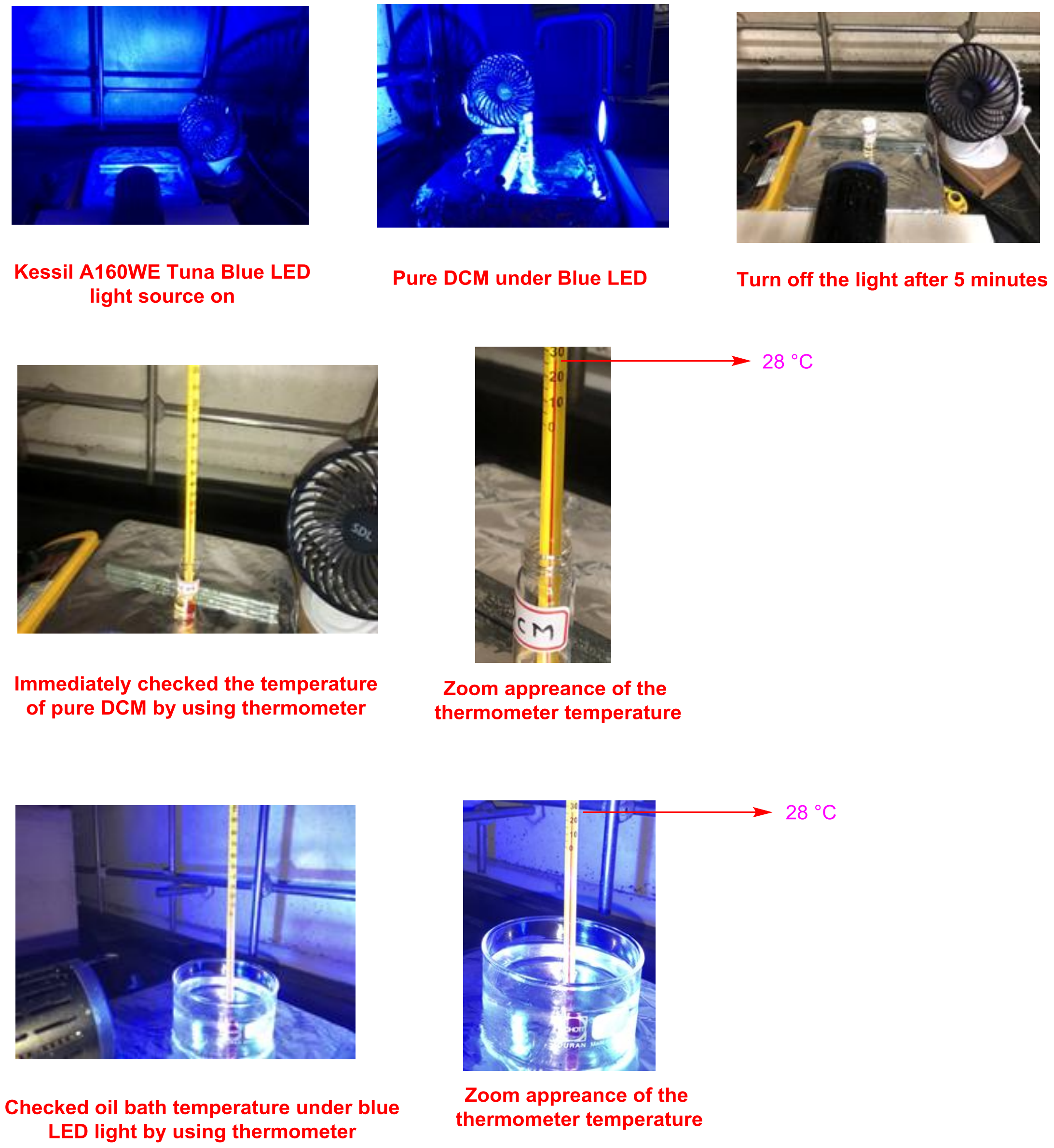

Supplementary Figure 5. Pictures represent the pure DCM solvent and oil bath temperature under light-irradiation (5 minutes) of 40 W Kessil A160WE Tuna Blue LED.

1.2.5. Reaction under traditional heating condition in absence of light source

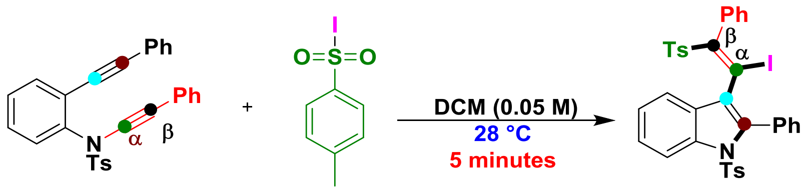

0.1 mmol, **1a** 0.11 mmol, **2a** **3,** 0%

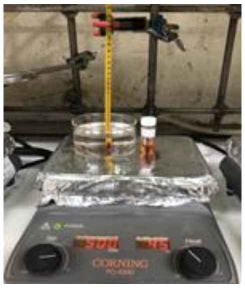

Reaction under similar temperature with heating

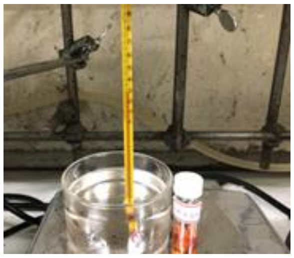

Close appreance of the reaction

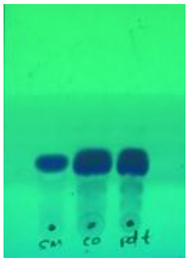

25% EA+Hexane

Reaction mixture TLC after 5 minutes

Supplementary Figure 6. Pictures represent the similar temperature in absence of light source.

1.2.6. Complete reaction setup and Blue LED technical details

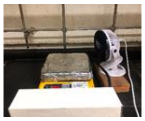

Front side view of stirrer with fan (For clarity we took picture without cardboard box)

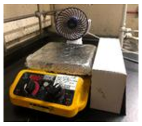

side view of stirrer with fan (For clarity we took picture without cardboard box)

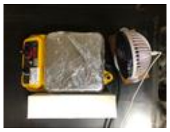

Upper view of stirrer with fan (For clarity we took picture without cardboard box)

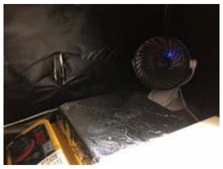

The reaction setup under cardboard box

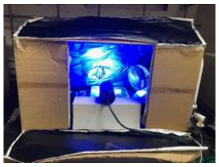

Outside view of the setup (door open)

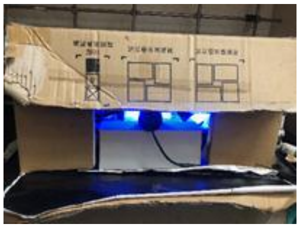

Outside view of the setup(door closed)

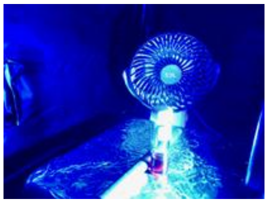

Cooling of the reaction under light irradiation

Scale

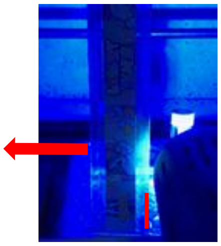

1 cm hight (light source)

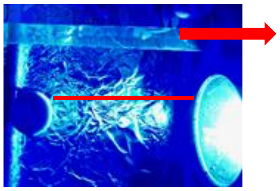

Scale

8.5 cm distance from light source to reaction vial

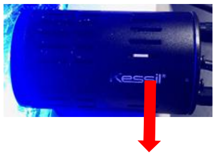

A160WE Tuna Blue

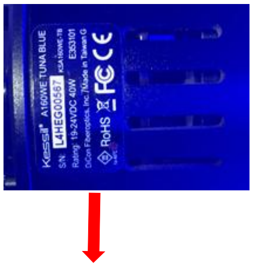

Manufacturing and other technical details on light source

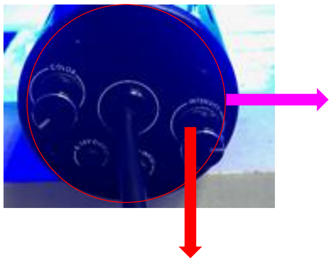

Half of the deep ocean blue LED intensity we setup for our reaction

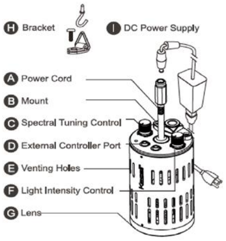

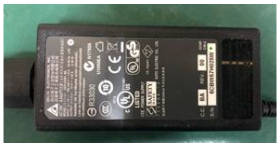

A160WE Tuna Blue power adapter

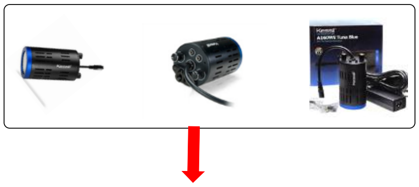

This A160WE Tuna Blue LED purched from amazon Taiwan website

Technical Specifications

Specifications

**Dimensions**	4" x 2.48" (H x D)
**Unit Weight**	0.69 lb / 0.32kg
**Spectrum**	Deep Ocean Blue to Sky
**Coverage**	Up to 24" surface diameter
**Power Adapter**	100-240V AC (input)<br>19-24V DC (output)
**Power Consumption**	40W

https://www.kessil.com/aquarium/saltwater_A160.php

https://www.kessil.com/support/downloadfiles/aquarium/A160WE_UserManual.pdf

Supplementary Figure 7. Pictures represent the complete reaction setup and other technical details of the 40 W Blue LED (Kessil A160WE Controllable LED Aquarium Light).

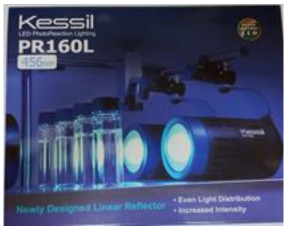

Outside box appearance of Kessil 40 W PR160L-456 nm blue LED light

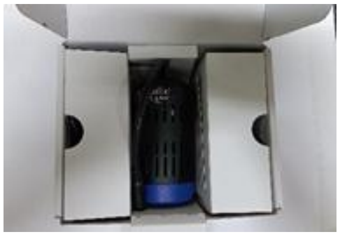

Kessil PR160L-456 nm blue LED light and supporting power cables inside the box

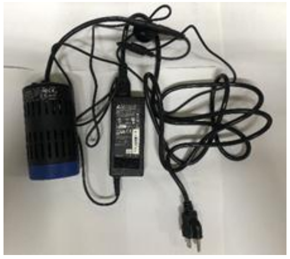

Kessil PR160L-456 nm blue LED light and supporting power cables outside the box

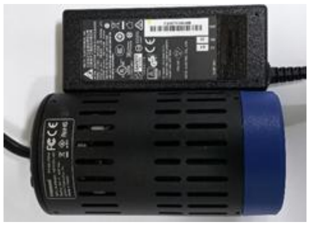

40 W blue LED light and adapter

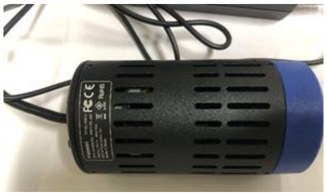

40 W blue LED light

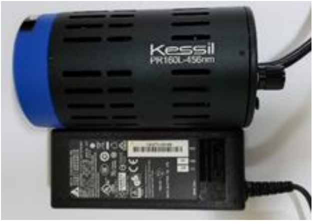

Kessil PR160L-450 nm

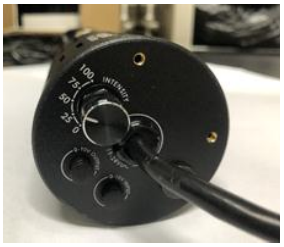

50% intensity of blue LED used for our reaction

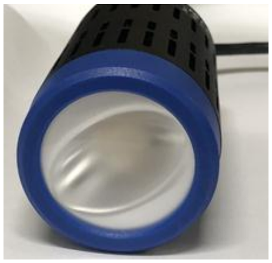

Frontside view of Blue LED light

Technical Specifications
Specifications

Power Consumption	=	456 nm (max 40W)
Input Voltage	=	100-240 VAC
Operating Temperature	=	0 - 40 °C / 32 - 104 °F
Beam Angle	=	56°
Average Intensity of PR160 series	=	352mW/cm2 (measured from 1 cm distance)
Dimensions	=	4.49" x 2.48" / 11.4cm x 6.3cm (H **x D)**

For more details please find the following link

https://kessil.com/science/PR160L.php

Supplementary Figure 8. Pictures represent the 40 W blue LED lamp (PR160L-456 nm) and other technical details.

1.3. Reasons for very broad signal and no sharp peaks in compound (3) derivatives

We examined the (*E*)-3-(1-iodo-2-phenyl-2-tosylvinyl)-2-phenyl-1-tosylindole (**3**) in $CDCl_3$ solvent at 600 MHz (proton and carbon) to determine the reasons for very broad signal and no sharp peaks (^{1}H NMR and ^{13}C NMR). In addition, we were used different solvents to check the result of the spectra, but there was no significant change in the shape of the peaks and the spectra were provided (see spectra section). Next, we checked the distance and bond angles of the crystal bond of **3** as shown below. The bond distance between I-H17: 4.146 Å, I-H21: 4.491 Å and the bond angle C22-I-H17: 39.48 ◦ , C22-I-H21: 77.73 ◦ as shown in Supplementary Figure 5. Based on the results, we hypothesized that bulky iodine could affect the neighboring aromatic protons, so that the ortho-protons (in the case of R^1= aromatic) were broadened at ~ 7.8 ppm in the 1H NMR. Moreover, indirect confirmation of the broad peaks in the products can be confirmed by the proton NMR of R^1= aliphatic in the (*E*)-3-(1-iodo-2-phenyl-2-tosylvinyl)-2-phenyl-1-tosylindole derivatives as shown below (see spectra of compounds **13** and **14**).

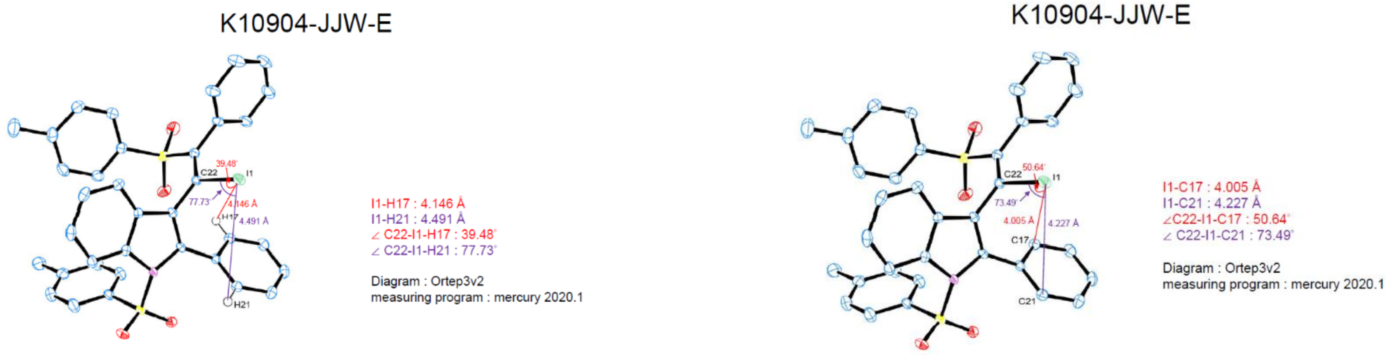

Supplementary Figure 9. Iodine and phenyl group bond distance and bond angles in the crystal structure of (*E*)-3-(1-iodo-2-phenyl-2-tosylvinyl)-2-phenyl-1-tosylindole (**3**).

1.4. Investigation and explanation of the side product (*E*)-2-phenyl-1-(2-phenyl-1-tosylindol-3-yl)-2-tosylvinyl 4-methylbenzenesulfonate (4) formations

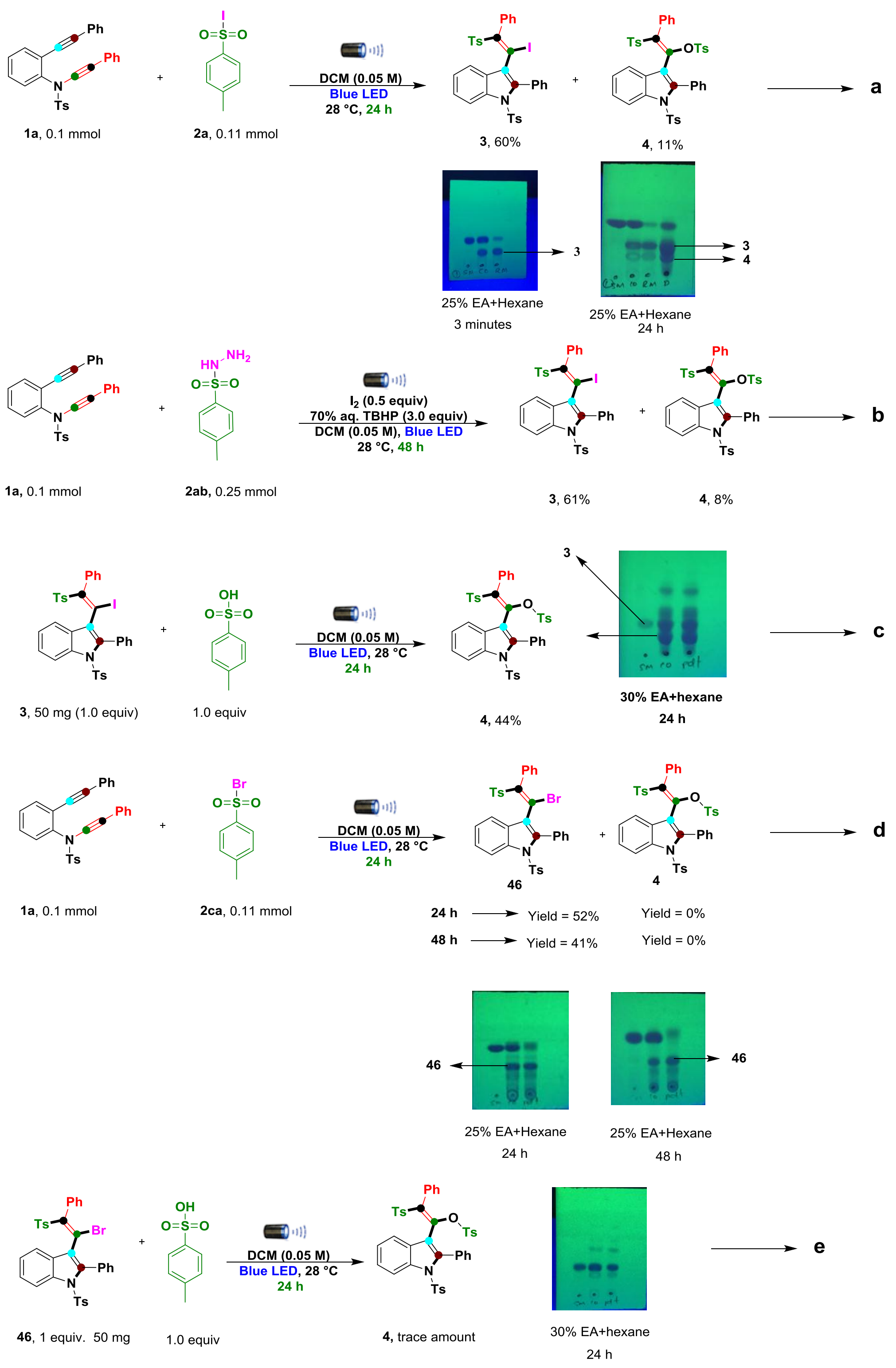

Supplementary Figure 10. Investigation of side product (*E*)-2-phenyl-1-(2-phenyl-1-tosylindol-3-yl)-2-tosylvinyl 4-methylbenzenesulfonate (**4**) with various radical precursors under longer reaction time.

Generation of the side product (*E*)-2-phenyl-1-(2-phenyl-1-tosylindol-3-yl)-2-tosylvinyl 4-methylbenzenesulfonate (**4**) was investigated with various radical precursors as shown above Supplementary Figure 10. First, we chose 4-methyl-*N*-(phenylethynyl)-*N*-(2-(phenylethynyl)phenyl)benzenesulfonamide **(1a)** with 4-methylbenzenesulfonyl iodide **(2a)** as radical precursor under standard reaction conditions for 24 h (Supplementary Figure 10a). At short reaction time (2-5 minutes), we did not observe the formation of the compound **4** but under longer reaction time, observed formation of the side product (**4)** with 11% yield. We hypothesized that the probable reason for the side product formation could be the in situ generation of TsOH from **2a** with a trace amount of water (The compound **2a** was synthesized from sodium p-toluenesulfinate and I_2 in H_2O as a solvent. After formation of yellow solid in the reaction, filtered by using Büchner funnel. The yellow solid was washed with water (2-3 times) to remove unreacted starting material (sodium p-toluenesulfinate or I_2). The yellow solid was dried 5-10 minutes (longer time, the compound will decompose) under high vacuum pump and carried to the next step without further workup or purification. In this entire process, we believe there is a trace amount of water in the compound **2a**).

In case of 4-methylbenzenesulfonohydrazide (**2ab**) as a radical precursor under standard conditions for 48 h (Supplementary Figure 10b), observed side product **4** formation in 8% yield (herein, the water source is aq. TBHP).

Next, we chose compound **3** as a starting material with commercially available TsOH.H_2O under standard reaction conditions for 24 h (Supplementary Figure 10c) and the reaction affords the side product **4** formation in 44% yield. This reaction suggests that the in situ generated TsOH can act as a nucleophile with weak C-I bond in the product **3**.

In addition to the above radical precursors, we chose the 4-methylbenzenesulfonyl bromide (**2ca**) as radical precursors to check the formation of side products in our standard reaction condition (24-48 h) as shown above (Supplementary Figure 10d) but we did not observe any side products (**4**). Next, we chose compound **3** as a starting material with commercially available TsOH.H_2O under standard reaction condition for 24 h (Supplementary Figure 10e) and the reaction did not afford the side product **4** except a trace amount on TLC. The probable reason could be stronger $C_{sp}{}^2$-Br bond compared to $C_{sp}{}^2$-I bond in compound **46** for the in situ generated TsOH.

1.5. Preparation of Starting Materials

1.5.1. Procedure for the synthesis of (Bromoethynyl)benzene

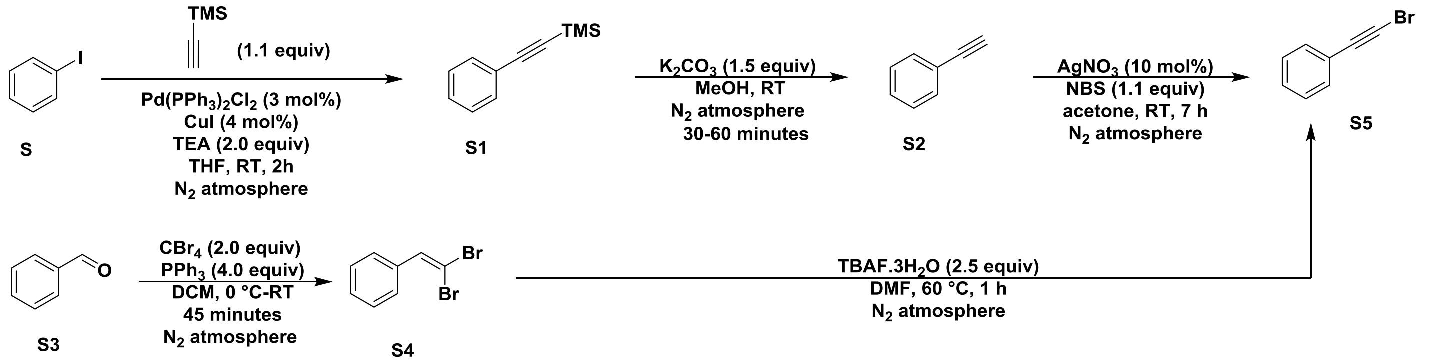

1.5.1a. Procedure for the synthesis of trimethyl(phenylethynyl)silane (S1):

To a dried schlenk flask equipped with a stir bar was charged with iodobenzene (6 g, 29.41 mmol, 1.0 equiv), trimethylsilylacetylene (3.17 g, 32.35 mmol, 1.1 equiv) in THF at room temperature. Next, the schlenk tube was evacuated and filled with nitrogen (three cycles), followed by freshly distilled Et_3N (5.95 g, 58.82 mmol, 2.0 equiv), $Pd(PPh_3)_2Cl_2$ (0.61 g, 8.82 mmol, 3 mol%), CuI (0.22 g, 1.17 mmol, 4 mol%) were added under the N_2 atmosphere. The resulting mixture was stirred at room temperature for 2 h. After the completion of the reaction by TLC, the reaction mixture was diluted with water, and extracted with ethyl acetate. The combined organic layer was dried over Na_2SO_4, filtered, and concentrated to give the crude material. The crude material was purified by column chromatography using hexane (100%) as the eluent gave brown liquid trimethyl(phenylethynyl)silane in 88% (4.5 g).

1.5.1b. Procedure for the synthesis of ethynylbenzene (S2)

To a dried schlenk flask equipped with a stir bar was charged with trimethyl(phenylethynyl)silane (4.5 g, 25.86 mmol, 1.0 equiv) in MeOH at room temperature. Next, the schlenk flask was evacuated and filled with nitrogen (three cycles), followed by K_2CO_3 (5.36 g, 38.79 mmol, 1.5 equiv) was added under the N_2 atmosphere. The resulting mixture was stirred at room temperature for 30-60 minutes. The solvent was removed under reduced pressure and the resulting solid/liquid was dissolved in ethyl acetate, washed with water and brine, and dried over Na_2SO_4. The crude material was purified by column chromatography using hexane-ethyl acetate (99:1%) as the eluent gave colorless liquid ethynylbenzene in 94% (2.5 g).

1.5.1c. Procedure for the synthesis of (bromoethynyl)benzene (S5)

To a dried schlenk flask equipped with a stir bar was charged with ethynylbenzene (2.5 g, 24.47 mmol, 1.0 equiv) in dry acetone. Next, the schlenk flask was evacuated and filled with nitrogen (three cycles), followed by $AgNO_3$ (0.41 g, 2.44 mmol, 0.1 equiv), NBS (4.79 g, 26.92 mmol, 1.1 equiv) added in portions under the N_2 atmosphere. The mixture was stirred for 7 h at room temperature (the flask was covered with aluminium foil). After the completion of the reaction by TLC, the solvent was concentrated in vacuum. The reaction mixture was diluted with water, and extracted with ethyl acetate. The combined organic layer was dried over Na_2SO_4, filtered, and concentrated to give the crude material. The crude material was purified by column chromatography using hexane (100%) as the eluent gave yellow color liquid (bromoethynyl)benzene in 70% (3.1 g).

1.5.2. Alternative route for the synthesis of compound S5

1.5.2a. Procedure for Synthesis of (2,2-dibromovinyl)benzene (S4): CBr_4 (1873 mg, 5.64 mmol, 2.0 equiv) and PPh_3 (2958 mg, 11.28 mmol, 4.0 equiv) were combined in a flask which was evacuated and refilled with nitrogen (three cycles). CH_2Cl_2 (0.2 M) was added and the resulting solution was stirred for 10 minutes at room temperature. 1.0 equiv. of Benzaldehyde (0.3 g, 2.82 mmol) in CH_2Cl_2 (2 mL) was added to the above reaction mixture at 0 °C and stirred for 45 minutes, slowly the reaction mixture warming to room temperature. The solvent was removed under reduced pressure and the residue was dissolved in the minimum quantity of CH_2Cl_2. Hexane was added to the flask and quickly filtered through a pad of silica. This was repeated until all the contents had been transferred to the filter bed. The filter cake was washed with a hexane:Et_2O mixture (95:5, 200 mL), and the solvent was removed to give the as a yellow oil which was used for the next step.

1.5.2b. Procedure for synthesis of (bromoethynyl)benzene (S5)

(2,2-dibromovinyl)benzene (500 mg, 1.90 mmol, 1.0 equiv) was dissolved in 5.0 mL of dry DMF. Next, the schlenk tube was evacuated and filled with nitrogen (three cycles) and TBAF·$3H_2O$ (1.2 g, 3.80 mmol, TBAF·$3H_2O$ (1505 mg, 4.77 mmol, 2.5 equiv) was added to the solution and the seal tube was evacuated and filled with nitrogen (three cycles). The reaction mixture was heated at 60 °C for 1 h (TLC). The reaction mixture was cooled to room temperature and diluted with diethyl ether (50 mL). The organic phase was washed with water and brine, dried over anhydrous $MgSO_4$, filtered, and concentrated under reduced pressure. The crude product was purified by using silica gel column chromatography using n-hexane as eluent gave the desired yellow liquid in 72% (250 mg).

(Note: Other (bromoethynyl)benzene derivatives were obtained in a similar protocol based on availability of the starting material)

1.5.3. General procedure (A) for the preparation of 2-alkynyl-ynamides derivatives (1)

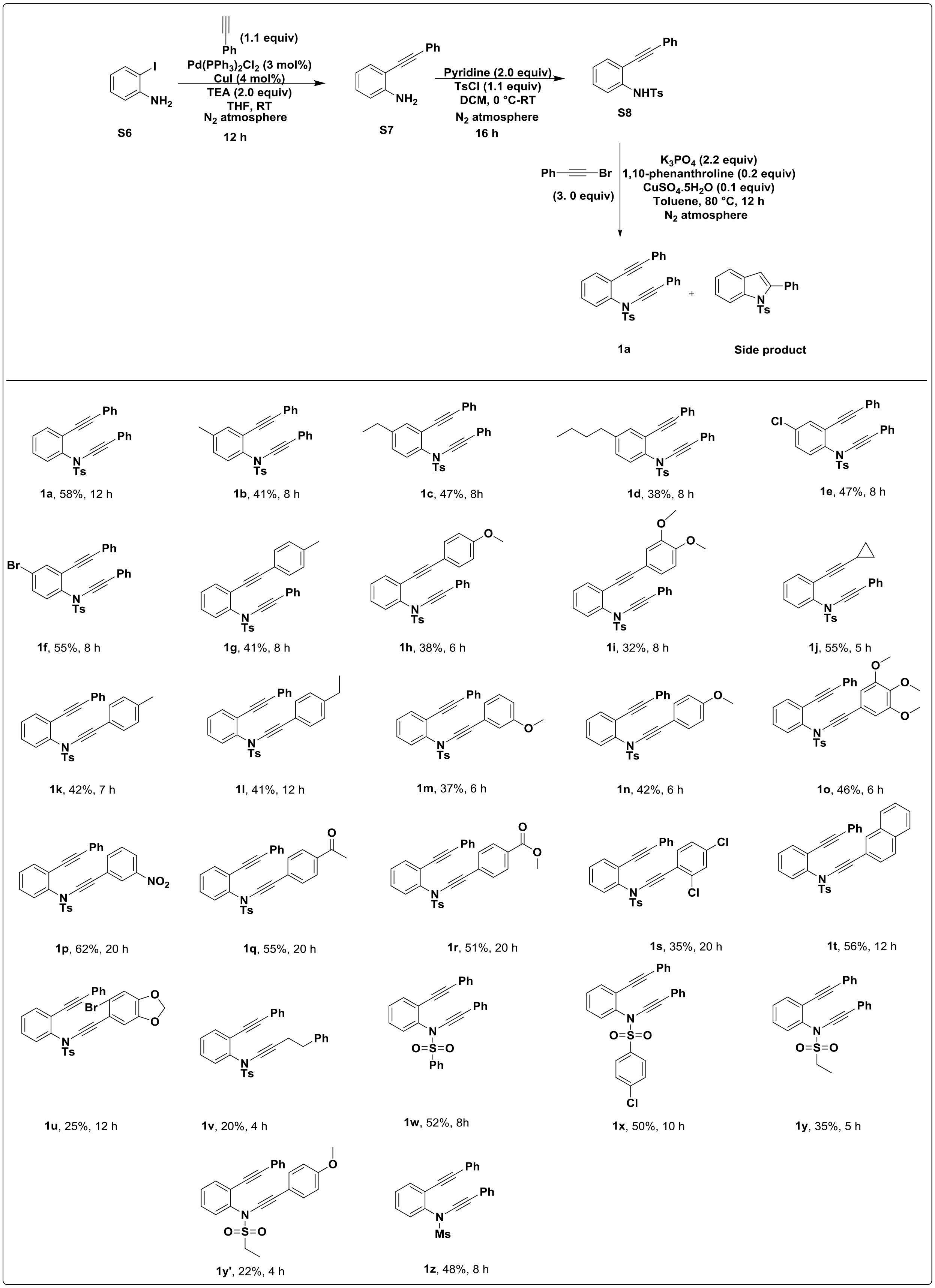

1.5.3.1. Procedure for the synthesis of 2-(phenylethynyl)aniline (S7)

To a dried schlenk flask equipped with a stir bar was charged with 2-iodoaniline (10 g, 45.65 mmol, 1.0 equiv) in THF. Next, the schlenk flask was evacuated and filled with nitrogen (three cycles), alkyne (5.12 g, 50.22 mmol, 1.1 equiv), freshly distilled Et_3N (9.24 g, 91.31 mmol, 2.0 equiv), $Pd(PPh_3)_2Cl_2$ (0.96 g, 1.3697 mmol, 3 mol%) and CuI (0.34 g, 1.82 mmol, 4 mol%) were added under the nitrogen atmosphere. The resulting mixture was stirred at room temperature for 12 h. After the completion of the reaction by TLC, the reaction mixture was cooled to room temperature, diluted with water, and extracted with ethyl acetate. The combined organic layer was dried over Na_2SO_4, filtered, and concentrated to give the crude material. The crude material was purified by column chromatography using hexane-ethyl acetate (95:5%) as the eluent gave yellow solid 2-(phenylethynyl)aniline in 85% (7.50 g).

1.5.3.2. Procedure for the synthesis of 4-methyl-*N*-(2-(phenylethynyl)phenyl)benzenesulfonamide (S8)

To a dried schlenk flask equipped with a stir bar was charged with 2-(phenylethynyl)aniline (7 g, 36.22 mmol, 1.0 equiv) in DCM at 0℃. Next, the schlenk flask was evacuated and filled with nitrogen (three cycles) followed by pyridine (5.73 g, 72.45 mmol, 2.0 equiv) and 4-methylbenzenesulfonyl chloride (7.59 g, 39.84 mmol, 1.1 equiv: The TsCl was added portionwise in 20 minutes) were added under nitrogen atmosphere. The resulting mixture was continued at room temperature for 16 h (if starting material was not consumed, heated at 40 ℃ (oil bath)). The solvent was removed under reduced pressure and the resulting solid was dissolved in DCM, washed with water and brine, and dried over $MgSO_4$. The crude material was purified by column chromatography using hexane-ethyl acetate (90:10%) as the eluent gave yellow solid 4-methyl-*N*-(2-(phenylethynyl)phenyl)benzenesulfonamide in 63% (8.23 g).

1.5.3.3. Procedure for the synthesis of 4-methyl-*N*-(phenylethynyl)-*N*-(2-(phenylethynyl)phenyl)benzenesulfonamide derivatives (1a)

To a dried flask was added 4-methyl-*N*-(2-(phenylethynyl)phenyl) (4 g, 11.52 mmol, 1.0 equiv), $CuSO_4{\cdot}5H_2O$ (288 mg, 1.15 mmol, 0.1 equiv), 1,10-phenanthroline (414 mg 2.30 mmol, 0.2 equiv), and K_3PO_4 (5.38 g, 25.36 mmol, 2.2 equiv) in dry toluene. Next, the flask was evacuated and filled with nitrogen (three cycles) followed by addition of bromoalkyne (6.25 g, 34.58 mmol, 3.0 equiv) (note: need to prepare freshly before performing the reaction) and the mixture was stirred at 80 ^{o}C 12 h (Note: need vigorous stirring). The resulting mixture was filtered through silica gel and then concentrated in vacuum. The residue was purified by flash column chromatography on silica gel (Formation of the ynamide and indole side products were very close on TLC, so need to pack long silica and long run with hexane-ethyl acetate (99:1). A pale-yellow solid was obtained and

washed with HPLC grade n-pentane gave the pure brown solid 4-methyl-*N*-(phenylethynyl)-*N*-(2-(phenylethynyl)phenyl)benzenesulfonamide (3 g, 58%).

Note: Other ynamide derivatives were obtained (250 mg scale of respective **S8** starting material) through the procedure reported above. Compounds known in the literature were confirmed by comparing their ^{1}H and ^{13}C NMR spectra.[1] The characterization data for unknown ynamides **1b -1z** were given.

1.5.4. Procedure (B) for the synthesis of 4-methyl-*N*-(phenylethynyl)-*N*-(3-phenylprop-2-yn-1-yl)benzenesulfonamide (1aa)[2,3]

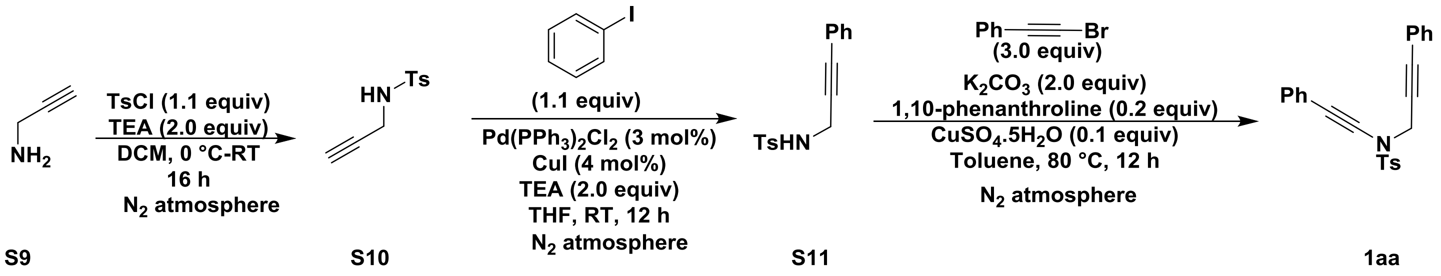

1.5.4.1 Procedure for the synthesis of 4-methyl-*N*-(prop-2-yn-1-yl) benzenesulfonamide (S10)

To a solution of prop-2-yn-1-amine (250 mg, 4.54 mmol, 1.0 equiv) in DCM at 0 °C, TEA (198 mg, 9.09 mmol, 2.0 equiv) and the schlenk flask was evacuated and filled with nitrogen (three cycles) followed by 4-methylbenzenesulfonyl chloride (953 mg, 5.00 mmol, 1.1 equiv) were added. Next, the schlenk flask was evacuated and filled with nitrogen (three cycles). The resulting mixture was continued at room temperature for 16 h. The solvent was removed under reduced pressure and the resulting solid was dissolved in ethyl acetate, washed with water and brine and dried over $MgSO_4$. The solvent was removed under reduced pressure and the crude product washed with pentane and carried out without further purification 4-methyl-*N*-(prop-2-yn-1-yl)benzenesulfonamide in 84% (800 mg) (**S10**).

1.5.4.2. Procedure for the synthesis of 4-methyl-*N*-(3-phenylprop-2-yn-1-yl)benzenesulfonamide (S11)

To a solution of 4-methyl-*N*-(prop-2-yn-1-yl)benzenesulfonamide (500 mg, 2.39 mmol, 1.0 equiv) in THF at room temperature, iodobenzene (534 mg, 2.63 mmol, 1.1 equiv) was added. Next, the schlenk flask was evacuated and filled with nitrogen (three cycles), followed by freshly distilled Et_3N (483 mg, 4.78 mmol, 2.0 equiv) followed by $Pd(PPh_3)_2Cl_2$ (50 mg, 0.07 mmol, 3 mol%) and CuI (18 mg, 0.09 mmol, 4 mol%) were added under a nitrogen atmosphere. The resulting mixture was stirred at room temperature for 12 h. After the completion of the reaction by TLC, the reaction mixture was cooled to RT, diluted with water, and extracted with ethyl acetate. The combined organic layer was dried over Na_2SO_4, filtered, and concentrated to give the

crude material. The crude material was purified by column chromatography using hexane/ethyl acetate (85:15) as the eluent and gave 4-methyl-*N*-(3-phenylprop-2-yn-1-yl)benzenesulfonamide in 66% (450 mg).

1.5.4.3. Procedure for the synthesis of 4-methyl-*N*-(phenylethynyl)-*N*-(3-phenylprop-2-yn-1-yl)benzenesulfonamide (1aa)

To a mixture of 4-methyl-*N*-(3-phenylprop-2-yn-1-yl)benzenesulfonamide (300 mg, 1.05 mmol, 1.0 equiv), $CuSO_4{\cdot}5H_2O$ (26 mg, 0.105 mmol, 0.1 equiv), 1,10-phenanthroline (38 mg, 0.210 mmol, 0.2 equiv) and K_2CO_3 (291 mg, 2.69 mmol, 2.0 equiv), dry toluene (4 mL). Next, the seal tube was evacuated and filled with nitrogen (three cycles), followed by (bromoethynyl)benzene (572 mg, 3.15 mmol, 3.0 equiv) were added. The vessel was stoppered under a nitrogen atmosphere and heated in an oil bath maintained at 80 °C for 12 h. The mixture was passed through celite and concentrated in a vacuum. The crude product was purified by using silica gel column chromatography using hexane/ethyl acetate (95:5) as eluent and gave the desired 4-methyl-*N*-(phenylethynyl)-*N*-(3-phenylprop-2-yn-1-yl)benzenesulfonamide as a brown solid in 69% (280 mg).

1.5.5. Procedure (B1) for the synthesis of 4-methyl-*N*-(4-phenylbut-3-yn-1-yl)-*N*-(phenylethynyl)benzenesulfonamide (1ab)[2,3,4]

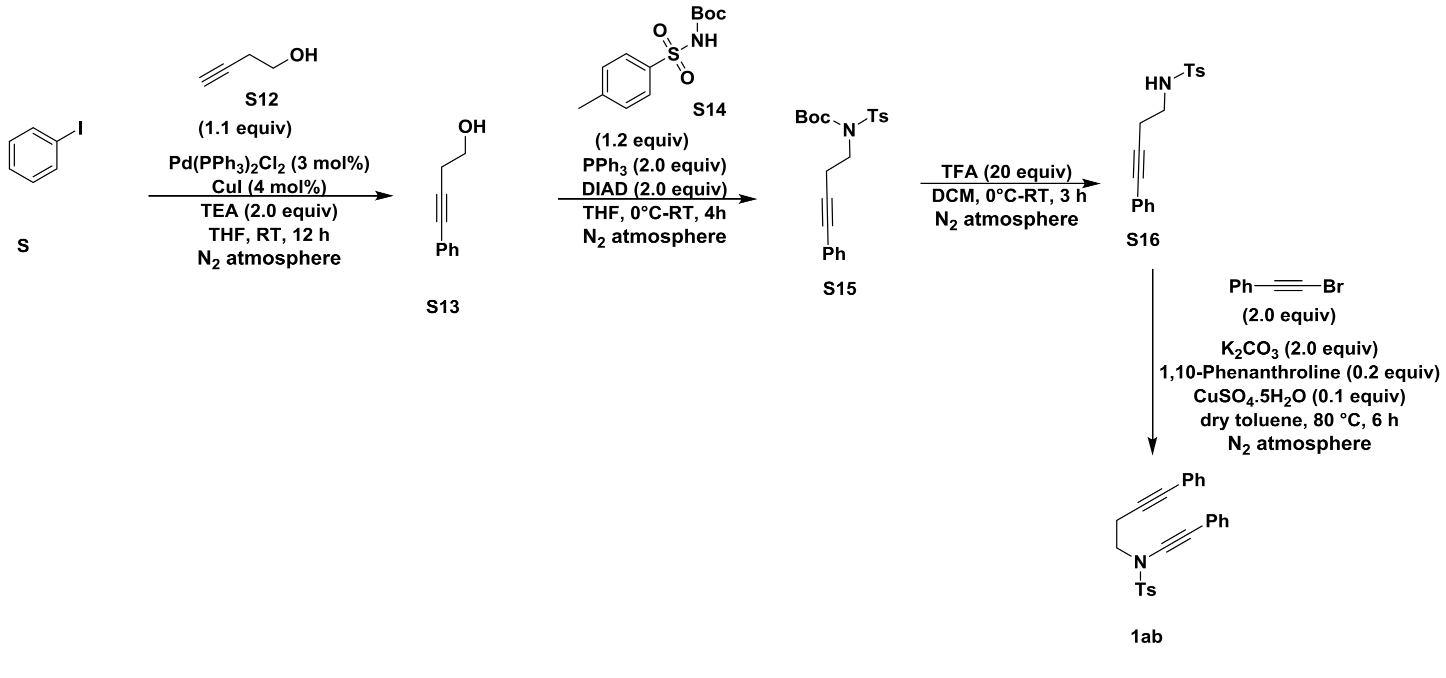

1.5.5.1. Procedure for the synthesis of 4-phenylbut-3-yn-1-ol (S13)

To a solution of but-3-yn-1-ol (567 mg, 8.08 mmol, 1.1 equiv) in THF at room temperature, iodobenzene (1.5 g, 7.35 mmol, 1.0 equiv), was added. Next, the schlenk flask was evacuated and filled with nitrogen (three cycles), followed by freshly distilled Et_3N (1.48 g, 14.70 mmol, 2.0 equiv) followed by $Pd(PPh_3)_2Cl_2$ (155 mg, 2.20 mmol, 3 mol%) and CuI (56 mg, 2.94 mmol, 4 mol%) were added under a nitrogen atmosphere. The resulting

mixture was stirred at room temperature for 12 h. After the completion of the reaction by TLC, the reaction mixture was cooled to RT, diluted with water, and extracted with ethyl acetate. The combined organic layer was dried over Na_2SO_4, filtered, and concentrated to give the crude material. The crude material was purified by column chromatography using hexane/ethyl acetate (80:20) as a eluent gave 4-phenylbut-3-yn-1-ol in 84% (900 mg).

1.5.5.2. Procedure for the synthesis of *tert*-butyl (4-phenylbut-3-yn-1-yl)(tosyl)carbamate (S15)

Tert-butyl tosylcarbamate (1.0 g, 3.69 mmol, 1.2 equiv) was dissolved in dry THF and triphenylphosphine (2.42 g, 9.24 mmol, 3.0 equiv) was added. Then the schlenk flask was evacuated and filled with nitrogen gas (three cycles), followed by addition of 4-phenylbut-3-yn-1-ol (450 mg, 3.08 mmol, 1.0 equiv) and diisopropyl azodicarboxylate (1.24 g, 1.16 mmol, 2.0 equiv). The mixture was stirred at room temperature for 4 h. After the completion of the reaction by TLC, the reaction mixture concentrated under reduced pressure and the product was purified by using silica gel column chromatography using hexane/ethyl acetate (88:12) as a eluent and gave the desired *tert*-butyl (4-phenylbut-3-yn-1-yl)(tosyl)carbamate in 69% yield (850 mg).

1.5.5.3. Procedure for the synthesis of 4-methyl-*N*-(4-phenylbut-3-yn-1-yl)benzenesulfonamide (S16)

To a solution of the *tert*-butyl (4-phenylbut-3-yn-1-yl)(tosyl)carbamate (850 mg, 2.13 mmol, 1.0 equiv) in CH_2Cl_2 (30 mL) was then added trifluoroacetic acid (4.85 g, 42.60, 20 equiv) at 0 ^oC under nitrogen atmosphere, and the mixture was stirred at room temperature for 3 h. The mixture was diluted with DCM, and the organic layer was washed with saturated $NaHCO_3$ solution and saturated NaCl solution, dried and concentrated to afford crude product. The crude product was purified by using silica gel column chromatography using hexane/ethyl acetate (85:15) as eluent and gave the desired 4-methyl-*N*-(4-phenylbut-3-yn-1-yl)benzenesulfonamide in 66% yield (420 mg) as a white solid.

1.5.5.4. Procedure for the synthesis of 4-methyl-*N*-(4-phenylbut-3-yn-1-yl)-*N*-(phenylethynyl)benzenesulfonamide (1ab)

To a mixture of 4-methyl-*N*-(4-phenylbut-3-yn-1-yl)benzenesulfonamide (250 mg, 0.83 mmol, 1.0 equiv), $CuSO_4{\cdot}5H_2O$ (21 mg, 0.083 mmol, 0.1 equiv), 1,10-phenanthroline (30 mg, 0.16 mmol, 0.2 equiv) and K_2CO_3 (291 mg, 1.62 mmol, 2.0 equiv), dry toluene (4 mL). Next, the seal tube was evacuated and filled with nitrogen (three cycles), followed by (bromoethynyl)benzene (303 mg, 1.67 mmol, 2.0 equiv) were added. The vessel was stoppered under a nitrogen atmosphere and heated in an oil bath maintained at 80 ℃ for 6 h. The mixture was passed through celite and concentrated in a vacuum. The crude product was purified by using silica gel column chromatography using hexane/ethyl acetate (93:7) as eluent. This gave the desired 4-methyl-*N*-(4-phenylbut-3-yn-1-yl)-*N*-(phenylethynyl)benzenesulfonamide as yellow gummy compound in 63% (220 mg). (Note: The

product is unstable (2-3 days) in the presence of trace amount of water (This starting material easily hydration happened on the ynamide alkyne).

1.5.6. Procedure for the preparation of sodium *p*-toluenesulfinate (S18)[5]

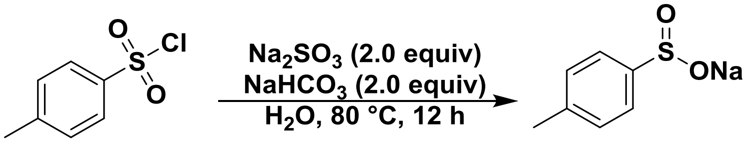

S17

S18

To a round-bottom flask was added 4-toluenesulfonyl chloride (2.0 g, 10.49 mmol, 1.0 equiv), was added to a stirred solution of sodium sulfite (2.64 g, 20.98 mmol, 2.0 equiv), and sodium bicarbonate (1.76 g, 20.98 mmol, 2.0 equiv) in H_2O. After being stirred at 80 °C for 4-12 h, then water was removed by a rotary evaporator. Then the remaining solid was extracted and recrystallized from ethanol to get the desired sodium p-toluenesulfinate (1.2 g, 64%) (Note: Oher sulfinates were obtained in a similar protocol)

1.5.7. Procedure for the preparation of 4-methylbenzenesulfonyl iodide (2a)[6,7]

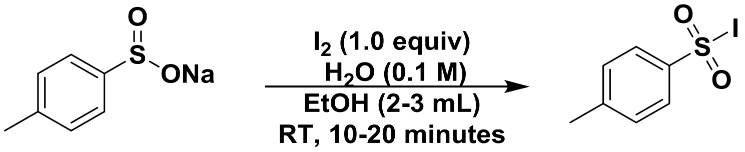

S18

2a

To a round-bottom flask (50 mL) was added sodium *p*-toluenesulfinate (200 mg, 1.12 mmol, 1.0 equiv) in distilled water at room temperature. A saturated solution of iodine (284 mg, 1.12 mmol, 1.0 equiv) in ethanol (2-3 mL) was prepared and added gradually to the sodium p-tolylsulfinate solution. During this addition period, yellow precipitates were formed gradually. The precipitates were filtered, washed with cold water, and dried carefully at room temperature to give p-toluenesulfonyl iodide as a yellow solid. (Note: The synthesized 4-methylbenzenesulfonyl iodide immediately used for next step, we belive the products containing a trace amount of water due to less time drying under high vacuum) (Note: Other sulfonyl iodides were obtained in a similar protocol).

1.5.8. Procedure for the preparation of 4-methylbenzenesulfonohydrazide (2ba)[8]

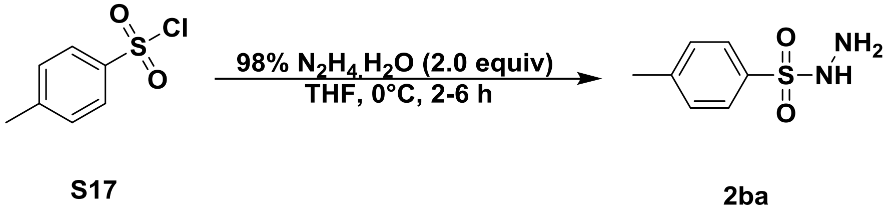

To an oven dried round-bottom flask (20 mL) was added hydrazine monohydrate (131 mg, 2.62 mmol, 2.0 mmol) dropwise to a solution of 4-toluenesulfonyl chloride (250 mg, 1.31 1.0 equiv) in tetrahydrofuran (20 mL) surrounded by an ice-water bath. The mixture was stirred for 2-6 h under a nitrogen atmosphere (detected by TLC). After the mixture was diluted with ethyl acetate, the solution was washed with saturated brine and the organic layer was dried with anhydrous magnesium sulfate. Then filtering the inorganic salt, and added slowly to stirred diethyl ether (40 mL) over 6 min. After being stirred for 15 min, the mixture was filtered, and the collected solid was dried in vacuum to gave 90% (220 mg) (Note: Other sulfonyl hydrazides were obtained in a similar protocol).

1.5.9. Procedure for the synthesis of 4-methylbenzenesulfonyl bromide (2ca)[9]

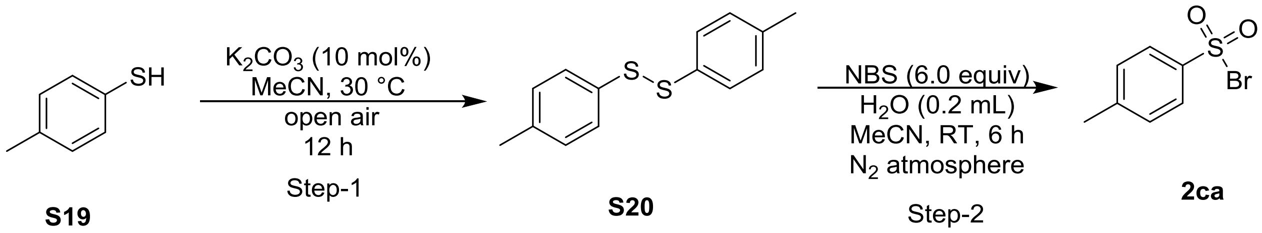

1.5.9.1. Procedure for the synthesis of 1,2-di-p-tolyldisulfane (S20) (step-1)

To an oven dried 25 mL round bottom flask with a magnetic bar was added K_2CO_3 (28 mg, 0.20 mmol, 0.1 equiv) and 4-methylbenzenethiol (250 mg, 2.01 mmol, 1.0 equiv) in MeCN (10 mL) were added. Then the reaction mixture was stirred at 30℃ under open air for 12 h and monitored by TLC. The solution was diluted with ethyl acetate (10 mL), and evaporated under vacuum. The residue was purified by column chromatography on silica gel (ethyl acetate/hexane-1:99) to afford product 1,2-di-p-tolyldisulfane in 82%.

1.5.9.2. Procedure for the synthesis of 4-methylbenzenesulfonyl bromide (2ca) (step-2)

In a 50 mL round bottom flask, 1,2-di-p-tolyldisulfane (200 mg, 0.81 mmol, 1.0 equiv) was dissolved in acetonitrile (10 mL) and water (0.2 mL) under N_2 atmosphere. Next, the round bottom flask was evacuated and filled with nitrogen (three cycles). The round bottom flask was covered with aluminum foil to shield it from

light. *N*-bromo succinimide (1067 mg, 4.87 mmol, 6.0 equiv) was added to the mixture under N_2 atmosphere, and the resulting mixture was stirred at room temperature for 6 h. Water (20 mL) was added and the resulting reaction mixture was extracted with ethyl acetate. The extract was washed with brine, dried over anhydrous magnesium sulfate, and evaporated. Chromatography on silica gel using n-hexane/ethyl acetate (95:5) as a eluent gave the 4-methylbenzenesulfonyl bromide (white solid) in 62%.

1.5.10. Synthesis of Se-phenyl 4-methylbenzenesulfonoselenoate (2da)[5]

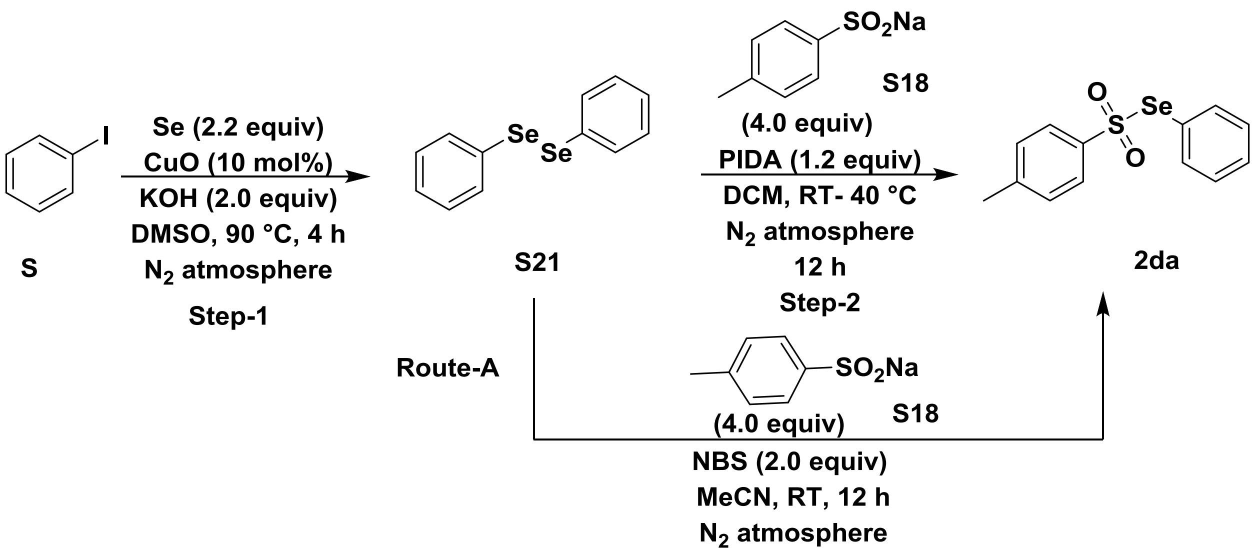

1.5.10.1. Procedure for the synthesis of 1,2-diphenyldiselane (S21) (Step-1)

To a stirred solution of Se metal (426 mg, 5.39 mmo, 2.2 equiv) and iodobenzene (500 mg, 2.45 mmol, 1.0 equiv) in DMSO (10 mL) was added CuO nanoparticles (20 mg, 0.245 mmol, 0.1 equiv). Next, the schlenk tube was evacuated and filled with nitrogen (three cycles), followed by KOH (4.90 mmol, 2.0 equiv) under nitrogen atmosphere. The resulting reaction mixture was stirred at 90 °C for 4 h. After the reaction was complete, the reaction mixture was allowed to cool, extracted with ethyl acetate. The combined organic layer was washed with brine, dried over Na_2SO_4, and concentrated in vacuo. The crude product was purified by column chromatography on silica gel (hexane:ethylacetae = 99:1) to gave 1,2-diphenyldiselane in 46% yield (**S21)**. (Caution: all selenium compounds have bad smell).

1.5.10.2. Procedure for the synthesis of Se-phenyl 4-methylbenzenesulfonoselenoate (2da) (Step-2)

A suspension of appropriate sodium p-toluenesulfinate (799 mg, 4.48 mmol, 4.0 equiv) in CH_2Cl_2 (30 mL) containing 1,2-diphenyldiselane (350 mg, 1.12 mmol, 1.0 equiv). Next, the schlenk tube was evacuated and filled with nitrogen (three cycles). Then reaction mixture was cooled at 0 °C and (diacetoxyiodo)benzene (433 mg, 1.34 mmol, 1.2 equiv) in DCM was added dropwise under nitrogen atmosphere. The mixture was stirred at room temperature to 40 °C for 12 h. The reaction mixture was washed with H_2O, dried over anhydrous Na_2SO_4.

The solvent CH_2Cl_2 was removed under reduced pressure and the residue was purified by column chromatography on silica gel (hexane:ethylacetae =95:5) to give Se-phenyl 4-methylbenzenesulfonoselenoate as a yellow solid in 53% (**2da**).

Alternative procedure for the synthesis of Se-phenyl 4-methylbenzenesulfonoselenoate (2da) (route-A)

A suspension of 1,2-diphenyldiselane (300 mg, 0.96 mmol, 1.0 equiv) in MeCN (30 mL) were added NBS (342 mg, 1.92 mmol, 2.0 equiv). The reaction solution was evacuated and filled with nitrogen (three cycles) and sodium p-toluenesulfinate (685 mg, 3.84 mmol, 4.0 equiv) was added under nitrogen atmosphere. Then the mixture was stirred at room temperature for 12 h. After the completion of the reaction by TLC, the reaction mixture was diluted with water, and extracted with ethyl acetate. The combined organic layer was dried over Na_2SO_4, filtered, and concentrated to give the crude material. The crude material was purified by column chromatography using hexane-ethyl acetate (95:5) as the eluent. It gave Se-phenyl 4-methylbenzenesulfonoselenoate as a yellow solid in 48% (**2da**).

(Note: Other sulfonoselenoates were obtained in a similar protocol).

1.5.11. Procedure (C) for the synthesis of *N*-(3-(4-methoxyphenyl)prop-2-yn-1-yl)-4-methyl-*N*-(2-(phenylethynyl)phenyl)benzenesulfonamide (S22)

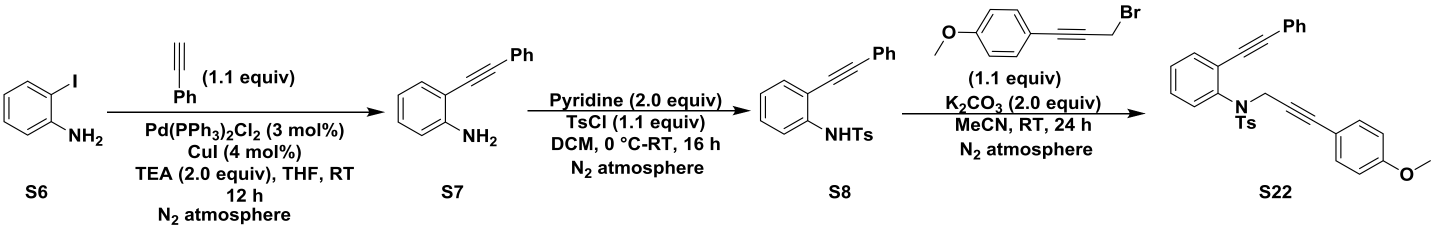

1.5.11.1. Procedure for the synthesis of 2-(phenylethynyl)aniline (S7)

To a solution of 2-iodoaniline (300 mg, 1.36 mmol, 1.0 equiv) in THF at room temperature. Next, the schlenk tube was evacuated and filled with nitrogen (three cycles). Phenylacetylene (154 mg, 1.50 mmol, 1.1 equiv), freshly distilled Et_3N (277 mg, 2.73 mmol, 2.0 equiv), $Pd(PPh_3)_2Cl_2$ (29 mg, 0.04 mmol, 3 mol%) and CuI (10 mg, 0.05 mmol, 4 mol%) were added under a nitrogen atmosphere. The resulting mixture was stirred at room temperature for 12 h. After the completion of the reaction by TLC, the reaction mixture was cooled to room temperature, diluted with water, and extracted with ethyl acetate. The combined organic layer was dried over Na_2SO_4, filtered, and concentrated to give the crude material. The crude material was purified by column chromatography using hexane-ethyl acetate (95:5%) as the eluent. It gave yellow solid 2-(phenylethynyl)aniline in 90% (240 mg).

1.5.11.2. Procedure for the synthesis of 4-methyl-*N*-(2-(phenylethynyl)phenyl)benzenesulfonamide (S8)

To a solution of 2-(phenylethynyl)aniline (200 mg, 1.03 mmol, 1.0 equiv) in DCM at 0℃, pyridine (164 mg, 2.07 mmol, 2.0 equiv). Next, the schlenk tube was evacuated and filled with nitrogen (three cycles) followed by p-methylbenzene sulfonyl chloride (217 mg, 1.13 mmol, 1.1 equiv) was added. The resulting mixture was continued at room temperature for 16 h (if starting material was not consumed, heated at 40 ℃ (oil bath)). The solvent was removed under reduced pressure and the resulting solid was dissolved in DCM, washed with water and brine, and dried over $MgSO_4$. The crude material was purified by column chromatography using hexane-ethyl acetate (90:10%) as the eluent. It gave yellow solid 4-methyl-*N*-(2-(phenylethynyl)phenyl)benzenesulfonamide in 69% (250 mg).

1.5.11.3. Procedure for the synthesis of *N*-(3-(4-methoxyphenyl)prop-2-yn-1-yl)-4-methyl-*N*-(2-(phenylethynyl)phenyl)benzenesulfonamide (S22)

To a solution of 4-methyl-*N*-(2-(phenylethynyl)phenyl)benzenesulfonamid (150 mg, 0.43 mmol, 1.0 equiv) in acetonitrile at room temperature, K_2CO_3 (119 mg, 0.86 mmol, 2.0 equiv). Next, the schlenk tube was evacuated and filled with nitrogen (three cycles) and 1-(3-bromoprop-1-yn-1-yl)-4-methoxybenzene (107 mg, 0.47 mmol, 1.1 equiv) were added to above reaction solution. The resulting mixture was continued at room temperature for 24 h. After the completion of the reaction by TLC (the product is same R_F with starting material), the reaction mixture was diluted with water, and extracted with ethyl acetate. The combined organic layer was dried over Na_2SO_4, filtered, and concentrated to give the crude material. The crude material was purified by column chromatography using hexane-ethyl acetate (93:7) as the eluent gave the desired product *N*-(3-(4-methoxyphenyl)prop-2-yn-1-yl)-4-methyl-*N*-(2-(phenylethynyl)phenyl)benzenesulfonamide in 60% (140 mg).

1.5.12. Procedure (A1) for Synthesis of 4-methyl-*N*-(phenylethynyl-2-^{13}C)-*N*-(2-(phenylethynyl)phenyl)benzenesulfonamide (1a‘)

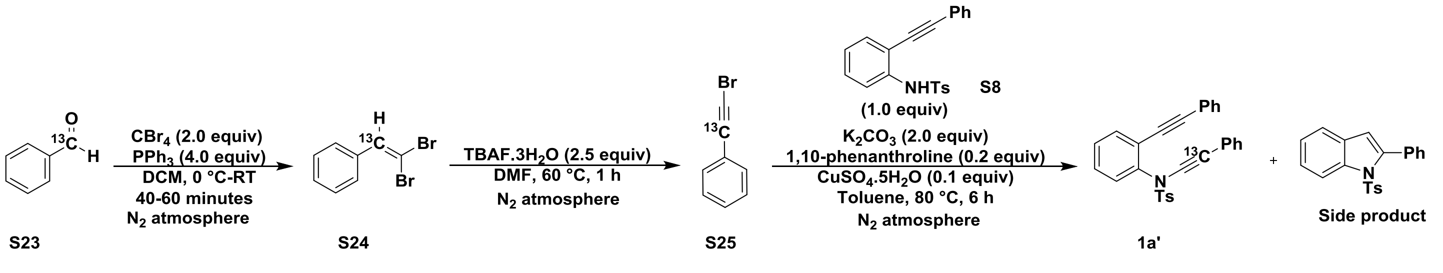

1.5.12.1. Procedure for the synthesis of ^{13}C- (2,2-dibromovinyl)benzene (S24)

CBr_4 (1548 mg, 4.66 mmol, 2.0 equiv) and PPh_3 (2448 mg, 9.33 mmol, 4.0 equiv) were combined in a flask which was evacuated and refilled with nitrogen (three cycles). CH_2Cl_2 (0.2 M) was added and the resulting

solution was stirred for 10 minutes at room temperature. 1.0 equiv. of ^{13}C-Benzaldehyde (0.25 g, 2.33 mmol) in CH_2Cl_2 (5 mL) was added at 0 °C and stirred for 45 minutes, slowly warming to room temperature. The solvent was removed under reduced pressure and the residue was dissolved in a minimum quantity of CH_2Cl_2. Hexane was added to the flask and quickly filtered through a pad of silica. This was repeated until all the contents had been transferred to the filter bed. The filter cake was washed with a Hexane:Et_2O mixture (95:5, 200 mL) and the solvent removed to give the ^{13}C-(2,2-dibromovinyl)benzene (**S24**) (65%) as a yellow oil which was used for the next step without purification.

1.5.12.2. Procedure for the synthesis of ^{13}C-1-(2-Bromoethynyl)benzene (S25)

^{13}C-(2,2-dibromovinyl)benzene (**S24**) (400 mg, 1.52 mmol, 1.0 equiv) was dissolved in 4.0 mL of dry DMF. Next, the schlenk tube was evacuated and filled with nitrogen (three cycles) and TBAF·$3H_2O$ (1.2 g, 3.80 mmol, 2.5 equiv) was added to the solution and the reaction mixture was heated at 60 °C for 1 h (TLC) under atmosphere. The reaction mixture was cooled to room temperature and diluted with diethyl ether (50 mL). The organic phase was washed with water and brine, dried over anhydrous MgSO4, filtered, and concentrated under reduced pressure. The crude product was purified by using silica gel column chromatography using n-hexane as eluent gave the desired yellow color ^{13}C- labeled 1-(2- Bromoethynyl)benzene in 85% (235 mg).

1.5.12.3. Procedure for the synthesis of 4-methyl-*N*-(phenylethynyl-2-^{13}C)-*N*-(2-(phenylethynyl)phenyl)benzenesulfonamide (1a')

To a mixture of 4-methyl-*N*-(2-(phenylethynyl)phenyl)benzenesulfonamide (200 mg, 0.57 mmol, 1.0 equiv), $CuSO_4·5H_2O$ (26 mg, 0.05 mmol, 0.1 equiv), 1,10-phenanthroline (21 mg, 0.11 mmol, 0.2 equiv) and K_2CO_3 (159 mg, 1.15 mmol, 2.0 equiv), dry toluene (5.0 mL). Next, the seal was evacuated and filled with nitrogen (three cycles). Then ^{13}C-labeled 1-(2-Bromoethynyl)benzene (210 mg, 1.15 mmol, 2.0 equiv) were added. The tube was stoppered under a nitrogen atmosphere and heated on an oil bath maintained at 80 °C overnight. The mixture was passed through celite and concentrated in a vacuum. The crude product was purified by using silica gel column chromatography using n-hexane and ethyl acetate (98:2) as eluent. It gave the desired 4-methyl-*N*-(phenylethynyl-2-^{13}C)-*N*-(2-(phenylethynyl)phenyl)benzenesulfonamide (**1a'**) as a brown solid in 49% (125 mg).

1.6. Reaction optimization for compound 76

Supplementary Table 1. Screening of reaction conditions[a]

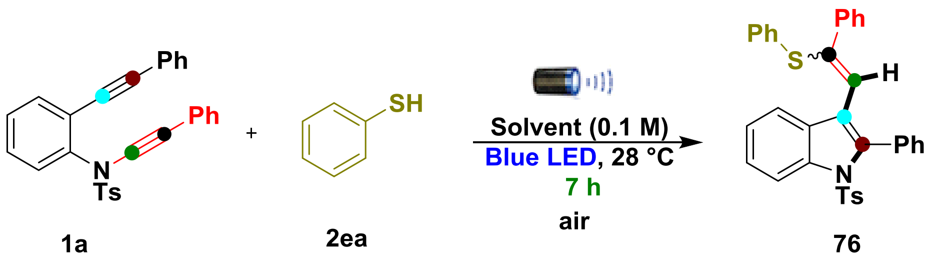

Entry	Radical initiator	Time (h)	Solvent	Yield % (76)[b]
1	Blue LED	7	DCM	Trace
2	Blue LED	7	DMSO	NR
3	Blue LED	7	Toluene	38
4	Blue LED	7	EtOH	Trace
4	Blue LED	7	THF	<5
5	Blue LED	7	Cyclohexanone	15
6	Blue LED	7	MeCN	55
7[c]	Blue LED	9	MeCN	45
8[d]	Blue LED	7	MeCN	38
9[e]	-	15	MeCN	40

[a]Reaction conditions, unless otherwise noted: **1a** (1.0 equiv), thiophenol (**2ea**) (2.5 equiv), and MeCN (0.1 M) were stirred at 28 °C under 40 W blue LED for 7 h. [b]Isolated yields. [c]0.05 M MeCN was used. [d]1.5 equiv of thiophenol was used. [e]Stirred at room temperature for 15 h. (Note: <5-10% unknown compound is forming but still the structure of the compound is not yet confirmed at present).

We commenced our radical strategy by using thiophenol (**2ea**) as a radical precursor with 4-methyl-*N*-(phenylethynyl)-*N*-(2-(phenylethynyl)phenyl)benzenesulfonamide (**1a**) in a DCM solvent under 40 W blue light-emitting diode (LED) light irradiation. But, to our disappointment, we observe only a trace amount of product **76** (Supplementary Table 1, entry 1). An extensive solvent screening process lead to these optimized reaction conditions (Supplementary Table 1, entries 2-6). Such conditions enabled the production of the desired product at maximum yield (55%) under MeCN solvent within 7 h. We assume that polar aprotic solvent acetonitrile would stabilize the radical species (thiyl) which may lower the activation energy and faster the reaction to produce the product. Encouraged by this finding, we altered the solvent molarity ratio from 0.1 M to

0.05 M, but the subsequent reaction failed to improve the yields (Supplementary Table 1, entry 7). Then, the equivalence of compound 2ea was altered but failed to improve the yield (Supplementary Table 1, entry, 8). We carried out the reaction in the absence of a light source (at room temperature) but this reaction did not improve the reaction yield (Table 1, entry 9).

1.7. Experimental procedures

1.7.1. General procedure (D) for the synthesis of (*E*)-3-(1-iodo-2-phenyl-2-tosylvinyl)-2-phenyl-1-tosylindole derivatives (3, 5-27, 28, 30, 41,43-45, 98)

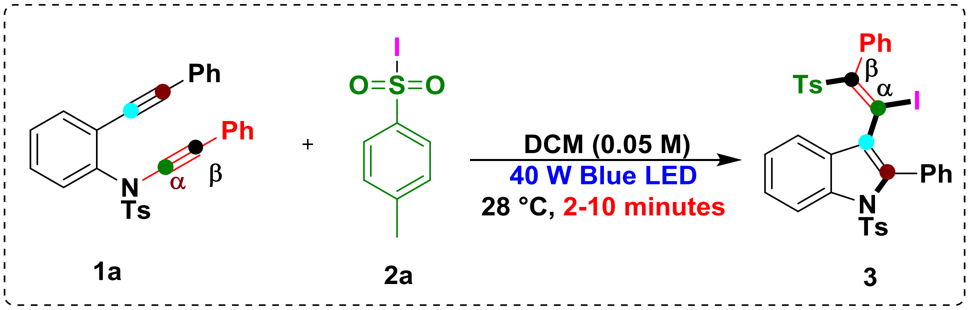

An oven-dried screw-capped, 8 mL vial equipped with a magnetic stir bar was charged with 4-methyl-*N*-(phenylethynyl)-*N*-(2-(phenylethynyl)phenyl)benzenesulfonamide (45 mg, 0.10 mmol, 1.0 equiv), 4-methylbenzenesulfonyl iodide (31 mg, 0.11 mmol, 1.1 equiv), and DCM (0.05 M) solvent was added. The resulting solution was stirred up to starting material completion (2-10 minutes) at 28 °C under a blue LED light (the reaction mixture vial was placed ~8.5 cm away from the LED light with a clip fan for cooling). After that, the crude reaction mixture was diluted with water and extracted with DCM. The organic layer was dried over Na_2SO_4, filtered, and concentrated. The crude material was purified by flash column chromatography using hexane-ethyl acetate (84:16) as the eluent gave the desired product (*E*)-3-(1-iodo-2-phenyl-2-tosylvinyl)-2-phenyl-1-tosylindole as a white solid in 85% (63 mg).

1.7.2. General procedure (E) for synthesis of (*E*)-3-(1-iodo-2-((4-methoxyphenyl)sulfonyl)-2-phenylvinyl)-2-phenyl-1-tosylindole derivatives (29, 31-40 and 42)

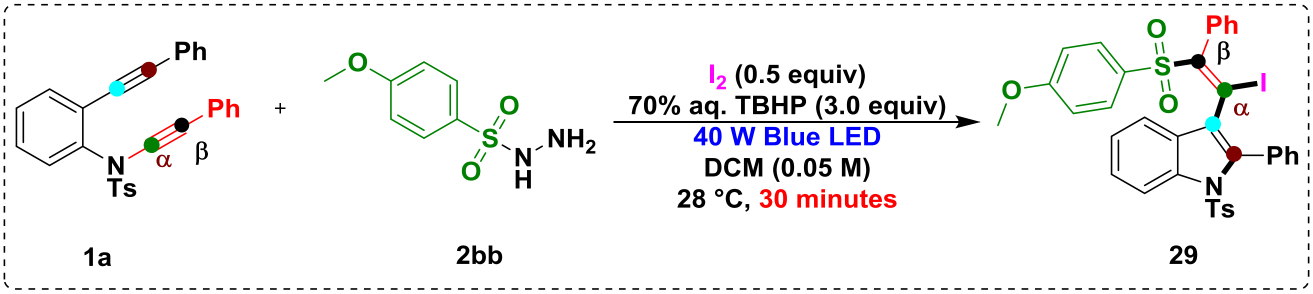

An oven-dried screw-capped 8 mL vial equipped with a magnetic stir bar was charged with 4-methyl-*N*-(phenylethynyl)-*N*-(2-(phenylethynyl)phenyl)benzenesulfonamide (45 mg, 0.10 mmol, 1.0 equiv) 4-

methoxybenzenesulfonohydrazide (31 mg, 0.15 mmol, 1.5 equiv) in DCM (0.05 M), I_2 (13 mg, 0.05 mmol, 0.5 equiv), aq. 70% TBHP (39 mg (42 µL) 0.30 mmol, 3.0 equiv) was added. The resulting solution was stirred up to starting material completion (30 minutes) at 28 °C under a blue LED light (the reaction mixture vial was placed ~8.5 cm away from the LED light with a clip fan for cooling). After that, the crude reaction mixture was diluted with water and extracted DCM. The crude material was purified by flash column chromatography using hexane-ethyl acetate (84:16) as the eluent gave the desired product (*E*)-3-(1-iodo-2-((4-methoxyphenyl)sulfonyl)-2-phenylvinyl)-2-phenyl-1-tosylindole as a white solid in 82% (61 mg).

1.7.3. General procedure (F) for synthesis of (*E*)-3-(1-bromo-2-phenyl-2-tosylvinyl)-2-phenyl-1-tosylindole (46 and 47)

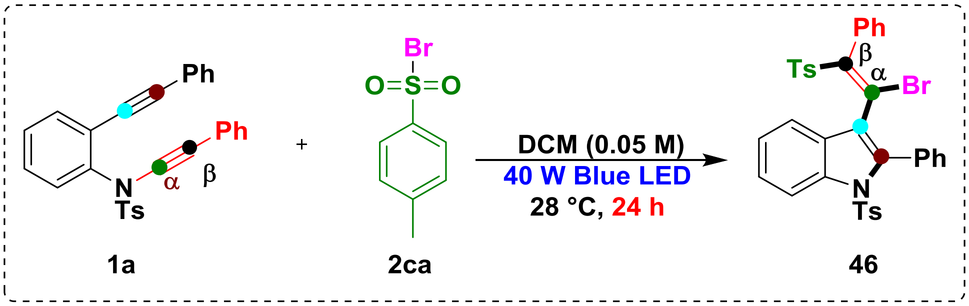

An oven-dried screw-capped, 8 mL vial equipped with a magnetic stir bar was charged with 4-methyl-*N*-(phenylethynyl)-*N*-(2-(phenylethynyl)phenyl)benzenesulfonamide (45 mg, 0.10 mmol, 1.0 equiv), 4-methylbenzenesulfonyl bromide (26 mg, 0.110 mmol, 1.1 equiv), and DCM (0.05 M) solvent was added. The resulting solution was stirred up to starting material completion (24 h) at 28 °C under a blue LED light (the reaction mixture vial was placed ~8.5 cm away from the LED light with a clip fan for cooling). After that, the crude reaction mixture was diluted water and extracted with DCM. The organic layer was dried over Na_2SO_4, filtered, and concentrated. The crude material was purified by flash column chromatography using hexane-ethyl acetate (84:16) as the eluent gave the desired product (*E*)-3-(1-bromo-2-phenyl-2-tosylvinyl)-2-phenyl-1-tosylindole as a lite yellow solid in 52% (36 mg).

1.7.4. General procedure (G) for the synthesis of (*E*)-2-phenyl-1-(2-phenyl-1-tosylindol-3-yl)-2-(phenylsulfonyl)vinyl benzenesulfonate derivatives (4, 48 and 49)

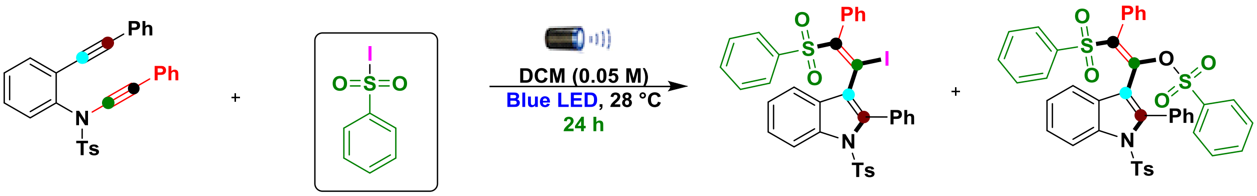

1a, 0.1 mmol | **2b**, 0.11 mmol | **28** | **48**

An oven-dried screw-capped, 8 mL vial equipped with a magnetic stir bar was charged with 4-methyl-*N*-(phenylethynyl)-*N*-(2-(phenylethynyl)phenyl)benzenesulfonamide (45 mg, 0.10 mmol, 1.0 equiv), benzenesulfonyl iodide (30 mg, 0.11 mmol, 1.1 equiv), and DCM (0.05 M) solvent was added. The resulting solution was stirred up to starting material completion (24 h) at 28 °C under a blue LED light (the reaction mixture vial was placed ~8.5 cm away from the LED light with clip a fan for cooling). After that, the crude reaction mixture was diluted with water and extracted with DCM. The organic layer was dried over Na_2SO_4, filtered, and concentrated. The crude material was purified by flash column chromatography using hexane-ethyl acetate (84:16), compound **28** and (85:15) compound **48** as the eluent gave the desired product **28** (61%, 44 mg) and ((*E*)-2-phenyl-1-(2-phenyl-1-tosylindol-3-yl)-2-(phenylsulfonyl)vinyl **48** as a white solid in 10% (8 mg).

(Or)

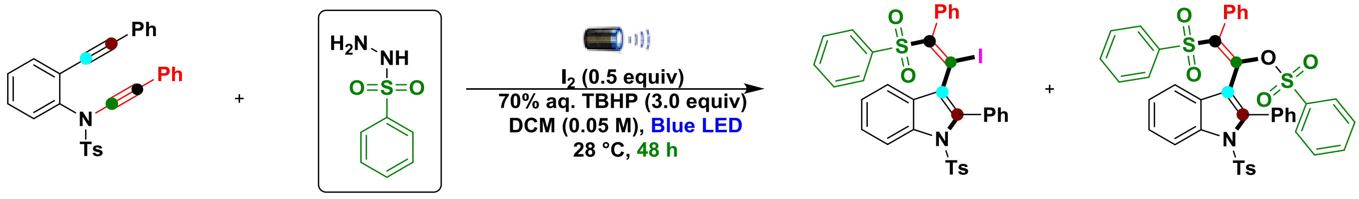

1a, 0.1 mmol **2bc**, 0.25 mmol **28** **48**

An oven-dried screw-capped 8 mL vial equipped with a magnetic stir bar was charged with 4-methyl-*N*-(phenylethynyl)-*N*-(2-(phenylethynyl)phenyl)benzenesulfonamide (45 mg, 0.10 mmol, 1.0 equiv) benzenesulfonohydrazide (43 mg, 0.15 mmol, 2.5 equiv) in DCM (0.05 M), I_2 (13 mg, 0.05 mmol, 0.5 equiv), aq. 70% TBHP (39 mg (42 μL) 0.30 mmol, 3.0 equiv) was added. The resulting solution was stirred up 48 h at 28 °C under a blue LED light (the reaction mixture vial was placed ~8.5 cm away from the LED light with clip a fan for cooling). After that, the crude reaction mixture was diluted with water and extracted DCM. The crude material (mixture of **28** and **48**) was purified by flash column chromatography using hexane-ethyl acetate (84:16) as the eluent gave the product (*E*)-3-(1-iodo-2-phenyl-2-(phenylsulfonyl)vinyl)-2-phenyl-1-tosylindole **28** as white solid in 58% (42 mg) and hexane-ethyl acetate (82:18) as the eluent gave the product (*E*)-2-phenyl-1-(2-phenyl-1-tosylindol-3-yl)-2-(phenylsulfonyl)vinyl benzenesulfonate **48** as a white solid in 8% (6 mg).

1.7.5. General procedure (H) for synthesis of (*E*)-2-phenyl-3-(2-phenyl-1-(phenylselanyl)-2-tosylvinyl)-1-tosylindole derivatives (51-75)

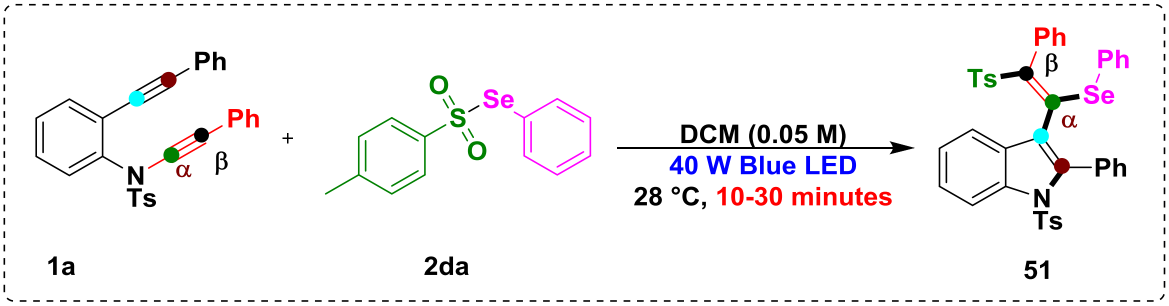

An oven-dried screw-capped, 8 mL vial equipped with a magnetic stir bar was charged with 4-methyl-N-(phenylethynyl)-N-(2-(phenylethynyl)phenyl)benzenesulfonamide (45 mg, 0.10 mmol, 1.0 equiv), Se-phenyl 4-methylbenzenesulfonoselenoate (26 mg, 0.11 mmol, 1.1 equiv), and DCM (0.05 M) solvent was added. The resulting solution was stirred up to starting material completion (10-30 minutes) at 28 °C under a blue LED light (the reaction mixture vial was placed ~8.5 cm away from the LED light with a clip fan for cooling). After that, the crude reaction mixture was diluted water and extracted with DCM. The organic layer was dried over Na_2SO_4, filtered, and concentrated. The crude material was purified by flash column chromatography using hexane-ethyl acetate (84:16) as the eluent gave the desired product (*E*)-2-phenyl-3-(2-phenyl-1-(phenylselanyl)-2-tosylvinyl)-1-tosylindole as a yellow solid in 68% (51 mg).

1.7.6. General procedure (I) for synthesis of (*E*/*Z*)-2-phenyl-3-(2-phenyl-2-(phenylthio)vinyl)-1-tosylindole derivatives (76-85)

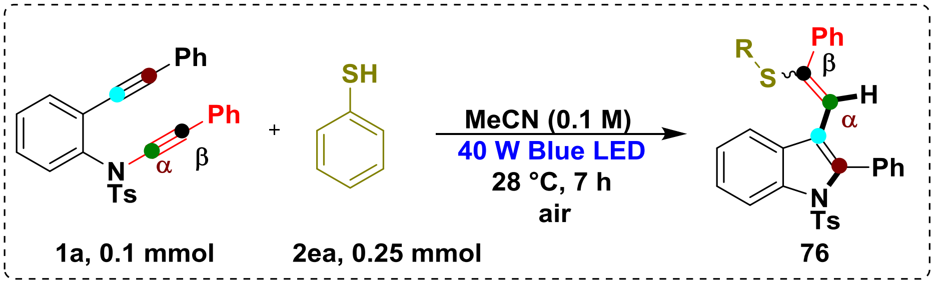

An oven-dried screw-capped, 8 mL vial equipped with a magnetic stir bar was charged with 4-methyl-*N*-(phenylethynyl)-*N*-(2-(phenylethynyl)phenyl)benzenesulfonamide (45 mg, 0.10 mmol, 1.0 equiv) aromatic thiols (28 mg, 0.25 mmol, 2.5 equiv), MeCN (0.1 M) was added. The resulting solution was stirred under a blue LED light (the reaction mixture vial was placed ~8.5 cm away from the LED light with a clip fan for cooling) up to starting material completion at 28 °C. After that, the crude reaction mixture was diluted with water and extracted with ethyl acetate. The organic layer was dried over Na_2SO_4, filtered, and concentrated. The crude material was purified by flash column chromatography using hexane-ethyl acetate (84:16) as the eluent gave the

desired product (*E*/*Z*)-2-phenyl-3-(2-phenyl-2-(phenylthio)vinyl)-1-tosylindole derivatives as a yellow solid in 55% (51 mg).

1.7.7. Reactions on a larger scale

1.7.7.1. Larger scale synthesis of (*E*)-3-(1-iodo-2-phenyl-2-tosylvinyl)-2-phenyl-1-tosylindole (3)

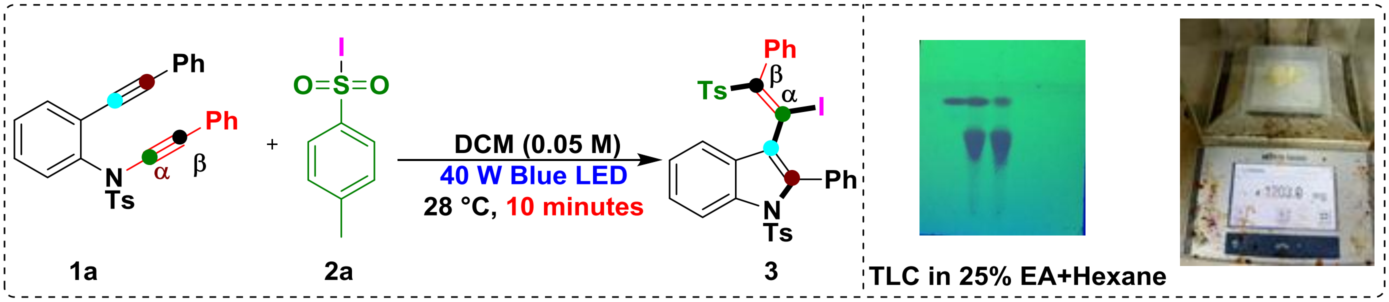

An oven-dried screw-capped 100 mL vial equipped with a magnetic stir bar was charged with 4-methyl-N-(phenylethynyl)-*N*-(2-(phenylethynyl)phenyl)benzenesulfonamide (1.0 g, 2.23 mmol, 1.0 equiv), 4-methylbenzenesulfonyl iodide (0.694 g, 2.46 mmol, 1.1 equiv), and DCM (0.05 M) solvent was added. The resulting solution was stirred up starting material completion (10 minutes) at 28 ℃ under a 40 W blue LED light (the reaction mixture vial was placed ~8.5 cm away from the LED light with a clip fan for cooling). After that, the crude reaction mixture was diluted with water and extracted with DCM. The organic layer was dried over Na_2SO_4, filtered, and concentrated. The crude material was purified by flash column chromatography using hexane-ethyl acetate (82:18) as the eluent gave the desired product (*E*)-3-(1-iodo-2-phenyl-2-tosylvinyl)-2-phenyl-1-tosylindole as a white solid in 74% (1.203 g).

1.7.7.2. Larger scale synthesis of (*E*)-2-phenyl-3-(2-phenyl-1-(phenylselanyl)-2-tosylvinyl)-1-tosylindole (51)

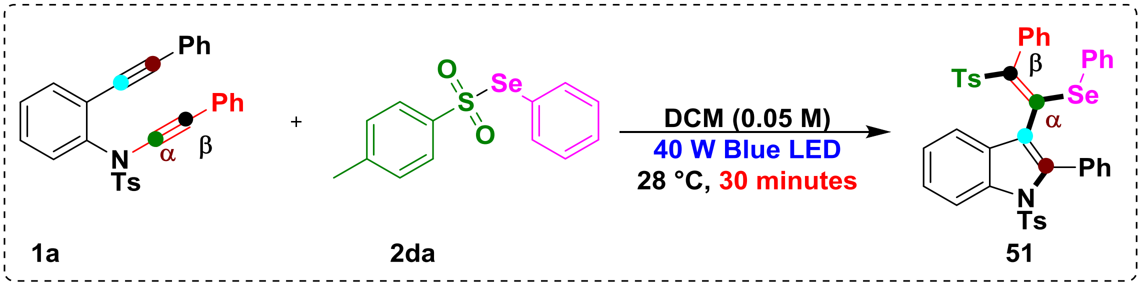

An oven-dried screw-capped, 25 mL vial equipped with a magnetic stir bar was charged with 4-methyl-*N*-(phenylethynyl)-*N*-(2-(phenylethynyl)phenyl)benzenesulfonamide (0.25g scale, 0.55 mmol, 1.0 equiv), Se-phenyl 4-methylbenzenesulfonoselenoate (192 mg, 0.61 mmol, 1.1 equiv), and DCM (0.05 M) solvent was added. The resulting solution was stirred up to starting material completion (30 minutes) at 28 ℃ under a blue LED light (the reaction mixture vial was placed ~8.5 cm away from the LED light with a clip fan for cooling).

After that, the crude reaction mixture was diluted with water and extracted with DCM. The organic layer was dried over Na_2SO_4, filtered, and concentrated. The crude material was purified by flash column chromatography using hexane-ethyl acetate (84:16) as the eluent gave the desired product (*E*)-2-phenyl-3-(2-phenyl-1-(phenylselanyl)-2-tosylvinyl)-1-tosylindole as a yellow solid in 60% (255 mg).

1.7.7.3. Larger scale synthesis of 2-phenyl-3-(2-phenyl-2-(phenylthio)vinyl)-1-tosylindole (76)

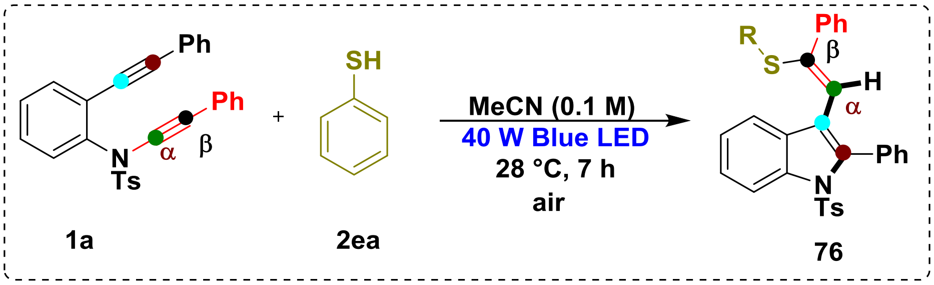

In an oven-dried screw-capped, 25 mL vial equipped with a magnetic stir bar was charged with 4-methyl-*N*-(phenylethynyl)-*N*-(2-(phenylethynyl)phenyl)benzenesulfonamide (0.25g scale, 0.55 mmol, 1.0 equiv) benzenethiol (154, 1.39 mmol, 2.5 equiv) in MeCN (0.1 M) was added. The resulting solution was stirred up to starting material completion (7 h) at 28 °C under a blue LED light (the reaction mixture vial was placed ~8.5 cm away from the LED light with a clip fan for cooling). After that, the crude reaction mixture was diluted with water and extracted with ethyl acetate. The organic layer was dried over Na_2SO_4, filtered, and concentrated. The crude material was purified by flash column chromatography using hexane-ethyl acetate (90:10) as the eluent gave the desired product 2-phenyl-3-(2-phenyl-2-(phenylthio)vinyl)-1-tosylindole as a yellow solid in 46% (145 mg).

1.7.8. Synthetic transformations of (*E*)-3-(1-iodo-2-phenyl-2-tosylvinyl)-2-phenyl-1-tosylindole

1.7.8.1. Procedure (J) for synthesis of (*Z*)-2-phenyl-3-(2-phenyl-1-(p-tolyl)-2-tosylvinyl)-1-tosylindole (86)

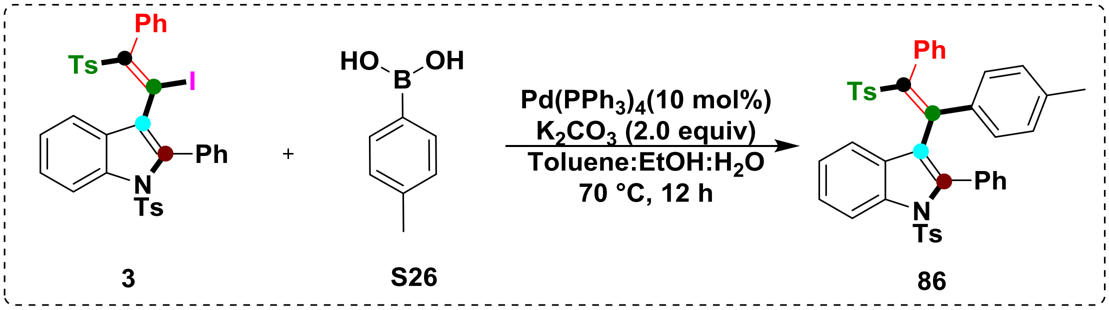

An overnight dried seal tube (15 mL) was charged with (*E*)-3-(1-iodo-2-phenyl-2-tosylvinyl)-2-phenyl-1-tosylindole (**3**) (70 mg, 0.09 mmol, 1.0 equiv), p-tolylboronic acid (17 mg, 0.12 mmol, 1.3 equiv), $Pd(PPh_3)_4$ (11 mg, 0.01 mmol, 10 mol%), K_2CO_3 (27 mg, 0.19 mmol, 2.0 equiv) in Toluene:EtOH:H_2O (5:2:1) (2 ml) under N_2 atmosphere. The resulting mixture was stirred at 70 °C for 12 h. After the completion of the reaction

by TLC, the reaction mixture was cooled to room temperature, diluted with water, and extracted with ethyl acetate. The organic layer dried over anhydrous Na_2SO_4, filtered, and concentrated to give the crude material. The crude material was purified by column chromatography using hexane-ethyl acetate (85:15) as the eluent to afford 2-phenyl-3-(2-phenyl-1-(p-tolyl)-2-tosylvinyl)-1-tosylindole in 61% (40 mg) (**86)**.

1.7.8.2. Procedure (K) for the synthesis of 2-phenyl-3-(phenylethynyl)-1-tosylindole(87)

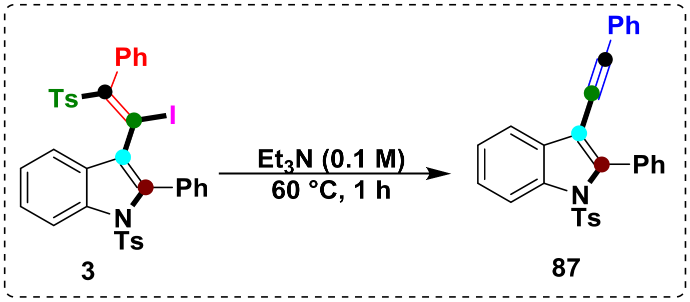

An overnight dried seal tube (15 mL) was charged with (*E*)-3-(1-iodo-2-phenyl-2-tosylvinyl)-2-phenyl-1-tosylindole (70 mg, 0.09 mmol, 1.0 equiv), Et_3N (0.1 M) under N_2 atmosphere. The resulting mixture was stirred at 60 ℃ for 1 h. After the completion of the reaction by TLC, the reaction mixture was cooled to room temperature, diluted with water, and extracted with ethyl acetate. The organic layer dried over anhydrous Na_2SO_4, filtered, and concentrated to give the crude material. The crude material was purified by column chromatography using hexane-ethyl acetate (95:5) as the eluent to afford 2-phenyl-3-(phenylethynyl)-1-tosylindole in 58% (24 mg) (**87)**.

1.7.8.3. Procedure (L) for the synthesis of 2-phenyl-3-(2-phenyl-2-tosylvinyl)-1-tosylindole (88)

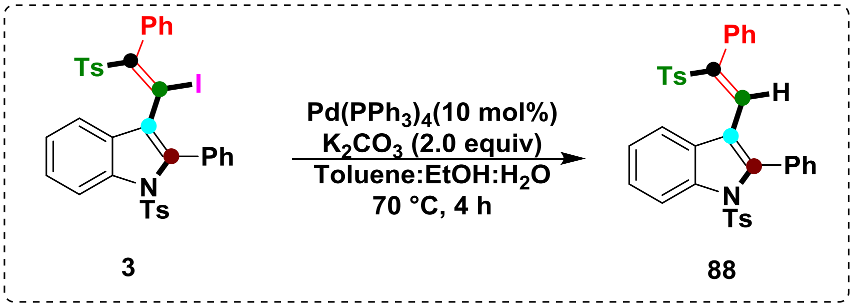

An overnight dried seal tube (15 mL) was charged with (*E*)-3-(1-iodo-2-phenyl-2-tosylvinyl)-2-phenyl-1-tosylindole (**3**) (70 mg, 0.09 mmol, 1.0 equiv), $Pd(PPh_3)_4$ (11 mg, 0.01 mmol, 10 mol%), K_2CO_3 (27 mg, 0.19 mmol, 2.0 equiv) in Toluene:EtOH:H_2O (5:2:1) (2 mL) under N_2 atmosphere. The resulting mixture was stirred at 70 ℃ for 4 h. After the completion of the reaction by TLC, the reaction mixture was cooled to room temperature, diluted with water, and extracted with ethyl acetate. The organic layer dried over anhydrous Na_2SO_4, filtered, and concentrated to give the crude material. The crude material was purified by column

chromatography using hexane-ethyl acetate as the eluent (85:16) to afford 2-phenyl-3-(2-phenyl-1-(p-tolyl)-2-tosylvinyl)-1-tosylindole 54% (32 mg) (**88**).

1.7.8.4. Procedure (M) for the synthesis of 2-(phenylethynyl)tetrahydrofuran (102)

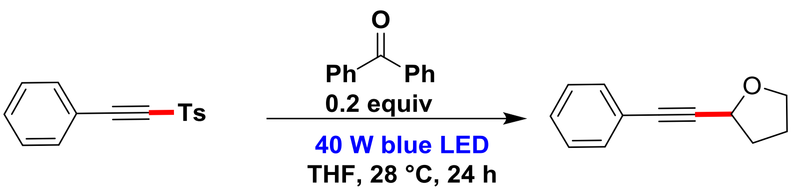

101 **102**

In an oven-dried screw-capped (8 mL) was charged with (1-methyl-4-((phenylethynyl)sulfonyl)benzene (**101**) (50 mg, 0.19 mmol, 1.0 equiv), benzophenone (11 mg, 0.03 mmol, 0.2 equiv), in THF (2 mL) under N_2 atmosphere. The resulting solution was stirred at 28 ℃ under a blue LED light (the reaction mixture vial was placed ~8.5 cm away from the LED light with a clip fan for cooling) for 24 h. After the completion of the reaction by TLC, the reaction mixture was diluted with water, and extracted with ethyl acetate. The organic layer dried over anhydrous Na_2SO_4 was filtered, and concentrated to give the crude material. The crude material was purified by column chromatography using hexane-ethyl acetate as the eluent (93:2) to afford 2-(phenylethynyl)tetrahydrofuran 19% (8 mg) (**102**).

1.8. List of unsuccessful radical precursors in our reaction transformation

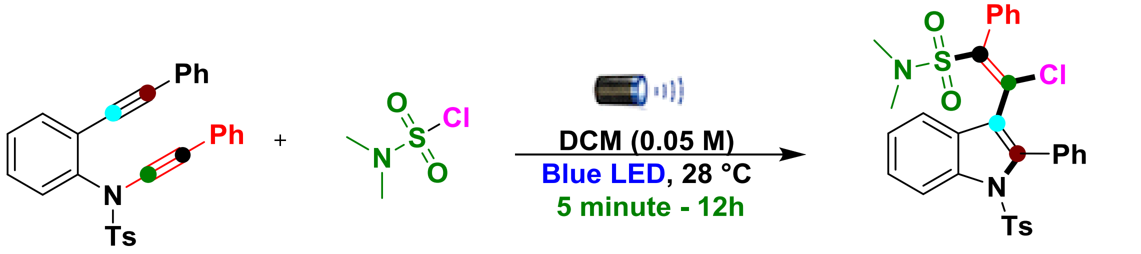

1a, 0.1 mmol 0.11 mmol Yield = 0%

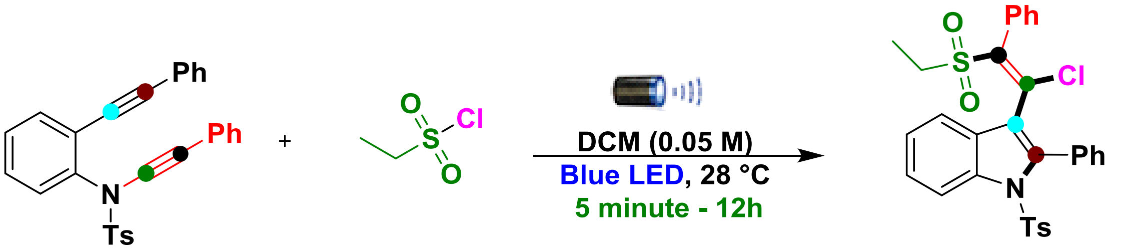

1a, 0.1 mmol 0.11 mmol Yield = 0%

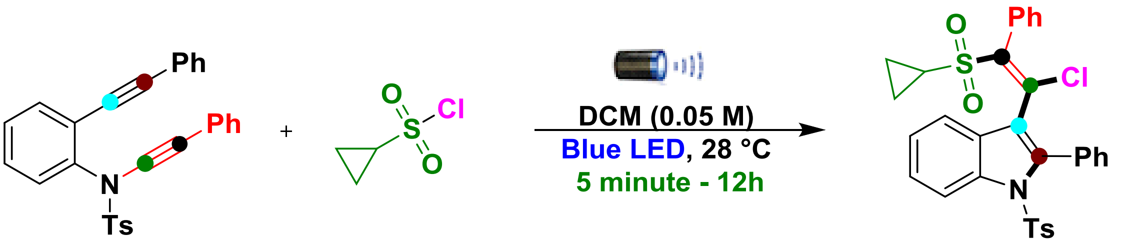

1a, 0.1 mmol 0.11 mmol Yield = 0%

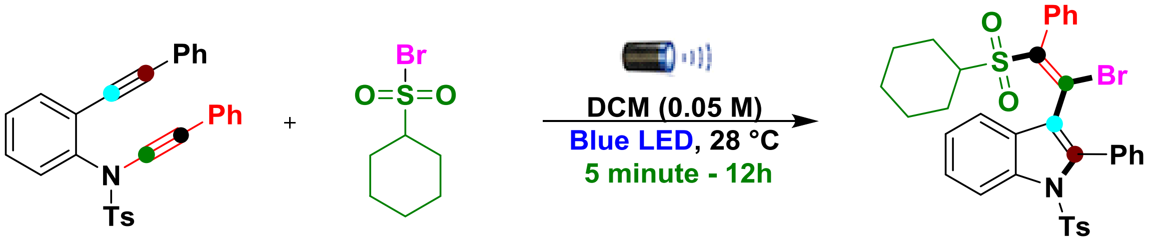

1a, 0.1 mmol 0.11 mmol Yield = 0%

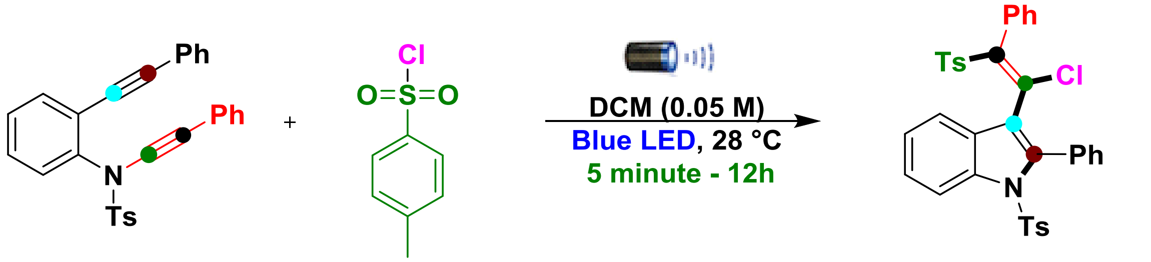

1a, 0.1 mmol 0.11 mmol Yield = 0%

1.9. Blue LED emission spectra and absorption spectra of compounds 2a, 2ca, 2da, 2bd, 2ea and 1a

1.9.1. Instrument used to measure Blue LED emission spectra

Emission spectra were measured using Ocean Optics USB 2000+ Spectrometer. Spectra were normalized to 1.0 at the emission maximum. This emission spectra was provided by Miss Angela Liou, Sales Specialist, DiCon Fiberoptics & DiCon Lighting, aliou@diconfiberoptics.com, Kaohsiung, Taiwan, +886 7 815-8055 Ext 485, DiCon Brands - Kessil | Fiilex | Cielux.

1.9.1a. 40 W Kessil A160WE Tuna Blue LED emission spectra

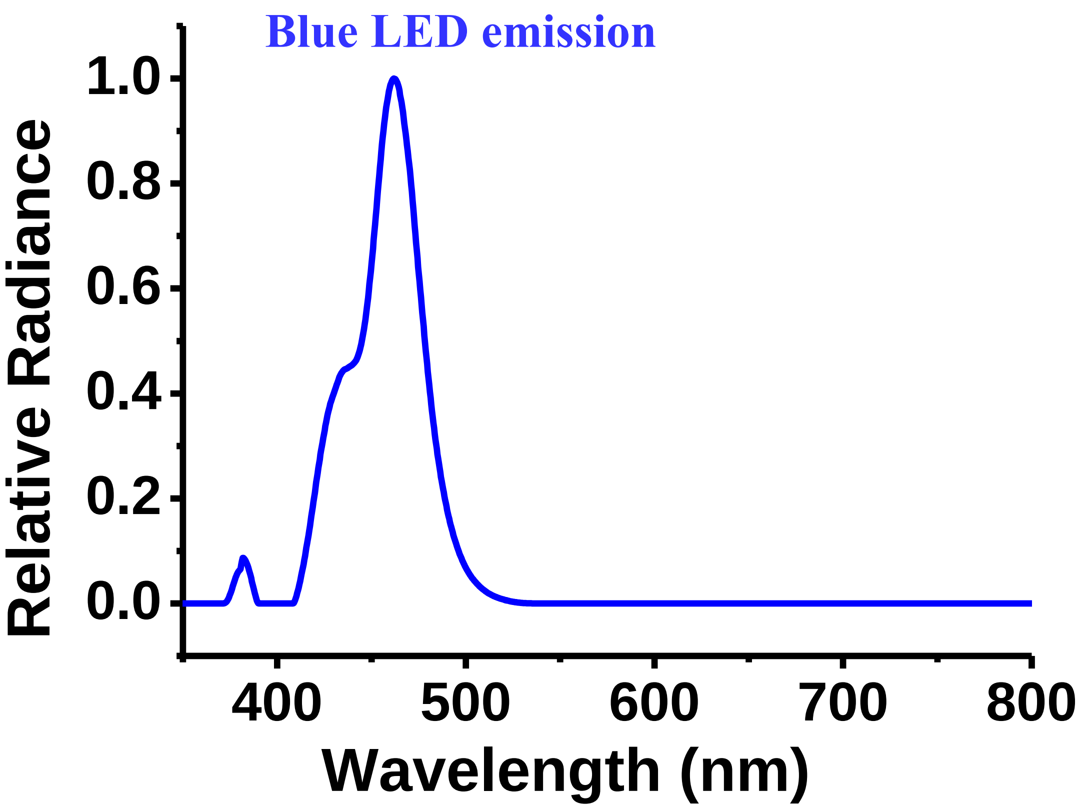

Supplementary Figure 11. Emission spectrum from a 40 W Kessil A160WE Tuna Blue LED shown as blue color line with emission maximum at λ max = 462 nm flanked by a second peak at λ = 382 nm.

1.9.1b. 40 W Kessil PR160L-456 nm Blue LED emission spectra

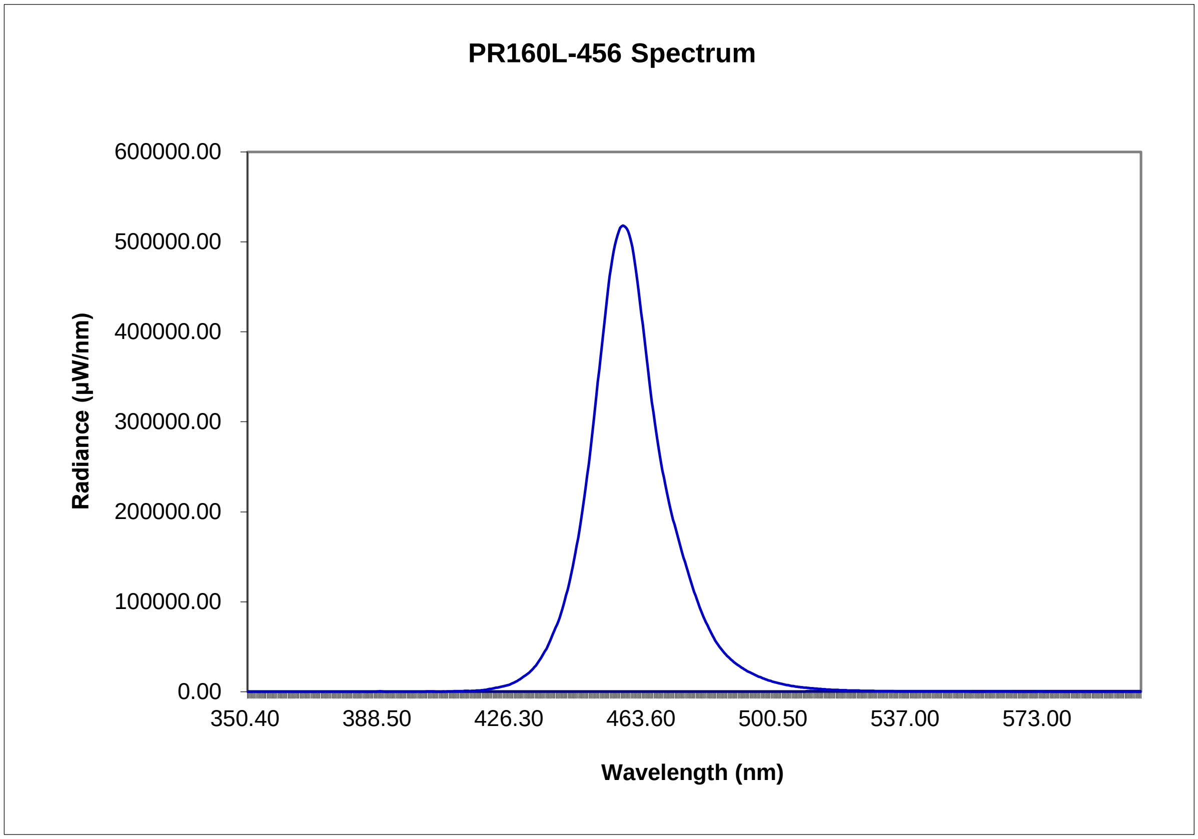

Supplementary Figure 12. Emission spectrum from a 40 W Kessil PR160L-456 nm Blue LED shown as blue color line with emission maximum at λ max = 456 nm.

1.9.2. Absorption spectra

The UV-vis absorption spectra were collected on Shimadzu UV-1900 UV-Vis Spectrophotometer using 1 cm width and 4.4cm height quartz cuvettes by filling 4 mL compound solution. We used HPLC grade solvent on open air for the compound solution preparation.

1.9.2.1a. Absorption spectra of 4-methylbenzenesulfonyl iodide (2a) in DCM solvent

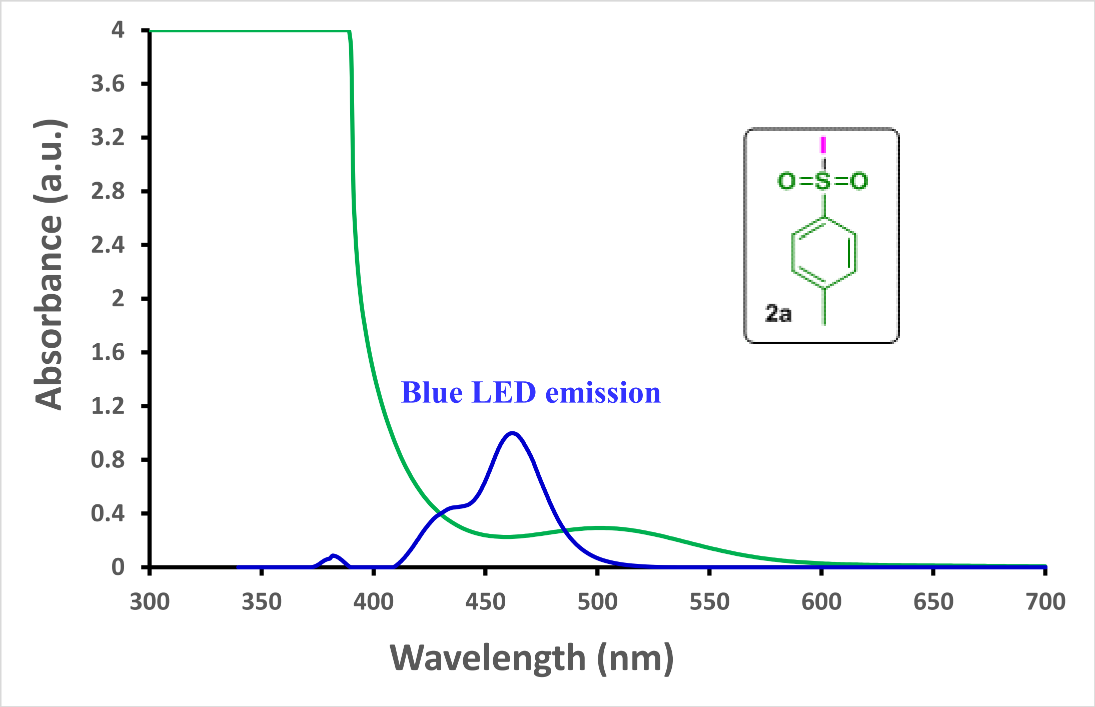

Supplementary Figure 13. Absorption spectra of 4-methylbenzenesulfonyl iodide (**2a**) (10^{-2} M) in DCM, and blue LED emission.

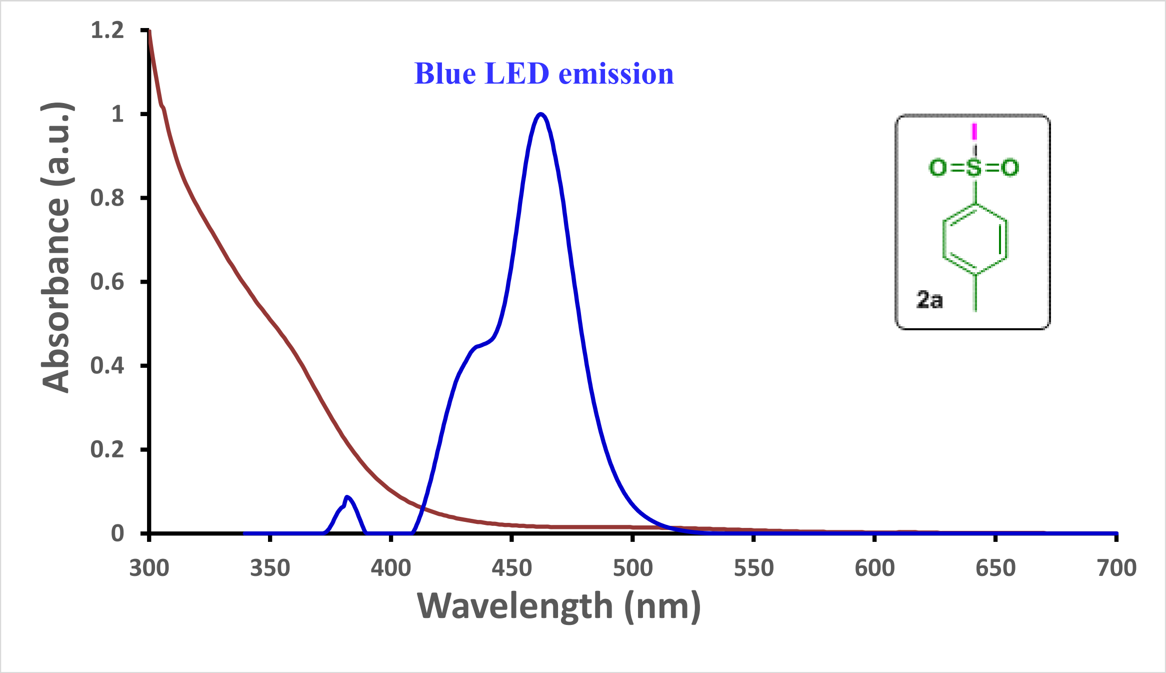

Supplementary Figure 14. Absorption spectra of 4-methylbenzenesulfonyl iodide (**2a**) (10^{-3} M) in DCM, and blue LED emission.

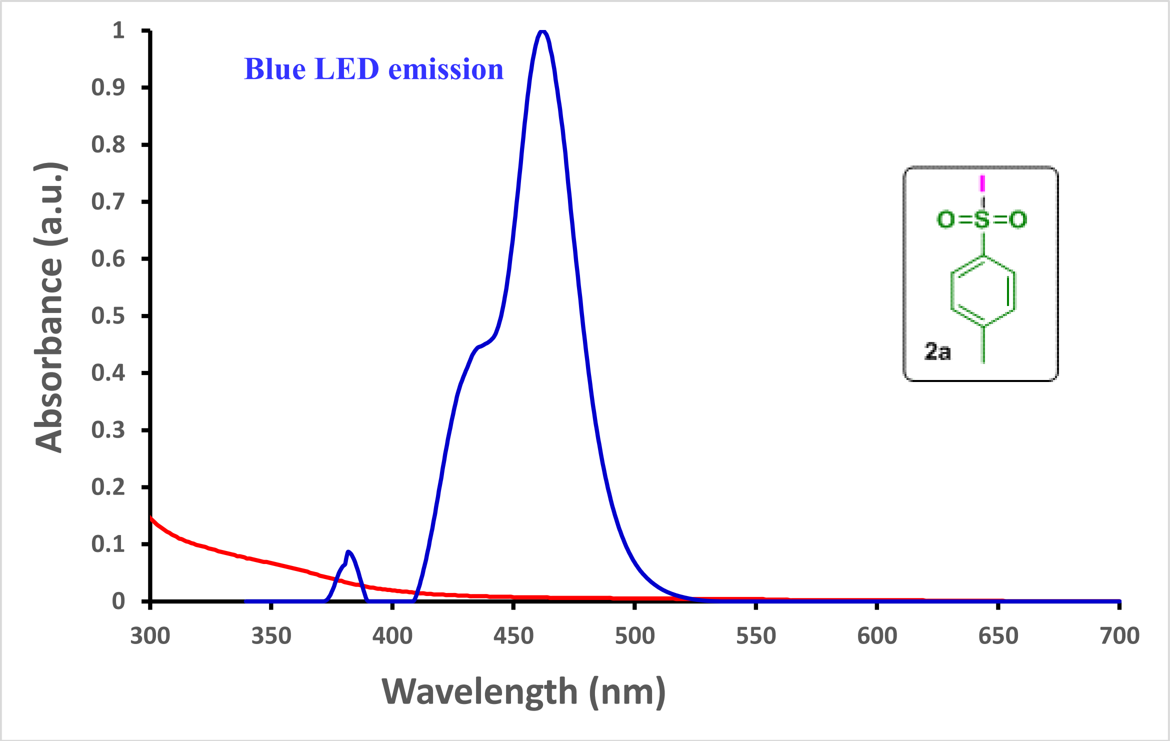

Supplementary Figure 15. Absorption spectra of 4-methylbenzenesulfonyl iodide (**2a**) (10^{-4} M) in DCM, and blue LED emission.

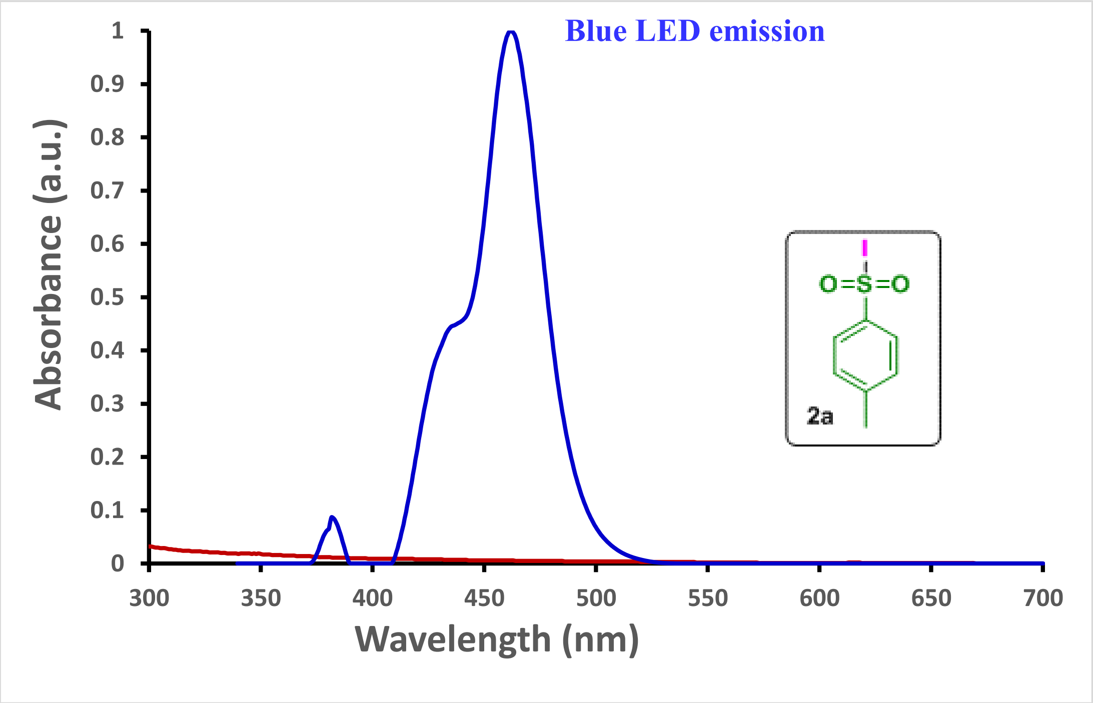

Supplementary Figure 16. Absorption spectra of 4-methylbenzenesulfonyl iodide (**2a**) (10^{-5} M) in DCM, and blue LED emission.

1.9.2.1b. Absorption spectra of 4-methylbenzenesulfonyl iodide (2a) in MeCN solvent

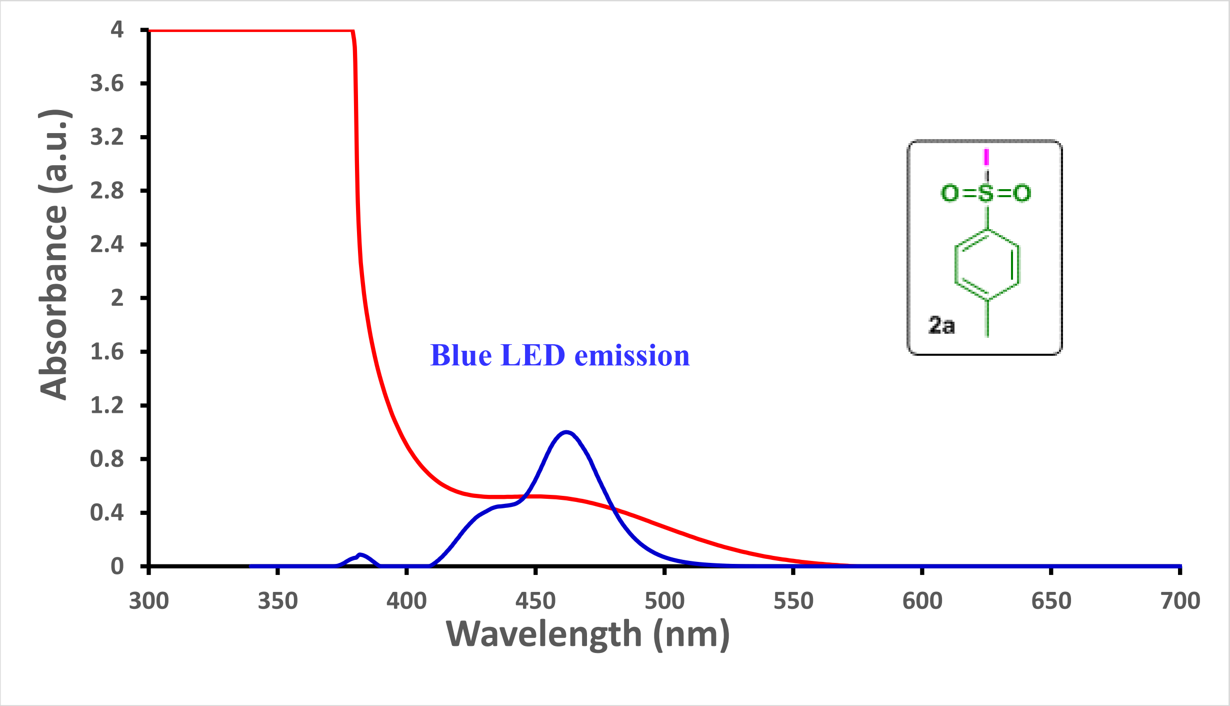

Supplementary Figure 17. Absorption spectra of 4-methylbenzenesulfonyl iodide (**2a**) (10^{-2} M) in MeCN, and blue LED emission.

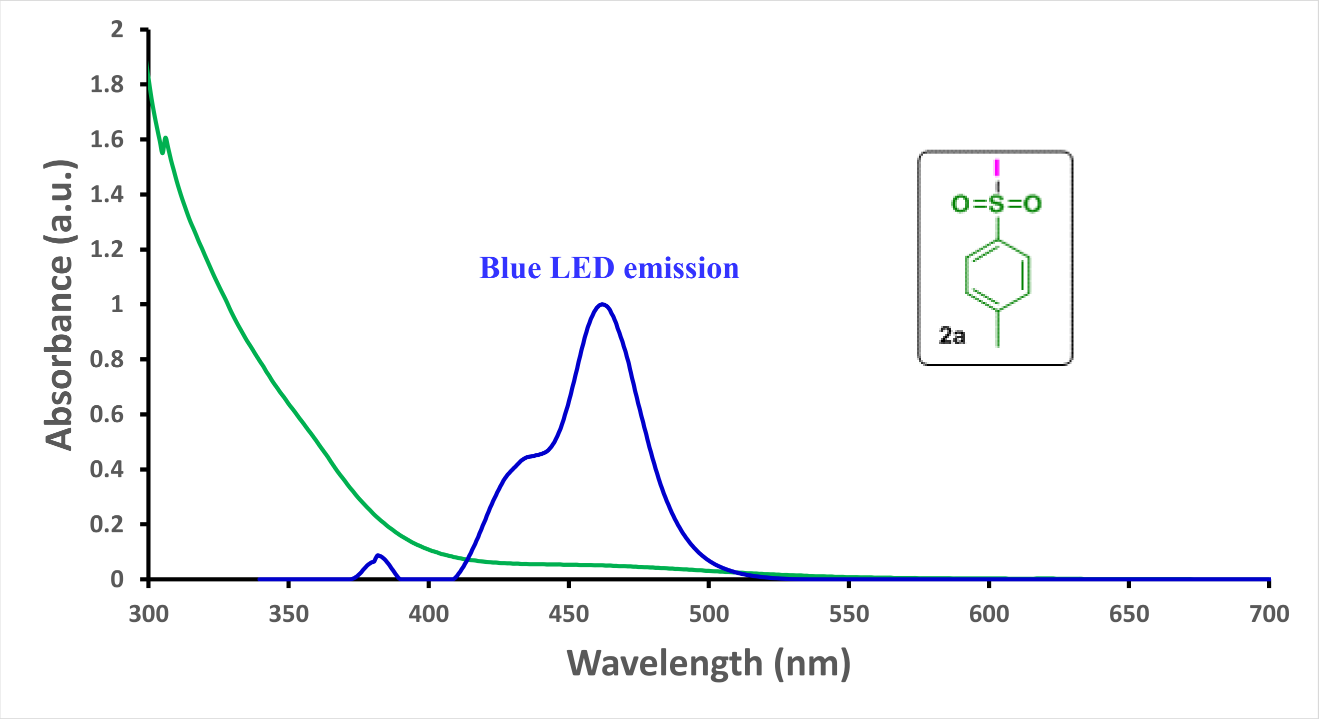

Supplementary Figure 18. Absorption spectra of 4-methylbenzenesulfonyl iodide (**2a**) (10^{-3} M) in MeCN, and blue LED emission.

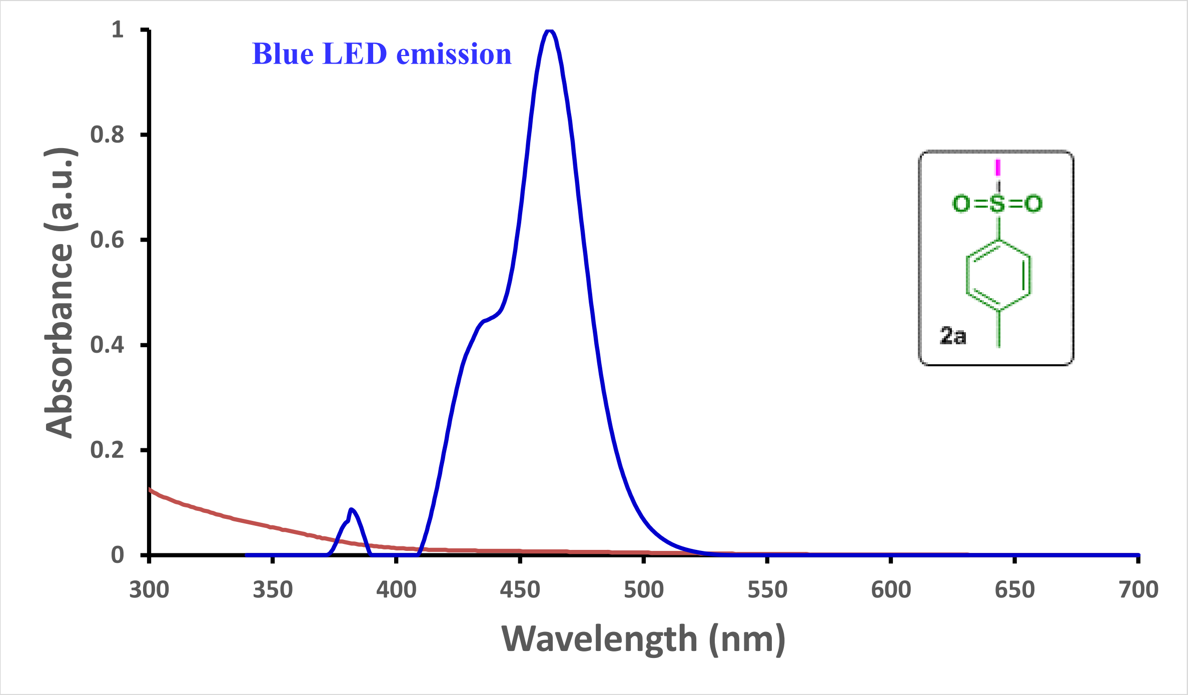

Supplementary Figure 19. Absorption spectra of 4-methylbenzenesulfonyl iodide (**2a**) (10^{-4} M) in MeCN, and blue LED emission.

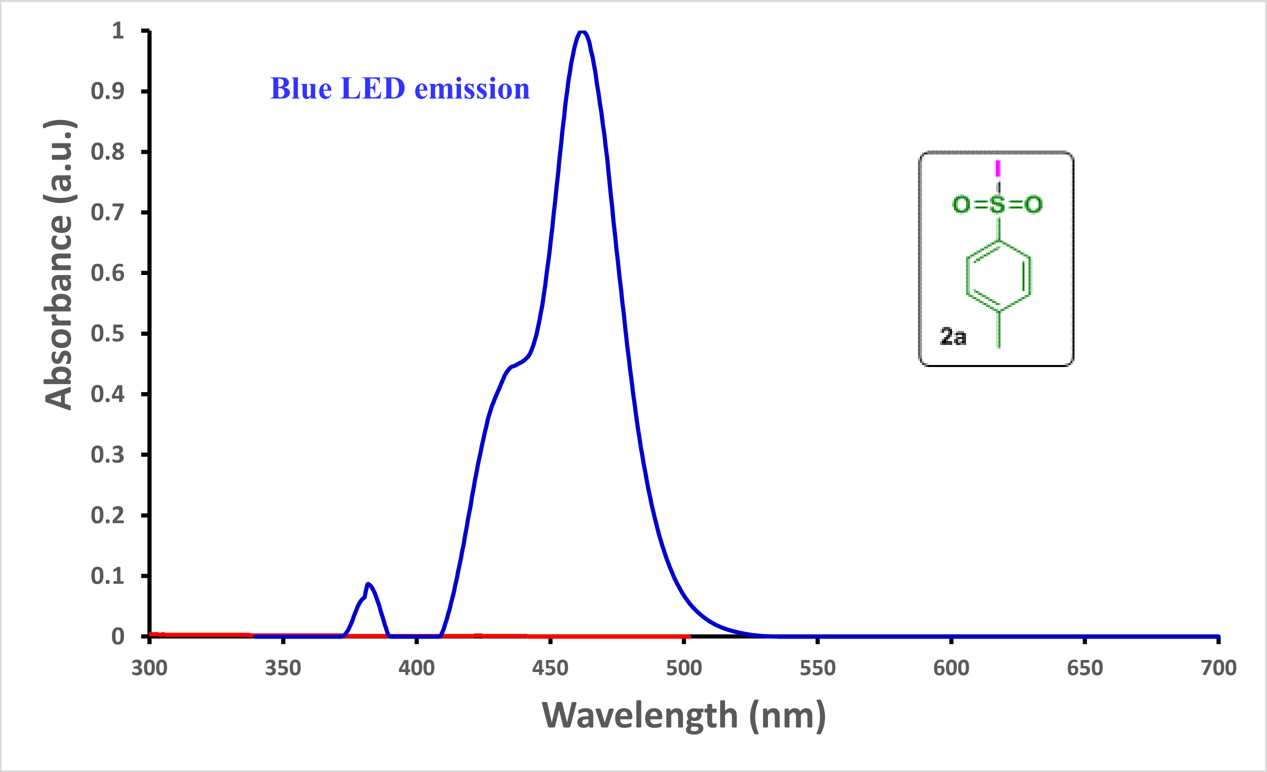

Supplementary Figure 20. Absorption spectra of 4-methylbenzenesulfonyl iodide (**2a**) (10^{-5} M) in MeCN, and blue LED emission.

1.9.2.1c. Absorption spectra of 4-methylbenzenesulfonyl iodide (2a) in THF solvent

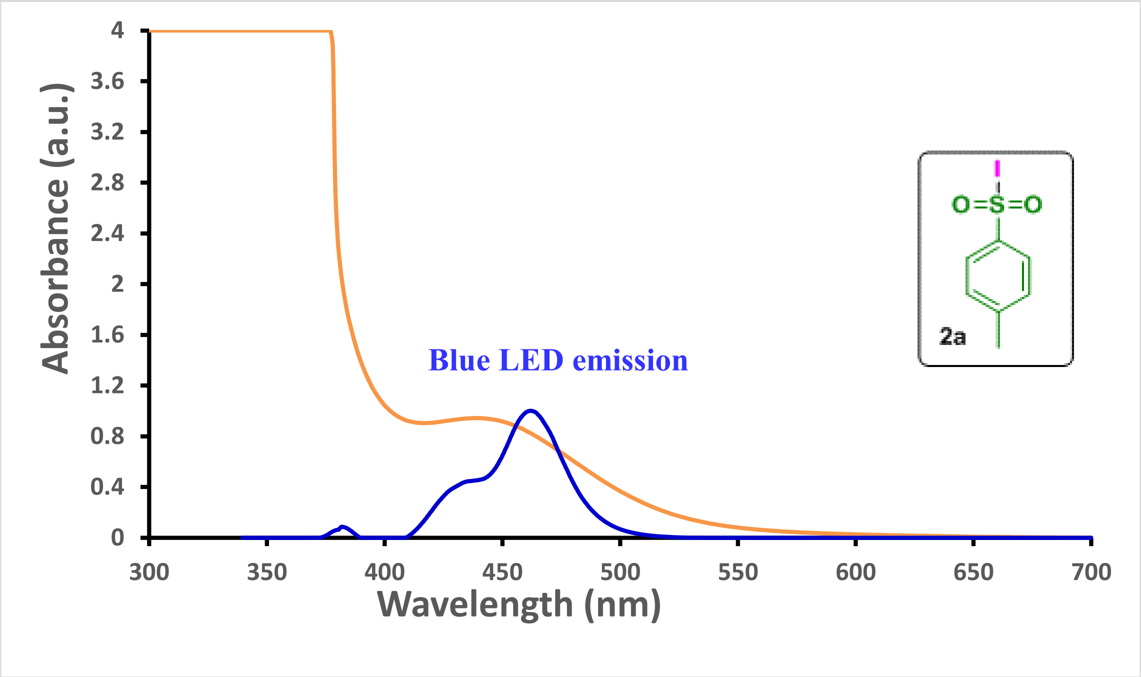

Supplementary Figure 21. Absorption spectra of 4-methylbenzenesulfonyl iodide (**2a**) (10^{-2} M) in THF, and blue LED emission.

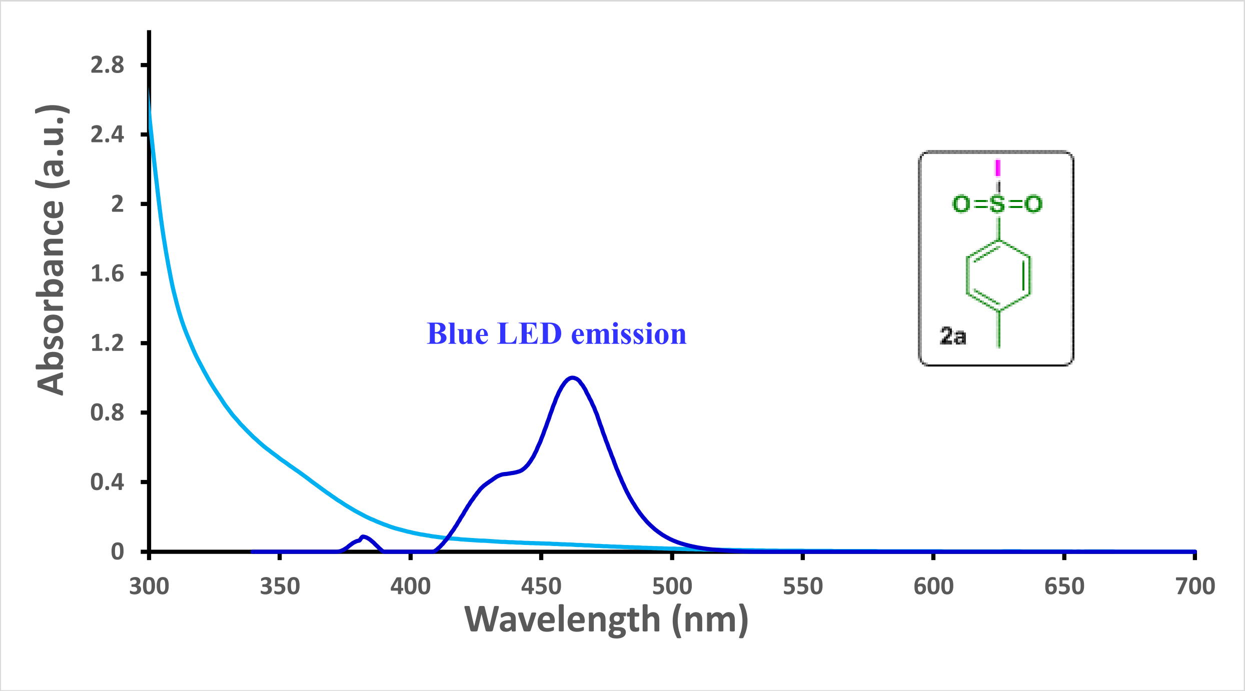

Supplementary Figure 22. Absorption spectra of 4-methylbenzenesulfonyl iodide (**2a**) (10^{-3} M) in THF, and blue LED emission.

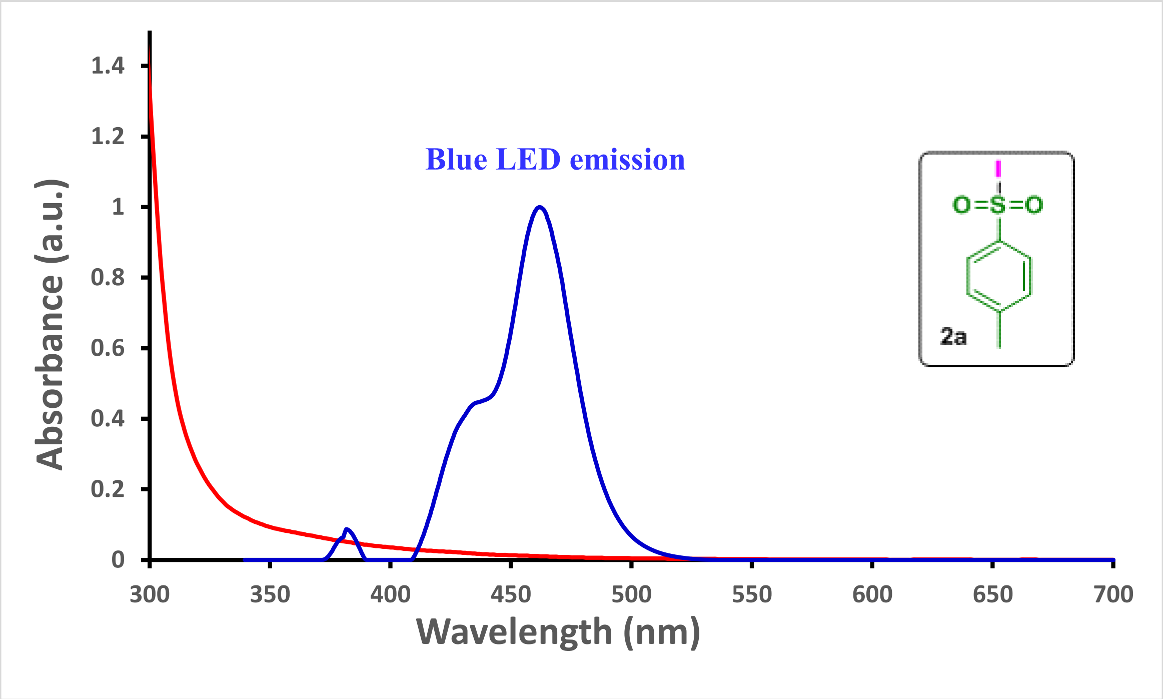

Supplementary Figure 23. Absorption spectra of 4-methylbenzenesulfonyl iodide (**2a**) (10^{-4} M) in THF, and blue LED emission.

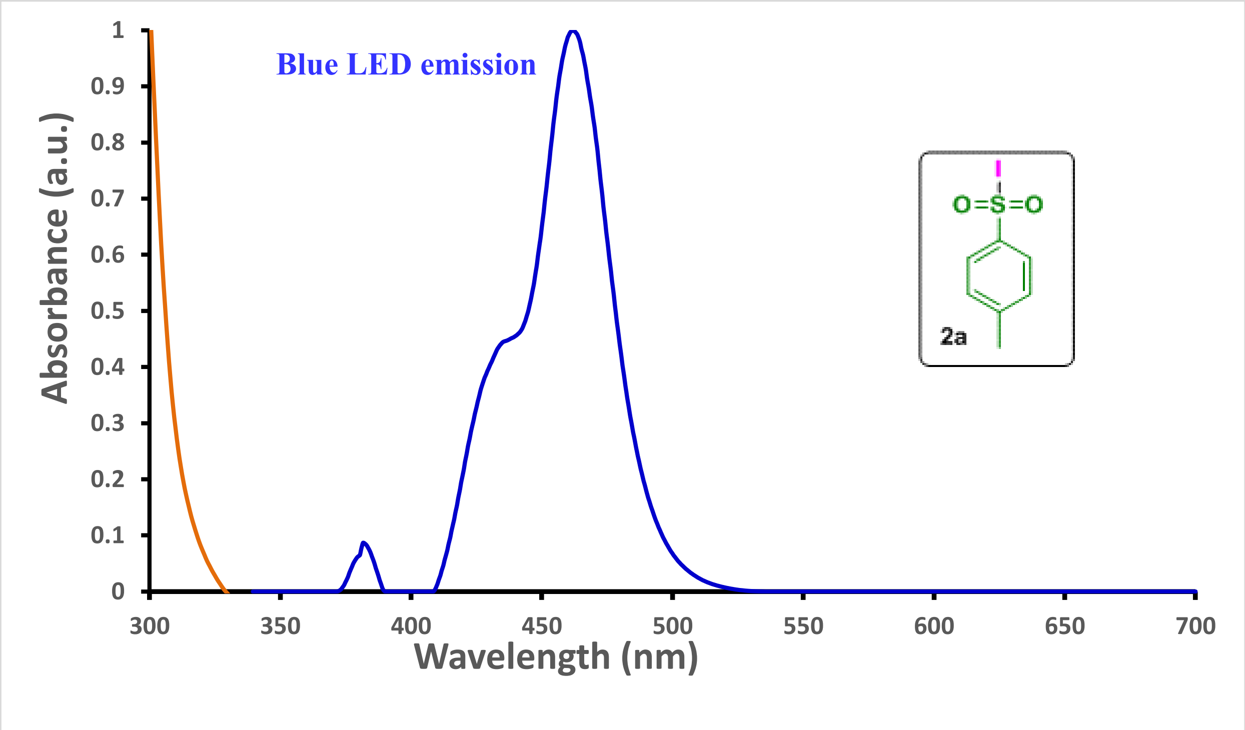

Supplementary Figure 24. Absorption spectra of 4-methylbenzenesulfonyl iodide (**2a**) (10^{-5} M) in THF, and blue LED emission.

1.9.2.2. Absorption spectra of 4-methylbenzenesulfonyl bromide (2ca) in DCM

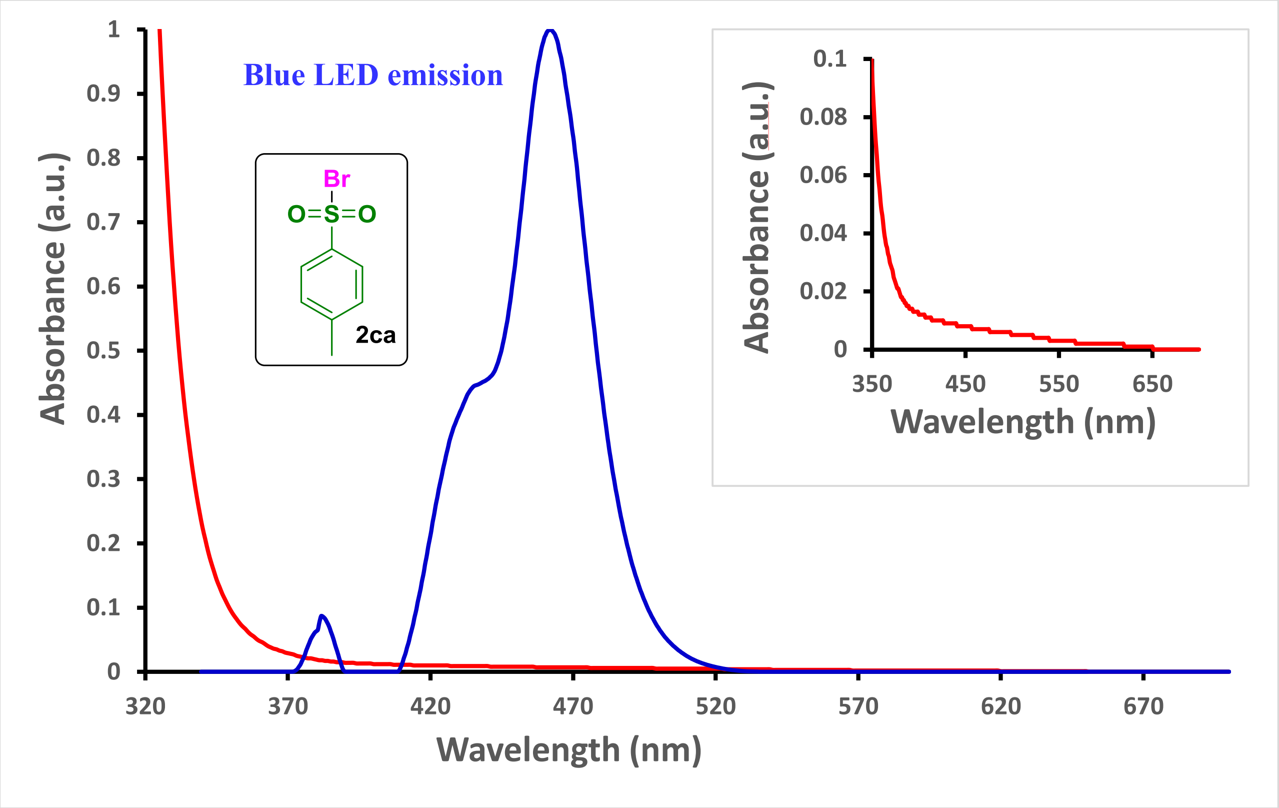

Supplementary Figure 25. Absorption spectra of 4-methylbenzenesulfonyl bromide (**2ca**) (10^{-2} M) in DCM, blue LED emission and zoom appearance.

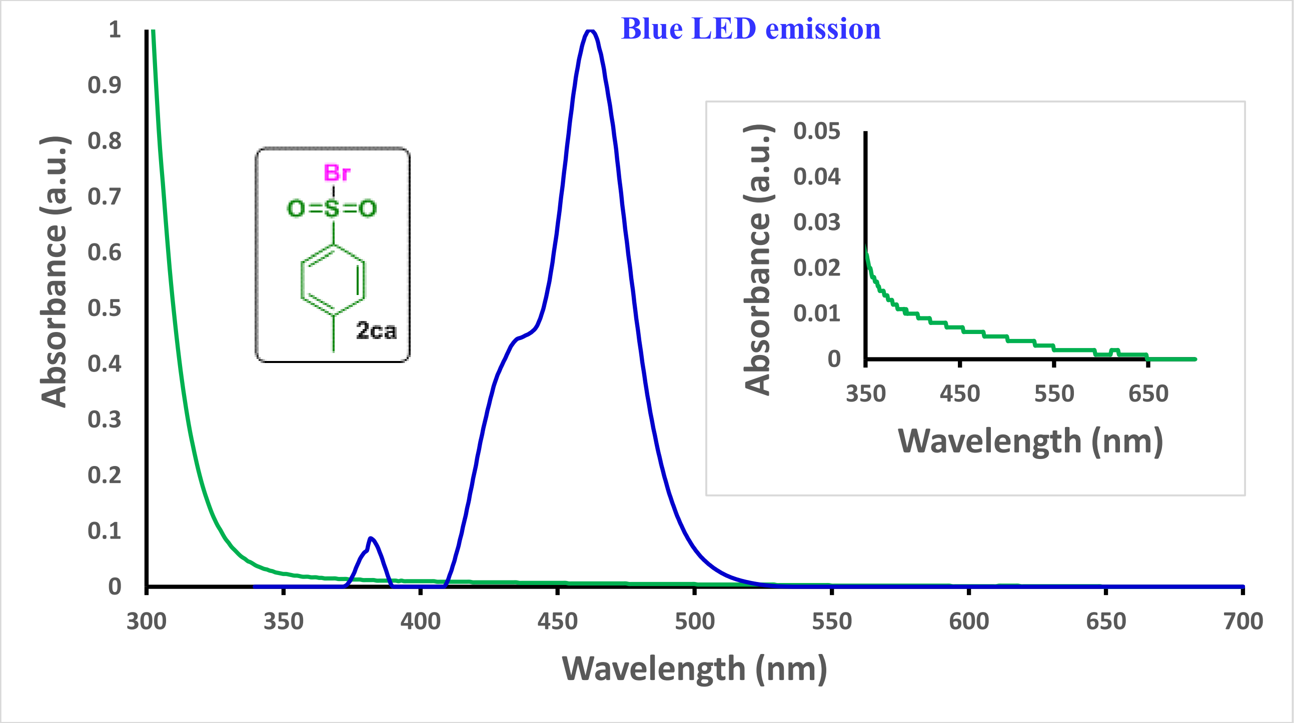

Supplementary Figure 26. Absorption spectra of 4-methylbenzenesulfonyl bromide (**2ca**) (10^{-3} M) in DCM, blue LED emission and zoom appearance.

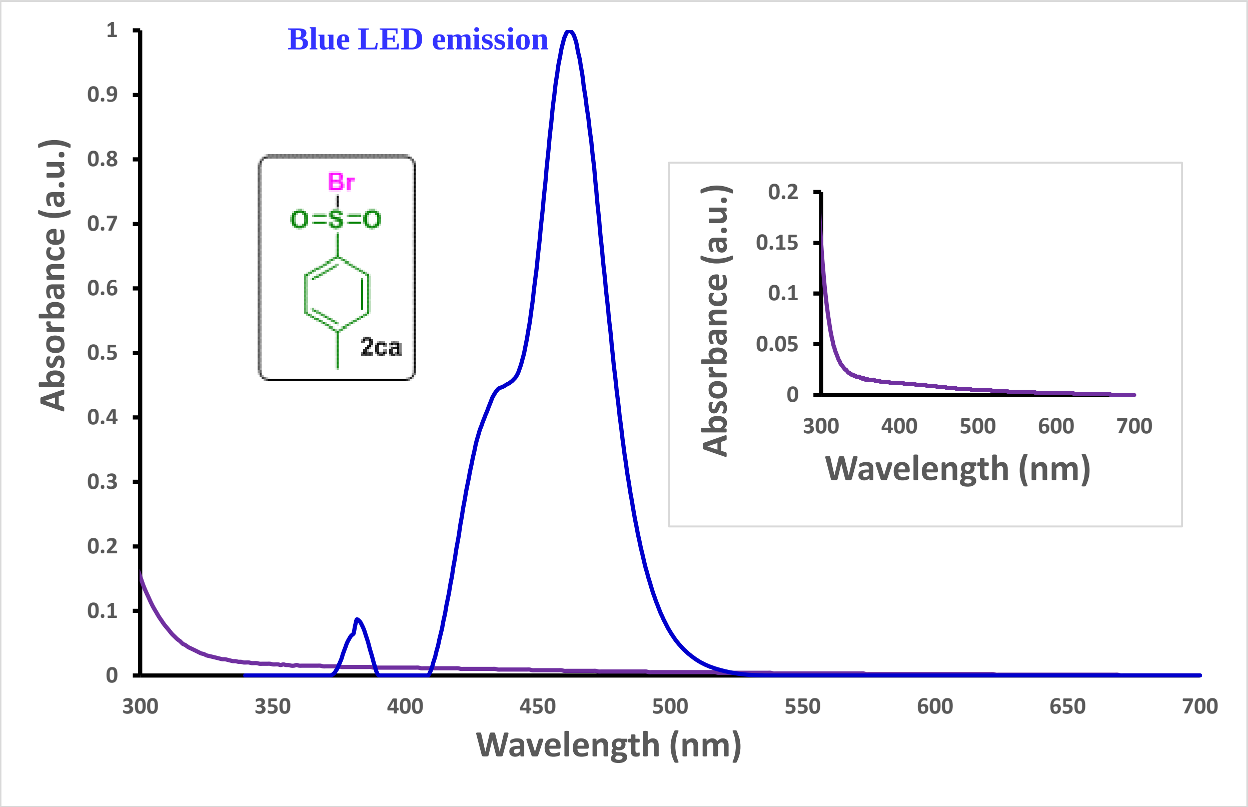

Supplementary Figure 27. Absorption spectra of 4-methylbenzenesulfonyl bromide (**2ca**) (10^{-4} M) in DCM, blue LED emission and zoom appearance.

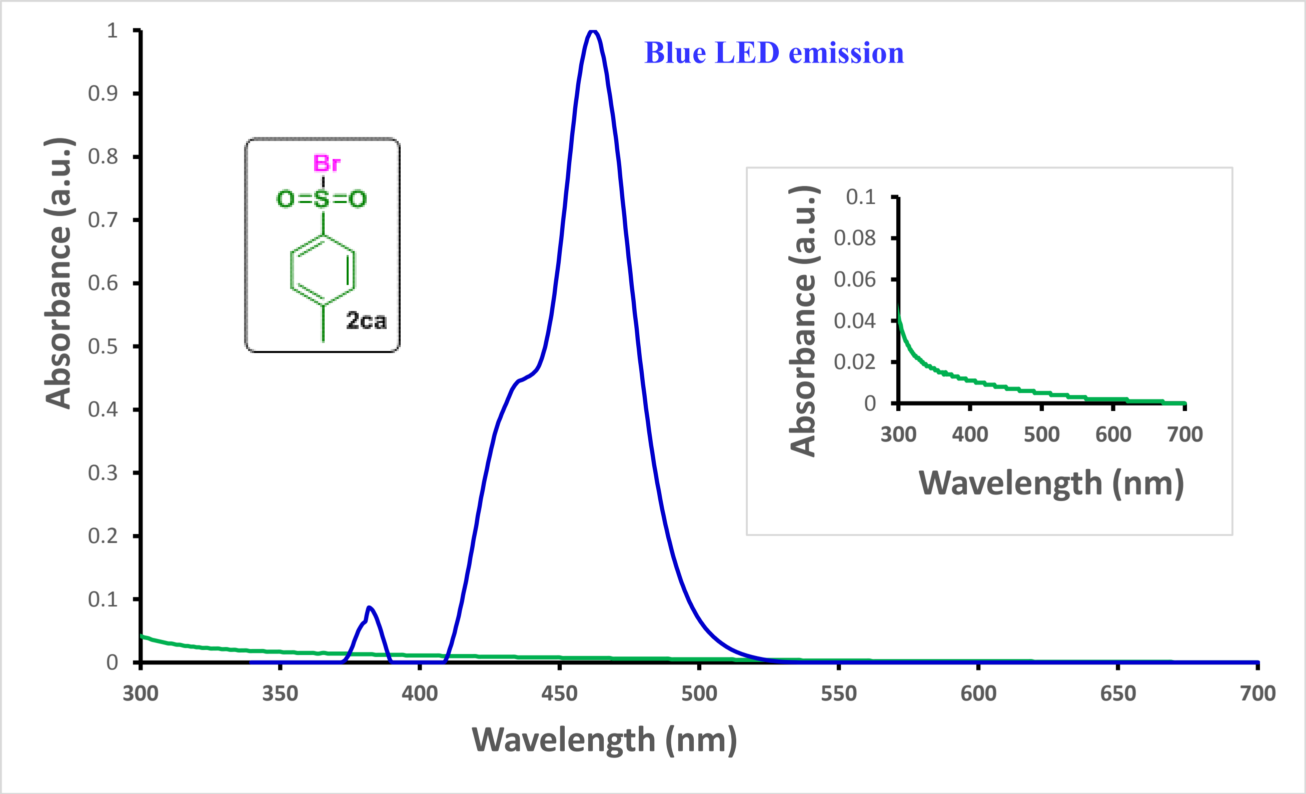

Supplementary Figure 28. Absorption spectra of 4-methylbenzenesulfonyl bromide (**2ca**) (10^{-5} M) in DCM, blue LED emission and zoom appearance.

1.9.2.3. Absorption spectra of Se-phenyl 4-methylbenzenesulfonoselenoate (2da) in DCM

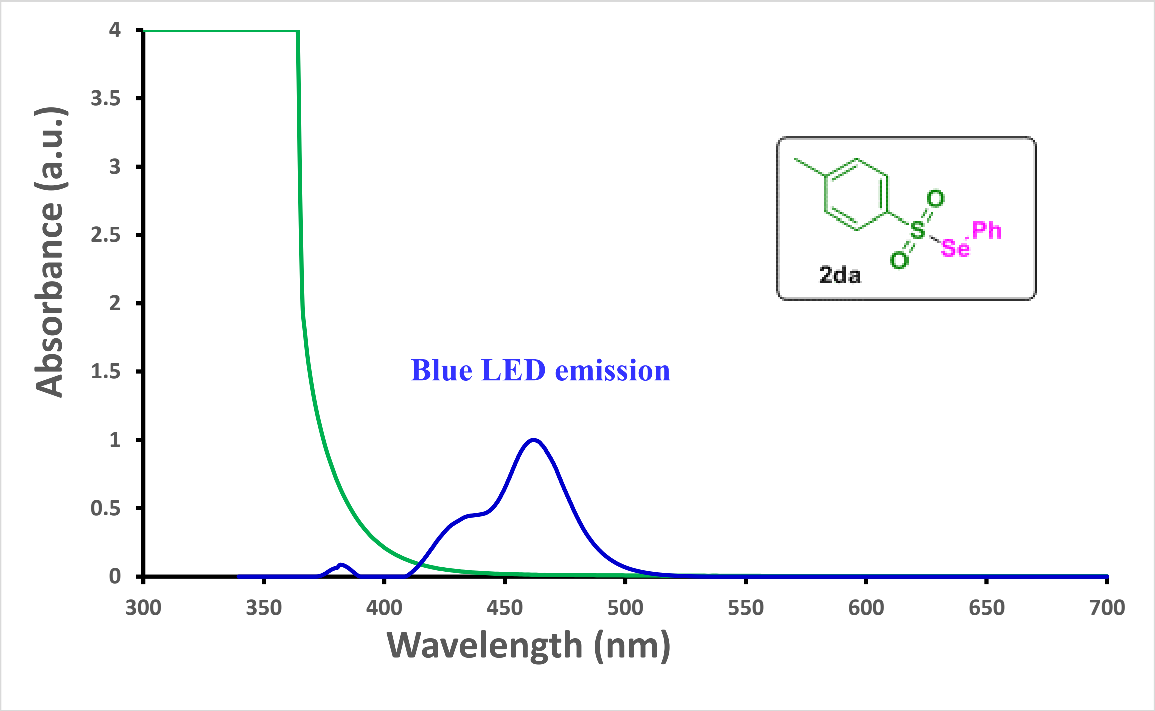

Supplementary Figure 29. Absorption spectra of Se-phenyl 4-methylbenzenesulfonoselenoate (**2da**) (10^{-2} M) in DCM, and blue LED emission.

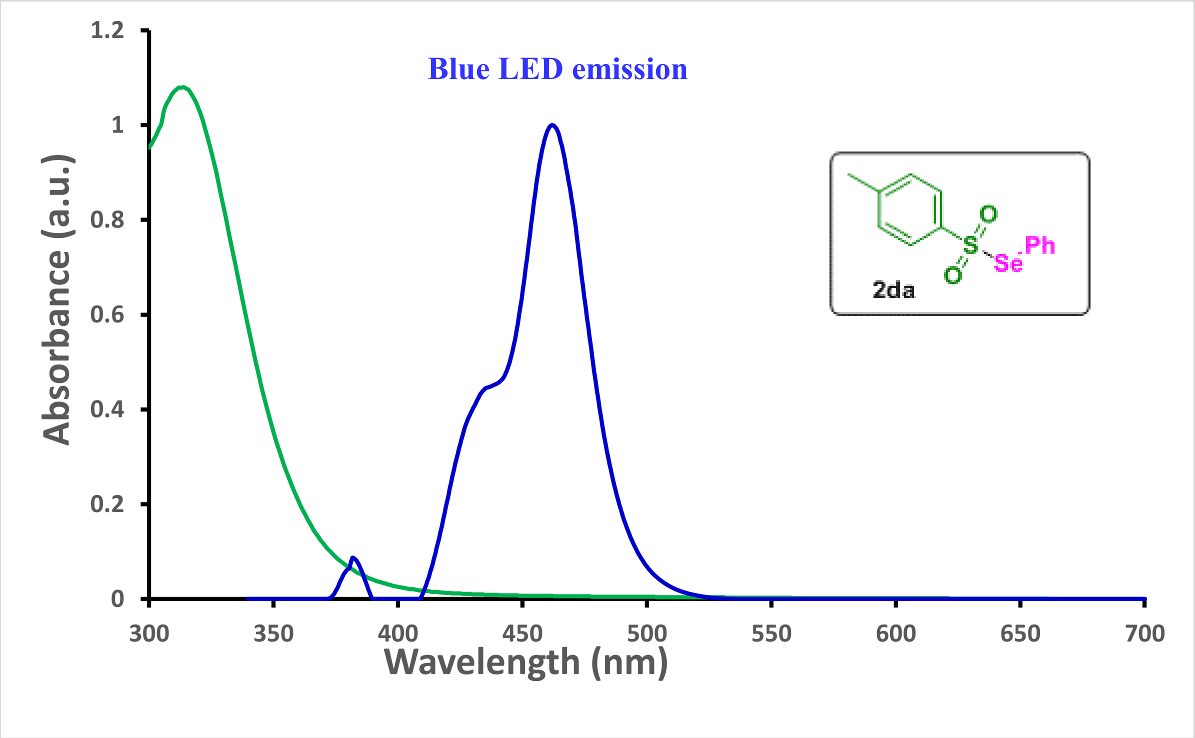

Supplementary Figure 30. Absorption spectra of Se-phenyl 4-methylbenzenesulfonoselenoate (**2da**) (10^{-3} M) in DCM, and blue LED emission.

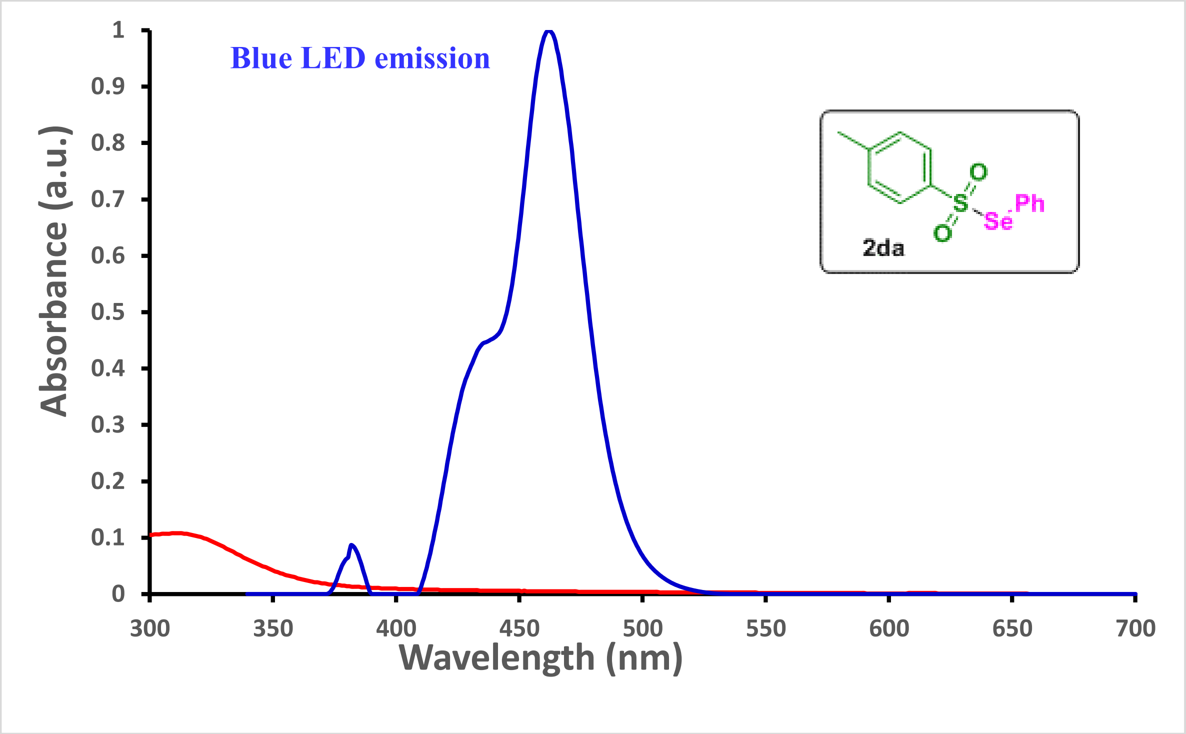

Supplementary Figure 31. Absorption spectra of Se-phenyl 4-methylbenzenesulfonoselenoate (**2da**) (10^{-4} M) in DCM, and blue LED emission.

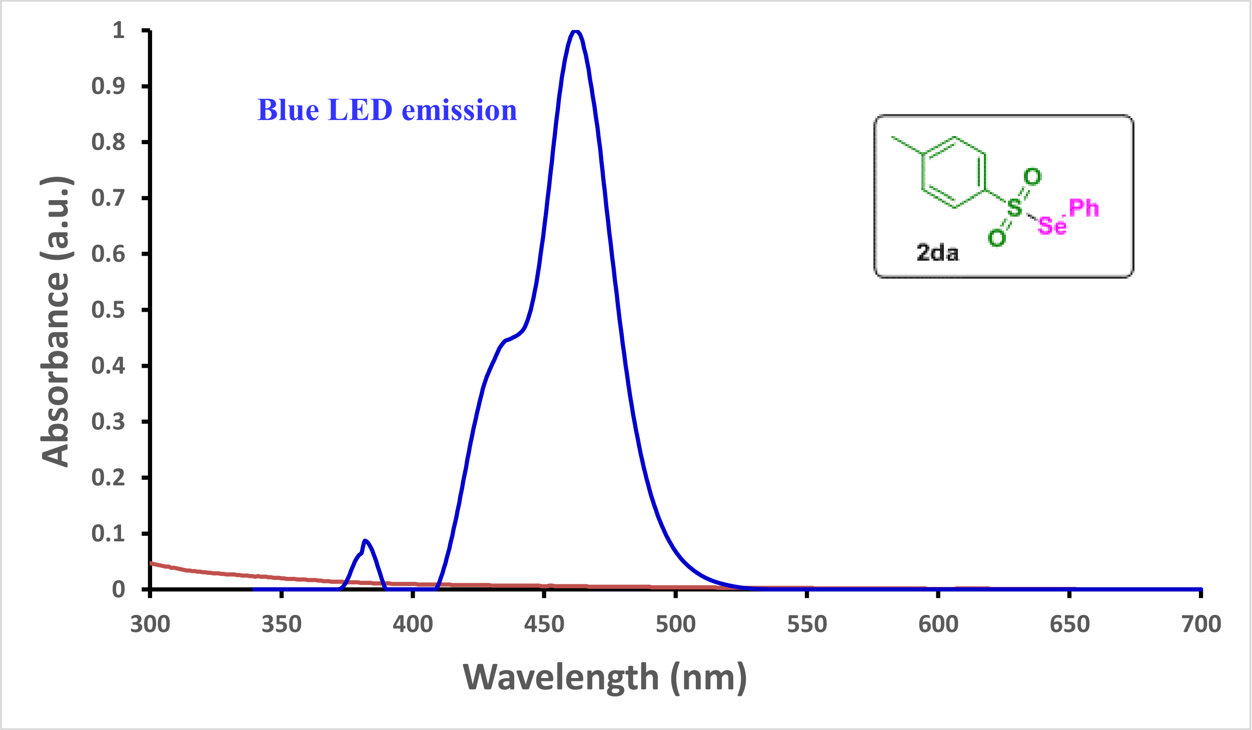

Supplementary Figure 32. Absorption spectra of Se-phenyl 4-methylbenzenesulfonoselenoate (**2da**) (10^{-5} M) in DCM, and blue LED emission.

1.9.2.4. Absorption spectra of 4-methylbenzenesulfonohydrazide (2bd) in DCM

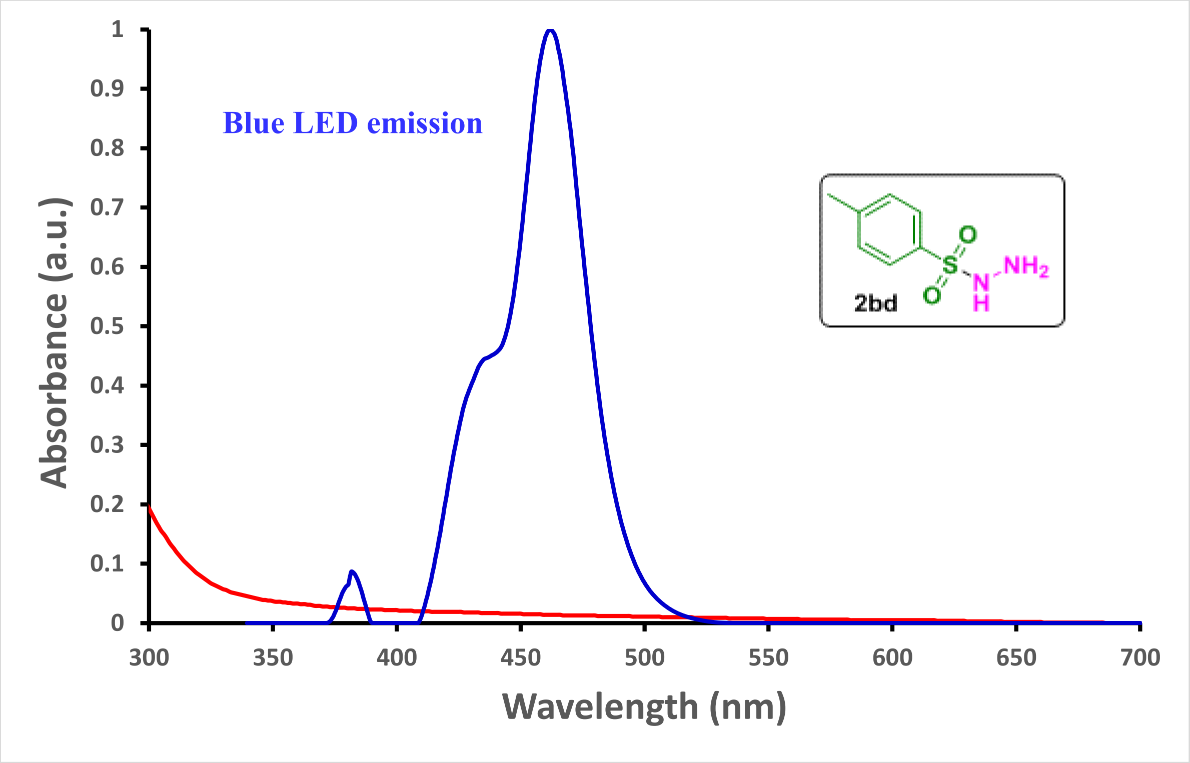

Supplementary Figure 33. Absorption spectra of 4-methylbenzenesulfonohydrazide (**2bd**) (10^{-2} M) in DCM, and blue LED emission.

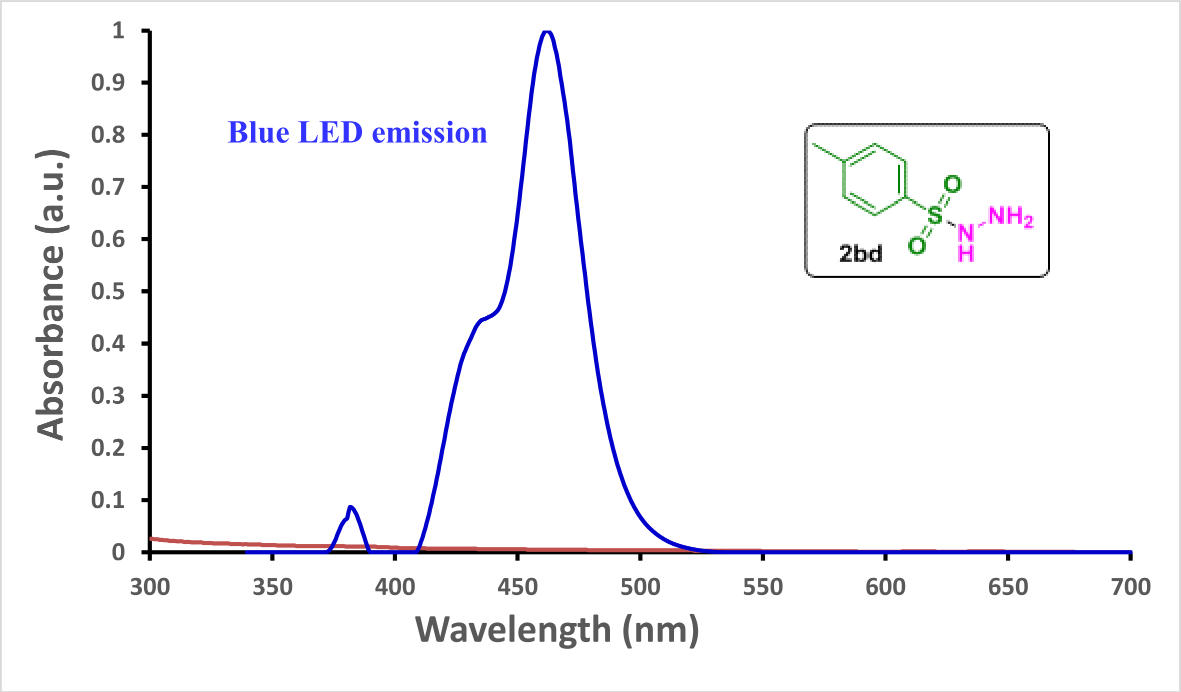

Supplementary Figure 34. Absorption spectra of 4-methylbenzenesulfonohydrazide (**2bd**) (10^{-4} M) in DCM, and blue LED emission.

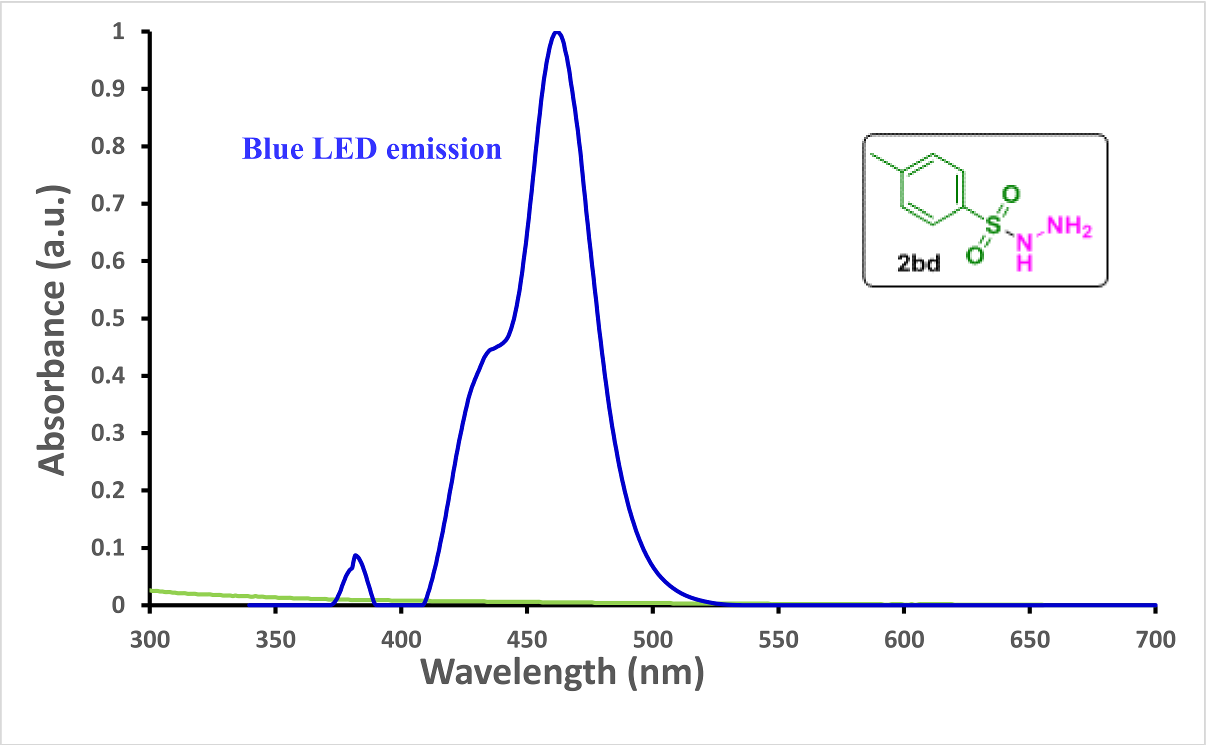

Supplementary Figure 35. Absorption spectra of 4-methylbenzenesulfonohydrazide (**2bd**) (10^{-5} M) in DCM, and blue LED emission.

1.9.2.5. Absorption spectra of benzenethiol (2ea) in MeCN

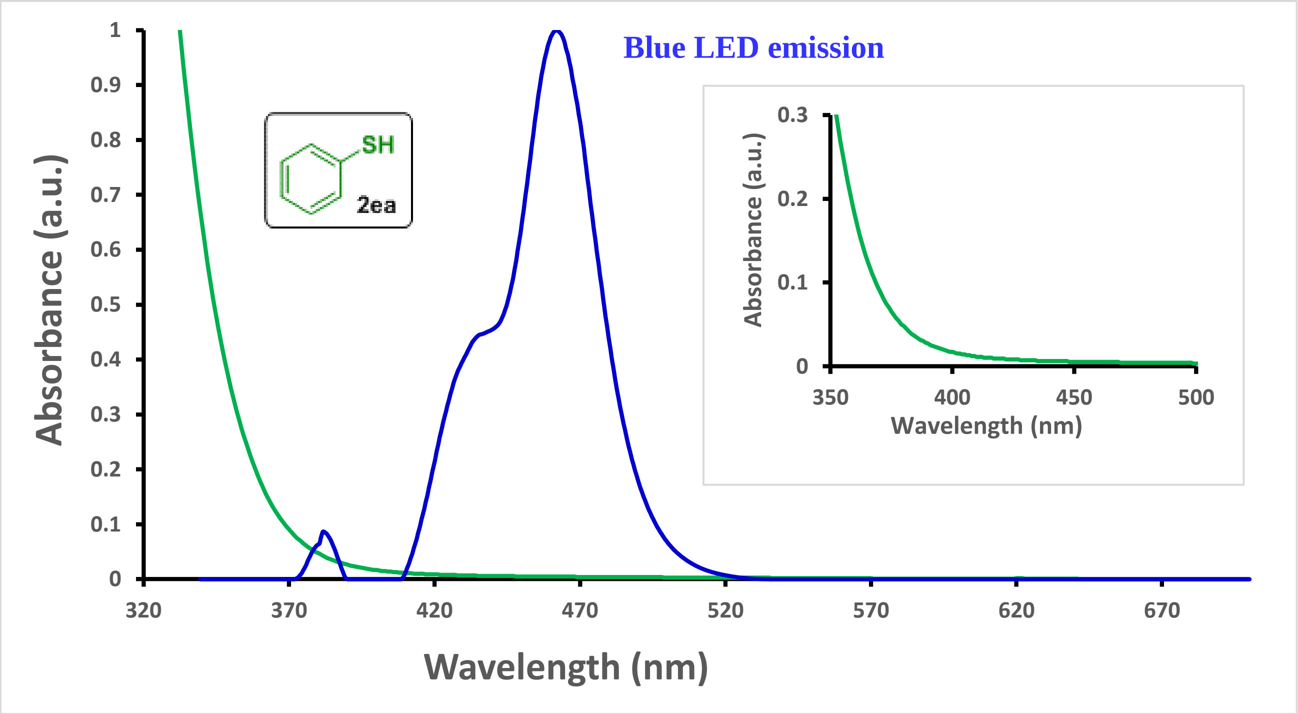

Supplementary Figure 36. Absorption spectra of benzenethiol (**2ea**) (10^{-1} M) in MeCN, blue LED emission and zoom appearance.

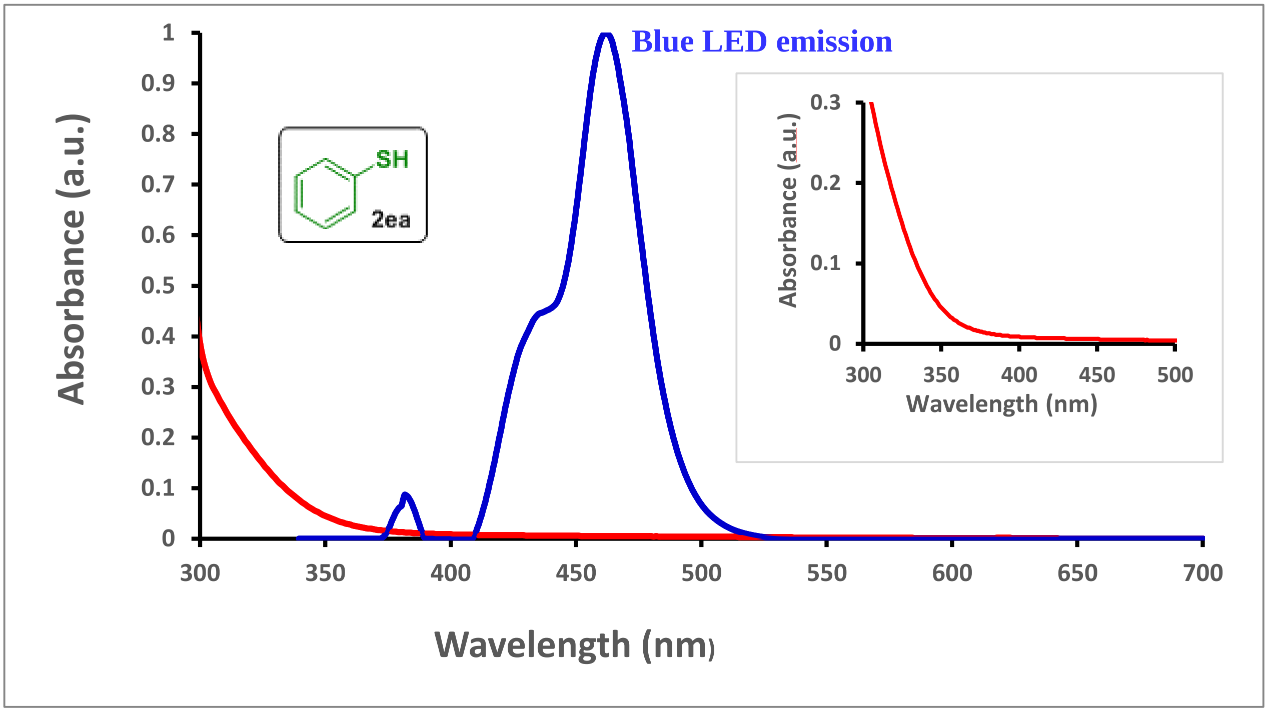

Supplementary Figure 37. Absorption spectra of benzenethiol (**2ea**) (10^{-2} M) in MeCN, blue LED emission and zoom appearance.

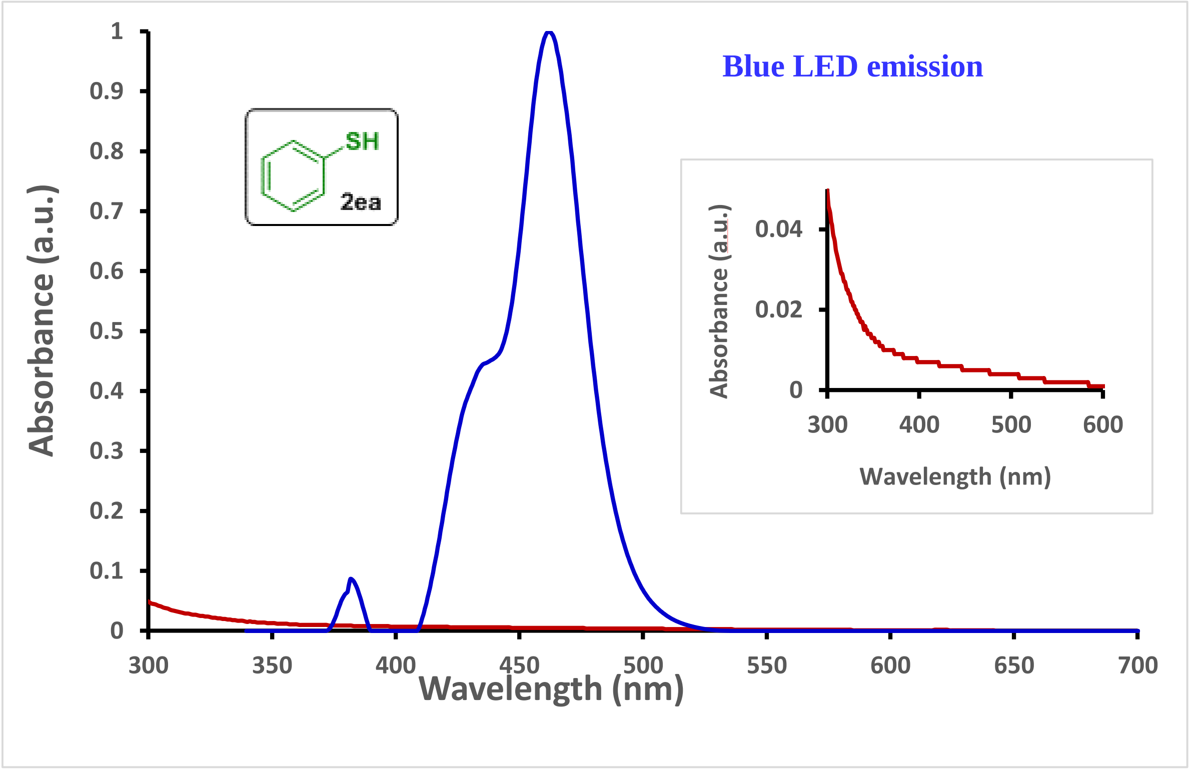

Supplementary Figure 38. Absorption spectra of benzenethiol (**2ea**) (10^{-3} M) in MeCN, blue LED emission and zoom appearance.

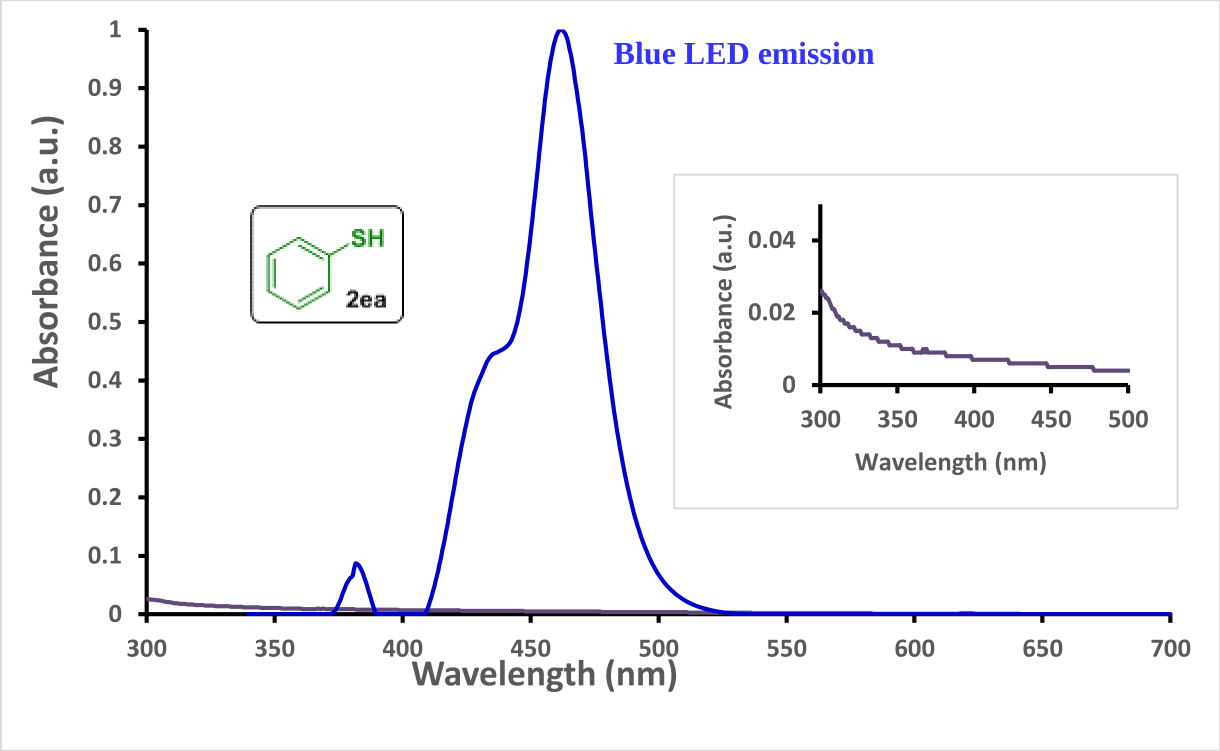

Supplementary Figure 39. Absorption spectra of benzenethiol (**2ea**) (10^{-4} M) in MeCN, blue LED emission and zoom appearance.

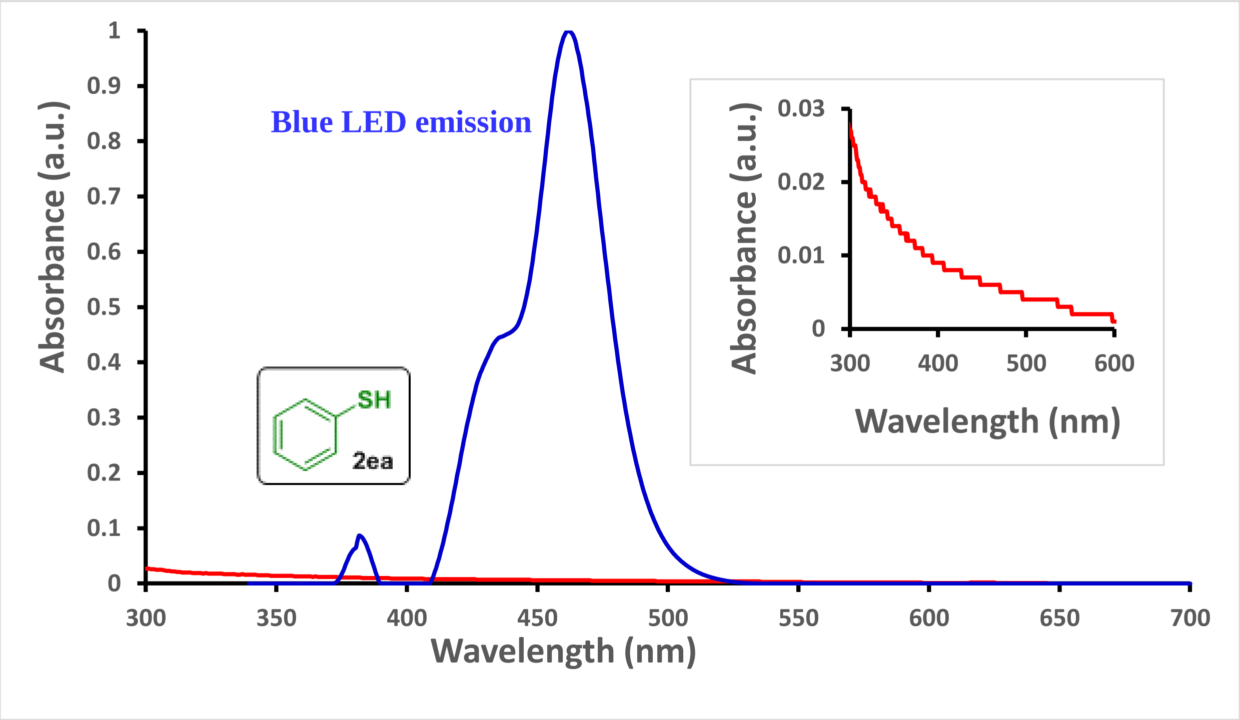

Supplementary Figure 40. Absorption spectra of benzenethiol (**2ea**) (10^{-5} M) in MeCN, blue LED emission and zoom appearance.

1.9.2.6. Absorption spectra of benzenethiol (2ea) in DCM

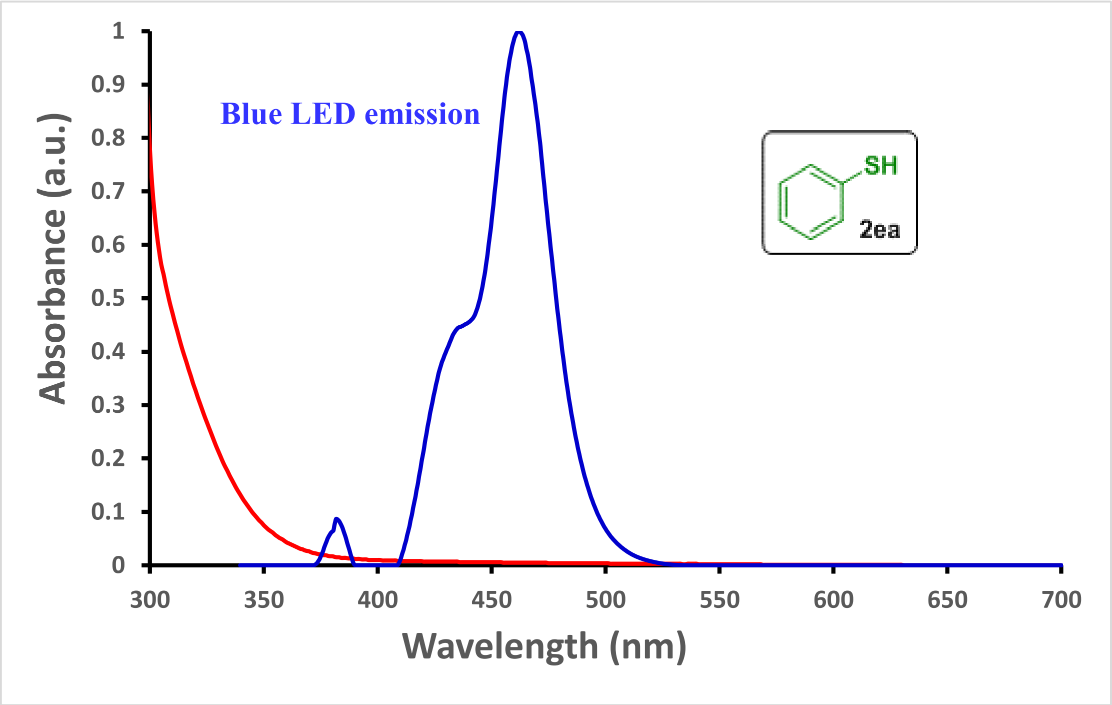

Supplementary Figure 41. Absorption spectra of benzenethiol (**2ea**) (10^{-2} M) in DCM, and blue LED emission.

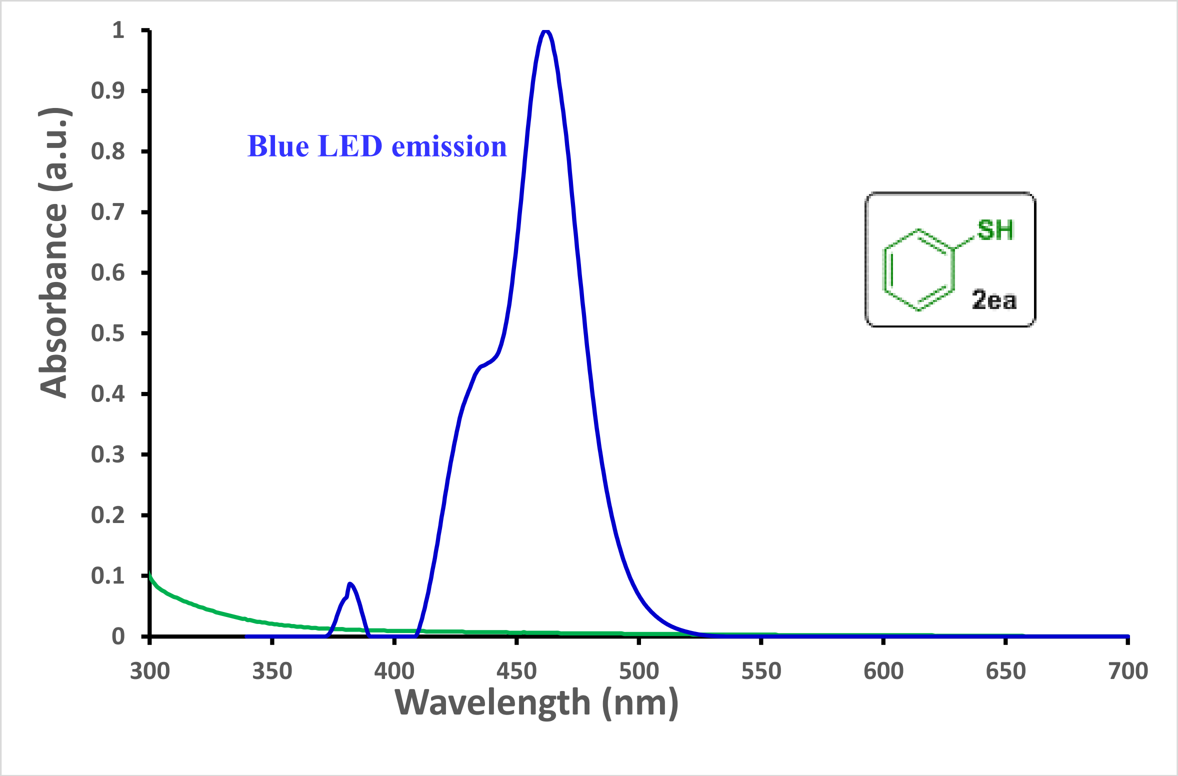

Supplementary Figure 42. Absorption spectra of benzenethiol (**2ea**) (10^{-3} M) in DCM, and blue LED emission.

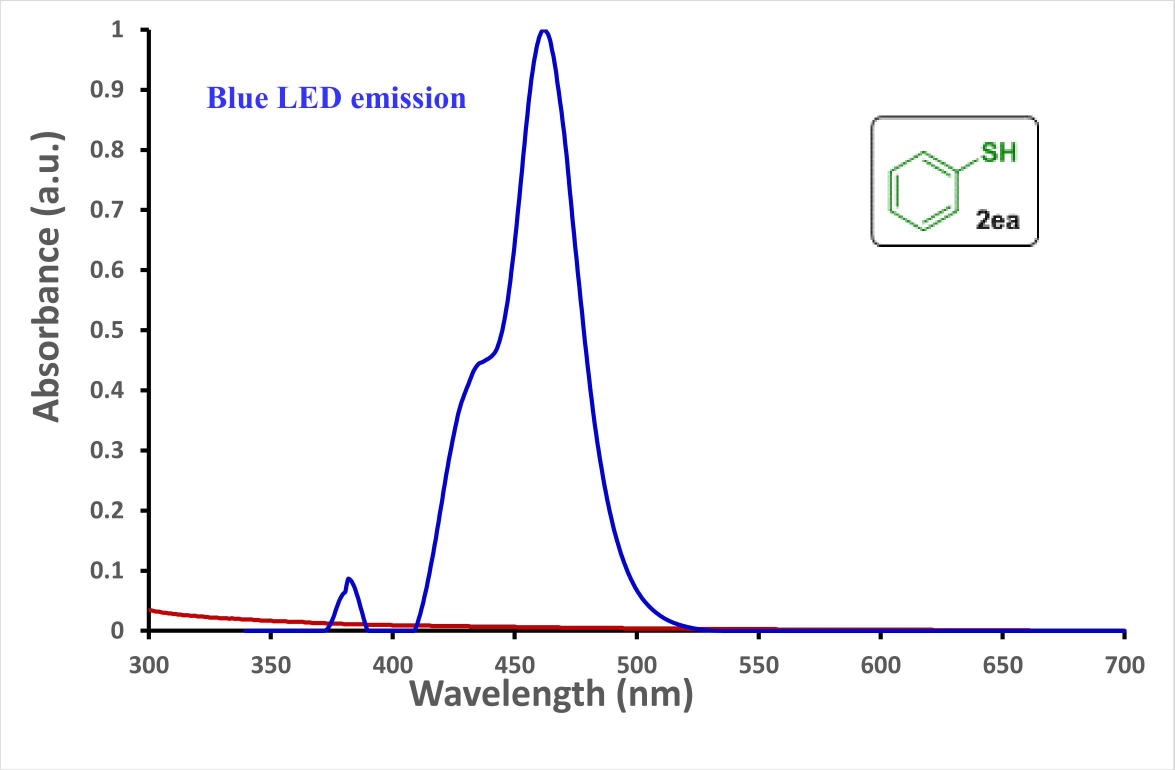

Supplementary Figure 43. Absorption spectra of benzenethiol (**2ea**) (10^{-4} M) in DCM, and blue LED emission.

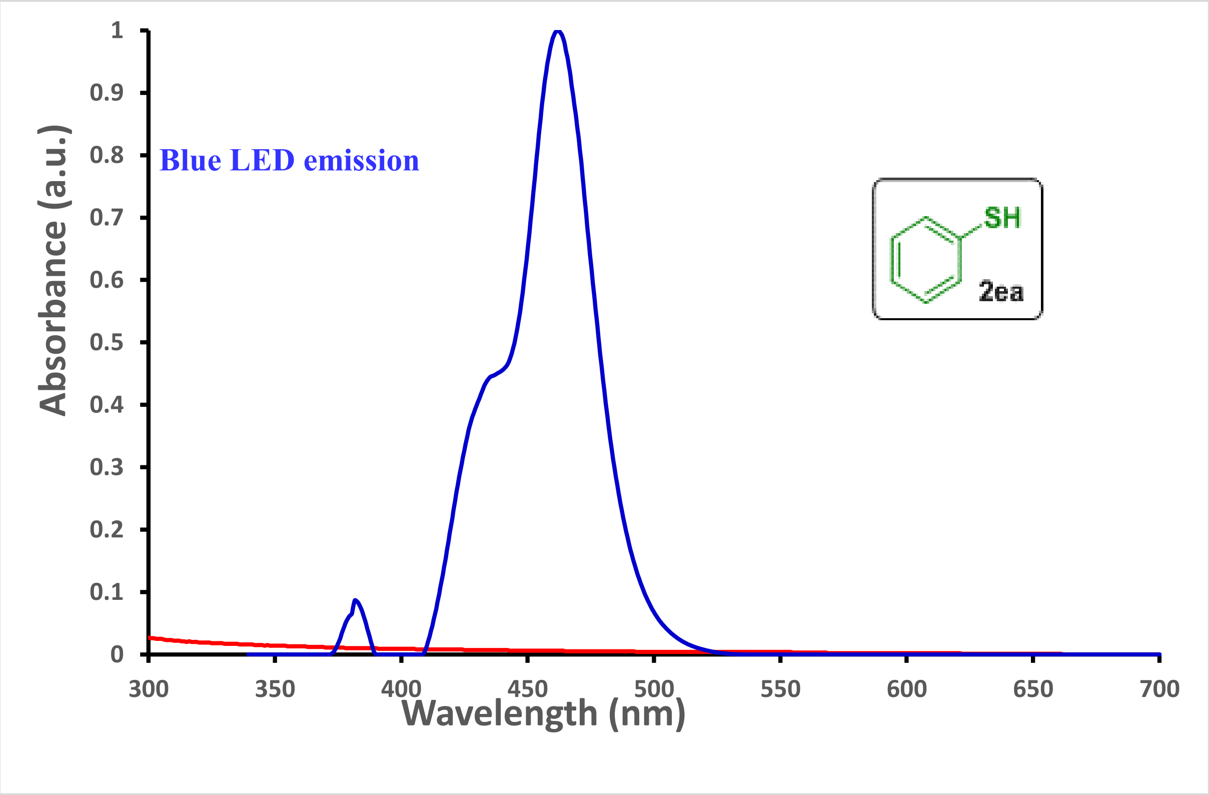

Supplementary Figure 44. Absorption spectra of benzenethiol (**2ea**) (10^{-5} M) in DCM, and blue LED emission.

1.9.2.7. Absorption spectra of 4-methyl-*N*-(phenylethynyl)-*N*-(2 (phenylethynyl)phenyl)benzenesulfonamide (1a) in DCM

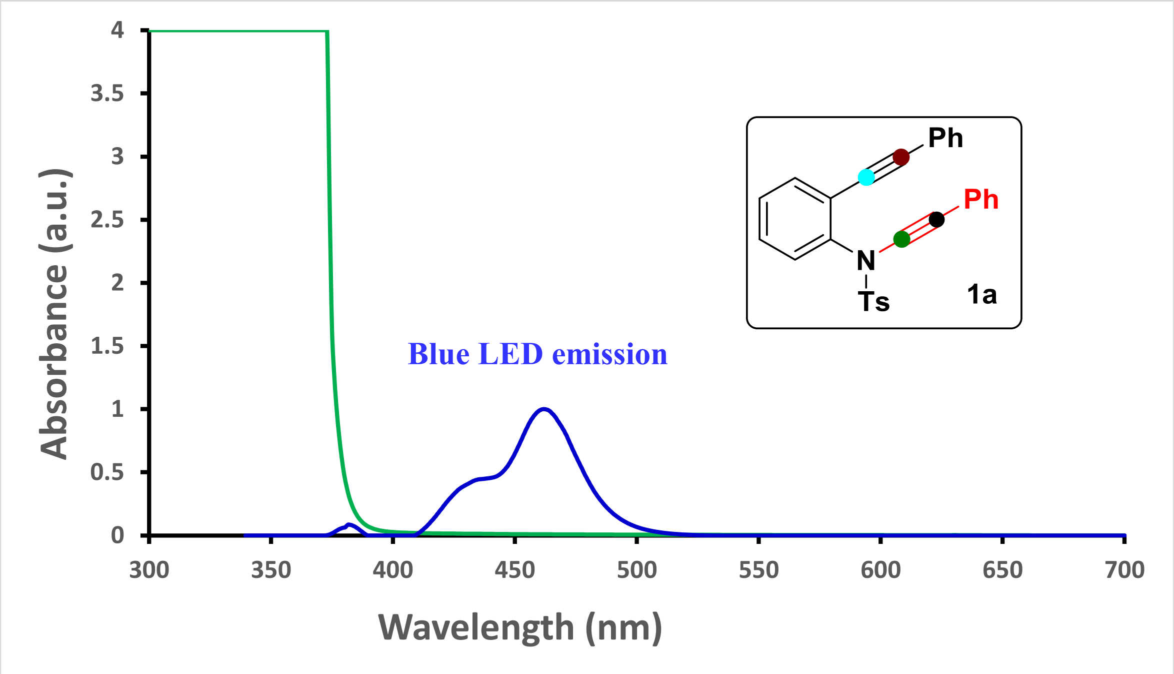

Supplementary Figure 45. Absorption spectra of 4-methyl-*N*-(phenylethynyl)-*N*-(2-(phenylethynyl)phenyl)benzenesulfonamide (**1a**) (10^{-2} M) in DCM, and blue LED emission.

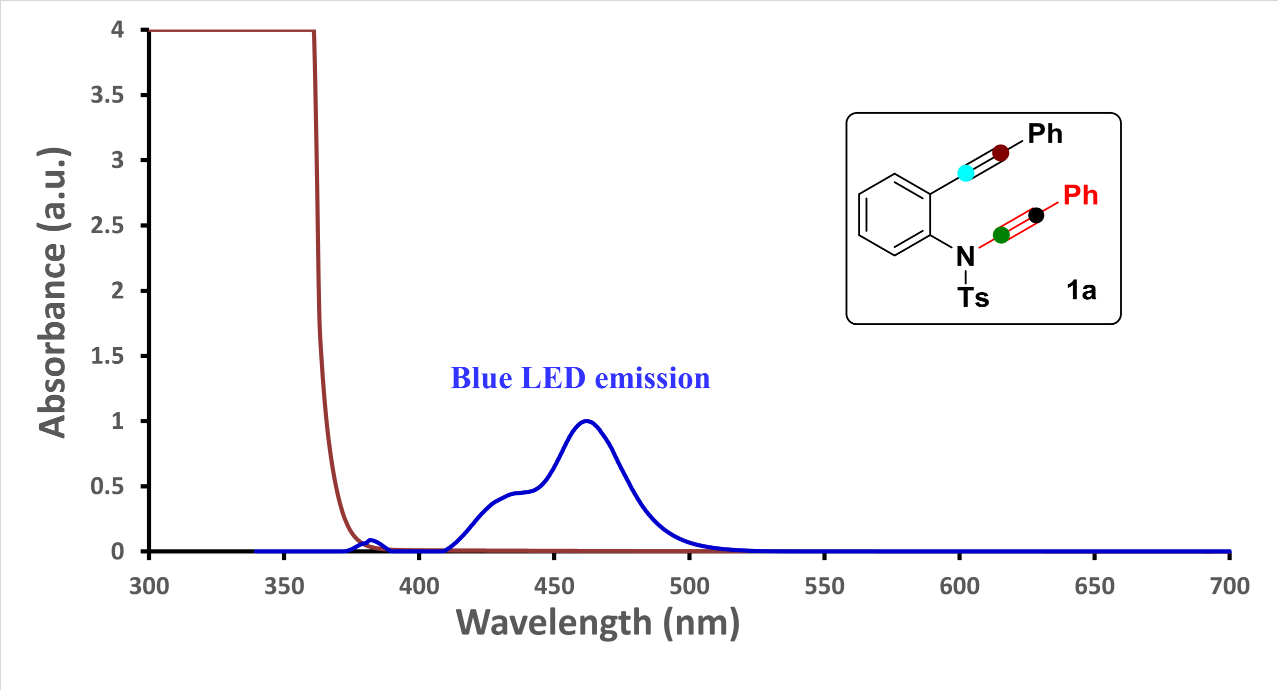

Supplementary Figure 46. Absorption spectra of 4-methyl-*N*-(phenylethynyl)-*N*-(2-(phenylethynyl)phenyl)benzenesulfonamide (**1a**) (10^{-3} M) in DCM, and blue LED emission.

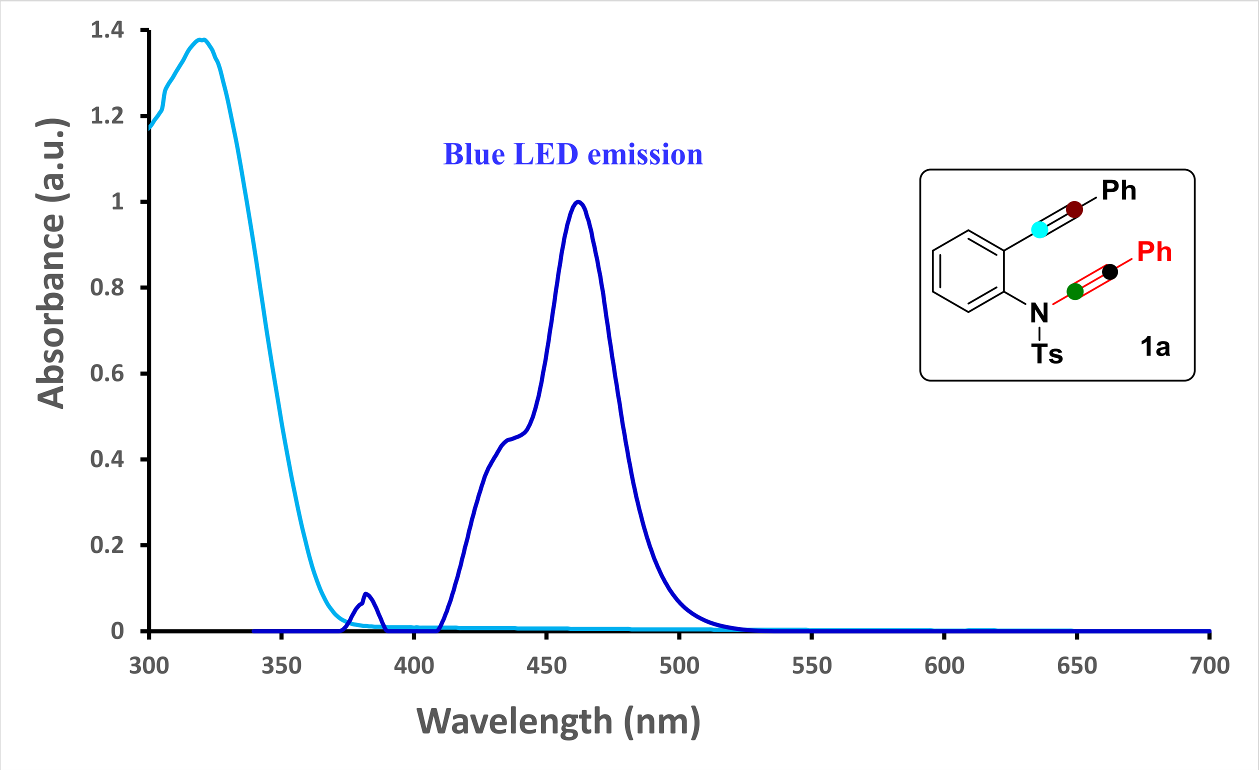

Supplementary Figure 47. Absorption spectra of 4-methyl-*N*-(phenylethynyl)-*N*-(2-(phenylethynyl)phenyl)benzenesulfonamide (**1a**) (10^{-4} M) in DCM, and blue LED emission.

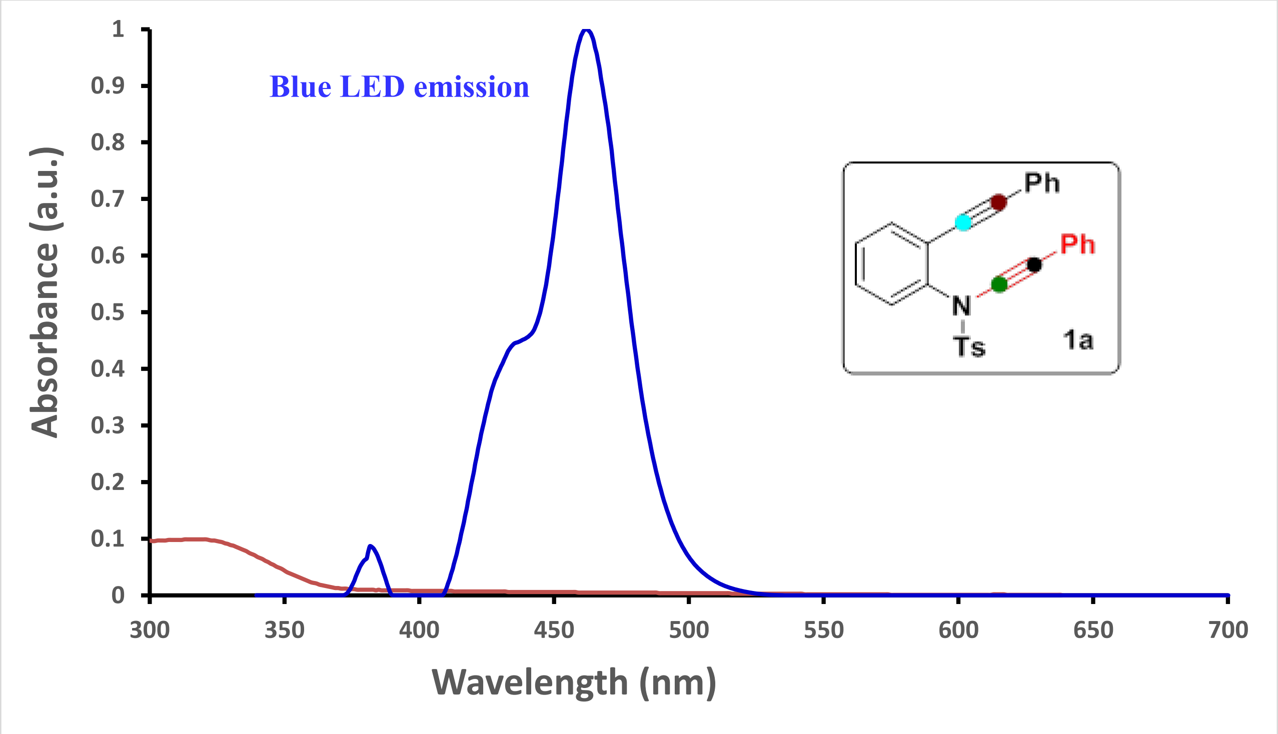

Supplementary Figure 48. Absorption spectra of 4-methyl-*N*-(phenylethynyl)-*N*-(2-(phenylethynyl)phenyl)benzenesulfonamide (**1a**) (10^{-5} M) in DCM, and blue LED emission.

1.10. Control studies for compounds 3, 51 and 76

1.10.1. Radical inhibitors studies in the synthesis of (*E*)-3-(1-iodo-2-phenyl-2-tosylvinyl)-2-phenyl-1-tosylindole (3)

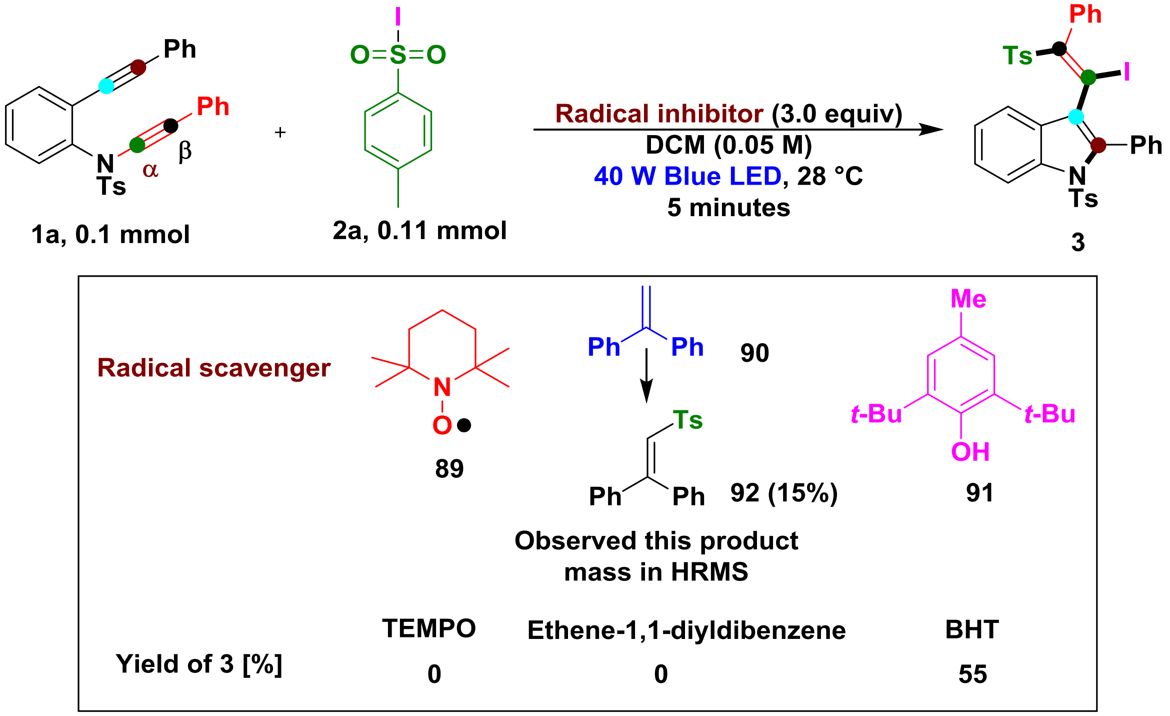

An oven-dried screw-capped, 8 mL vial equipped with a magnetic stir bar was charged with 4-methyl-*N*-(phenylethynyl)-*N*-(2-(phenylethynyl)phenyl)benzenesulfonamide (45 mg, 0.10 mmol, 1.0 equiv), 4-methylbenzenesulfonyl iodide (31 mg, 0.11 mmol, 1.1 equiv), and DCM (0.05 M) solvent was added. The resulting solution was stirred up to starting material completion (10-30 minutes) at 28 °C under a blue LED light (the reaction mixture vial placed ~8.5 cm away from the LED light with clip fan for cooling). (three seperate reactions were performed with three different radical inhibitors). After the standard reaction time, with ethyene-1,1-diylidibenzene reaction, the crude reaction mixture was diluted with water and extracted with DCM. The organic layer was dried over Na_2SO_4, filtered, and concentrated. The crude material was purified by flash column chromatography using hexane-ethyl acetate (80:20) as the eluent gave the radical traped product (2-tosylethene-1,1-diyl)dibenzene as white solid in 15% (8 mg)

Elemental Composition Report Page 1

Single Mass Analysis
Tolerance = 500.0 PPM / DBE: min = -10.0, max = 100.0
Element prediction: Off
Number of isotope peaks used for i-FIT = 3

Monoisotopic Mass, Even Electron Ions
481 formula(e) evaluated with 287 results within limits (up to 20 closest results for each mass)
Elements Used:
C: 1-100 H: 1-100 O: 1-10 Na: 0-1 S: 1-5 I: 0-1

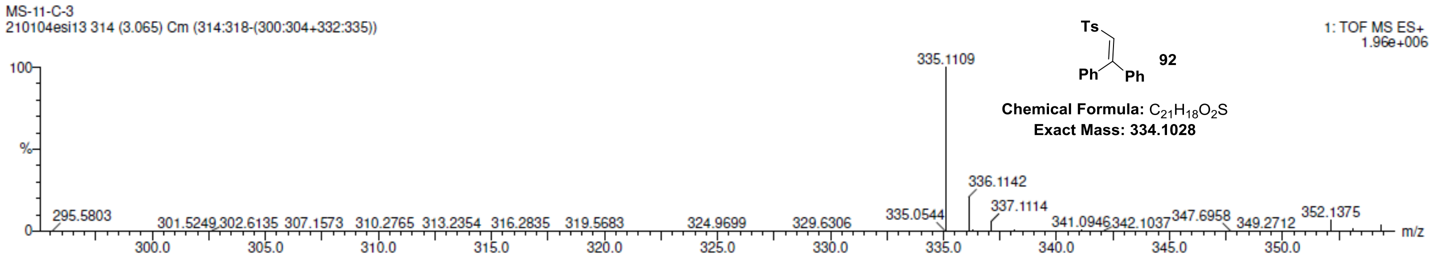

Minimum:				-10.0	
Maximum:		5.0	500.0	100.0	

Mass	Calc. Mass	mDa	PPM	DBE	Formula
335.1109	335.1106	0.3	0.9	12.5	C21 H19 O2 S
	335.1115	-0.6	-1.8	4.5	C16 H24 O2 Na S2
	335.1117	-0.8	-2.4	-7.5	C8 H32 O3 S I
	335.1082	2.7	8.1	9.5	C19 H20 O2 Na S
	335.1139	-3.0	-9.0	7.5	C18 H23 O2 S2
	335.1140	-3.1	-9.3	0.5	C12 H24 O7 Na S
	335.1149	-4.0	-11.9	-0.5	C13 H28 O2 Na S3
	335.1054	5.5	16.4	-6.5	C8 H31 O5 S4
	335.1164	-5.5	-16.4	3.5	C14 H23 O7 S
	335.1046	6.3	18.8	-5.5	C7 H27 O10 S2
	335.1173	-6.4	-19.1	2.5	C15 H27 O2 S3
	335.1174	-6.5	-19.4	-4.5	C9 H28 O7 Na S2
	335.1183	-7.4	-22.1	-5.5	C10 H32 O2 Na S4
	335.1030	7.9	23.6	-9.5	C6 H32 O5 Na S4
	335.1022	8.7	26.0	-8.5	C5 H28 O10 Na S2
	335.1021	8.8	26.3	-1.5	C11 H27 O5 S3
	335.1198	-8.9	-26.6	-1.5	C11 H27 O7 S2
	335.1012	9.7	28.9	-0.5	C10 H23 O10 S
	335.1207	-9.8	-29.2	-2.5	C12 H31 O2 S4
	335.1208	-9.9	-29.5	-9.5	C6 H32 O7 Na S3

Supplementary Figure 49. HRMS spectra with ethene-1,1-diyldibenzene as radical inhibitor under standard condition after purification.

1.10.1a. Additional mechanistic studies for the synthesis of (*E*)-3-(1-iodo-2-phenyl-2-tosylvinyl)-2-phenyl-1-tosylindole (3)

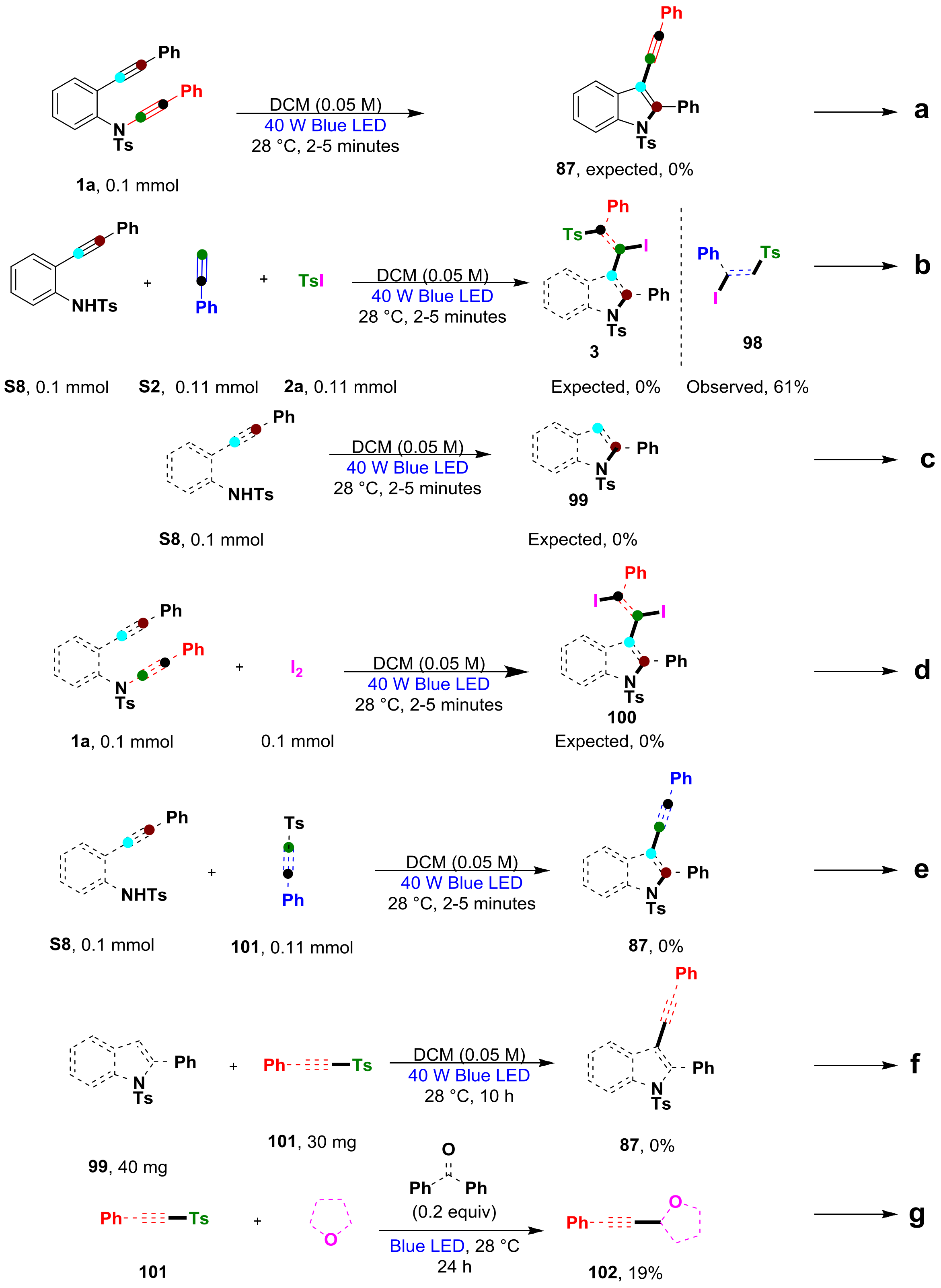

Supplementary Figure 50. Additional mechanistic studies.

1.10.2. Radical inhibitors studies in the synthesis of (*E*)-2-phenyl-3-(2-phenyl-1-(phenylselanyl)-2-tosylvinyl)-1-tosylindole (51)

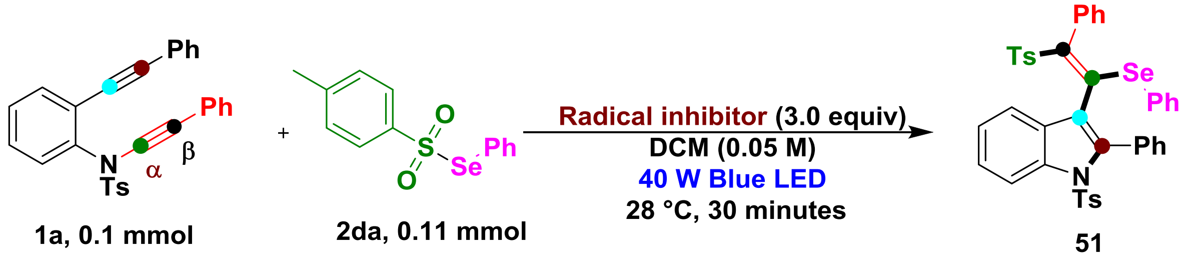

Radical scavenger

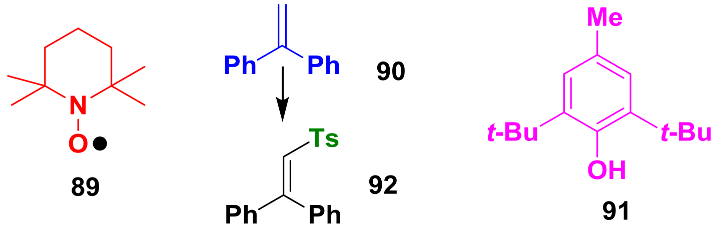

Observed this product mass in LCMS

	TEMPO	Ethene-1,1-diyldibenzene	BHT
Yield of 51 [%]	0	0	43

An oven-dried screw-capped, 8 mL vial equipped with a magnetic stir bar was charged with 4-methyl-*N*-(phenylethynyl)-*N*-(2-(phenylethynyl)phenyl)benzenesulfonamide (45 mg, 0.10 mmol, 1.0 equiv), Se-phenyl 4-methylbenzenesulfonoselenoate (34 mg, 0.11 mmol, 1.1 equiv), and DCM (0.05 M) solvent was added. The resulting solution was stirred up to starting material completion (10-30 minutes) at 28 °C under a blue LED light (the reaction mixture vial placed ~8.5 cm away from the LED light with clip fan for cooling). (three seperate reactions were performed with three different radical inhibitors). After the standard reaction time, with ethyene-1,1-diylidibenzene reaction crude was submitted to LCMS for analysis. The LCMS spectra are as follows.

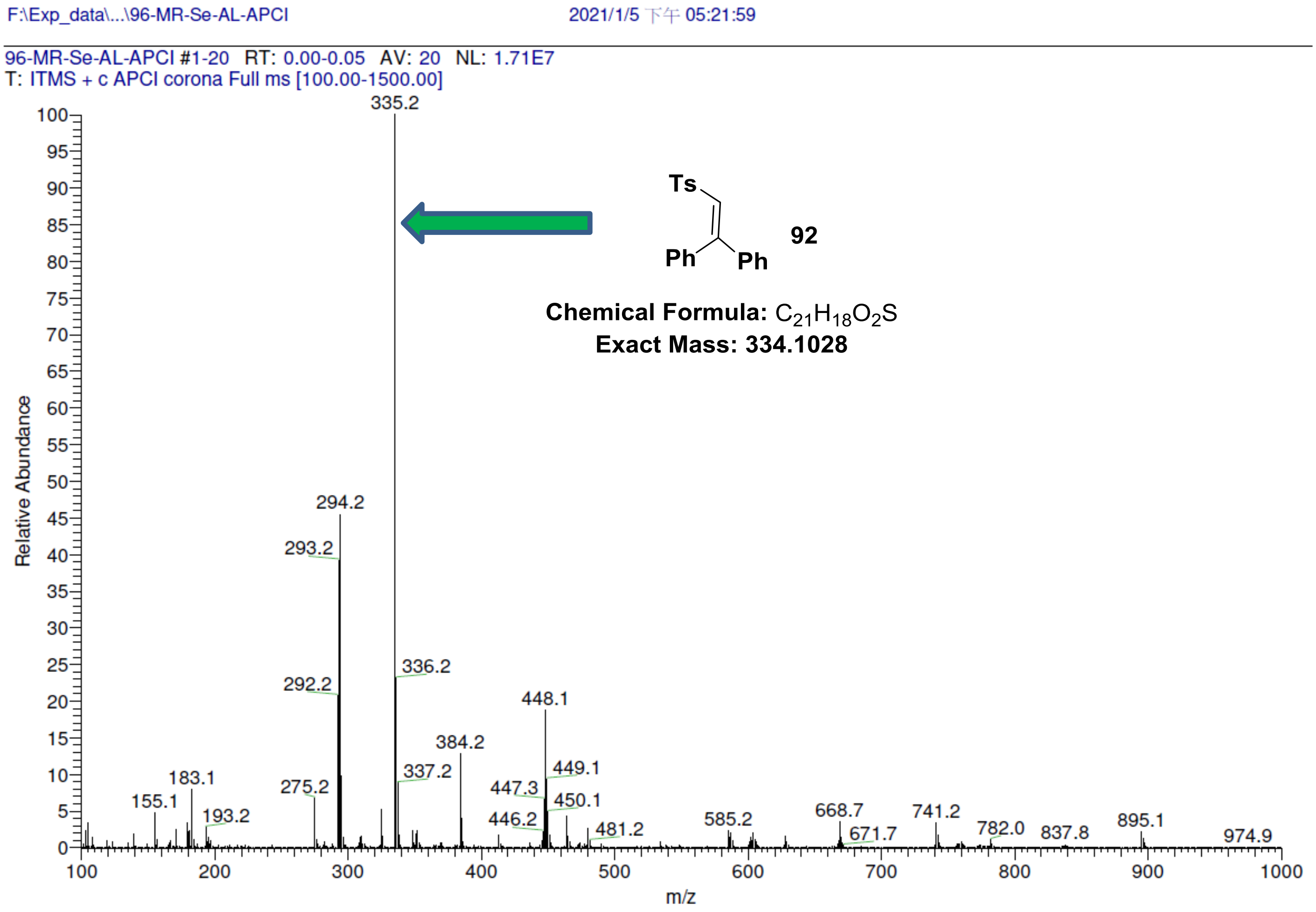

92

Chemical Formula: $C_{21}H_{18}O_2S$
Exact Mass: 334.1028

F:\Exp_data\...\96-MR-Se-AL-APCI 2021/1/5 下午 05:21:59

96-MR-Se-AL-APCI#1-20 RT: 0.00-0.05 AV: 20
T: ITMS + c APCI corona Full ms [100.00-1500.00]
m/z= 100.0-1000.0

m/z	Intensity	Relative
103.2	405998.3	2.38
105.1	579555.8	3.40
108.2	241769.5	1.42
139.1	316574.4	1.86
155.1	808615.9	4.74
171.1	420824.0	2.47
179.1	586855.5	3.44
180.2	379313.2	2.22
181.2	400202.9	2.35
183.1	1352910.2	7.93
193.2	473277.2	2.78
195.2	252318.0	1.48
275.2	1150945.1	6.75
292.2	3539583.0	20.76
293.2	6689359.8	39.23
294.2	7753463.5	45.47
295.2	1681270.4	9.86
296.2	249998.2	1.47
309.2	249726.8	1.46
310.2	280177.1	1.64
325.2	901753.0	5.29
326.2	256076.3	1.50
335.2	17052852.9	100.00
336.2	3942680.6	23.12
337.2	1503508.3	8.82
338.2	306307.0	1.80
348.1	408363.2	2.39
351.2	311896.9	1.83
352.1	397652.1	2.33
384.2	2191428.2	12.85
385.2	676152.0	3.97
413.0	297843.6	1.75
446.2	379618.6	2.23
447.3	1128475.8	6.62
448.1	3201983.2	18.78
449.1	1596071.9	9.36
450.1	834984.1	4.90
451.1	308638.5	1.81
464.1	727267.2	4.26
465.1	272366.7	1.60
480.2	452210.1	2.65
585.2	387412.5	2.27
587.3	355591.7	2.09
604.0	356043.1	2.09
628.2	273477.7	1.60
668.7	593615.6	3.48
669.8	254089.8	1.49
741.2	573171.0	3.36
742.2	301986.3	1.77
895.1	365076.6	2.14

Supplementary Figure 51. LCMS spectra with ethene-1,1-diyldibenzene as radical inhibitor under standard condition.

1.10.3. Radical inhibitors studies in the synthesis of (*E*/*Z*)-2-phenyl-3-(2-phenyl-2-(phenylthio)vinyl)-1-tosylindole

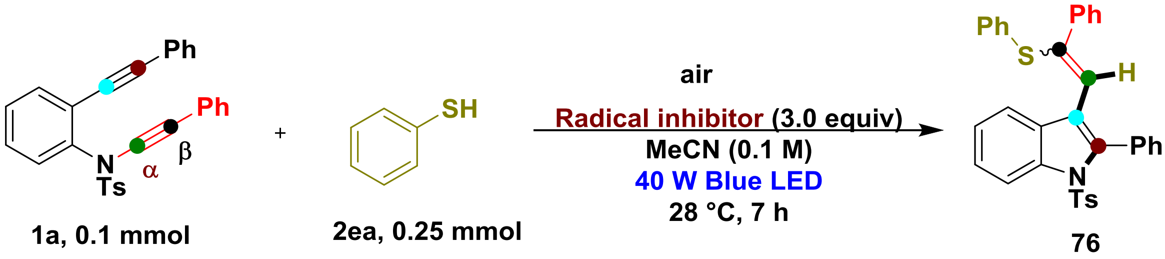

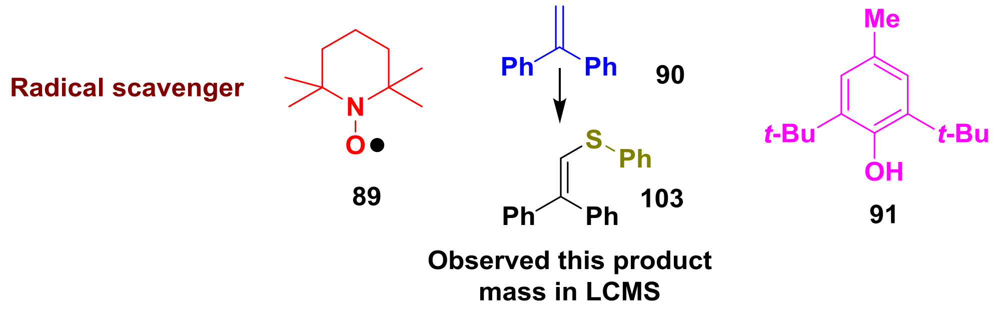

	TEMPO	Ethene-1,1-diyldibenzene	BHT
Yield of 76 [%]	0	0	35

An oven-dried screw-capped, 8 mL vial equipped with a magnetic stir bar was charged with 4-methyl-*N*-(phenylethynyl)-*N*-(2-(phenylethynyl)phenyl)benzenesulfonamide (45 mg, 0.10 mmol, 1.0 equiv), thiophenol (28 mg, 0.25 mmol, 2.5 equiv), and DCM (0.05 M) solvent was added. The resulting solution was stirred up to starting material completion (10-30 minutes) at 28 °C under a blue LED light (the reaction mixture vial placed ~8.5 cm away from the LED light with clip fan for cooling). (Three seperate reactions were performed with three different radical inhibitors) After the standard reaction time, the reaction crude was submitted to LCMS for analysis. The LCMS spectra are as follows.

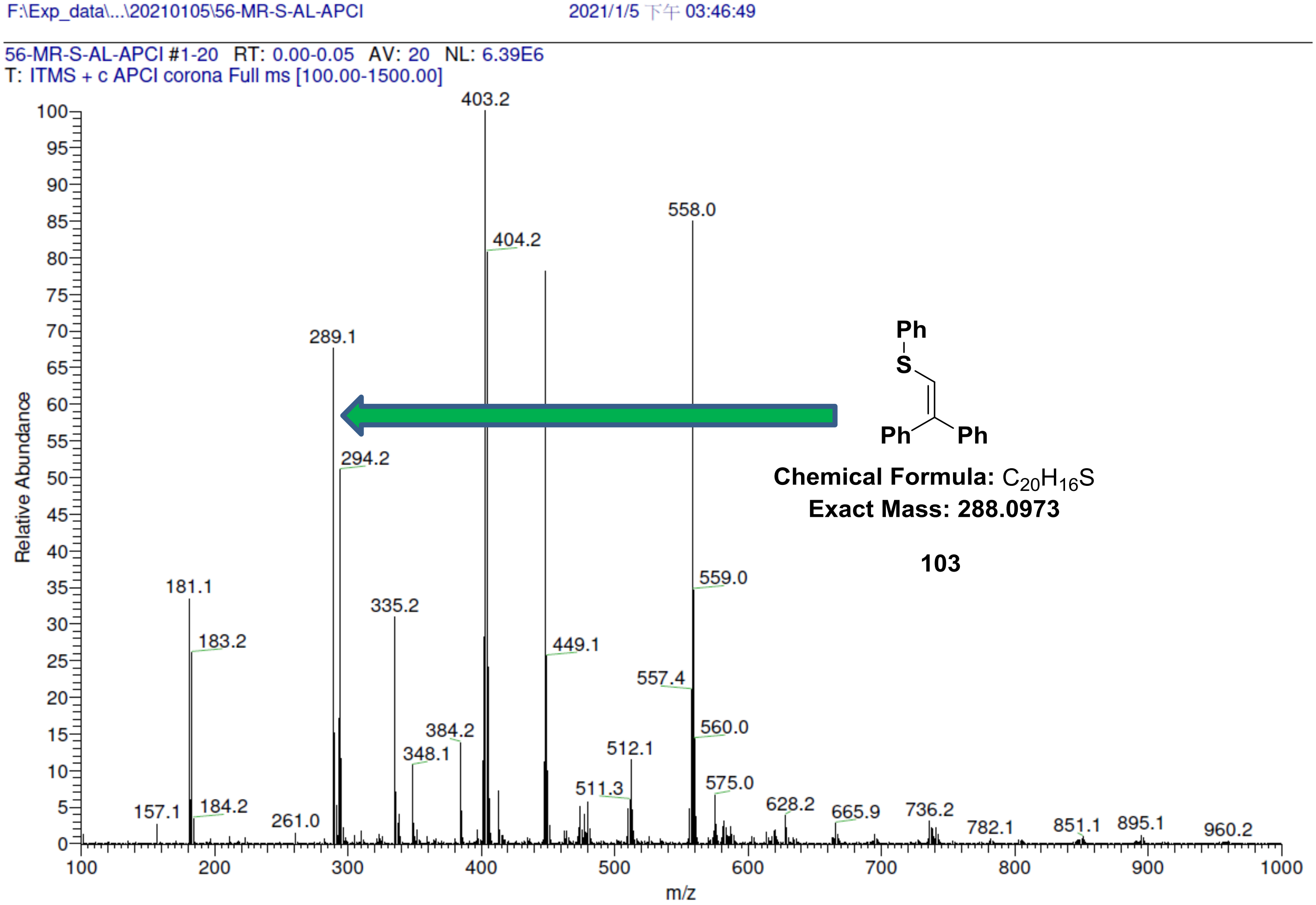

Chemical Formula: $C_{20}H_{16}S$
Exact Mass: 288.0973

103

F:\Exp_data\...\20210105\56-MR-S-AL-APCI 2021/1/5 下午 03:46:49

56-MR-S-AL-APCI#1-20 RT: 0.00-0.05 AV: 20
T: ITMS + c APCI corona Full ms [100.00-1500.00]
m/z= 100.0-1000.0

m/z	Intensity	Relative
157.1	171868.8	2.69
181.1	2138997.2	33.46
182.3	382495.7	5.98
183.2	1666366.8	26.07
184.2	221709.3	3.47
289.1	4319253.3	67.57
290.2	969812.0	15.17
291.2	331371.0	5.18
293.2	1088700.2	17.03
294.2	3260360.8	51.01
295.2	745538.1	11.66
335.2	1973212.8	30.87
336.2	453563.1	7.10
337.2	173394.1	2.71
338.4	253112.2	3.96
348.1	681369.5	10.66
349.2	173400.9	2.71
384.2	882289.7	13.80
385.3	287653.6	4.50
401.2	719377.4	11.25
402.3	1799377.0	28.15
403.2	6391816.9	100.00
404.2	5164726.3	80.80
405.2	1542886.3	24.14
406.2	391524.6	6.13
413.0	465255.9	7.28
447.4	712085.2	11.14
448.0	4991119.1	78.09
449.1	1635091.9	25.58
450.1	632069.0	9.89
451.1	159649.8	2.50
474.3	325754.7	5.10
477.2	253366.8	3.96
480.1	366609.7	5.74
510.2	307938.0	4.82
511.3	382410.3	5.98
512.1	731030.7	11.44
513.1	297779.1	4.66
556.2	308449.0	4.83
557.4	1342144.7	21.00
558.0	5429523.0	84.94
559.0	2215570.6	34.66
560.0	914825.2	14.31
561.0	241214.8	3.77
575.0	425448.8	6.66
576.0	169989.6	2.66
582.2	201120.1	3.15
628.2	248678.8	3.89
665.9	177371.4	2.77
736.2	194423.1	3.04

Supplementary Figure 52. LCMS spectra with ethene-1,1-diyldibenzene as radical inhibitor in presence of Se-phenyl 4-methylbenzenesulfonoselenoate.

1.10.3.1. Reaction of 4-methyl-*N*-(phenylethynyl)-*N*-(2-(phenylethynyl)phenyl)benzenesulfonamide with 1,2-diphenyldisulfane

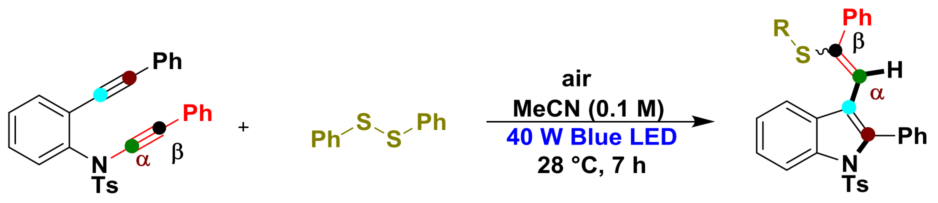

1a, 0.1 mmol **S20a, 0.05 mmol** **76, 0%**

An oven-dried screw-capped, 8 mL vial equipped with a magnetic stir bar was charged with 4-methyl-*N*-(phenylethynyl)-*N*-(2-(phenylethynyl)phenyl)benzenesulfonamide (45 mg, 0.10 mmol, 1.0 equiv) 1,2-diphenyldisulfane (11 mg, 0.05 mmol, 0.5 equiv), MeCN (0.1 M) was added. The resulting solution was stirred under a blue LED light (the reaction mixture vial was placed ~8.5 cm away from the LED light with a clip fan for cooling) at 28 °C. The progress of the reaction by TLC (both starting material intact in the reaction mixutre).

1.10.3.2. Reaction of 4-methyl-*N*-(phenylethynyl)-*N*-(2-(phenylethynyl)phenyl)benzenesulfonamide with 1,2-di-*p*-tolyldisulfane and benzenethiol

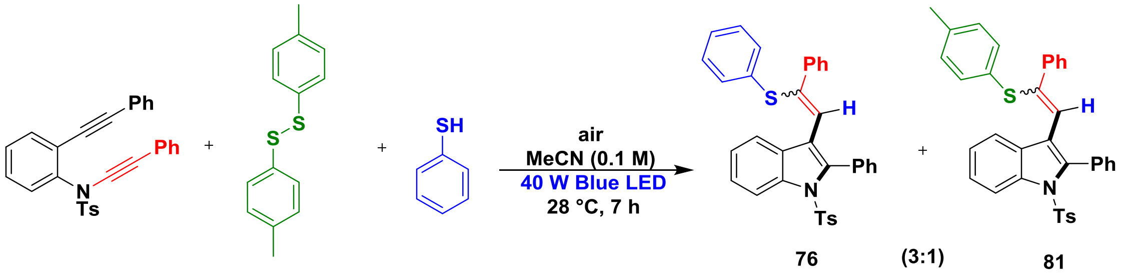

1a, 0.2 mmol 0.1 mmol, **S20** 0.2 mmol, **2ea**

76 **(3:1)** **81**

***E*/*Z*=12:88** ***E*/*Z*=11:89**

Mixture of the compounds
(Both products are same R_F on TLC)

An oven-dried screw-capped, 8 mL vial equipped with a magnetic stir bar was charged with 4-methyl-*N*-(phenylethynyl)-*N*-(2-(phenylethynyl)phenyl)benzenesulfonamide (90 mg, 0.20 mmol, 1.0 equiv) 1,2-di-*p*-tolyldisulfane (25 mg, 0.10 mmol, 0.5 equiv), thiophenol (22 mg, 0.20 mmol, 1.0 equiv) MeCN (0.1 M) was added. The resulting solution was stirred under a blue LED light (the reaction mixture vial was placed ~8.5 cm away from the LED light with a clip fan for cooling) up to starting material completion at 28 °C. After that, the crude reaction mixture was diluted with water and extracted with ethyl acetate. The organic layer was dried over Na_2SO_4, filtered, and concentrated. The crude material was purified by flash column chromatography using hexane-ethyl acetate (89:11) as the eluent gave the mixture of products (*E*/Z)-2-phenyl-3-(2-phenyl-2-(phenylthio)vinyl)-1-tosylindole (**76**) (major) and (*E*/*Z*)-2-phenyl-3-(2-phenyl-2-(p-tolylthio)vinyl)-1-tosylindole (**81**) as a white solid (minor) in 44% yield with 3.2:1 ration. Note: Both products are same R_F on TLC.

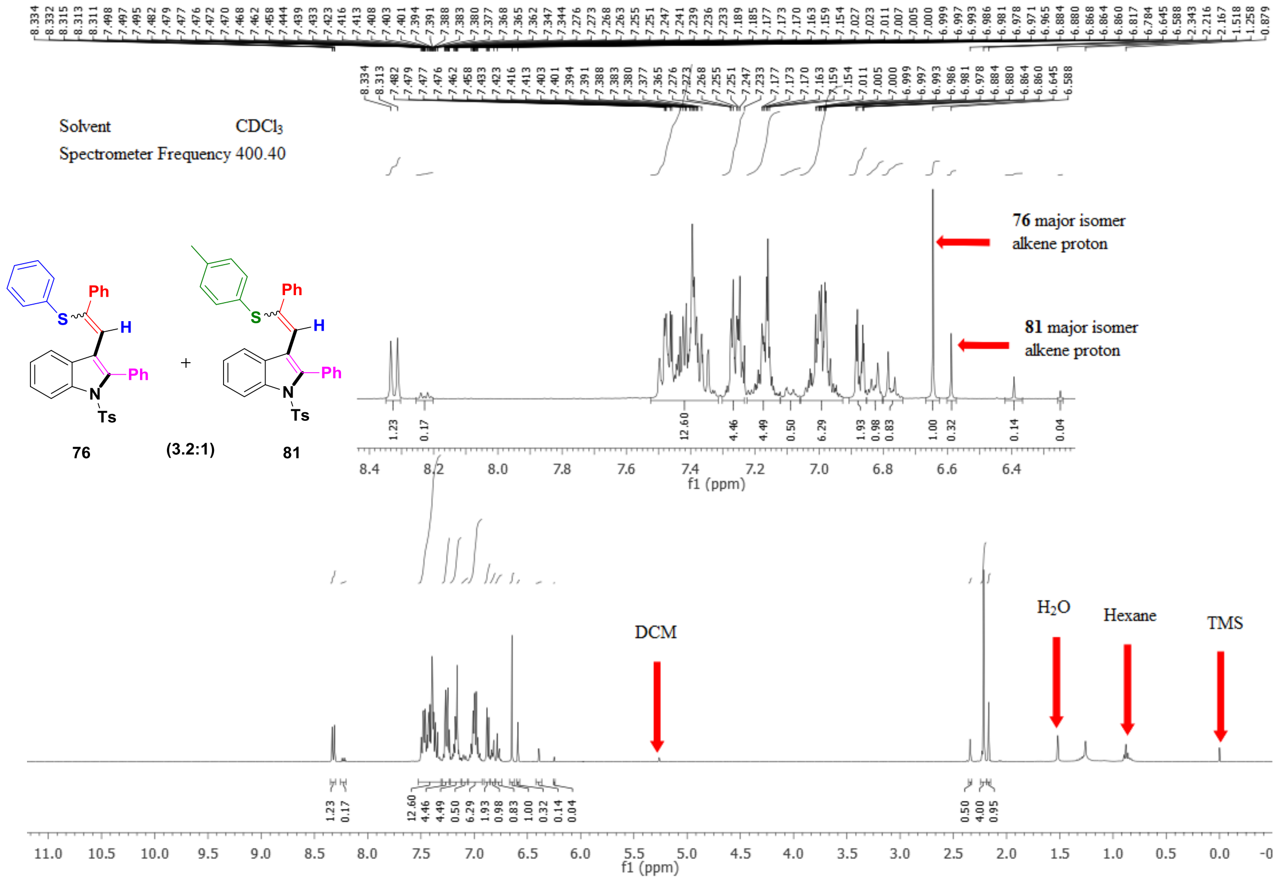

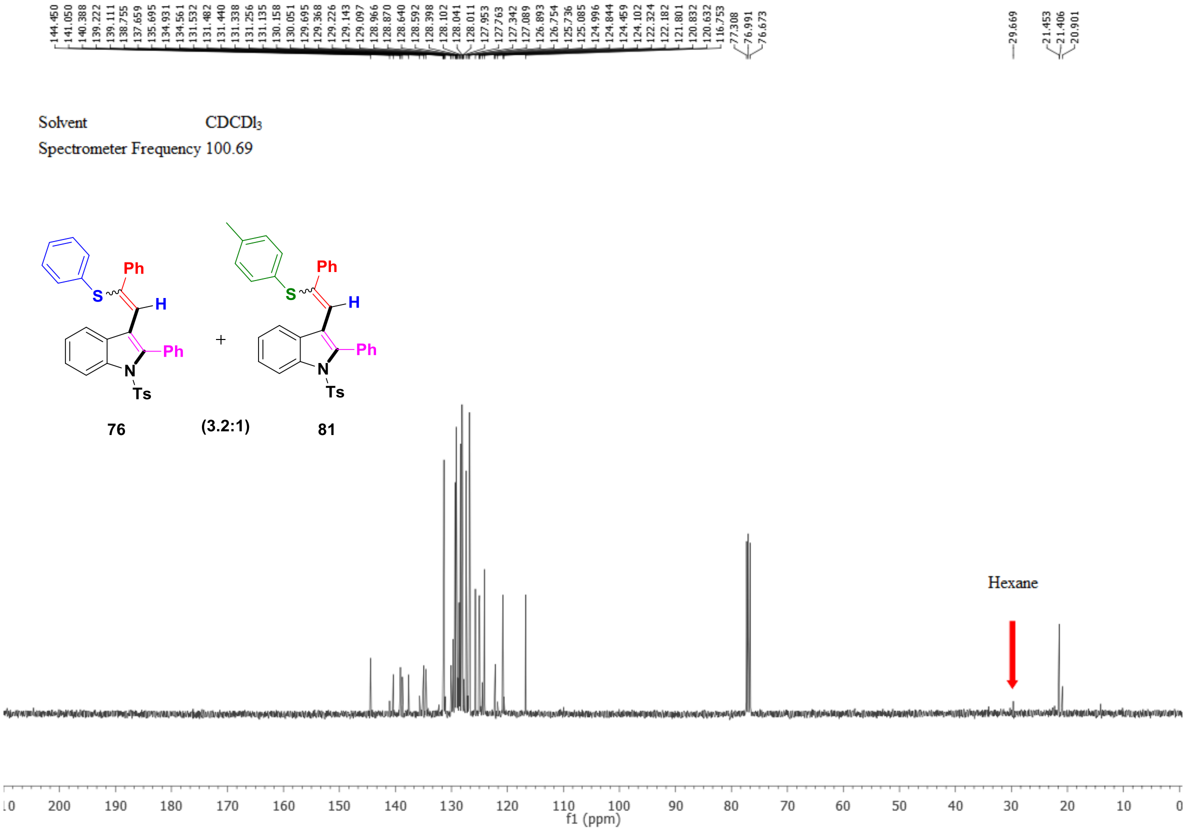

Supplementary Figure 53. ^{1}H NMR and ^{13}CNMR spectra of mixture of **76** and **81**.

1.10.3.3. Reaction of 4-methyl-*N*-(phenylethynyl)-*N*-(2-(phenylethynyl)phenyl)benzenesulfonamide with 1,2-di-*p*-tolyldisulfane and benzenethiol under nitrogen atmosphere

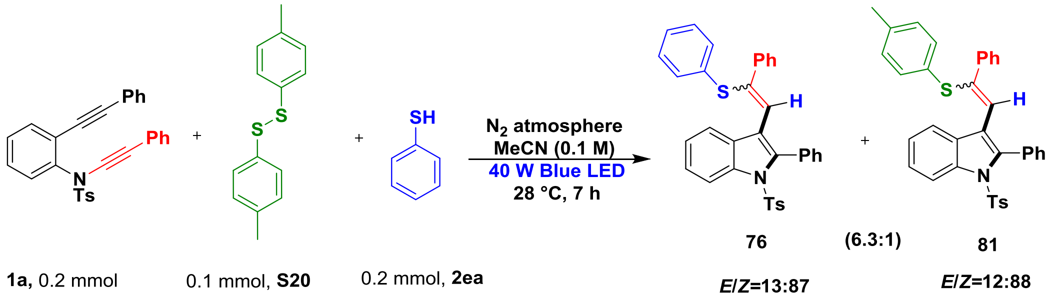

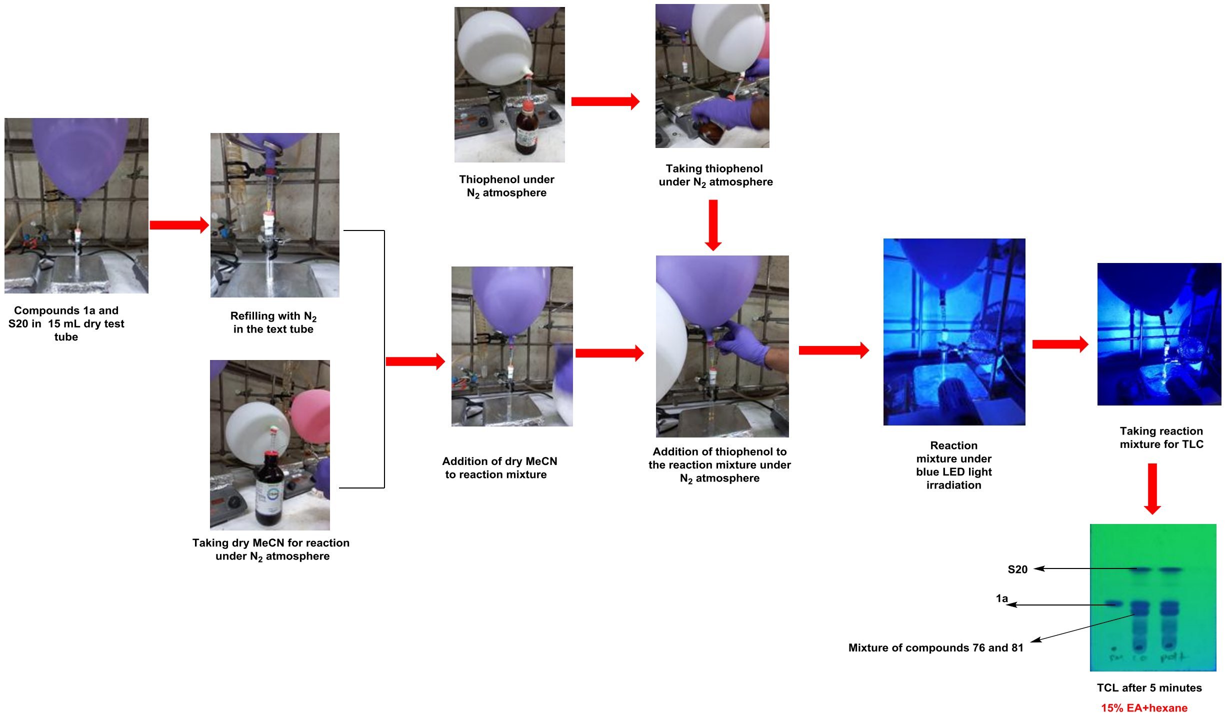

An oven-dried screw-capped, 8 mL vial equipped with a magnetic stir bar was charged with 4-methyl-*N*-(phenylethynyl)-*N*-(2-(phenylethynyl)phenyl)benzenesulfonamide (90 mg, 0.20 mmol, 1.0 equiv) 1,2-di-*p*-tolyldisulfane (25 mg, 0.10 mmol, 0.5 equiv) under nitrogen atmosphere. The reaction mixture test tube was refilled with nitrogen and MeCN (0.1 M) was added under nitrogen atmosphere. Finally compound thiophenol (22 mg, 0.20 mmol, 1.0 equiv) was added under nitrogen atmosphere. The resulting solution was stirred under a blue LED light (the reaction mixture vial was placed ~8.5 cm away from the LED light with a clip fan for cooling) up to starting material completion at 28 °C under nitrogen atmosphere. After standard reaction time the progress of the reaction was monitored by TLC. After that, the crude reaction mixture was diluted with water and extracted with ethyl acetate. The organic layer was dried over Na_2SO_4, filtered, and concentrated. The crude material was purified by flash column chromatography using hexane-ethyl acetate (90:10) as the eluent gave the mixture of products (*E*/Z)-2-phenyl-3-(2-phenyl-2-(phenylthio)vinyl)-1-tosylindole (**76**) (major) and (*E*/*Z*)-2-

phenyl-3-(2-phenyl-2-(p-tolylthio)vinyl)-1-tosylindole (**81**) as a white solid (minor) in 39% yield with 6.3:1 ration.

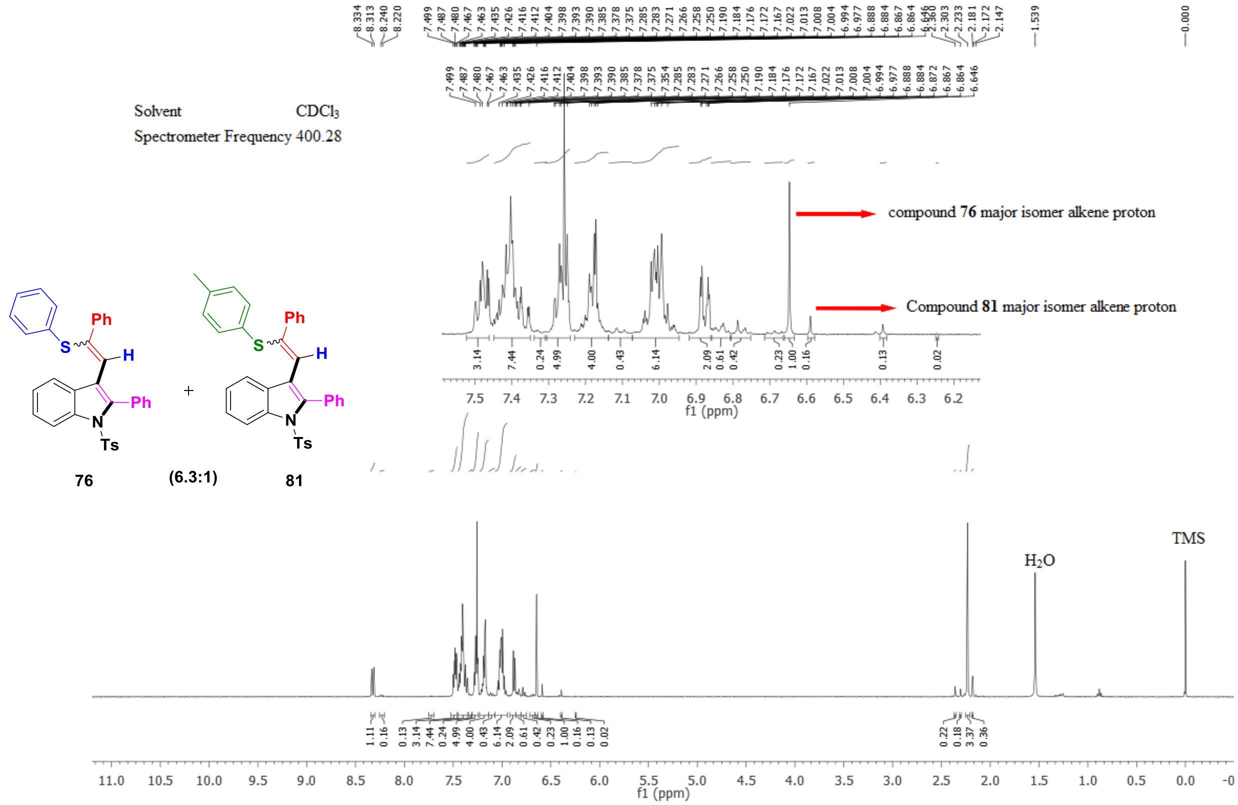

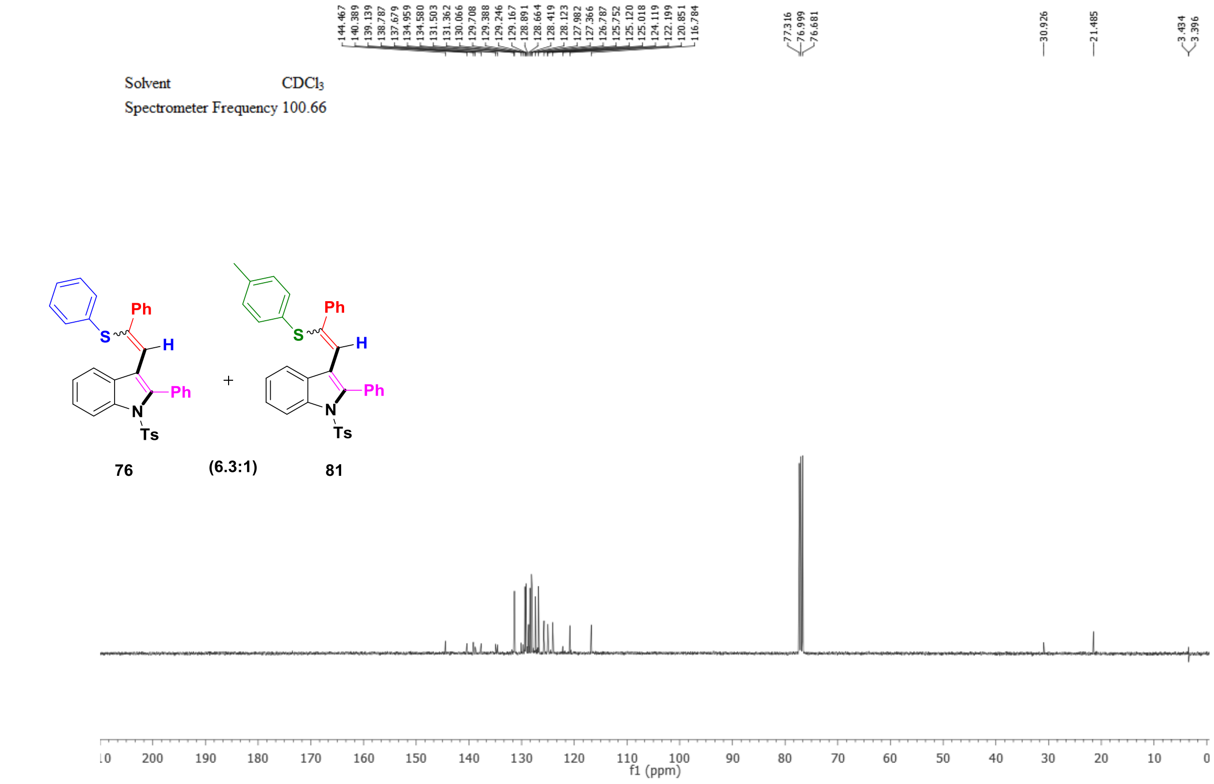

Supplementary Figure 54. ^{1}H NMR and ^{13}CNMR spectra of mixture of **76** and **81**.

1.11. Plausible reaction mechanism for compound 76 synthesis

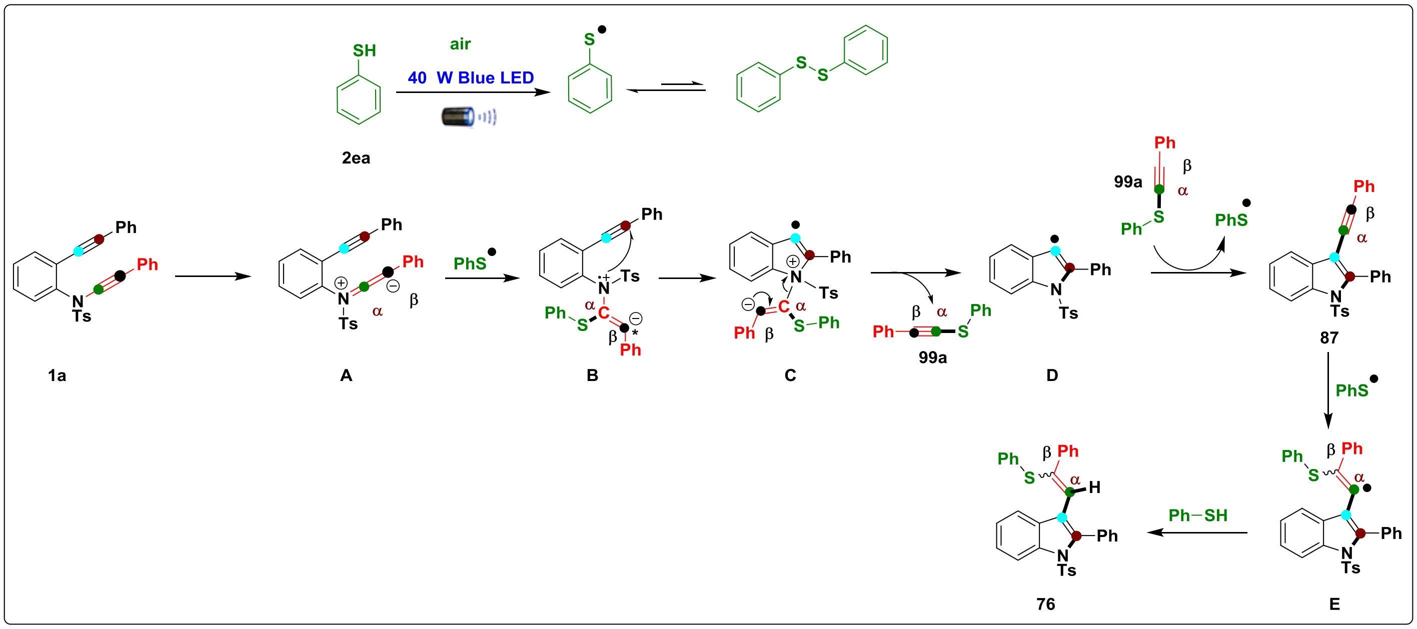

Supplementary Figure 55. Plausible reaction mechanism for synthesis of compound **76**.

1.12. Characterization data

4-methyl-*N*-(phenylethynyl)-*N*-(2-(phenylethynyl)phenyl)benzenesulfonamide (1a): The title compound

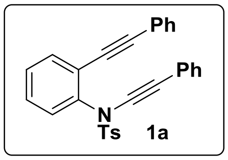

was prepared according to the general procedure A via column chromatography of silica eluting hexane-ethyl acetate (99:1) (Note: long run is needed to remove side product indole because two products are very close on TLC) to obtain as a brown solid; Mp. 130-135 °C; ^{1}H NMR (400 MHz, $CDCl_3$) δ 8.35 (d, *J* = 8.3 Hz, 1H), 7.87 (s, 1H), 7.79 – 7.67 (m, 6H), 7.54 – 7.49 (m, 3H), 7.48 – 7.42 (m, 3H), 7.41 – 7.35 (m, 2H), 7.29 (d, *J* = 8.4 Hz, 2H), 7.03 (d, *J* = 8.0 Hz, 2H), 2.26 (s, 3H). ^{13}C NMR (101 MHz, $CDCl_3$) δ 144.85, 143.46, 137.10, 134.42, 131.37, 131.20, 130.69, 130.66, 129.30, 129.06, 128.25, 128.24, 127.25, 126.80, 125.74, 124.69, 123.03, 120.08, 116.61, 108.19, 94.68, 81.43, 21.51; HRMS (ESI) calcd for $C_{29}H_{22}NO_2S$ $[M+H]^+$ 448.1371; found: 448.1370.

4-methyl-*N*-(4-methyl-2-(phenylethynyl)phenyl)-*N*-(phenylethynyl)benzenesulfonamide (1b): The title

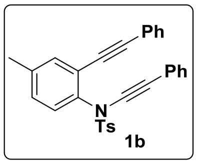

compound was prepared according to the general procedure A via column chromatography of silica eluting hexane-ethyl acetate (98:2) to obtain as a brown solid (The product and indole side product are similar R_F on TLC); Mp. 145-147 °C; ^{1}H NMR (400 MHz, $CDCl_3$) δ 8.20 (d, *J* = 8.5 Hz, 1H), 7.67 (d, *J* = 6.1 Hz, 2H), 7.45 (d, *J* = 7.0 Hz, 4H), 7.34 (d, *J* = 3.6 Hz, 2H), 7.28 – 7.16 (m, 6H), 6.95 (d, *J* = 8.1 Hz, 2H), 2.41 (s, 3H), 2.16 (s, 3H). ^{13}C NMR (101 MHz, $CDCl_3$) δ 144.66, 143.49, 135.21, 134.46, 134.18, 131.25, 131.06, 130.82, 130.68, 129.15, 128.91, 128.14, 127.12, 127.07, 126.63, 122.90, 119.87, 116.23, 107.98, 94.56, 81.52, 21.30, 21.15; HRMS (ESI) calcd for $C_{30}H_{24}NO_2S$ $[M+H]^+$ 462.1528; found: 462.1519.

***N*-(4-ethyl-2-(phenylethynyl)phenyl)-4-methyl-*N*-(phenylethynyl)benzenesulfonamide (1c):** The title

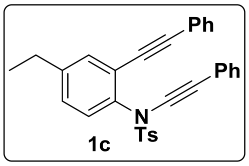

compound was prepared according to the general procedure A via column chromatography of silica eluting hexane-ethyl acetate (97:3) to obtain as a brown solid (The product and indole side product are similar R_F on TLC); Mp. 151-152 °C; ^{1}H NMR (400 MHz, $CDCl_3$) δ 8.22 (d, *J* = 8.6 Hz, 1H), 7.64 (ddd, *J* = 4.5, 2.4, 1.5 Hz, 2H), 7.47 (ddd, *J* = 5.8, 3.9, 1.8 Hz, 4H), 7.40 – 7.35 (m, 2H), 7.30 – 7.25 (m, 6H), 7.04 (d, *J* = 8.0 Hz, 2H), 2.77 (q, *J* = 7.6 Hz, 2H), 2.28 (s, 3H), 1.30 (t, *J* = 7.6 Hz, 3H). ^{13}C NMR (101 MHz, $CDCl_3$) δ 144.74, 143.57, 141.03, 135.43, 134.50, 131.41, 131.21, 130.84, 130.77, 129.29, 128.99, 128.25, 128.20, 127.22, 126.84, 126.09, 123.13, 118.71, 116.43, 108.10, 94.57, 81.64, 28.74, 21.53, 15.94. HRMS (EI) calcd for $C_{31}H_{26}NO_2S$ $[M+H]^+$ 476.1684; found: 476.1683.

***N*-(4-butyl-2-(phenylethynyl)phenyl)-4-methyl-*N*-(phenylethynyl)benzenesulfonamide (1d):** The title

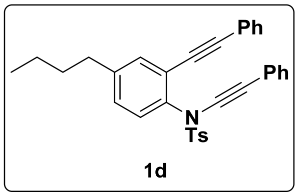

compound was prepared according to the general procedure A via column chromatography of silica eluting hexane-ethyl acetate (99:1) to obtain as a brown gummy compound (The product and indole side product are similar R_F on TLC); ^{1}H NMR (400 MHz, $CDCl_3$) δ 8.21 (d, *J* = 8.6 Hz, 1H), 7.67 – 7.60 (m, 2H), 7.50 – 7.45 (m, 3H), 7.44 – 7.42 (m, 1H), 7.40 – 7.34 (m, 2H), 7.29 – 7.24 (m, 5H), 7.23 (d, *J* = 1.8 Hz, 1H), 7.06 – 7.00 (m,

2H), 2.72 (t, *J* = 8.0 Hz, 2H), 2.27 (s, 3H), 1.69 – 1.61 (m, 2H), 1.37 (q, *J* = 7.6 Hz, 2H), 0.94 (t, *J* = 7.4 Hz, 3H). ^{13}C NMR (101 MHz, $CDCl_3$) δ 144.73, 143.56, 139.69, 135.44, 134.50, 131.40, 131.20, 130.80, 130.78, 129.26, 128.98, 128.24, 128.19, 127.21, 126.83, 126.55, 123.13, 119.27, 116.33, 108.08, 94.56, 81.65, 35.52, 33.94, 22.39, 21.51, 13.96. HRMS (ESI) calcd for $C_{33}H_{30}NO_2S$ $[M+H]^+$ 504.1997; found: 504.1995.

***N*-(4-chloro-2-(phenylethynyl)phenyl)-4-methyl-*N*-(phenylethynyl)benzenesulfonamide (1e):** The title

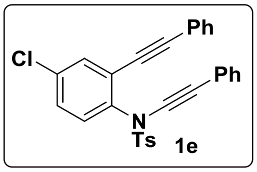

compound was prepared according to the general procedure A via column chromatography of silica eluting hexane-ethyl acetate (98:2) to obtain as a pale orange solid; Mp. 163-164 °C; ^{1}H NMR (400 MHz, $CDCl_3$) δ 8.30 (d, *J* = 8.9 Hz, 1H), 7.73 – 7.65 (m, 3H), 7.54 – 7.49 (m, 3H), 7.41 – 7.35 (m, 3H), 7.27 (dd, *J* = 6.0, 2.7 Hz, 4H), 7.04 (d, *J* = 8.2 Hz, 2H), 2.24 (s, 3H). ^{13}C NMR (101 MHz, $CDCl_3$) δ 145.08, 144.53, 135.30, 134.04, 131.84, 131.27, 131.11, 130.49, 130.06, 129.32, 129.26, 128.31, 128.16, 127.22, 126.64, 126.56, 125.79, 122.56, 119.63, 117.62, 107.31, 95.00, 80.58, 21.36. HRMS (ESI) calcd for $C_{29}H_{21}NO_2SCl$ $[M+H]^+$ 482.0982; found: 482.0979.

***N*-(4-bromo-2-(phenylethynyl)phenyl)-4-methyl-*N*-(phenylethynyl)benzenesulfonamide (1f):** The title

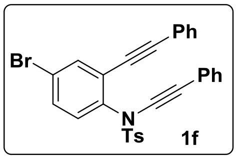

compound was prepared according to the general procedure A via column chromatography of silica eluting hexane-ethyl acetate (97:3) to obtain as an brown solid; Mp. 152-153 °C; ^{1}H NMR (400 MHz, $CDCl_3$) δ 8.20 (d, *J* = 8.9 Hz, 1H), 7.78 (d, *J* = 1.6 Hz, 1H), 7.63 (ddd, *J* = 4.8, 2.4, 1.4 Hz, 2H), 7.52 – 7.46 (m, 4H), 7.37 – 7.33 (m, 2H), 7.30 – 7.25 (m, 4H), 7.05 (d, *J* = 8.0 Hz, 2H), 2.28 (s, 3H). ^{13}C NMR (101 MHz, $CDCl_3$) δ 145.17, 144.44, 135.78, 134.17, 132.33, 131.38, 131.21, 130.09, 129.43, 129.35, 129.27, 128.56, 128.40, 128.26, 127.29, 126.76, 122.79, 122.69, 118.28, 118.03, 107.25, 95.01, 80.61, 21.52. HRMS (ESI) calcd for $C_{29}H_{19}NO_2SBr$ $[M+H]^+$ 524.0320; found: 524.0319.

4-methyl-*N*-(phenylethynyl)-*N*-(2-(p-tolylethynyl)phenyl)benzenesulfonamide (1g): The title compound

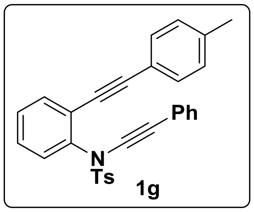

was prepared according to the general procedure A via column chromatography of silica eluting hexane-ethyl acetate (98:2) to obtain as a orange solid (The product and indole side product are similar R_F on TLC); Mp. 158-160 °C; ^{1}H NMR (400 MHz, $CDCl_3$) δ 8.32 (d, *J* = 8.3 Hz, 1H), 7.65 (d, *J* = 7.0 Hz, 1H), 7.56 (d, *J* = 8.1 Hz, 2H), 7.39 (tdd, *J* = 10.2, 7.9, 4.4 Hz, 4H), 7.33 – 7.27 (m, 7H), 7.05 (d, *J* = 8.2 Hz, 2H), 2.47 (s, 3H), 2.29 (s, 3H). ^{13}C NMR (101 MHz, $CDCl_3$) δ 144.79, 143.74, 139.10, 137.11, 134.40, 131.42, 131.06, 130.91, 129.28, 128.26, 128.21, 128.05, 127.77, 126.83, 125.60, 124.68, 119.99, 116.71, 94.63, 81.61, 21.60, 21.54. HRMS (ESI) calcd for $C_{30}H_{24}NO_2S$ $[M+H]^+$ 462.1521; found: 462.1521.

***N*-(2-((4-methoxyphenyl)ethynyl)phenyl)-4-methyl-*N*-(phenylethynyl)benzenesulfonamide (1h):** The title

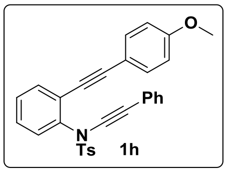

compound was prepared according to the general procedure A via column chromatography of silica eluting hexane-ethyl acetate (93:7) to obtain as a orange gummy compound (The product and indole side product are similar R_F on TLC); ^{1}H NMR (400 MHz, $CDCl_3$) δ 8.33 (d, *J* = 8.2 Hz, 1H), 7.67 – 7.57 (m, 3H), 7.41 – 7.30 (m, 4H), 7.29 – 7.22 (m, 5H), 7.04 – 6.95 (m, 4H), 3.86 (s, 3H), 2.22 (s, 3H). ^{13}C NMR (101 MHz, $CDCl_3$) δ 160.20, 144.75, 143.50, 136.99, 134.35, 132.58, 131.30,

130.80, 129.21, 128.20, 128.14, 126.70, 125.46, 124.65, 123.04, 122.85, 119.84, 116.64, 112.71, 107.45, 94.54, 81.61, 55.18, 21.41. HRMS (ESI) calcd for $C_{30}H_{24}NO_3S$ $[M+H]^+$ 478.1477; found: 478.1472.

***N*-(2-((3,4-dimethoxyphenyl)ethynyl)phenyl)-4-methyl-*N*-(phenylethynyl)benzenesulfonamide (1i):** The

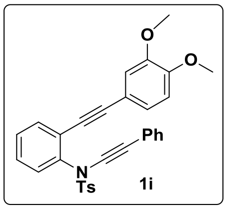

title compound was prepared according to the general procedure A via column chromatography of silica eluting hexane-ethyl acetate (90:10) to obtain as a lite yellow solid compound; Mp. 128-129 °C; ^{1}H NMR (400 MHz, $CDCl_3$) δ 8.34 (d, *J* = 8.2 Hz, 1H), 7.68 – 7.63 (m, 1H), 7.44 – 7.34 (m, 4H), 7.32 – 7.27 (m, 4H), 7.27 – 7.22 (m, 3H), 7.14 (d, *J* = 2.0 Hz, 1H), 7.05 (d, *J* = 8.1 Hz, 2H), 6.98 (d, *J* = 8.4 Hz, 1H), 4.00 (s, 3H), 3.90 (s, 3H), 2.30 (s, 3H). ^{13}C NMR (101 MHz, $CDCl_3$) δ 149.88, 147.77, 144.92, 143.62, 137.27, 134.71, 131.48, 130.89, 129.37, 128.43, 128.37, 126.99, 125.72, 124.82, 124.55, 123.20, 123.07, 120.03, 116.87, 114.71, 109.98, 107.62, 94.82, 81.80, 56.07, 56.00, 21.65. HRMS (ESI) calcd for $C_{31}H_{26}NO_4S$ $[M+H]^+$ 508.1583; found: 508.1583.

***N*-(2-(cyclopropylethynyl)phenyl)-4-methyl-*N*-(phenylethynyl)benzenesulfonamide (1j):** The title

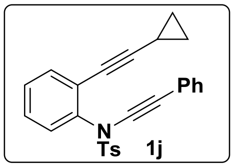

compound was prepared according to the general procedure A via column chromatography of silica eluting hexane-ethyl acetate (97:3) to obtain as a dark pink solid; Mp. 89-91 °C; ^{1}H NMR (400 MHz, $CDCl_3$) δ 8.25 (d, *J* = 7.7 Hz, 1H), 7.72 (d, *J* = 8.5 Hz, 2H), 7.67 – 7.63 (m, 1H), 7.56 – 7.52 (m, 2H), 7.40 – 7.29 (m, 5H), 7.24 – 7.20 (m, 2H), 2.47-2.43 (m, 1H), 2.36 (s, 3H), 1.31 – 1.26 (m, 2H), 1.12 – 1.07 (m, 2H). ^{13}C NMR (101 MHz, $CDCl_3$) δ 144.89, 144.32, 136.30, 136.06, 131.13, 129.97, 129.72, 128.37, 128.20, 126.59, 125.11, 123.81, 123.38, 119.47, 114.64, 103.32, 96.39, 81.23, 21.54, 9.87, 8.44. HRMS (ESI) calcd for $C_{26}H_{22}NO_2S$ $[M+H]^+$ 412.1371; found: 412.1367.

4-methyl-*N*-(2-(phenylethynyl)phenyl)-*N*-(p-tolylethynyl)benzenesulfonamide (1k): The title compound

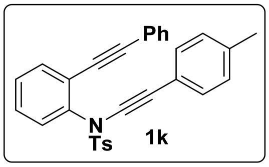

was prepared according to the general procedure A via column chromatography of silica eluting hexane-ethyl acetate (97:3) to obtain as a orange solid (The product and indole side product are similar R_F on TLC); Mp. 158-160 °C; ^{1}H NMR (400 MHz, $CDCl_3$) δ 8.33 (d, *J* = 8.3 Hz, 1H), 7.66 (ddd, *J* = 6.1, 2.1, 1.4 Hz, 3H), 7.49 – 7.45 (m, 3H), 7.41 (ddd, *J* = 8.4, 7.3, 1.4 Hz, 1H), 7.34 (td, *J* = 7.6, 1.1 Hz, 1H), 7.26 (t, *J* = 7.9 Hz, 4H), 7.07 (d, *J* = 7.9 Hz, 2H), 7.02 (d, *J* = 8.0 Hz, 2H), 2.31 (s, 3H), 2.25 (s, 3H). ^{13}C NMR (101 MHz, $CDCl_3$) δ 144.80, 143.17, 138.43, 137.12, 134.37, 131.27, 131.18, 130.79, 130.72, 129.27, 129.01, 128.98, 127.21, 126.77, 126.71, 125.69, 124.66, 120.10, 119.93, 116.61, 108.43, 94.91, 80.70, 21.48, 21.45. HRMS (ESI) calcd for $C_{30}H_{24}NO_2S$ $[M+H]^+$ 462.1528; found: 462.1521

(*N*-((4-ethylphenyl)ethynyl)-4-methyl-*N*-(2-(phenylethynyl)phenyl)benzenesulfonamide (1l): The title

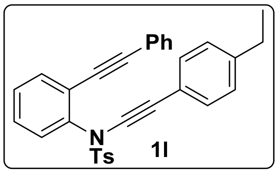

compound was prepared according to the general procedure A via column chromatography of silica eluting hexane-ethyl acetate (96:4) to obtain as a orange solid (The product and indole side product are similar R_F on TLC); Mp. 123-124 °C; ^{1}H NMR (400 MHz, $CDCl_3$) δ 8.33 (d, *J* = 8.1 Hz, 1H), 7.68 – 7.63 (m, 3H), 7.47 (dd, *J* = 4.9, 1.8 Hz, 3H), 7.44 – 7.39 (m, 1H), 7.37 – 7.32 (m, 1H), 7.28 (dd, *J* = 8.4, 3.1 Hz, 4H), 7.11 (d, *J* = 8.5 Hz, 2H), 7.03 (d, *J* = 8.0 Hz, 2H), 2.61 (q, *J* = 7.6 Hz, 2H), 2.26 (s, 3H), 1.20 (t, *J* = 7.6 Hz, 3H). ^{13}C NMR (101 MHz, $CDCl_3$) δ 144.80,

144.75, 143.19, 137.12, 134.39, 131.38, 131.19, 130.73, 129.28, 128.99, 127.83, 127.22, 126.79, 125.69, 124.66, 120.18, 120.11, 116.62, 108.44, 94.92, 80.69, 28.78, 21.50, 15.31. HRMS (ESI) calcd for $C_{31}H_{26}NO_2S$ $[M+H]^+$ 476.1684; found: 476.1674.

***N*-((3-methoxyphenyl)ethynyl)-4-methyl-*N*-(2-(phenylethynyl)phenyl)benzenesulfonamide (1m):** The title

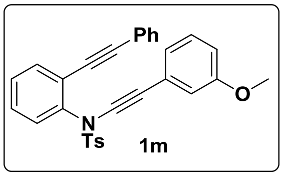

compound was prepared according to the general procedure A via column chromatography of silica eluting hexane-ethyl acetate (95:5) to obtain as a orange solid (The product and indole side product are similar R_F on TLC); Mp. 117-118 ℃; ^{1}H NMR (400 MHz, $CDCl_3$) δ 8.36 – 8.32 (m, 1H), 7.69 – 7.63 (m, 3H), 7.51 – 7.46 (m, 3H), 7.42 (ddd, *J* = 8.5, 5.0, 1.3 Hz, 1H), 7.38 – 7.34 (m, 1H), 7.30 – 7.26 (m, 2H), 7.22 – 7.16 (m, 1H), 7.05 (dd, *J* = 8.6, 0.6 Hz, 2H), 6.98 – 6.94 (m, 1H), 6.89 – 6.82 (m, 2H), 3.78 (s, 3H), 2.29 (s, 3H). ^{13}C NMR (101 MHz, $CDCl_3$) δ 159.23, 144.87, 143.60, 137.12, 134.48, 131.24, 130.67, 129.33, 129.09, 127.26, 126.83, 125.75, 124.70, 124.06, 123.97, 120.10, 116.63, 116.33, 114.70, 108.12, 94.60, 81.33, 77.31, 76.99, 76.67, 55.25, 21.54. HRMS (ESI) calcd for $C_{27}H_{28}NO_3S_2$ $[M+H]^+$ 478.1511; found: 478.1470.

***N*-((4-methoxyphenyl)ethynyl)-4-methyl-*N*-(2-(phenylethynyl)phenyl)benzenesulfonamide (1n):** The title

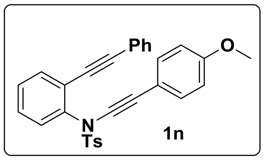

compound was prepared according to the general procedure A via column chromatography of silica eluting hexane-ethyl acetate (95:5) to obtain as a orange solid (The product and indole side product are similar R_F on TLC); Mp. 115-117 ℃; (dd, *J* = 5.0, 1.9 Hz, 3H), 7.40 (ddd, *J* = 9.0, 5.2, 1.6 Hz, 1H), 7.35 (dd, *J* = 7.6, 1.0 Hz, 1H), 7.28 (dd, *J* = 9.8, 8.7 Hz, 4H), 7.02 (d, *J* = 8.0 Hz, 2H), 6.80 (d, *J* = 8.9 Hz, 2H), 3.76 (s, 3H), 2.25 (s, 3H). ^{13}C NMR (101 MHz, $CDCl_3$) δ 159.57, 144.78, 142.89, 137.13, 134.32, 132.83, 131.15, 130.81, 130.78, 129.25, 128.93, 127.19, 127.17, 126.75, 125.66, 124.64, 120.08, 116.61, 115.08, 113.90, 108.57, 94.73, 80.00, 55.21, 21.46. HRMS (ESI) calcd for $C_{30}H_{24}NO_3S$ $[M+H]^+$ 478.1477; found: 478.1472.

4-methyl-*N*-(2-(phenylethynyl)phenyl)-*N*-((3,4,5-trimethoxyphenyl)ethynyl)benzenesulfonamide (1o): T

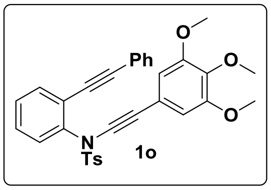

The title compound was prepared according to the general procedure A via column chromatography of silica eluting hexane-ethyl acetate (80:20) to obtain as a yellow solid; Mp. 140-141 ℃; ^{1}H NMR (400 MHz, $CDCl_3$)) δ 8.34 (d, *J* = 8.3 Hz, 1H), 7.70 –7.63 (m, 3H), 7.48 (dd, *J* = 4.9, 1.8 Hz, 3H), 7.43 (ddd, *J* = 8.4, 7.3, 1.4 Hz, 1H), 7.37 (dd, *J* = 7.6, 1.0 Hz, 1H), 7.28 (d, *J* = 8.4 Hz, 2H), 7.05 (d, *J* = 8.0 Hz, 2H), 6.58 (s, 2H), 3.84 (s, 3H), 3.82 (s, 6H), 2.29 (s, 3H). ^{13}C NMR (101 MHz, $CDCl_3$) δ 152.99, 144.87, 143.49, 138.86, 137.08, 134.43, 131.28, 130.75, 130.57, 129.31, 129.02, 127.20, 126.81, 125.76, 124.66, 120.07, 118.04, 116.62, 108.59, 108.08, 94.68, 80.65, 60.93, 56.08, 21.52. HRMS (ESI) calcd for $C_{37}H_{33}NO_4S_2ClSe$ $[M+H]^+$ 734.0705; found: 734.0713.

4-methyl-*N*-((3-nitrophenyl)ethynyl)-*N*-(2-(phenylethynyl)phenyl)benzenesulfonamide (1p): The title

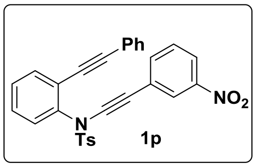

compound was prepared according to the general procedure A via column chromatography of silica eluting hexane-ethyl acetate (92:8) to obtain as a pale yellow

solid; Mp. 164-165 °C; ^{1}H NMR (400 MHz, $CDCl_3$) δ 8.35 (d, *J* = 8.3 Hz, 1H), 8.17 (s, 1H), 8.10 (d, *J* = 8.2 Hz, 1H), 7.66 (d, *J* = 7.5 Hz, 1H), 7.61 (dt, *J* = 8.9, 4.3 Hz, 3H), 7.51 (dd, *J* = 5.1, 1.6 Hz, 3H), 7.44 (dd, *J* = 7.5, 5.5 Hz, 2H), 7.38 (t, *J* = 7.2 Hz, 1H), 7.29 (d, *J* = 8.3 Hz, 2H), 7.07 (d, *J* = 8.2 Hz, 2H), 2.29 (s, 3H). ^{13}C NMR (101 MHz, $CDCl_3$) δ 148.02, 145.05, 144.55, 136.95, 136.88, 134.54, 131.18, 130.34, 130.13, 129.41, 129.39, 129.28, 127.37, 126.81, 126.01, 125.93, 124.89, 124.79, 122.74, 119.93, 116.53, 106.93, 92.02, 84.38, 21.52. HRMS (ESI) calcd for $C_{29}H_{19}N_2O_4S$ $[M+H]^+$ 491.1066; found: 491.1066.

(methyl 4-(((4-methyl-*N*-(2-(phenylethynyl)phenyl)phenyl)sulfonamido)ethynyl)benzoate (1q): The title

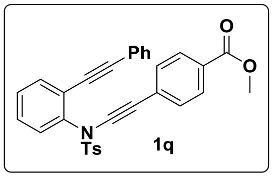

compound was prepared according to the general procedure A via column chromatography of silica eluting hexane-ethyl acetate (93:7) to obtain as a yellow solid; Mp. 182-183 °C; ^{1}H NMR (400 MHz, $CDCl_3$) δ 8.34 (d, *J* = 8.3 Hz, 1H), 7.95 (d, *J* = 8.6 Hz, 2H), 7.69 – 7.62 (m, 3H), 7.52 – 7.48 (m, 3H), 7.43 (ddd, *J* = 8.4, 7.3, 1.4 Hz, 1H), 7.40 – 7.35 (m, 3H), 7.28 (d, *J* = 8.4 Hz, 2H), 7.05 (d, *J* = 8.0 Hz, 2H), 3.90 (s, 3H), 2.28 (s, 3H). ^{13}C NMR (101 MHz, $CDCl_3$) δ 166.40, 144.96, 144.18, 137.02, 134.46, 131.21, 131.19, 130.45, 130.35, 129.41, 129.35, 129.32, 129.26, 127.73, 127.30, 126.79, 125.85, 124.75, 119.99, 116.57, 107.55, 93.91, 84.68, 52.19, 21.52. HRMS (ESI) calcd for $C_{31}H_{24}NO_4S$ $[M+H]^+$ 506.1426; found: 506.1426.

***N*-((4-acetylphenyl)ethynyl)-4-methyl-*N*-(2-(phenylethynyl)phenyl)benzenesulfonamide (1r):** The title

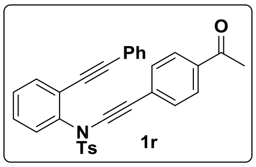

compound was prepared according to the general procedure A via column chromatography of silica eluting hexane-ethyl acetate (93:7) to obtain as a pale green solid; Mp. 167-170 °C; ^{1}H NMR (400 MHz, $CDCl_3$) δ 8.34 (d, *J* = 8.3 Hz, 1H), 7.86 (d, *J* = 8.6 Hz, 2H), 7.65 (dddd, *J* = 7.4, 3.5, 1.8, 0.8 Hz, 3H), 7.53 – 7.48 (m, 3H), 7.46 – 7.34 (m, 4H), 7.29 (d, *J* = 8.4 Hz, 2H), 7.05 (d, *J* = 8.0 Hz, 2H), 2.56 (s, 3H), 2.28 (s, 3H). ^{13}C NMR (101 MHz, $CDCl_3$) δ 197.13, 144.98, 144.25, 137.01, 136.02, 134.47, 132.62, 131.37, 131.21, 130.44, 130.29, 129.35, 129.27, 128.23, 128.16, 127.91, 127.29, 126.79, 125.87, 124.75, 119.98, 116.56, 107.49, 93.89, 85.07, 26.53, 21.50. HRMS (ESI) calcd for $C_{31}H_{24}NO_3S$ $[M+H]^+$ 490.1477; found: 490.1478.

***N*-((2,4-dichlorophenyl)ethynyl)-4-methyl-*N*-(2-(phenylethynyl)phenyl)benzenesulfonamide(1s):** The title

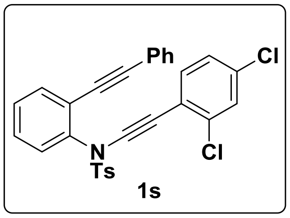

compound was prepared according to the general procedure A via column chromatography of silica eluting hexane-ethyl acetate (95:5) to obtain as a yellow solid (53 mg, yield = 70%); Mp. 166-167 °C; ^{1}H NMR (400 MHz, $CDCl_3$) δ 8.34 (d, *J* = 8.3 Hz, 1H), 7.70 (d, *J* = 7.3 Hz, 1H), 7.66 – 7.61 (m, 2H), 7.50 – 7.45 (m, 3H), 7.42 (dd, *J* = 8.3, 1.3 Hz, 1H), 7.37 (ddd, *J* = 8.2, 5.4, 0.9 Hz, 2H), 7.28 (dd, *J* = 11.1, 8.5 Hz, 3H), 7.15 (dd, *J* = 8.4, 2.0 Hz, 1H), 7.06 (d, *J* = 8.2 Hz, 2H), 2.29 (s, 3H). ^{13}C NMR (101 MHz, $CDCl_3$) δ 144.99, 144.09, 137.00, 136.24, 134.54, 134.39, 133.56, 131.27, 130.43, 130.39, 129.39, 129.24, 129.21, 127.34, 126.90, 126.84, 125.87, 124.80, 121.69, 120.11, 116.52, 107.49, 90.44, 87.75, 21.54. HRMS (ESI) calcd for $C_{29}H_{18}NO_2SCl_2$ $[M+H]^+$ 514.0435; found: 514.0436.

4-methyl-*N*-(naphthalen-2-ylethynyl)-*N*-(2-(phenylethynyl)phenyl)benzenesulfonamide (1t): The title

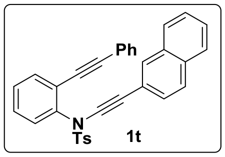

compound was prepared according to the general procedure A via column chromatography of silica eluting hexane-ethyl acetate (93:7) to obtain as a brown solid; Mp. 191-192 °C; ^{1}H NMR (400 MHz, $CDCl_3$) δ 8.35 (d, *J* = 8.3 Hz, 1H), 7.87 (s, 1H), 7.79 – 7.67 (m, 6H), 7.54 – 7.49 (m, 3H), 7.48 – 7.43 (m, 3H), 7.42 – 7.34 (m, 3H), 7.29 (d, *J* = 8.4 Hz, 2H), 7.03 (d, *J* = 8.0 Hz, 2H), 2.26 (s, 3H). ^{13}C NMR (101 MHz, $CDCl_3$) δ 144.87, 143.55, 137.14, 134.41, 132.85, 132.69, 131.24, 131.17, 130.73, 130.70, 129.31, 129.12, 128.11, 127.92, 127.70, 127.64, 127.29, 126.80, 126.66, 126.54, 125.77, 124.73, 120.32, 120.13, 116.64, 108.24, 95.16, 81.83, 21.50. HRMS (ESI) calcd for $C_{33}H_{23}NO_2NaS$ $[M+Na]^+$ 520.1347; found: 520.1348.

***N*-((6-bromobenzo[d][1,3]dioxol-5-yl)ethynyl)-4-methyl-*N*-(2-(phenylethynyl)phenyl)benzenesulfonamide**

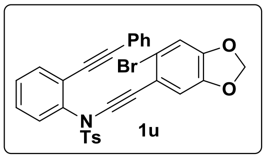

(1v): The title compound was prepared according to the general procedure A via column chromatography of silica eluting hexane-ethyl acetate (95:5) to obtain as a brown solid; Mp. 201-202 °C; ^{1}H NMR (400 MHz, $CDCl_3$) δ 8.33 (d, *J* = 8.3 Hz, 1H), 7.73 (dd, *J* = 7.6, 0.7 Hz, 1H), 7.68 – 7.62 (m, 2H), 7.50 – 7.45 (m, 3H), 7.42 (ddd, *J* = 8.4, 7.3, 1.4 Hz, 1H), 7.36 (td, *J* = 7.5, 1.0 Hz, 1H), 7.28 (d, *J* = 8.4 Hz, 2H), 7.05 (d, *J* = 8.1 Hz, 2H), 6.98 (s, 1H), 6.76 (s, 1H), 5.96 (s, 2H), 2.29 (s, 3H). ^{13}C NMR (101 MHz, $CDCl_3$) δ 148.53, 146.91, 144.90, 143.44, 137.04, 134.50, 131.27, 130.69, 130.58, 129.34, 129.10, 127.32, 126.82, 125.76, 124.74, 120.27, 118.02, 117.24, 116.52, 112.58, 112.06, 108.00, 102.05, 93.21, 84.63, 21.53. HRMS (ESI) calcd for $C_{30}H_{21}NO_4SBr$ $[M+H]^+$ 570.0375; found: 570.0377

4-methyl-*N*-(4-phenylbut-1-yn-1-yl)-*N*-(2-(phenylethynyl)phenyl)benzenesulfonamide (1w): The title

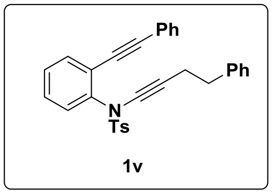

compound was prepared according to the general procedure A via column chromatography of silica eluting hexane-ethyl acetate (98:2) to obtain as a brown solid (The product and indole side product are similar R_F on TLC); Mp. 118-119 °C; ^{1}H NMR (400 MHz, $CDCl_3$) δ 8.28 (d, *J* = 8.3 Hz, 1H), 7.56 – 7.51 (m, 2H), 7.46 – 7.40 (m, 4H), 7.40 – 7.35 (m, 1H), 7.31 – 7.18 (m, 6H), 7.14 (d, *J* = 6.4 Hz, 2H), 7.03 (d, *J* = 8.0 Hz, 2H), 2.80 (t, *J* = 7.3 Hz, 2H), 2.64 (td, *J* = 7.3, 0.7 Hz, 2H), 2.28 (s, 3H). ^{13}C NMR (101 MHz, $CDCl_3$) δ 144.69, 142.67, 140.41, 136.96, 134.52, 131.21, 131.13, 130.80, 129.25, 128.84, 128.44, 128.32, 127.23, 126.81, 126.23, 125.53, 124.47, 120.06, 116.47, 108.61, 94.97, 72.82, 34.86, 21.73, 21.52. HRMS (ESI) calcd for $C_{31}H_{26}NO_2S$ $[M+H]^+$ 476.1684; found: 476.1681.

***N*-(phenylethynyl)-*N*-(2-(phenylethynyl)phenyl)benzenesulfonamide (1w):** The title compound was

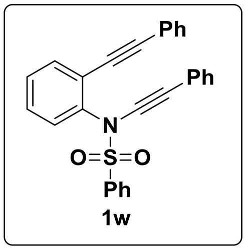

prepared according to the general procedure A via column chromatography of silica eluting hexane-ethyl acetate (98:2) to obtain as a yellow solid (The product and indole side product are similar R_F on TLC); Mp. 143-144 °C; ^{1}H NMR (400 MHz, $CDCl_3$) δ ^{1}H 8.34 (d, *J* = 8.3 Hz, 1H), 7.69 – 7.61 (m, 3H), 7.50 – 7.33 (m, 10H), 7.29 – 7.23 (m, 5H). ^{13}C NMR (101 MHz, $CDCl_3$) δ 143.37, 137.30, 137.10, 133.76, 131.39, 131.21, 130.67,

130.55, 129.12, 128.69, 128.26, 127.30, 126.76, 125.83, 124.78, 122.99, 120.15, 116.58, 108.31, 94.77, 81.31. HRMS (ESI) calcd for $C_{28}H_{20}NO_2S$ [M+H]$^+$ 434.1215; found: 434.1210.

4-chloro-*N*-(phenylethynyl)-*N*-(2-(phenylethynyl)phenyl)benzenesulfonamide (1x): The title compound was prepared according to the general procedure A via column chromatography of silica eluting hexane-ethyl acetate (97:3) to obtain as a white solid (The product and indole side product are similar R$_F$ on TLC); Mp. 139-140 °C; ^{1}H NMR (400 MHz, $CDCl_3$) δ 8.33 – 8.29 (m, 1H), 7.70 – 7.62 (m, 3H), 7.51 – 7.47 (m, 3H), 7.43 (ddt, *J* = 9.3, 3.2, 1.7 Hz, 2H), 7.39 – 7.35 (m, 3H), 7.32 – 7.27 (m, 5H), 7.25 – 7.21 (m, 2H). ^{13}C NMR (101 MHz, $CDCl_3$) δ 143.16, 140.49, 137.04, 135.56, 131.44, 131.16, 130.83, 130.42, 129.26, 129.04, 128.37, 128.28, 128.20, 127.39, 126.01, 125.06, 122.90, 120.33, 116.62, 108.79, 95.07, 81.11. HRMS (ESI) calcd for $C_{28}H_{19}NO_2SCl$ [M+H]$^+$ 468.0821; found: 468.0821.

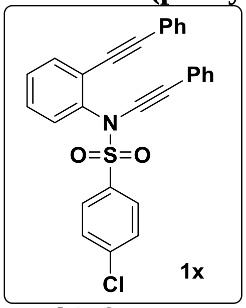

***N*-(phenylethynyl)-*N*-(2-(phenylethynyl)phenyl)ethanesulfonamide (1y):** The title compound was prepared according to the general procedure A via column chromatography of silica eluting hexane-ethyl acetate (98:2) to obtain as a white solid; Mp. 120-122 °C; ^{1}H NMR (400 MHz, $CDCl_3$) δ 8.16 – 8.10 (m, 1H), 7.84 – 7.77 (m, 1H), 7.73 – 7.67 (m, 2H), 7.49 – 7.43 (m, 3H), 7.39 (ddd, *J* = 9.0, 5.9, 3.4 Hz, 4H), 7.31 – 7.24 (m, 3H), 2.97 (q, *J* = 7.4 Hz, 2H), 0.98 (t, *J* = 7.4 Hz, 3H). ^{13}C NMR (101 MHz, $CDCl_3$) δ 143.67, 136.88, 131.34, 130.99, 130.24, 129.95, 129.12, 128.23, 127.33, 125.84, 124.58, 122.96, 120.33, 115.44, 106.97, 94.60, 81.17, 48.31, 7.38. HRMS (ESI) calcd for $C_{24}H_{20}NO_2S$ [M+H]$^+$ 386.1215; found: 386.1207.

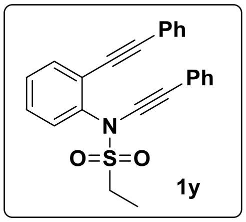

***N*-((4-methoxyphenyl)ethynyl)-*N*-(2-(phenylethynyl)phenyl)ethanesulfonamide (1y'):** The title compound was prepared according to the general procedure A via column chromatography of silica eluting hexane-ethyl acetate (95:5) to obtain as a lite yellow solid (The product and starting material are similar R$_F$ on TLC); Mp. 142-144. °C; ^{1}H NMR (400 MHz, $CDCl_3$) δ 8.17 – 8.11 (m, 1H), 7.83 – 7.78 (m, 1H), 7.74 – 7.68 (m, 2H), 7.51 – 7.45 (m, 3H), 7.45 – 7.40 (m, 2H), 7.35 (d, *J* = 8.9 Hz, 2H), 6.84 (d, *J* = 8.9 Hz, 2H), 3.81 (s, 3H), 3.01 (q, *J* = 7.4 Hz, 2H), 1.03 (t, *J* = 7.4 Hz, 3H). ^{13}C NMR (101 MHz, $CDCl_3$) δ 159.66, 143.25, 137.06, 132.94, 131.08, 130.46, 130.21, 129.12, 127.39, 125.86, 124.61, 120.47, 115.59, 115.22, 113.98, 109.99, 107.47, 94.70, 79.82, 55.30, 48.30, 7.49. HRMS (ESI) calcd for $C_{25}H_{21}NNaO_3S$ [M+Na]$^+$ 438.11344; found: 438.11369.

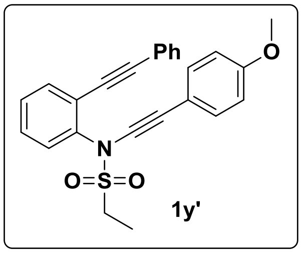

***N*-(phenylethynyl)-*N*-(2-(phenylethynyl)phenyl)methanesulfonamide (1z):** The title compound was prepared according to the general procedure A via column chromatography of silica eluting hexane-ethyl acetate (97:3) to obtain as a yellow solid (The product and indole side product are similar R$_F$ on TLC); Mp. 131-133 °C; ^{1}H NMR (400 MHz, $CDCl_3$) δ 8.16 – 8.10 (m, 1H), 7.81 (ddd, *J* = 6.0, 3.1, 0.6 Hz, 1H), 7.72 – 7.68 (m, 2H), 7.50 – 7.38 (m, 7H), 7.29 (dd, *J* = 4.0, 2.6 Hz, 3H), 2.74 (s, 3H). ^{13}C NMR (101 MHz, $CDCl_3$) δ 143.14, 136.80, 131.43, 130.86,

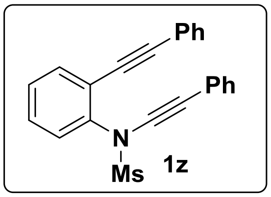

130.43, 130.24, 129.22, 128.33, 128.28, 127.48, 126.05, 124.91, 122.93, 120.41, 115.77, 107.92, 94.91, 81.06, 39.73. HRMS (ESI) calcd for $C_{23}H_{16}NO_2S$ $[M+H]^+$ 370.0902; found: 370.0901.

4-methyl-*N*-(phenylethynyl)-*N*-(3-phenylprop-2-yn-1-yl)benzenesulfonamide (1aa): The title compound

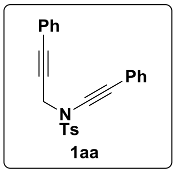

was prepared according to the procedure B via column chromatography of silica eluting hexane-ethyl acetate (95:5) to obtain as a brown gummy compound; ^{1}H NMR (400 MHz, $CDCl_3$) δ 7.93 – 7.88 (m, 2H), 7.38 (ddd, J = 8.5, 4.0, 2.4 Hz, 2H), 7.30 – 7.19 (m, 8H), 7.17 – 7.12 (m, 2H), 4.54 (s, 2H), 2.30 (s, 3H). ^{13}C NMR (101 MHz, $CDCl_3$) δ 144.81, 134.05, 131.46, 131.39, 129.46, 128.46, 128.13, 128.05, 128.00, 127.85, 122.42, 121.82, 86.38, 81.93, 81.02, 71.03, 42.75, 21.39. HRMS (ESI) calcd for $C_{24}H_{20}NO_2S$ $[M+H]^+$ 386.1215; found: 386.1210.

4-methyl-*N*-(4-phenylbut-3-yn-1-yl)-*N*-(phenylethynyl)benzenesulfonamide (1ab): The title compound was

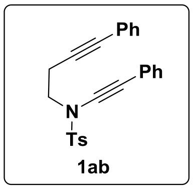

prepared according to the procedure B1 via column chromatography of silica eluting hexane-ethyl acetate (93:7) to obtain as a yellow gummy compound; ^{1}H NMR (400 MHz, $CDCl_3$) δ 7.85 (d, J = 8.3 Hz, 2H), 7.39 – 7.31 (m, 4H), 7.31 – 7.19 (m, 8H), 3.70 – 3.65 (m, 2H), 2.81 (t, J = 7.5 Hz, 2H), 2.38 (s, 3H). ^{13}C NMR (101 MHz, $CDCl_3$) δ 144.70, 134.45, 131.51, 131.48, 131.47, 131.31, 129.72, 128.19, 128.15, 128.06, 127.86, 127.81, 127.52, 123.03, 122.49, 85.16, 82.57, 81.75, 71.00, 50.33, 21.48, 19.39. HRMS (ESI) calcd for $C_{25}H_{21}NNaO_2S$ $[M+Na]^+$ 422.11852; found: 422.11876.

4-methyl-*N*-(phenylethynyl-2-^{13}C)-*N*-(2-(phenylethynyl)phenyl)benzenesulfonamide (1a'): The title

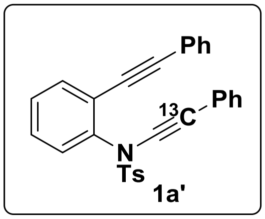

compound was prepared according to the procedure A1 via column chromatography of silica eluting hexane-ethyl acetate (98:2) to obtain as a brown solid (The product and indole side product are similar R_F on TLC); Mp. 113-115 °C; ^{1}H NMR (400 MHz, $CDCl_3$) δ 8.34 (d, J = 8.3 Hz, 1H), 7.69 – 7.63 (m, 3H), 7.49 (d, J = 3.3 Hz, 3H), 7.46 – 7.41 (m, 1H), 7.39 – 7.34 (m, 3H), 7.31 – 7.27 (m, 5H), 7.06 (d, J = 8.1 Hz, 2H), 2.29 (s, 3H). ^{13}C NMR (101 MHz, $CDCl_3$) δ 144.86, 143.46, 137.12, 134.56, 134.46, 132.90, 132.44, 131.41, 131.39, 131.23, 130.68, 129.32, 129.08, 128.30, 128.24, 127.26, 126.83, 125.75, 124.70, 123.52, 122.61, 120.10, 116.64, 109.98, 108.27, 94.68, 82.29, 21.54. HRMS (ESI) calcd for $H_{22}NO_2S_{12}C_{28}{}^{13}C$ $[M+H]^+$ 449.1405; found: 449.1407.

***N*-(3-(4-methoxyphenyl)prop-2-yn-1-yl)-4-methyl-*N*-(2-(phenylethynyl)phenyl)benzenesulfonamide (S22):**

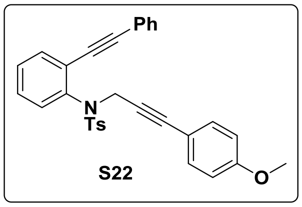

The title compound was prepared according to the general procedure C via column chromatography of silica eluting hexane-ethyl acetate (98:2) to obtain as a yellow solid; Mp. 95-97 °C; ^{1}H NMR (400 MHz, $CDCl_3$) δ 7.73 (d, J = 8.3 Hz, 2H), 7.57 – 7.53 (m, 1H), 7.42 – 7.39 (m, 1H), 7.38 – 7.34 (m, 2H), 7.34 – 7.29 (m, 5H), 7.14 (ddd, J = 5.9, 3.5, 1.4 Hz, 4H), 6.75 (d, J = 8.9 Hz, 2H), 4.80 (s, 2H), 3.76 (s, 3H), 2.26 (s, 3H). ^{13}C NMR (101 MHz, $CDCl_3$) δ 159.53, 143.31, 139.83, 137.38, 133.19, 132.94, 131.65, 131.54, 129.39, 128.72, 128.47, 128.46,

128.15, 127.73, 124.24, 122.78, 114.54, 113.71, 94.29, 85.98, 85.24, 82.17, 55.20, 41.17, 21.37. HRMS (ESI) calcd for $C_{31}H_{26}NO_3S$ [M+H]$^+$ 492.1633; found: 492.1633.

(*E*)-3-(1-iodo-2-phenyl-2-tosylvinyl)-2-phenyl-1-tosylindole (3): The title compound was prepared according to the general procedure D via column chromatography of silica eluting hexane-ethyl acetate (84:16) to obtain as a white solid (63 mg, yield = 85%); Mp. 189-191 °C; ^{1}H NMR (400 MHz, $CDCl_3$) δ 8.29 (d, *J* = 8.1 Hz, 1H), 7.77 (brs, 2H), 7.56 – 7.50 (m, 3H), 7.43 – 7.39 (m, 4H), 7.36 – 7.33 (m, 1H), 7.32 – 7.27 (m, 1H), 7.25 – 7.18 (m, 2H), 7.10 (d, *J* = 8.2 Hz, 2H), 6.97 – 6.89 (m, 5H), 6.81 (d, *J* = 7.3 Hz, 1H), 2.30 (s, 3H), 2.25 (s, 3H). ^{13}C NMR (101 MHz, $CDCl_3$) δ 151.42, 144.69, 144.42, 138.81, 137.12, 136.26, 135.74, 135.13, 131.08, 130.11, 129.99, 129.41, 129.12, 129.06, 129.02, 128.59, 128.26, 128.12, 127.92, 127.40, 126.79, 126.07, 125.42, 124.44, 119.36, 116.47, 108.31, 21.53, 21.51. HRMS (ESI) calcd for $C_{36}H_{29}NO_4S_2I$ [M+H]$^+$ 730.0583; found: 730.0588.

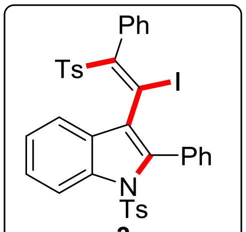

3

(*E*)-2-phenyl-1-(2-phenyl-1-tosylindol-3-yl)-2-tosylvinyl 4-methylbenzenesulfonate (4): The title compound was prepared according to the general procedure D via column chromatography of silica eluting hexane-ethyl acetate (84:16) to obtain as a white solid (9 mg, yield = 12%); Mp. 159-161 °C; ^{1}H NMR (400 MHz, $CDCl_3$) δ 8.24 – 8.18 (m, 1H), 7.78 – 7.72 (m, 1H), 7.53 (brs, 1H), 7.45 – 7.40 (m, 3H), 7.35 (t, *J* = 7.8 Hz, 2H), 7.31 – 7.26 (m, 3H), 7.20 (t, *J* = 7.6 Hz, 2H), 7.09 (d, *J* = 8.2 Hz, 4H), 7.04 (d, *J* = 8.3 Hz, 5H), 6.93 (d, *J* = 8.1 Hz, 2H), 6.80 (d, *J* = 8.4 Hz, 2H), 2.37 (s, 3H), 2.35 (s, 3H), 2.26 (s, 3H). ^{13}C NMR (101 MHz, $CDCl_3$) δ 145.58, 145.01, 144.79, 144.57, 140.94, 140.65, 136.63, 135.82, 135.35, 133.15, 130.82, 130.08, 130.02, 129.76, 129.45, 129.34, 129.03, 129.00, 128.88, 128.74, 127.88, 127.39, 127.24, 126.70, 125.30, 124.43, 120.98, 116.21, 115.80, 21.58, 21.58, 21.54. HRMS (ESI) calcd for $C_{43}H_{35}NO_7S_3Na$ [M+Na]$^+$ 796.1473; found: 796.1481.

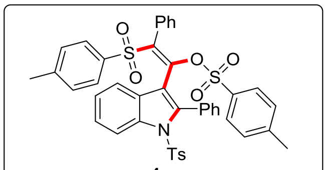

4

(*E*)-3-(1-iodo-2-phenyl-2-tosylvinyl)-5-methyl-2-phenyl-1-tosylindole (5): The title compound was prepared according to the general procedure D via column chromatography of silica eluting hexane-ethyl acetate (83:17) to obtain as a white solid (59 mg, yield = 80%); Mp. 188-190 °C; ^{1}H NMR (400 MHz, $CDCl_3$) δ 8.15 (d, *J* = 8.5 Hz, 1H), 7.75 (brs, 2H), 7.57 – 7.50 (m, 3H), 7.41 (d, *J* = 8.4 Hz, 2H), 7.34 – 7.29 (m, 1H), 7.26 (dd, *J* = 8.2, 0.8 Hz, 1H), 7.24 – 7.19 (m, 2H), 7.11 (d, *J* = 8.0 Hz, 2H), 7.05 – 7.03 (m, 1H), 6.99 (d, *J* = 6.9 Hz, 1H), 6.95 – 6.88 (m, 4H), 6.84 (d, *J* = 7.8 Hz, 1H), 2.45 – 2.42 (m, 3H), 2.31 (s, 3H), 2.27 (d, *J* = 5.8 Hz, 3H). ^{13}C NMR (101 MHz, $CDCl_3$) δ 151.48, 144.62, 144.35, 138.97, 136.40, 135.91, 135.34, 135.12, 134.09, 131.24, 130.12, 130.05, 129.40, 129.16, 129.02, 128.94, 128.78, 128.64, 128.31, 128.19, 127.96, 127.40, 126.87, 126.85, 125.95, 119.22, 116.20, 108.93, 21.55, 21.53, 21.43. HRMS (ESI) calcd for $C_{37}H_{31}NO_4S_2I$ [M+H]$^+$ 744.0739; found: 744.0728.

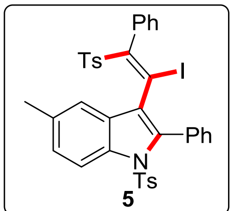

5

(*E*)-5-ethyl-3-(1-iodo-2-phenyl-2-tosylvinyl)-2-phenyl-1-tosylindole (6): The title compound was prepared according to the general procedure D via column chromatography of silica eluting hexane-ethyl acetate (84:16) to obtain as a white solid (58 mg, yield = 75%); Mp. 186-188 °C; ^{1}H

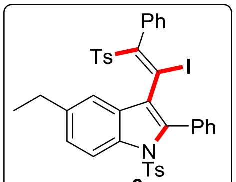

6

NMR (400 MHz, $CDCl_3$) δ 8.18 (d, *J* = 8.6 Hz, 1H), 7.73 (brs, 2H), 7.55 – 7.50 (m, 3H), 7.41 (d, *J* = 8.4 Hz, 2H), 7.34 – 7.29 (m, 1H), 7.28 – 7.21 (m, 3H), 7.10 (d, *J* = 8.0 Hz, 3H), 6.95 (s, 4H), 6.91 (d, *J* = 7.8 Hz, 1H), 6.86 (d, *J* = 7.5 Hz, 1H), 2.81 – 2.69 (m, 2H), 2.31 (s, 3H), 2.26 (s, 3H), 1.33 (t, *J* = 7.6 Hz, 3H). ^{13}C NMR (101 MHz, $CDCl_3$) δ 151.35, 144.59, 144.36, 140.42, 138.93, 136.37, 135.91, 135.44, 135.26, 131.16, 130.14, 129.40, 129.14, 129.02, 128.98, 128.63, 128.32, 128.10, 127.89, 127.38, 126.85, 125.89, 125.75, 117.96, 116.22, 108.91, 28.75, 21.56, 21.53, 15.80. HRMS (ESI) calcd for $C_{38}H_{33}NO_4S_2I$ $[M+H]^+$ 758.0896; found: 758.0893.

(*E*)-5-butyl-3-(1-iodo-2-phenyl-2-tosylvinyl)-2-phenyl-1-tosylindole (7): The title compound was prepared

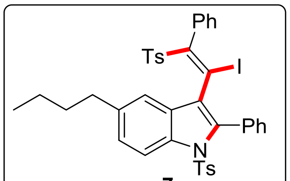

7

according to the general procedure D via column chromatography of silica eluting hexane-ethyl acetate (86:14) to obtain as a yellow solid (50 mg, yield = 64%); ^{1}H NMR (400 MHz, $CDCl_3$) δ 8.17 (d, *J* = 8.5 Hz, 1H), 7.74 (d, *J* = 10.1 Hz, 2H), 7.53 (brs, 3H), 7.41 (d, *J* = 8.4 Hz, 2H), 7.35 – 7.27 (m, 2H), 7.23 (dd, *J* = 7.5, 6.0 Hz, 2H), 7.09 (dd, *J* = 17.5, 8.1 Hz, 4H), 6.95 (s, 4H), 6.90 (d, *J* = 8.2 Hz, 1H), 6.86 (d, *J* = 7.5 Hz, 1H), 2.76 – 2.65 (m, 2H), 2.32 (s, 3H), 2.27 (s, 3H), 1.72 – 1.65 (m, 2H), 1.45 (dt, *J* = 14.8, 7.4 Hz, 2H), 0.99 (t, *J* = 7.3 Hz, 3H). ^{13}C NMR (101 MHz, $CDCl_3$) δ 151.24, 144.59, 144.36, 139.18, 138.92, 136.34, 135.91, 135.45, 135.26, 131.15, 130.17, 129.40, 129.15, 129.03, 129.00, 128.66, 128.34, 128.11, 127.87, 127.40, 126.87, 126.22, 125.87, 118.55, 116.18, 108.94, 35.68, 33.99, 22.55, 21.59, 21.55, 14.07. HRMS (ESI) calcd for $C_{40}H_{37}NO_4$ S_2I $[M+H]^+$ 786.1209; found: 786.1206.

(*E*)-5-chloro-3-(1-iodo-2-phenyl-2-tosylvinyl)-2-phenyl-1-tosylindole (8): The title compound was prepared

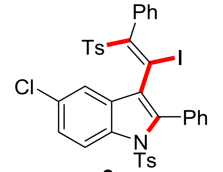

8

according to the general procedure D via column chromatography of silica eluting hexane-ethyl acetate (82:18) to obtain as a white solid (57 mg, yield = 75%); Mp. 176-178 °C; ^{1}H NMR (400 MHz, $CDCl_3$) δ 8.22 (d, *J* = 8.9 Hz, 1H), 7.77 (brs, 2H), 7.55 (brs, 3H), 7.40 (d, *J* = 8.4 Hz, 2H), 7.36 (dd, *J* = 8.9, 2.1 Hz, 1H), 7.34 – 7.30 (m, 1H), 7.27 (s, 1H), 7.23 (s, 1H), 7.18 (d, *J* = 1.7 Hz, 1H), 7.14 (d, *J* = 8.0 Hz, 2H), 6.99 – 6.95 (m, 2H), 6.94 – 6.87 (m, 4H), 2.33 (s, 3H), 2.29 (s, 3H). ^{13}C NMR (101 MHz, $CDCl_3$) δ 152.38, 145.10, 144.72, 138.74, 137.86, 135.71, 135.37, 134.90, 130.56, 130.16, 130.08, 129.86, 129.57, 129.42, 129.28, 129.13, 128.95, 128.56, 128.38, 128.03, 127.50, 126.90, 125.52, 125.05, 118.92, 117.57, 107.32, 21.59, 21.57. HRMS (ESI) calcd for $C_{36}H_{28}NO_4S_2ClI$ $[M+H]^+$ 764.0193; found: 764.0190.

(*E*)-5-bromo-3-(1-iodo-2-phenyl-2-tosylvinyl)-2-phenyl-1-tosylindole (9): The title compound was prepared

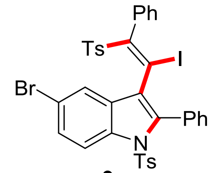

9

according to the general procedure D via column chromatography of silica eluting hexane-ethyl acetate (82:18) to obtain as a white solid (56 mg, yield = 70%); Mp. 192-193 °C; ^{1}H NMR (400 MHz, $CDCl_3$) δ 8.25 (dd, *J* = 9.1, 4.4 Hz, 1H), 7.75 (brs, 2H), 7.58 – 7.51 (m, 3H), 7.39 (d, *J* = 8.4 Hz, 2H), 7.34 – 7.29 (m, 1H), 7.25 (q, *J* = 4.7 Hz, 2H), 7.16 – 7.10 (m, 3H), 6.96 (d, *J* = 8.2 Hz, 3H), 6.92 (d, *J* = 8.5 Hz, 3H), 6.83 (d, *J* = 7.3 Hz, 1H), 2.32 (s, 3H), 2.28 (s, 3H). ^{13}C NMR (101 MHz, $CDCl_3$) δ 161.35, 158.95, 152.04, 144.98, 144.65, 138.72, 138.13, 135.72, 134.88, 133.35, 131.47, 130.78, 130.37, 130.07, 129.86, 129.52, 129.35, 129.24, 129.13, 129.10, 128.57, 128.34, 128.03, 127.50,

126.86, 125.79, 125.75, 117.88, 117.79, 113.39, 113.14, 107.43, 105.18, 104.94, 21.56, 21.54. HRMS (ESI) calcd for $C_{36}H_{28}NO_4S_2BrI$ $[M+H]^+$ 807.9688; found: 807.9681.

(*E*)-3-(1-iodo-2-phenyl-2-tosylvinyl)-2-(p-tolyl)-1-tosylindole (10): The title compound was prepared

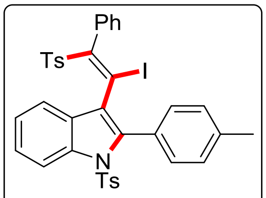

10

according to the general procedure D via column chromatography of silica eluting hexane-ethyl acetate (84:16) to obtain as a white solid (65 mg, yield = 88%); ^1H NMR (400 MHz, $CDCl_3$) δ 8.15 (d, *J* = 8.5 Hz, 1H), 7.75 (brs, 2H), 7.57 – 7.50 (m, 3H), 7.41 (d, *J* = 8.4 Hz, 2H), 7.34 – 7.29 (m, 1H), 7.26 (dd, *J* = 8.2, 0.8 Hz, 1H), 7.24 – 7.19 (m, 2H), 7.11 (d, *J* = 8.0 Hz, 2H), 7.05 – 7.03 (m, 1H), 6.99 (d, *J* = 6.9 Hz, 1H), 6.95 – 6.88 (m, 4H), 6.84 (d, *J* = 7.8 Hz, 1H), 2.45 – 2.42 (m, 3H), 2.31 (s, 3H), 2.27 (d, *J* = 5.8 Hz, 3H). ^{13}C NMR (101 MHz, $CDCl_3$) δ 151.48, 144.62, 144.35, 138.97, 136.40, 135.91, 135.34, 135.12, 134.09, 131.24, 130.12, 130.05, 129.40, 129.16, 129.02, 128.94, 128.78, 128.64, 128.31, 128.19, 127.96, 127.40, 126.87, 126.85, 125.95, 119.22, 116.20, 108.93, 21.55, 21.53, 21.43. HRMS (ESI) calcd for $C_{37}H_{31}NO_4S_2I$ $[M+H]^+$ 744.0739; found: 744.0741.

(*E*)-3-(1-iodo-2-phenyl-2-tosylvinyl)-2-(4-methoxyphenyl)-1-tosylindole (11): The title compound was

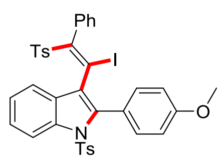

11

prepared according to the general procedure D via column chromatography of silica eluting hexane-ethyl acetate (83:17) to obtain as a white solid (55 mg, yield = 72%); ^1H NMR (400 MHz, $CDCl_3$) δ 8.28 (d, *J* = 8.3 Hz, 1H), 7.67 (brs, 2H), 7.42 – 7.38 (m, 3H), 7.36 – 7.29 (m, 3H), 7.29 – 7.20 (m, 3H), 7.10 (d, *J* = 8.1 Hz, 2H), 7.06 (d, *J* = 8.9 Hz, 2H), 6.96 (s, 4H), 6.91 (dd, *J* = 11.7, 7.7 Hz, 2H), 3.95 (s, 3H), 2.32 (s, 3H). ^{13}C NMR (101 MHz, $CDCl_3$) δ 160.29, 151.44, 144.64, 144.42, 138.95, 137.09, 136.58, 135.86, 135.21, 130.20, 130.12, 129.41, 129.16, 129.02, 128.65, 128.32, 128.16, 127.96, 126.84, 125.58, 125.20, 124.39, 123.32, 119.22, 116.57, 112.93, 108.94, 55.30, 21.58, 21.54. HRMS (ESI) calcd for $C_{37}H_{31}NO_5S_2I$ $[M+H]^+$ 760.0688; found: 760.0694.

(*E*)-2-(3,4-dimethoxyphenyl)-3-(1-iodo-2-phenyl-2-tosylvinyl)-1-tosylindole (12): The title compound was

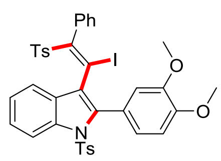

12

prepared according to the general procedure D via column chromatography of silica eluting hexane-ethyl acetate (80:20) to obtain as a white solid (40 mg, yield = 51%); ^1H NMR (400 MHz, $CDCl_3$) δ 8.31 (d, *J* = 8.3 Hz, 1H), 7.47 – 7.37 (m, 4H), 7.37 – 7.28 (m, 4H), 7.24 – 7.16 (m, 2H), 7.11 (d, *J* = 8.1 Hz, 2H), 7.06 – 6.88 (m, 7H), 6.80 (d, *J* = 27.8 Hz, 1H), 4.02 (s, 3H), 3.93 (s, 3H), 2.32 (s, 3H), 2.27 (s, 3H). ^{13}C NMR (101 MHz, $CDCl_3$) δ 151.48, 149.67, 144.63, 144.53, 138.85, 137.12, 136.47, 135.78, 135.50, 130.30, 130.05, 129.37, 129.21, 129.06, 128.69, 128.37, 127.95, 126.96, 125.25, 124.28, 123.17, 119.16, 116.53, 109.97, 56.11, 55.86, 21.57, 21.52. HRMS (ESI) calcd for $C_{38}H_{33}NO_6S_2I$ $[M+H]^+$ 790.0794; found: 790.0789.

(*E*)-2-butyl-3-(1-iodo-2-phenyl-2-tosylvinyl)-1-tosylindole (13): The title compound was prepared according

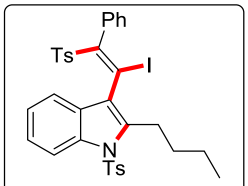

13

to the general procedure D via column chromatography of silica eluting hexane-ethyl acetate (85:15) to obtain as a white solid (52 mg, yield = 74%); Mp. 127-129 °C; ^1H NMR (400 MHz, $CDCl_3$) δ 8.06 (d, *J* = 8.4 Hz, 1H), 7.80 (d, *J* = 8.4 Hz, 2H), 7.44 (dd, *J* = 15.8,

8.5 Hz, 2H), 7.36 (dd, J = 14.8, 6.2 Hz, 2H), 7.26 (s, 1H), 7.25 – 7.20 (m, 2H), 7.15 – 7.04 (m, 3H), 6.86 (d, J = 8.3 Hz, 2H), 6.73 (d, J = 8.0 Hz, 2H), 3.37 – 3.28 (m, 1H), 3.19 (ddd, J = 14.4, 11.1, 5.5 Hz, 1H), 2.29 (s, 3H), 2.22 (s, 3H), 1.98 – 1.87 (m, 1H), 1.79 (dt, J = 13.1, 7.2 Hz, 1H), 1.61 – 1.55 (m, 2H), 1.02 (t, J = 7.4 Hz, 3H). ^{13}C NMR (101 MHz, $CDCl_3$) δ 153.07, 144.95, 144.18, 139.67, 139.10, 136.27, 136.10, 135.84, 130.10, 129.84, 129.39, 128.89, 128.61, 128.31, 127.25, 126.81, 124.31, 123.48, 122.34, 119.10, 114.79, 108.57, 30.35, 28.26, 23.27, 21.52, 21.45, 13.85. HRMS (ESI) calcd for $C_{34}H_{33}NO_4S_2I$ [M+H]$^+$ 710.0896; found: 710.0900.

(*E*)-2-cyclopropyl-3-(1-iodo-2-phenyl-2-tosylvinyl)-1-tosylindole (14): T The title compound was prepared

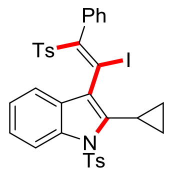

14

according to the general procedure D via column chromatography of silica eluting hexane-ethyl acetate (83:17) to obtain as a white solid (56 mg, yield = 82%); Mp. 152-153 °C; ^{1}H NMR (400 MHz, $CDCl_3$) δ 8.06 (d, J = 8.4 Hz, 1H), 7.80 (d, J = 8.4 Hz, 2H), 7.44 (dd, J = 15.8, 8.5 Hz, 2H), 7.36 (dd, J = 14.8, 6.2 Hz, 2H), 7.26 (s, 1H), 7.25 – 7.20 (m, 2H), 7.15 – 7.04 (m, 3H), 6.86 (d, J = 8.3 Hz, 2H), 6.73 (d, J = 8.0 Hz, 2H), 3.37 – 3.28 (m, 1H), 3.19 (ddd, J = 14.4, 11.1, 5.5 Hz, 1H), 2.29 (s, 3H), 2.22 (s, 3H), 1.98 – 1.87 (m, 1H), 1.79 (dt, J = 13.1, 7.2 Hz, 1H), 1.61 – 1.55 (m, 2H), 1.02 (t, J = 7.4 Hz, 3H). ^{13}C NMR (101 MHz, $CDCl_3$) δ 152.05, 144.84, 144.07, 139.05, 137.91, 136.71, 136.18, 135.94, 130.44, 129.74, 129.34, 128.98, 128.06, 126.84, 126.73, 124.77, 123.10, 121.87, 119.38, 114.36, 108.72, 21.56, 21.46, 10.35, 8.80, 7.24. HRMS (ESI) calcd for $C_{33}H_{29}NO_4S_2I$ [M+H]$^+$ 694.0583; found: 694.0580.

((*E*)-2-(3-(1-iodo-2-phenyl-2-tosylvinyl)-1-tosylindol-2-yl)ethan-1-ol (15): The title compound was prepared

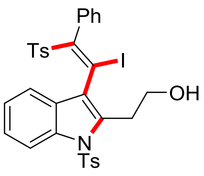

15

according to the general procedure D via column chromatography of silica eluting hexane-ethyl acetate (75:25) to obtain as a gummy compound (20 mg, yield = 29%); ^{1}H NMR (400 MHz, $CDCl_3$) δ 8.08 (d, J = 8.3 Hz, 1H), 7.77 (d, J = 8.4 Hz, 2H), 7.43 (dd, J = 14.6, 6.2 Hz, 3H), 7.29 (d, J = 8.5 Hz, 2H), 7.24 (d, J = 8.3 Hz, 2H), 7.16 (dt, J = 12.6, 7.0 Hz, 3H), 6.97 (d, J = 7.1 Hz, 1H), 6.90 (d, J = 8.3 Hz, 2H), 6.79 (d, J = 8.1 Hz, 2H), 3.94 – 3.76 (m, 2H), 3.49 – 3.30 (m, 2H), 2.29 (s, 3H), 2.24 (s, 3H). ^{13}C NMR (101 MHz, $CDCl_3$) δ 153.01, 145.06, 144.37, 138.91, 138.51, 135.99, 135.95, 135.83, 129.90, 129.42, 128.99, 128.40, 126.69, 124.57, 123.71, 119.10, 114.92, 108.20, 62.37, 31.46, 24.74, 21.53, 21.50. HRMS (ESI) calcd for $C_{32}H_{29}NO_5S_2I$ [M+H]$^+$ 698.0532; found: 698.0538.

(*E*)-3-(1-iodo-2-(p-tolyl)-2-tosylvinyl)-2-phenyl-1-tosylindole (16): The title compound was prepared

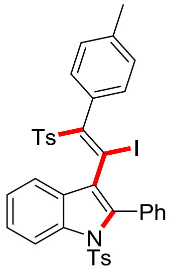

16

according to the general procedure D via column chromatography of silica eluting hexane-ethyl acetate (84:16) to obtain as a white solid (61 mg, yield = 83%); Mp. 196-198 °C; ^{1}H NMR (400 MHz, $CDCl_3$) δ 8.28 (d, J = 8.3 Hz, 1H), 7.82 (brs, 2H), 7.56 – 7.51 (m, 3H), 7.43 – 7.40 (m, 2H), 7.38 (dd, J = 5.0, 1.8 Hz, 1H), 7.36 – 7.30 (m, 2H), 7.10 (d, J = 8.1 Hz, 2H), 7.05 (d, J = 6.9 Hz, 2H), 6.94 (s, 4H), 6.80 (d, J = 6.6 Hz, 1H), 6.72 (d, J = 7.3 Hz, 1H), 2.32 (s, 3H), 2.31 (s, 3H), 2.26 (s, 3H). ^{13}C NMR (101 MHz, $CDCl_3$) δ 151.50, 144.68, 144.32, 139.24,

137.11, 136.28, 135.93, 135.88, 135.15, 131.11, 130.05, 129.93, 129.42, 129.05, 129.00, 128.61, 128.11, 127.40, 126.83, 126.14, 125.38, 124.39, 119.39, 116.46, 108.52, 21.56, 21.52, 21.41. HRMS (ESI) calcd for $C_{37}H_{31}NO_4S_2I$ $[M+H]^+$ 744.0739; found: 744.0741.

((*E*)-3-(2-(4-ethylphenyl)-1-iodo-2-tosylvinyl)-2-phenyl-1-tosylindole (17): The title compound was prepared

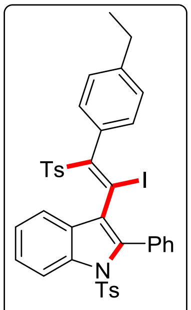

17

according to the general procedure D via column chromatography of silica eluting hexane-ethyl acetate (83:17) to obtain as a white solid (57 mg, yield = 74%); Mp. 133-134 °C; ^{1}H NMR (400 MHz, $CDCl_3$) δ 8.28 (d, *J* = 8.3 Hz, 1H), 7.75 (brs, 2H), 7.57 – 7.50 (m, 3H), 7.42 (d, *J* = 8.4 Hz, 2H), 7.40 – 7.37 (m, 1H), 7.36 – 7.34 (m, 1H), 7.32 (dd, *J* = 7.8, 0.9 Hz, 1H), 7.13 – 7.03 (m, 4H), 6.97 – 6.91 (m, 4H), 6.82 (d, *J* = 7.7 Hz, 1H), 6.74 (d, *J* = 7.6 Hz, 1H), 2.62 (q, *J* = 7.6 Hz, 2H), 2.31 (s, 3H), 2.26 (s, 3H), 1.20 (t, *J* = 7.6 Hz, 3H). ^{13}C NMR (101 MHz, $CDCl_3$) δ 151.59, 145.50, 144.69, 144.29, 137.11, 136.31, 136.11, 135.92, 135.14, 131.13, 129.99, 129.89, 129.43, 129.06, 128.97, 128.62, 128.12, 127.77, 127.41, 126.84, 126.16, 125.39, 124.41, 119.41, 116.47, 108.37, 28.62, 21.56, 21.54, 15.24. HRMS (ESI) calcd for $C_{38}H_{33}NO_4S_2I$ $[M+H]^+$ 758.0896; found: 758.0894.

(*E*)-3-(1-iodo-2-(3-methoxyphenyl)-2-tosylvinyl)-2-phenyl-1-tosylindole (18): The title compound was

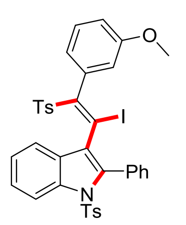

18

prepared according to the general procedure D via column chromatography of silica eluting hexane-ethyl acetate (84:16) to obtain as a white solid (48 mg, yield = 63%); Mp. 153-155 °C; ^{1}H NMR (400 MHz, $CDCl_3$) δ 8.29 (d, *J* = 8.6 Hz, 1H), 7.75 (brs, 2H), 7.54 (d, *J* = 2.3 Hz, 3H), 7.41 (d, *J* = 8.1 Hz, 4H), 7.37 – 7.31 (m, 1H), 7.12 (dd, *J* = 16.2, 8.1 Hz, 3H), 6.97 (s, 4H), 6.83 (dd, *J* = 8.2, 2.2 Hz, 1H), 6.51 – 6.30 (m, 2H), 3.69 (d, *J* = 6.0 Hz, 3H), 2.31 (s, 3H), 2.26 (s, 3H). ^{13}C NMR (101 MHz, $CDCl_3$) δ 159.09, 158.91, 151.30, 144.71, 144.42, 139.89, 135.84, 135.14, 131.13, 129.43, 129.36, 129.06, 129.02, 128.65, 127.40, 126.82, 126.07, 125.42, 124.44, 122.49, 122.43, 119.39, 116.50, 115.35, 115.27, 115.07, 55.16, 21.53. HRMS (ESI) calcd for $C_{37}H_{31}NO_5S_2I$ $[M+H]^+$ 760.0688; found: 760.0690.

(*E*)-3-(1-iodo-2-phenyl-2-tosylvinyl)-2-(4-methoxyphenyl)-1-tosylindole (19): The title compound was

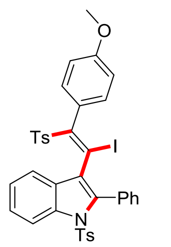

19

prepared according to the general procedure D via column chromatography of silica eluting hexane-ethyl acetate (84:16) to obtain as a lite yellow solid (52 mg, yield = 68%); Mp. 175-178 °C; ^{1}H NMR (400 MHz, $CDCl_3$) δ 8.28 (d, *J* = 8.3 Hz, 1H), 7.74 (brs, 2H), 7.55 – 7.51 (m, 3H), 7.43 – 7.39 (m, 3H), 7.38 – 7.32 (m, 2H), 7.10 (d, *J* = 8.1 Hz, 2H), 6.99 – 6.93 (m, 4H), 6.84 (d, *J* = 7.7 Hz, 1H), 6.74 (d, *J* = 7.6 Hz, 3H), 3.78 (s, 3H), 2.31 (s, 3H), 2.26 (s, 3H). ^{13}C NMR (101 MHz, $CDCl_3$) δ 160.06, 151.14, 144.68, 144.30, 137.12, 136.22, 135.92, 135.15, 131.49, 131.13, 131.10, 129.42, 129.03, 128.57, 128.16, 127.41, 126.82, 126.19, 125.41, 124.42, 119.39, 116.48, 113.55, 108.99, 55.19, 21.57, 21.54. HRMS (ESI) calcd for $C_{37}H_{31}NO_5S_2I$ $[M+H]^+$ 760.0688; found: 760.0687.

(*E*)-3-(1-iodo-2-tosyl-2-(3,4,5-trimethoxyphenyl)vinyl)-2-phenyl-1-tosylindole (20): The title compound was

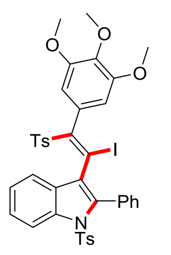

20

prepared according to the general procedure D via column chromatography of silica eluting hexane-ethyl acetate (70:30) to obtain as a yellow solid (39 mg, yield = 47%); Mp. 122-124 °C; ^{1}H NMR (400 MHz, CDCl$_3$) ^{1}H NMR (400 MHz, CDCl$_3$) δ 8.30 (d, *J* = 7.9 Hz, 1H), 7.73 (brs, 2H), 7.53 (d, *J* = 2.4 Hz, 3H), 7.49 – 7.46 (m, 1H), 7.46 – 7.43 (m, 1H), 7.41 (d, *J* = 7.1 Hz, 2H), 7.37 (dd, *J* = 7.3, 1.0 Hz, 1H), 7.11 (d, *J* = 8.0 Hz, 2H), 7.06 – 7.00 (m, 4H), 6.00 (s, 1H), 5.89 (s, 1H), 3.82 (s, 3H), 3.66 (d, *J* = 7.7 Hz, 6H), 2.32 (s, 3H), 2.28 (s, 3H).

^{13}C NMR (101 MHz, CDCl$_3$) δ ^{13}C NMR (101 MHz, CDCl$_3$) δ 151.16, 144.75, 144.41, 137.15, 136.20, 135.96, 135.21, 133.78, 131.20, 129.46, 129.04, 128.98, 128.76, 128.07, 127.28, 126.93, 126.79, 125.52, 124.47, 119.47, 116.50, 108.19, 107.45, 105.33, 61.01, 56.06, 21.56, 21.50. HRMS (ESI) calcd for $C_{39}H_{34}N\ O_7NaS_2I$ [M+Na]$^+$ 842.0719; found: 842.0718.

(*E*)-3-(1-iodo-2-(3-nitrophenyl)-2-tosylvinyl)-2-phenyl-1-tosylindole (21): The title compound was prepared

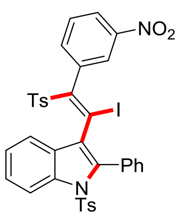

21

according to the general procedure D via column chromatography of silica eluting hexane-ethyl acetate (80:20) to obtain as a lite yellow solid (49 mg, yield = 59%); Mp. 173-174 °C; ^{1}H NMR (400 MHz, CDCl$_3$) δ 8.31 (d, *J* = 8.3 Hz, 1H), 8.18 (d, *J* = 8.0 Hz, 1H), 7.83 (brs, 2H), 7.69 (d, *J* = 7.5 Hz, 2H), 7.57 (d, *J* = 10.1 Hz, 4H), 7.45 (dd, *J* = 17.2, 10.3 Hz, 4H), 7.36 (d, *J* = 15.2 Hz, 2H), 7.21 (d, *J* = 7.0 Hz, 1H), 7.12 (d, *J* = 7.9 Hz, 2H), 7.04 – 6.90 (m, 4H), 2.32 (s, 3H), 2.27 (s, 3H). ^{13}C NMR (101 MHz, CDCl$_3$) δ 147.52, 145.36, 144.89, 140.33, 136.99, 136.65, 136.32, 135.10, 130.80, 129.62, 129.47, 129.20, 128.50, 127.87, 127.61, 127.48, 126.84, 125.59, 125.39, 125.21, 124.50, 124.03, 119.32, 116.62, 116.42, 109.85, 21.55, 21.54. HRMS (ESI) calcd for $C_{36}H_{28}N_2O_6S_2I$ [M+H]$^+$ 75.0433; found: 775.0437.

methyl (*E*)-4-(2-iodo-2-(2-phenyl-1-tosylindol-3-yl)-1-tosylvinyl)benzoate (22): The title compound was

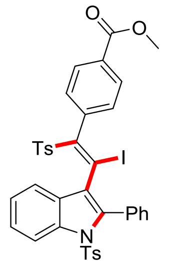

22

prepared according to the general procedure D via column chromatography of silica eluting hexane-ethyl acetate (78:22) to obtain as a white solid (50 mg, yield = 64%); Mp. 185-186 °C; ^{1}H NMR (400 MHz, CDCl$_3$) δ 8.30 (d, *J* = 8.3 Hz, 1H), 7.91 (t, *J* = 7.3 Hz, 2H), 7.74 (brs, 2H), 7.55 (s, 3H), 7.45 – 7.35 (m, 5H), 7.11 (d, *J* = 8.2 Hz, 2H), 6.94 (dt, *J* = 14.3, 7.4 Hz, 6H), 3.91 (s, 3H), 2.32 (s, 3H), 2.27 (s, 3H). ^{13}C NMR (101 MHz, CDCl$_3$) δ 166.35, 150.73, 144.79, 144.77, 143.18, 137.11, 136.39, 135.46, 135.13, 130.97, 130.62, 130.29, 129.56, 129.44, 129.23, 128.58, 127.95, 127.47, 126.82, 125.63, 125.51, 124.47, 119.28, 116.52, 108.51, 52.29, 21.59, 21.54. HRMS (ESI) calcd for $C_{38}H_{31}NO_6S_2I$ [M+H]$^+$ 788.0637; found: 788.0642.

(*E*)-1-(4-(2-iodo-2-(2-phenyl-1-tosylindol-3-yl)-1-tosylvinyl)phenyl)ethan-1-one (23): The title compound

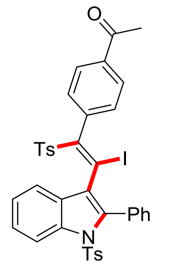

23

was prepared according to the general procedure D via column chromatography of silica eluting hexane-ethyl acetate (75:25) to obtain as a lite yellow solid (32 mg, yield = 42%); Mp. 185-186 °C; ^{1}H NMR (400 MHz, CDCl$_3$) δ 8.30 (d, *J* = 8.3 Hz, 1H), 7.85 (dd, *J* = 15.9, 9.4 Hz,

3H), 7.71 (brs, 1H), 7.55 (s, 3H), 7.45 – 7.39 (m, 3H), 7.38 – 7.32 (m, 2H), 7.11 (d, *J* = 8.1 Hz, 2H), 7.01 (d, *J* = 7.7 Hz, 1H), 6.96 (q, *J* = 8.6 Hz, 5H), 2.59 (s, 3H), 2.32 (s, 3H), 2.27 (s, 3H). ^{13}C NMR (101 MHz, $CDCl_3$) δ 197.35, 150.74, 144.83, 144.79, 143.31, 137.14, 137.07, 136.42, 135.46, 135.11, 130.94, 130.47, 129.44, 129.24, 129.21, 128.55, 128.36, 127.85, 127.46, 126.82, 125.53, 125.50, 124.44, 119.27, 116.49, 108.60, 26.65, 21.58, 21.53. HRMS (ESI) calcd for $C_{38}H_{31}NO_5S_2I$ $[M+H]^+$ 772.0688; found: 772.0693

(*E*/*Z*)-3-(2-(2,4-dichlorophenyl)-1-iodo-2-tosylvinyl)-2-phenyl-1-tosylindole (24): The title compound was

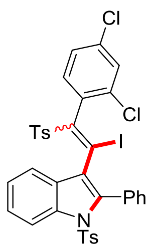

24

prepared according to the general procedure D via column chromatography of silica eluting hexane-ethyl acetate (80:22) to obtain mixture two isomers (*E*/*Z*= 52:48) as a white solid (43 mg, yield = 43%); ^{1}H NMR (400 MHz, $CDCl_3$) δ 8.30 (d, *J* = 8.4 Hz, 1H), 8.26 (d, *J* = 8.4 Hz, 1H), 7.74 (s, 3H), 7.56 – 7.50 (m, 4H), 7.48 (dd, *J* = 3.7, 2.0 Hz, 2H), 7.45 – 7.42 (m, 4H), 7.41 – 7.38 (m, 2H), 7.33 – 7.26 (m, 6H), 7.24 – 7.18 (m, 2H), 7.16 – 7.09 (m, 5H), 7.06 (dd, *J* = 8.0, 1.4 Hz, 3H), 6.95 (dt, *J* = 15.1, 6.0 Hz, 6H), 2.34 (s, 3H), 2.30 (s, 4H), 2.27 (s, 5H). ^{13}C NMR (101 MHz, $CDCl_3$) δ 149.01, 148.33, 144.96, 144.93,144.86, 144.82, 137.09, 136.84, 136.75, 136.24, 135.92, 135.65, 135.58, 135.26, 135.17, 134.76, 132.64, 132.58, 130.97, 130.82, 129.61, 129.49, 129.46, 129.41, 129.32, 129.16, 129.03, 128.95, 128.06, 127.59, 127.51, 127.39, 127.16, 126.96, 126.91, 125.43, 125.34, 125.25, 124.49, 124.30, 119.71, 119.22, 116.47, 116.24, 111.33, 109.97, 21.61, 21.55. HRMS (ESI) calcd for $C_{36}H_{26}Cl_2INO_4S_2$ $[M+H]^+$ 797.9250; found: 797.9252.

(*E*/*Z*)-3-(1-iodo-2-(naphthalen-2-yl)-2-tosylvinyl)-2-phenyl-1-tosylindole (25): The title compound was

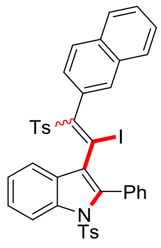

25

prepared according to the general procedure D via column chromatography of silica eluting hexane-ethyl acetate (78:22) to obtain mixture two isomers (*E*/*Z*= 81:19) as a white solid (43 mg, yield = 55%); ^{1}H NMR (400 MHz, $CDCl_3$) δ ^{1}H NMR (400 MHz, $CDCl_3$) δ 8.37 (d, J = 8.4 Hz, 0.24 H), 8.31 (d, *J* = 8.3 Hz, 1H), 7.82 – 7.74 (m, 2H), 7.69 (dd, *J* = 8.8, 2.4 Hz, 2H), 7.56 (d, *J* = 12.8 Hz, 4H), 7.52 – 7.47 (m, 3H), 7.43 (d, *J* = 8.5 Hz, 3H), 7.39 (dd, *J* = 7.9, 3.0 Hz, 2H), 7.11 (d, *J* = 8.3 Hz, 2H), 7.08 (d, *J* = 8.3 Hz, 0.534H), 6.97 – 6.93 (m, 2H), 6.92 (s, 3H), 6.84 (d, *J* = 7.3 Hz, 0.29 H), 6.56 (d, *J* = 8.1 Hz, 0.29 H), 6.50 (d, *J* = 8.3 Hz, 1H), 2.28 (s, 4H), 2.27 (s, 3H), 2.16 (s, 0.27H), 2.14 (s, 0.36H). ^{13}C NMR (101 MHz, $CDCl_3$) δ 144.73, 144.47, 137.19, 136.23, 135.19, 133.12, 130.01, 129.87, 129.45, 129.32, 129.14, 129.07, 128.72, 128.66, 128.61, 128.49, 128.22, 128.08, 127.90, 127.73, 127.65, 127.48, 127.34, 127.03, 126.86, 126.36, 125.47, 124.50, 124.41, 119.46, 116.54, 21.55, 21.33. HRMS (ESI) calcd for $C_{40}H_{31}NO_4S_2I$ $[M+H]^+$ 780.0739; found: 780.0734.

(*E*/*Z*)-3-(2-(6-bromobenzo[d][1,3]dioxol-5-yl)-1-iodo-2-tosylvinyl)-2-phenyl-1-tosylindole (26): The title

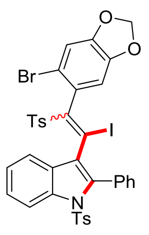

26

compound was prepared according to the general procedure D via column chromatography of silica eluting hexane-ethyl acetate (80:20) to obtain mixture two isomers (*E*/*Z*= 76:24) as a yellow solid (30 mg, yield = 35%); Mp. 141-192 °C; ^{1}H NMR (400 MHz, $CDCl_3$) δ 8.30 (d, *J* = 7.5 Hz, 0.32H), 8.24 (d, *J* = 8.4 Hz, 1H), 7.75 (brs, 3H), 7.53 (s, 3H), 7.44 (dd, *J* = 10.6, 8.5 Hz,

3H), 7.37 (ddd, J = 8.4, 5.3, 3.3 Hz, 2H), 7.25 – 7.20 (m, 1H), 7.16 – 7.10 (m, 4H), 7.06 (d, J = 8.3 Hz, 0.55H), 6.94 (t, J = 6.5 Hz, 3H), 6.89 (s, 1H), 6.86 – 6.77 (m, 1H), 6.84 (d, J = 8.8 Hz, 0.54H), 6.63 (s, 1H), 6.35 (s, 0.1H), 6.03 (dd, J = 11.4, 1.3 Hz, 2H), 5.99 (s, 0.38H), 2.33 (s, 0.7H), 2.29 (s, 3H), 2.27 (s, 3H), 2.26 (s, 0.7H). ^{13}C NMR (101 MHz, $CDCl_3$) δ 150.09, 149.36, 147.18, 144.80, 144.72, 136.81, 135.38, 135.18, 131.86, 130.89, 129.48, 129.42, 129.28, 129.04, 128.64, 127.77, 127.39, 126.99, 126.90, 125.24, 124.22, 120.03, 116.17, 116.11, 112.55, 111.76, 111.20, 102.25, 21.62, 21.56. HRMS (ESI) calcd for $C_{37}H_{27}NO_6S_2BrNaI$ $[M+Na]^+$ 873.9406; found: 873.9410.

(*E*)-3-(1-iodo-4-phenyl-2-tosylbut-1-en-1-yl)-2-phenyl-1-tosylindole (27): The title compound was prepared according to the general procedure D via column chromatography of silica eluting hexane-ethyl acetate (82:18) to obtain as a yellow solid (45 mg, yield = 59%); Mp. 142-143 °C; ^{1}H NMR (400 MHz, $CDCl_3$) δ 8.24 (d, J = 8.4 Hz, 1H), 7.68 (brs, 2H), 7.52 – 7.45 (m, 3H), 7.45 – 7.41 (m, 2H), 7.35 (ddd, J = 8.5, 7.3, 1.3 Hz, 1H), 7.29 – 7.26 (m, 1H), 7.25 (d, J = 0.7 Hz, 1H), 7.21 – 7.10 (m, 8H), 7.02 (dd, J = 1.2, 0.7 Hz, 1H), 6.98 (d, J = 7.9 Hz, 2H), 2.85 (dq, J = 13.5, 5.9 Hz, 2H), 2.77 – 2.63 (m, 2H), 2.30 (s, 3H), 2.28 (s, 3H). ^{13}C NMR (101 MHz, $CDCl_3$) δ 149.46, 144.82, 144.54, 140.34, 136.83, 136.56, 136.52, 135.14, 130.93, 129.47, 129.45, 129.08, 128.47, 128.28, 128.03, 127.50, 127.36, 126.97, 126.34, 126.28, 125.18, 124.17, 119.29, 116.29, 107.33, 42.08, 33.89, 21.55, 21.52. HRMS (ESI) calcd for $C_{38}H_{32}NO_4S_2NaI$ $[M+Na]^+$ 780.0715; found: 780.0711.

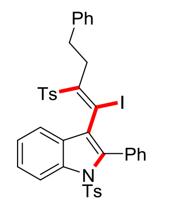

27

(*E*)-3-(1-iodo-2-phenyl-2-(phenylsulfonyl)vinyl)-2-phenyl-1-tosylindole (28): The title compound was prepared according to the general procedure D via column chromatography of silica eluting hexane-ethyl acetate (83:17) to obtain as a white solid (62 mg, yield = 88%); Mp. 187-188 °C; ^{1}H NMR (400 MHz, $CDCl_3$) δ 8.30 (d, J = 9.1 Hz, 1H), 7.75 (brs, 2H), 7.58 – 7.51 (m, 3H), 7.46 – 7.40 (m, 5H), 7.39 – 7.34 (m, 1H), 7.29 (ddd, J = 7.4, 4.4, 1.3 Hz, 1H), 7.25 – 7.19 (m, 2H), 7.17 (t, J = 3.2 Hz, 2H), 7.13 – 7.09 (m, 2H), 7.08 – 7.04 (m, 2H), 6.89 (d, J = 7.5 Hz, 1H), 6.80 (d, J = 7.5 Hz, 1H), 2.26 (s, 3H). ^{13}C NMR (101 MHz, $CDCl_3$) δ 151.08, 144.72, 138.70, 138.67, 137.17, 136.29, 135.16, 133.36, 131.10, 130.13, 130.03, 129.44, 129.20, 129.12, 128.57, 128.40, 128.32, 128.15, 127.97, 127.46, 126.83, 126.04, 125.52, 124.55, 119.31, 116.55, 108.85, 21.54. HRMS (ESI) calcd for $C_{35}H_{27}NO_4S_2I$ $[M+H]^+$ 716.0426; found: 716.0421.

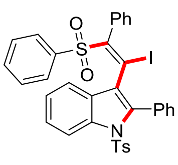

28

(*E*)-3-(1-iodo-2-((4-methoxyphenyl)sulfonyl)-2-phenylvinyl)-2-phenyl-1-tosylindole (29): The title compound was prepared according to the general procedure E via column chromatography of silica eluting hexane-ethyl acetate (80:20) to obtain as a white solid (61 mg, yield = 82%); Mp. 170-172 °C; ^{1}H NMR (400 MHz, $CDCl_3$) δ 8.29 (d, J = 8.0 Hz, 1H), 7.75 (brs, 2H), 7.54 (d, J = 2.4 Hz, 3H), 7.42 (dt, J = 8.8, 1.5 Hz, 4H), 7.38 – 7.29 (m, 2H), 7.27 – 7.21 (m, 2H), 7.10 (d, J = 8.2 Hz, 2H), 6.97 (d, J = 8.9 Hz, 2H), 6.92 (d, J = 8.3 Hz, 1H), 6.82 (d, J = 7.2 Hz, 1H), 6.62 (d, J = 8.9 Hz, 2H), 3.78 (s, 3H), 2.26 (s, 3H). ^{13}C NMR (101 MHz, $CDCl_3$) δ 163.49, 151.69, 144.70, 138.97, 137.15, 136.24, 135.14, 131.11, 130.83, 130.22, 130.11, 129.98, 129.43, 129.12, 129.07, 128.30, 128.17, 127.94,

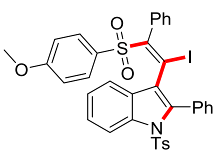

29

127.43, 126.82, 126.15, 125.45, 124.47, 119.37, 116.51, 113.58, 107.83, 55.55, 21.54. HRMS (ESI) calcd for $C_{36}H_{28}NO_5S_2NaI$ [M+H]$^+$ 768.0351; found: 768.0352.

(*E*)-3-(2-((4-(tert-butyl)phenyl)sulfonyl)-1-iodo-2-phenylvinyl)-2-phenyl-1-tosylindole (30): The title compound was prepared according to the general procedure E via column chromatography of silica eluting hexane-ethyl acetate (88:12) to obtain as a white solid (55 mg, yield = 71%); ^{1}H NMR (400 MHz, $CDCl_3$) δ 8.29 (d, *J* = 8.3 Hz, 1H), 7.76 (brs, 2H), 7.58 – 7.51 (m, 3H), 7.45 – 7.37 (m, 4H), 7.36 – 7.32 (m, 1H), 7.29 (ddd, *J* = 7.4, 4.4, 1.1 Hz, 1H), 7.24 – 7.19 (m, 2H), 7.18 – 7.14 (m, 2H), 7.11 (d, *J* = 8.1 Hz, 2H), 7.03 – 6.97 (m, 2H), 6.85 (dd, *J* = 13.5, 7.6 Hz, 2H), 2.26 (s, 3H), 1.26 (s, 9H). ^{13}C NMR (101 MHz, $CDCl_3$) δ 157.46, 151.69, 144.70, 138.95, 137.09, 136.46, 135.69, 135.16, 131.11, 130.04, 129.97, 129.44, 129.10, 128.47, 128.26, 128.03, 127.85, 127.42, 126.85, 126.00, 125.44, 125.36, 124.46, 119.35, 116.45, 108.30, 35.10, 30.97, 21.54. HRMS (ESI) calcd for $C_{39}H_{35}NO_4S_2I$ [M+H]$^+$ 772.1052; found: 772.1041.

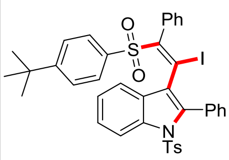

30

(*E*)-3-(2-((4-fluorophenyl)sulfonyl)-1-iodo-2-phenylvinyl)-2-phenyl-1-tosylindole (31): The title compound was prepared according to the general procedure E via column chromatography of silica eluting hexane-ethyl acetate (81:19) to obtain as a white solid (55 mg, yield = 75%); Mp. 195-197 °C; ^{1}H NMR (400 MHz, $CDCl_3$) δ 8.31 (d, *J* = 8.3 Hz, 1H), 7.75 (brs, 2H), 7.58 – 7.53 (m, 3H), 7.46 – 7.43 (m, 1H), 7.43 – 7.38 (m, 4H), 7.37 – 7.29 (m, 2H), 7.27 (d, *J* = 1.5 Hz, 1H), 7.25 – 7.23 (m, 1H), 7.11 (d, *J* = 8.0 Hz, 2H), 7.02 (dd, *J* = 9.0, 5.1 Hz, 2H), 6.94 (d, *J* = 8.3 Hz, 1H), 6.82 (ddd, *J* = 15.1, 8.6, 5.2 Hz, 3H), 2.27 (s, 3H). ^{13}C NMR (101 MHz, $CDCl_3$) δ 166.78, 164.23, 150.83, 144.77, 138.55, 137.22, 136.18, 135.12, 134.75, 131.44, 131.35, 131.07, 130.13, 130.02, 129.44, 129.37, 129.13, 128.45, 128.17, 128.13, 127.50, 126.84, 125.95, 125.61, 124.59, 119.18, 116.67, 115.81, 115.59, 109.02, 21.55. HRMS (ESI) calcd for $C_{35}H_{26}NO_4FS_2I$ [M+H]$^+$ 734.0332; found: 734.0337.

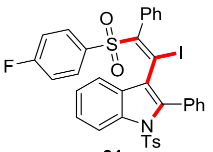

31

(*E*)-3-(2-((4-chlorophenyl)sulfonyl)-1-iodo-2-phenylvinyl)-2-phenyl-1-tosylindole (32): The title compound was prepared according to the general procedure E via column chromatography of silica eluting hexane-ethyl acetate (80:20) to obtain as a white solid (49 mg, yield = 65%); Mp. 205-206 °C; ^{1}H NMR (400 MHz, $CDCl_3$) δ 8.31 (d, *J* = 8.3 Hz, 1H), 7.75 (brs, 2H), 7.58 – 7.52 (m, 3H), 7.46 – 7.42 (m, 1H), 7.42 – 7.39 (m, 2H), 7.37 – 7.34 (m, 2H), 7.34 – 7.30 (m, 1H), 7.28 – 7.23 (m, 2H), 7.12 (dd, *J* = 2.0, 1.4 Hz, 2H), 7.10 (dd, *J* = 3.9, 1.4 Hz, 2H), 6.96 (d, *J* = 6.8 Hz, 1H), 6.94 – 6.90 (m, 2H), 6.80 (d, *J* = 8.0 Hz, 1H), 2.27 (s, 3H). ^{13}C NMR (101 MHz, $CDCl_3$) δ 150.64, 144.78, 140.12, 138.39, 137.20, 137.17, 136.18, 135.06, 131.02, 130.13, 130.02, 129.91, 129.43, 129.14, 128.69, 128.48, 128.16, 128.09, 127.49, 126.82, 125.85, 125.60, 124.59, 119.14, 116.65, 109.37, 21.54. HRMS (ESI) calcd for $C_{35}H_{26}NO_4S_2ClI$ [M+H]$^+$ 750.0036; found750.0037.

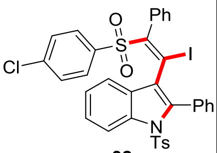

32

(*E*)-3-(2-((4-bromophenyl)sulfonyl)-1-iodo-2-phenylvinyl)-2-phenyl-1-tosylindole (33): The title compound was prepared according to the general procedure E via column chromatography of silica

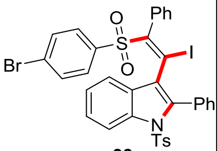

33

eluting hexane-ethyl acetate (80:20) to obtain as a white solid (56 mg, yield = 70%); Mp. 192-193 °C; ^{1}H NMR (400 MHz, $CDCl_3$) δ 8.31 (d, *J* = 8.3 Hz, 1H), 7.75 (brs, 2H), 7.58 – 7.52 (m, 3H), 7.46 – 7.42 (m, 1H), 7.40 (d, *J* = 8.5 Hz, 2H), 7.35 (d, *J* = 4.0 Hz, 2H), 7.32 (d, *J* = 7.3 Hz, 1H), 7.29 – 7.24 (m, 4H), 7.10 (d, *J* = 8.1 Hz, 2H), 6.96 (d, *J* = 7.0 Hz, 1H), 6.85 (d, *J* = 8.7 Hz, 2H), 6.80 (d, *J* = 7.7 Hz, 1H), 2.27 (s, 3H). ^{13}C NMR (101 MHz, $CDCl_3$) δ 150.63, 144.79, 138.37, 137.77, 137.17, 136.21, 135.08, 131.68, 131.01, 130.14, 130.04, 129.96, 129.44, 129.15, 128.79, 128.49, 128.18, 128.07, 127.50, 126.83, 125.83, 125.61, 124.60, 119.14, 116.66, 109.43, 21.54. HRMS (ESI) calcd for $C_{35}H_{26}NO_4S_2BrI$ $[M+H]^+$ 793.9531; found: 793.9538.

(*E*)-3-(1-iodo-2-((4-iodophenyl)sulfonyl)-2-phenylvinyl)-2-phenyl-1-tosylindole (34): The title compound was prepared according to the general procedure E via column chromatography of silica eluting hexane-ethyl acetate (80:20) to obtain as a white solid (65 mg, yield = 78%); Mp. 187-188 °C; ^{1}H NMR (400 MHz, $CDCl_3$) δ 8.30 (d, *J* = 8.3 Hz, 1H), 7.75 (brs, 2H), 7.58 – 7.52 (m, 3H), 7.50 – 7.46 (m, 2H), 7.46 – 7.42 (m, 1H), 7.42 – 7.38 (m, 2H), 7.35 – 7.30 (m, 3H), 7.29 – 7.23 (m, 2H), 7.10 (d, *J* = 8.1 Hz, 2H), 6.97 (d, *J* = 7.0 Hz, 1H), 6.81 (d, *J* = 7.7 Hz, 1H), 6.69 (d, *J* = 8.6 Hz, 2H), 2.26 (s, 3H). ^{13}C NMR (101 MHz, $CDCl_3$) δ 150.66, 144.79, 138.44, 138.35, 137.64, 137.12, 136.22, 135.05, 130.98, 130.12, 130.02, 129.72, 129.42, 129.14, 128.48, 128.16, 128.01, 127.48, 126.82, 125.78, 125.60, 124.59, 119.13, 116.62, 109.41, 101.53, 21.54. HRMS (ESI) calcd for $C_{35}H_{26}NO_4S_2I_2$ $[M+H]^+$ 841.9393; found: 841.9400.

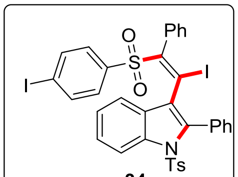

34

(*E*)-3-(1-iodo-2-((2-nitrophenyl)sulfonyl)-2-phenylvinyl)-2-phenyl-1-tosylindole (35): The title compound was prepared according to the general procedure E via column chromatography of silica eluting hexane-ethyl acetate (75:25) to obtain as a yellow solid (41 mg, yield = 53%); Mp. 197-198 °C; ^{1}H NMR (400 MHz, $CDCl_3$) δ 8.13 (d, *J* = 8.2 Hz, 1H), 7.60 – 7.48 (m, 7H), 7.37 – 7.21 (m, 11H), 7.05 (d, *J* = 8.0 Hz, 3H), 2.27 (s, 3H). ^{13}C NMR (101 MHz, $CDCl_3$) δ 151.60, 147.52, 144.94, 137.61, 137.08, 136.95, 134.91, 134.12, 133.53, 131.92, 131.56, 130.53, 130.14, 129.82, 129.68, 129.48, 129.44, 128.54, 128.47, 127.52, 127.31, 126.74, 125.51, 124.33, 124.28, 120.42, 116.16, 104.24, 21.54. HRMS (ESI) calcd for $C_{35}H_{25}N_2O_6NaS_2I$ $[M+Na]^+$ 783.0096; found: 783.0083.

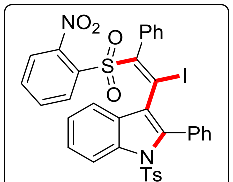

35

((*E*)-3-(1-iodo-2-phenyl-2-((4-(trifluoromethyl)phenyl)sulfonyl)vinyl)-2-phenyl-1-tosylindole (36): The title compound was prepared according to the general procedure E via column chromatography of silica eluting hexane-ethyl acetate (80:20) to obtain as a white solid (49 mg, yield = 62%); Mp. 199-201 °C; ^{1}H NMR (400 MHz, $CDCl_3$) δ 8.31 (d, *J* = 8.4 Hz, 1H), 7.77 (brs, 2H), 7.60 – 7.53 (m, 3H), 7.46 – 7.41 (m, 2H), 7.39 (t, *J* = 5.5 Hz, 3H), 7.36 – 7.31 (m, 3H), 7.28 – 7.23 (m, 3H), 7.15 – 7.10 (m, 4H), 6.96 (d, *J* = 7.5 Hz, 1H), 6.81 (d, *J* = 7.2 Hz, 1H), 2.28 (s, 3H). ^{13}C NMR (101 MHz, $CDCl_3$) δ 150.28, 144.86, 142.34, 138.13, 137.17, 136.31, 135.09, 130.96, 130.14, 129.58, 129.46, 129.23, 129.00, 128.57, 128.25, 127.98, 127.54, 126.87, 125.68, 125.62, 125.46, 125.42, 124.63, 119.03, 116.70, 110.20, 109.98, 21.55. HRMS (ESI) calcd for $C_{36}H_{26}NO_4F_3S_2$ $[M+H]^+$ 784.0300; found: 784.0303.

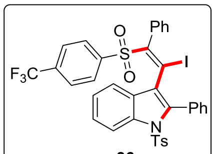

36

(*E*)-3-(1-iodo-2-phenyl-2-((2,4,5-trichlorophenyl)sulfonyl)vinyl)-2-phenyl-1-tosylindole (37): The title compound was prepared according to the general procedure E via column chromatography of silica eluting hexane-ethyl acetate (75:25) to obtain as a white solid (34 mg, yield = 40%); Mp. 197-198 °C; ^{1}H NMR (400 MHz, $CDCl_3$) δ 8.24 (d, *J* = 8.3 Hz, 1H), 7.97 (s, 1H), 7.57 (brs, 3H), 7.49 (dt, *J* = 7.1, 2.6 Hz, 3H), 7.41 – 7.38 (m, 1H), 7.37 – 7.34 (m, 2H), 7.33 – 7.27 (m, 3H), 7.21 (d, *J* = 8.4 Hz, 2H), 7.13 (s, 1H), 7.03 (d, *J* = 8.0 Hz, 2H), 6.96 (d, *J* = 7.0 Hz, 1H), 2.26 (s, 3H). ^{13}C NMR (101 MHz, $CDCl_3$) δ 149.02, 144.89, 138.08, 137.36, 137.22, 137.07, 136.51, 134.85, 132.23, 131.89, 131.65, 131.14, 130.10, 129.91, 129.85, 129.72, 129.58, 129.37, 128.67, 128.34, 127.41, 127.25, 126.81, 126.70, 125.89, 124.32, 124.25, 119.71, 116.65, 116.52, 108.08, 21.55. HRMS (ESI) calcd for $C_{35}H_{24}NO_4S_2C_{l3}I$ $[M+H]^+$ 817.9257; found: 817.9258.

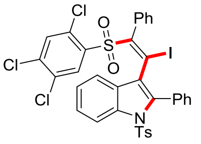

37

(*E*)-3-(1-iodo-2-(methylsulfonyl)-2-phenylvinyl)-2-phenyl-1-tosylindole (38): The title compound was prepared according to the general procedure E via column chromatography of silica eluting hexane-ethyl acetate (83:17) to obtain as a white solid (35 mg, yield = 54%); Mp. 164-165 °C; ^{1}H NMR (400 MHz, $CDCl_3$) δ 8.27 (d, *J* = 7.5 Hz, 1H), 8.00 (t, *J* = 7.3 Hz, 1H), 7.68 (dd, *J* = 16.0, 9.0 Hz, 2H), 7.57 – 7.51 (m, 4H), 7.48 – 7.38 (m, 5H), 7.34 (d, *J* = 8.3 Hz, 2H), 7.09 (d, *J* = 8.2 Hz, 3H), 2.29 (s, 3H), 2.16 (s, 3H). ^{13}C NMR (101 MHz, $CDCl_3$) δ 149.52, 144.73, 138.71, 137.21, 135.35, 135.00, 131.22, 129.83, 129.41, 129.25, 129.14, 128.86, 127.49, 126.65, 125.71, 124.74, 122.70, 119.46, 116.57, 109.86, 41.00, 21.55. HRMS (ESI) calcd for $C_{30}H_{25}NO_4S_2I$ $[M+H]^+$ 654.0270; found: 654.0273.

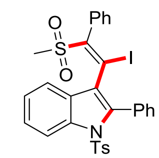

38

(*E*)-3-(2-(ethylsulfonyl)-1-iodo-2-phenylvinyl)-2-phenyl-1-tosylindole (39): The title compound was prepared according to the general procedure E via column chromatography of silica eluting hexane-ethyl acetate (83:17) to obtain as a white solid (40 mg, yield = 60%); Mp. 100-103 °C; ^{1}H NMR (400 MHz, $CDCl_3$) δ 8.26 (d, *J* = 7.2 Hz, 1H), 7.68 (brs, 2H), 7.56 – 7.50 (m, 4H), 7.49 – 7.46 (m, 1H), 7.45 – 7.42 (m, 2H), 7.41 (d, *J* = 1.7 Hz, 1H), 7.40 – 7.37 (m, 2H), 7.35 – 7.32 (m, 2H), 7.11 – 7.04 (m, 3H), 2.44 (dd, *J* = 13.6, 7.4 Hz, 1H), 2.29 (s, 3H), 2.07 (dd, *J* = 13.6, 7.5 Hz, 1H), 0.98 (t, *J* = 7.4 Hz, 3H). ^{13}C NMR (101 MHz, $CDCl_3$) δ 148.84, 144.65, 138.64, 137.17, 135.41, 135.03, 131.22, 129.76, 129.65, 129.39, 129.09, 128.81, 128.62, 127.39, 126.67, 126.41, 125.63, 124.67, 119.46, 116.53, 110.23, 47.15, 21.55, 5.53. HRMS (ESI) calcd for $C_{31}H_{27}NO_4S_2I$ $[M+H]^+$ 668.0426; found: 668.0424.

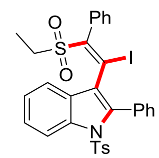

39

(*E*)-3-(2-(cyclopropylsulfonyl)-1-iodo-2-phenylvinyl)-2-phenyl-1-tosylindole (40): The title compound was prepared according to the general procedure E via column chromatography of silica eluting hexane-ethyl acetate (84:16) to obtain as a white solid (53 mg, yield = 77%); Mp. 176-178 °C; ^{1}H NMR (400 MHz, $CDCl_3$) δ 8.28 – 8.24 (m, 1H), 7.68 (brs, 2H), 7.56 – 7.50 (m, 4H), 7.47 – 7.37 (m, 6H), 7.36 – 7.32 (m, 2H), 7.11 (d, *J* = 6.2 Hz, 1H), 7.07 (d, *J* = 8.0 Hz, 2H), 2.28 (s, 3H), 1.67 (tt, *J* = 7.9, 4.9 Hz, 1H), 0.74 – 0.68 (m, 2H), 0.65 – 0.59 (m, 2H). ^{13}C NMR (101 MHz, $CDCl_3$) δ 150.18, 144.60, 138.89, 137.12, 135.96, 135.05, 131.13, 130.06, 129.96, 129.56, 129.45, 129.39, 129.08, 128.63,

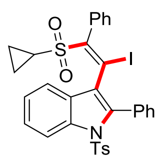

40

128.54, 127.37, 126.71, 126.23, 125.58, 124.63, 119.37, 116.50, 108.26, 30.73, 21.54, 5.93, 5.27. HRMS (ESI) calcd for $C_{32}H_{27}NO_4S_2I$ [M+H]$^+$ 680.0426; found: 680.0425.

(*E*)-3-(1-iodo-2-(naphthalen-1-ylsulfonyl)-2-phenylvinyl)-2-phenyl-1-tosylindole (41): The title compound

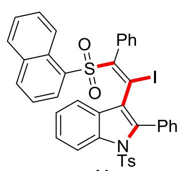

41

was prepared according to the general procedure D via column chromatography of silica eluting hexane-ethyl acetate (75:25) to obtain as a white solid (79 mg, yield = 90%); Mp. 204-205 °C; ^{1}H NMR (400 MHz, $CDCl_3$) δ 8.28 (d, *J* = 8.4 Hz, 1H), 7.80 (d, *J* = 8.1 Hz, 2H), 7.66 – 7.60 (m, 3H), 7.60 – 7.49 (m, 6H), 7.46 (d, *J* = 8.4 Hz, 2H), 7.39 – 7.35 (m, 1H), 7.33 (dd, *J* = 7.0, 4.3 Hz, 2H), 7.28 (t, *J* = 7.4 Hz, 1H), 7.25 – 7.21 (m, 2H), 7.17 (d, *J* = 7.0 Hz, 1H), 7.13 (d, *J* = 8.2 Hz, 2H), 7.09 (dd, *J* = 8.6, 1.8 Hz, 1H), 6.88 (d, *J* = 7.1 Hz, 2H), 2.26 (s, 3H). ^{13}C NMR (101 MHz, $CDCl_3$) δ 151.37, 144.75, 138.80, 137.10, 136.49, 135.62, 135.17, 134.97, 131.67, 131.09, 130.62, 130.15, 130.08, 129.48, 129.25, 129.24, 129.18, 128.53, 128.38, 127.96, 127.89, 127.74, 127.46, 127.33, 126.91, 125.91, 125.50, 124.35, 123.19, 119.27, 116.46, 109.10, 21.54. HRMS (ESI) calcd for $C_{39}H_{29}NO_4S_2I$ [M+H]$^+$ 766.0583; found: 766.0581.

((*E*)-3-(1-iodo-2-phenyl-2-(thiophen-2-ylsulfonyl)vinyl)-2-phenyl-1-tosylindole (42): The title compound

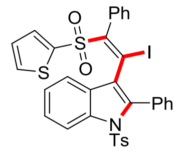

42

was prepared according to the general procedure E via column chromatography of silica eluting hexane-ethyl acetate (82:18) to obtain as a white solid (46 mg, yield = 63%); Mp. 198-200 °C; ^{1}H NMR (400 MHz, $CDCl_3$) δ 8.29 (d, *J* = 7.4 Hz, 1H), 7.70 (brs, 2H), 7.54 – 7.50 (m, 5H), 7.43 (dd, *J* = 7.3, 1.5 Hz, 1H), 7.41 – 7.38 (m, 3H), 7.34 – 7.30 (m, 1H), 7.26 (d, *J* = 6.6 Hz, 2H), 7.08 (dd, *J* = 13.6, 7.9 Hz, 3H), 6.82 (t, *J* = 5.1 Hz, 3H), 2.26 (s, 3H). ^{13}C NMR (101 MHz, $CDCl_3$) δ 151.05, 144.69, 139.68, 138.65, 137.08, 135.12, 134.64, 131.08, 129.95, 129.87, 129.44, 129.34, 129.17, 128.36, 128.12, 128.06, 127.43, 127.32, 126.77, 125.53, 124.57, 119.38, 116.39, 108.41, 21.54. HRMS (ESI) calcd for $C_{33}H_2NO_4S_3I$ [M+H]$^+$ 721.9990; found: 721.9996.

(*E*)-3-(1-iodo-2-phenyl-2-tosylvinyl)-2-phenyl-1-(phenylsulfonyl)indole (43): The title compound was

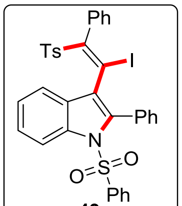

43

prepared according to the general procedure D via column chromatography of silica eluting hexane-ethyl acetate (82:18) to obtain as a white solid (53 mg, yield = 72%); Mp. 184-185 °C; ^{1}H NMR (400 MHz, $CDCl_3$) δ 8.31 (d, *J* = 8.3 Hz, 1H), 7.72 (brs, 2H), 7.57 – 7.50 (m, 5H), 7.40 (dddd, *J* = 15.7, 7.8, 3.7, 1.3 Hz, 4H), 7.33 – 7.28 (m, 3H), 7.24 – 7.18 (m, 2H), 6.97 – 6.87 (m, 5H), 6.82 (d, *J* = 7.5 Hz, 1H), 2.30 (s, 3H). ^{13}C NMR (101 MHz, $CDCl_3$) δ 151.48, 144.45, 138.78, 137.97, 137.20, 136.18, 135.69, 133.67, 130.99, 130.11, 129.98, 129.14, 129.04, 128.82, 128.61, 128.29, 128.18, 127.93, 127.47, 126.78, 126.34, 125.54, 124.57, 119.42, 116.56, 108.14, 21.55. HRMS (ESI) calcd for $C_{35}H_{27}NO_4S_2I$ [M+H]$^+$ 716.0426; found: 716.0422.

(*E*)-1-((4-chlorophenyl)sulfonyl)-3-(1-iodo-2-phenyl-2-tosylvinyl)-2-phenylindole (44): The title compound was prepared according to the general procedure D via column chromatography of silica eluting hexane-ethyl acetate (83:17) to obtain as a white solid (55 mg, yield = 77%); Mp. 193-194 °C; ^{1}H NMR (400 MHz, $CDCl_3$) δ 8.27 (d, *J* = 9.2 Hz, 1H), 7.73 (brs, 2H), 7.59 – 7.52 (m, 3H), 7.49 – 7.37 (m, 5H), 7.34 – 7.19 (m, 6H), 6.97 (dt, *J* = 6.4, 5.2 Hz, 4H), 6.90 (d, *J* = 7.4 Hz, 1H), 6.83 (d, *J* = 7.6 Hz, 1H), 2.33 (s, 3H). ^{13}C NMR (101 MHz, $CDCl_3$) δ 151.57, 144.56, 140.30, 138.68, 137.14, 136.30, 136.04, 135.57, 130.89, 130.13, 129.97, 129.27, 129.20, 129.15, 129.10, 128.63, 128.36, 128.31, 128.22, 127.96, 127.58, 126.82, 125.76, 124.87, 119.59, 116.58, 107.73, 21.58. HRMS (ESI) calcd for $C_{35}H_{25}NO_4S_2ClNaI$ [M+Na]$^+$ 771.9856; found: 771.9851.

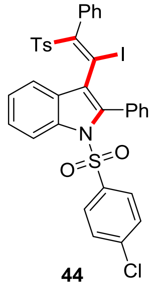

44

(*E*)-1-(ethylsulfonyl)-3-(1-iodo-2-phenyl-2-tosylvinyl)-2-phenylindole (45): The title compound was prepared according to the general procedure D via column chromatography of silica eluting hexane-ethyl acetate (83:17) to obtain as a white solid (48 mg, yield = 73%); Mp. 177-178 °C; ^{1}H NMR (400 MHz, $CDCl_3$) δ 8.25 – 8.18 (m, 1H), 7.85 (brs, 2H), 7.64 – 7.59 (m, 1H), 7.58 – 7.53 (m, 3H), 7.51 – 7.45 (m, 2H), 7.35 – 7.26 (m, 2H), 7.21 (d, *J* = 7.4 Hz, 1H), 7.19 – 7.15 (m, 2H), 7.11 – 7.07 (m, 2H), 6.98 (d, *J* = 6.9 Hz, 1H), 6.81 (d, *J* = 7.4 Hz, 1H), 3.13 (qd, *J* = 7.3, 3.4 Hz, 2H), 2.36 (s, 3H), 1.15 (t, *J* = 7.4 Hz, 3H). ^{13}C NMR (101 MHz, $CDCl_3$) δ 151.52, 144.75, 138.60, 137.08, 136.46, 135.50, 130.85, 130.78, 130.27, 130.16, 129.34, 129.21, 128.76, 128.40, 127.88, 127.61, 127.57, 125.69, 125.39, 124.51, 119.66, 115.81, 108.21, 48.69, 21.62, 7.52. HRMS (ESI) calcd for $C_{31}H_{27}NO_4S_2I$ [M+H]$^+$ 668.0426; found: 668.0423.

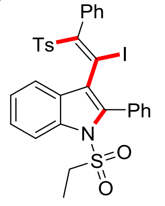

45

(*E*)-3-(1-bromo-2-phenyl-2-tosylvinyl)-2-phenyl-1-tosylindole (46): The title compound was prepared according to the general procedure F via column chromatography of silica eluting hexane-ethyl acetate (84:16) to obtain as a white solid (36 mg, yield = 52%); Mp. 178-179 °C; ^{1}H NMR (400 MHz, $CDCl_3$) δ 8.31 (d, *J* = 9.0 Hz, 1H), 7.72 (brs, 2H), 7.53 (s, 3H), 7.46 – 7.41 (m, 2H), 7.41 – 7.38 (m, 2H), 7.37 – 7.26 (m, 2H), 7.25 – 7.19 (m, 2H), 7.10 (d, *J* = 8.0 Hz, 2H), 7.01 – 6.89 (m, 5H), 6.81 (s, 1H), 2.31 (s, 3H), 2.28 (s, 3H). ^{13}C NMR (101 MHz, $CDCl_3$) δ 147.27, 144.76, 144.42, 138.07, 137.12, 135.75, 135.23, 135.04, 130.54, 130.11, 129.41, 129.21, 129.03, 129.00, 128.77, 128.64, 128.53, 128.04, 127.40, 126.83, 125.45, 124.51, 122.45, 119.16, 116.50, 21.56, 21.54. HRMS (ESI) calcd for $C_{36}H_{29}NO_4S_2Br$ [M+H]$^+$ 682.0721; found: 682.0712.

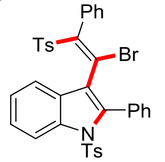

46

(*E*)-3-(1-bromo-2-phenyl-2-tosylvinyl)-1-(methylsulfonyl)-2-phenylindole (47): The title compound was prepared according to the general procedure F via column chromatography of silica eluting hexane-ethyl acetate (82:18) to obtain as a white solid (16 mg, yield = 20%); Mp. 138.4-139.5 °C; ^{1}H NMR (400 MHz, $CDCl_3$) δ 8.25 – 8.19 (m, 1H), 7.82 (brs, 2H), 7.61 (dd, *J* = 6.2, 2.9 Hz, 1H), 7.56 (dd, *J* = 6.6, 3.5 Hz, 3H), 7.52 – 7.47 (m, 2H), 7.32 (t, *J* = 7.4 Hz, 1H), 7.26 (brs, 2H), 7.14 (d, *J* = 8.4 Hz, 2H), 7.07 (d, *J* = 8.2 Hz, 2H), 6.93 (s, 2H), 2.91 (s, 3H), 2.36 (s, 3H). ^{13}C NMR (101 MHz, $CDCl_3$) δ 147.66, 144.80, 137.89, 137.11, 135.48, 134.77, 130.77, 130.34, 130.24, 129.48,

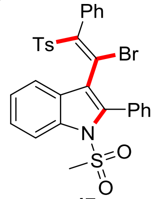

47

129.22, 129.15, 128.78, 128.40, 127.74, 125.91, 124.97, 122.95, 119.47, 116.30, 40.43, 21.62. HRMS (ESI) calcd for $C_{30}H_{25}NO_4S_2Br$ $[M+H]^+$ 606.0408; found: 606.0402.

(*E*)-2-phenyl-1-(2-phenyl-1-tosylindol-3-yl)-2-(phenylsulfonyl)vinyl benzenesulfonate (48): The title compound was prepared according to the general procedure G via column chromatography of silica eluting hexane-ethyl acetate (80:20) to obtain as a white solid (8 mg, yield = 10%); Mp. 173-174 °C; ^1H NMR (400 MHz, $CDCl_3$) δ 8.30 (d, J = 9.0 Hz, 1H), 7.94 (dd, J = 8.5, 1.2 Hz, 1H), 7.84 – 7.78 (m, 1H), 7.78 – 7.68 (m, 1H), 7.66 – 7.59 (m, 2H), 7.58 – 7.52 (m, 3H), 7.47 – 7.40 (m, 5H), 7.40 – 7.32 (m, 2H), 7.31 – 7.27 (m, 1H), 7.24 – 7.20 (m, 2H), 7.20 – 7.14 (m, 3H), 7.10 (d, J = 8.0 Hz, 2H), 7.08 – 7.04 (m, 2H), 6.89 (d, J = 7.4 Hz, 1H), 6.80 (d, J = 7.7 Hz, 1H), 2.26 (s, 3H). ^{13}C NMR (101 MHz, $CDCl_3$) δ 151.07, 144.72, 138.67, 138.63, 137.15, 136.35, 136.27, 135.12, 133.37, 131.43, 131.14, 131.09, 130.20, 130.11, 130.01, 129.66, 129.43, 129.19, 129.10, 128.54, 128.39, 128.35, 128.31, 128.14, 127.96, 127.45, 126.81, 126.04, 125.51, 124.55, 119.30, 116.53, 108.83, 21.53. HRMS (ESI) calcd for $C_{41}H_{31}NO_7S_3Na$ $[M+Na]^+$ 768.1160; found: 768.1161.

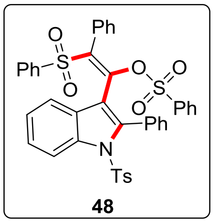

(*E*)-2-((4-(*tert*-butyl)phenyl)sulfonyl)-2-phenyl-1-(2-phenyl-1-tosylindol-3-yl)vinyl4-(*tert*-butyl)benzenesulfonate (49): The title compound was prepared according to the general procedure G via column chromatography of silica eluting hexane-ethyl acetate (83:17) to obtain as a white solid (7 mg, yield = 7%); Mp. 159-161 °C; ^1H NMR (400 MHz, $CDCl_3$) δ 8.22 – 8.15 (m, 1H), 7.77 – 7.72 (m, 1H), 7.49 (s, 1H), 7.46 – 7.40 (m, 3H), 7.34 (dd, J = 8.3, 7.3 Hz, 2H), 7.27 (ddd, J = 11.7, 5.5, 3.2 Hz, 5H), 7.21 (dd, J = 11.5, 4.6 Hz, 2H), 7.18 – 7.12 (m, 4H), 7.07 (d, J = 8.1 Hz, 4H), 6.88 (s, 1H), 6.82 (d, J = 8.7 Hz, 2H), 2.26 (s, 3H), 1.34 (s, 9H), 1.29 (s, 9H). ^{13}C NMR (101 MHz, $CDCl_3$) δ 157.90, 157.64, 145.70, 144.77, 141.16, 141.02, 136.61, 135.69, 135.29, 132.99, 130.76, 130.18, 130.02, 129.52, 129.46, 129.37, 128.91, 128.63, 127.88, 127.20, 126.63, 125.41, 125.38, 125.29, 124.43, 121.10, 115.87, 115.83, 35.18, 35.16, 30.99, 30.91, 22.31, 21.54, 14.04. HRMS (ESI) calcd for $C_{49}H_{47}NO_7S_3Na$ $[M+Na]^+$ 880.2412; found: 880.2414.

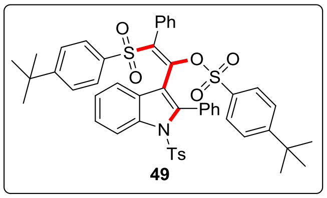

(*Z*)-2-(iodo(phenyl)methylene)-3-phenyl-1,4-ditosyl-2,5-dihydropyrrole (50): The title compound was prepared according to the general procedure D via column chromatography of silica eluting hexane-ethyl acetate (84:16) to obtain as a white solid (16 mg, yield = 23%); Mp. 169-170 °C; ^1H NMR (400 MHz, $CDCl_3$) δ 7.86 (d, J = 8.3 Hz, 2H), 7.39 (d, J = 8.0 Hz, 2H), 7.01 (s, 4H), 6.89 (dd, J = 7.2, 3.9 Hz, 2H), 6.86 – 6.81 (m, 1H), 6.77 (t, J = 7.2 Hz, 2H), 6.70 (t, J = 7.8 Hz, 2H), 6.12 (d, J = 7.1 Hz, 2H), 4.66 (s, 2H), 2.51 (s, 3H), 2.35 (s, 3H). ^{13}C NMR (101 MHz, $CDCl_3$) δ 145.80, 145.68, 144.97, 144.53, 140.76, 140.60, 136.47, 133.33, 130.35, 129.90, 129.29, 129.25, 128.67, 128.35, 128.25, 127.77, 127.61, 127.33, 126.96, 101.76, 54.51, 21.74, 21.58. HRMS (ESI) calcd for $C_{31}H_{27}NO_4S_2I$ $[M+H]^+$ 668.0426; found: 668.0423.

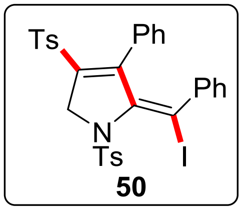

(*E*)-2-phenyl-3-(2-phenyl-1-(phenylselanyl)-2-tosylvinyl)-1-tosylindole (51): The title compound was prepared according to the general procedure H via column chromatography of silica eluting hexane-ethyl acetate (85:15) to obtain as a white solid (51 mg, yield = 68%); Mp. 196-197 °C; ^{1}H NMR (400 MHz, $CDCl_3$) δ 8.08 (d, *J* = 8.3 Hz, 1H), 7.48 – 7.35 (m, 7H), 7.30 (ddd, *J* = 8.4, 6.8, 1.7 Hz, 2H), 7.27 – 7.17 (m, 4H), 7.11 (d, *J* = 7.4 Hz, 1H), 7.09 – 7.04 (m, 3H), 6.95 (s, 4H), 6.76 (t, *J* = 7.8 Hz, 2H), 6.60 – 6.55 (m, 2H), 2.34 (s, 3H), 2.20 (s, 3H). ^{13}C NMR (101 MHz, $CDCl_3$) δ 146.48, 144.43, 143.87, 140.15, 137.47, 136.88, 136.78, 136.63, 134.89, 134.59, 131.26, 130.63, 130.54, 130.19, 129.37, 129.32, 129.09, 128.87, 128.80, 128.60, 128.34, 127.94, 127.05, 125.79, 124.82, 124.01, 120.02, 119.70, 116.32, 21.53, 21.47. HRMS (ESI) calcd for $C_{42}H_{33}NO_4S_2Se$ $[M+H]^+$ 760.1097; found: 760.1097.

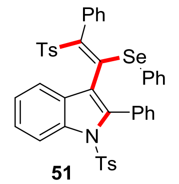

51

(*E*)-5-methyl-2-phenyl-3-(2-phenyl-1-(phenylselanyl)-2-tosylvinyl)-1-tosylindole (52): The title compound was prepared according to the general procedure H via column chromatography of silica eluting hexane-ethyl acetate (82:18) to obtain as a pale yellow solid (48 mg, yield = 62%); Mp. 226-227 °C; ^{1}H NMR (400 MHz, $CDCl_3$) δ 7.95 (d, *J* = 8.5 Hz, 1H), 7.51 – 7.32 (m, 9H), 7.28 (s, 1H), 7.19 (d, *J* = 7.3 Hz, 1H), 7.14 – 7.03 (m, 5H), 6.93 (d, *J* = 8.2 Hz, 2H), 6.90 – 6.86 (m, 2H), 6.83 (s, 1H), 6.78 (dd, *J* = 10.9, 4.6 Hz, 2H), 6.53 (d, *J* = 6.9 Hz, 2H), 2.36 (s, 3H), 2.35 (s, 3H), 2.21 (s, 3H). ^{13}C NMR (101 MHz, $CDCl_3$) δ 144.40, 143.74, 140.24, 137.70, 137.19, 136.58, 135.00, 134.88, 134.82, 133.52, 131.37, 130.56, 130.30, 129.42, 129.33, 129.08, 128.94, 128.76, 128.63, 128.38, 127.98, 127.18, 126.25, 125.94, 119.82, 119.54, 116.08, 21.55, 21.49, 21.31. HRMS (ESI) calcd for $C_{43}H_{35}NO_4S_2Se$ $[M+H]^+$ 774.1254; found: 774.1257.

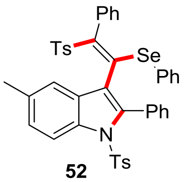

52

(*E*)-5-ethyl-2-phenyl-3-(2-phenyl-1-(phenylselanyl)-2-tosylvinyl)-1-tosylindole (53): The title compound was prepared according to the general procedure H via column chromatography of silica eluting hexane-ethyl acetate (82:18) to obtain as a lite yellow solid (45 mg, yield = 56%); Mp. 189-191 °C; ^{1}H NMR (400 MHz, $CDCl_3$) δ 7.96 (d, *J* = 8.5 Hz, 1H), 7.56 – 7.36 (m, 8H), 7.31 (d, *J* = 20.7 Hz, 3H), 7.14 – 7.04 (m, 5H), 6.95 – 6.90 (m, 5H), 6.76 (t, *J* = 7.8 Hz, 2H), 6.58 (d, *J* = 8.2 Hz, 2H), 2.75 – 2.59 (m, 2H), 2.34 (s, 3H), 2.21 (s, 3H), 1.29 (t, *J* = 7.6 Hz, 3H). ^{13}C NMR (101 MHz, $CDCl_3$) δ 147.13, 144.35, 143.76, 140.18, 139.91, 137.54, 137.16, 136.70, 135.15, 134.99, 134.78, 131.41, 130.49, 130.36, 129.39, 129.33, 129.09, 128.95, 128.81, 128.74, 128.65, 128.34, 127.92, 127.16, 126.79, 125.84, 125.17, 119.96, 118.32, 116.14, 28.67, 21.55, 21.49, 15.90. HRMS (ESI) calcd for $C_{44}H_{37}NO_4S_2Se$ $[M+H]^+$ 788.1411; found: 788.1407.

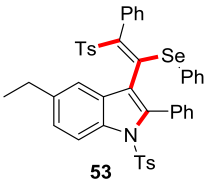

53

(*E*)-5-chloro-2-phenyl-3-(2-phenyl-1-(phenylselanyl)-2-tosylvinyl)-1-tosylindole (54): The title compound was prepared according to the general procedure H via column chromatography of silica eluting hexane-ethyl acetate (80:20) to obtain as a white solid (43 mg, yield = 55%); Mp. 207-208 °C; ^{1}H NMR (400 MHz, $CDCl_3$) δ 8.01 (d, *J* = 8.9 Hz, 1H), 7.52 – 7.40 (m, 6H), 7.39 – 7.35 (m, 3H), 7.29 (s, 1H), 7.22 (dd, *J* = 8.9, 2.1 Hz, 1H), 7.17 – 7.06 (m, 5H), 6.97 (s, 1H), 6.95 (d, *J* = 1.7 Hz, 2H), 6.87 (d, *J* = 8.4 Hz, 2H), 6.81 (t, *J* = 7.8 Hz, 2H), 6.60 (dd, *J* = 8.2, 1.2 Hz,

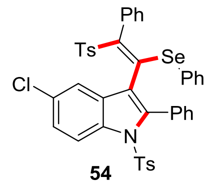

54

2H), 2.37 (s, 3H), 2.24 (s, 3H). ^{13}C NMR (101 MHz, $CDCl_3$) δ 145.92, 144.91, 144.27, 141.13, 139.03, 136.90, 136.77, 135.01, 134.67, 134.62, 131.37, 131.21, 130.46, 129.74, 129.52, 129.29, 129.19, 128.99, 128.48, 128.10, 127.21, 126.82, 125.73, 124.86, 119.16, 117.41, 21.62, 21.54. HRMS (ESI) calcd for $C_{42}H_{32}NO_4S_2SeCl$ $[M+H]^+$ 794.0706; found: 794.0704.

(*E*)-5-bromo-2-phenyl-3-(2-phenyl-1-(phenylselanyl)-2-tosylvinyl)-1-tosylindole (55): The title compound

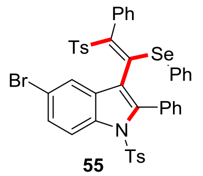

55

was prepared according to the general procedure H via column chromatography of silica eluting hexane-ethyl acetate (80:20) to obtain as a white solid (52 mg, yield = 62%); Mp. 208-209 °C; ^{1}H NMR (400 MHz, $CDCl_3$) δ 7.96 (d, *J* = 8.8 Hz, 1H), 7.55 – 7.39 (m, 6H), 7.39 – 7.26 (m, 6H), 7.09 (ddd, *J* = 10.5, 8.9, 4.7 Hz, 5H), 6.96 (d, *J* = 8.2 Hz, 2H), 6.87 (d, *J* = 8.3 Hz, 2H), 6.81 (t, *J* = 7.7 Hz, 2H), 6.61 (d, *J* = 6.9 Hz, 2H), 2.38 (s, 3H), 2.24 (s, 3H). ^{13}C NMR (101 MHz, $CDCl_3$) δ 145.87, 144.91, 144.27, 141.14, 138.85, 136.85, 136.76, 135.37, 134.65, 134.58, 131.73, 131.20, 130.43, 129.76, 129.68, 129.51, 129.28, 129.18, 129.03, 128.57, 128.46, 128.09, 127.74, 127.50, 127.19, 126.81, 125.69, 122.22, 118.92, 117.74, 117.45, 21.69, 21.53. HRMS (ESI) calcd for $C_{42}H_{32}NO_4S_2SeBr$ $[M+H]^+$ 838.0201; found: 838.0203.

(*E*)-3-(2-phenyl-1-(phenylselanyl)-2-tosylvinyl)-2-(p-tolyl)-1-tosylindole (56): The title compound was

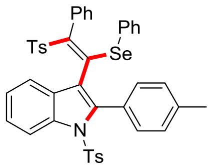

56

prepared according to the general procedure H via column chromatography of silica eluting hexane-ethyl acetate (82:18)to obtain as a white solid (55 mg, yield = 70%); Mp. 117-118 °C; ^{1}H NMR (400 MHz, $CDCl_3$) δ 8.05 (d, *J* = 8.3 Hz, 1H), 7.42 – 7.35 (m, 5H), 7.32 (s, 1H), 7.27 (t, *J* = 1.8 Hz, 1H), 7.25 – 7.19 (m, 5H), 7.16 (ddd, *J* = 12.0, 4.9, 1.8 Hz, 2H), 7.09 – 7.04 (m, 3H), 6.97 – 6.94 (m, 4H), 6.76 (t, *J* = 7.8 Hz, 2H), 6.58 (dd, *J* = 8.2, 1.2 Hz, 2H), 2.47 (s, 3H), 2.35 (s, 3H), 2.20 (s, 3H). ^{13}C NMR (101 MHz, $CDCl_3$) δ 146.82, 144.38, 143.84, 140.09, 138.76, 137.79, 137.00, 136.77, 136.72, 134.91, 134.70, 131.34, 130.73, 129.37, 129.31, 129.09, 128.87, 128.64, 128.39, 127.90, 127.56, 127.35, 127.08, 125.79, 124.65, 123.98, 119.71, 119.63, 116.37, 21.64, 21.55, 21.48. HRMS (ESI) calcd for $C_{43}H_{36}NO_4S_2Se$ $[M+H]^+$ 774.1173; found: 774.1254.

(*E*)-2-(4-methoxyphenyl)-3-(2-phenyl-1-(phenylselanyl)-2-tosylvinyl)-1-tosylindole (57): The title

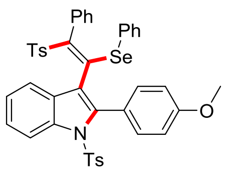

57

compound was prepared according to the general procedure H via column chromatography of silica eluting hexane-ethyl acetate (82:18) to obtain as a white solid (38 mg, yield = 50%); Mp. 174-175 °C; ^{1}H NMR (400 MHz, $CDCl_3$) δ 8.06 (d, *J* = 8.3 Hz, 1H), 7.54 – 7.31 (m, 6H), 7.30 – 7.23 (m, 3H), 7.22 – 7.17 (m, 2H), 7.13 – 7.04 (m, 4H), 6.94 (d, *J* = 8.0 Hz, 6H), 6.76 (t, *J* = 7.7 Hz, 2H), 6.58 (d, *J* = 6.9 Hz, 2H), 3.93 (s, 3H), 2.35 (s, 3H), 2.20 (s, 3H). ^{13}C NMR (101 MHz, $CDCl_3$) δ 160.06, 147.00, 144.39, 143.85, 140.12, 137.61, 136.98, 136.72, 136.66, 134.93, 134.67, 131.32, 130.65, 129.38, 129.31, 129.08, 128.87, 128.62, 128.36, 127.90, 127.05, 125.89, 124.57, 123.98, 122.61, 119.50, 119.28, 116.41, 112.22, 55.25, 21.54, 21.47. HRMS (ESI) calcd for $C_{42}H_{33}NO_5S_2Se$ $[M+H]^+$ 776.0965; found: 776.1047.

(*E*)-2-(3,4-dimethoxyphenyl)-3-(2-phenyl-1-(phenylselanyl)-2-tosylvinyl)-1-tosylindole (58): The title compound was prepared according to the general procedure H via column chromatography of silica eluting hexane-ethyl acetate (75:25) to obtain as a white solid (34 mg, yield = 40%); Mp. 138.4-139.5 °C; ^{1}H NMR (400 MHz, $CDCl_3$) δ 8.11 (d, *J* = 8.3 Hz, 1H), 7.48 – 7.37 (m, 3H), 7.36 – 7.28 (m, 5H), 7.21 (d, *J* = 5.8 Hz, 2H), 7.12 – 7.04 (m, 4H), 7.01 – 6.93 (m, 5H), 6.90 (d, *J* = 8.0 Hz, 1H), 6.78 (t, *J* = 7.7 Hz, 2H), 6.60 (d, *J* = 7.4 Hz, 2H), 4.00 (s, 3H), 3.97 (s, 3H), 2.36 (s, 3H), 2.22 (s, 3H). ^{13}C NMR (101 MHz, $CDCl_3$) δ 149.48, 147.25, 144.38, 143.94, 139.88, 137.61, 136.90, 136.64, 134.55, 131.37, 130.79, 129.44, 129.29, 129.08, 128.91, 128.67, 128.32, 127.91, 127.15, 126.07, 124.66, 123.87, 122.50, 119.48, 116.55, 109.28, 56.25, 55.83, 21.56, 21.46. HRMS (ESI) calcd for $C_{44}H_{37}NO_6S_2SeNa$ [M+Na]$^+$ 842.1127; found: 842.1129.

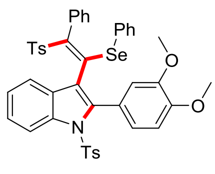

58

(*E*)-2-butyl-3-(2-phenyl-1-(phenylselanyl)-2-tosylvinyl)-1-tosylindole (59): The title compound was prepared according to the general procedure H via column chromatography of silica eluting hexane-ethyl acetate (85:15) to obtain as a white solid (28 mg, yield = 38%); Mp. 153-1154 °C; ^{1}H NMR (400 MHz, $CDCl_3$) δ ^{1}H NMR (400 MHz, $CDCl_3$) δ 7.86 – 7.81 (m, 2H), 7.64 (d, *J* = 8.5 Hz, 2H), 7.48 (brs, 2H), 7.42 (d, *J* = 8.0 Hz, 2H), 7.38 (brs, 1H), 7.20 (d, *J* = 8.0 Hz, 2H), 7.17 – 7.11 (m, 2H), 7.07 (td, *J* = 7.4, 1.1 Hz, 1H), 7.00 – 6.90 (m, 4H), 6.84 (s, 2H), 6.78 – 6.73 (m, 2H), 3.01 – 2.86 (m, 2H), 2.30 (s, 3H), 2.26 (s, 3H), 1.87 – 1.76 (m, 1H), 1.56 – 1.42 (m, 3H), 1.01 (t, *J* = 7.2 Hz, 3H), 0.86 (ddd, *J* = 13.3, 9.4, 6.3 Hz, 2H). ^{13}C NMR (101 MHz, $CDCl_3$) δ 148.08, 146.42, 144.48, 143.64, 140.54, 140.03, 137.01, 136.65, 136.58, 135.39, 134.71, 131.47, 130.97, 130.37, 129.77, 129.44, 129.09, 129.03, 128.78, 128.37, 128.05, 126.57, 126.47, 123.74, 123.01, 119.28, 116.07, 114.34, 31.15, 29.67, 28.22, 23.52, 22.01, 21.50, 21.46, 13.81. HRMS (ESI) calcd for $C_{40}H_{37}NO_4S_2Se$ [M+H]$^+$ 740.1429; found: 740.1410.

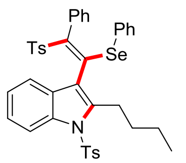

59

(*E*)-2-cyclopropyl-3-(2-phenyl-1-(phenylselanyl)-2-tosylvinyl)-1-tosylindole (60): The title compound was prepared according to the general procedure H via column chromatography of silica eluting hexane-ethyl acetate (83:17) to obtain as a pale yellow solid (52 mg, yield = 74%); Mp. 177-179 °C; ^{1}H NMR (400 MHz, $CDCl_3$) δ 7.96 (d, *J* = 8.3 Hz, 1H), 7.61 (d, *J* = 8.4 Hz, 2H), 7.55 – 7.36 (m, 4H), 7.26 (s, 1H), 7.24 – 7.18 (m, 4H), 7.12 (td, *J* = 7.5, 1.0 Hz, 1H), 7.09 – 7.04 (m, 2H), 6.99 (tt, *J* = 7.6, 1.3 Hz, 1H), 6.86 (ddd, *J* = 6.6, 2.3, 1.1 Hz, 4H), 6.73 (dd, *J* = 10.6, 4.9 Hz, 2H), 2.35 (s, 3H), 2.25 (s, 3H), 2.10 – 2.01 (m, 1H), 0.96 – 0.88 (m, 3H), 0.72 (dt, *J* = 8.9, 4.1 Hz, 1H). ^{13}C NMR (101 MHz, $CDCl_3$) δ 147.11, 144.37, 143.55, 139.25, 138.41, 137.02, 136.98, 136.92, 135.71, 134.65, 129.61, 129.50, 129.10, 128.80, 128.37, 128.21, 127.90, 126.64, 126.47, 124.25, 122.88, 119.37, 114.91, 114.10, 21.55, 21.44, 10.57, 7.46, 7.20. HRMS (ESI) calcd for $C_{39}H_{33}NO_4S_2Se$ [M+H]$^+$ 724.1097; found: 724.1093.

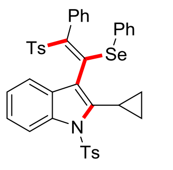

60

(*E*)-2-phenyl-3-(1-(phenylselanyl)-2-(p-tolyl)-2-tosylvinyl)-1-tosylindole (61): The title compound was prepared according to the general procedure H via column chromatography of silica eluting hexane-ethyl acetate (80:20) to obtain as a lite yellow solid (53 mg, yield = 69%); Mp. 131-132 °C; ^{1}H NMR (400 MHz, $CDCl_3$) δ 8.07 (d, *J* = 8.3 Hz, 1H), 7.42 (ddd, *J* = 23.7, 15.3, 7.7 Hz, 6H), 7.29 (ddd, *J* = 8.4, 6.4, 2.1 Hz, 1H), 7.18 (ddd, *J* = 17.5, 9.1, 7.9 Hz, 5H), 7.07 (d, *J* = 7.7 Hz, 3H), 7.01 (d, *J* = 29.3 Hz, 2H), 6.97 – 6.91 (m, 4H), 6.76 (t, *J* = 7.7 Hz, 2H), 6.57 (d, *J* = 6.9 Hz, 2H), 2.36 (d, *J* = 8.7 Hz, 6H), 2.20 (s, 3H). ^{13}C NMR (101 MHz, $CDCl_3$) δ 146.56, 144.43, 143.77, 140.16, 139.48, 137.51, 137.08, 136.81, 136.67, 134.93, 131.64, 131.10, 130.55, 130.23, 129.67, 129.33, 129.06, 128.85, 128.77, 128.64, 127.93, 127.10, 126.77, 125.91, 124.79, 123.97, 120.09, 119.75, 116.34, 21.55, 21.48. HRMS (ESI) calcd for $C_{43}H_{35}NO_4S_2Se$ $[M+H]^+$ 774.1254; found: 774.1260.

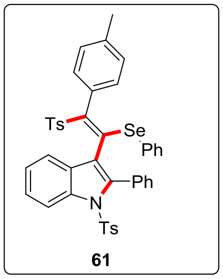

(E)-3-(2-(4-ethylphenyl)-1-(phenylselanyl)-2-tosylvinyl)-2-phenyl-1-tosylindole (62): The title compound was prepared according to the general procedure H via column chromatography of silica eluting hexane-ethyl acetate (80:20)to obtain as a white solid (50 mg, yield = 65%); ^{1}H NMR (400 MHz, $CDCl_3$) δ 8.07 (d, *J* = 8.3 Hz, 1H), 7.43 (d, *J* = 4.9 Hz, 2H), 7.37 (d, *J* = 8.4 Hz, 2H), 7.29 (td, *J* = 6.2, 3.2 Hz, 1H), 7.20 (t, *J* = 6.6 Hz, 2H), 7.16 (s, 2H), 7.08 (d, *J* = 8.7 Hz, 3H), 6.97 – 6.91 (m, 4H), 6.76 (t, *J* = 7.7 Hz, 2H), 6.57 (d, *J* = 7.0 Hz, 2H), 2.67 (q, *J* = 7.6 Hz, 2H), 2.35 (s, 3H), 2.21 (s, 3H), 1.25 (t, *J* = 7.6 Hz, 3H). ^{13}C NMR (101 MHz, $CDCl_3$) δ 146.53, 145.69, 144.44, 143.74, 140.24, 137.52, 137.10, 136.81, 136.67, 134.91, 131.83, 131.16, 130.57, 130.24, 129.34, 129.06, 128.82, 128.78, 128.63, 127.93, 127.11, 126.84, 125.92, 124.79, 123.98, 120.14, 119.76, 116.35, 28.70, 21.56, 21.49, 15.23. HRMS (ESI) calcd for $C_{44}H_{37}NO_4S_2Se$ $[M+H]^+$ 788.1412; found: 788.1411.

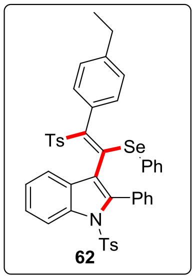

(*E*)-2-(4-methoxyphenyl)-3-(2-phenyl-1-(phenylselanyl)-2-tosylvinyl)-1-tosylindole (63): The title compound was prepared according to the general procedure H via column chromatography of silica eluting hexane-ethyl acetate (82:18) to obtain as a white solid (42 mg, yield = 53%); Mp. 141-142 °C; ^{1}H NMR (400 MHz, $CDCl_3$) δ 8.07 (d, *J* = 8.3 Hz, 1H), 7.47 – 7.39 (m, 3H), 7.36 (d, *J* = 8.4 Hz, 3H), 7.29 (ddd, *J* = 8.4, 6.7, 1.8 Hz, 1H), 7.25 – 7.20 (m, 2H), 7.17 (dd, *J* = 10.7, 4.2 Hz, 1H), 7.10 – 7.03 (m, 4H), 7.01 – 6.94 (m, 5H), 6.91 (d, *J* = 8.1 Hz, 1H), 6.83 (d, *J* = 7.4 Hz, 1H), 6.77 (t, *J* = 7.7 Hz, 2H), 6.58 (d, *J* = 6.9 Hz, 2H), 3.82 (s, 3H), 2.35 (s, 3H), 2.21 (s, 3H). ^{13}C NMR (101 MHz, $CDCl_3$) δ 160.32, 146.86, 144.43, 143.77, 139.74, 137.44, 137.03, 136.81, 136.65, 134.94, 132.70, 132.04, 130.61, 130.23, 129.33, 129.07, 128.89, 128.78, 128.61, 127.94, 127.07, 126.72, 126.61, 125.97, 124.82, 124.01, 120.13, 119.74, 116.34, 114.09, 55.23, 21.56, 21.49. HRMS (ESI) calcd for $C_{43}H_{35}NO_5S_2Se$ $[M+H]^+$ 790.1205; found: 790.1204.

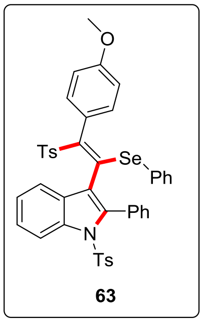

(*E*)-3-(2-(3-nitrophenyl)-1-(phenylselanyl)-2-tosylvinyl)-2-phenyl-1-tosylindole (64): The title compound was prepared according to the general procedure H via column chromatography of silica eluting hexane-ethyl acetate (75:25) to obtain as a yellow solid (21 mg, yield = 25%); Mp. 141-142 °C; ^{1}H NMR (400 MHz, $CDCl_3$) δ 8.23 (s, 1H), 8.11 (s, 1H), 7.98 (brs, 1H), 7.80 (dd, *J* = 9.5, 7.6 Hz, 1H), 7.61 (s, 1H), 7.47 (dt, *J* = 22.3, 19.0 Hz, 4H), 7.38 (d, *J* = 8.1 Hz, 2H), 7.34 – 7.31 (m, 1H), 7.24 – 7.19 (m, 2H), 7.08 (dd, *J* = 12.4, 4.9 Hz, 3H), 6.96 (dd, *J* = 30.8, 7.4 Hz, 4H), 6.79 (t, *J* = 7.7 Hz, 2H), 6.60 (s, 2H), 2.36 (s, 3H), 2.23 (s, 3H). ^{13}C NMR (101 MHz, $CDCl_3$) δ 147.90, 146.37, 144.63, 140.75, 138.50, 138.26, 137.81, 137.53, 136.61, 136.32, 135.21, 134.98, 130.39, 130.02, 129.40, 129.28, 129.02, 128.53, 128.24, 127.73, 127.12, 125.77, 125.02, 124.18, 124.09, 121.04, 119.60, 118.83, 116.39, 21.56, 21.50. HRMS (ESI) calcd for $C_{42}H_{32}N_2O_6S_2SeNa$ $[M+Na]^+$ 827.0867; found: 827.0871.

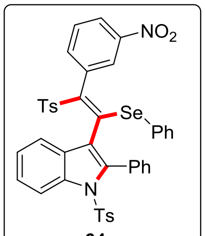

64

methyl (*E*)-4-(2-(2-phenyl-1-tosylindol-3-yl)-2-(phenylselanyl)-1-tosylvinyl)benzoate (65): The title compound was prepared according to the general procedure H via column chromatography of silica eluting hexane-ethyl acetate (70:30) to obtain as a white solid (27 mg, yield = 33%); Mp. 123-124 °C; ^{1}H NMR (400 MHz, $CDCl_3$) δ 8.10 (d, *J* = 8.3 Hz, 1H), 8.05 (d, *J* = 7.1 Hz, 1H), 7.98 (d, *J* = 7.1 Hz, 1H), 7.50 – 7.40 (m, 4H), 7.37 (d, *J* = 8.4 Hz, 2H), 7.31 (ddd, *J* = 8.5, 6.2, 2.3 Hz, 2H), 7.24 – 7.21 (m, 2H), 7.20 – 7.16 (m, 1H), 7.09 (t, *J* = 7.2 Hz, 3H), 6.96 (q, *J* = 8.4 Hz, 5H), 6.78 (t, *J* = 7.7 Hz, 2H), 6.58 (d, *J* = 6.7 Hz, 2H), 3.93 (s, 3H), 2.36 (s, 3H), 2.22 (s, 3H). ^{13}C NMR (101 MHz, $CDCl_3$) δ 166.51, 146.99, 144.52, 144.21, 139.51, 139.27, 137.58, 136.81, 136.63, 130.82, 130.37, 130.15, 129.36, 129.27, 129.06, 128.93, 128.61, 128.09, 127.09, 126.84, 125.58, 124.93, 124.05, 119.62, 116.40, 52.31, 21.59, 21.50. HRMS (ESI) calcd for $C_{44}H_{35}NO_6S_2SeNa$ $[M+Na]^+$ 840.0966; found: 840.0970.

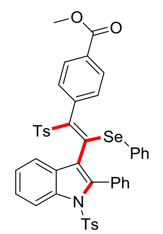

65

(*E*)-1-(4-(2-(2-phenyl-1-tosylindol-3-yl)-2-(phenylselanyl)-1-tosylvinyl)phenyl)ethan-1-one (66): The title compound was prepared according to the general procedure H via column chromatography of silica eluting hexane-ethyl acetate (70:30) to obtain as a white solid (29 mg, yield = 36%); Mp. 118-119 °C; ^{1}H NMR (400 MHz, $CDCl_3$) δ 8.07 (d, *J* = 8.3 Hz, 1H), 7.91 (d, *J* = 18.3 Hz, 2H), 7.47 – 7.40 (m, 3H), 7.35 (d, *J* = 8.4 Hz, 3H), 7.29 (dt, *J* = 8.4, 4.3 Hz, 2H), 7.21 – 7.16 (m, 3H), 7.08 – 7.03 (m, 4H), 6.92 (ddd, *J* = 9.9, 7.6, 1.3 Hz, 5H), 6.75 (t, *J* = 7.8 Hz, 2H), 6.55 (dd, *J* = 8.2, 1.2 Hz, 2H), 2.60 (s, 3H), 2.33 (s, 3H), 2.19 (s, 3H). ^{13}C NMR (101 MHz, $CDCl_3$) δ 197.52, 144.55, 144.25, 139.48, 137.62, 137.37, 136.79, 136.71, 136.62, 134.91, 131.74, 130.28, 130.15, 129.37, 129.30, 129.08, 128.94, 128.78, 128.57, 128.10, 127.11, 126.86, 125.53, 124.92, 124.03, 119.60, 116.40, 26.70, 21.58, 21.50. HRMS (ESI) calcd for $C_{44}H_{35}NO_5S_2Se$ $[M+H]^+$ 803.1201; found: 803.1206.

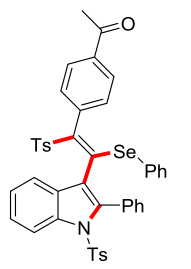

66

(*E*)-2-phenyl-3-(2-phenyl-1-(phenylselanyl)-2-(phenylsulfonyl)vinyl)-1-tosylindole (67): The title compound

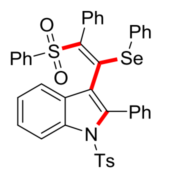

67

was prepared according to the general procedure H via column chromatography of silica eluting hexane-ethyl acetate (85:15) to obtain as a white solid (53 mg, yield = 69%); Mp. 186-187 °C; ^{1}H NMR (400 MHz, $CDCl_3$) δ 8.30 (d, *J* = 9.0 Hz, 1H), 7.94 (dd, *J* = 8.5, 1.2 Hz, 1H), 7.84 – 7.78 (m, 1H), 7.78 – 7.68 (m, 1H), 7.66 – 7.59 (m, 2H), 7.58 – 7.52 (m, 3H), 7.47 – 7.40 (m, 5H), 7.40 – 7.32 (m, 2H), 7.31 – 7.27 (m, 1H), 7.24 – 7.20 (m, 2H), 7.20 – 7.14 (m, 3H), 7.10 (d, *J* = 8.0 Hz, 2H), 7.08 – 7.04 (m, 2H), 6.89 (d, *J* = 7.4 Hz, 1H), 6.80 (d, *J* = 7.7 Hz, 1H), 2.26 (s, 3H). ^{13}C NMR (101 MHz, $CDCl_3$) δ 151.07, 144.72, 138.67, 138.63, 137.15, 136.35, 136.27, 135.12, 133.37, 131.43, 131.14, 131.09, 130.20, 130.11, 130.01, 129.66, 129.43, 129.19, 129.10, 128.54, 128.39, 128.35, 128.31, 128.14, 127.96, 127.45, 126.81, 126.04, 125.51, 124.55, 119.30, 116.53, 108.83, 21.53. HRMS (ESI) calcd for $C_{41}H_{32}NO_4S_2Se$ $[M+H]^+$ 747.0934; found: 747.0936.

(*E*)-3-(2-((4-methoxyphenyl)sulfonyl)-2-phenyl-1-(phenylselanyl)vinyl)-2-phenyl-1-tosylindole (68): The

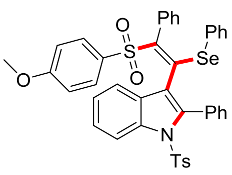

68

title compound was prepared according to the general procedure H via column chromatography of silica eluting hexane-ethyl acetate (82:18) to obtain as a white solid (34 mg, yield = 43%); Mp. 100-101 °C; ^{1}H NMR (400 MHz, $CDCl_3$) δ 8.08 (d, *J* = 8.3 Hz, 1H), 7.49 – 7.35 (m, 8H), 7.34 – 7.19 (m, 6H), 7.13 – 7.04 (m, 4H), 7.02 – 6.97 (m, 2H), 6.79 – 6.73 (m, 2H), 6.63 (d, *J* = 9.0 Hz, 2H), 6.58 (dd, *J* = 8.2, 1.2 Hz, 2H), 3.80 (s, 3H), 2.20 (s, 3H). ^{13}C NMR (101 MHz, $CDCl_3$) δ 163.18, 145.78, 144.43, 140.48, 137.47, 136.80, 136.65, 134.94, 134.70, 131.48, 131.25, 130.74, 130.64, 130.56, 130.21, 129.35, 129.32, 129.07, 128.86, 128.79, 128.34, 127.94, 127.06, 126.72, 125.82, 124.84, 124.03, 120.06, 119.71, 116.33, 113.42, 55.52, 21.47. HRMS (ESI) calcd for $C_{42}H_{34}NO_5S_2Se$ $[M+H]^+$ 776.0965; found: 776.1046.

(*E*)-3-(2-((4-chlorophenyl)sulfonyl)-2-phenyl-1-(phenylselanyl)vinyl)-2-phenyl-1-tosylindole (69): T The

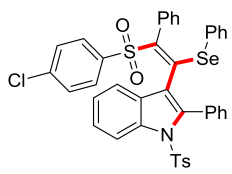

69

title compound was prepared according to the general procedure H via column chromatography of silica eluting hexane-ethyl acetate (83:17) to obtain as a white solid (37 mg, yield = 48%); Mp. 213-214 °C; ^{1}H NMR (400 MHz, $CDCl_3$) δ ^{1}H NMR (400 MHz, , $CDCl_3$) δ 8.12 (d, *J* = 8.4 Hz, 1H), 7.47 (ddd, *J* = 16.2, 10.3, 3.3 Hz, 5H), 7.41 – 7.37 (m, 3H), 7.33 (ddd, *J* = 8.4, 7.0, 1.6 Hz, 2H), 7.29 (s, 1H), 7.26 – 7.17 (m, 3H), 7.16 – 7.07 (m, 6H), 6.95 (d, *J* = 8.7 Hz, 2H), 6.78 (t, *J* = 7.8 Hz, 2H), 6.59 (dd, *J* = 8.2, 1.2 Hz, 2H), 2.23 (s, 3H). ^{13}C NMR (101 MHz, $CDCl_3$) δ ^{13}C NMR (101 MHz, $cdcl_3$) δ 148.29, 144.55, 139.63, 139.42, 138.43, 136.75, 136.62, 134.90, 134.29, 131.32, 130.25, 130.11, 129.93, 129.64, 129.35, 129.23, 129.09, 128.92, 128.51, 128.04, 127.16, 126.82, 125.66, 124.98, 124.11, 119.69, 119.38, 116.46, 21.49. HRMS (ESI) calcd for $C_{41}H_{31}NO_4S_2SeCl$ $[M+H]^+$ 781.0548; found: 781.0550.

(*E*)-3-(2-((4-fluorophenyl)sulfonyl)-2-phenyl-1-(phenylselanyl)vinyl)-2-phenyl-1-tosylindole (70): The title

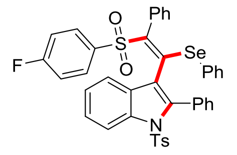
70

compound was prepared according to the general procedure H via column chromatography of silica eluting hexane-ethyl acetate (83:17) to obtain as a white solid (35 mg, yield = 39%); ^{1}H NMR (400 MHz, $CDCl_3$) δ 8.10 (d, *J* = 8.3 Hz, 1H), 7.51 – 7.40 (m, 5H), 7.40 – 7.35 (m, 3H), 7.32 (ddd, *J* = 8.4, 6.0, 2.5 Hz, 2H), 7.26 (s, 1H), 7.25 (d, *J* = 0.9 Hz, 1H), 7.24 – 7.19 (m, 2H), 7.13 – 7.01 (m, 6H), 6.83 (t, *J* = 8.6 Hz, 2H), 6.77 (t, *J* = 7.8 Hz, 2H), 6.61 – 6.55 (m, 2H), 2.22 (s, 3H). ^{13}C NMR (101 MHz, $CDCl_3$) δ 166.59, 164.05, 147.69, 144.53, 139.63, 137.60, 136.81, 136.66, 135.98, 134.96, 134.40, 131.37, 131.27, 129.60, 129.35, 129.23, 128.92, 128.05, 127.16, 125.00, 124.12, 119.78, 119.44, 116.49, 115.61, 115.38, 21.50. HRMS (ESI) calcd for $C_{41}H_{30}NO_4S_2SeF$ $[M+H]^+$ 764.0847; found: 764.0851.

(*E*)-2-phenyl-3-(2-phenyl-1-(phenylselanyl)-2-(thiophen-2-ylsulfonyl)vinyl)-1-tosylindole (71): The title

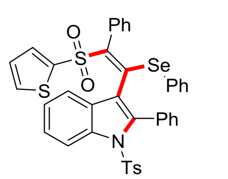
71

compound was prepared according to the general procedure H via column chromatography of silica eluting hexane-ethyl acetate (80:20) to obtain as a lite yellow solid (15 mg, yield = 20%); Mp. 114-115 °C; ^{1}H NMR (400 MHz, $CDCl_3$) δ 8.10 – 8.05 (m, 1H), 7.53 (dd, *J* = 4.9, 1.4 Hz, 1H), 7.47 – 7.33 (m, 8H), 7.32 – 7.27 (m, 5H), 7.17 (d, *J* = 6.3 Hz, 1H), 7.12 – 7.09 (m, 1H), 7.06 (dd, *J* = 10.3, 7.8 Hz, 3H), 6.83 (dd, *J* = 4.8, 3.8 Hz, 1H), 6.82 – 6.76 (m, 3H), 6.61 (dd, *J* = 8.2, 1.2 Hz, 2H), 2.22 (s, 3H). ^{13}C NMR (101 MHz, $CDCl_3$) δ 146.76, 144.41, 140.96, 139.63, 137.27, 136.88, 136.58, 134.92, 134.55, 134.34, 133.75, 131.15, 130.80, 130.61, 130.19, 129.57, 129.34, 129.17, 128.88, 128.57, 128.01, 127.13, 126.95, 126.80, 125.83, 124.99, 124.25, 120.05, 119.69, 116.34, 21.50. HRMS (ESI) calcd for $C_{39}H_{29}NO_4S_3Se$ $[M+H]^+$ 752.0504; found: 752.0511.

(*E*)-2-phenyl-3-(2-phenyl-1-(p-tolylselanyl)-2-tosylvinyl)-1-tosylindole (72): The title compound was

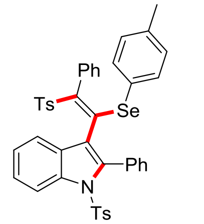
72

prepared according to the general procedure H via column chromatography of silica eluting hexane-ethyl acetate (84:16) to obtain as a lite yellow solid (46 mg, yield = 59%) Mp. 104-105 °C; ^{1}H NMR (400 MHz, $CDCl_3$) δ 8.08 (d, *J* = 8.3 Hz, 1H), 7.43 (ddd, *J* = 23.6, 16.0, 8.6 Hz, 8H), 7.34 – 7.27 (m, 3H), 7.19 (dd, *J* = 12.3, 4.7 Hz, 3H), 7.09 (t, *J* = 8.8 Hz, 3H), 6.98 – 6.92 (m, 4H), 6.57 (d, *J* = 7.8 Hz, 2H), 6.45 (d, *J* = 8.0 Hz, 2H), 2.35 (s, 3H), 2.21 (s, 3H), 2.17 (s, 3H). ^{13}C NMR (101 MHz, $CDCl_3$) δ 146.90, 144.44, 143.82, 140.05, 139.29, 137.44, 137.01, 136.85, 136.61, 134.96, 134.71, 131.34, 130.69, 130.54, 130.31, 130.24, 129.34, 129.00, 128.87, 128.77, 128.64, 128.33, 127.11, 126.84, 124.75, 123.97, 122.36, 120.19, 119.77, 116.32, 21.56, 21.50, 21.17. HRMS (ESI) calcd for $C_{43}H_{35}NO_4S_2Se$ $[M+H]^+$ 774.1254; found: 774.1259.

(*E*)-3-(1-((4-fluorophenyl)selanyl)-2-phenyl-2-tosylvinyl)-2-phenyl-1-tosylindole (73): The title compound

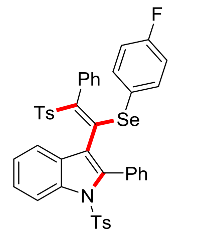
73

was prepared according to the general procedure H via column chromatography of silica eluting hexane-ethyl acetate (84:16) to obtain as a lite yellow solid (43 mg, yield = 59%) Mp. 180-181 °C; ^{1}H NMR (400 MHz, $CDCl_3$) δ 8.11 (d, *J* = 8.4 Hz, 1H), 7.54 – 7.40 (m, 4H),

7.38 (dd, J = 6.7, 1.7 Hz, 4H), 7.31 (ddd, J = 8.4, 6.2, 2.3 Hz, 3H), 7.22 (td, J = 6.2, 0.8 Hz, 3H), 7.08 (d, J = 8.0 Hz, 3H), 6.99 – 6.91 (m, 4H), 6.59 – 6.53 (m, 2H), 6.45 (ddd, J = 10.6, 5.9, 2.5 Hz, 2H), 2.34 (s, 3H), 2.21 (s, 3H). ^{13}C NMR (101 MHz, $CDCl_3$) δ 164.42, 161.93, 145.98, 144.56, 143.95, 140.49, 138.79, 138.70, 137.50, 136.82, 136.78, 134.87, 134.46, 131.19, 130.58, 130.34, 130.24, 129.44, 129.35, 128.90, 128.61, 128.37, 127.09, 126.86, 125.00, 124.08, 120.78, 120.74, 119.89, 119.56, 116.45, 115.28, 115.06, 109.94, 21.54, 21.47. HRMS (ESI) calcd for $C_{42}H_{33}FNO_4S_2Se$ $[M+H]^+$ 778.0995; found: 778.0999.

(*E*)-3-(1-((4-chlorophenyl)selanyl)-2-phenyl-2-tosylvinyl)-2-phenyl-1-tosylindole (74): The title compound

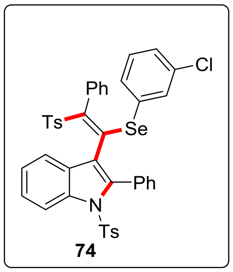

was prepared according to the general procedure H via column chromatography of silica eluting hexane-ethyl acetate (84:16) to obtain as a lite yellow solid (42 mg, yield = 52%) Mp. 97-99 °C; ^{1}H NMR (400 MHz, $CDCl_3$) δ 8.12 (d, J = 8.3 Hz, 1H), 7.46 (d, J = 2.7 Hz, 3H), 7.42 – 7.36 (m, 4H), 7.32 (dt, J = 8.4, 4.3 Hz, 2H), 7.26 – 7.22 (m, 3H), 7.15 – 7.03 (m, 5H), 7.00 – 6.90 (m, 5H), 6.66 (t, J = 7.9 Hz, 1H), 6.60 – 6.57 (m, 1H), 6.42 – 6.38 (m, 1H), 2.35 (s, 3H), 2.20 (s, 3H). ^{13}C NMR (101 MHz, $CDCl_3$) δ 145.68, 144.51, 144.02, 140.73, 137.65, 136.80, 136.73, 136.56, 134.80, 134.42, 134.37, 133.35, 131.17, 130.53, 130.48, 130.12, 129.51, 129.42, 129.34, 129.28, 128.92, 128.83, 128.63, 128.42, 127.39, 127.05, 126.94, 125.07, 124.24, 119.87, 119.47, 116.54, 21.54, 21.47. HRMS (ESI) calcd for $C_{42}H_{33}NO_4S_2SeCl$ $[M+H]^+$ 795.1254; found: 795.1253.

(*E*)-3-(1-(pentylselanyl)-2-phenyl-2-tosylvinyl)-2-phenyl-1-tosylindole (75): The title compound was

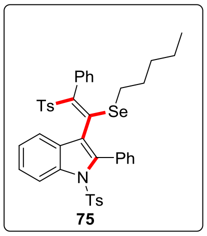

prepared according to the general procedure H via column chromatography of silica eluting hexane-ethyl acetate (84:16) to obtain as a lite yellow solid (13 mg, yield = 18%) Mp. 130-131 °C; ^{1}H NMR (400 MHz, $CDCl_3$) δ 8.35 (d, J = 8.4 Hz, 1H), 7.77 (brs, 2H), 7.55 – 7.39 (m, 7H), 7.36 – 7.26 (m, 4H), 7.14 – 7.06 (m, 3H), 7.01 (brs, 1H), 6.91 (d, J = 8.1 Hz, 2H), 6.86 (d, J = 8.4 Hz, 2H), 2.30 (s, 3H), 2.26 (s, 3H), 1.79 (dt, J = 11.2, 7.3 Hz, 1H), 1.68 – 1.62 (m, 1H), 1.05 – 0.88 (m, 4H), 0.87 – 0.73 (m, 2H), 0.66 (t, J = 7.2 Hz, 3H). ^{13}C NMR (101 MHz, $CDCl_3$) δ 144.62, 143.53, 138.80, 137.18, 136.91, 136.87, 135.04, 134.73, 132.65, 130.78, 129.76, 129.70, 129.36, 129.07, 128.93, 128.80, 128.41, 128.14, 127.30, 127.19, 127.12, 126.72, 125.41, 124.55, 120.06, 119.48, 116.78, 31.59, 28.33, 27.33, 21.94, 21.53, 21.50, 13.67. HRMS (ESI) calcd for $C_{41}H_{39}NO_4S_2Se$ $[M+H]^+$ 755.1254; found: 755.1260.

(*E*/*Z*)-2-phenyl-3-(2-phenyl-2-(phenylthio)vinyl)-1-tosylindole (76): The title compound was prepared

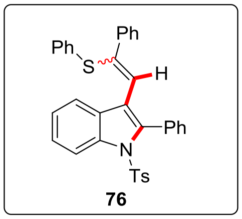

according to the general procedure I via column chromatography of silica eluting hexane-ethyl acetate (90:10) in to obtain mixture two isomers (*E*/*Z*= 44:66) as a yellow solid (31 mg, yield = 55%); Mp. 138.4-139.5 °C; ^{1}H NMR (400 MHz, $CDCl_3$) δ 8.32 (d, J = 8.4 Hz, 1H), 8.23 (d, J = 8.4 Hz, 1H), 7.51 – 7.45 (m, 3H), 7.45 – 7.37 (m, 8H), 7.36 – 7.32 (m, 2H), 7.27 (ddd, J = 7.2, 5.8, 1.9 Hz, 7H), 7.24 – 7.12 (m, 6H), 7.10 (d, J = 8.2 Hz, 2H), 6.99 (dddd, J = 16.1, 12.7, 6.8, 2.0 Hz, 9H), 6.91 – 6.80 (m, 4H), 6.76 (d, J = 7.9 Hz, 1H), 6.65 (s, 1H), 6.39 (s, 1H), 2.35 (s, 2H), 2.22 (s, 3H). ^{13}C NMR (101 MHz, $CDCl_3$) δ 144.45, 144.43, 140.38, 139.40, 139.11, 138.83, 138.77, 137.90,

137.66, 137.56, 134.93, 134.61, 134.55, 134.14, 131.49, 131.44, 131.34, 131.26, 130.05, 129.41, 129.37, 129.15, 129.10, 128.97, 128.88, 128.65, 128.57, 128.41, 128.11, 127.96, 127.84, 127.77, 127.35, 127.32, 127.09, 126.90, 126.76, 125.74, 125.10, 125.00, 124.85, 124.11, 124.09, 122.61, 122.19, 121.82, 120.84, 120.64, 116.76, 21.57, 21.47. HRMS (ESI) calcd for $C_{35}H_{28}NO_2S_2$ $[M+H]^+$ 558.1561; found: 558.1569.

(*Z*)-5-chloro-2-phenyl-3-(2-phenyl-2-(phenylthio)vinyl)-1-tosylindole (77): The title compound was prepared according to the general procedure I via column chromatography of silica eluting hexane-ethyl acetate (88:12) to obtain single isomers (*Z*) as a white solid (25 mg, yield = 42%); Mp. 138.4-139.5 °C; ^{1}H NMR (400 MHz, $CDCl_3$) δ 8.24 (d, *J* = 8.9 Hz, 1H), 7.50 – 7.44 (m, 3H), 7.43 – 7.39 (m, 5H), 7.32 (dd, *J* = 8.9, 2.2 Hz, 1H), 7.25 – 7.22 (m, 2H), 7.21 – 7.17 (m, 3H), 7.05 – 6.98 (m, 5H), 6.88 – 6.85 (m, 2H), 6.59 (s, 1H), 2.26 (s, 3H). ^{13}C NMR (101 MHz, $CDCl_3$) δ 144.78, 141.07, 140.04, 138.97, 135.92, 134.64, 134.38, 131.33, 131.24, 130.97, 129.93, 129.39, 129.30, 128.94, 128.46, 128.29, 128.16, 127.97, 127.42, 126.76, 125.91, 125.10, 124.08, 121.37, 120.53, 117.73, 21.52. HRMS (ESI) calcd for $C_{35}H_{27}NO_2S_2Cl$ $[M+H]^+$ 592.1172; found: 592.1168.

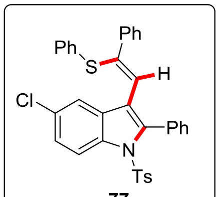

77

(*Z*)-5-bromo-2-phenyl-3-(2-phenyl-2-(phenylthio)vinyl)-1-tosylindole (78): The title compound was prepared according to the general procedure I via column chromatography of silica eluting hexane-ethyl acetate (88:12) to obtain mixture two isomers (*E*/*Z*= 10:90) as a white solid (34 mg, yield = 47%); Mp. 138.4-139.5 °C; ^{1}H NMR (400 MHz, $CDCl_3$) δ 8.18 (d, *J* = 8.9 Hz, 1H), 7.60 (d, *J* = 1.9 Hz, 1H), 7.49 – 7.43 (m, 3H), 7.42 – 7.37 (m, 5H), 7.25 – 7.22 (m, 2H), 7.19 (dd, *J* = 5.1, 1.9 Hz, 3H), 7.05 – 6.98 (m, 5H), 6.88 – 6.85 (m, 2H), 6.58 (s, 1H), 2.27 (s, 3H). ^{13}C NMR (101 MHz, $CDCl_3$) δ 144.80, 141.01, 139.90, 138.97, 136.29, 134.66, 134.41, 131.98, 131.59, 131.35, 130.90, 129.32, 129.26, 128.95, 128.91, 128.48, 128.31, 128.17, 127.96, 127.76, 127.42, 126.88, 126.76, 125.90, 124.10, 123.62, 121.21, 118.05, 117.66, 21.52. HRMS (ESI) calcd for $C_{35}H_{27}NO_2S_2Br$ $[M+H]^+$ 636.0667; found: 636.0670.

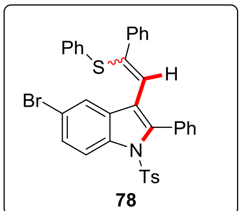

78

(*E*/*Z*)-3-(2-phenyl-2-(phenylthio)vinyl)-2-(p-tolyl)-1-tosylindole (79): The title compound was prepared according to the general procedure I via column chromatography of silica eluting hexane-ethyl acetate (89:11) to obtain mixture two isomers (*E*/*Z*= 47:53) as a yellow solid (28 mg, yield = 50%); Mp. 136-137 °C; ^{1}H NMR (400 MHz, $CDCl_3$) δ 8.31 (d, *J* = 8.3 Hz, 1H), 8.22 (d, *J* = 8.3 Hz, 1H), 7.51 – 7.45 (m, 3H), 7.38 – 7.35 (m, 1H), 7.34 – 7.31 (m, 2H), 7.30 – 7.24 (m, 9H), 7.23 – 7.14 (m, 9H), 7.13 – 7.08 (m, 3H), 7.07 – 7.01 (m, 3H), 7.01 – 6.96 (m, 4H), 6.96 – 6.90 (m, 1H), 6.89 – 6.86 (m, 2H), 6.85 – 6.79 (m, 2H), 6.70 (ddd, *J* = 7.9, 1.2, 0.7 Hz, 1H), 6.65 (s, 1H), 6.44 (s, 1H), 2.46 (s, 3H), 2.41 (s, 3H), 2.35 (s, 3H), 2.22 (s, 3H). ^{13}C NMR (101 MHz, $CDCl_3$) δ 144.39, 144.37, 139.96, 139.31, 139.17, 139.07, 138.66, 138.59, 137.91, 137.64, 135.06, 134.57, 134.49, 134.45, 131.20, 131.13, 131.11, 130.15, 129.38, 129.26, 129.11, 129.07, 129.01, 128.82, 128.58, 128.37, 128.13, 128.10, 128.06, 127.95, 127.79, 127.72, 126.92, 126.90, 126.76, 125.66, 125.47, 124.85, 124.69, 124.08, 122.80, 122.40, 121.95, 120.79,

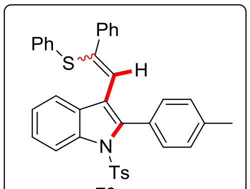

79

120.62, 116.81, 77.31, 76.99, 76.67, 21.57, 21.52, 21.48, 21.46. HRMS (ESI) calcd for $C_{36}H_{30}NO_2S_2$ $[M+H]^+$ 572.1718; found: 572.1713.

(*E*/*Z*)-2-phenyl-3-(2-(phenylthio)-2-(p-tolyl)vinyl)-1-tosylindole (80): The title compound was prepared

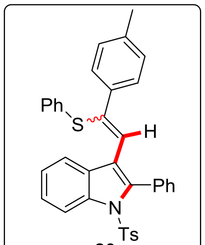

80

according to the general procedure I via column chromatography of silica eluting hexane-ethyl acetate (90:10) to obtain mixture two isomers (*E*/*Z*= 17:83) as a yellow solid (25 mg, yield = 44%); Mp. 173-174 °C; ^{1}H NMR (400 MHz, $CDCl_3$) δ 8.31 (d, *J* = 8.3 Hz, 1H), 7.47 (d, *J* = 7.4 Hz, 1H), 7.41 (dd, *J* = 4.3, 3.4 Hz, 2H), 7.40 – 7.36 (m, 5H), 7.28 – 7.23 (m, 4H), 7.01 (dd, *J* = 10.4, 8.1 Hz, 7H), 6.87 (d, *J* = 6.8 Hz, 2H), 6.62 (s, 1H), 2.25 (s, 3H), 2.23 (s, 3H). ^{13}C NMR (101 MHz, $CDCl_3$) δ 144.42, 140.05, 138.72, 138.09, 137.66, 136.24, 135.23, 134.52, 131.52, 131.33, 131.25, 130.12, 129.14, 129.13, 128.86, 128.60, 128.51, 128.40, 127.78, 127.34, 127.28, 126.91, 126.76, 125.58, 124.97, 124.52, 124.08, 122.34, 120.87, 116.76, 21.48, 21.09. HRMS (ESI) calcd for $C_{36}H_{30}NO_2S_2$ $[M+H]^+$ 572.1718; 572.1714.

(*E*/*Z*)-2-phenyl-3-(2-phenyl-2-(p-tolylthio)vinyl)-1-tosylindole (81): The title compound was prepared

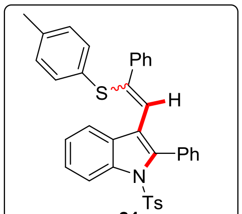

81

according to the general procedure I via column chromatography of silica eluting hexane-ethyl acetate (90:10) to obtain mixture two isomers (*E*/*Z*= 13:87) as a white solid (26 mg, yield = 47%); Mp. 178-179 °C; ^{1}H NMR (400 MHz, $CDCl_3$) δ 8.32 (d, *J* = 8.3 Hz, 1H), 7.48 (ddd, *J* = 7.8, 1.3, 0.7 Hz, 1H), 7.47 – 7.42 (m, 4H), 7.41 – 7.38 (m, 4H), 7.37 – 7.34 (m, 1H), 7.29 – 7.24 (m, 4H), 7.18 – 7.15 (m, 2H), 7.02 – 6.99 (m, 2H), 6.83 (d, *J* = 8.0 Hz, 2H), 6.78 (d, *J* = 8.3 Hz, 2H), 6.59 (s, 1H), 2.22 (s, 3H), 2.18 (s, 3H). ^{13}C NMR (101 MHz, $CDCl_3$) δ 144.44, 141.05, 139.24, 138.70, 137.71, 135.71, 134.58, 132.23, 131.55, 131.35, 131.28, 131.16, 130.17, 129.77, 129.71, 129.24, 129.16, 129.09, 128.91, 128.61, 128.45, 128.06, 128.03, 128.01, 127.77, 127.34, 127.28, 126.92, 126.77, 124.98, 124.81, 124.49, 124.11, 122.33, 120.86, 120.65, 120.23, 116.80, 21.47, 20.92. HRMS (ESI) calcd for $C_{36}H_{30}NO_2S_2$ $[M+H]^+$ 572.1718; found: 572.1716.

(*E*/*Z*)-3-(2-((3-methoxyphenyl)thio)-2-phenylvinyl)-2-phenyl-1-tosylindole (82): The title compound was

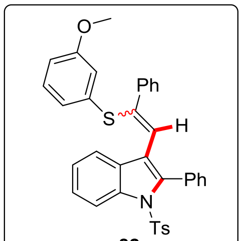

82

prepared according to the general procedure I via column chromatography of silica eluting hexane-ethyl acetate (92:8) to obtain mixture two isomers (*E*/*Z*= 27:73) as a yellow solid (23 mg, yield = 38%); Mp. 81-82 °C; ^{1}H NMR (400 MHz, $CDCl_3$) δ 8.32 (d, *J* = 8.4 Hz, 1H), 8.23 (d, *J* = 8.4 Hz, 1H), 7.52 – 7.46 (m, 3H), 7.45 – 7.33 (m, 8H), 7.29 – 7.23 (m, 4H), 7.22 – 7.17 (m, 3H), 7.09 (dd, *J* = 8.0, 5.5 Hz, 1H), 7.06 – 7.02 (m, 1H), 6.99 (d, *J* = 7.9 Hz, 2H), 6.97 – 6.92 (m, 1H), 6.88 – 6.80 (m, 2H), 6.76 (d, *J* = 7.8 Hz, 1H), 6.66 (s, 1H), 6.55 – 6.47 (m, 2H), 6.44 – 6.40 (m, 1H), 3.68 (s, 1H), 3.59 (s, 3H), 2.35 (s, 1H), 2.23 (s, 3H). ^{13}C NMR (101 MHz, $CDCl_3$) δ 159.68, 159.36, 144.47, 140.07, 139.27, 138.90, 138.83, 137.66, 136.30, 135.60, 134.62, 134.56, 131.46, 131.35, 131.26, 130.01, 129.63, 129.36, 129.19, 129.16, 129.10, 128.98, 128.67, 128.60, 128.19, 128.17, 127.86, 127.80, 127.34, 126.92, 126.75, 125.53, 125.00, 124.88, 124.11, 123.26, 122.72, 122.57, 122.11, 121.45, 120.85, 120.65,

116.78, 116.75, 116.12, 114.27, 112.99, 111.88, 55.14, 55.04, 21.57, 21.46. HRMS (ESI) calcd for $C_{36}H_{30}NO_3S_2$ [M+H]$^+$ 588.1667; found: 588.1660.

(*E*/*Z*)-3-(2-((4-methoxyphenyl)thio)-2-phenylvinyl)-2-phenyl-1-tosylindole (83): The title compound was

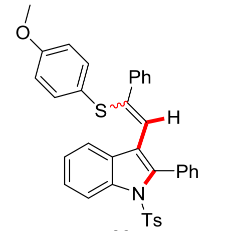

83

prepared according to the general procedure I via column chromatography of silica eluting hexane-ethyl acetate (92:8) to obtain mixture two isomers (*E*/*Z*= 48:52) as a yellow solid (25 mg, yield = 42%); Mp. 86-88 °C; ^{1}H NMR (400 MHz, $CDCl_3$) δ 8.33 (d, *J* = 8.3 Hz, 1H), 8.21 (d, *J* = 8.3 Hz, 1H), 7.51 (dd, *J* = 7.8, 0.6 Hz, 1H), 7.47 – 7.36 (m, 12H), 7.33 – 7.26 (m, 8H), 7.25 – 7.15 (m, 6H), 7.09 (d, *J* = 8.0 Hz, 2H), 7.04 – 6.98 (m, 5H), 6.97 – 6.93 (m, 1H), 6.89 – 6.84 (m, 2H), 6.83 – 6.79 (m, 2H), 6.76 – 6.72 (m, 3H), 6.56 (d, *J* = 8.9 Hz, 2H), 6.52 (s, 1H), 6.03 (s, 1H), 3.74 (s, 3H), 3.68 (s, 3H), 2.35 (s, 3H), 2.23 (s, 3H). ^{13}C NMR (101 MHz, $CDCl_3$) δ 159.58, 158.30, 144.46, 144.36, 142.07, 141.40, 139.29, 138.49, 138.32, 137.88, 137.67, 137.61, 135.10, 134.60, 134.56, 132.14, 131.56, 131.35, 131.27, 130.26, 129.62, 129.16, 129.07, 128.76, 128.58, 128.35, 128.17, 127.98, 127.94, 127.83, 127.79, 127.32, 127.23, 126.90, 126.78, 124.99, 124.75, 124.09, 124.00, 123.53, 123.23, 122.78, 122.27, 120.80, 120.64, 117.98, 116.78, 116.72, 114.67, 114.09, 55.24, 55.14, 21.57, 21.48. HRMS (ESI) calcd for $C_{36}H_{30}NO_3S_2$ [M+H]$^+$ 588.1667; found: 588.1661.

(*E*/*Z*)-3-(2-((2-bromophenyl)thio)-2-phenylvinyl)-2-phenyl-1-tosylindole (84): The title compound was

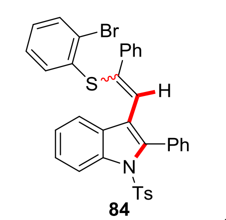

84

prepared according to the general procedure I via column chromatography of silica eluting hexane-ethyl acetate (90:10) to obtain mixture two isomers (*E*/*Z*= 14:86) as a white solid (32 mg, yield = 50%); Mp. 175-176 °C; ^{1}H NMR (400 MHz, $CDCl_3$) δ 8.32 (d, *J* = 8.4 Hz, 1H), 7.50 – 7.35 (m, 11H), 7.27 – 7.18 (m, 7H), 7.02 (d, *J* = 8.1 Hz, 2H), 6.86 (ddd, *J* = 13.6, 7.5, 1.7 Hz, 2H), 6.77 (s, 1H), 6.65 (dd, *J* = 7.7, 1.8 Hz, 1H), 2.26 (s, 3H). ^{13}C NMR (101 MHz, $CDCl_3$) δ 144.56, 139.48, 138.93, 138.66, 137.72, 136.44, 134.55, 132.55, 131.42, 131.39, 131.30, 130.40, 130.00, 129.20, 129.13, 129.03, 128.69, 128.33, 128.28, 127.98, 127.85, 127.70, 127.36, 127.15, 126.93, 126.71, 126.66, 125.07, 124.23, 122.96, 121.93, 120.60, 116.80, 21.57. HRMS (ESI) calcd for $C_{35}H_{26}NO_2NaS_2Br$ [M+Na]$^+$ 658.0486; found: 658.0490.

(*E*/*Z*)-3-(2-((4-bromophenyl)thio)-2-phenylvinyl)-2-phenyl-1-tosylindole (85): The title compound was

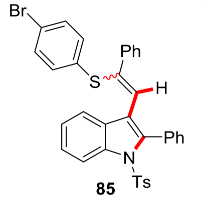

85

prepared according to the general procedure I via column chromatography of silica eluting hexane-ethyl acetate (90:10) to obtain mixture two isomers (*E*/*Z*= 27:73) as a white solid (34 mg, yield = 53%); Mp. 180.4-181 °C; ^{1}H NMR (400 MHz, $CDCl_3$) δ 8.34 (d, *J* = 8.4 Hz, 1H), 7.46 – 7.42 (m, 3H), 7.40 – 7.37 (m, 5H), 7.28 – 7.25 (m, 3H), 7.21 – 7.18 (m, 3H), 7.13 – 7.09 (m, 3H), 7.02 (dd, *J* = 8.6, 0.6 Hz, 2H), 6.68 (d, *J* = 8.1 Hz, 3H), 2.25 (s, 3H). ^{13}C NMR (101 MHz, $CDCl_3$) δ 144.52, 139.56, 138.75, 138.71, 137.59, 134.70, 134.33, 132.43, 131.94, 131.47, 131.38, 131.26, 130.65, 129.90, 129.18, 129.14, 128.99, 128.72, 128.35, 128.28, 128.01, 127.87, 127.38, 126.90, 126.79, 125.86, 125.10, 124.93, 124.12, 121.80, 120.60, 120.55, 119.59, 116.74, 21.50. HRMS (ESI) calcd for $C_{35}H_{27}NO_2S_2Br$ [M+H]$^+$ 636.0667; found: 636.0671

2-phenyl-3-(2-phenyl-1-(p-tolyl)-2-tosylvinyl)-1-tosylindole (86): The title compound was prepared according to the procedure J via column chromatography of silica eluting hexane-ethyl acetate (80:20) to obtain as a white solid (40 mg, yield = 61%); Mp. 138.4-139.5 °C; ^{1}H NMR (400 MHz, $CDCl_3$) δ 8.30 (d, *J* = 8.4 Hz, 1H), 7.46 – 7.41 (m, 3H), 7.37 (ddd, *J* = 6.8, 5.5, 1.7 Hz, 3H), 7.29 – 7.20 (m, 4H), 7.15 – 7.09 (m, 4H), 7.08 – 7.03 (m, 4H), 6.92 (s, 3H), 6.51 (d, *J* = 8.0 Hz, 2H), 6.32 (d, *J* = 8.2 Hz, 2H), 2.30 (s, 6H), 2.03 (s, 3H). ^{13}C NMR (101 MHz, $CDCl_3$) δ 144.58, 144.43, 143.67, 143.10, 137.49, 137.28, 137.22, 136.73, 136.28, 135.26, 134.13, 132.21, 131.31, 131.24, 130.68, 129.35, 128.74, 128.61, 128.57, 128.47, 128.13, 127.90, 127.64, 127.15, 126.93, 124.83, 124.34, 124.27, 119.61, 116.51, 21.55, 21.49, 21.00. HRMS (ESI) calcd for $C_{43}H_{36}NO_4S_2$ [M+H]$^+$ 694.2007; found: 695.2010.

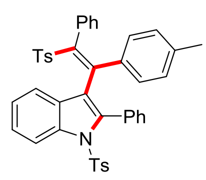

86

2-phenyl-3-(phenylethynyl)-1-tosylindole (87): The title compound was prepared according to the procedure K via column chromatography of silica eluting hexane-ethyl acetate (95:5) to obtain as an white solid (24 mg, yield = 58%); Mp. 138.4-139.5 °C; ^{1}H NMR (400 MHz, $CDCl_3$) δ 8.33 (d, *J* = 8.3 Hz, 1H), 7.69 – 7.62 (m, 3H), 7.51 – 7.47 (m, 3H), 7.45 – 7.40 (m, 1H), 7.38 – 7.34 (m, 3H), 7.30 – 7.26 (m, 5H), 7.05 (d, *J* = 8.0 Hz, 2H), 2.29 (s, 3H). ^{13}C NMR (101 MHz, $CDCl_3$) δ 144.86, 143.49, 137.14, 134.50, 131.41, 131.24, 130.72, 130.69, 129.32, 129.08, 128.27, 128.25, 127.27, 126.84, 125.75, 124.70, 123.09, 120.10, 116.64, 109.99, 108.21, 94.69, 81.46, 21.54. HRMS (ESI) calcd for $C_{29}H_{22}NO_2S$ [M+H]$^+$ 448.1371; found: 448.1365.

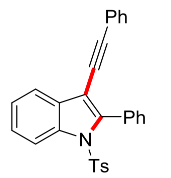

87

(*Z*)-2-phenyl-3-(2-phenyl-2-tosylvinyl)-1-tosylindole (88): The title compound was prepared according to the procedure L via column chromatography of silica eluting hexane-ethyl acetate (80:20) to obtain as a white solid (32 mg, yield = 54%); Mp. 138.4-139.5 °C; ^{1}H NMR (400 MHz, $CDCl_3$) δ 8.29 (d, *J* = 8.3 Hz, 1H), 7.57 – 7.53 (m, 1H), 7.52 – 7.47 (m, 2H), 7.46 – 7.41 (m, 3H), 7.40 – 7.32 (m, 4H), 7.29 – 7.24 (m, 1H), 7.21 (d, *J* = 4.5 Hz, 4H), 7.11 (d, *J* = 8.4 Hz, 4H), 7.01 (d, *J* = 8.0 Hz, 2H), 6.57 (s, 1H), 2.33 (s, 3H), 2.26 (s, 3H). ^{13}C NMR (101 MHz, $CDCl_3$) δ 147.04, 144.61, 144.11, 137.76, 137.60, 136.60, 135.11, 134.91, 132.21, 131.30, 131.23, 130.98, 129.63, 129.29, 128.99, 128.93, 128.80, 128.29, 128.07, 127.36, 126.73, 125.18, 124.43, 120.68, 119.98, 116.57, 21.54. HRMS (ESI) calcd for $C_{36}H_{30}NO_4S_2$ [M+H]$^+$ 604.1616; found: 604.1619.

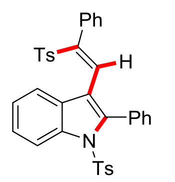

88

(2-tosylethene-1,1-diyl)dibenzene (92): The title compound was prepared according to the modified general procedure D via column chromatography of silica eluting hexane-ethyl acetate (80:20) to obtain as a white solid (8 mg, yield = 15%); ^{1}H NMR (400 MHz, $CDCl_3$) δ 7.47 (d, *J* = 8.3 Hz, 2H), 7.35 (ddd, *J* = 4.5, 3.7, 2.7 Hz, 2H), 7.32 – 7.27 (m, 4H), 7.21 – 7.19 (m, 2H), 7.15 (dd, *J* = 8.5, 0.6 Hz, 2H), 7.11 – 7.08 (m, 2H), 6.99 (s, 1H), 2.38 (s, 3H). ^{13}C NMR (101 MHz, $CDCl_3$) δ 154.66, 143.71, 139.19, 138.56, 135.53, 130.18, 129.73, 129.29, 128.91, 128.79, 128.52, 128.16, 127.76, 127.65, 21.53. HRMS (ESI) calcd for $C_{21}H_{19}O_2S$ [M+H]$^+$ 335.1106; found: 335.1109.

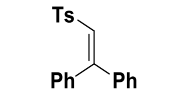

92

(*E*)-*N*-(2-iodo-2-phenyl-1-tosylvinyl)-4-methyl-*N*-phenylbenzenesulfonamide (94a): The title compound was prepared according to the general procedure D via column chromatography of silica eluting hexane-ethyl acetate (85:15 to obtain as a lite brown solid (37 mg, yield = 58%); Mp. 138.4-139.5 °C; ^{1}H NMR (400 MHz, $CDCl_3$) δ 7.95 (dd, *J* = 8.1, 1.7 Hz, 2H), 7.70 (d, *J* = 8.4 Hz, 2H), 7.45 – 7.37 (m, 3H), 7.32 (d, *J* = 8.4 Hz, 2H), 7.17 (d, *J* = 8.1 Hz, 3H), 7.12 (t, *J* = 7.2 Hz, 2H), 6.97 (d, *J* = 8.0 Hz, 3H), 6.72 (s, 1H), 2.36 (s, 3H), 2.33 (s, 3H). ^{13}C NMR (101 MHz, $CDCl_3$) δ 147.96, 144.05, 143.55, 140.03, 138.96, 137.29, 135.94, 131.45, 129.18, 129.12, 129.04, 128.65, 128.55, 128.29, 127.83, 127.46, 123.78, 21.58, 21.51. HRMS (ESI) calcd for $C_{28}H_{24}NO_4NaS_2I$ $[M+Na]^+$ 652.0089; found: 652.0097.

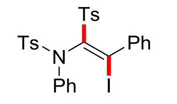

94a

((*E*)-3-(1-iodo-2-phenyl-2-tosylvinyl-2-^{13}C)-2-phenyl-1-tosylindole (3'): The title compound was prepared according to the general procedure D via column chromatography of silica eluting hexane-ethyl acetate (80:20) to obtain as a white solid (56 mg, yield = 79%); Mp. 189-191 °C; ^{1}H NMR (400 MHz, $CDCl_3$) δ 8.34 (d, *J* = 8.3 Hz, 1H), 7.70 – 7.63 (m, 3H), 7.49 (dd, *J* = 5.5, 2.4 Hz, 3H), 7.45 – 7.41 (m, 1H), 7.37 (ddd, *J* = 6.7, 2.9, 1.3 Hz, 3H), 7.31 – 7.27 (m, 5H), 7.06 (d, *J* = 8.1 Hz, 2H), 2.29 (s, 3H). ^{13}C NMR (101 MHz, $CDCl_3$) δ 151.44, 145.24, 144.70, 144.43, 139.12, 138.54, 137.14, 136.30, 135.81, 135.72, 135.15, 131.10, 130.13, 130.02, 129.43, 129.14, 129.09, 129.04, 128.62, 128.27, 128.14, 128.12, 127.95, 127.43, 126.83, 126.08, 125.44, 124.45, 122.56, 119.37, 116.50, 108.76, 107.93, 100.62, 21.57, 21.54. HRMS (ESI) calcd for $H_{29}NO_4S_2I^{12}C_{35}{}^{13}C$ $[M+H]^+$ 731.0616; found: 731.0620.

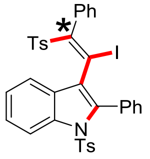

3'

* = ^{13}C labeled carbon

(*E*)-3-(1-iodo-2-phenyl-2-tosylvinyl)-1-(methylsulfonyl)-2-phenylindole (95): The title compound was prepared according to the general procedure D via column chromatography of silica eluting hexane-ethyl acetate (80:20) to obtain as a white solid (54 mg, yield = 80%); Mp. 187-188°C; ^{1}H NMR (400 MHz, $CDCl_3$) δ 8.25 – 8.18 (m, 1H), 7.82 (s, 2H), 7.63 – 7.53 (m, 4H), 7.52 – 7.47 (m, 2H), 7.35 – 7.26 (m, 2H), 7.21 (d, *J* = 7.4 Hz, 1H), 7.17 (d, *J* = 8.4 Hz, 2H), 7.09 (d, *J* = 8.0 Hz, 2H), 7.02 (d, *J* = 6.8 Hz, 1H), 6.81 (d, *J* = 7.3 Hz, 1H), 2.88 (s, 3H), 2.36 (s, 3H). ^{13}C NMR (101 MHz, $CDCl_3$) δ 151.74, 144.83, 138.46, 137.24, 136.24, 135.43, 130.90, 130.53, 130.26, 130.18, 129.38, 129.28, 129.24, 128.76, 128.40, 127.92, 127.74, 126.60, 125.92, 124.94, 119.69, 116.40, 107.70, 40.13, 21.62. HRMS (ESI) calcd for $C_{30}H_{25}NO_4S_2I$ $[M+H]^+$ 654.0270; found: 654.0275.

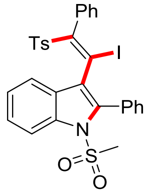

95

(*E*)-1-(ethylsulfonyl)-3-(1-iodo-2-(4-methoxyphenyl)-2-tosylvinyl)-2-phenylindole (96): The title compound was prepared according to the general procedure D via column chromatography of silica eluting hexane-ethyl acetate (80:21) to obtain as a white solid (41 mg, yield = 58%); Mp. 115-116°C; ^{1}H NMR (400 MHz, $CDCl_3$) 8.25 – 8.18 (m, 1H), 7.80 (brs, 2H), 7.61 – 7.51 (m, 4H), 7.50 – 7.45 (m, 2H), 7.18 (d, *J* = 8.3 Hz, 2H), 7.10 (d, *J* = 8.1 Hz, 2H), 6.91 (d, *J* = 7.7 Hz, 1H), 6.80 (d, *J* = 7.8 Hz, 1H), 6.71 (s, 2H), 3.79 (s, 3H), 3.17 – 3.06 (m, 2H), 2.37 (s, 3H), 1.14 (t, *J* = 7.4 Hz, 3H). ^{13}C NMR (101 MHz, $CDCl_3$) δ 160.14, 151.25, 144.63, 137.10, 136.42, 135.70, 131.65, 130.87, 130.83, 129.32,

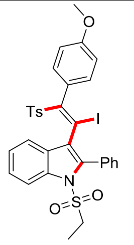

96

129.22, 128.74, 127.66, 127.57, 125.67, 125.52, 124.48, 119.68, 115.82, 113.63, 113.47, 109.97, 108.92, 55.21, 48.69, 21.63, 7.52. HRMS (ESI) calcd for $C_{32}H_{28}INNaO_5S_2$ $[M+Na]^+$ 720.0345; found: 720.0348.

(*E*)-*N*-(3-iodo-3-(4-methoxyphenyl)-2-tosylallyl)-4-methyl-*N*-(2 (phenylethynyl)phenyl)benzenesulfonamide (97a): The title compound was prepared according to the general

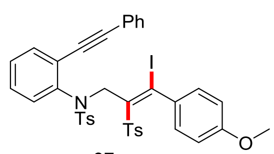

97a

procedure D via column chromatography of silica eluting hexane-ethyl acetate (80:20) to obtain as a white solid (41 mg, yield = 55%); Mp. 126-128 °C; ^1H NMR (400 MHz, $CDCl_3$) δ 7.70 (d, *J* = 8.3 Hz, 2H), 7.60 – 7.57 (m, 1H), 7.56 – 7.52 (m, 1H), 7.43 (ddd, *J* = 7.2, 6.0, 2.3 Hz, 2H), 7.40 – 7.35 (m, 2H), 7.34 –7.29 (m, 3H), 7.11 (d, *J* = 8.1 Hz, 2H), 7.07 (d, *J* = 8.3 Hz, 2H), 6.94 (d, *J* = 8.1 Hz, 2H), 6.74 (d, *J* = 8.2 Hz, 2H), 6.51 (d, *J* = 8.1 Hz, 2H), 5.58 (d, *J* = 14.7 Hz, 1H), 5.22 (d, *J* = 14.7 Hz, 1H), 3.76 (s, 3H), 2.30 (s, 3H), 2.24 (s, 3H). ^{13}C NMR (101 MHz, $CDCl_3$) δ 160.00, 144.02, 143.42, 143.23, 140.00, 138.17, 136.15, 135.04, 133.73, 133.56, 131.62, 129.87, 129.34, 128.81, 128.51, 128.37, 128.33, 128.08, 127.29, 124.62, 123.09, 121.16, 112.85, 94.38, 86.92, 57.95, 55.33, 21.47, 21.43. HRMS (ESI) calcd for $C_{38}H_{32}NO_5NaS_2I$ $[M+Na]^+$ 796.0664; found: 796.0667.

(*E*)-1-((2-iodo-2-phenylvinyl)sulfonyl)-4-methylbenzene (98): The title compound was prepared according to

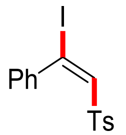

98

the modified general procedure D via column chromatography of silica eluting hexane-ethyl acetate (82:18) to obtain as a white solid (28 mg, yield = 61%); Mp. 81-83 °C; ^1H NMR (400 MHz, $CDCl_3$) δ 7.44 (d, *J* = 8.3 Hz, 2H), 7.33 – 7.19 (m, 5H), 7.17 (d, *J* = 8.0 Hz, 2H), 2.37 (s, 3H). ^{13}C NMR (101 MHz, $CDCl_3$) δ 144.45, 141.13, 139.50, 137.15, 129.64, 129.54, 127.77, 127.71, 127.55, 114.08, 21.51. HRMS (ESI) calcd for $C_{15}H_{13}INaO_2S$ $[M+Na]^+$ 406.9573; found: 406.9574.

2-(phenylethynyl)tetrahydrofuran (102): The title compound was prepared according to the procedure M via column chromatography of silica eluting hexane-ethyl acetate (98:2) to obtain as a yellow liquid (8 mg, yield =

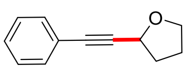

102

19%); Mp. 126-128 °C; ^1H NMR (400 MHz, $CDCl_3$) δ 7.45 – 7.41 (m, 2H), 7.31 – 7.27 (m, 3H), 4.81 (dd, *J* = 7.2, 5.1 Hz, 1H), 4.05 – 3.98 (m, 1H), 3.89 – 3.83 (m, 1H), 2.26 – 2.19 (m, 1H), 2.13 – 2.05 (m, 2H), 1.98 – 1.91 (m, 1H). ^{13}C NMR (101 MHz, $CDCl_3$) δ 131.65, 128.19, 128.15, 122.74, 89.00, 84.43, 68.56, 67.89, 33.37, 25.45. HRMS (ESI) calcd for $C_{12}H_{13}O$ $[M+H]^+$ 173.0966; found: 173.0966.

1.13. Copies of ^{1}H, ^{13}C spectra

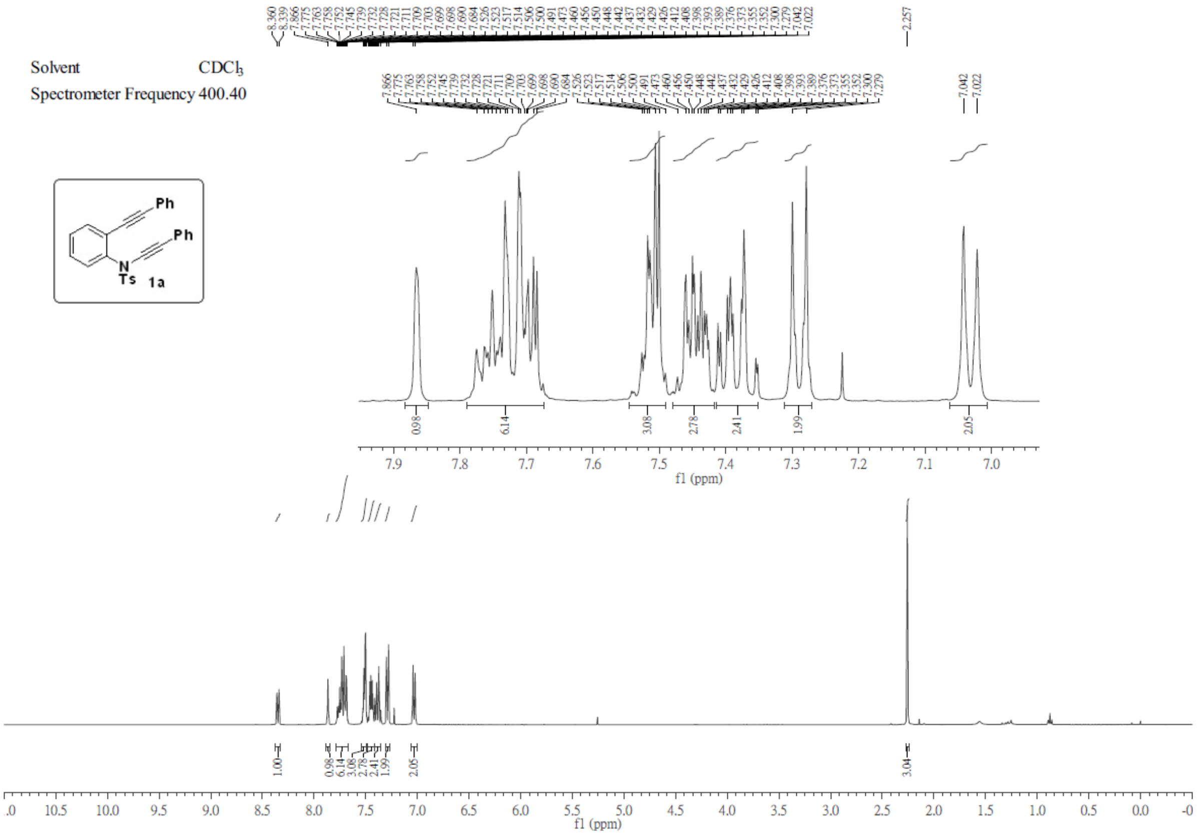

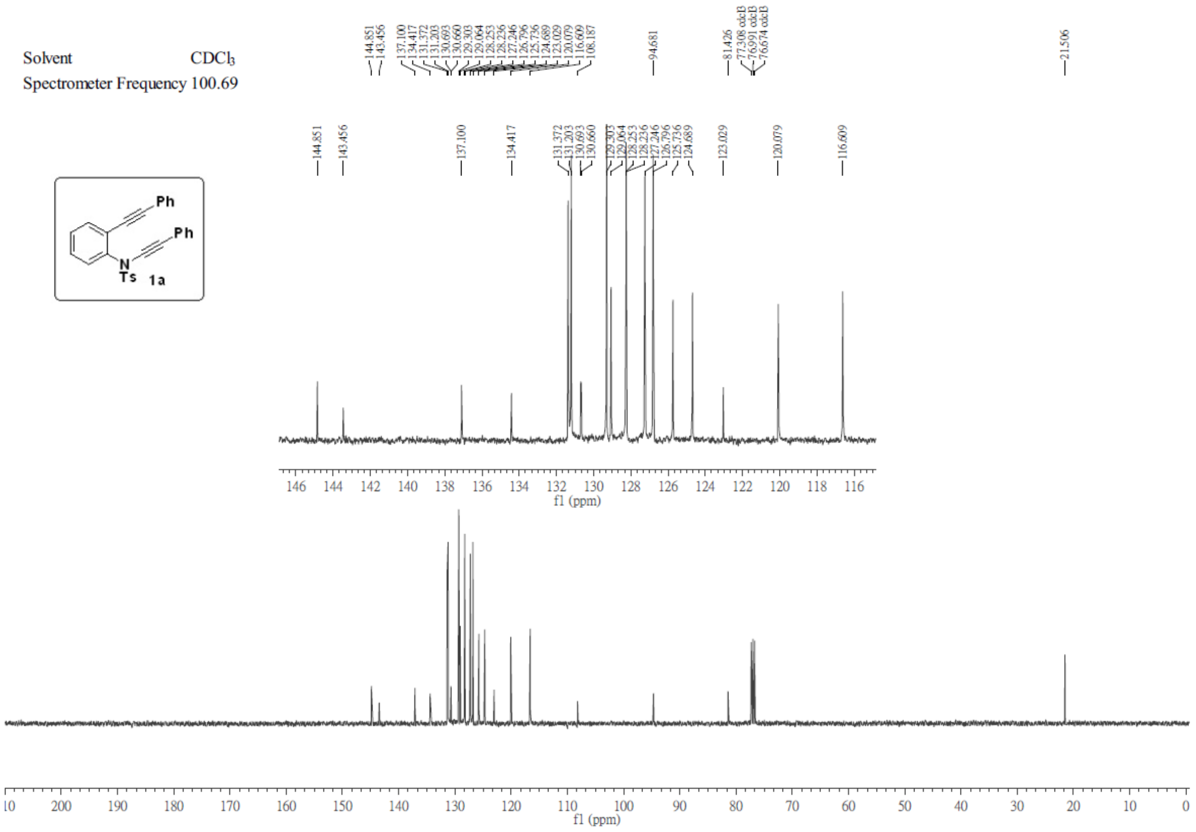

Supplementary Figure 56. ^{1}H (top) and ^{13}C (bottom) NMR spectra of compound **1a**.

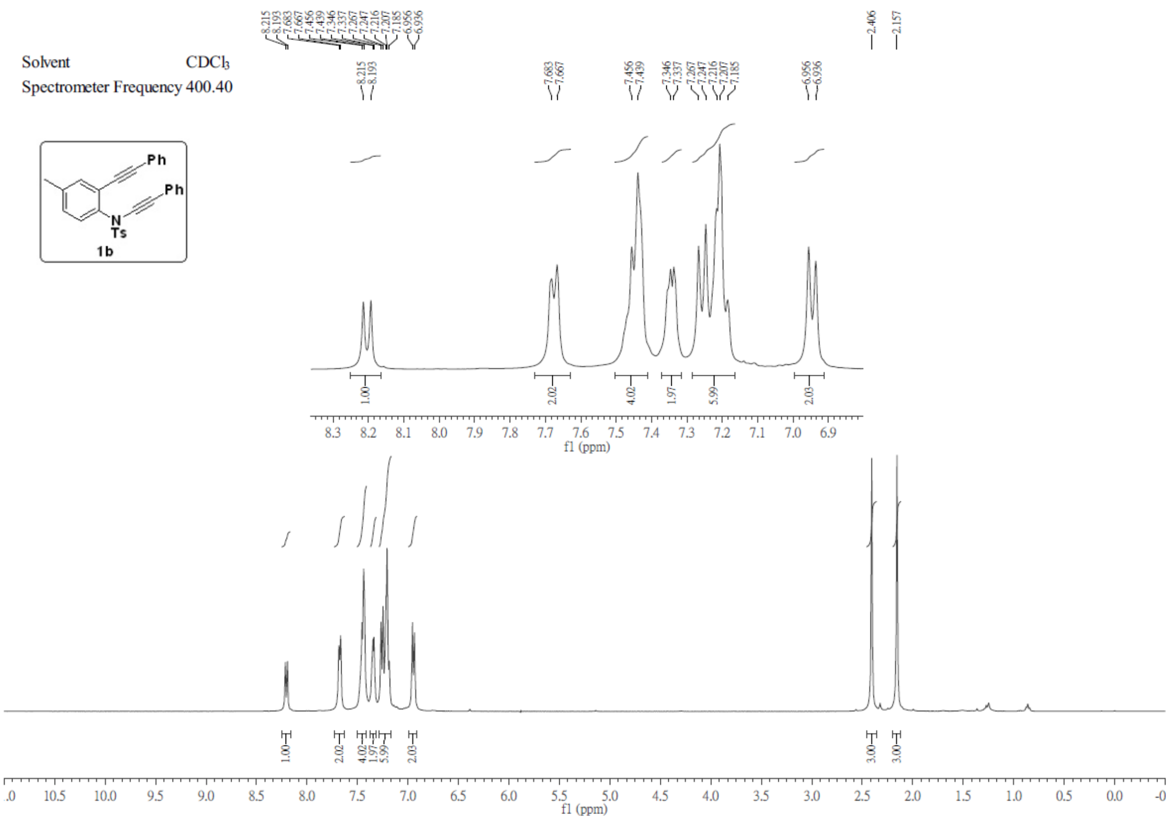

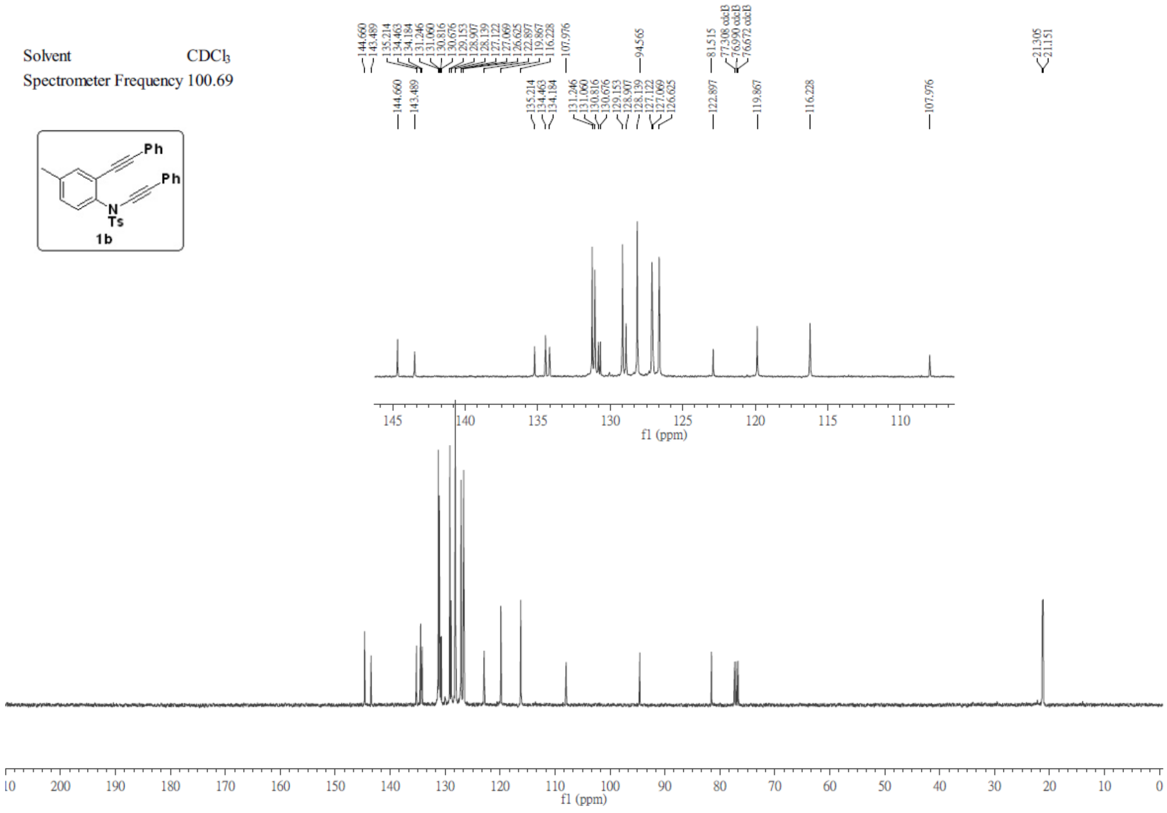

Supplementary Figure 57. ^{1}H (top) and ^{13}C (bottom) NMR spectra of compound **1b**.

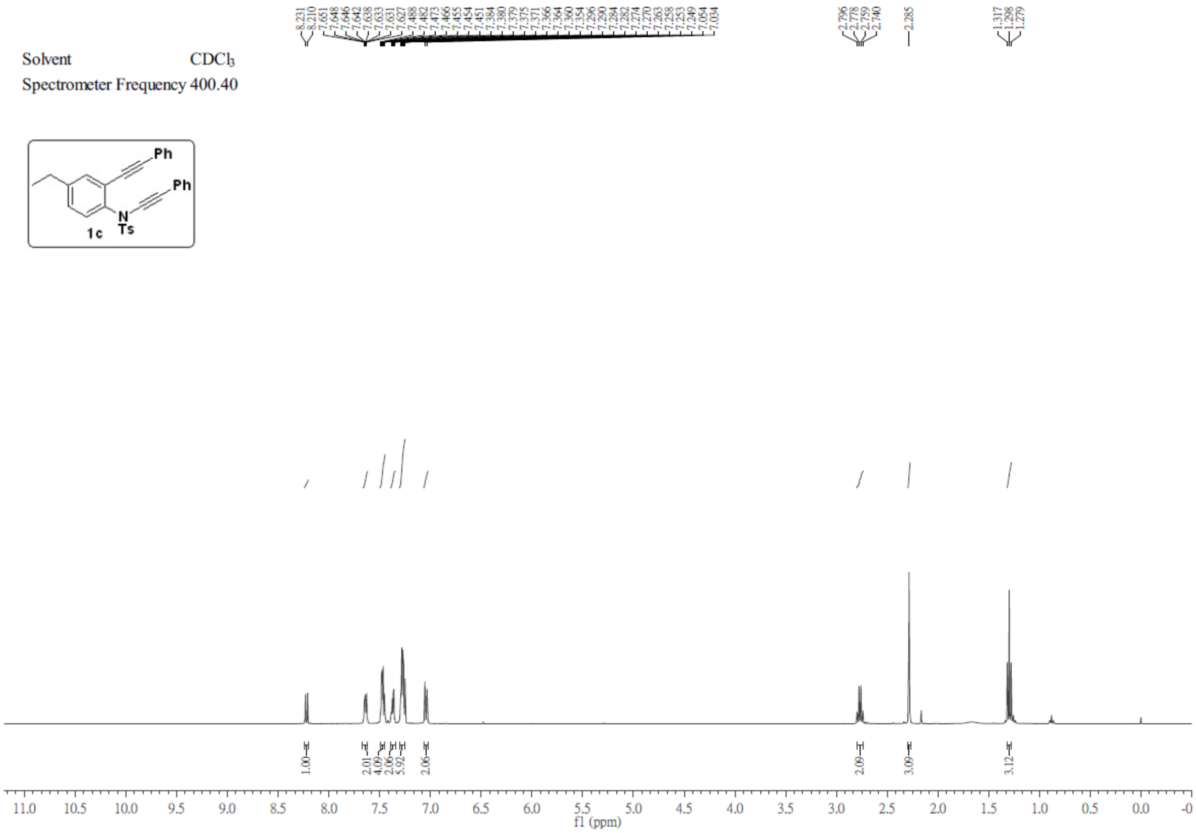

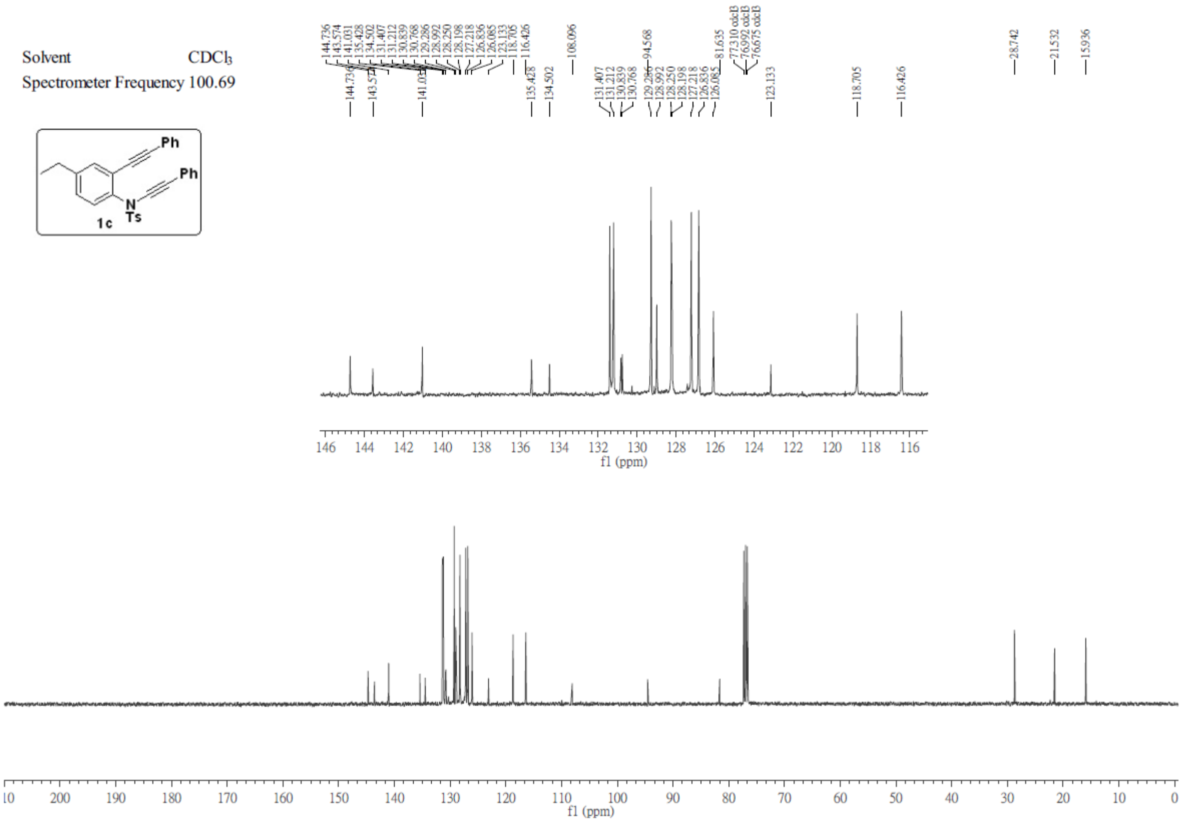

Supplementary Figure 58. ^{1}H (top) and ^{13}C (bottom) NMR spectra of compound **1c**.

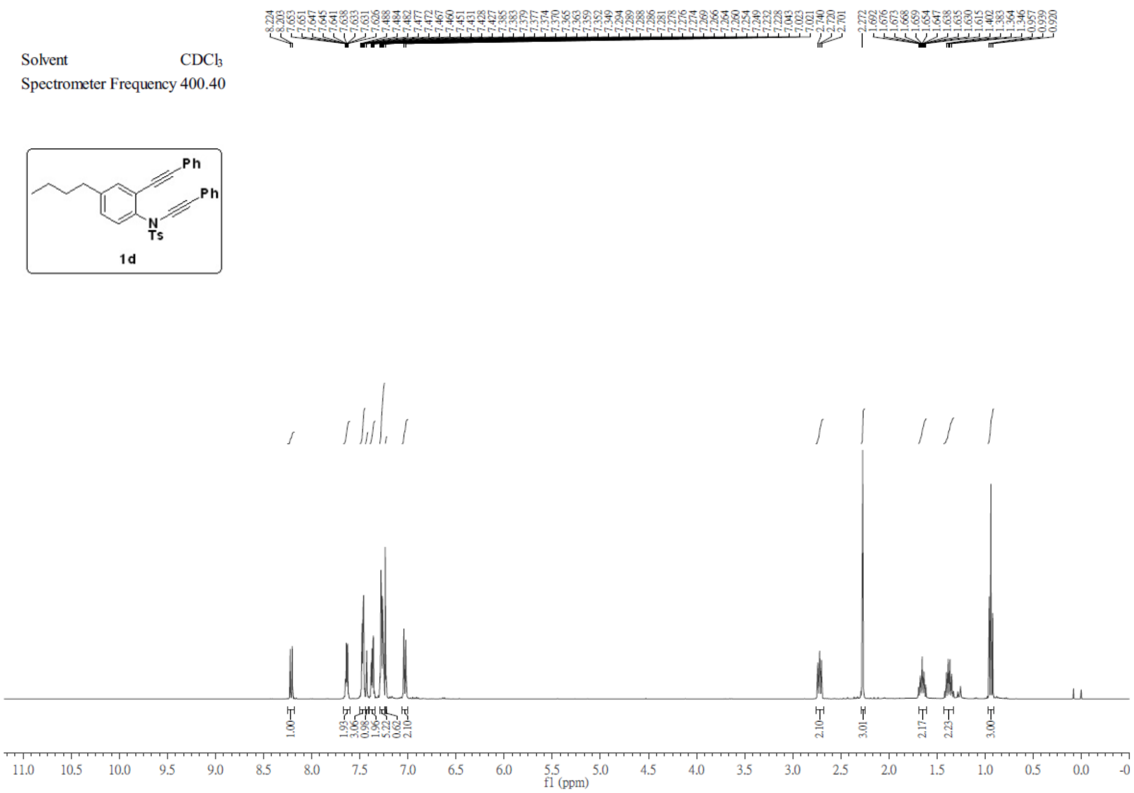

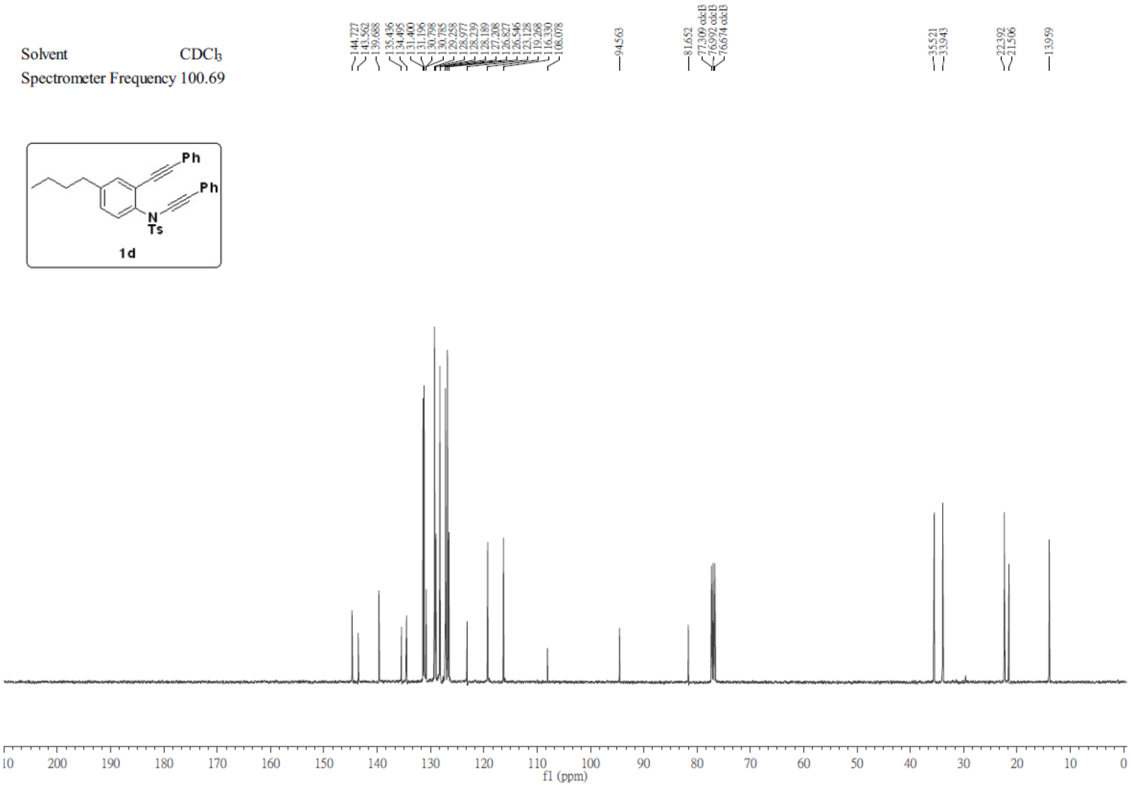

Supplementary Figure 59. ^{1}H (top) and ^{13}C (bottom) NMR spectra of compound **1d**.

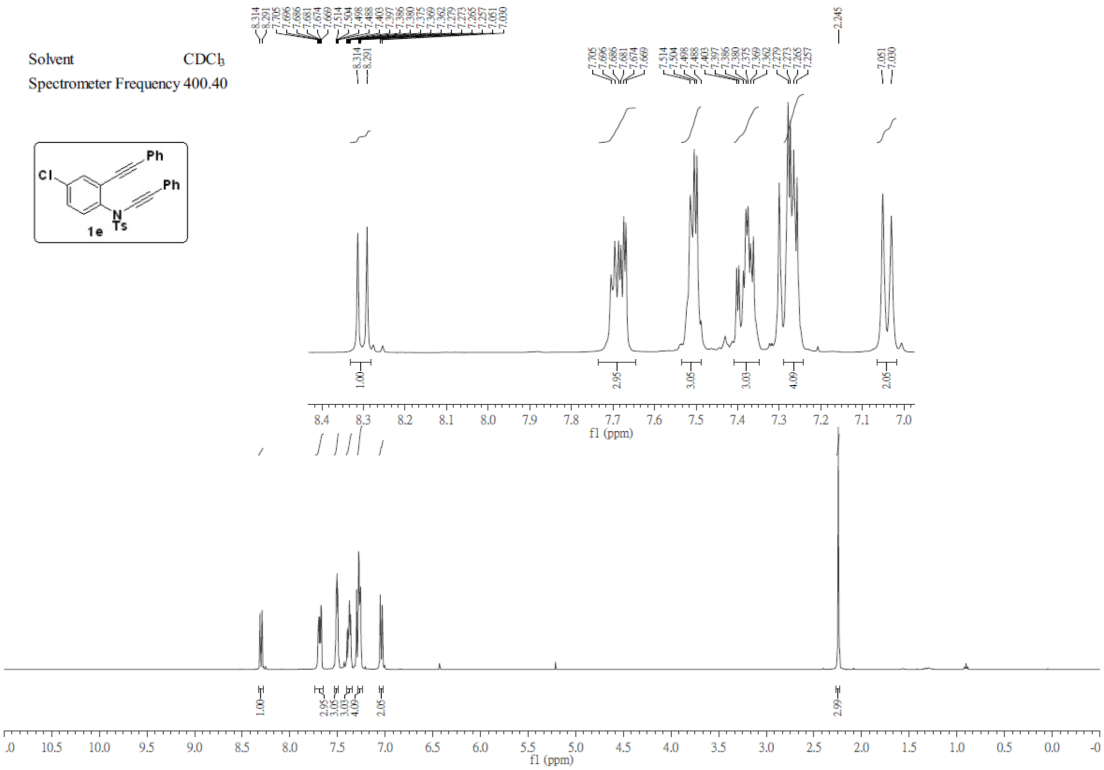

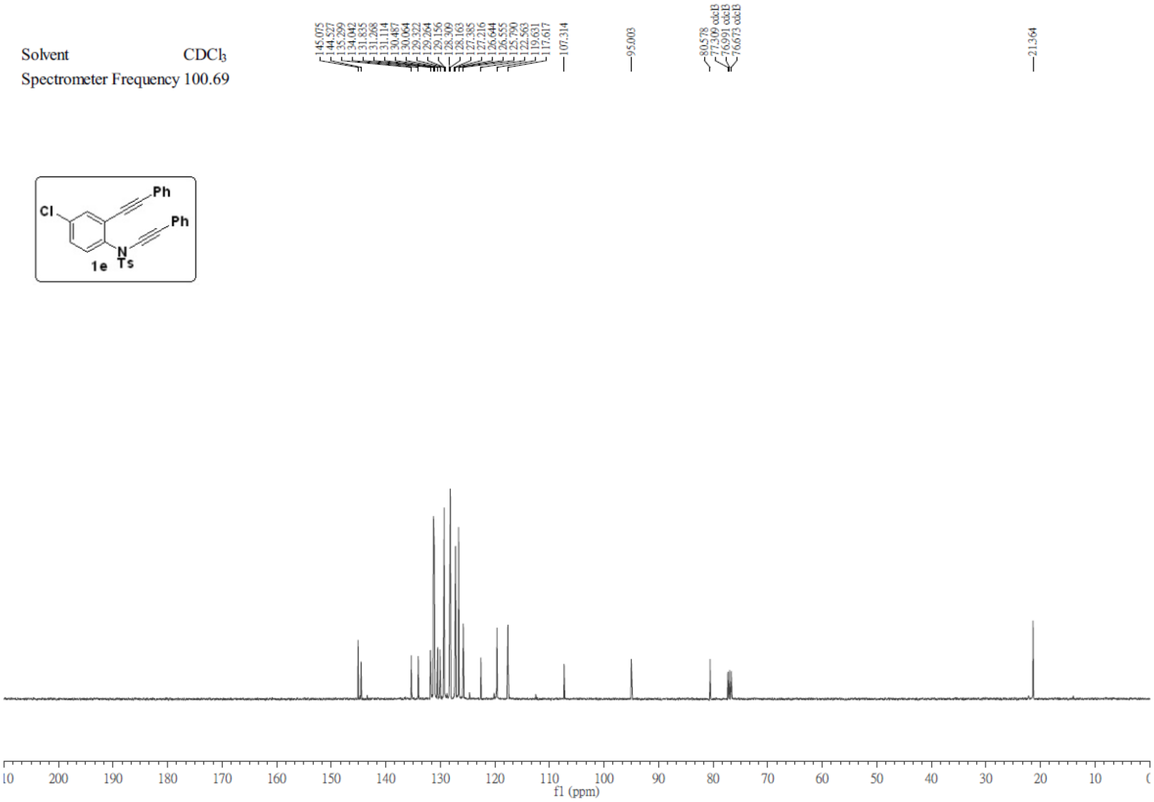

Supplementary Figure 60. ^{1}H (top) and ^{13}C (bottom) NMR spectra of compound **1e**.

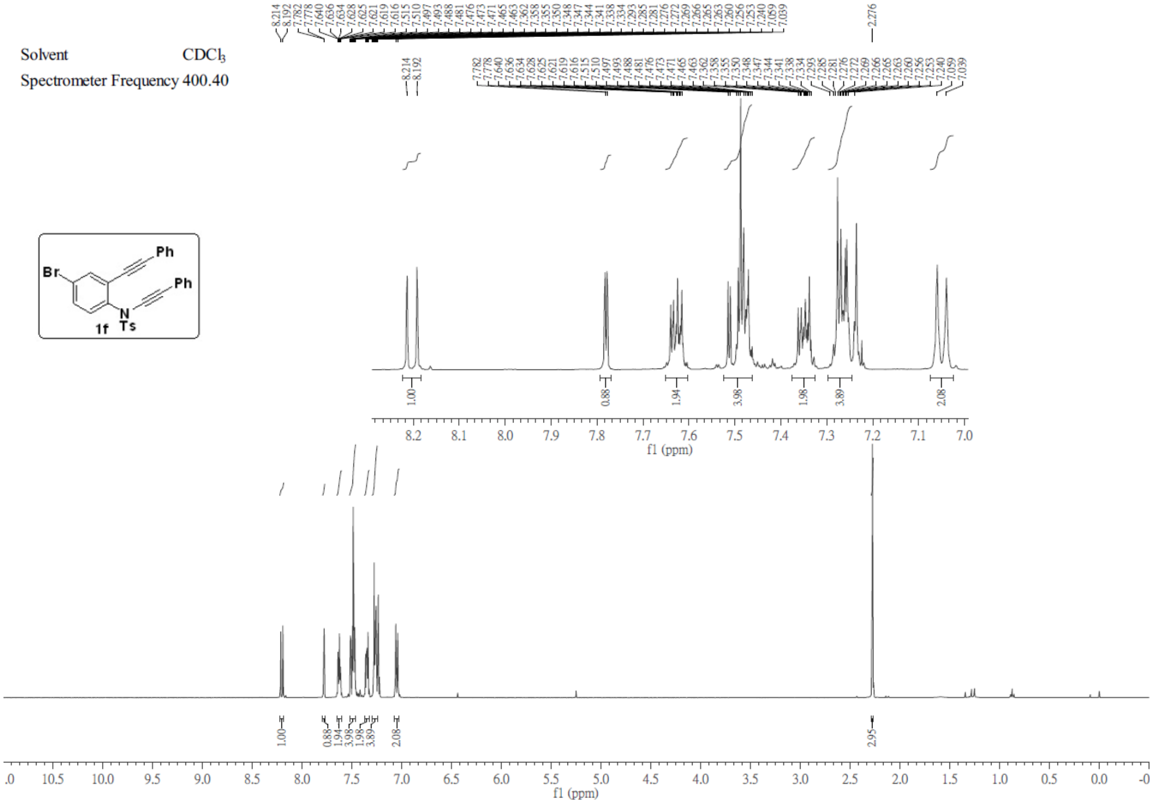

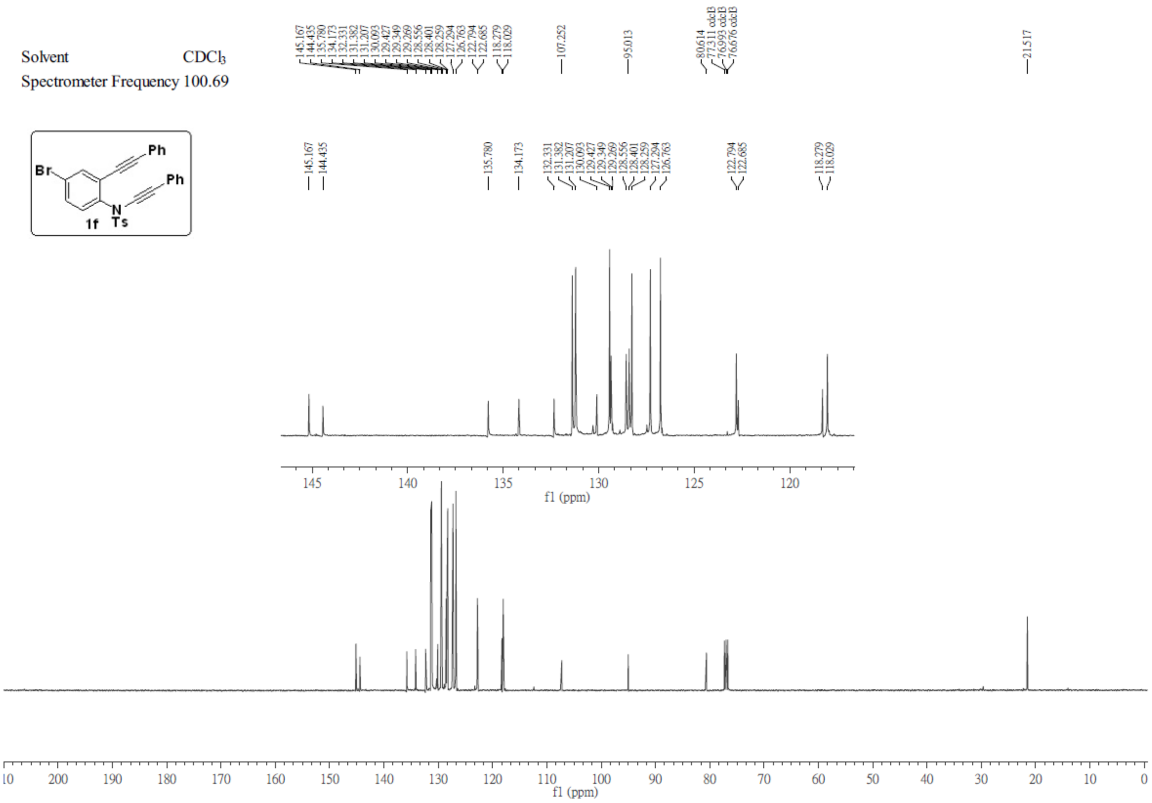

Supplementary Figure 61. ^{1}H (top) and ^{13}C (bottom) NMR spectra of compound **1f**.

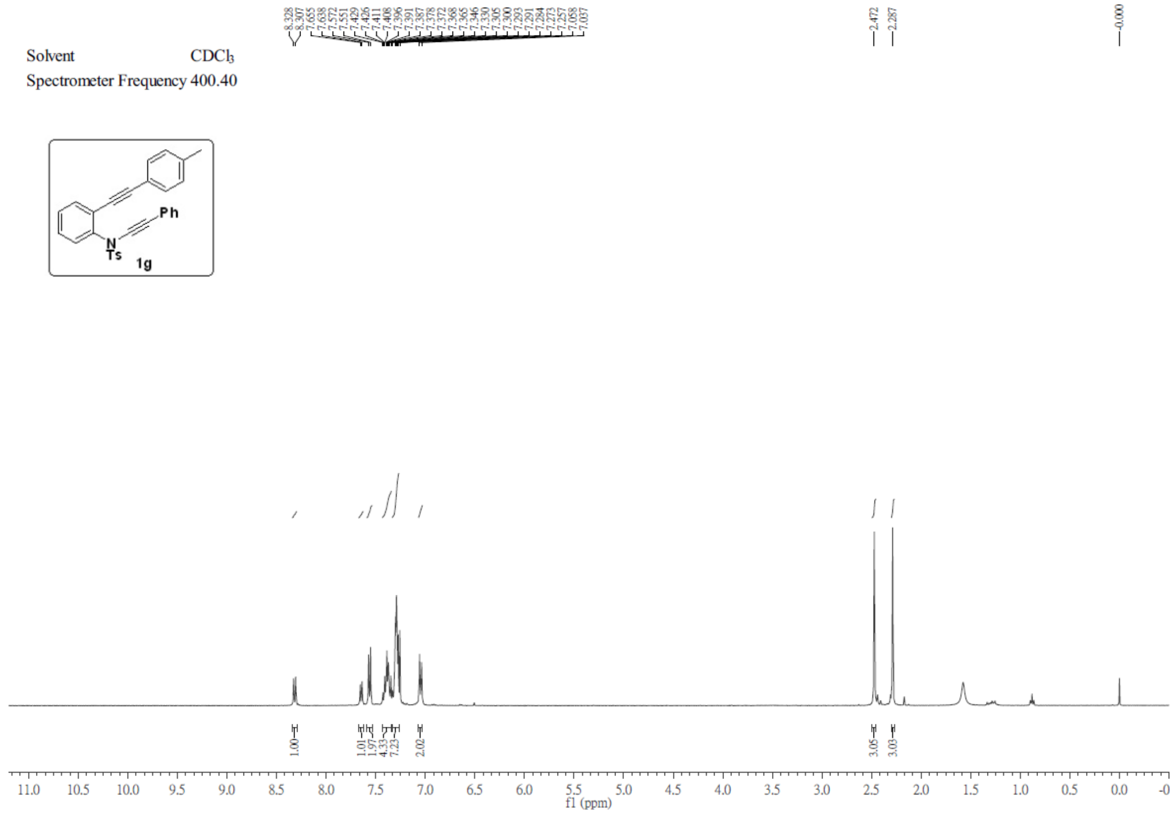

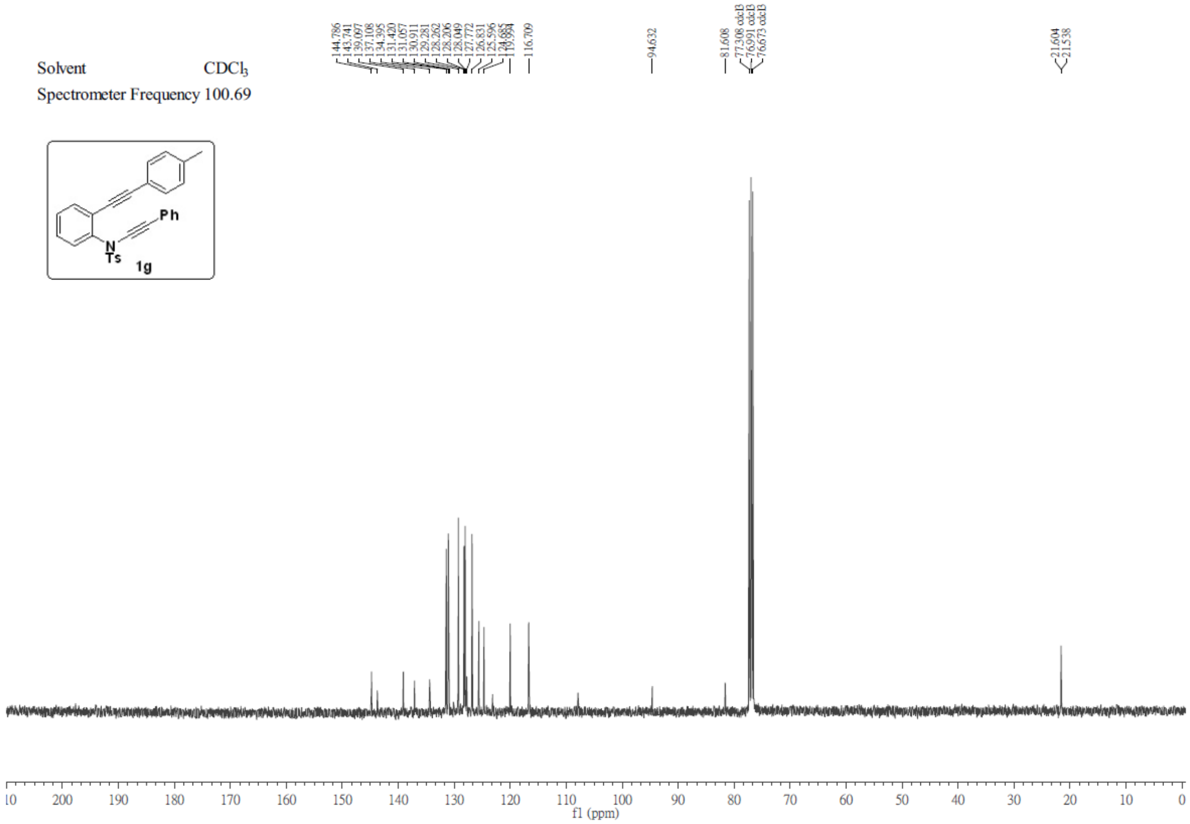

Supplementary Figure 62. ^{1}H (top) and ^{13}C (bottom) NMR spectra of compound **1g**.

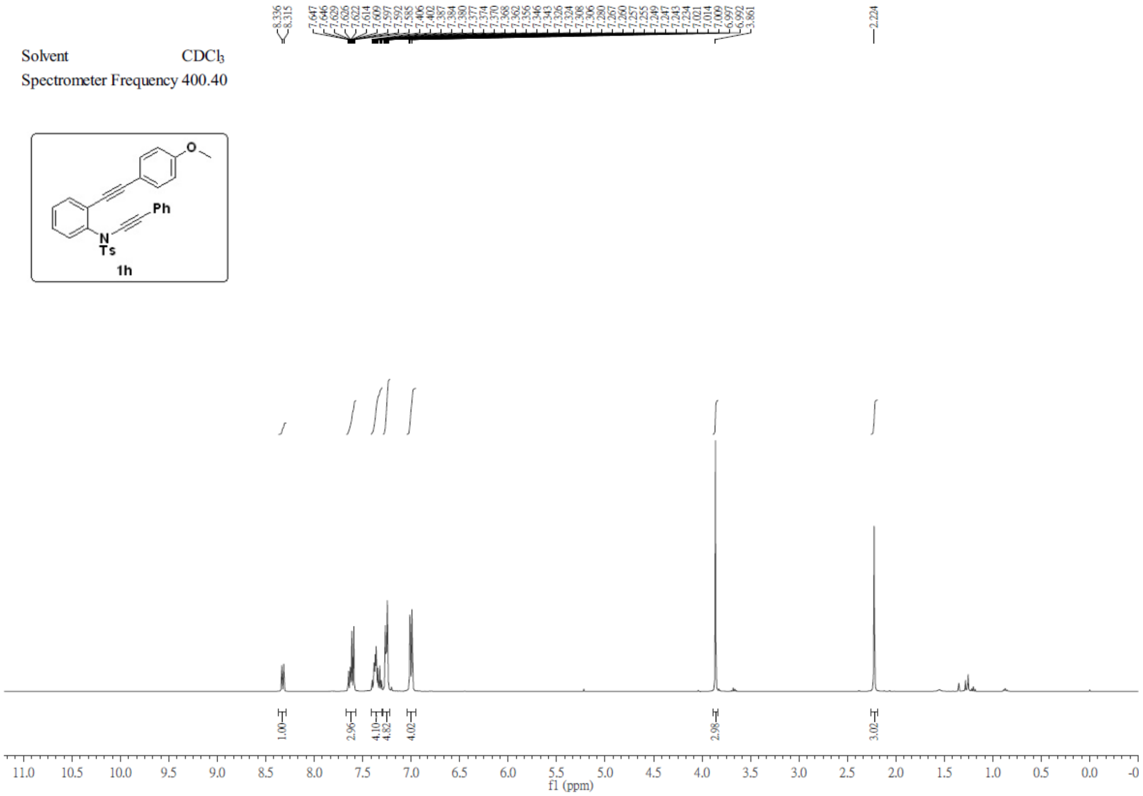

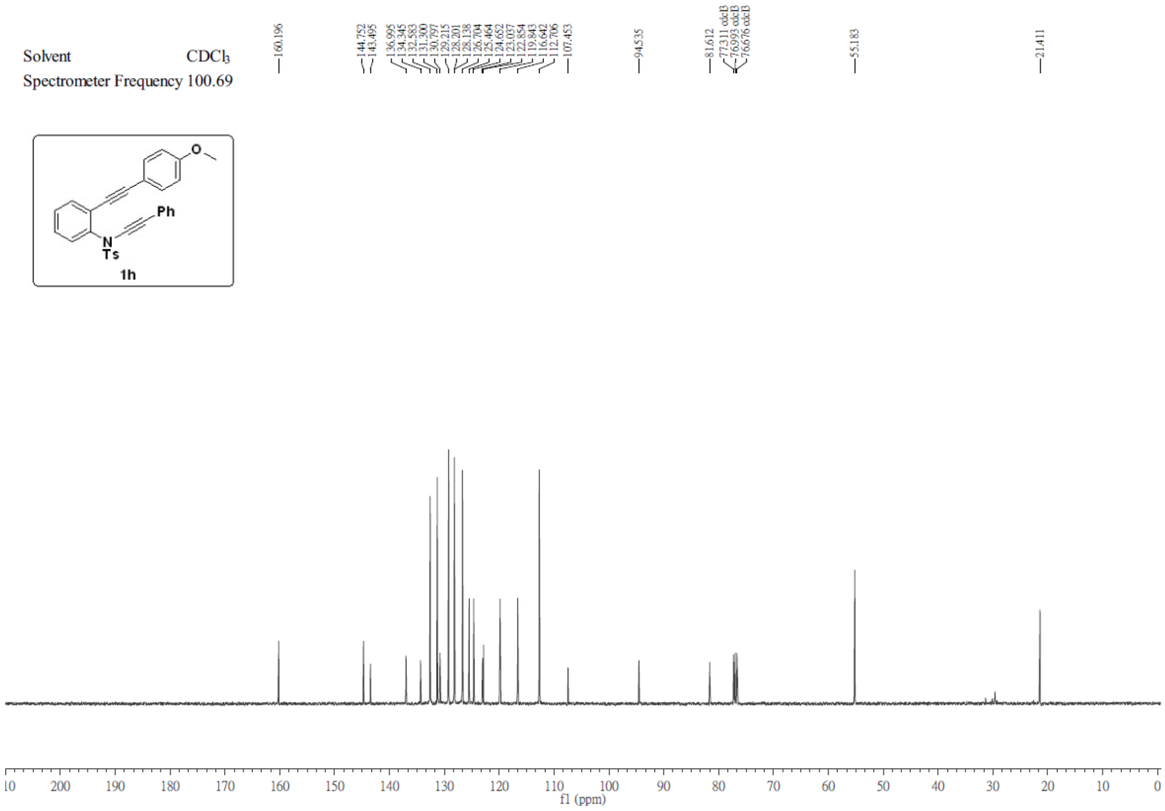

Supplementary Figure 63. ^{1}H (top) and ^{13}C (bottom) NMR spectra of compound **1h**.

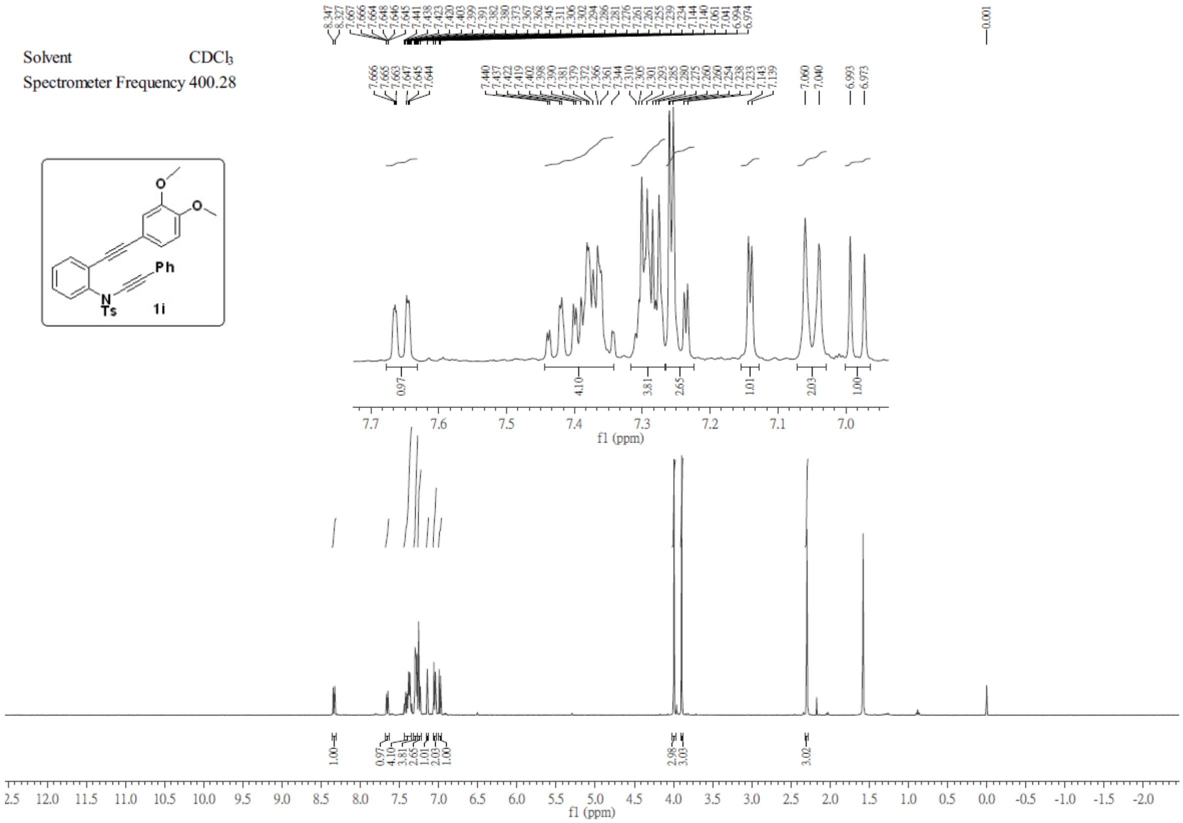

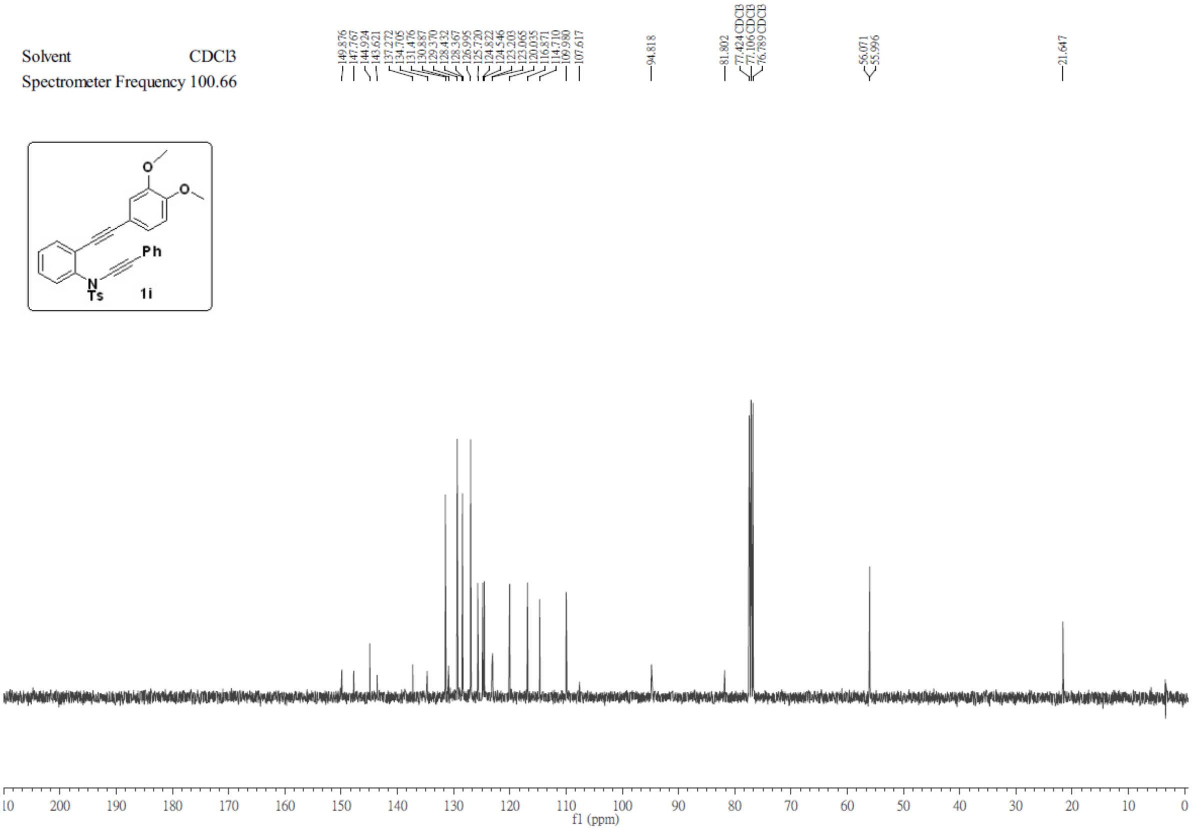

Supplementary Figure 64. ^{1}H (top) and ^{13}C (bottom) NMR spectra of compound **1i**.

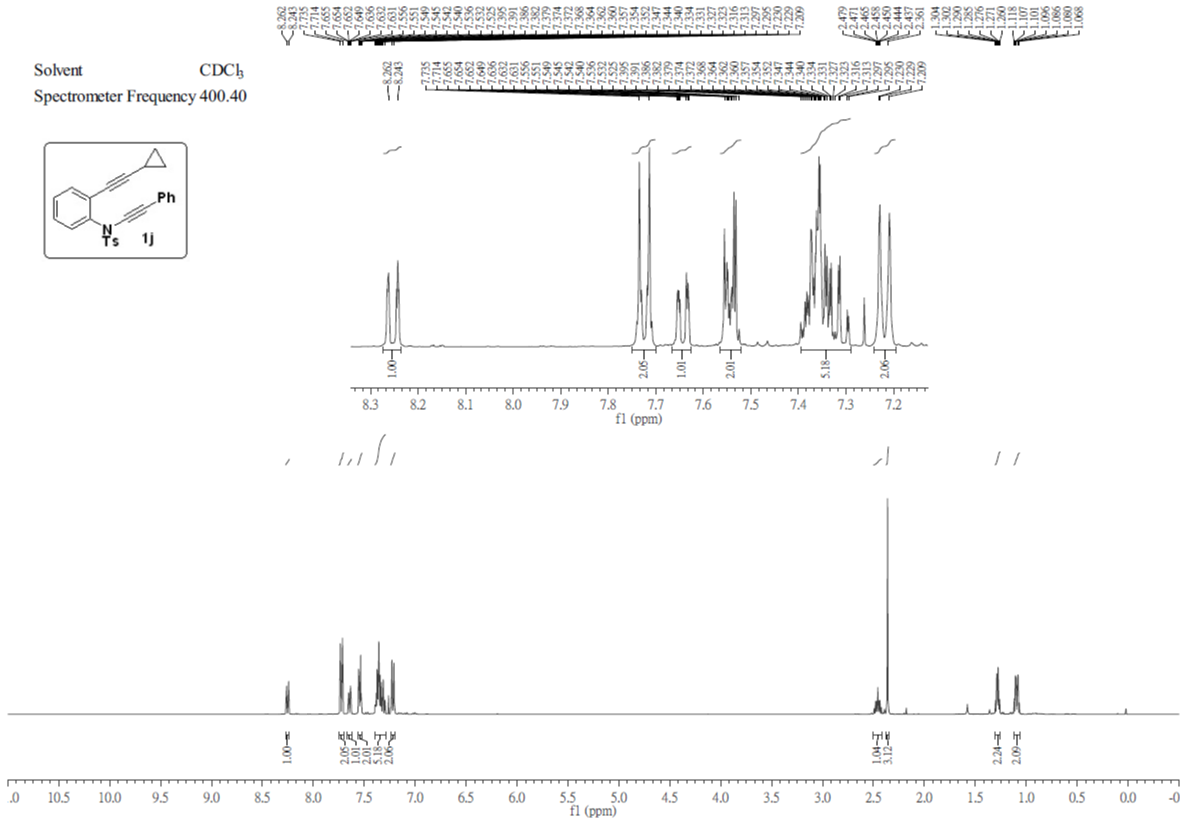

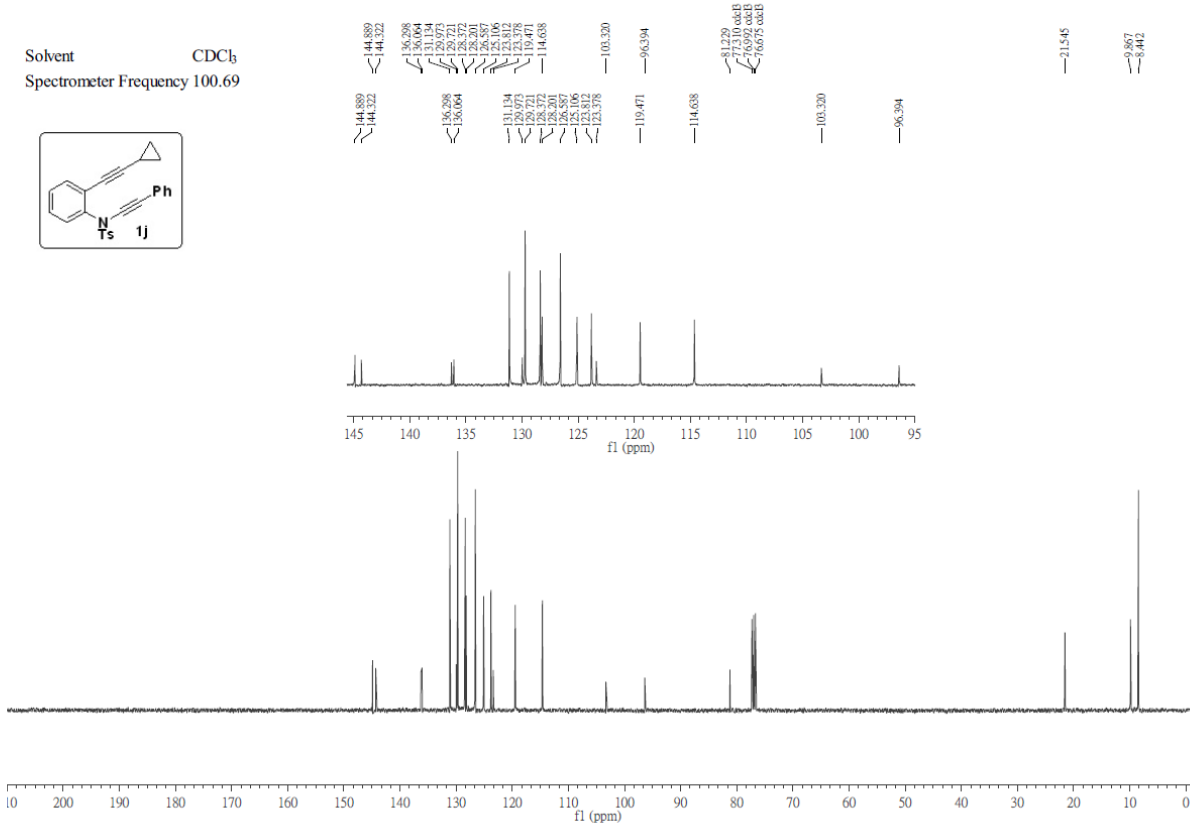

Supplementary Figure 65. ^{1}H (top) and ^{13}C (bottom) NMR spectra of compound **1j**.

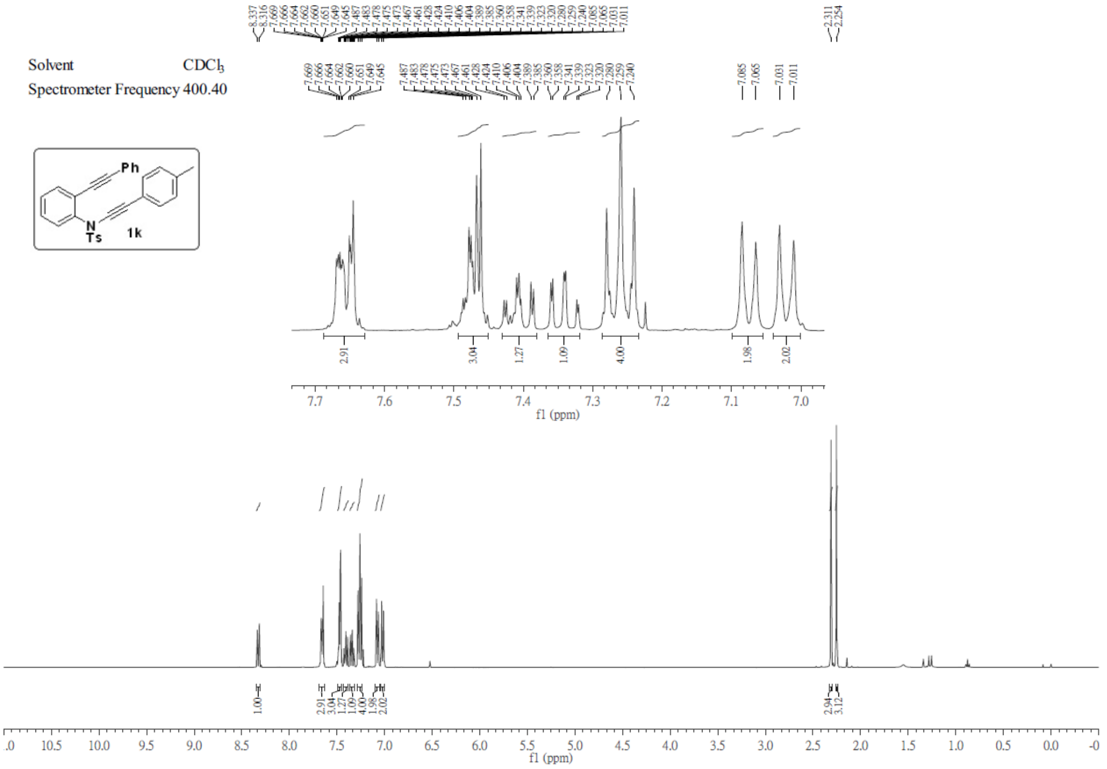

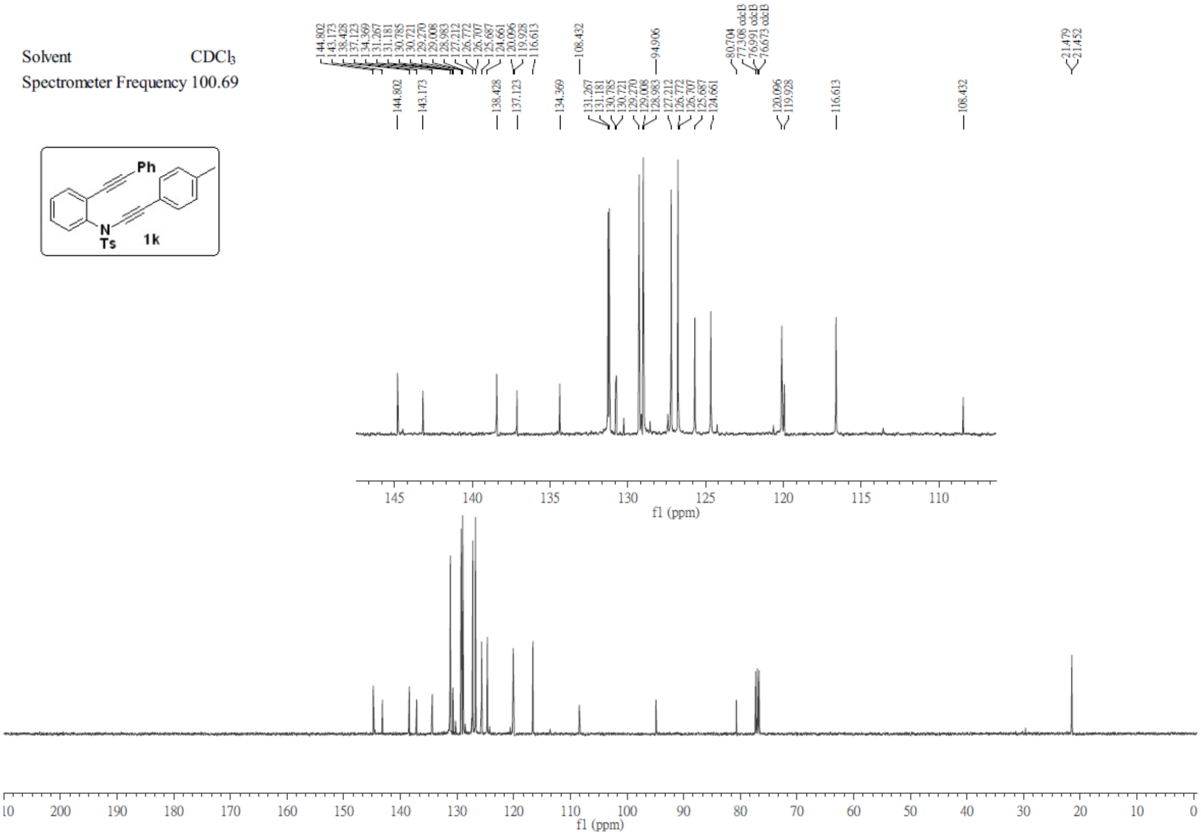

Supplementary Figure 66. ^{1}H (top) and ^{13}C (bottom) NMR spectra of compound **1k**.

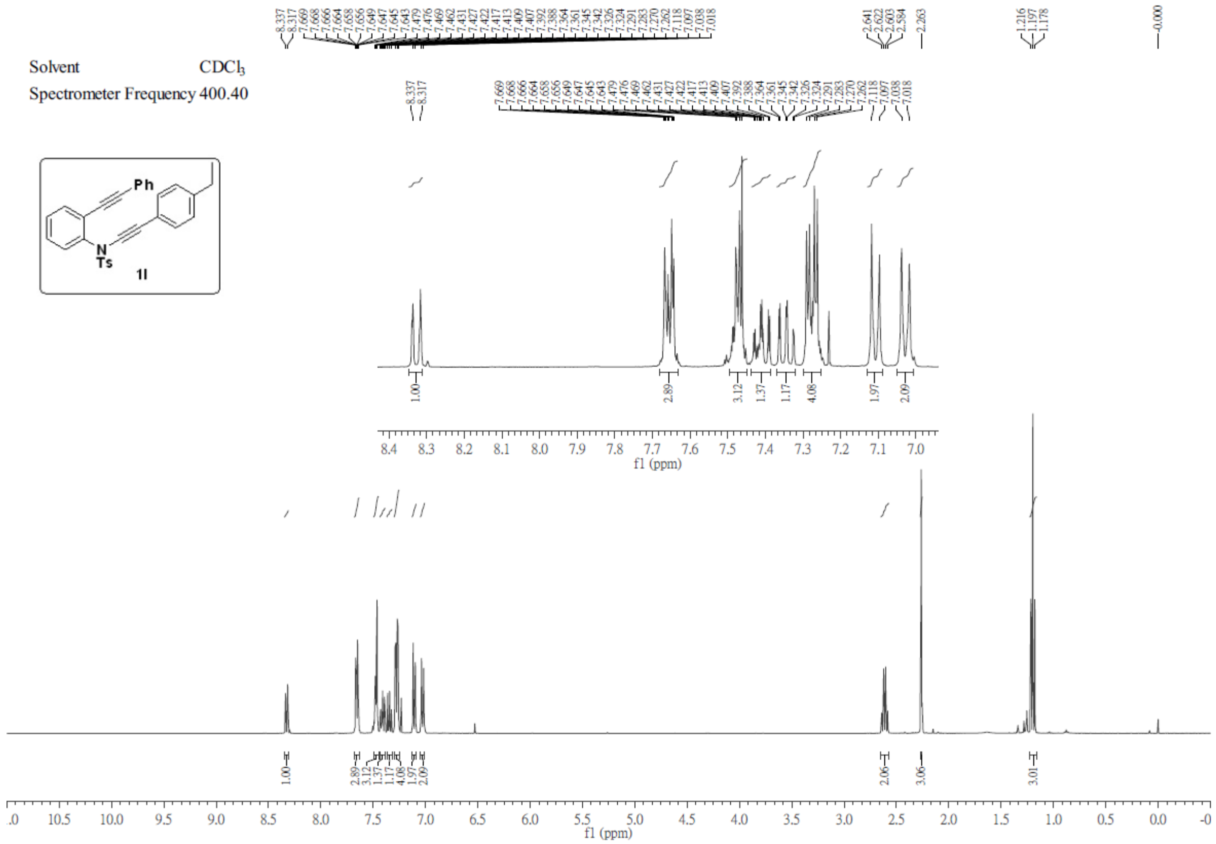

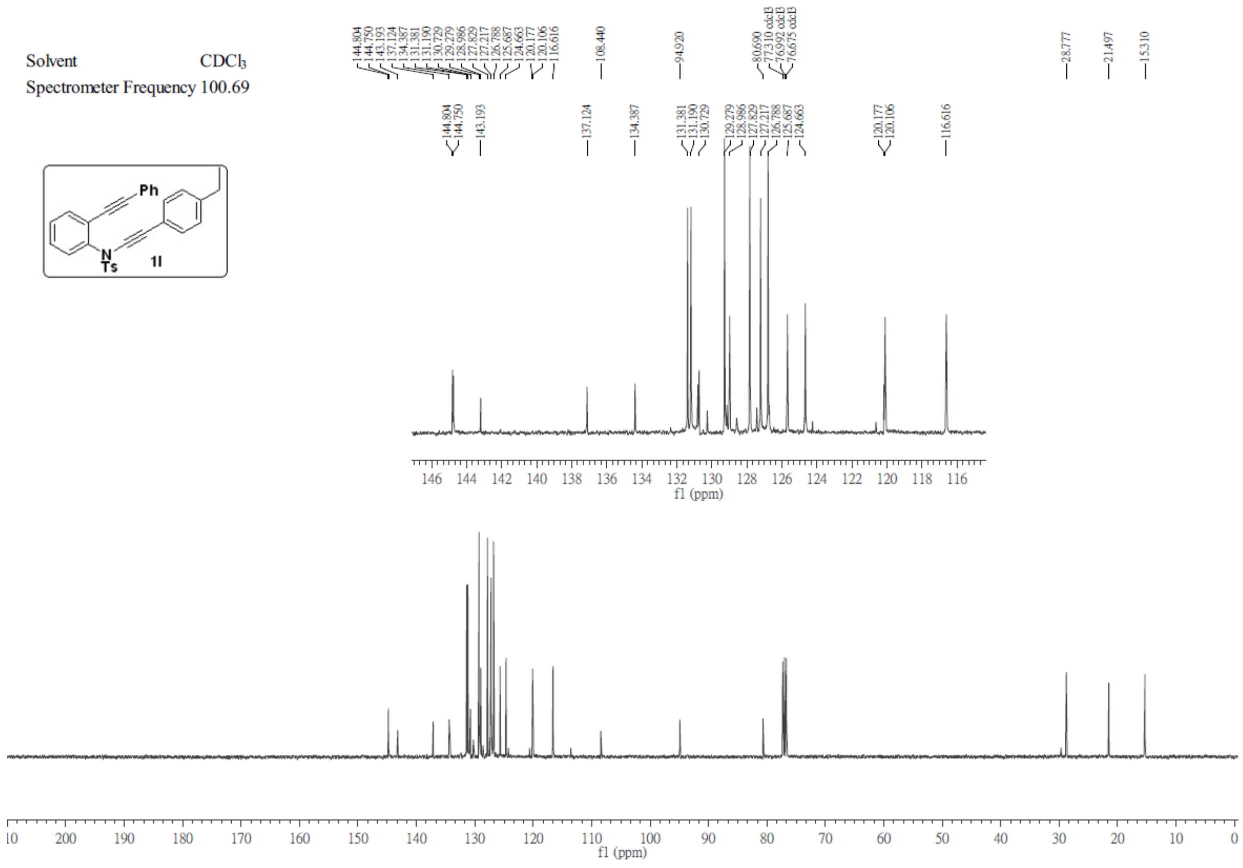

Supplementary Figure 67. ^{1}H (top) and ^{13}C (bottom) NMR spectra of compound **1l**.

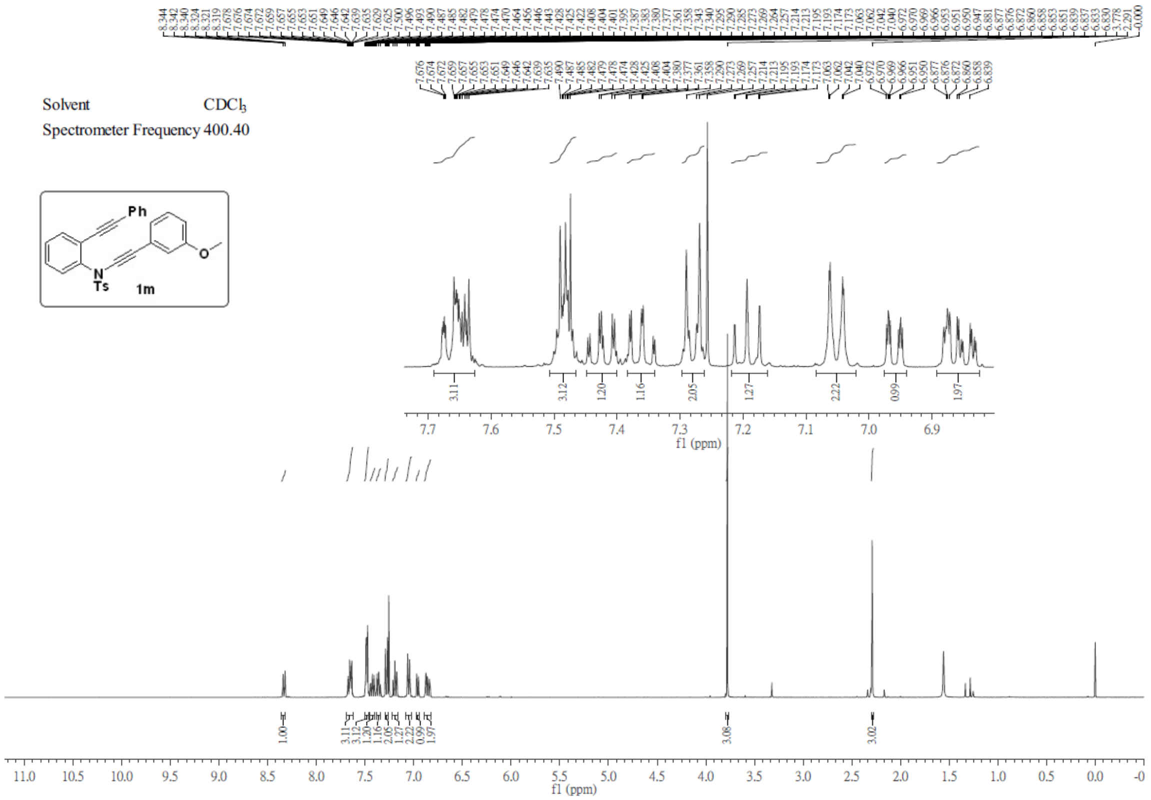

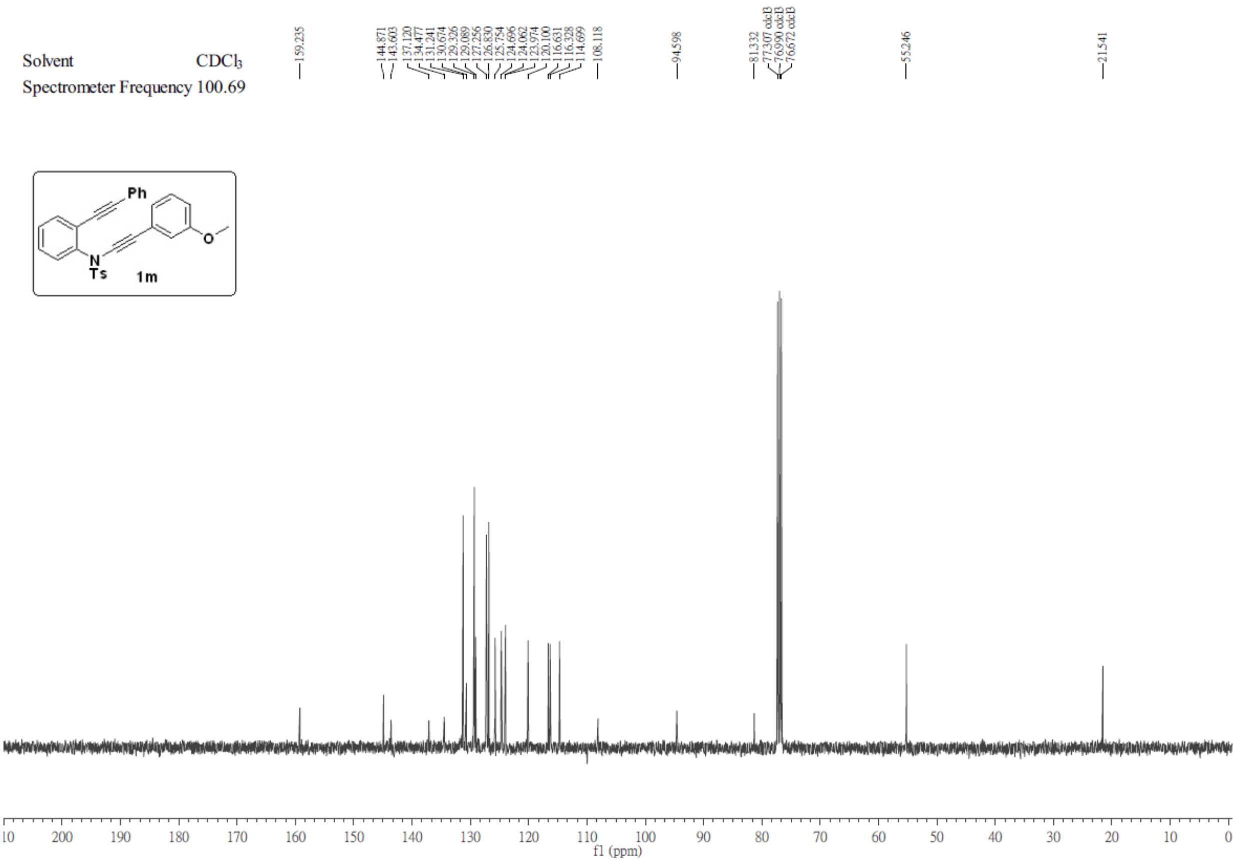

Supplementary Figure 68. ^{1}H (top) and ^{13}C (bottom) NMR spectra of compound **1m**.

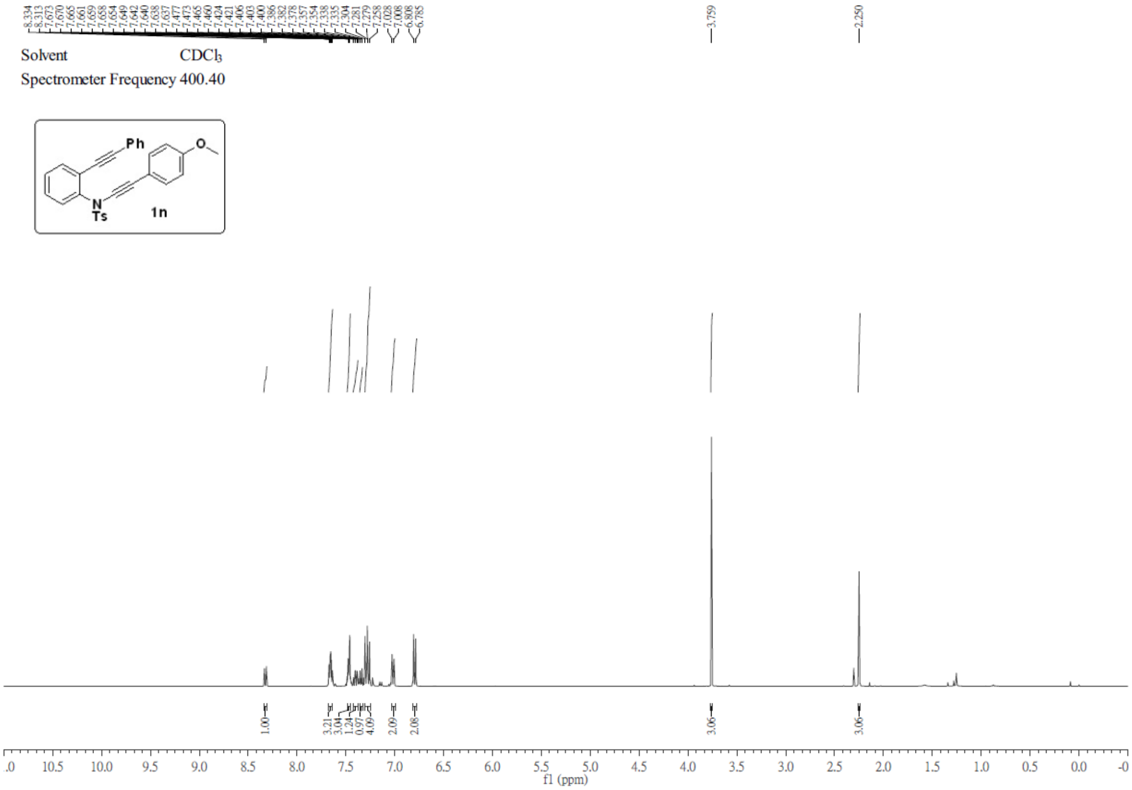

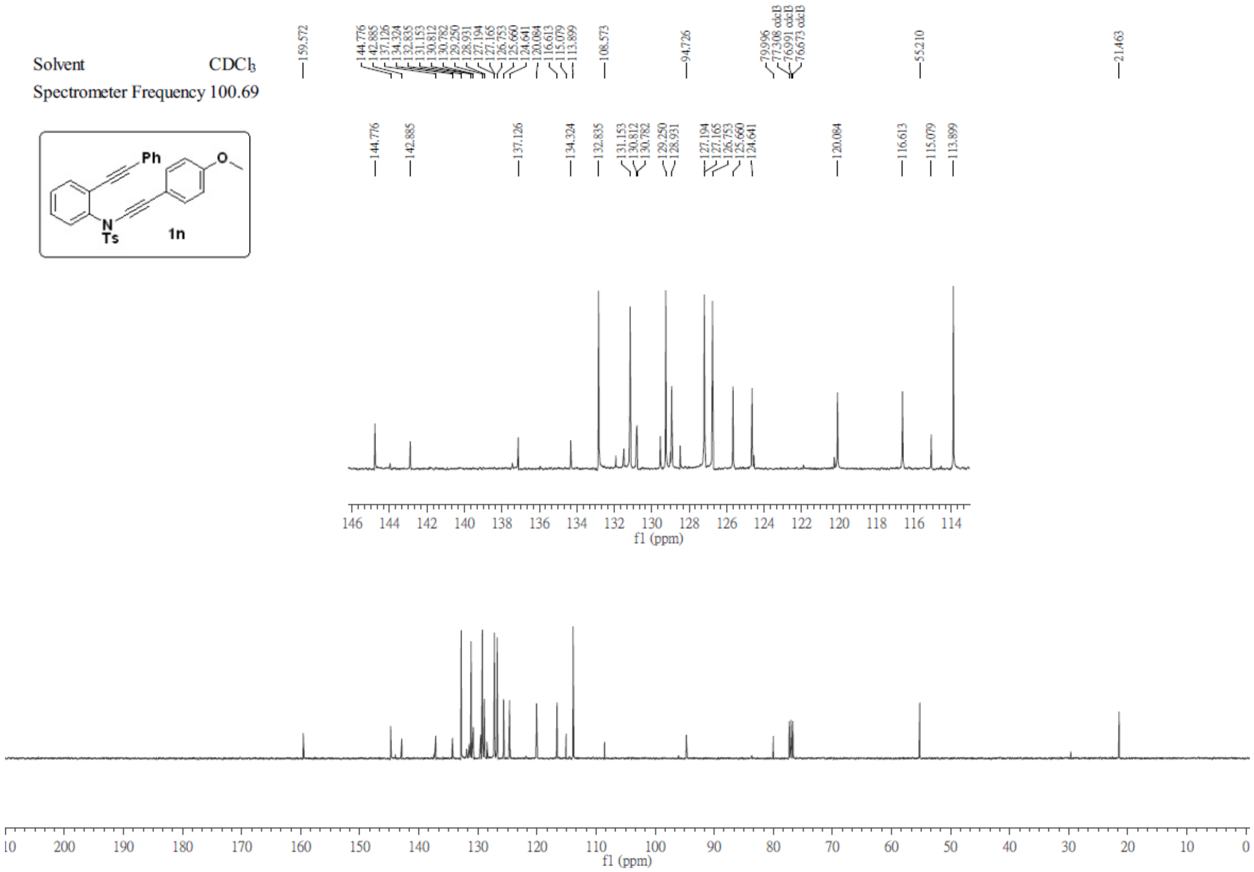

Supplementary Figure 69. ^{1}H (top) and ^{13}C (bottom) NMR spectra of compound **1n**.

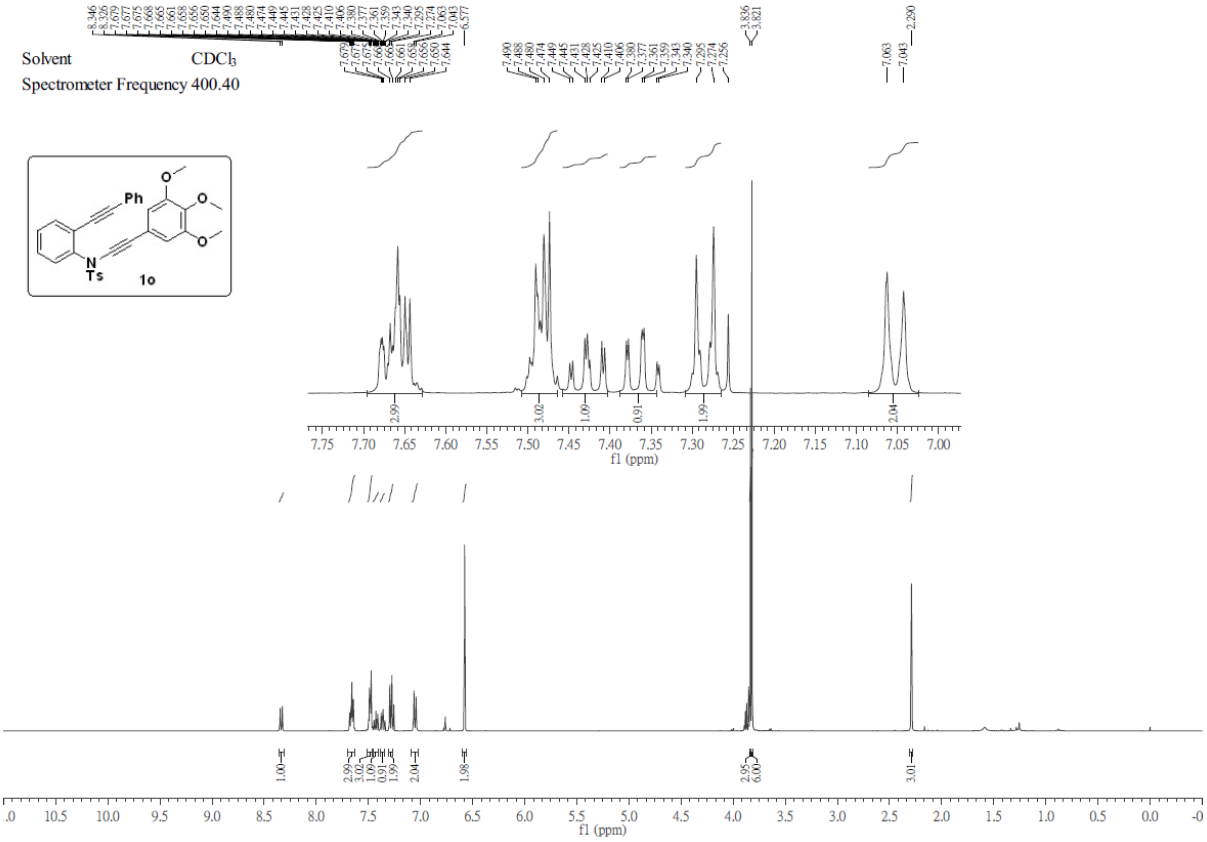

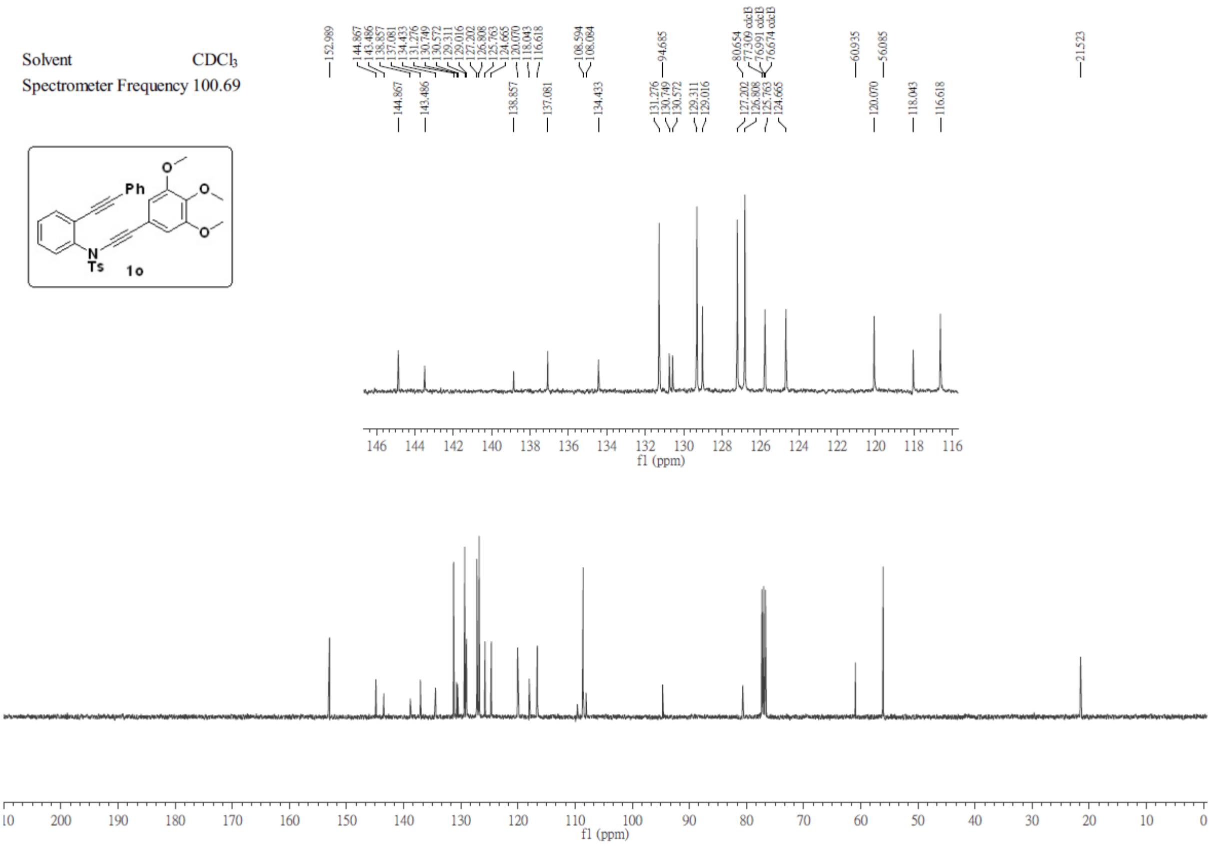

Supplementary Figure 70. ^{1}H (top) and ^{13}C (bottom) NMR spectra of compound **1o**.

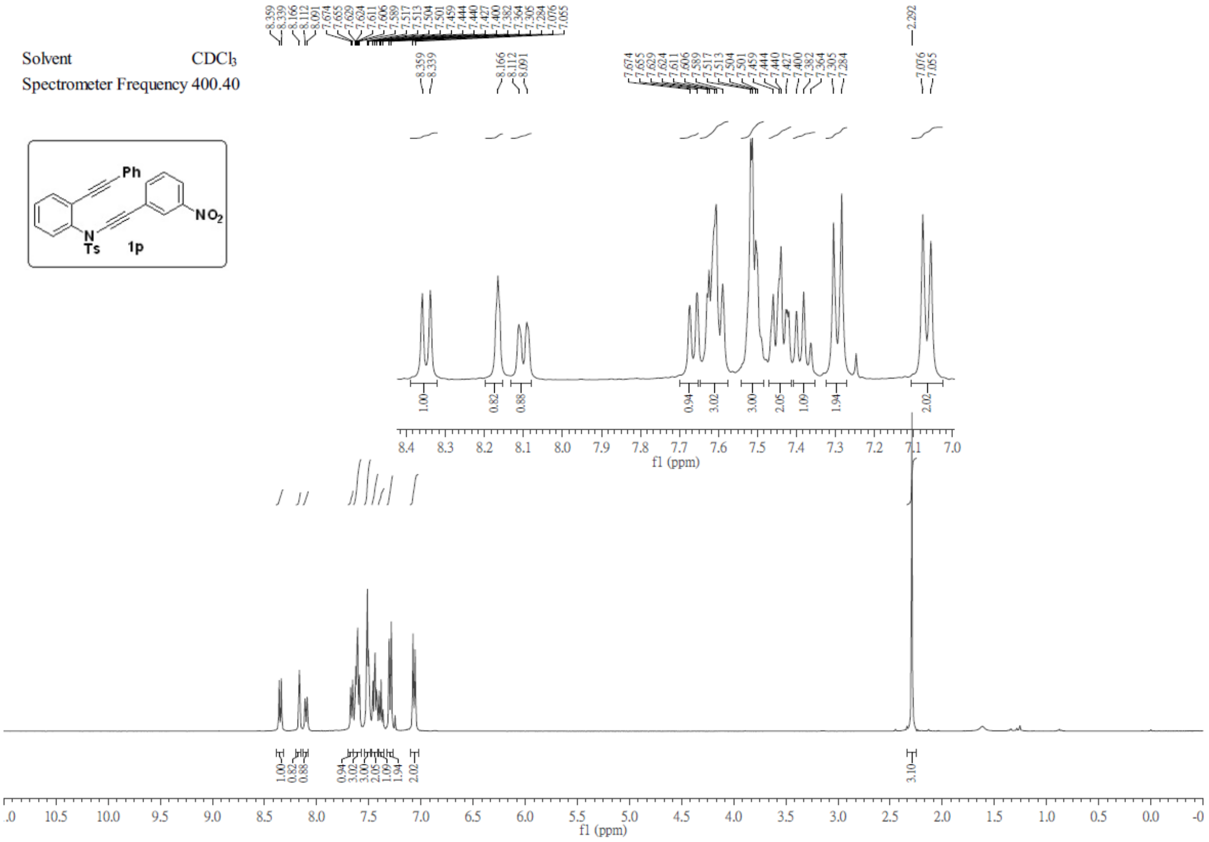

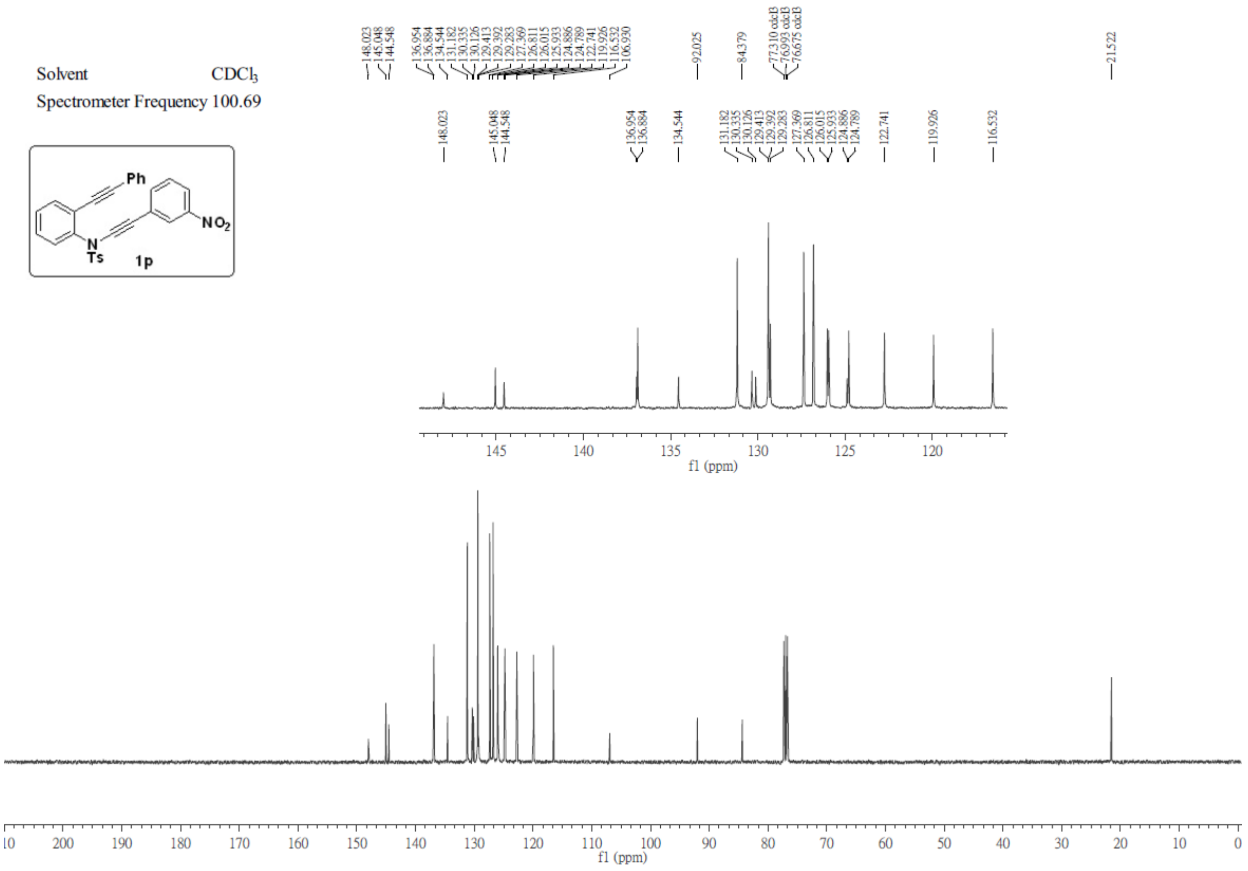

Supplementary Figure 71. ^{1}H (top) and ^{13}C (bottom) NMR spectra of compound **1p**.

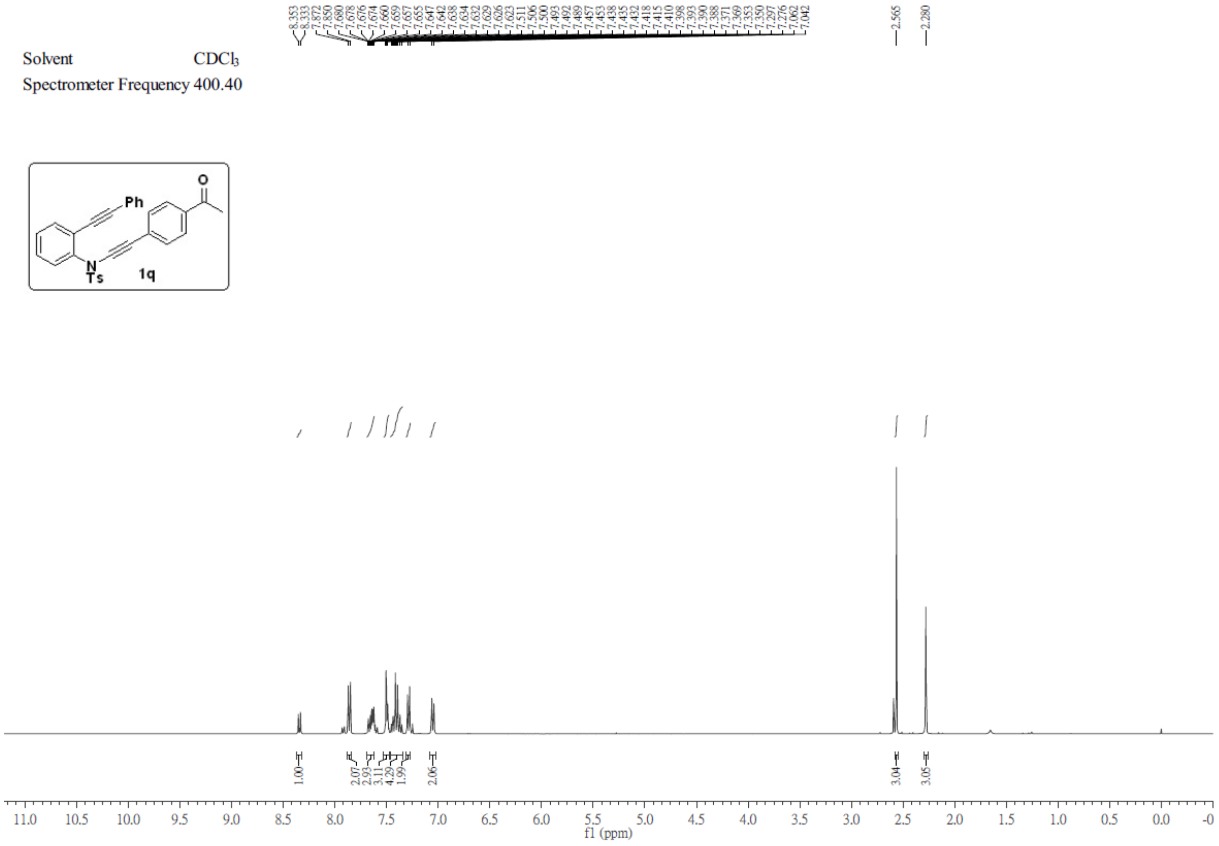

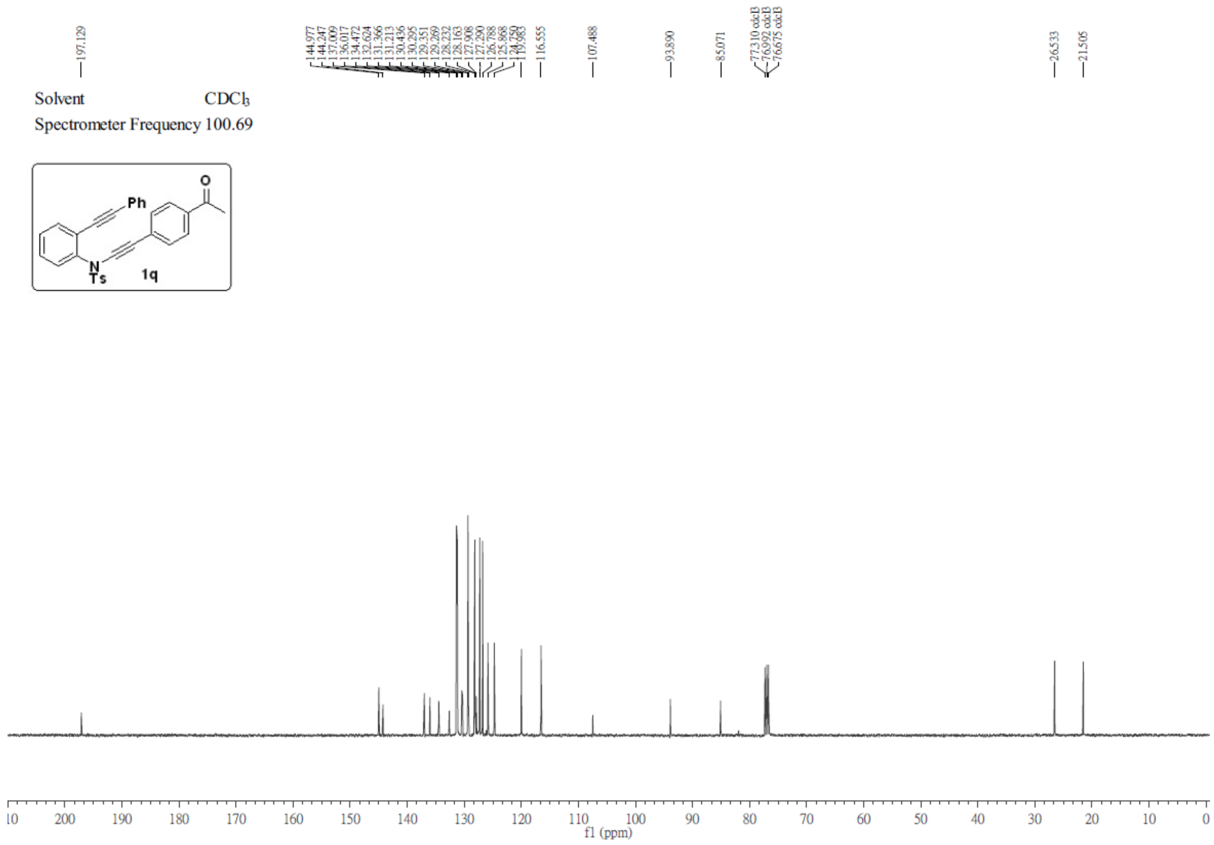

Supplementary Figure 72. ^{1}H (top) and ^{13}C (bottom) NMR spectra of compound **1q**.

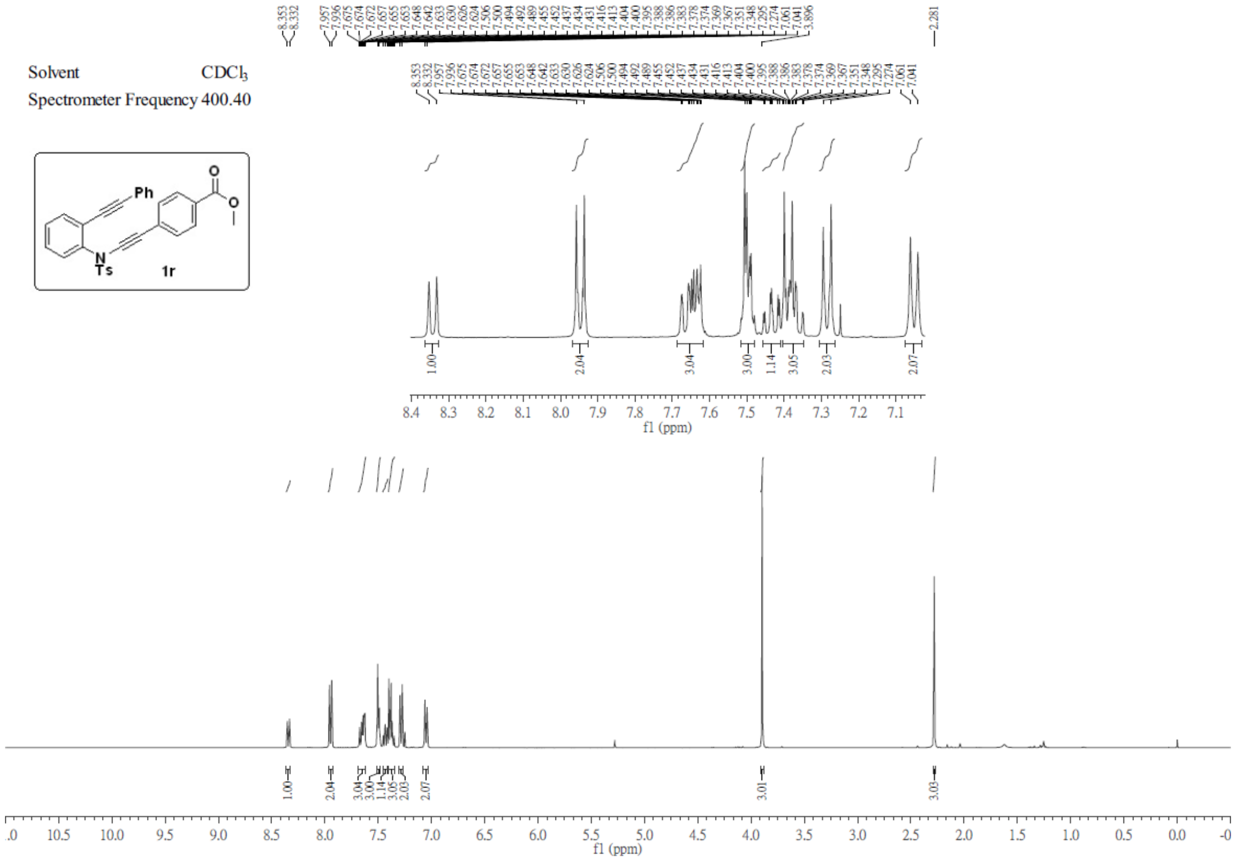

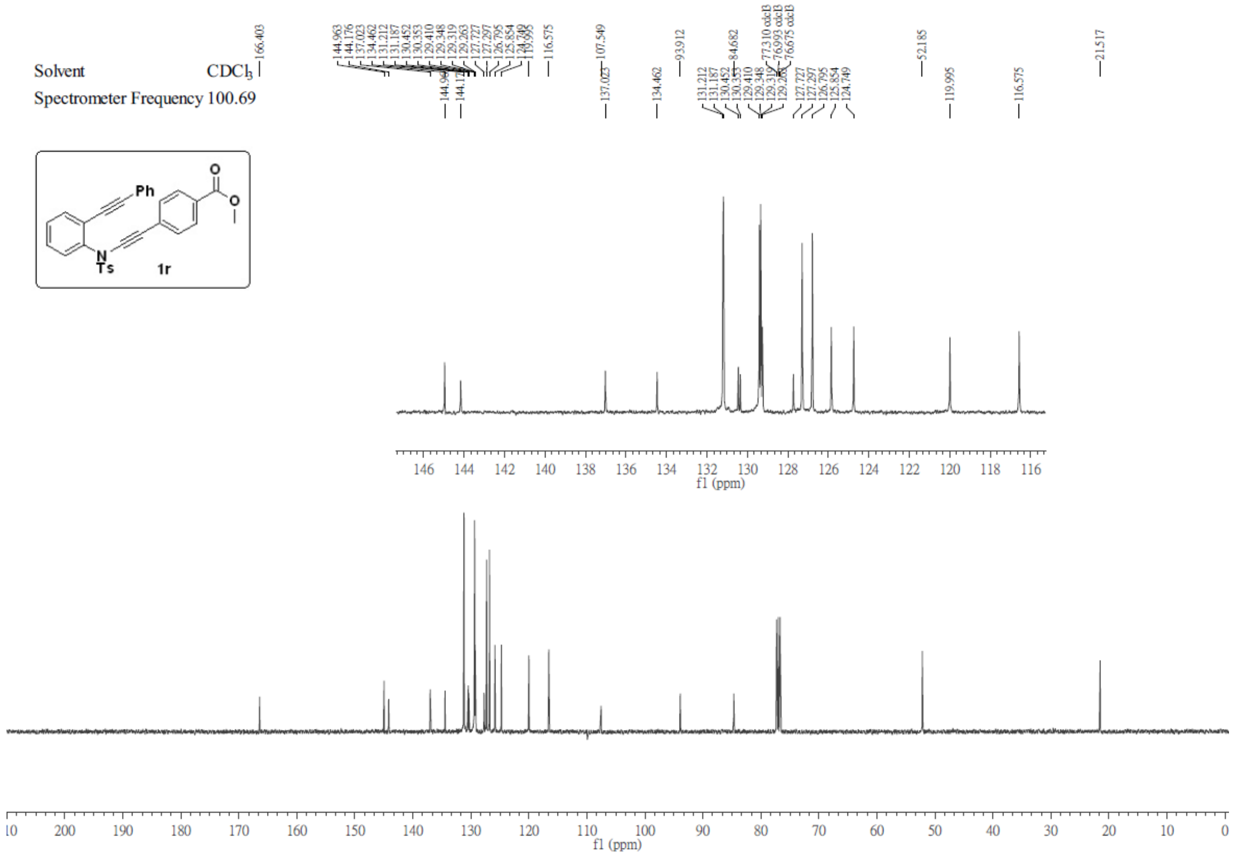

Supplementary Figure 73. ^{1}H (top) and ^{13}C (bottom) NMR spectra of compound **1r**.

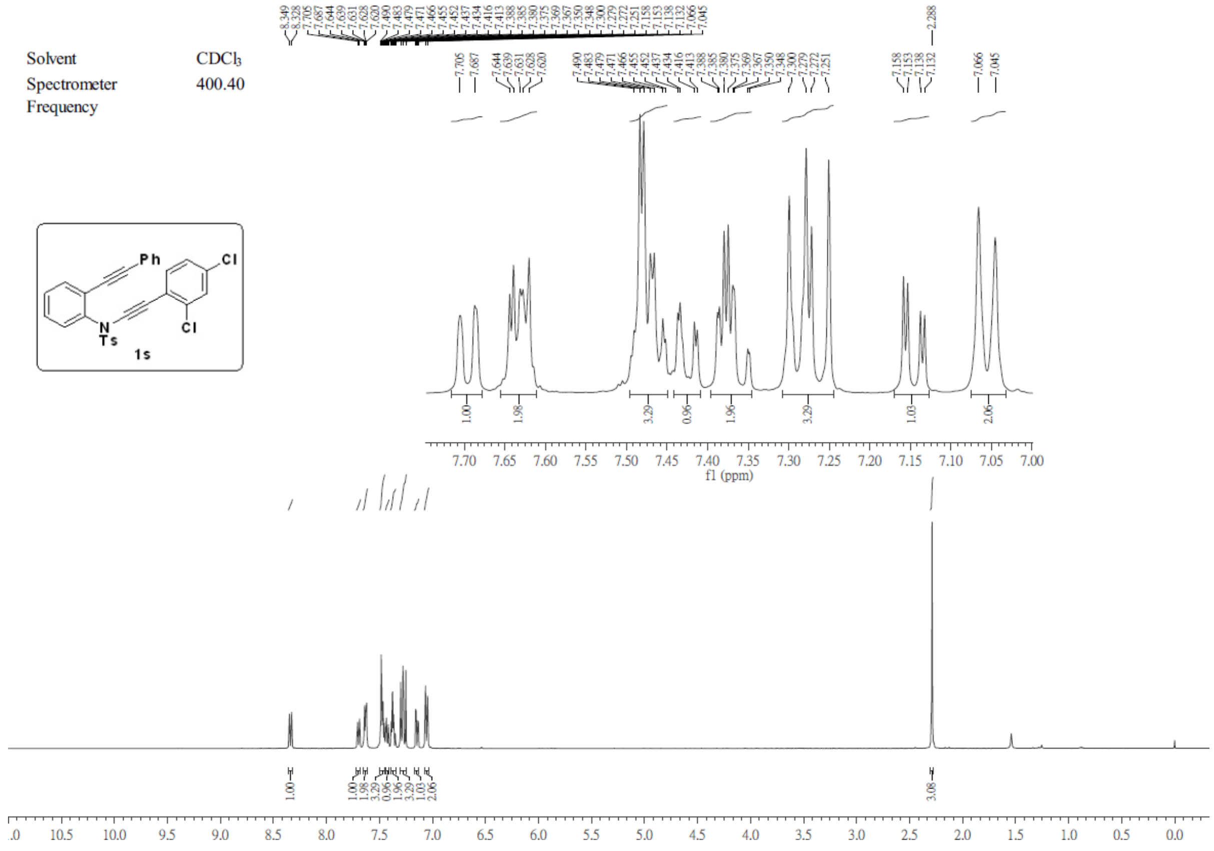

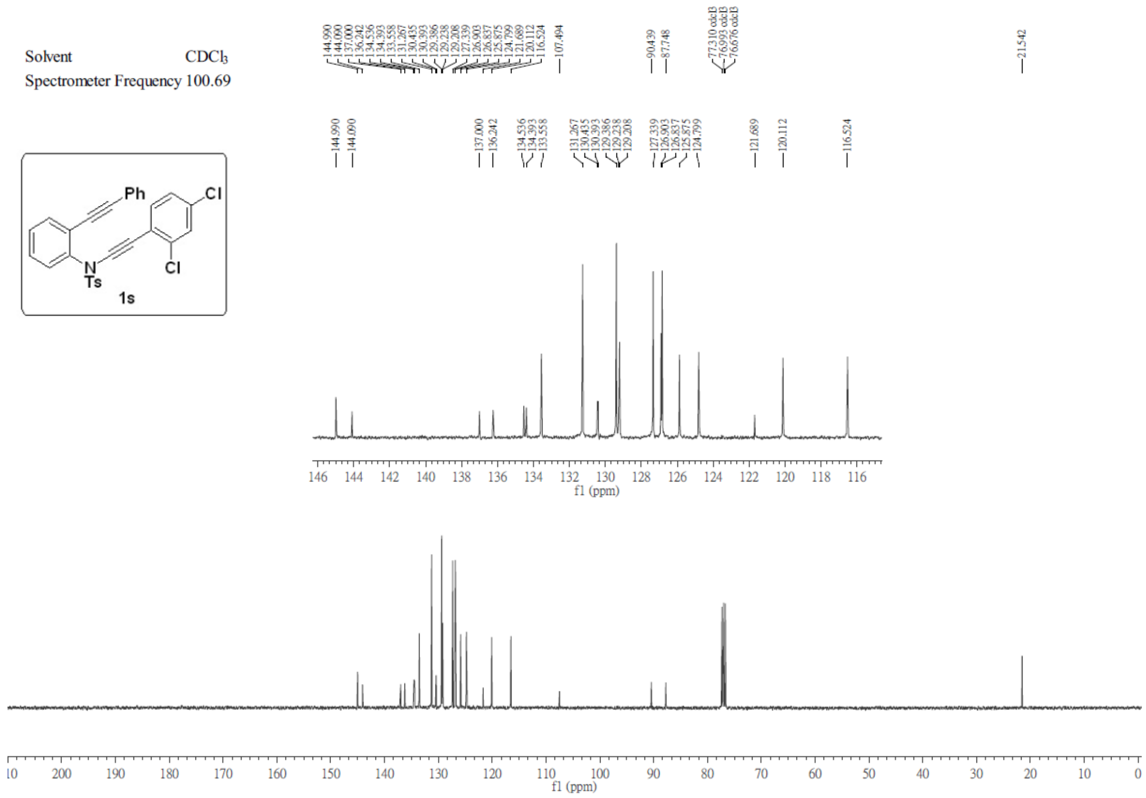

Supplementary Figure 74. ^{1}H (top) and ^{13}C (bottom) NMR spectra of compound **1s**.

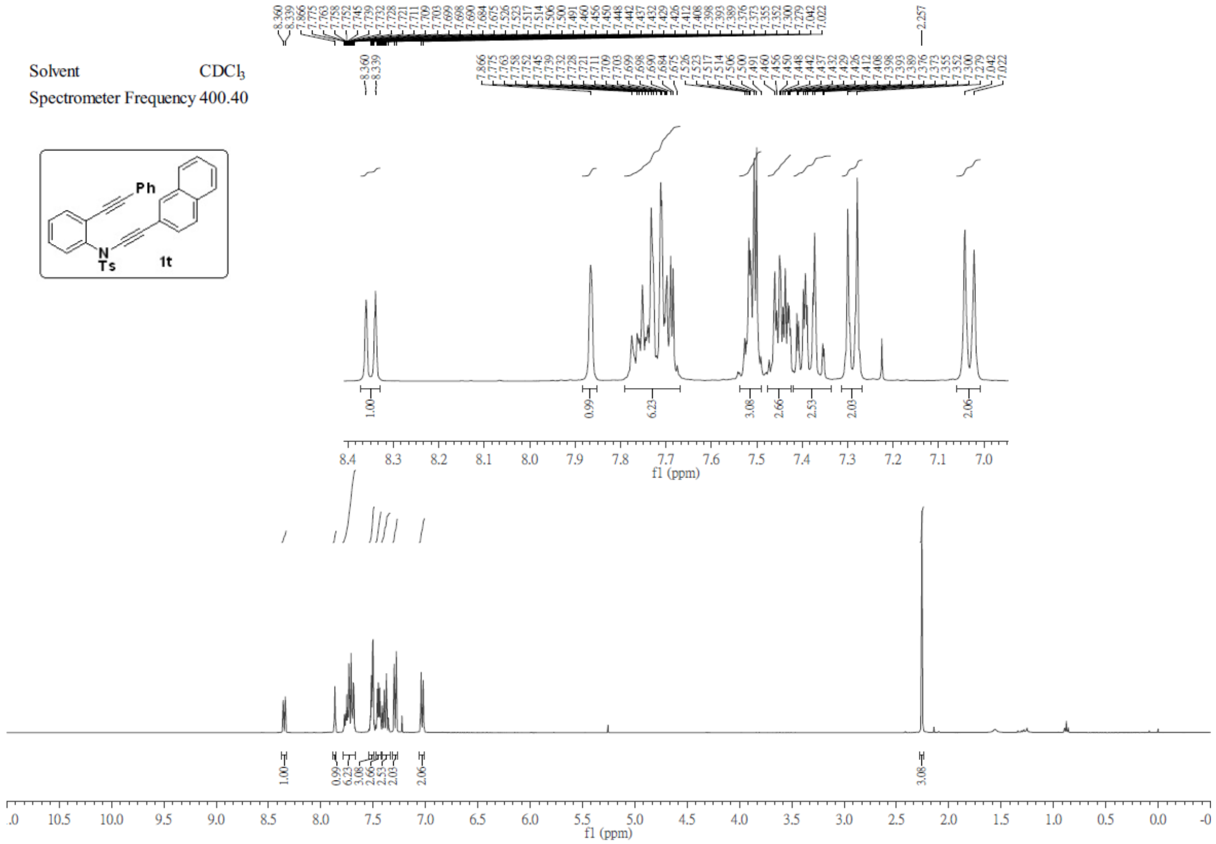

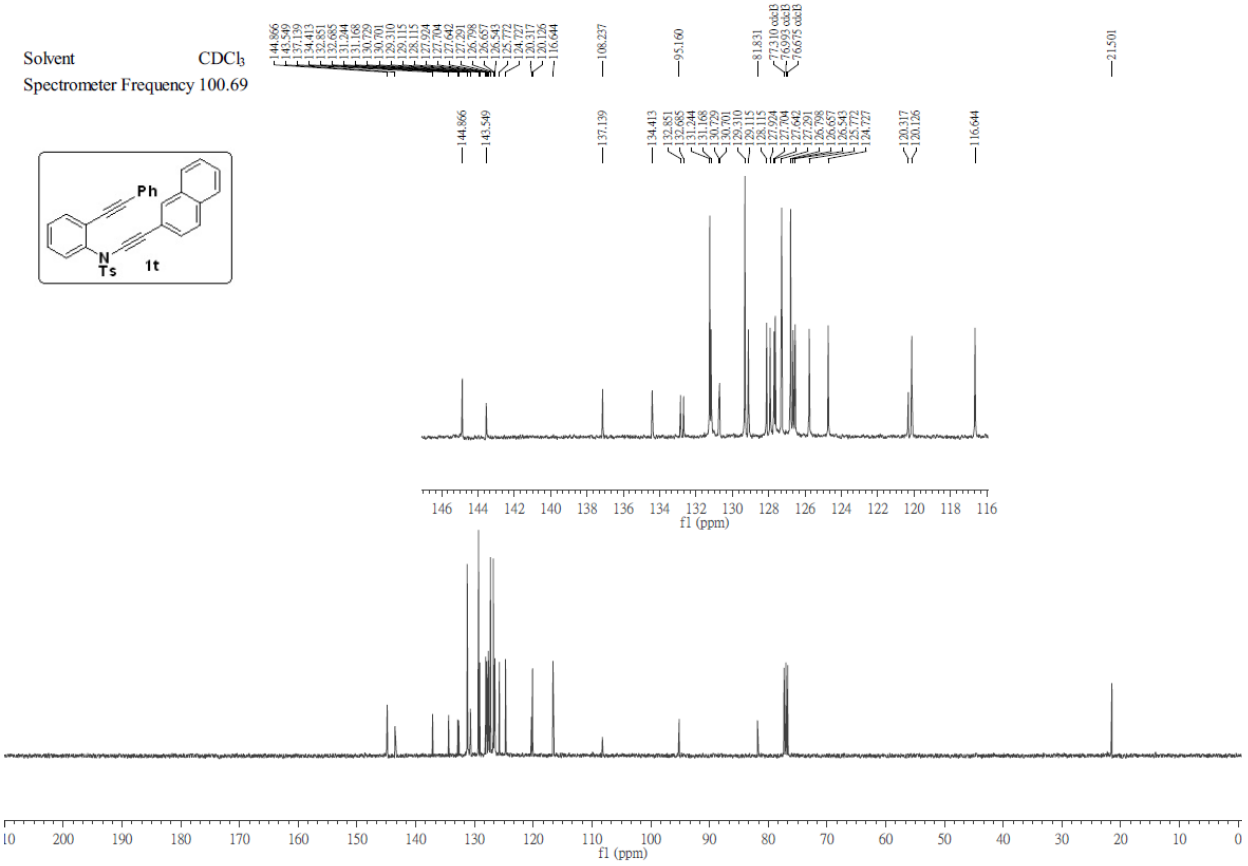

Supplementary Figure 75. ^{1}H (top) and ^{13}C (bottom) NMR spectra of compound **1t**.

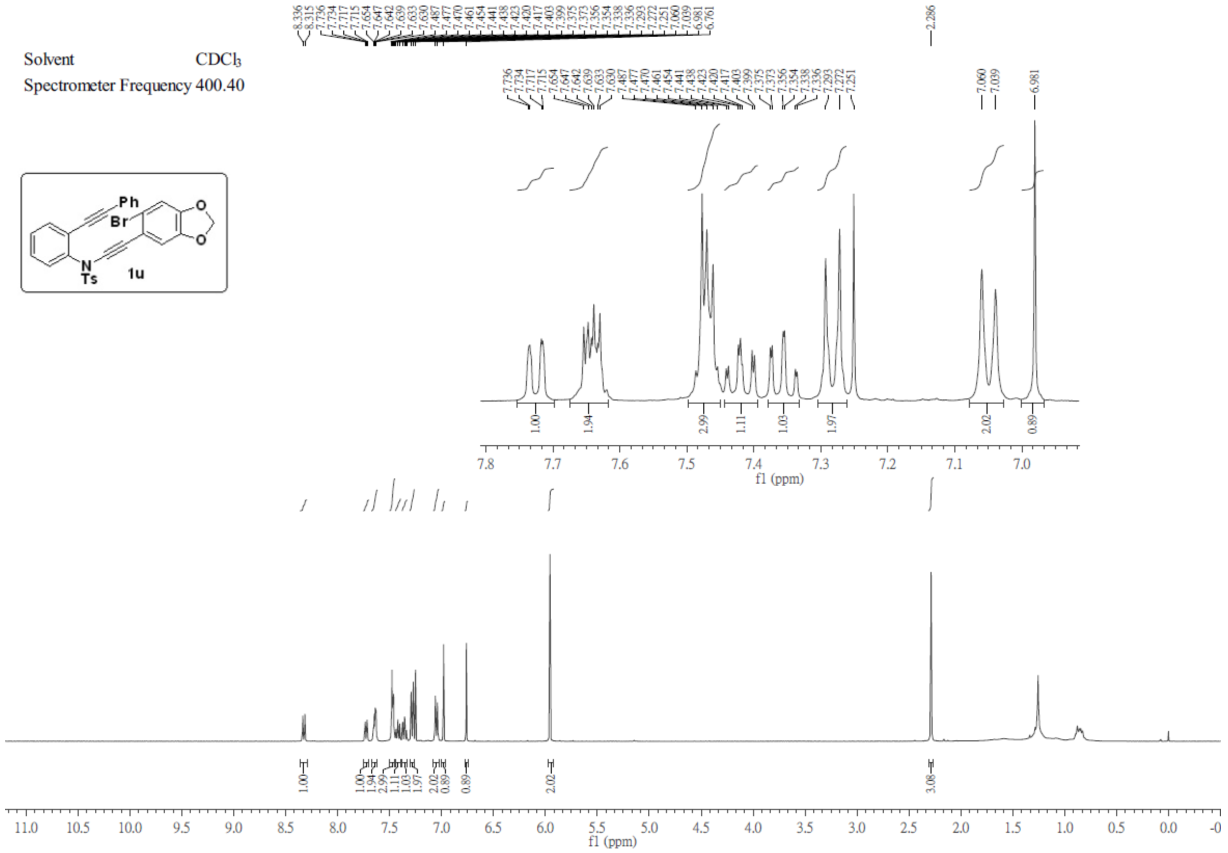

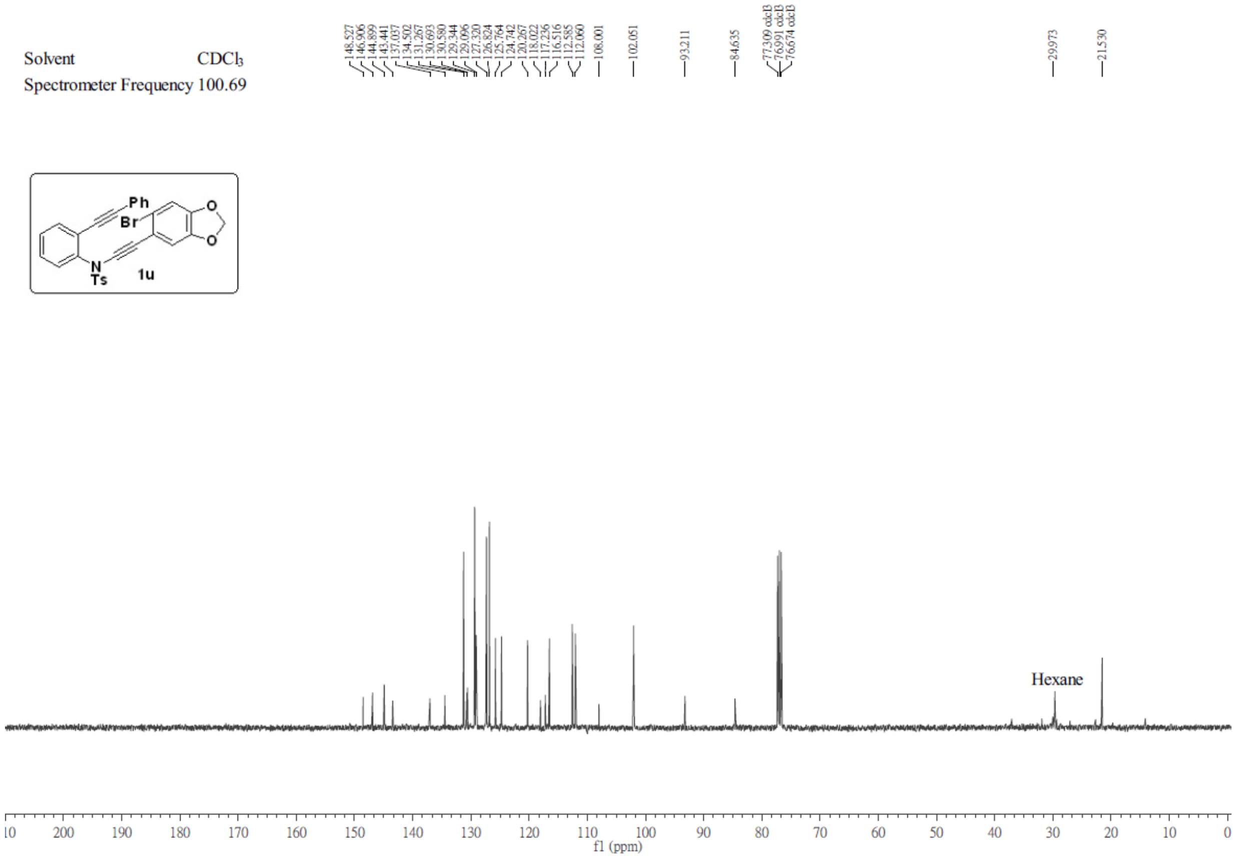

Supplementary Figure 76. ^{1}H (top) and ^{13}C (bottom) NMR spectra of compound **1u**.

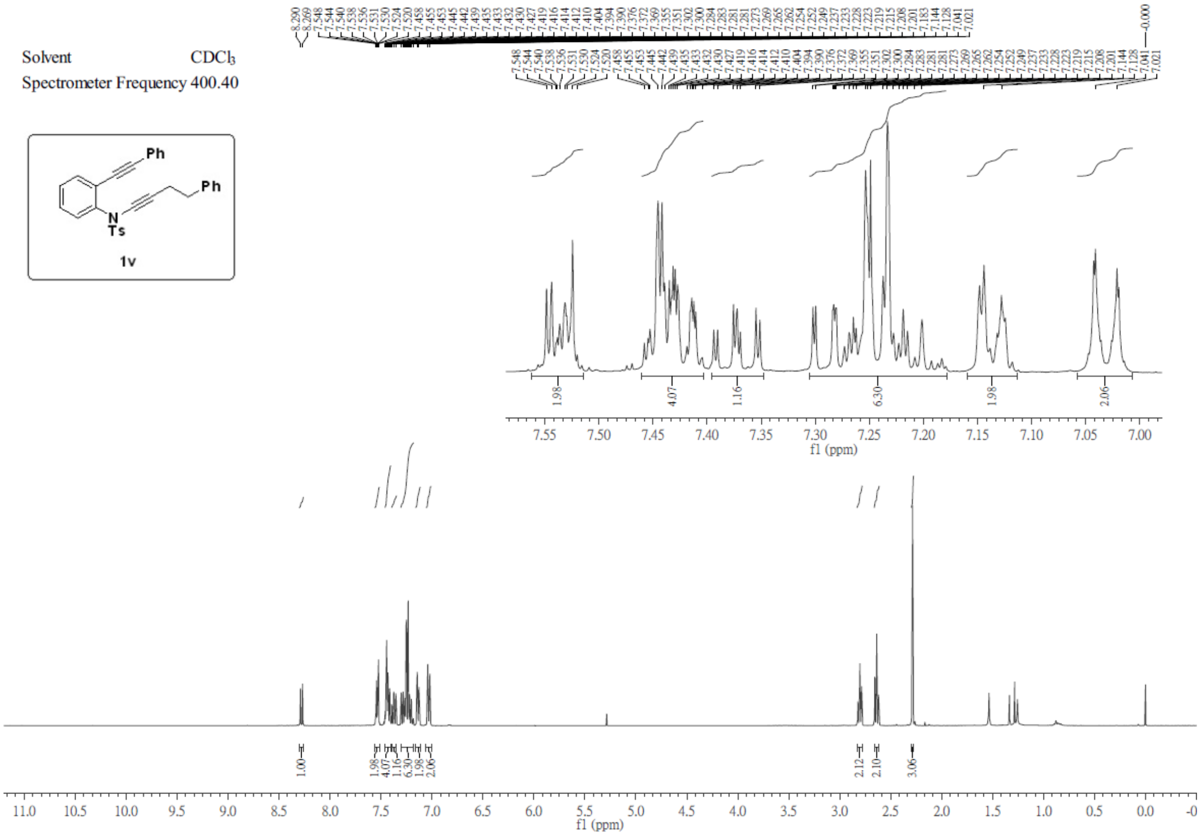

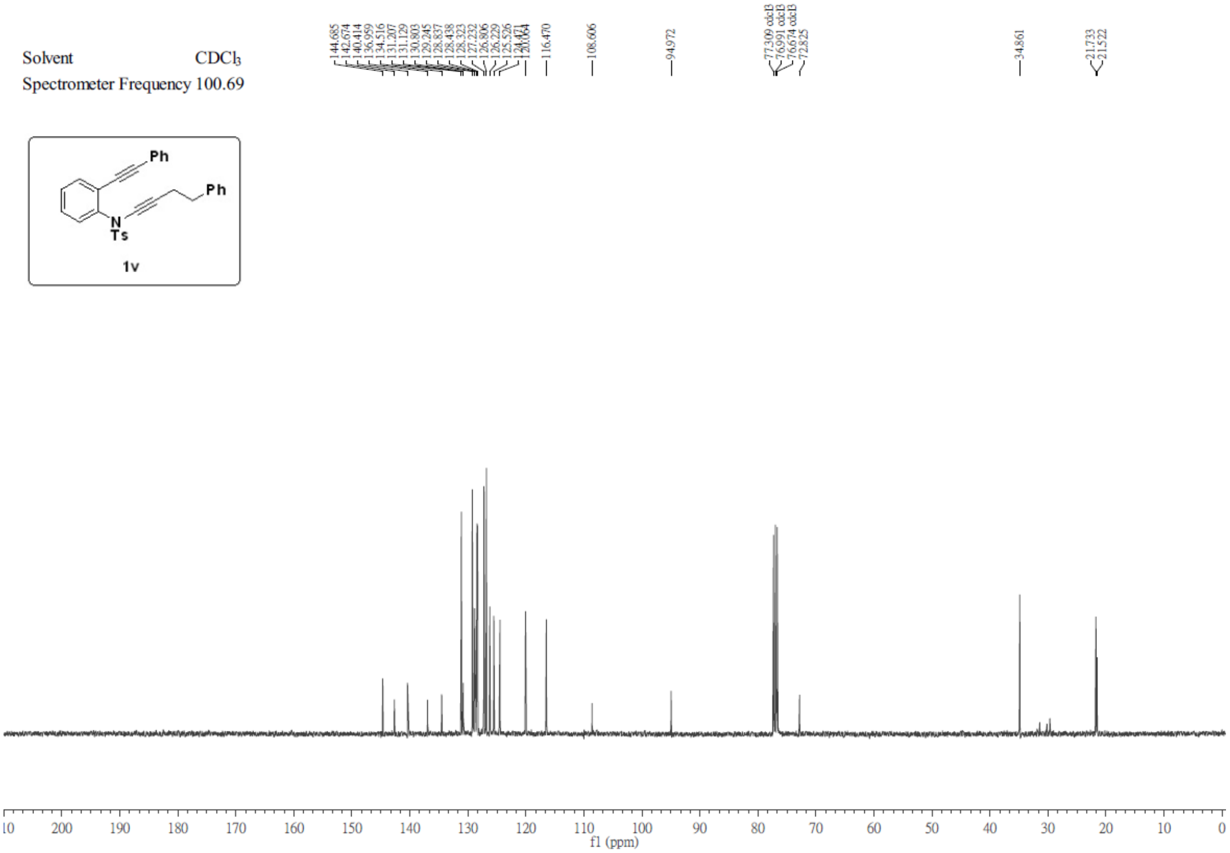

Supplementary Figure 77. ^{1}H (top) and ^{13}C (bottom) NMR spectra of compound **1v**.

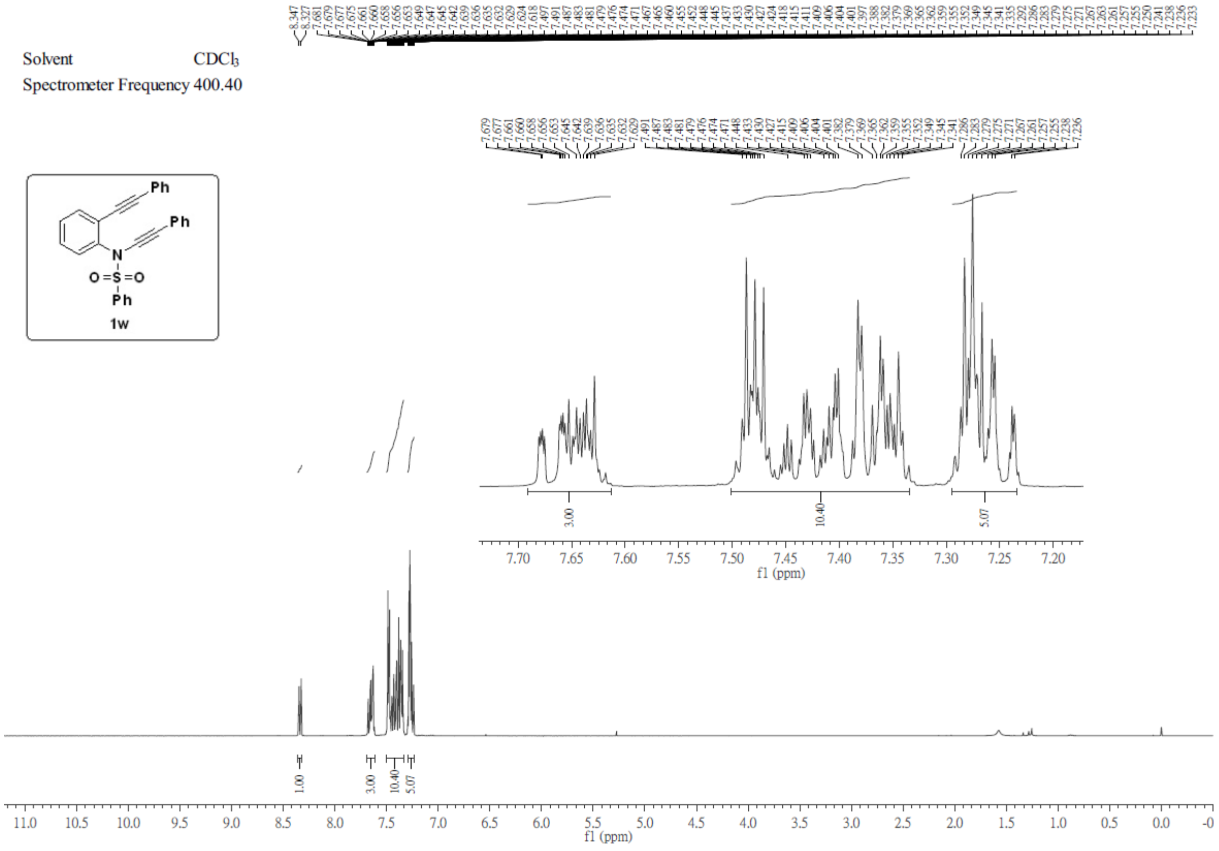

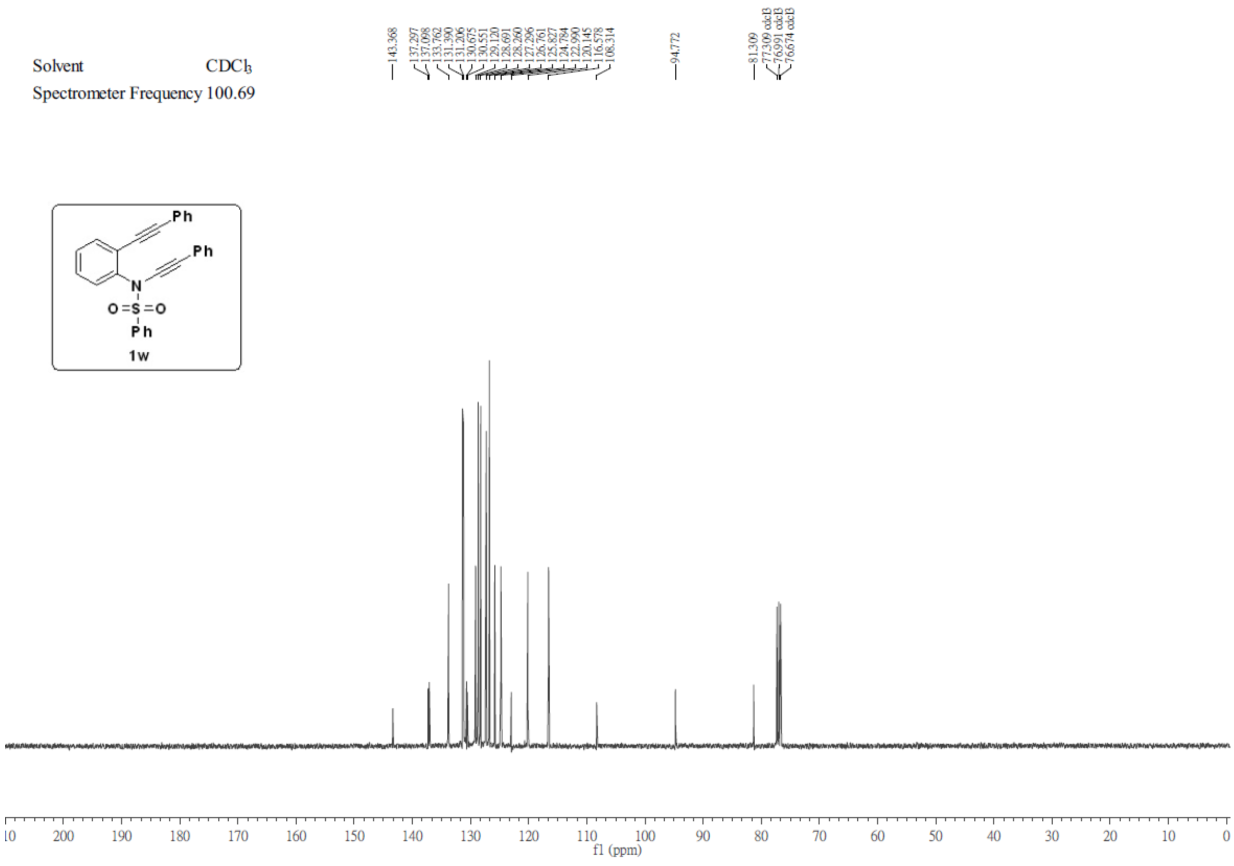

Supplementary Figure 78. ^{1}H (top) and ^{13}C (bottom) NMR spectra of compound **1w**.

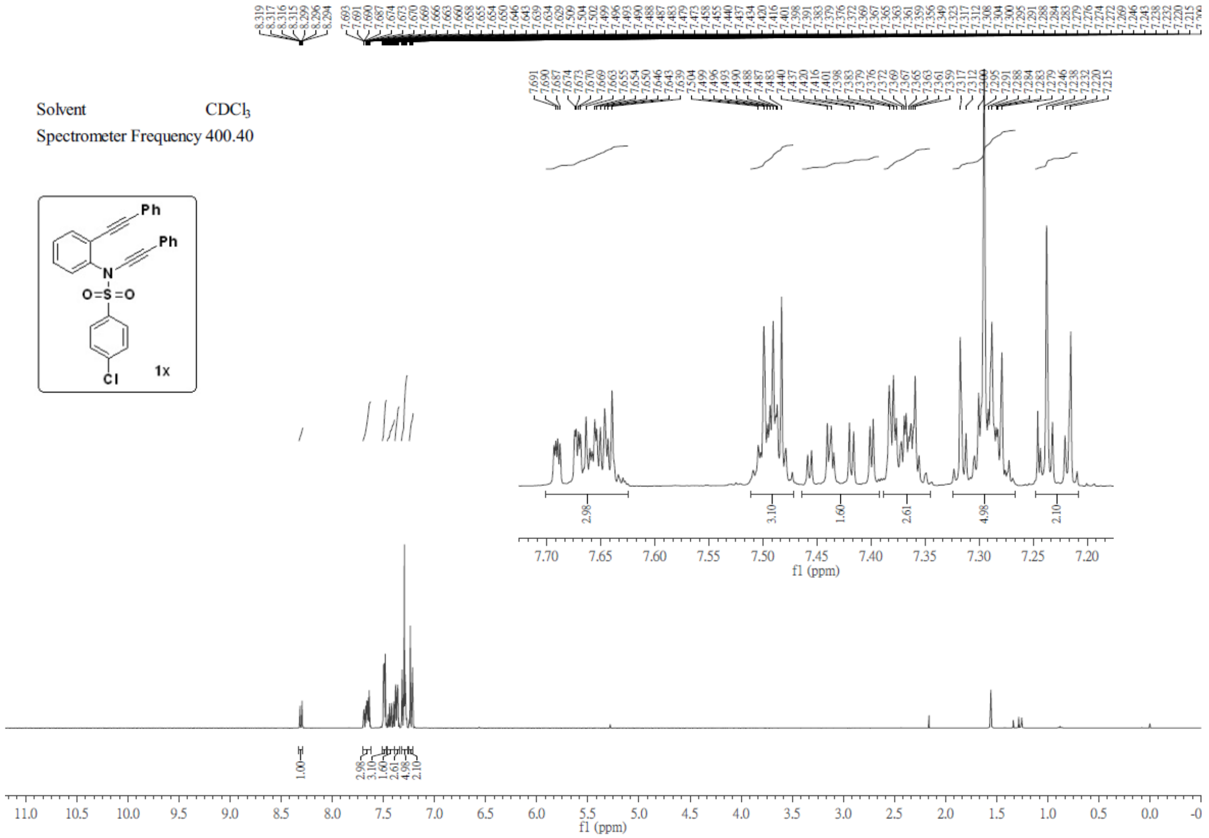

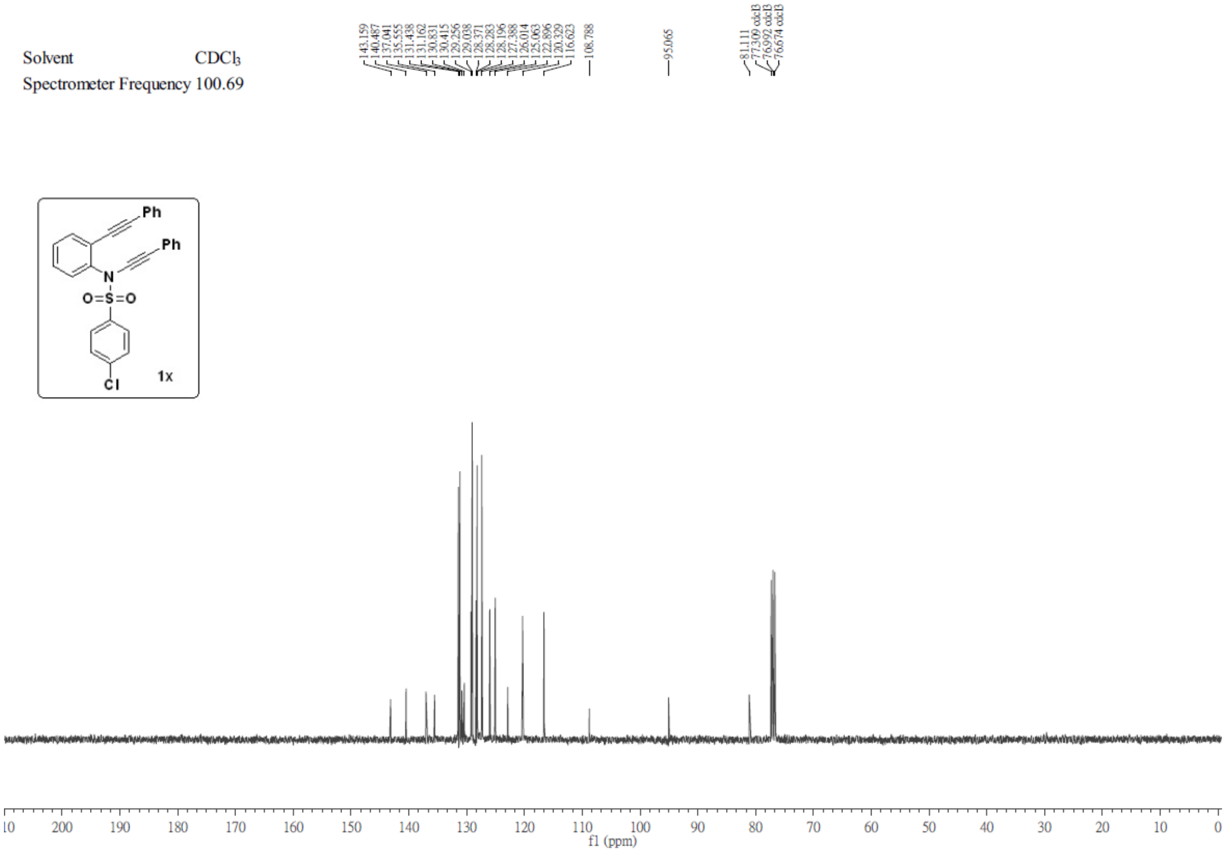

Supplementary Figure 79. ^{1}H (top) and ^{13}C (bottom) NMR spectra of compound **1x**.

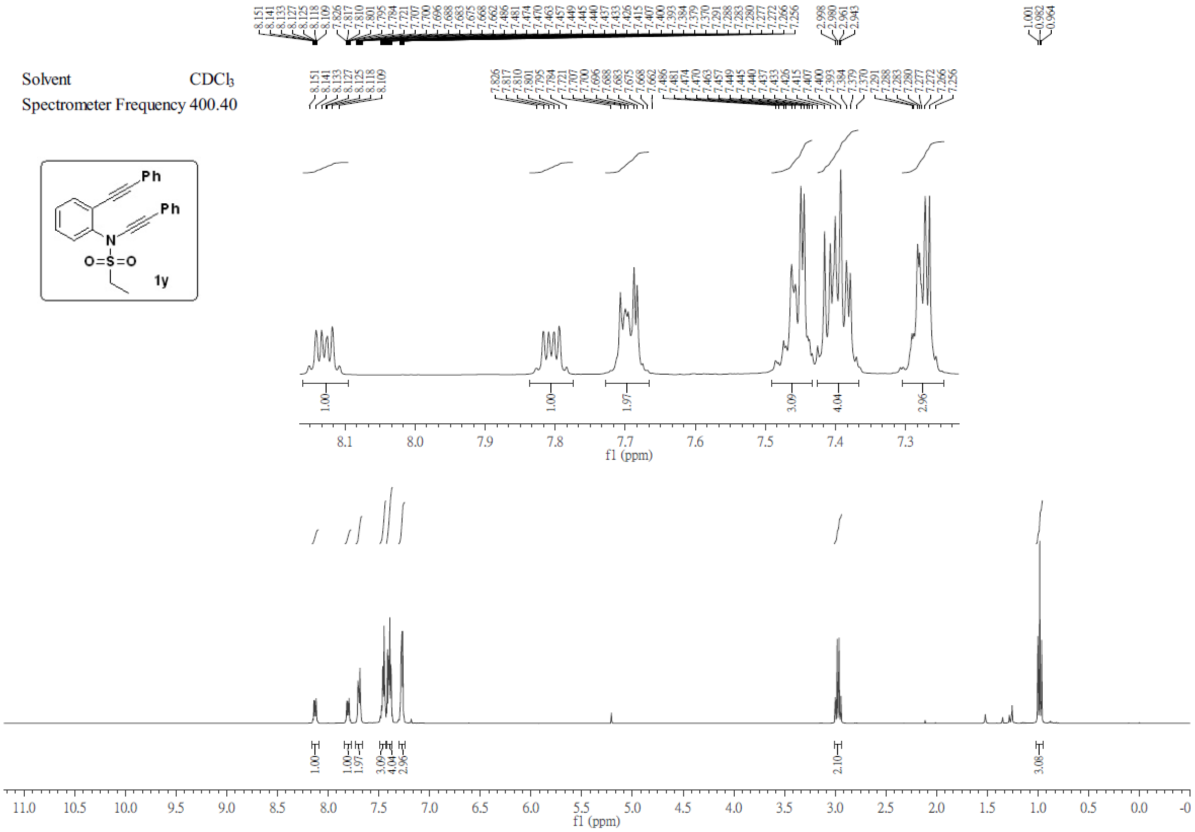

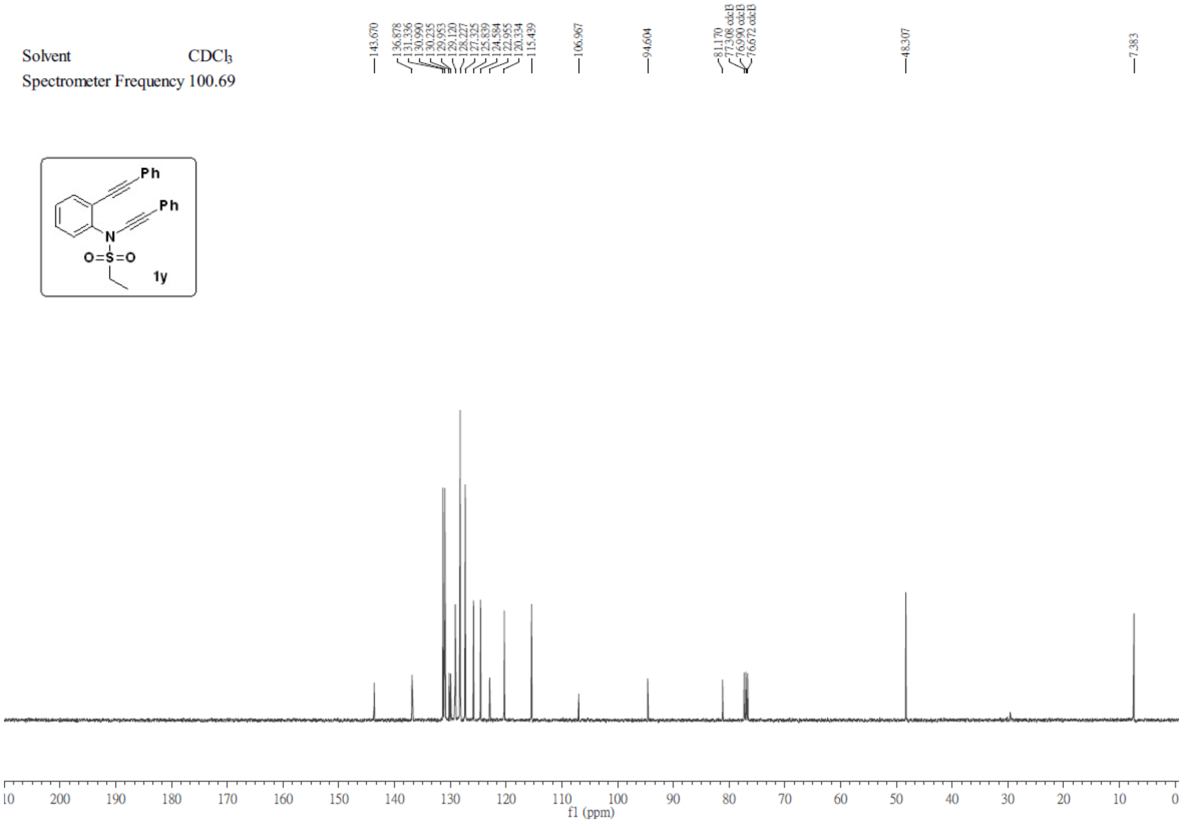

Supplementary Figure 80. ^{1}H (top) and ^{13}C (bottom) NMR spectra of compound **1y**.

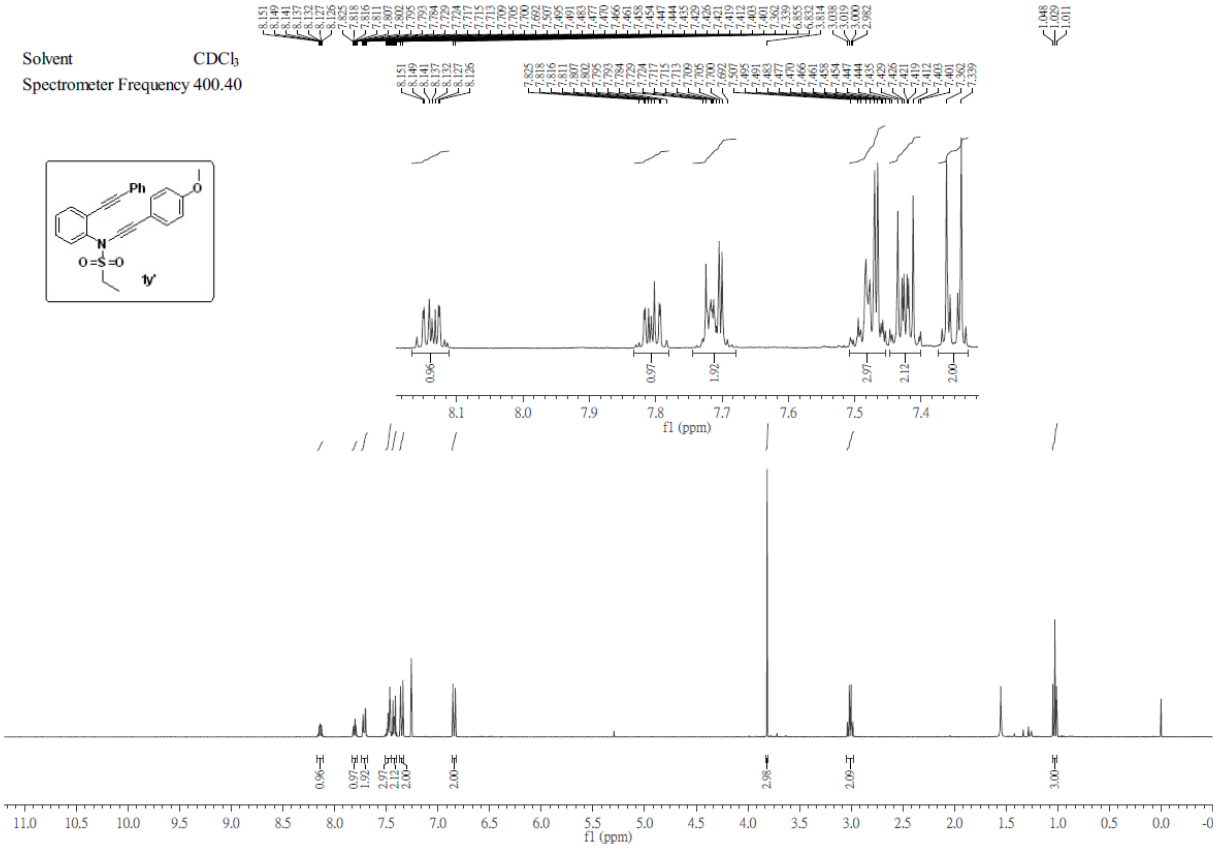

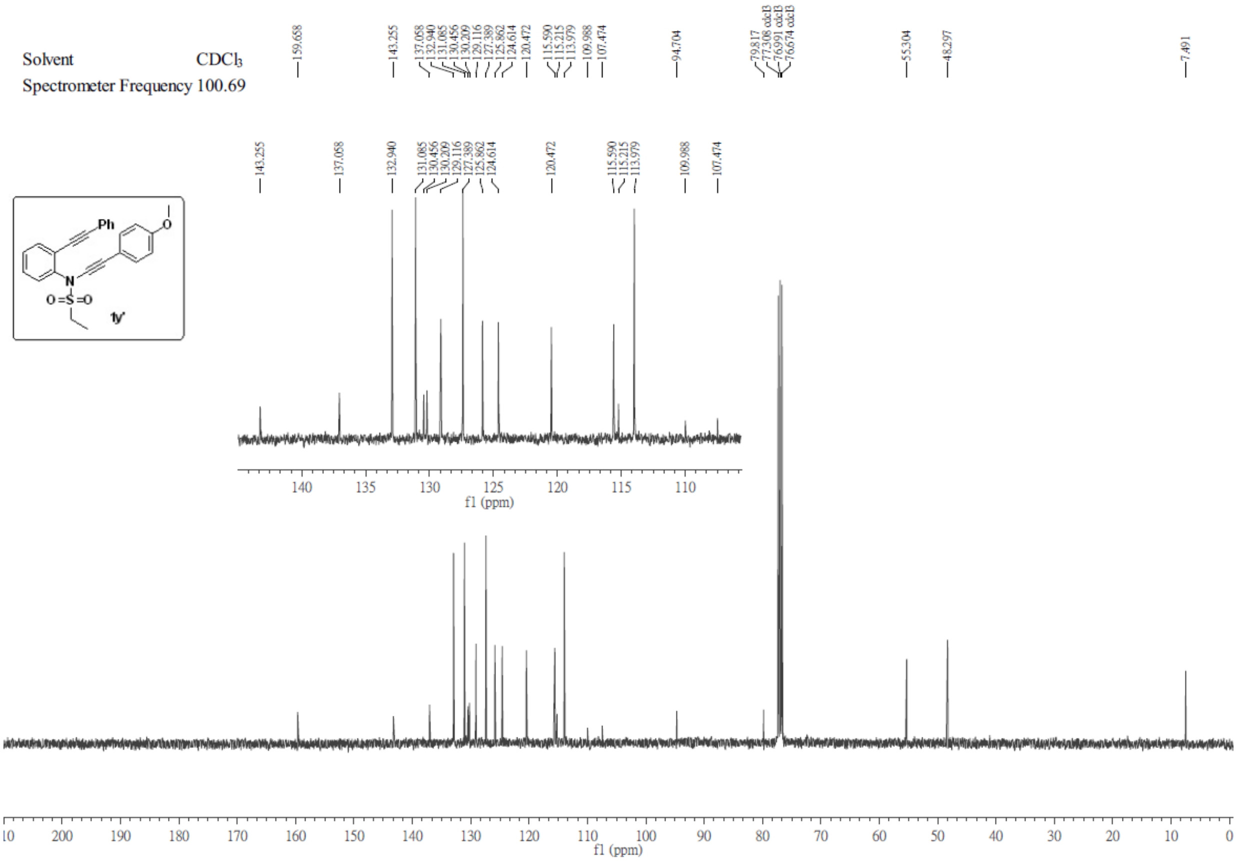

Supplementary Figure 81. ^{1}H (top) and ^{13}C (bottom) NMR spectra of compound **1y’** .

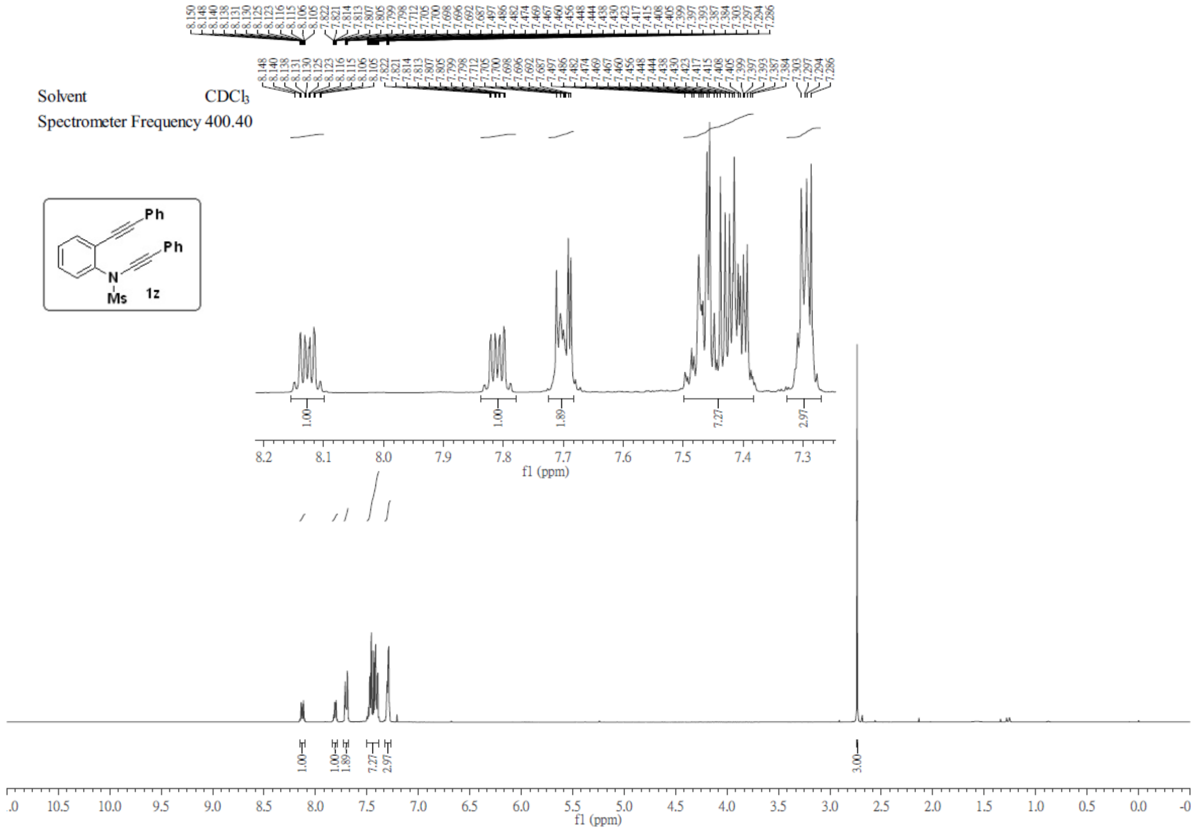

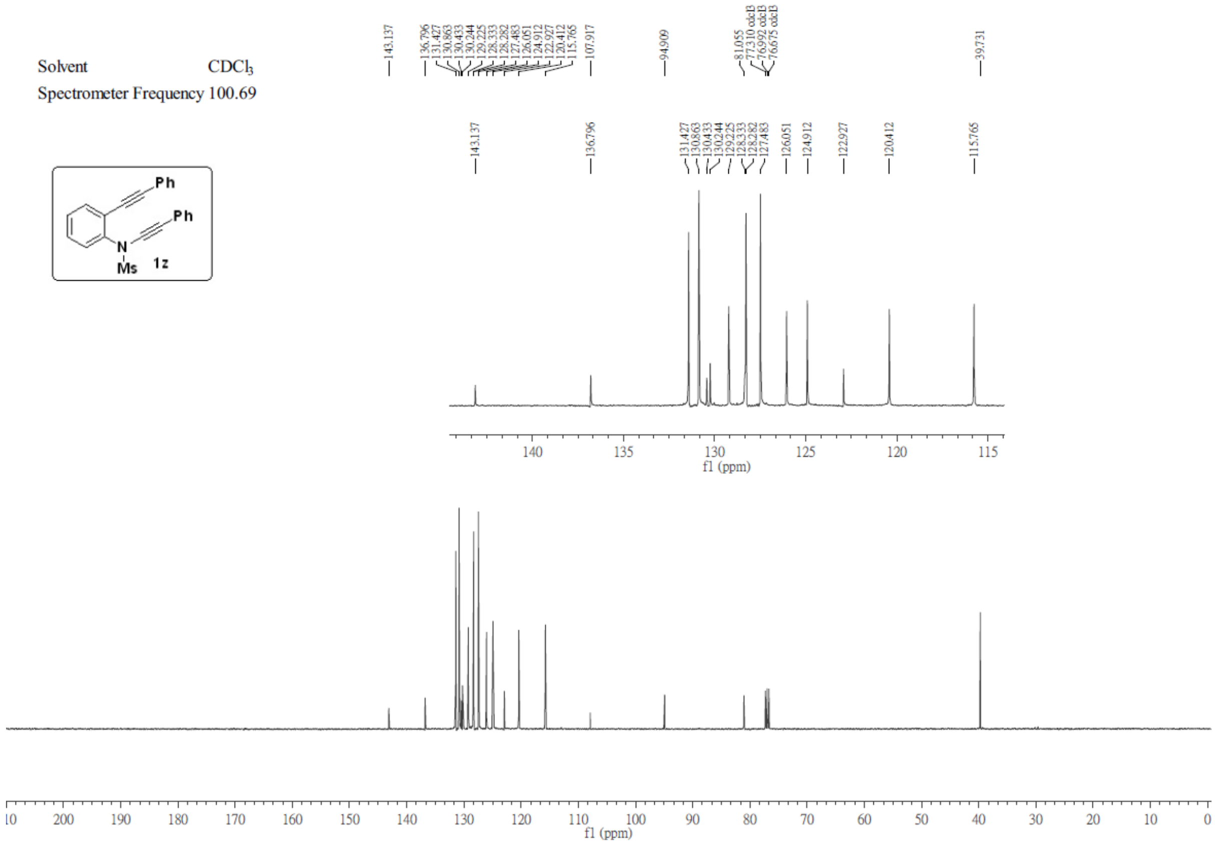

Supplementary Figure 82. ^{1}H (top) and ^{13}C (bottom) NMR spectra of compound **1z**.

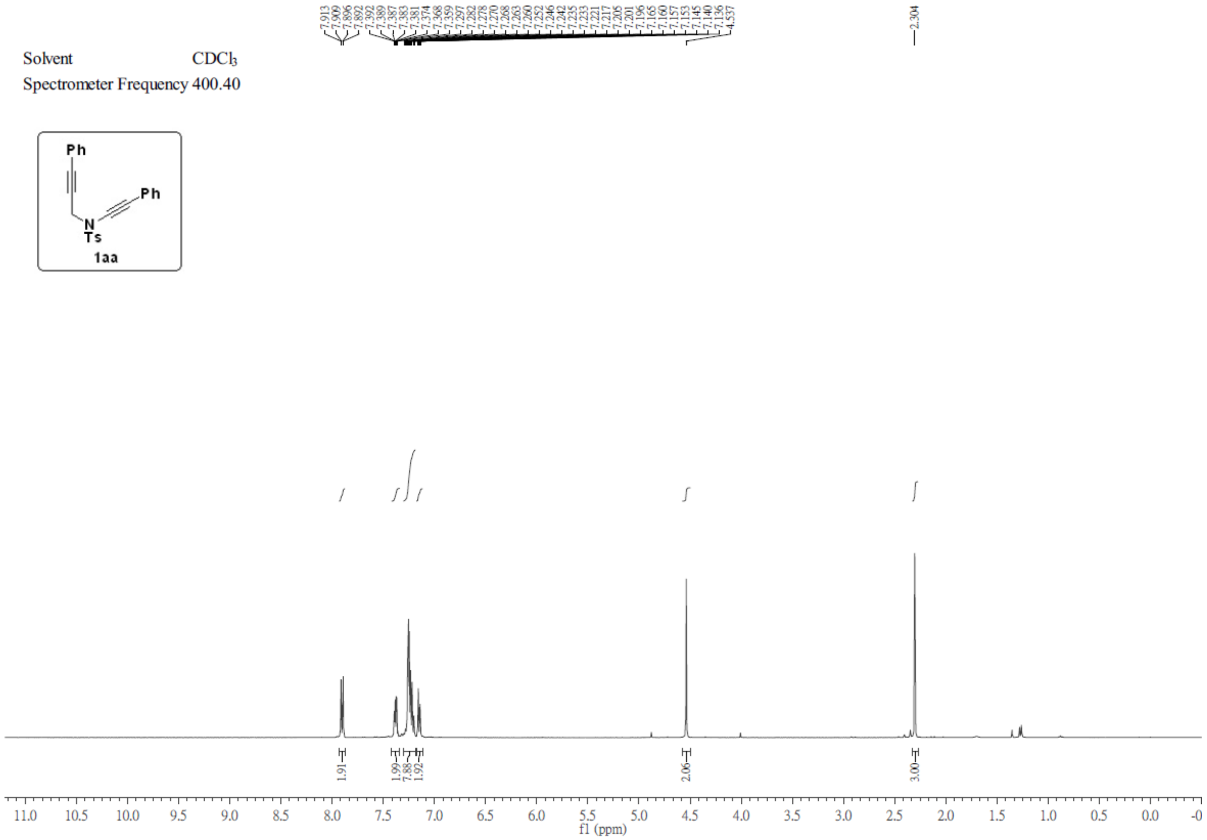

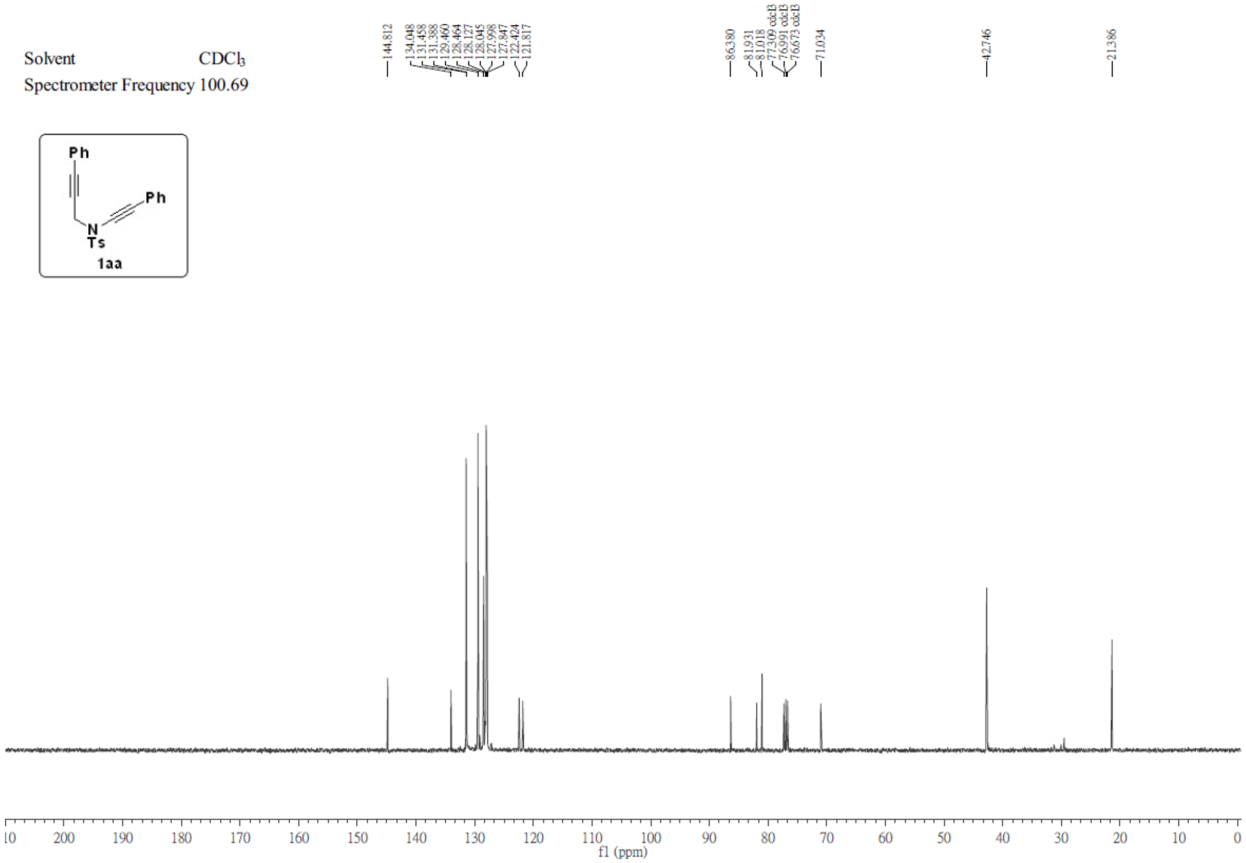

Supplementary Figure 83. ^{1}H (top) and ^{13}C (bottom) NMR spectra of compound **1aa**.

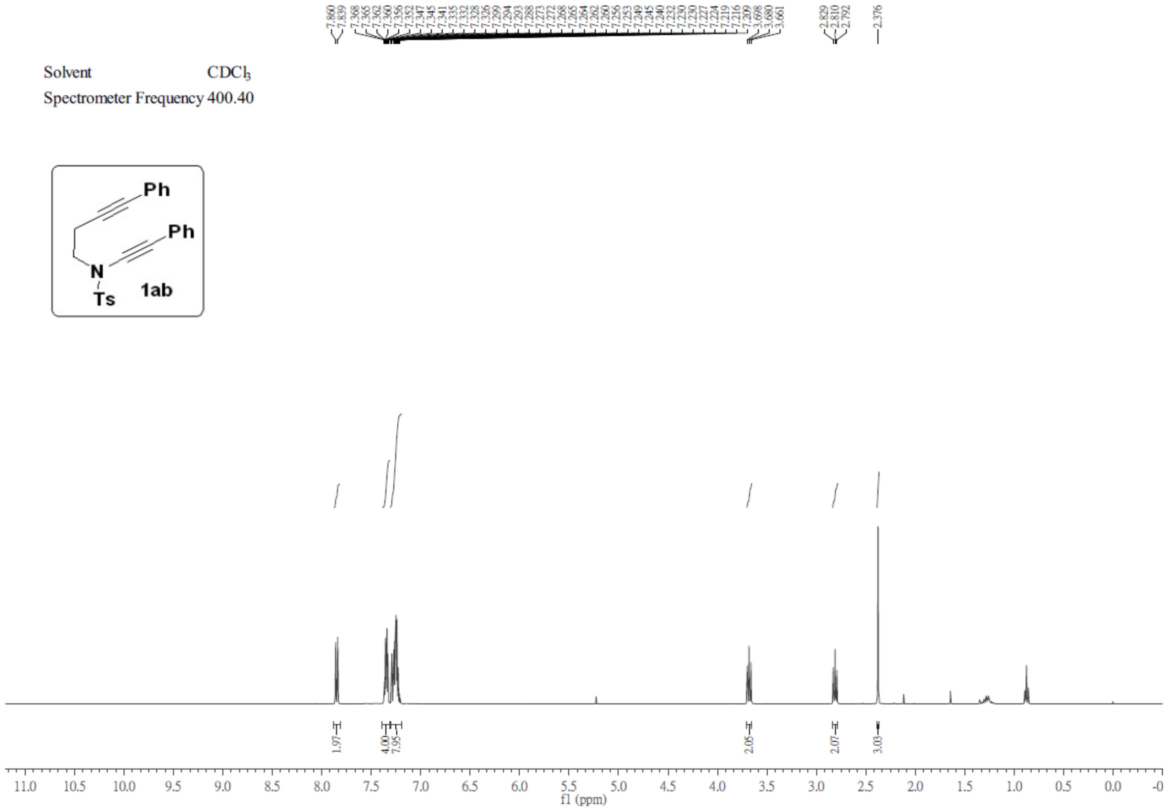

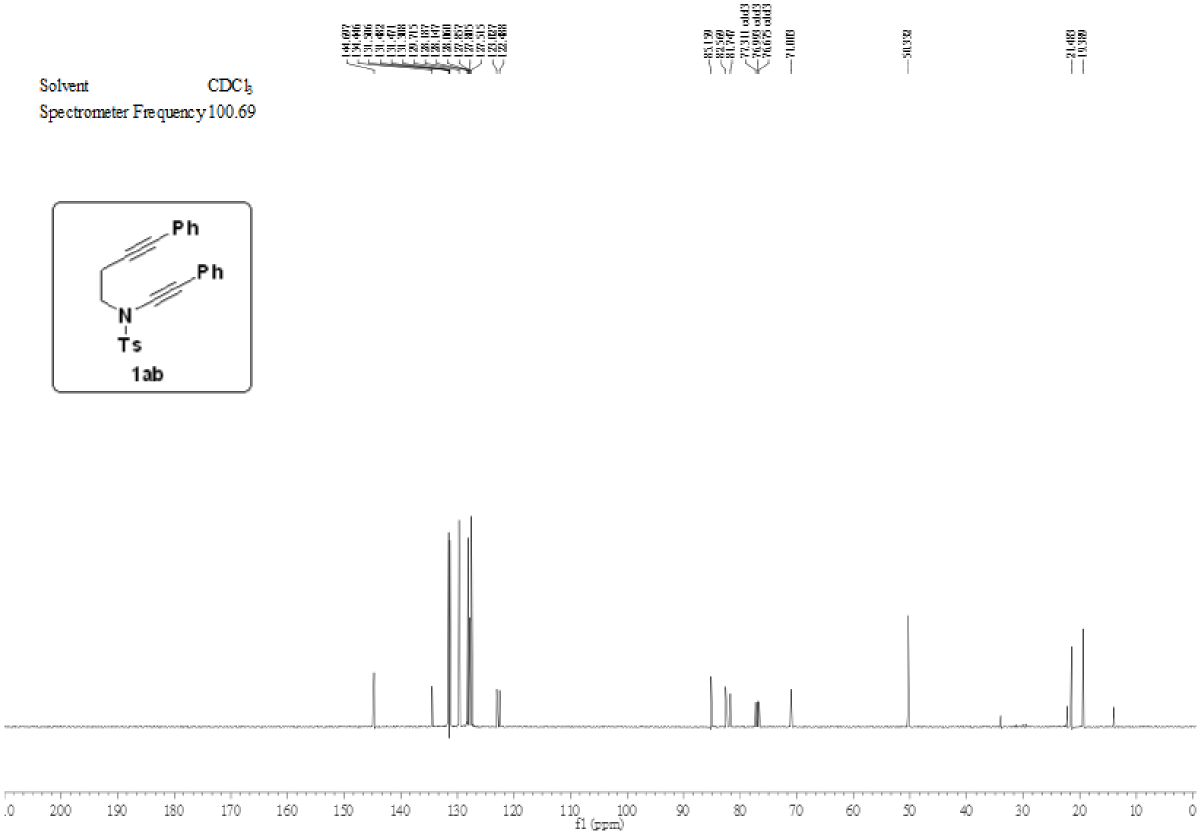

Supplementary Figure 84. ^{1}H (top) and ^{13}C (bottom) NMR spectra of compound **1ab**.

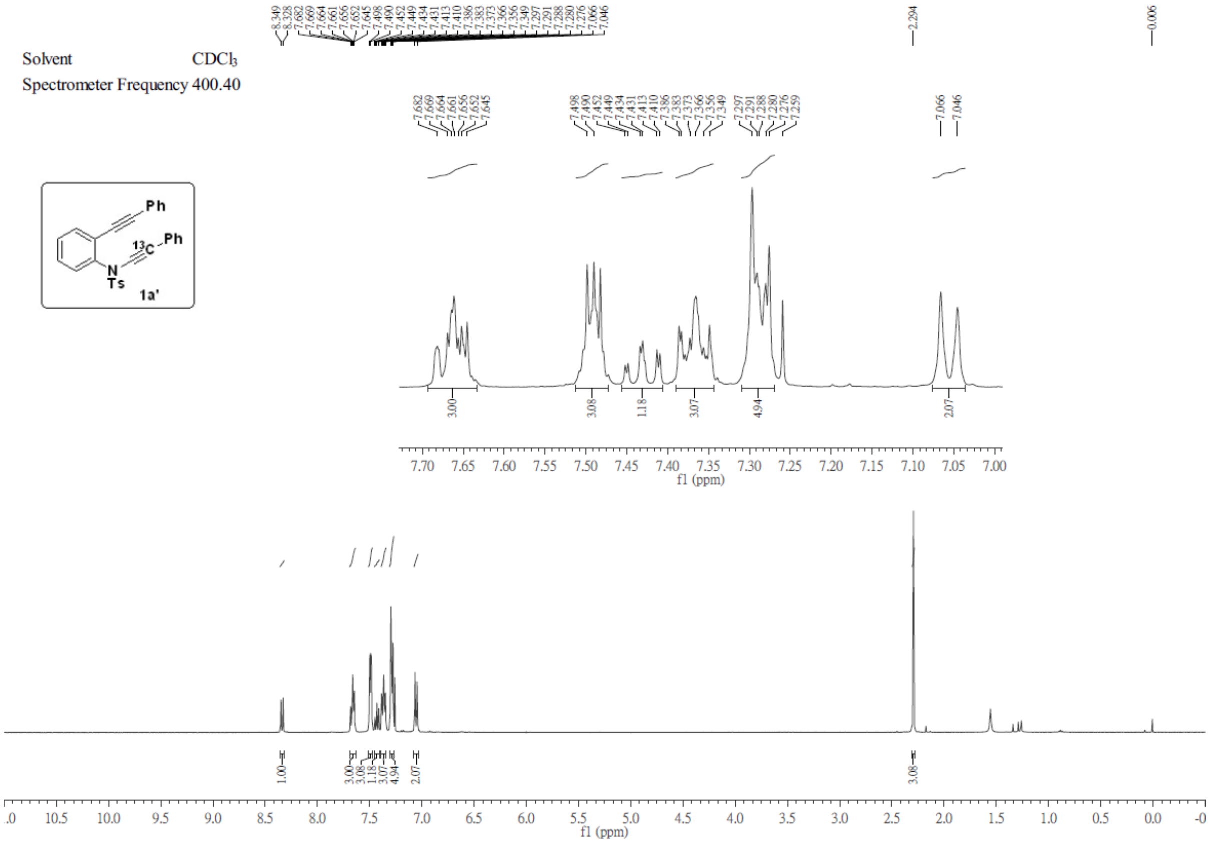

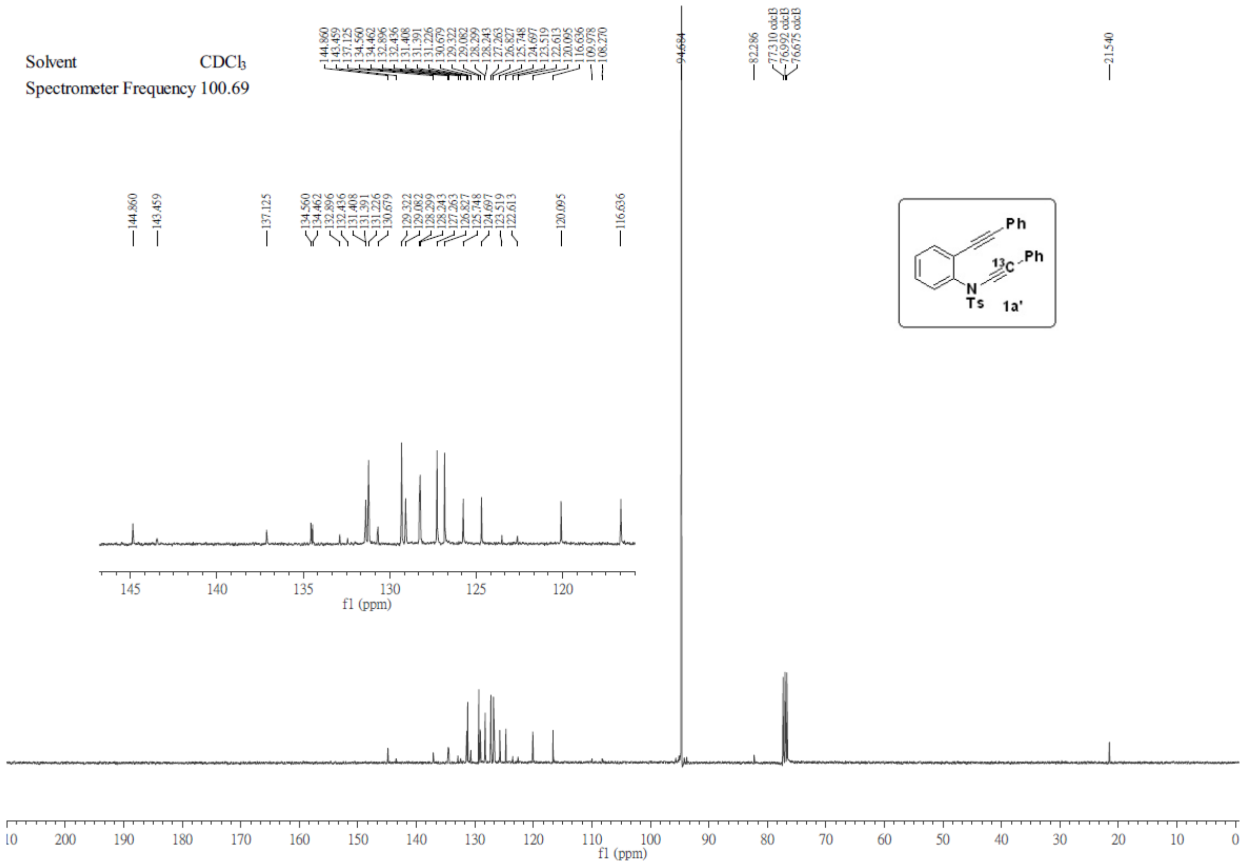

Supplementary Figure 85. ^{1}H (top) and ^{13}C (bottom) NMR spectra of compound **1a'** .

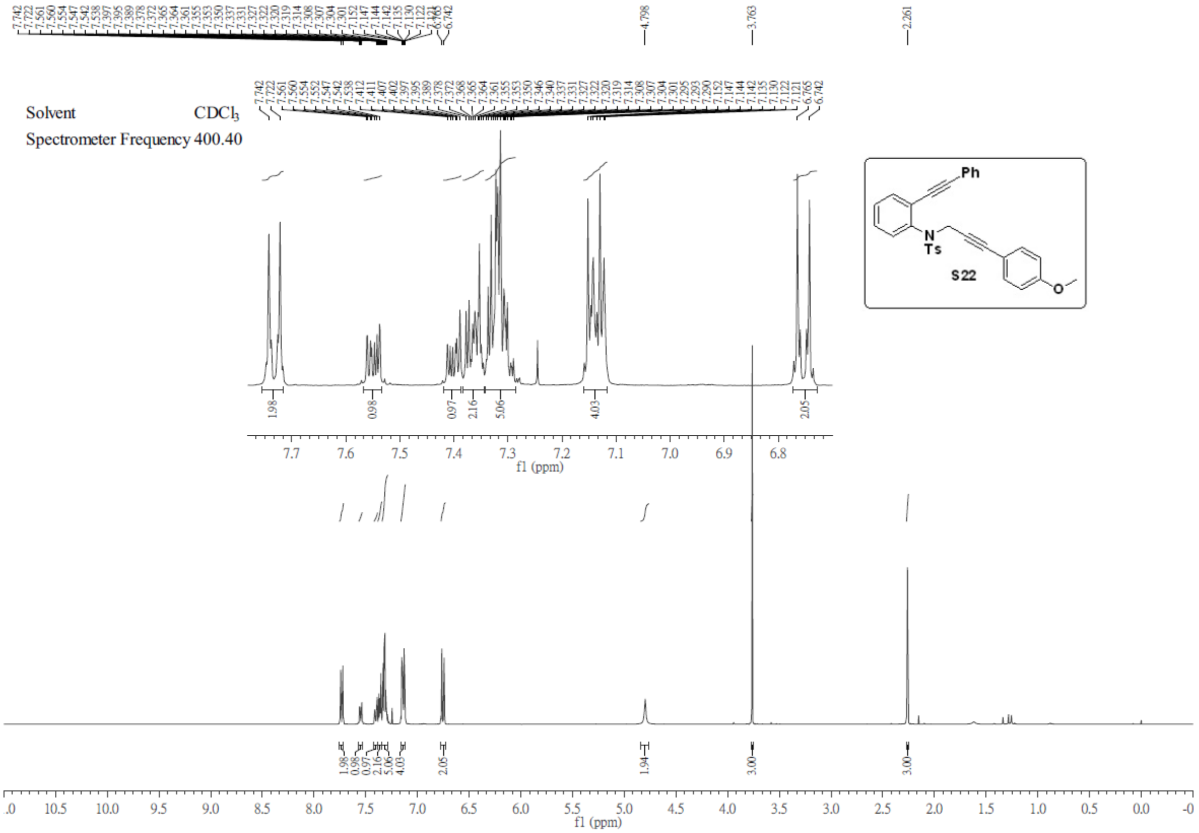

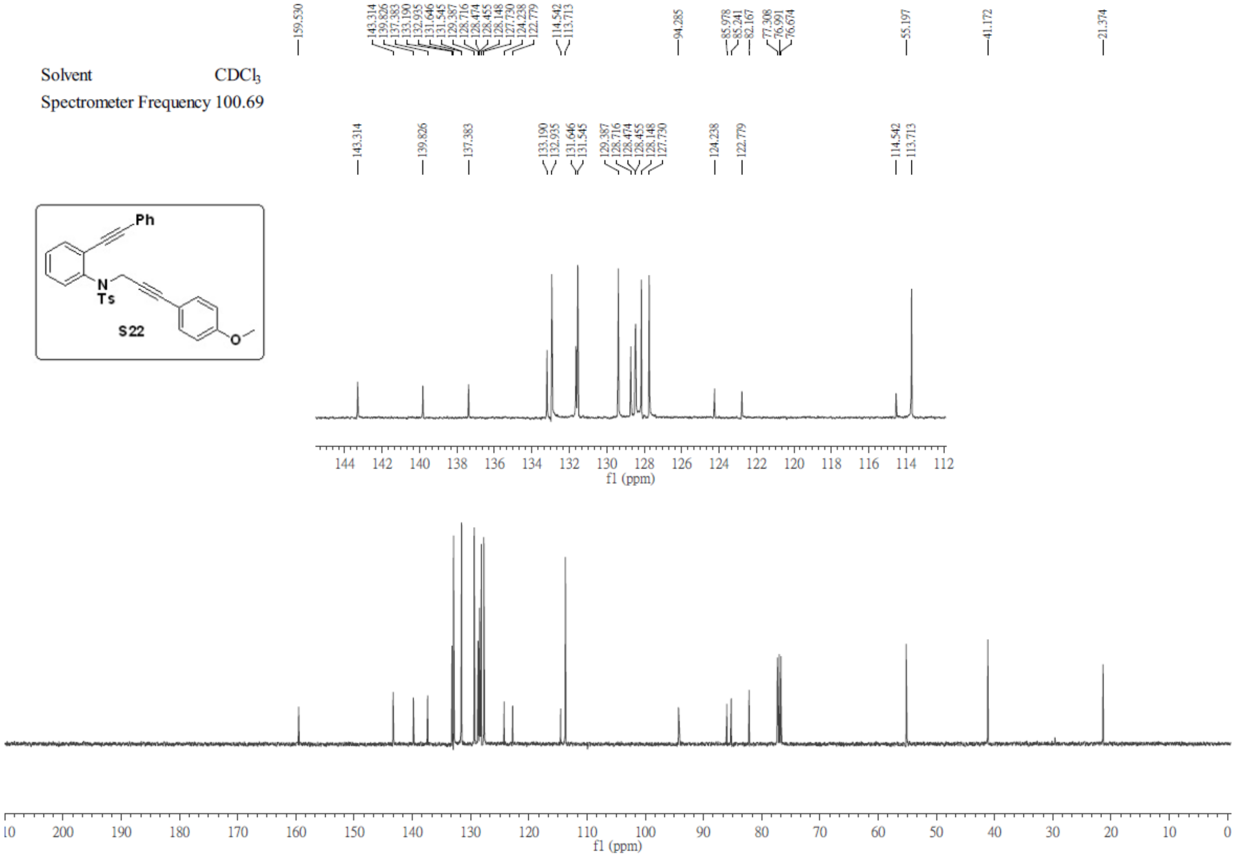

Supplementary Figure 86. ^{1}H (top) and ^{13}C (bottom) NMR spectra of compound **S22**.

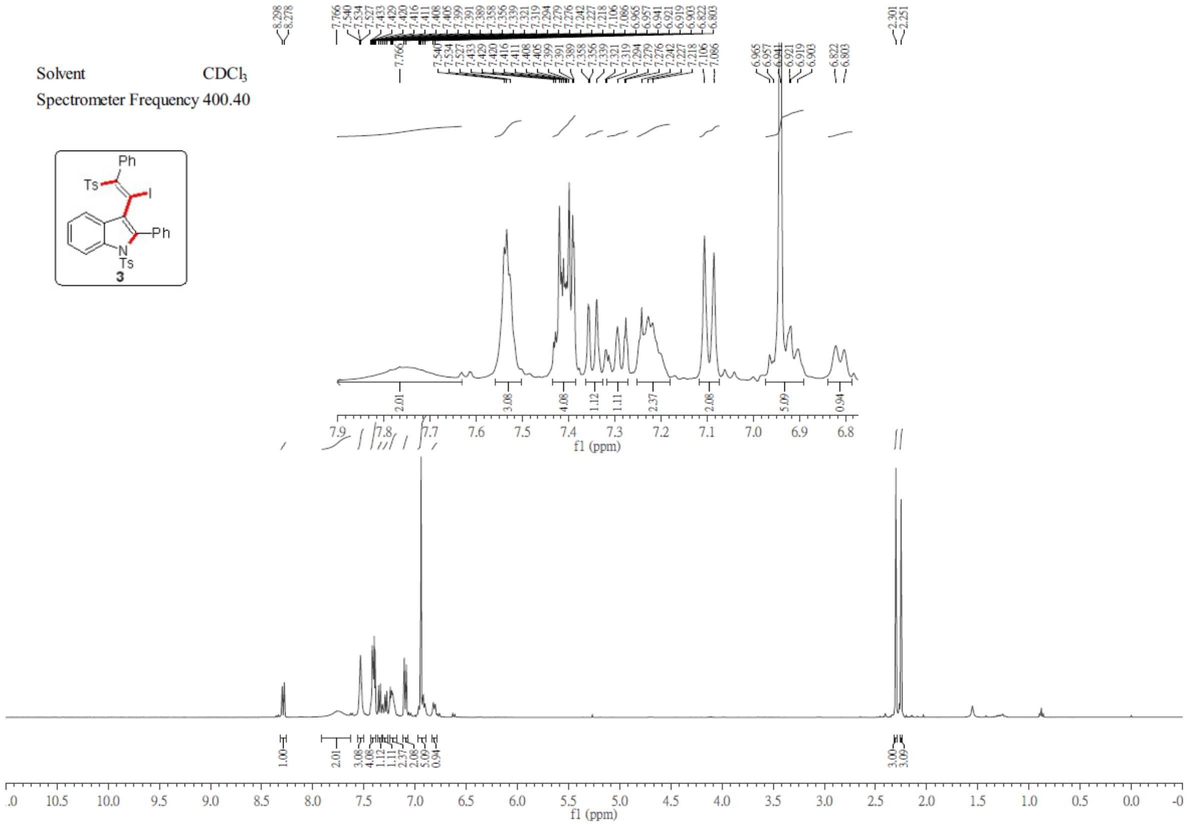

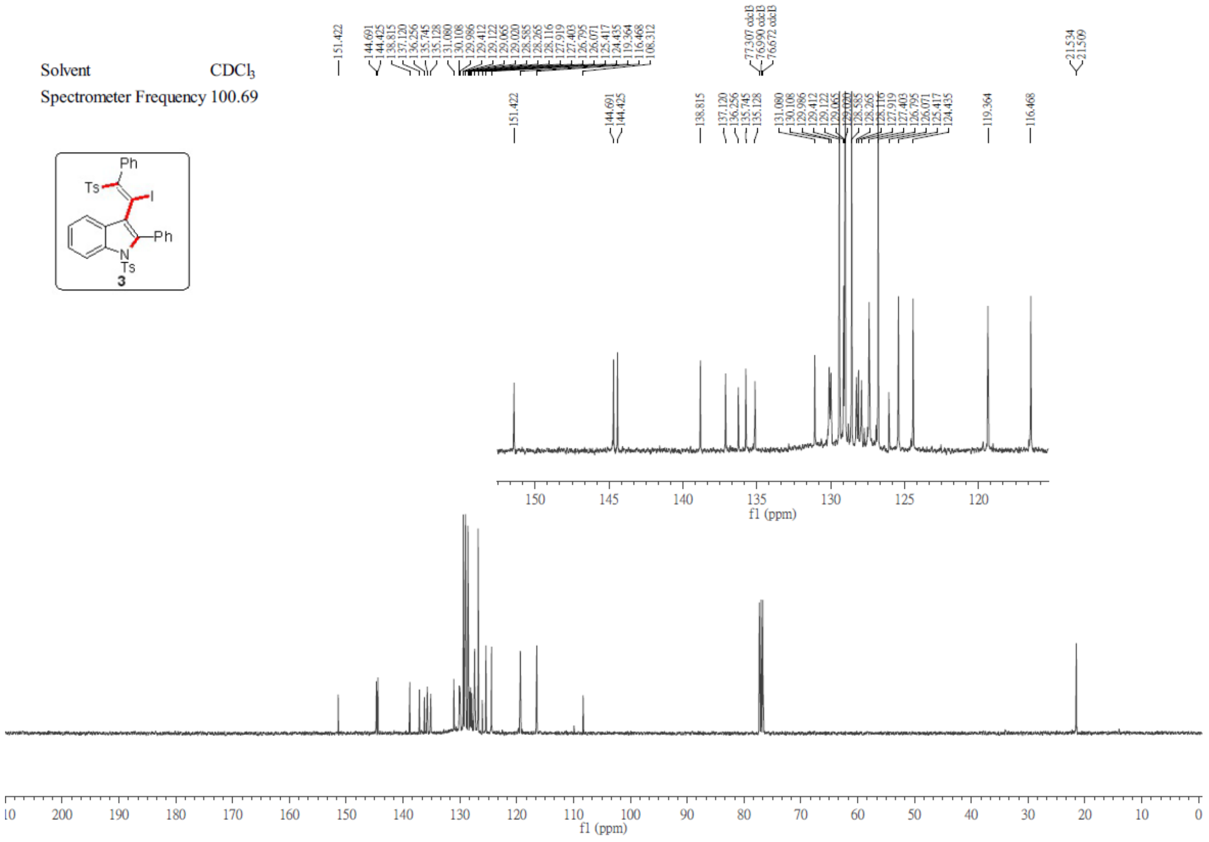

Supplementary Figure 87. ^{1}H (top) and ^{13}C (bottom) NMR spectra of compound **3**.

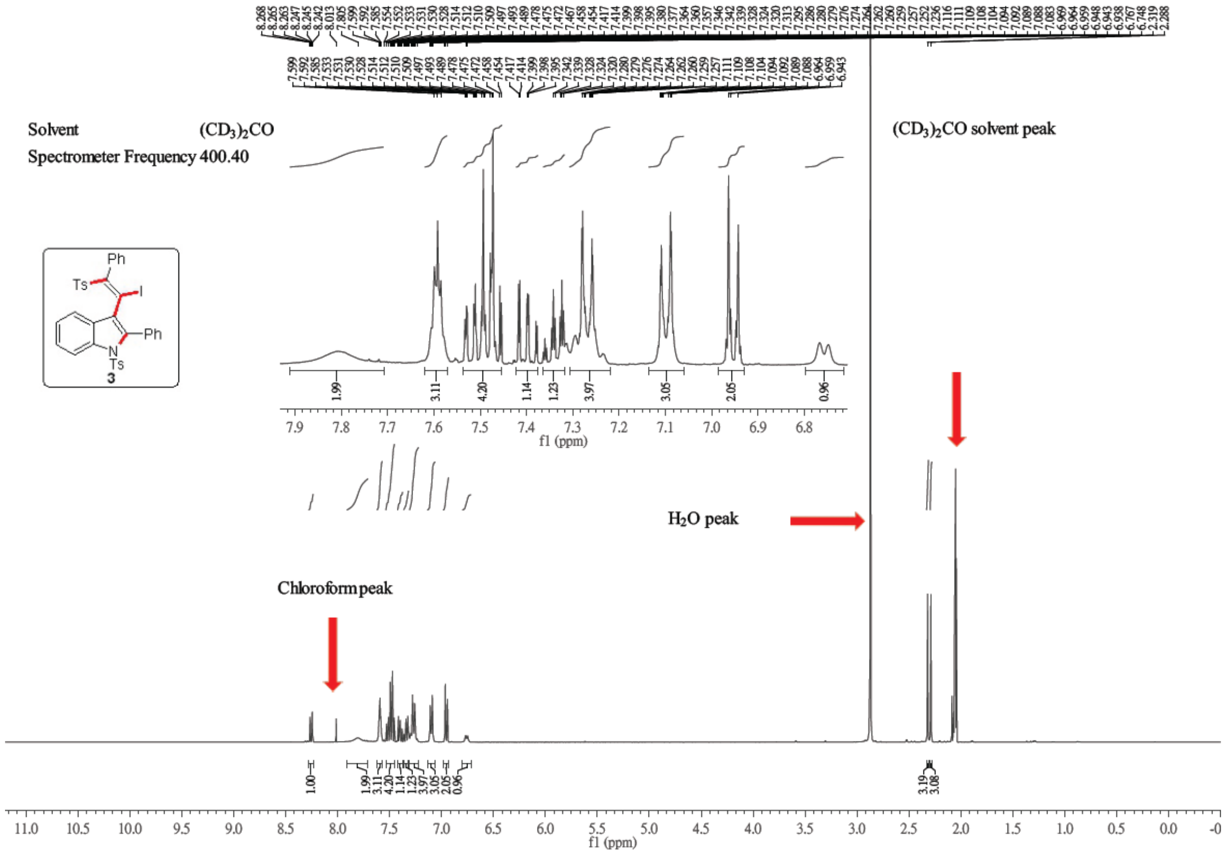

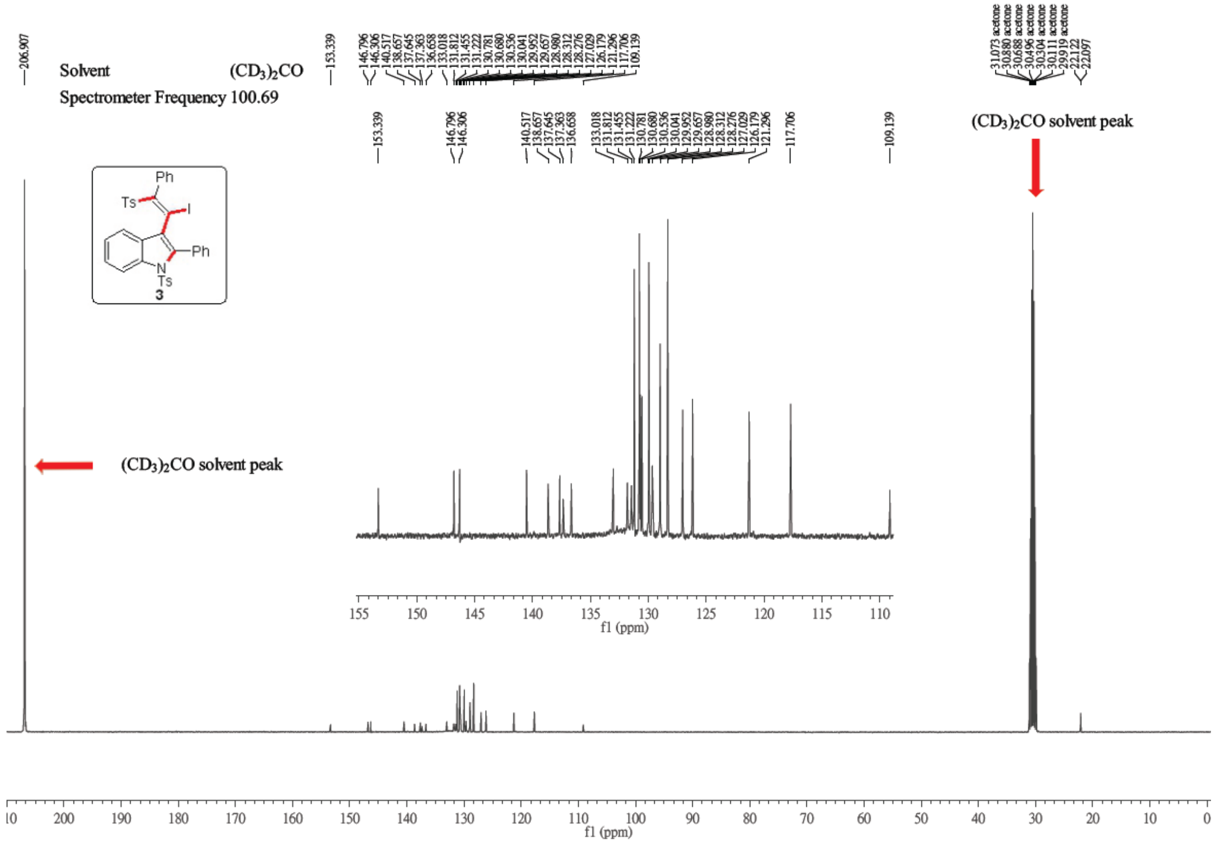

Supplementary Figure 88. ^{1}H (top) and ^{13}C (bottom) NMR spectra of compound **3** in $(CD_3)_2CO$.

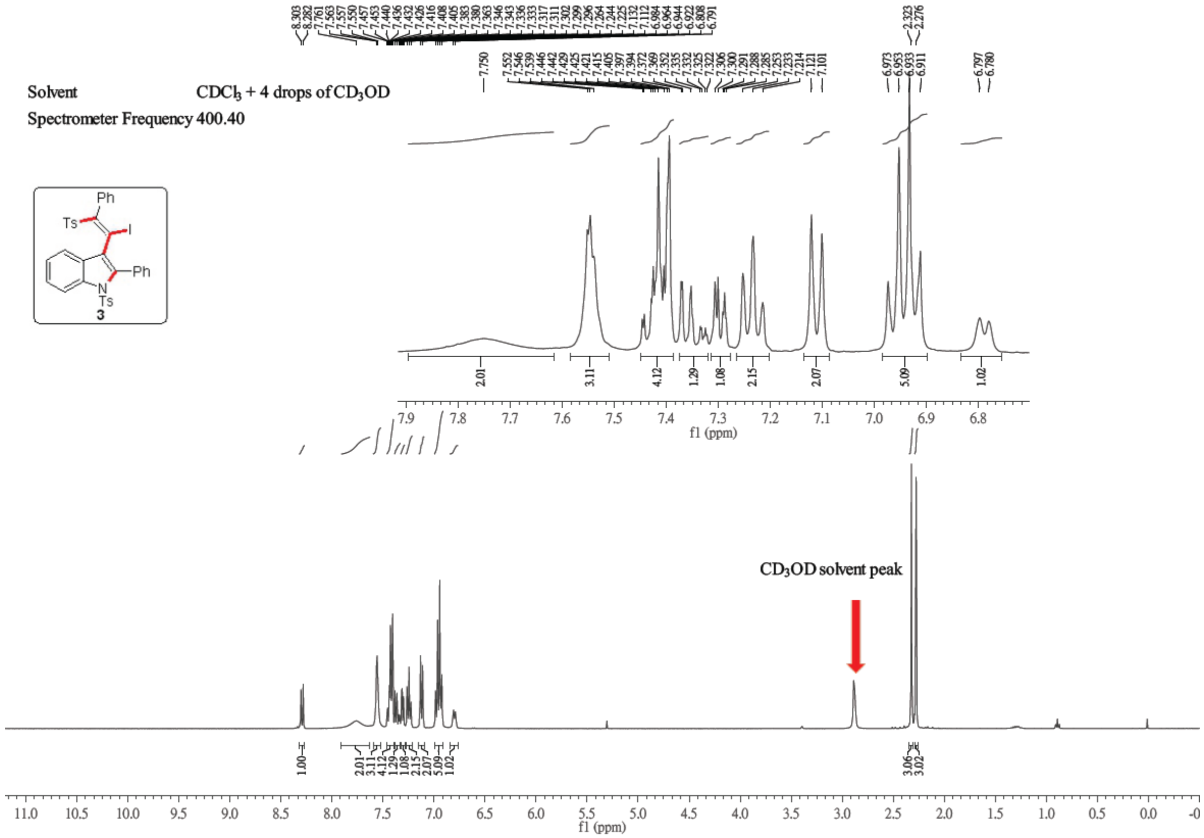

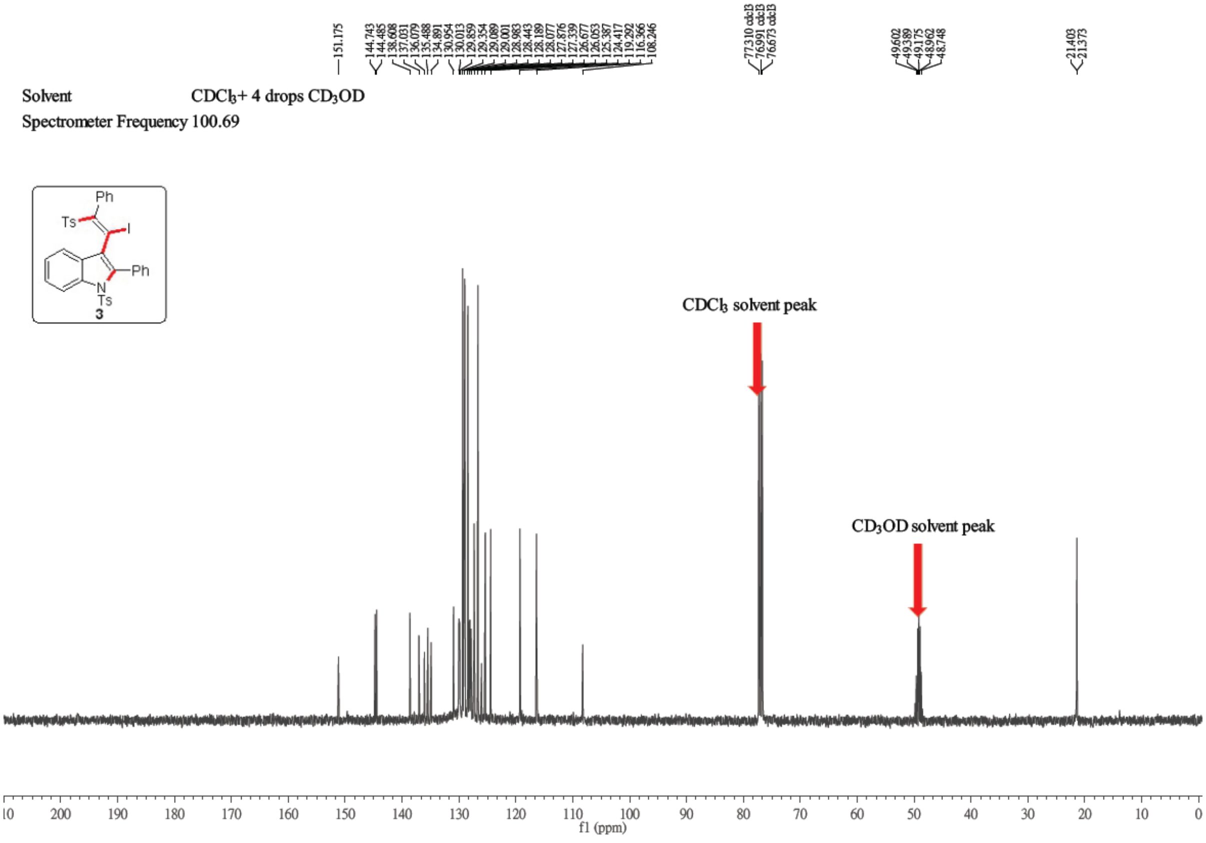

Supplementary Figure 89. ^{1}H (top) and ^{13}C (bottom) NMR spectra of compound **3** in $CDCl_3$+CD_3OD.

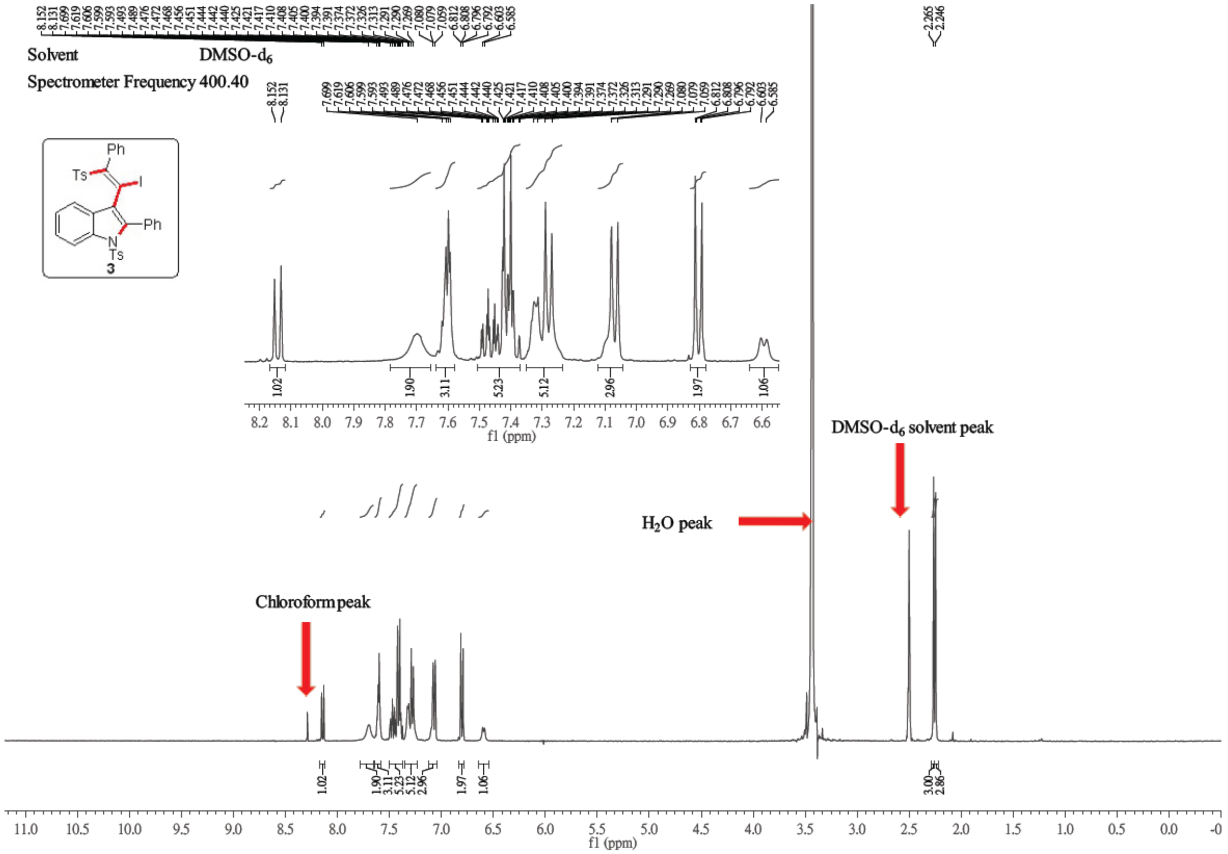

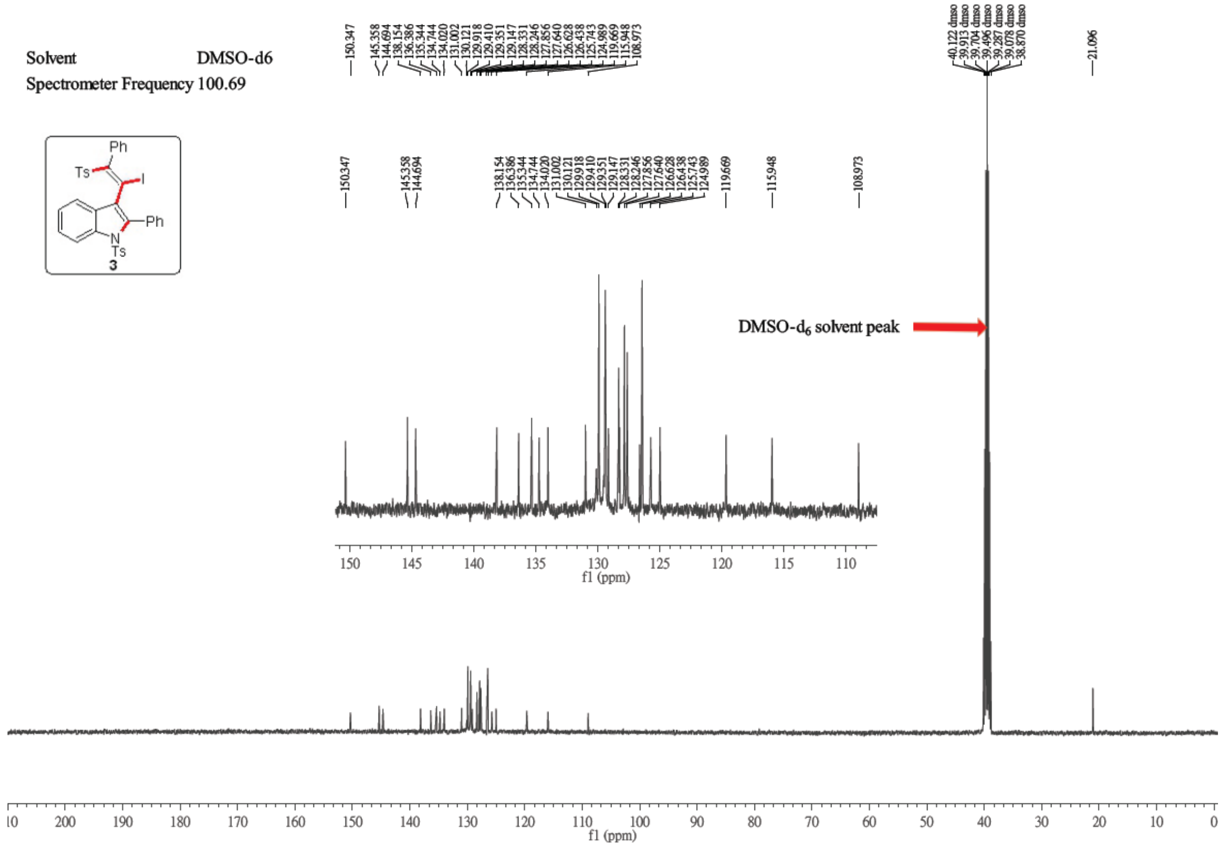

Supplementary Figure 90. ^{1}H (top) and ^{13}C (bottom) NMR spectra of compound **3** in DMSO-d$_6$.

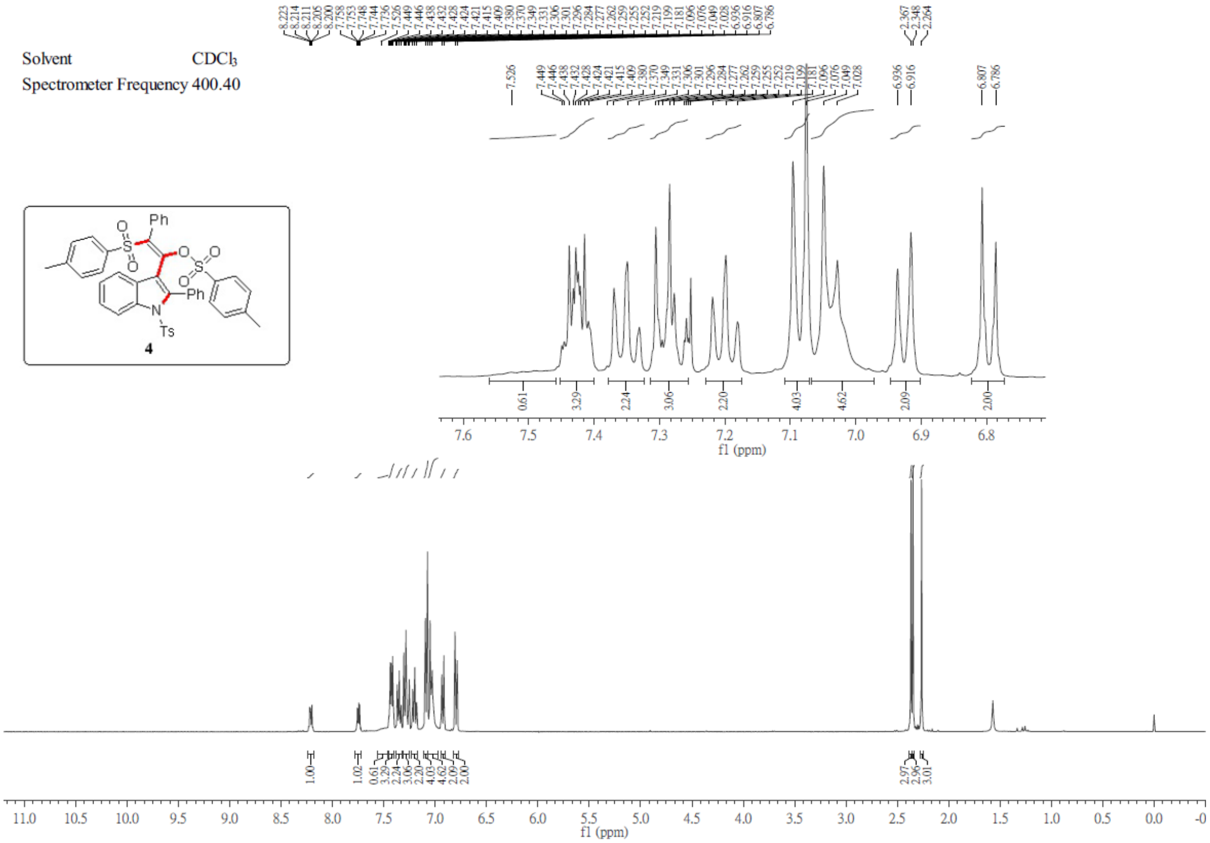

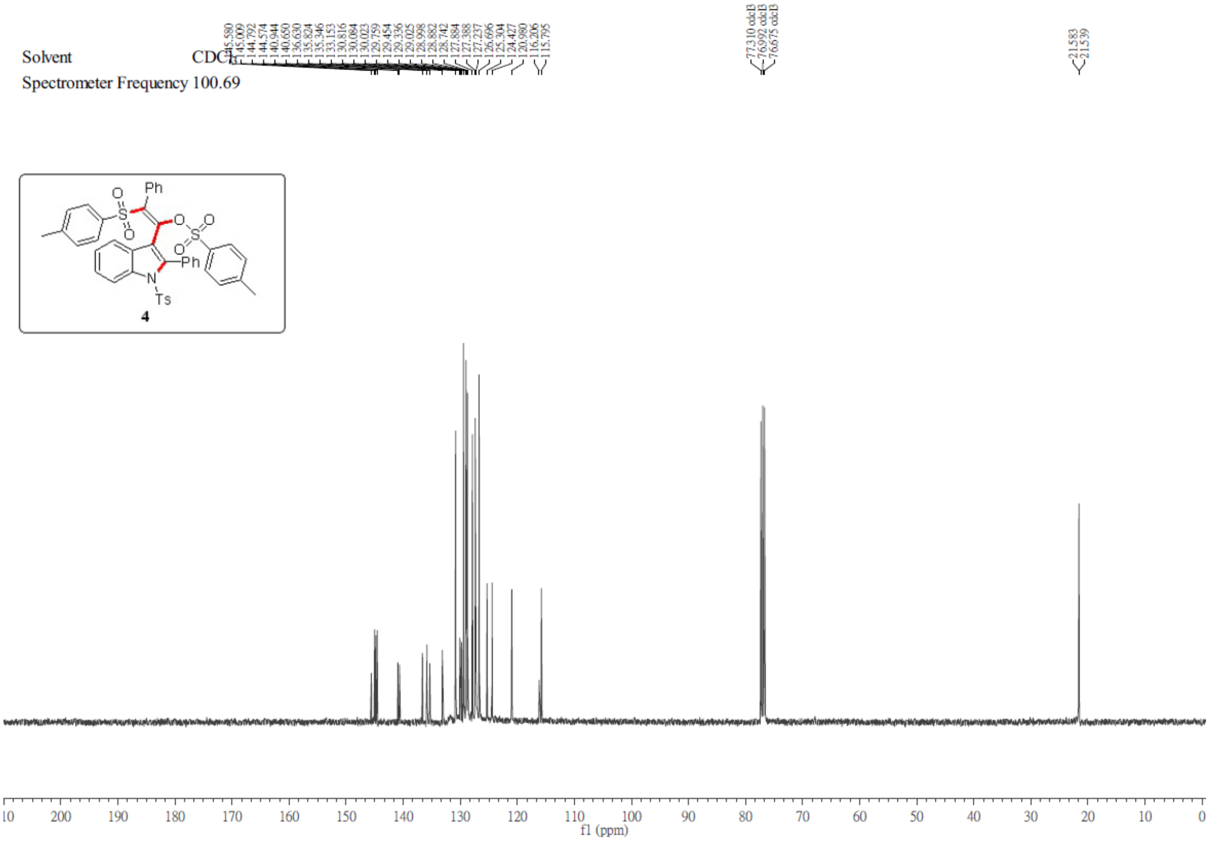

Supplementary Figure 91. ^{1}H (top) and ^{13}C (bottom) NMR spectra of compound **4**.

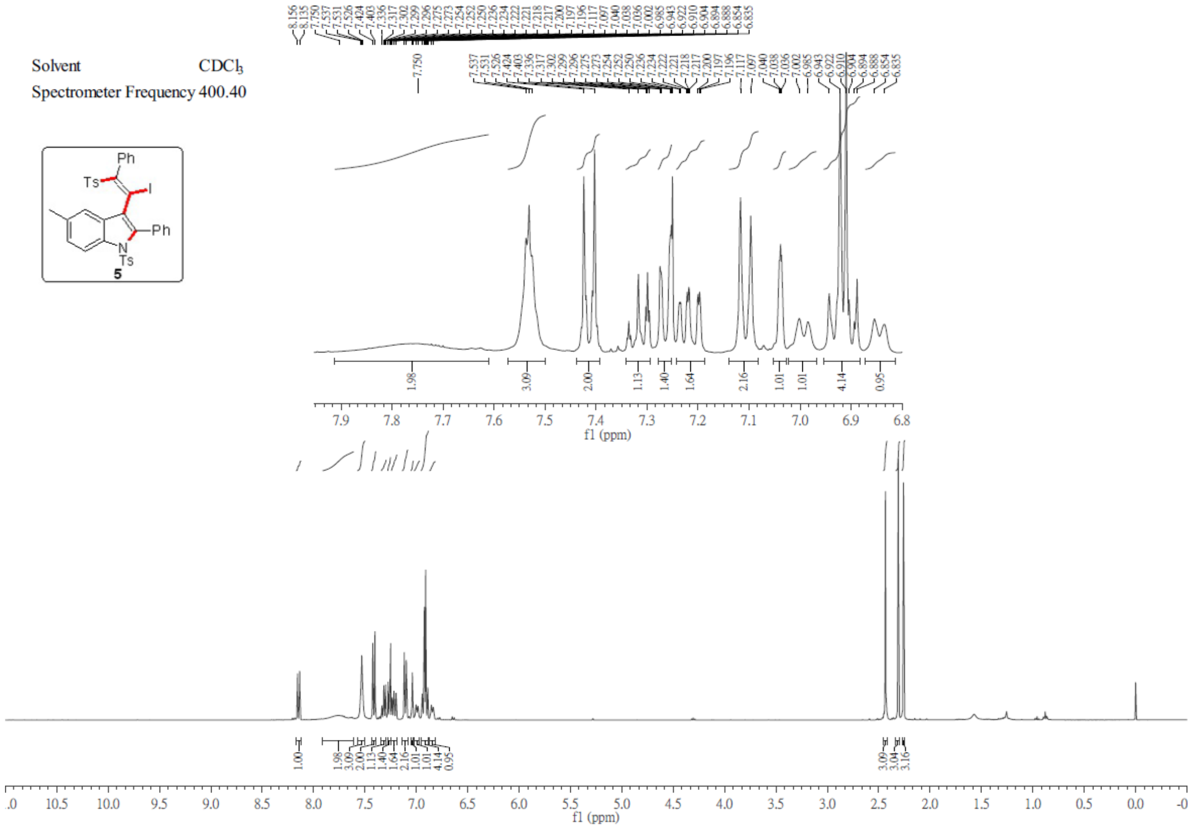

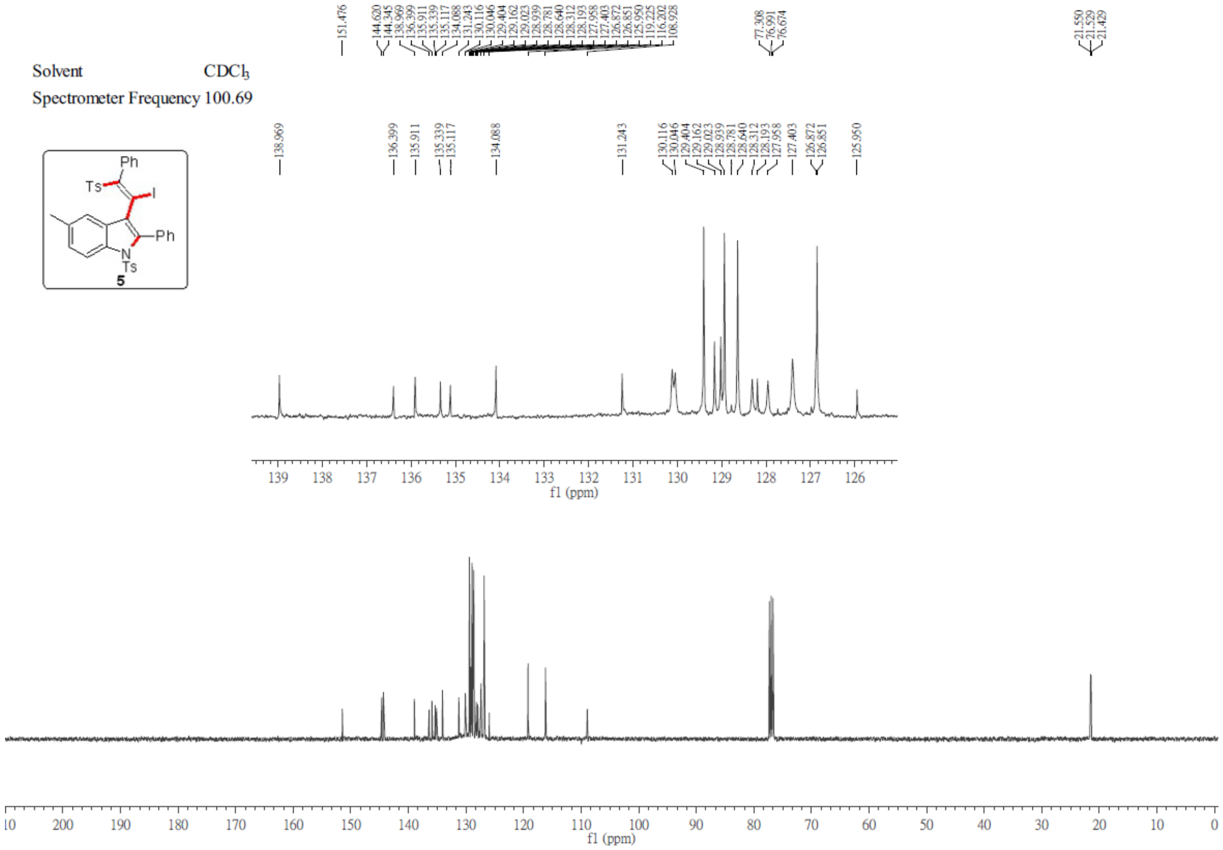

Supplementary Figure 92. ^{1}H (top) and ^{13}C (bottom) NMR spectra of compound **5**.

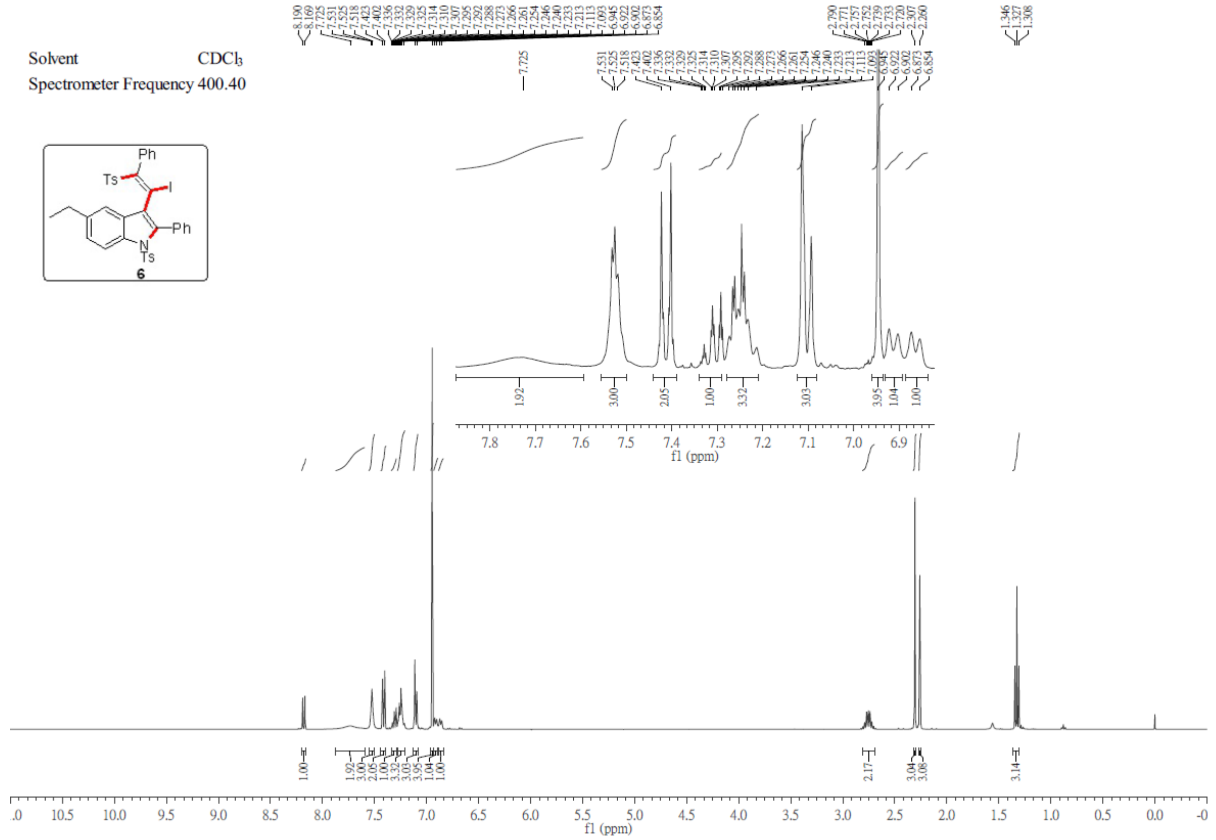

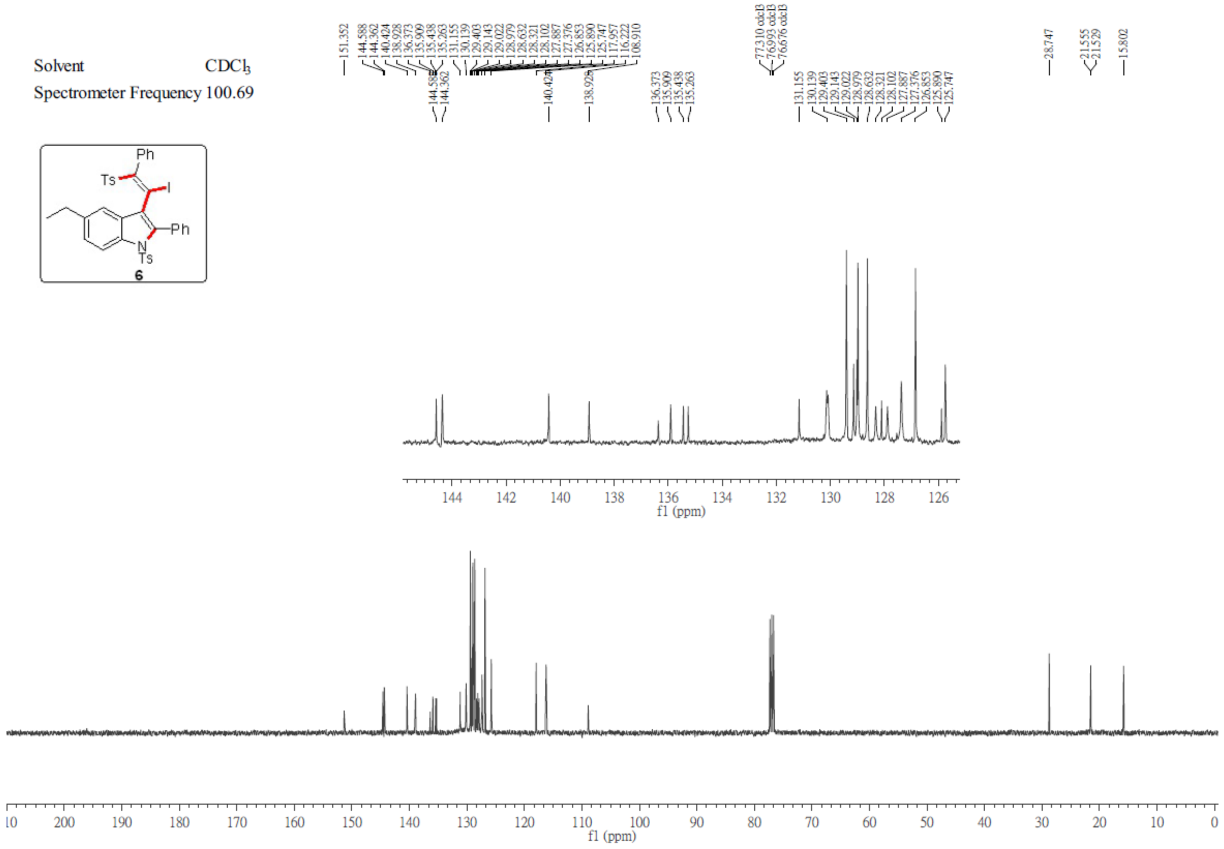

Supplementary Figure 93. ^{1}H (top) and ^{13}C (bottom) NMR spectra of compound **6**.

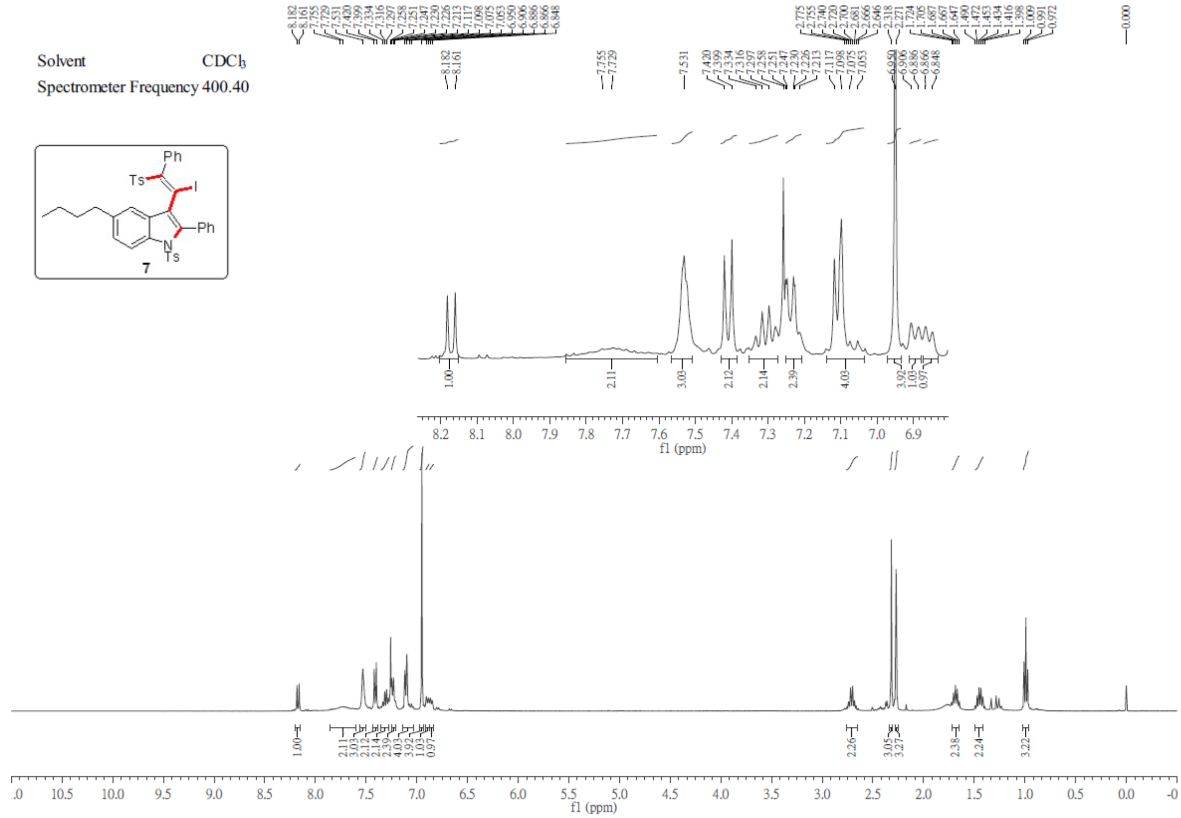

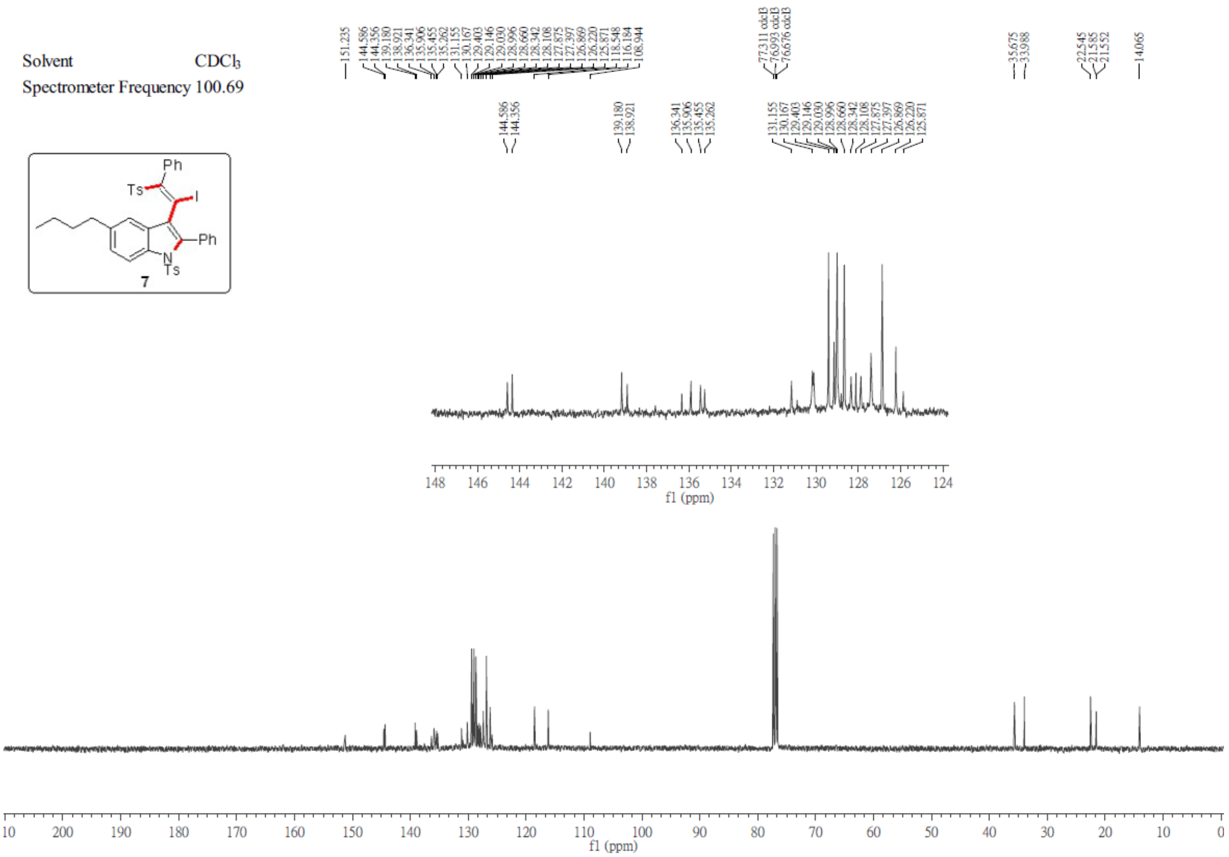

Supplementary Figure 94. ^{1}H (top) and ^{13}C (bottom) NMR spectra of compound **7**.

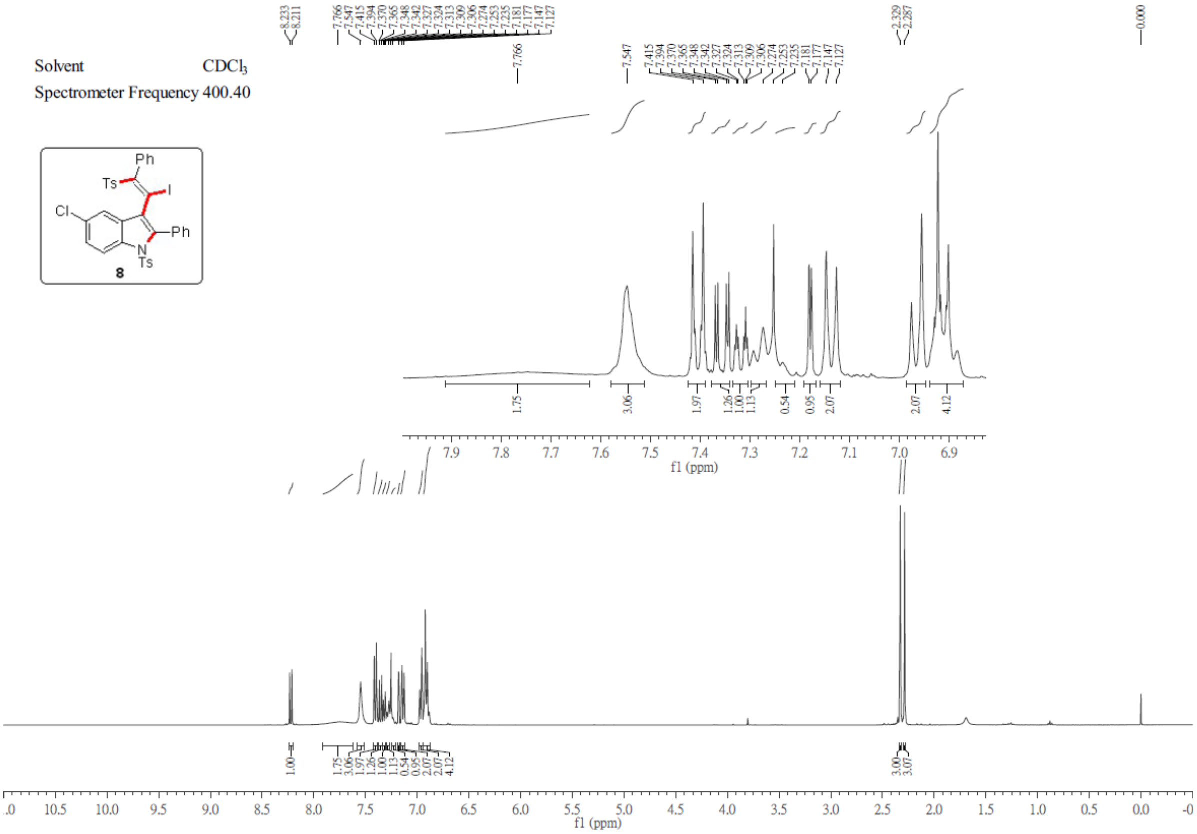

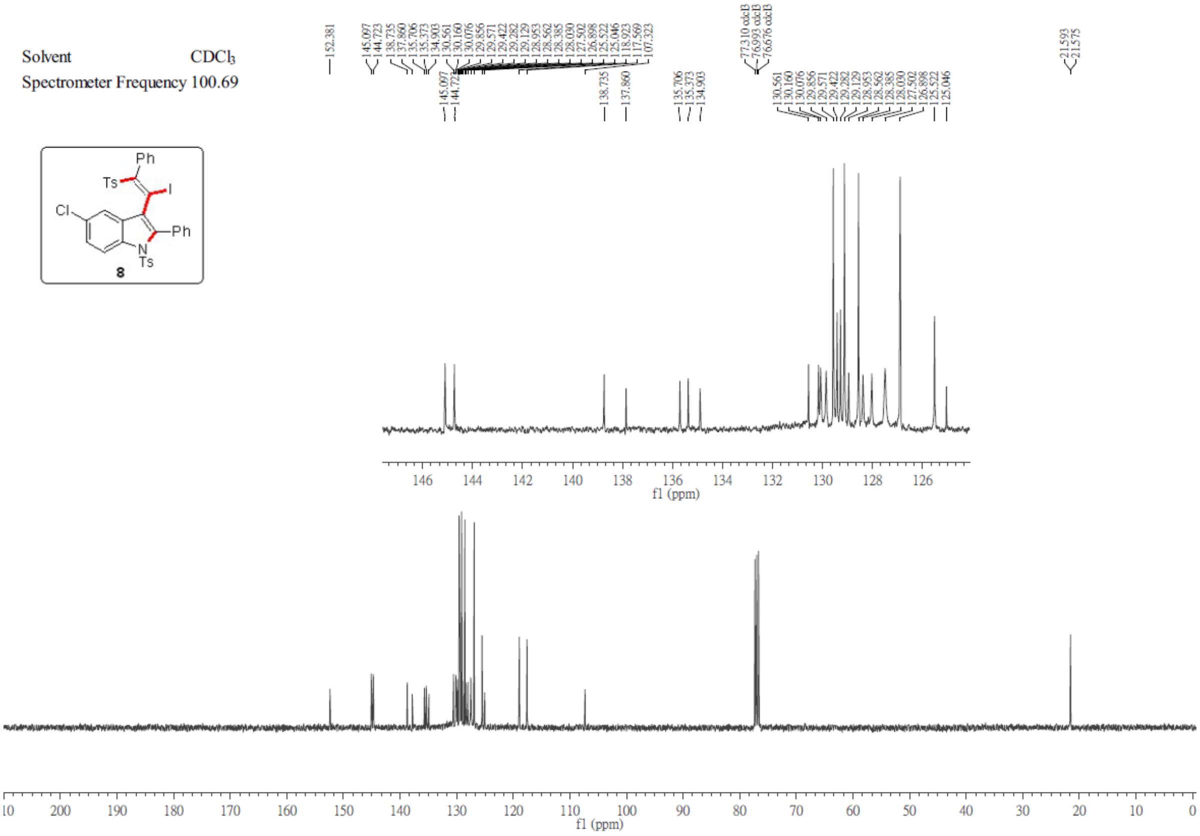

Supplementary Figure 95. ^{1}H (top) and ^{13}C (bottom) NMR spectra of compound **8**.

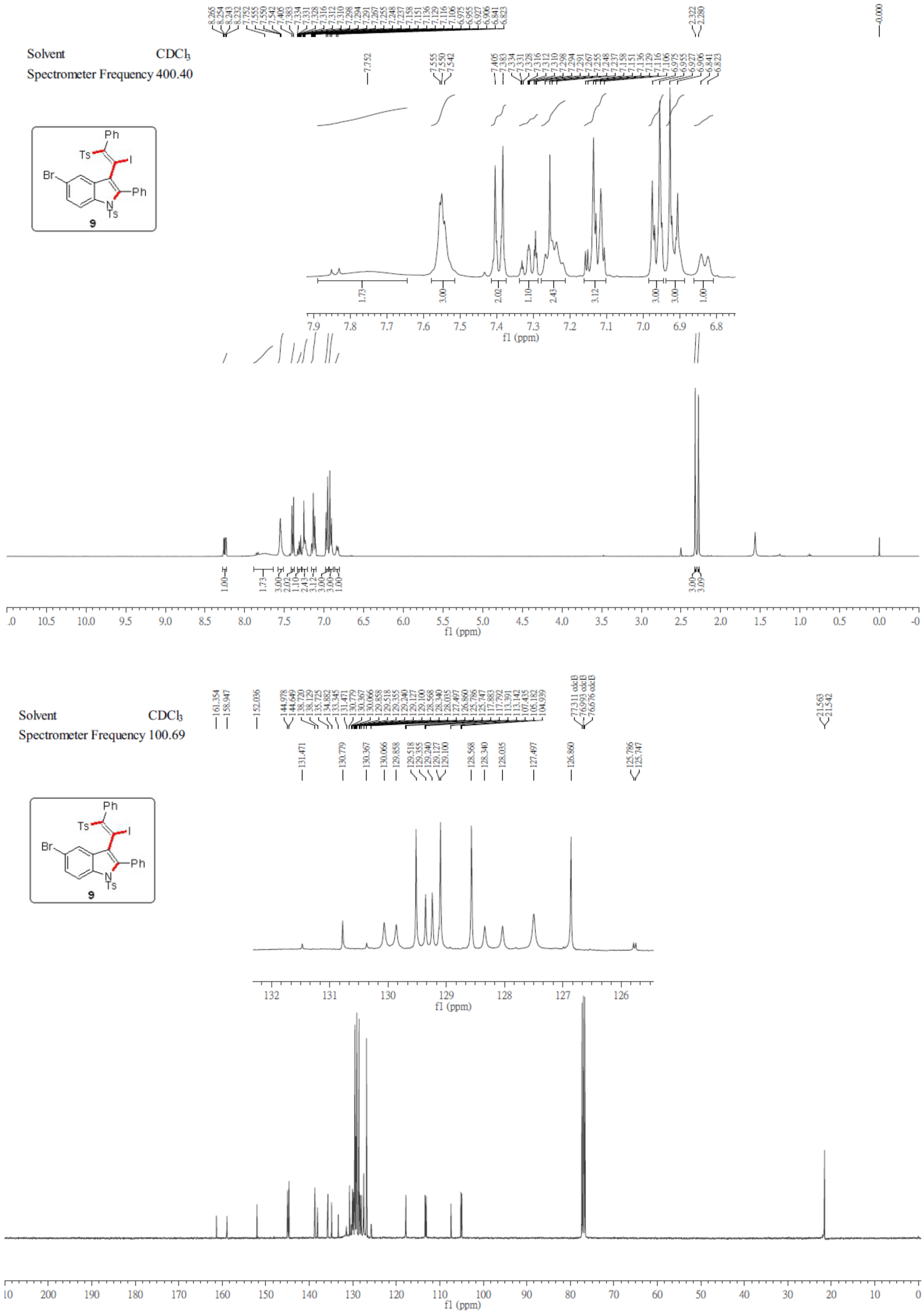

Supplementary Figure 96. ^{1}H (top) and ^{13}C (bottom) NMR spectra of compound **9**.

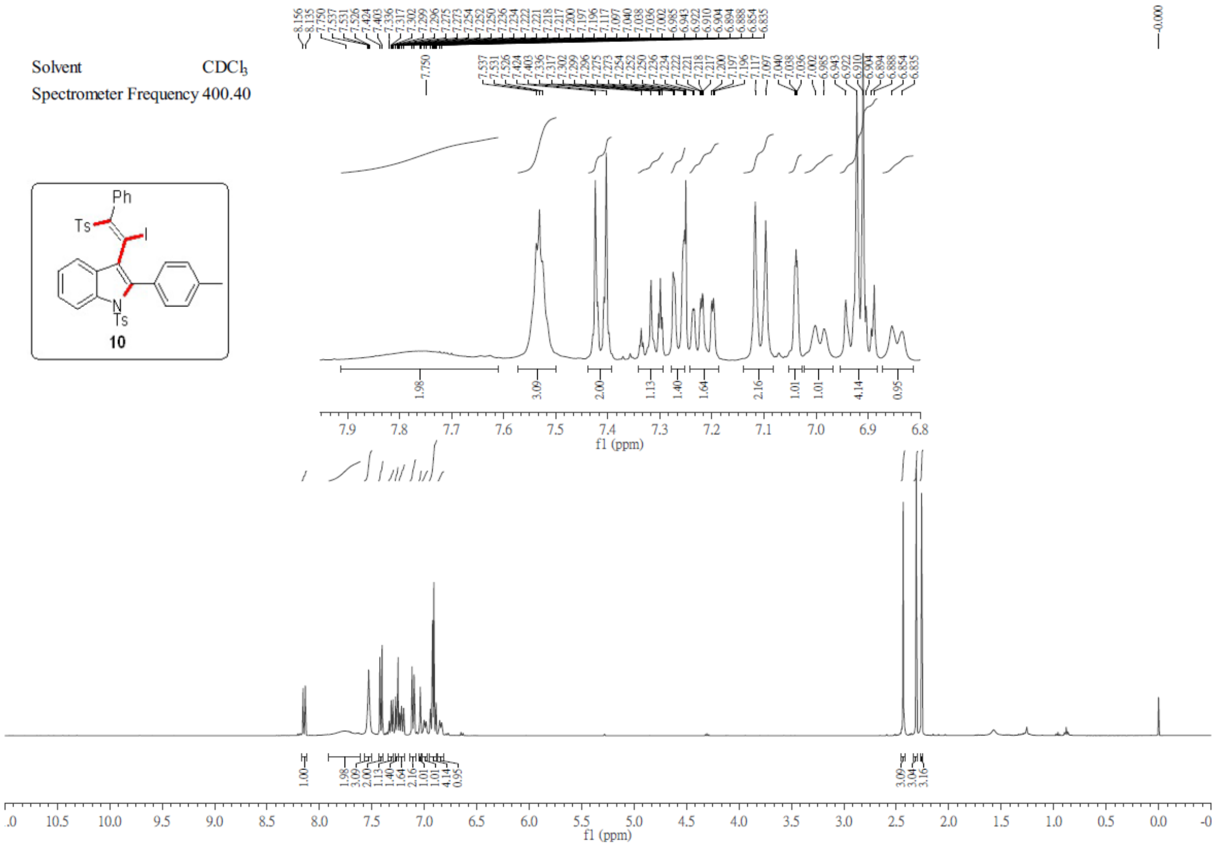

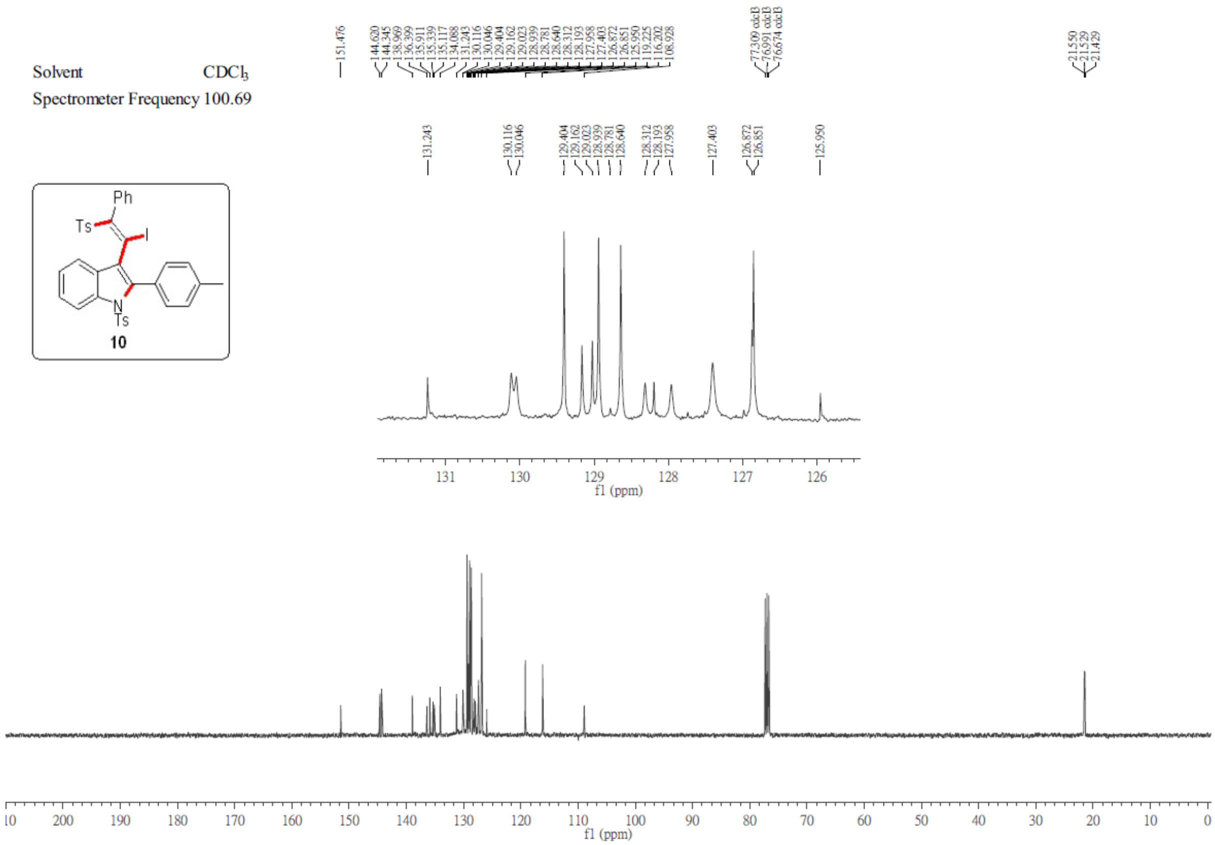

Supplementary Figure 97. ^{1}H (top) and ^{13}C (bottom) NMR spectra of compound **10**.

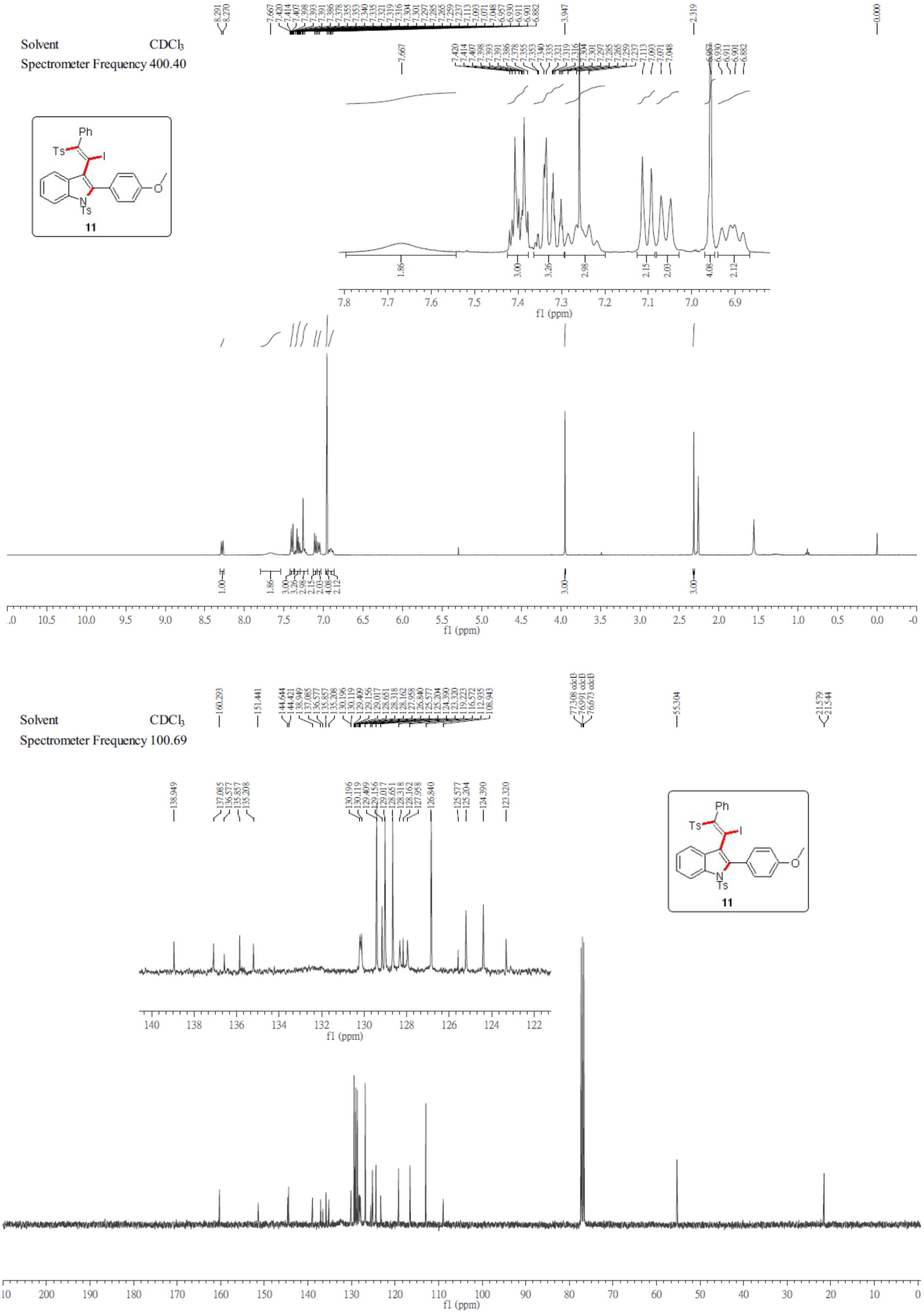

Supplementary Figure 98. ^{1}H (top) and ^{13}C (bottom) NMR spectra of compound **11**.

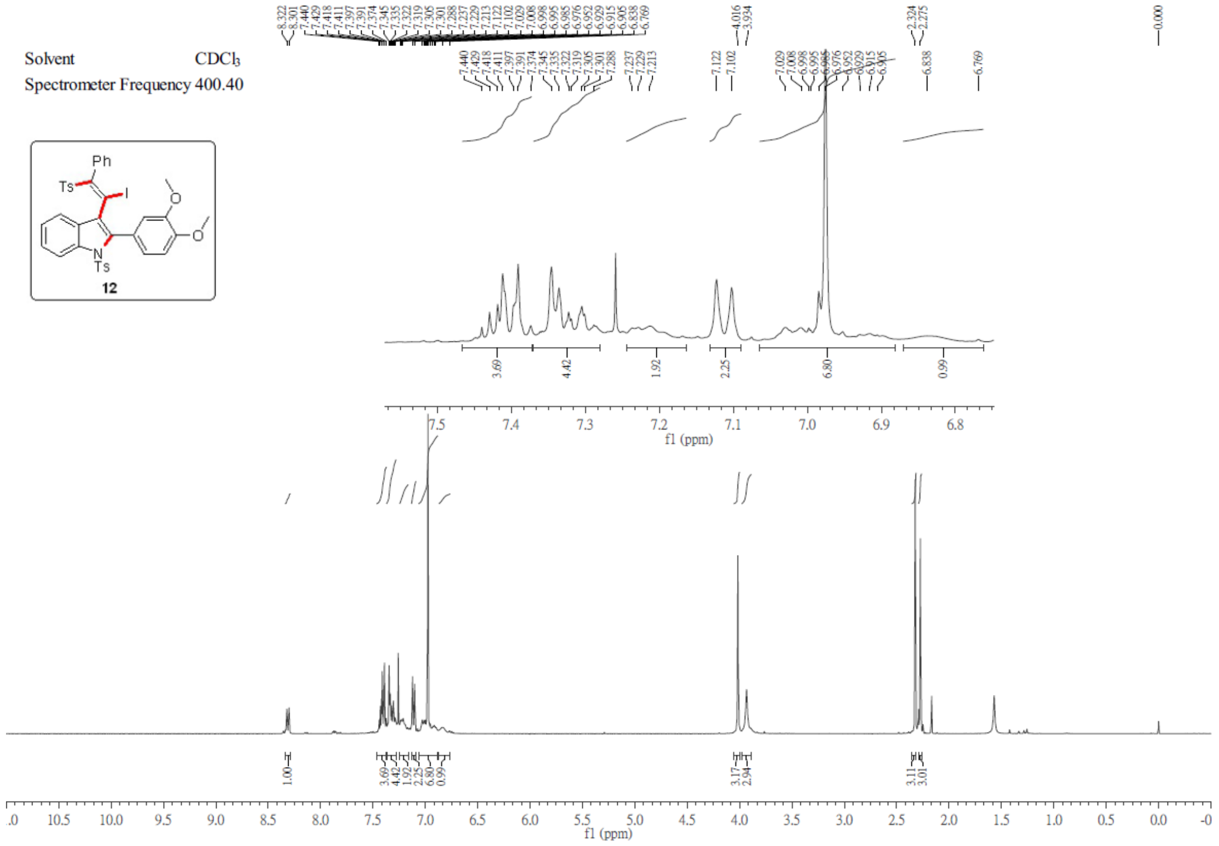

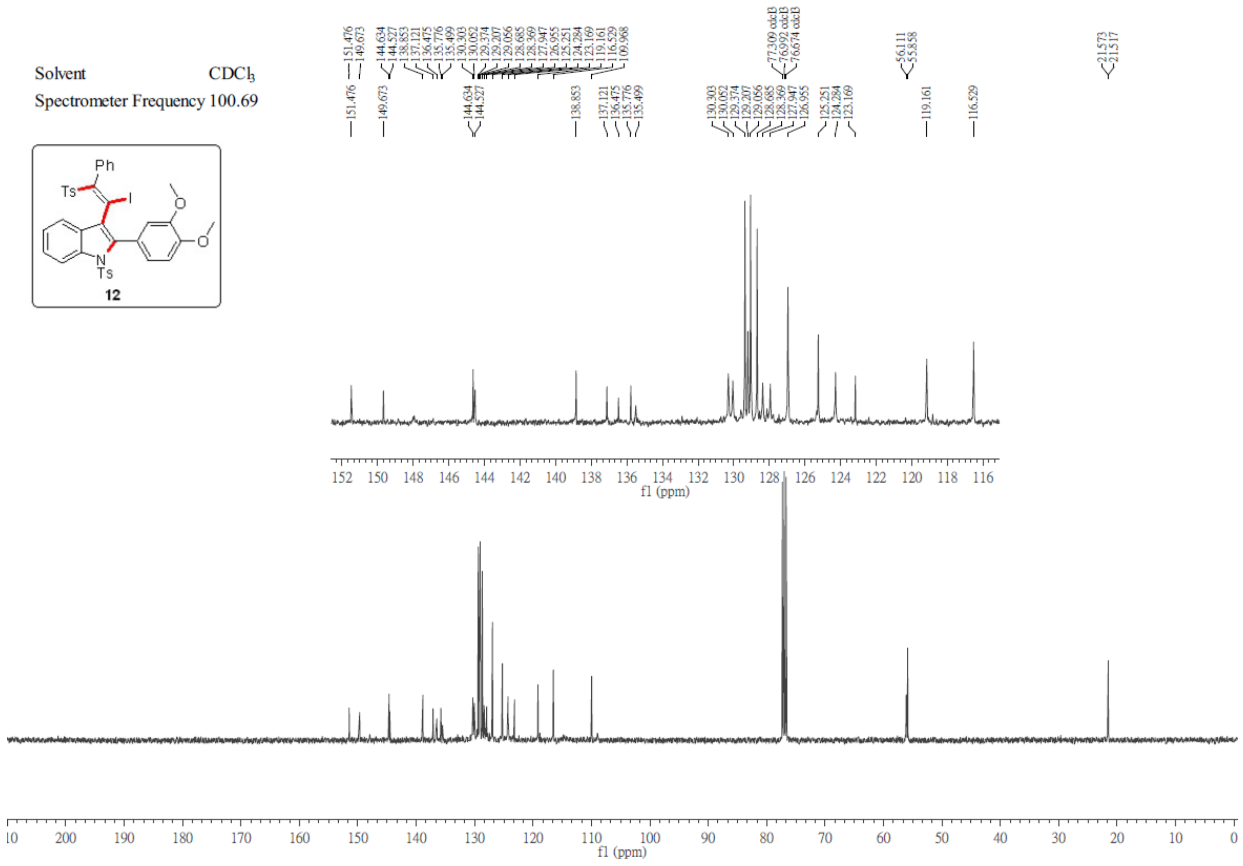

Supplementary Figure 99. ^{1}H (top) and ^{13}C (bottom) NMR spectra of compound **12**.

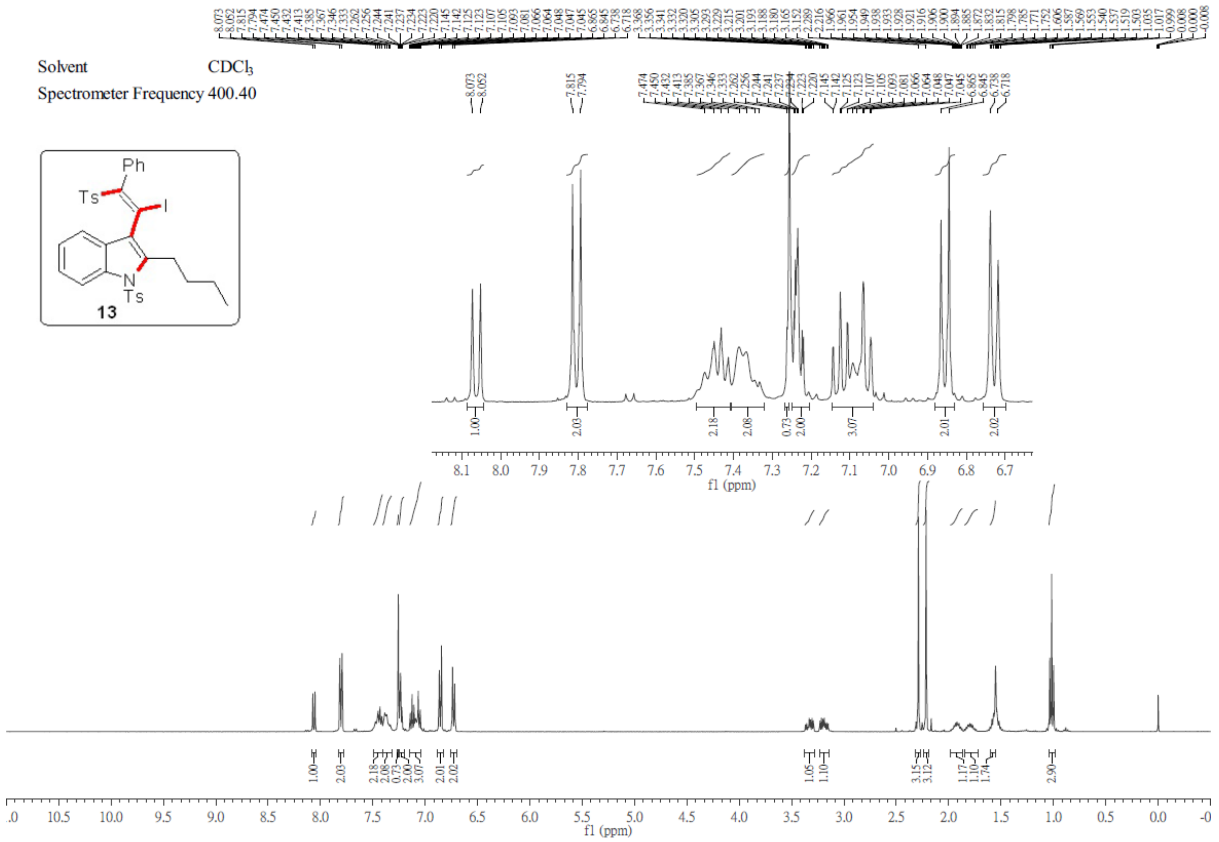

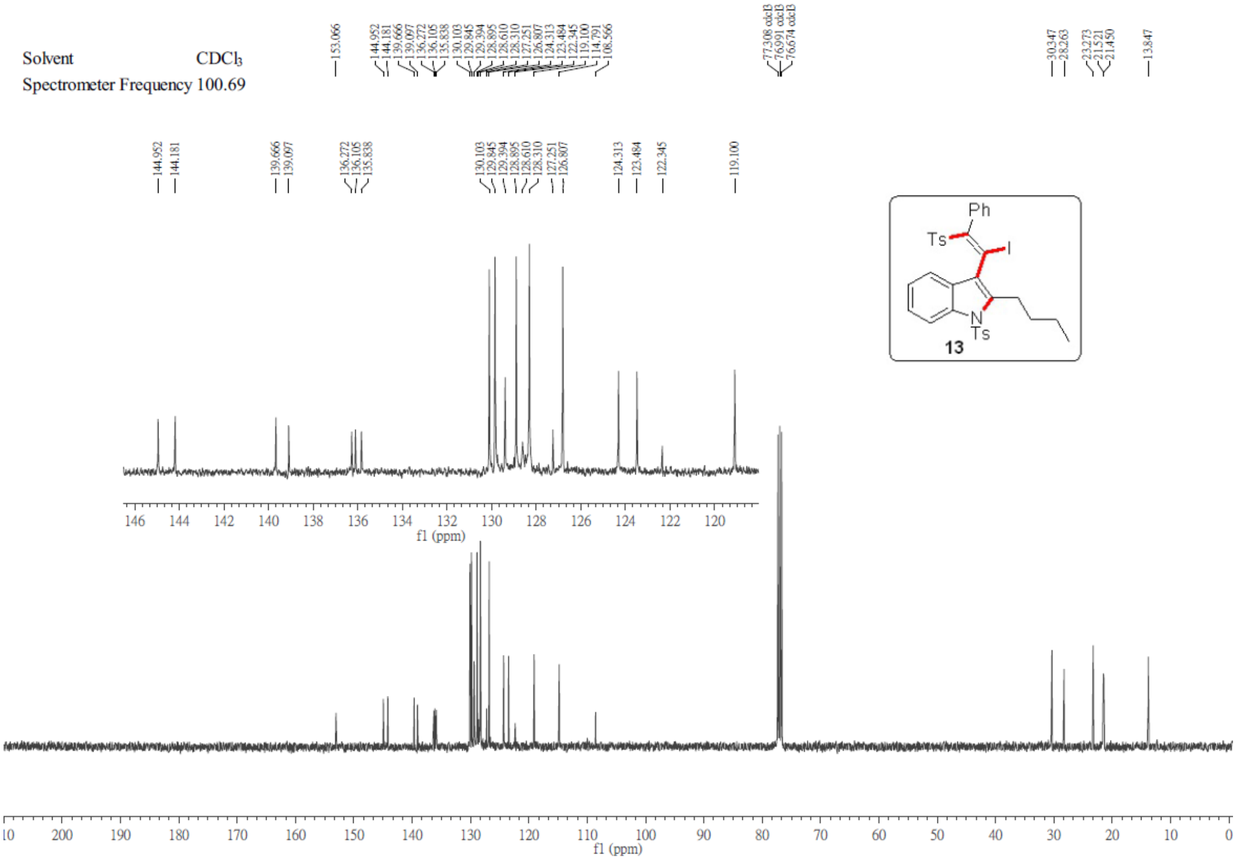

Supplementary Figure 100. ^{1}H (top) and ^{13}C (bottom) NMR spectra of compound **13**.

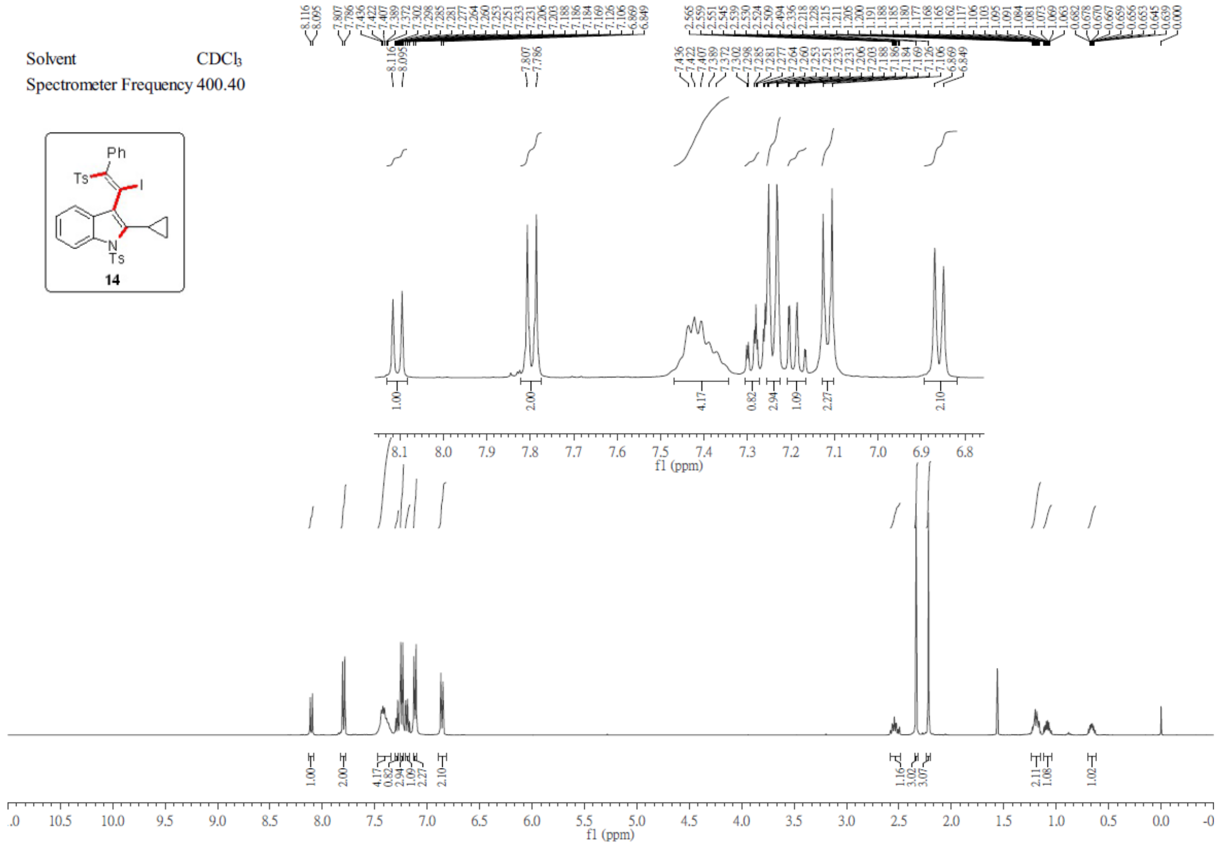

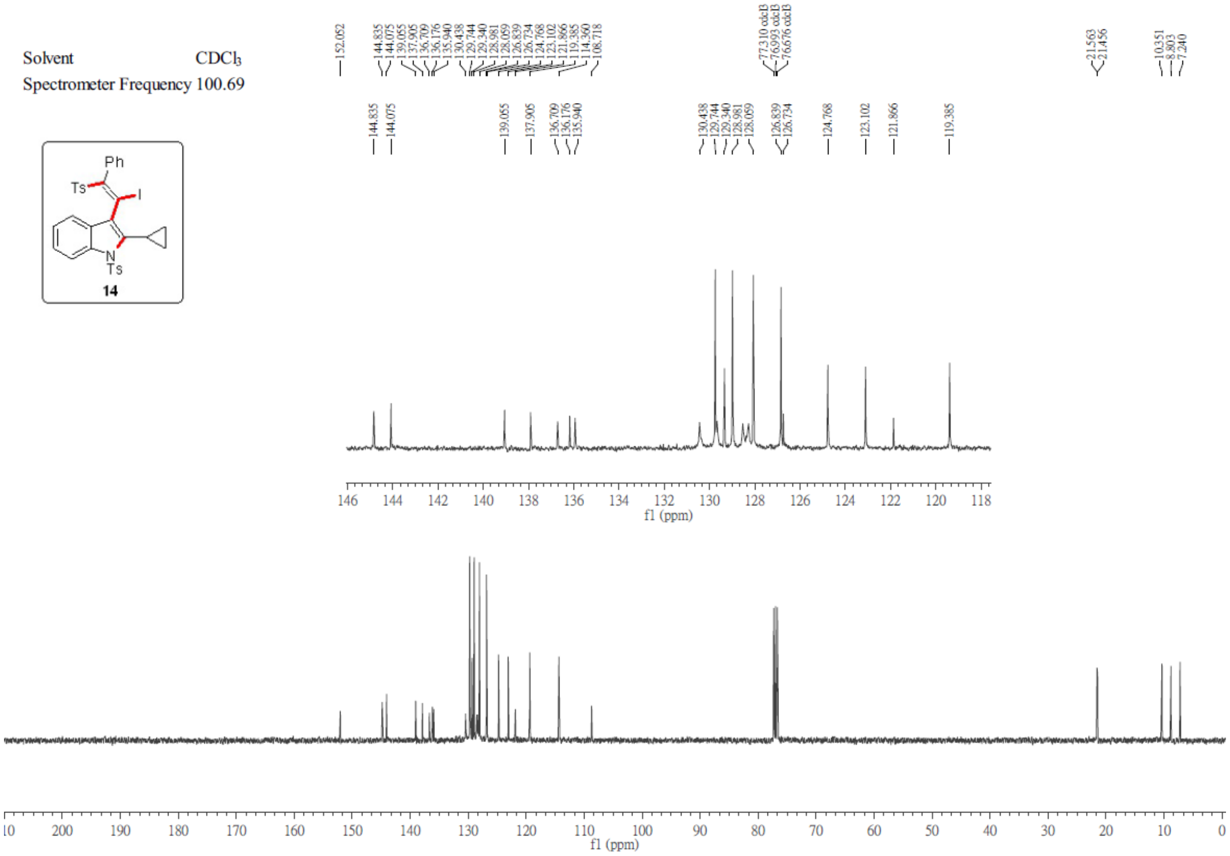

Supplementary Figure 101. ^{1}H (top) and ^{13}C (bottom) NMR spectra of compound **14**.

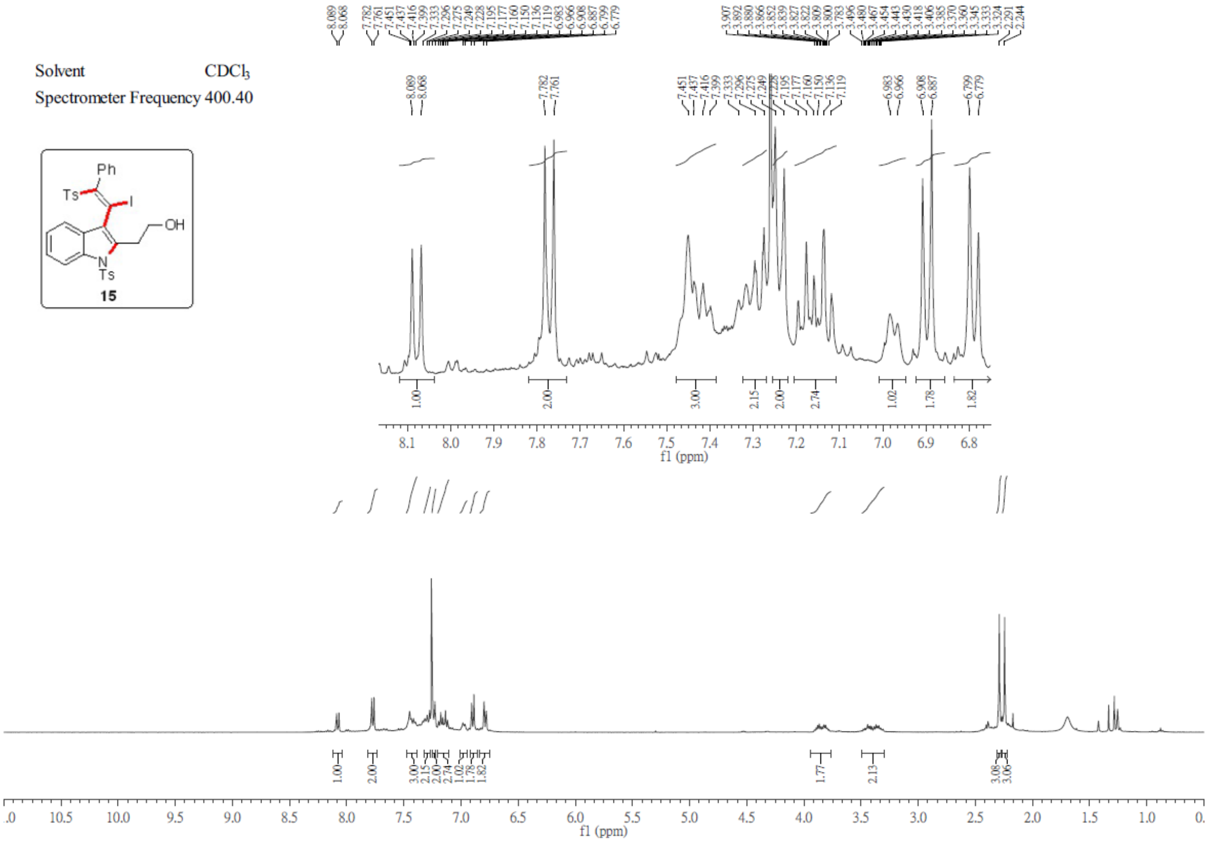

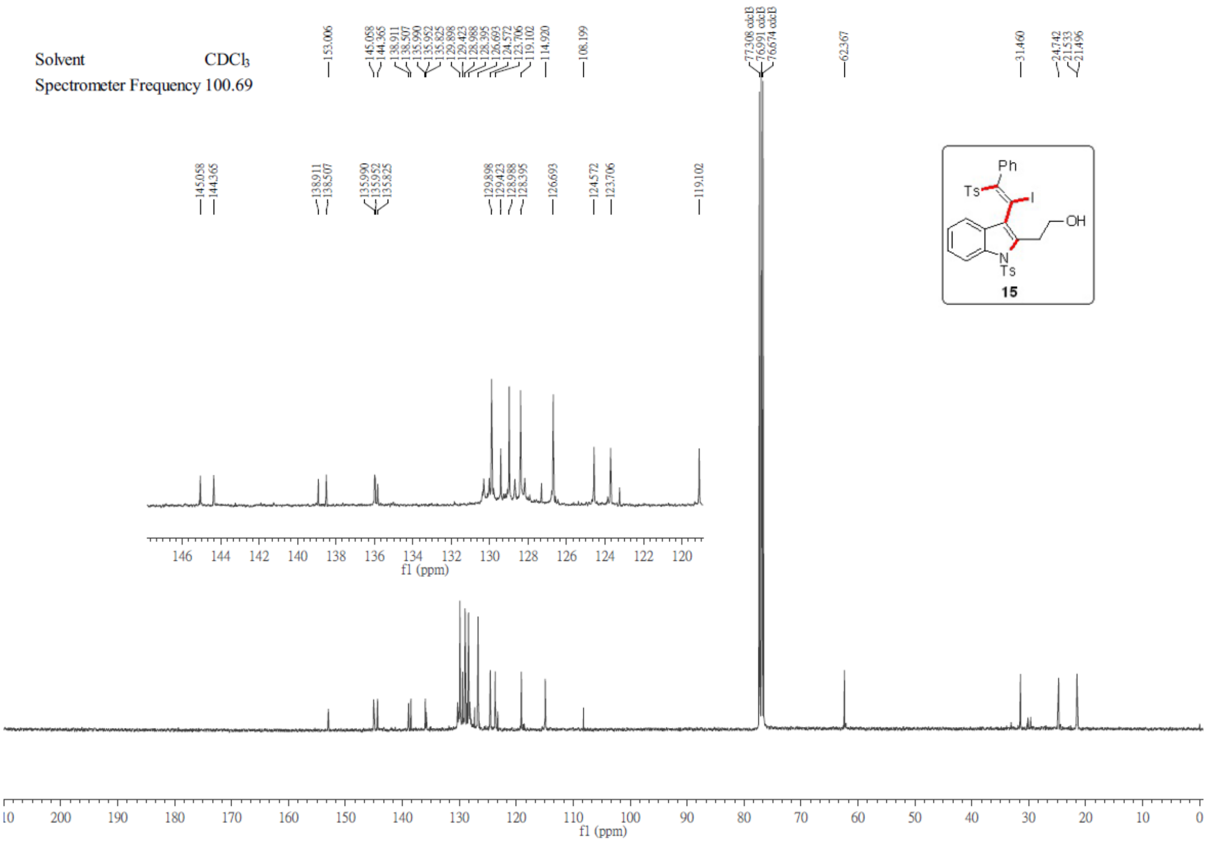

Supplementary Figure 102. ^{1}H (top) and ^{13}C (bottom) NMR spectra of compound **15**.

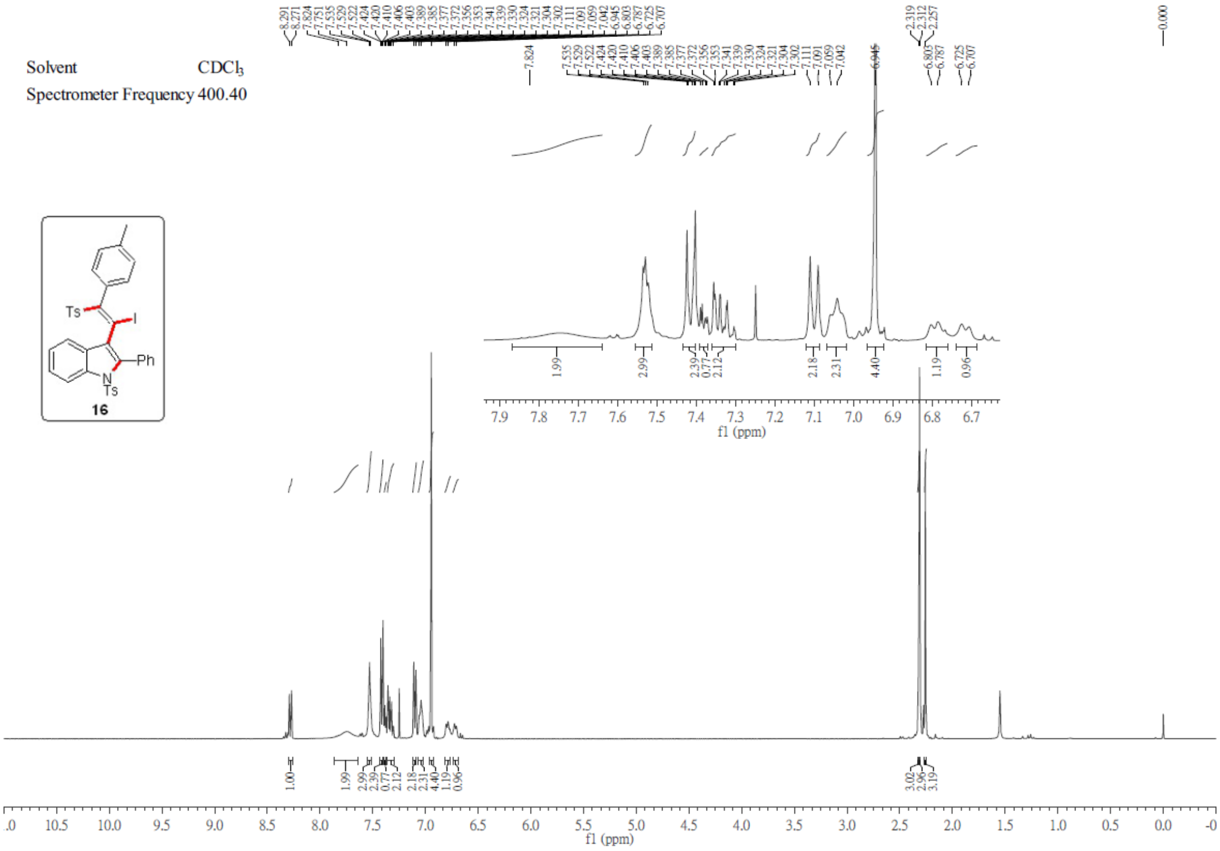

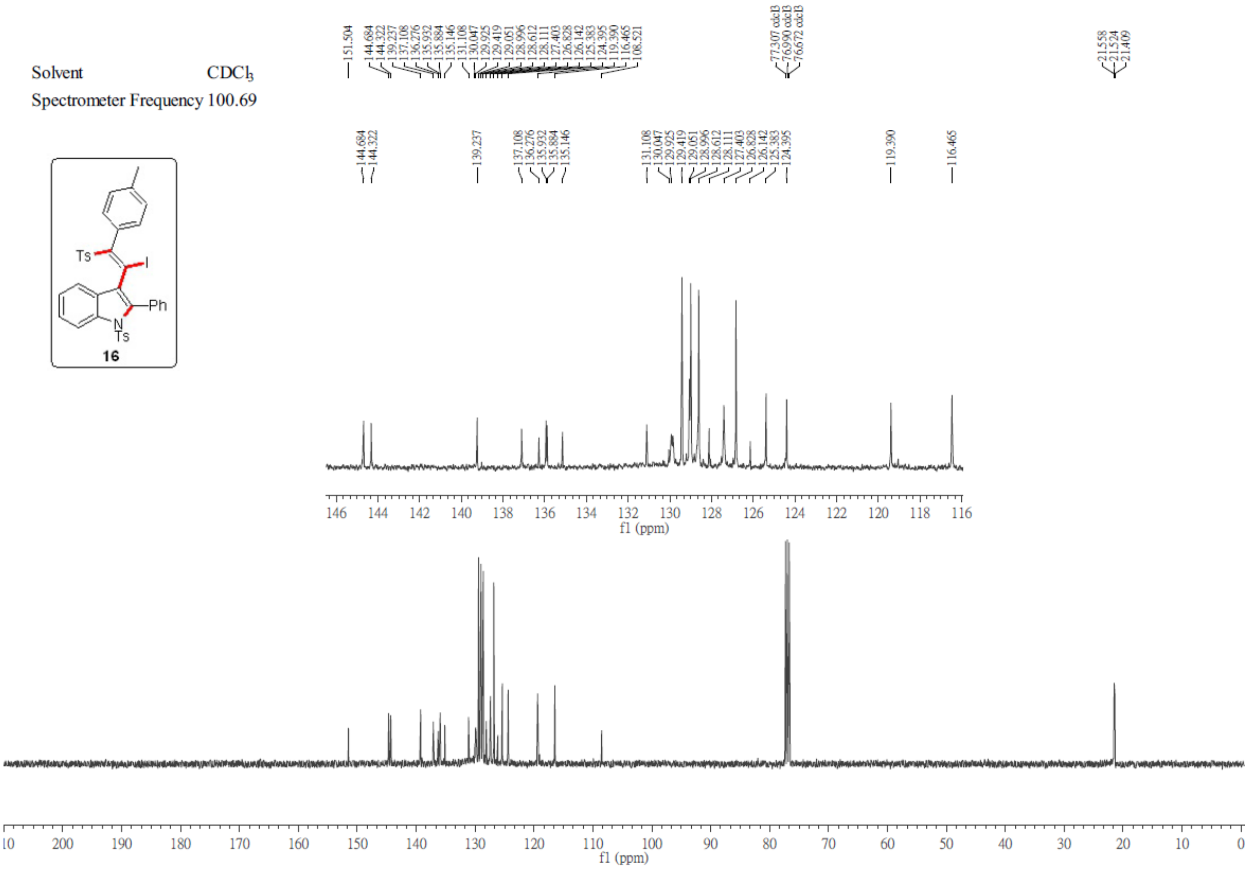

Supplementary Figure 103. ^{1}H (top) and ^{13}C (bottom) NMR spectra of compound **16**.

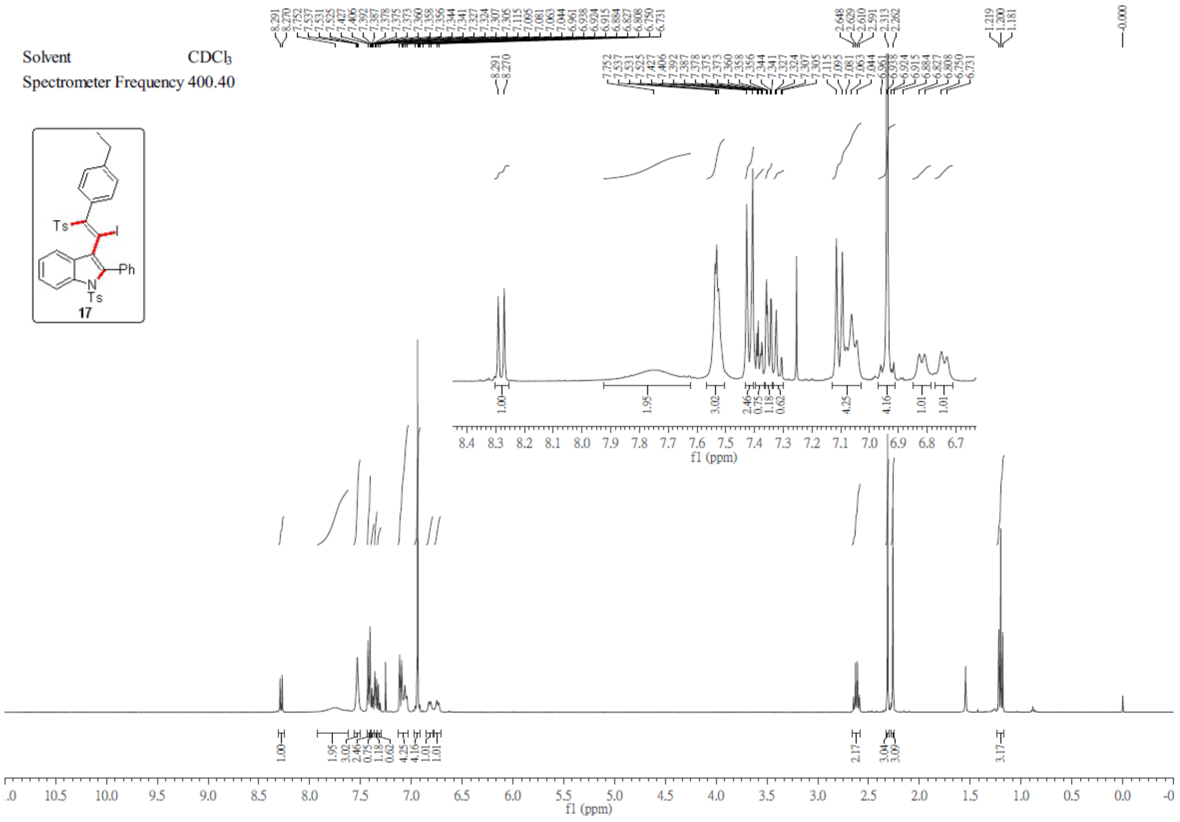

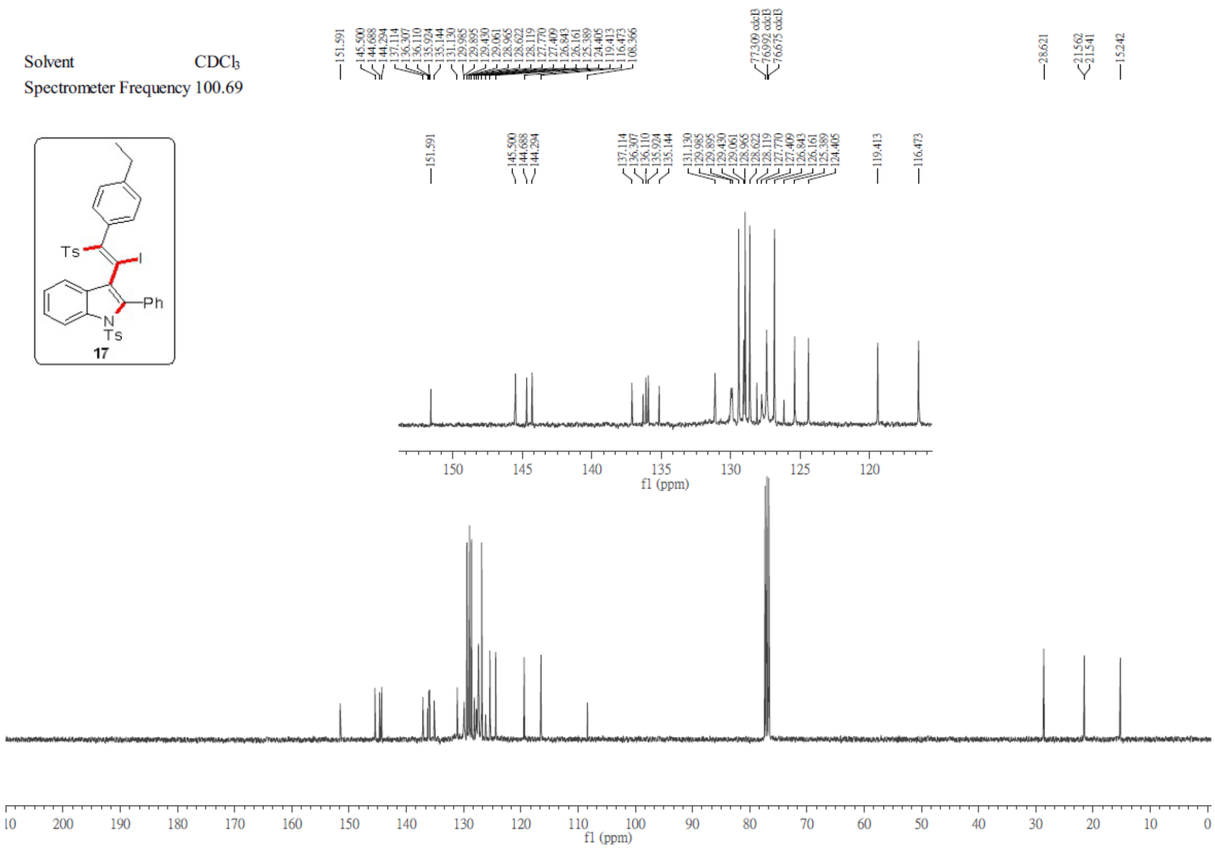

Supplementary Figure 104. ^{1}H (top) and ^{13}C (bottom) NMR spectra of compound **17**.

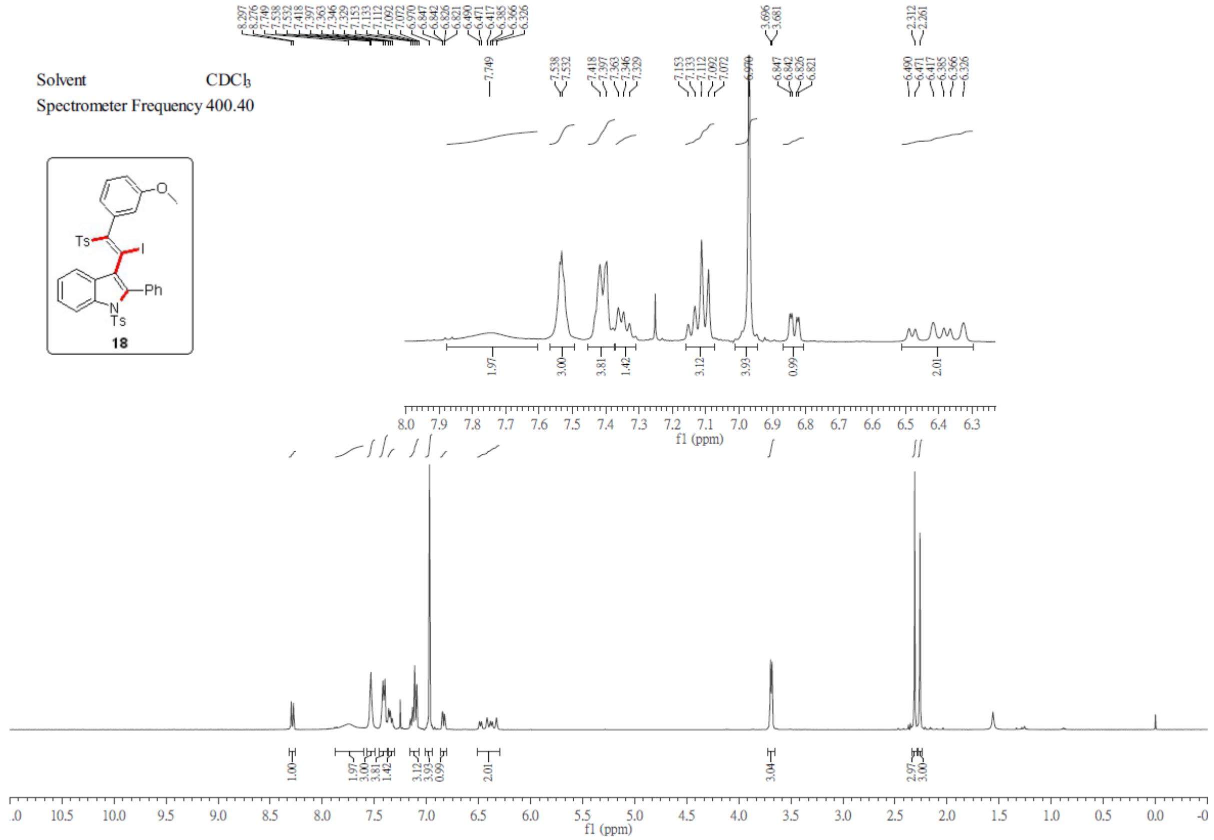

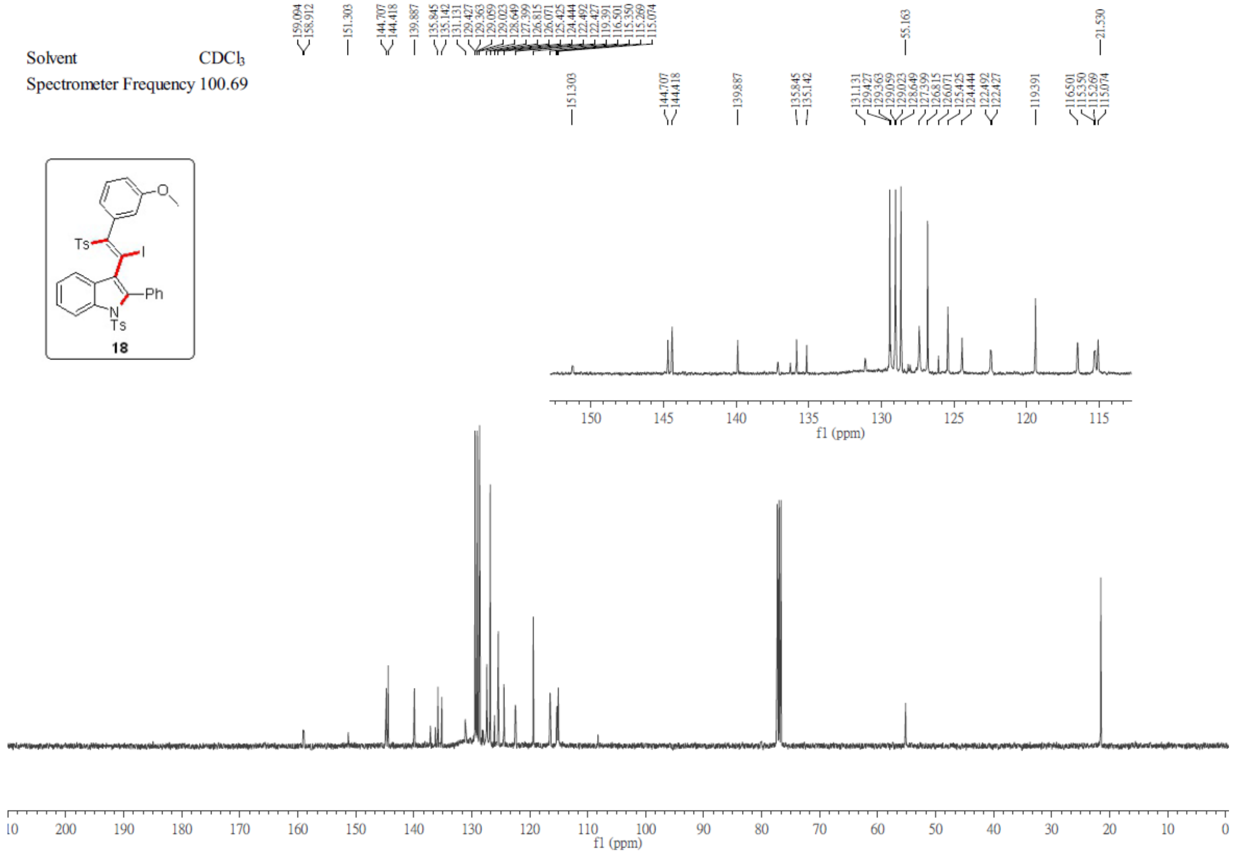

Supplementary Figure 105. ^{1}H (top) and ^{13}C (bottom) NMR spectra of compound **18**.

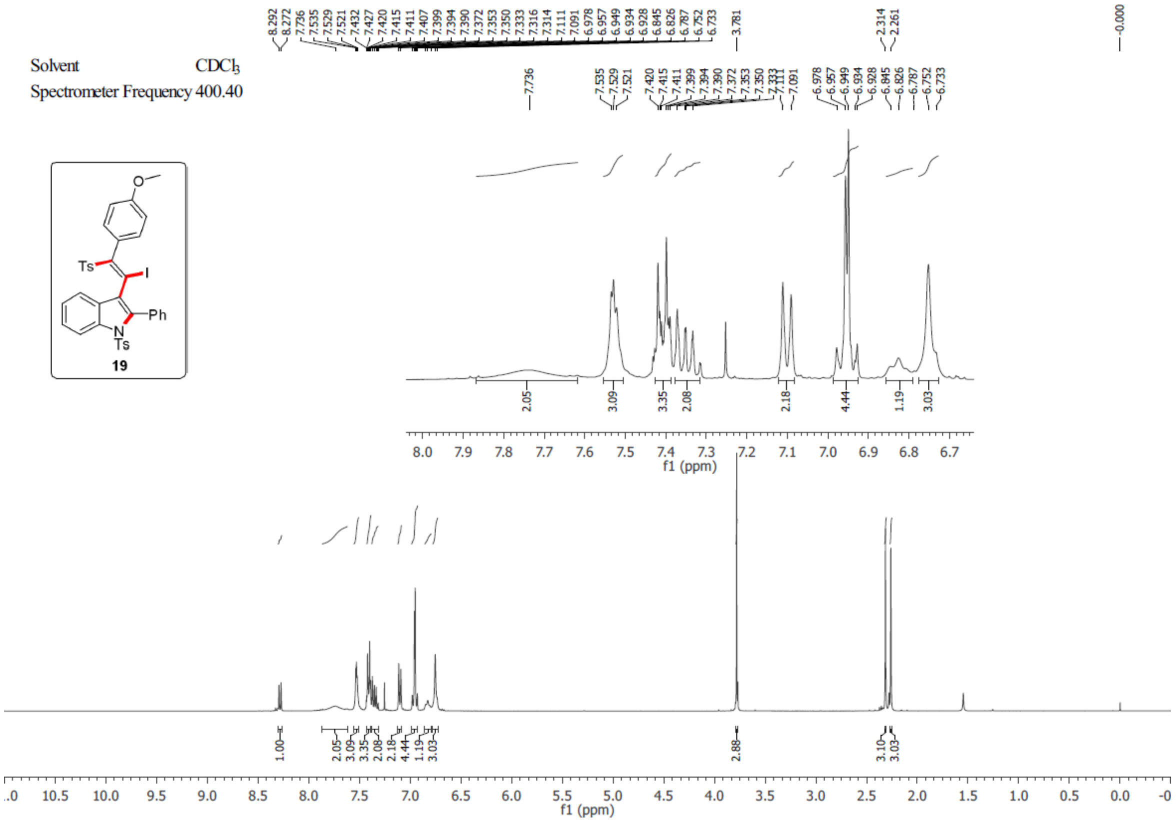

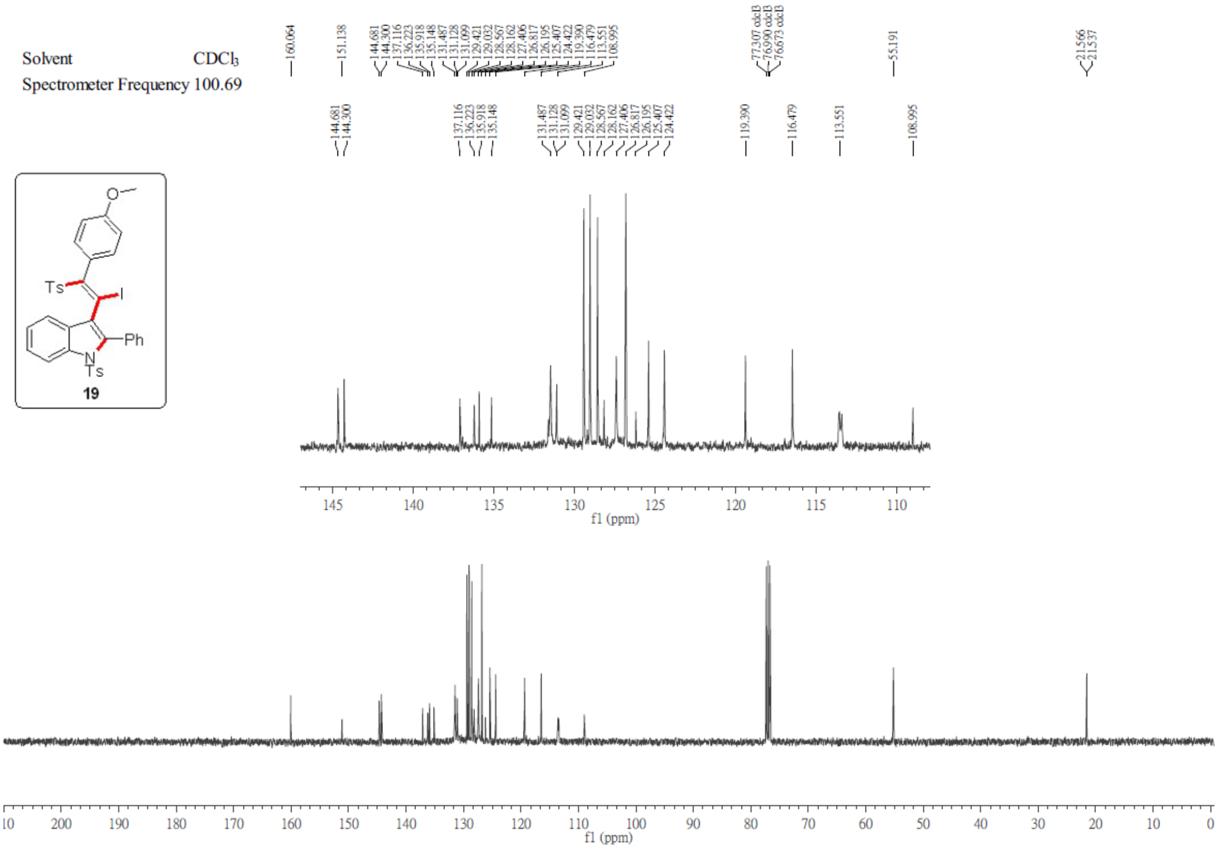

Supplementary Figure 106. ^{1}H (top) and ^{13}C (bottom) NMR spectra of compound **19**.

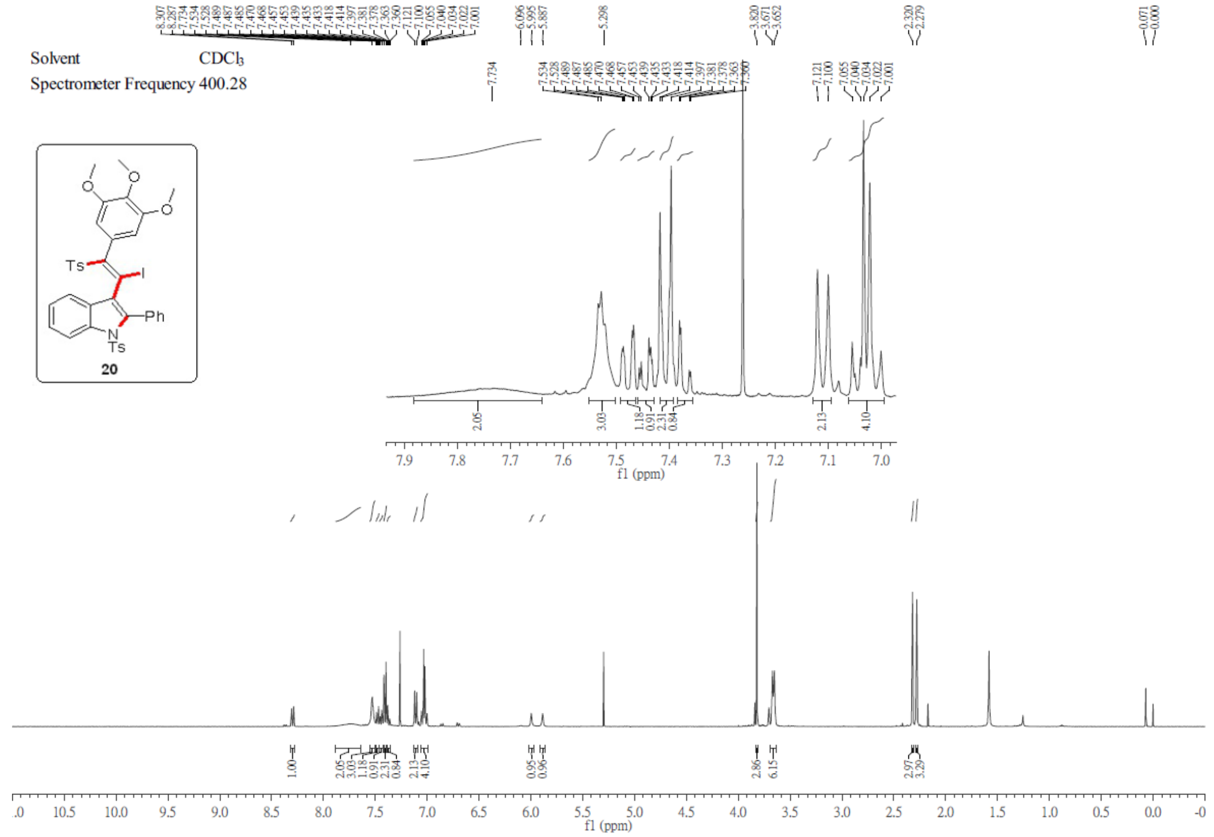

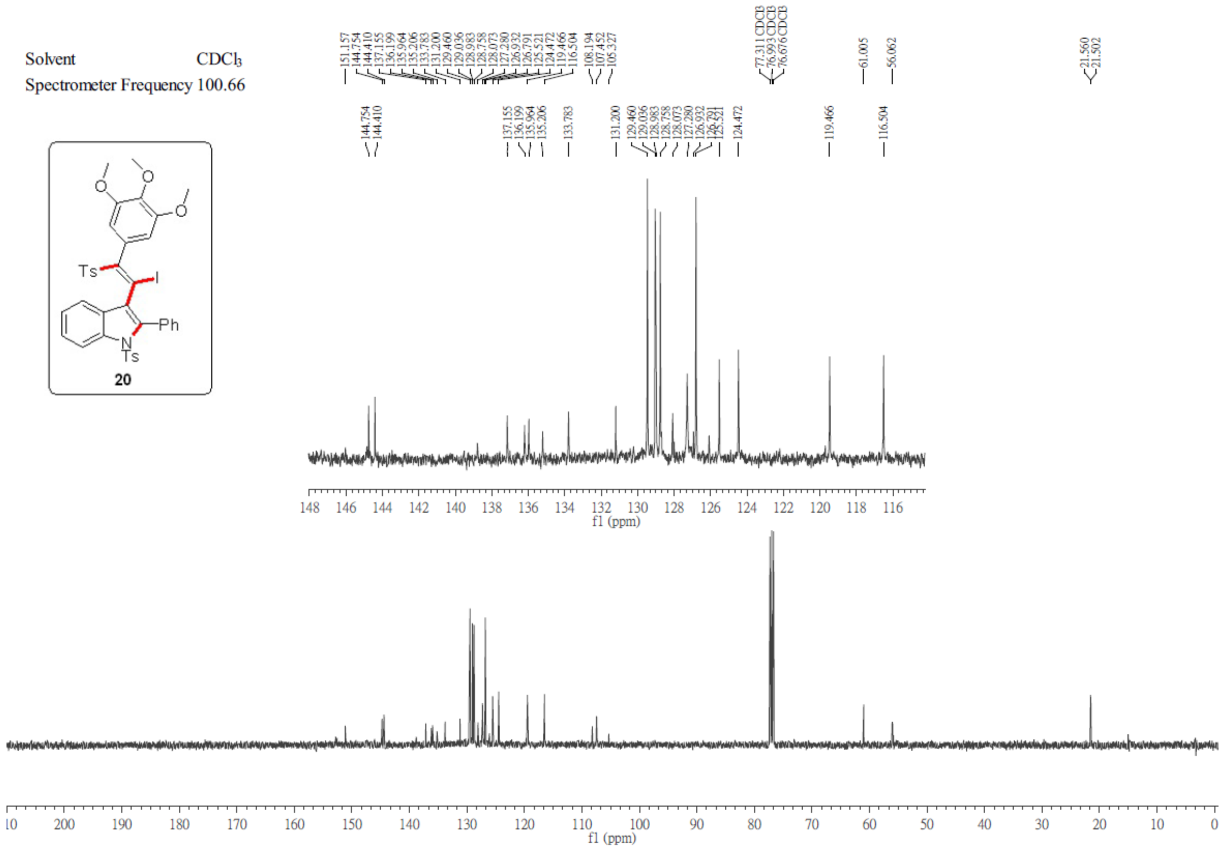

Supplementary Figure 107. ^{1}H (top) and ^{13}C (bottom) NMR spectra of compound **20**.

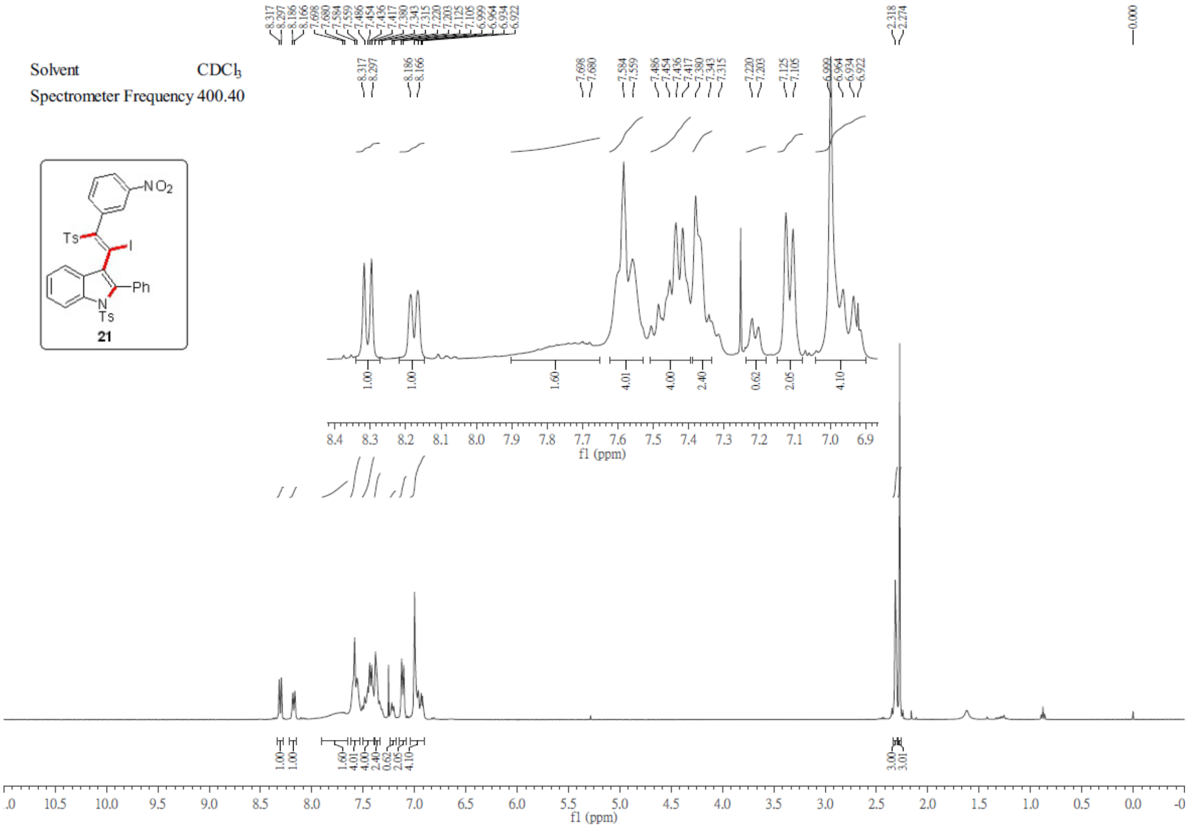

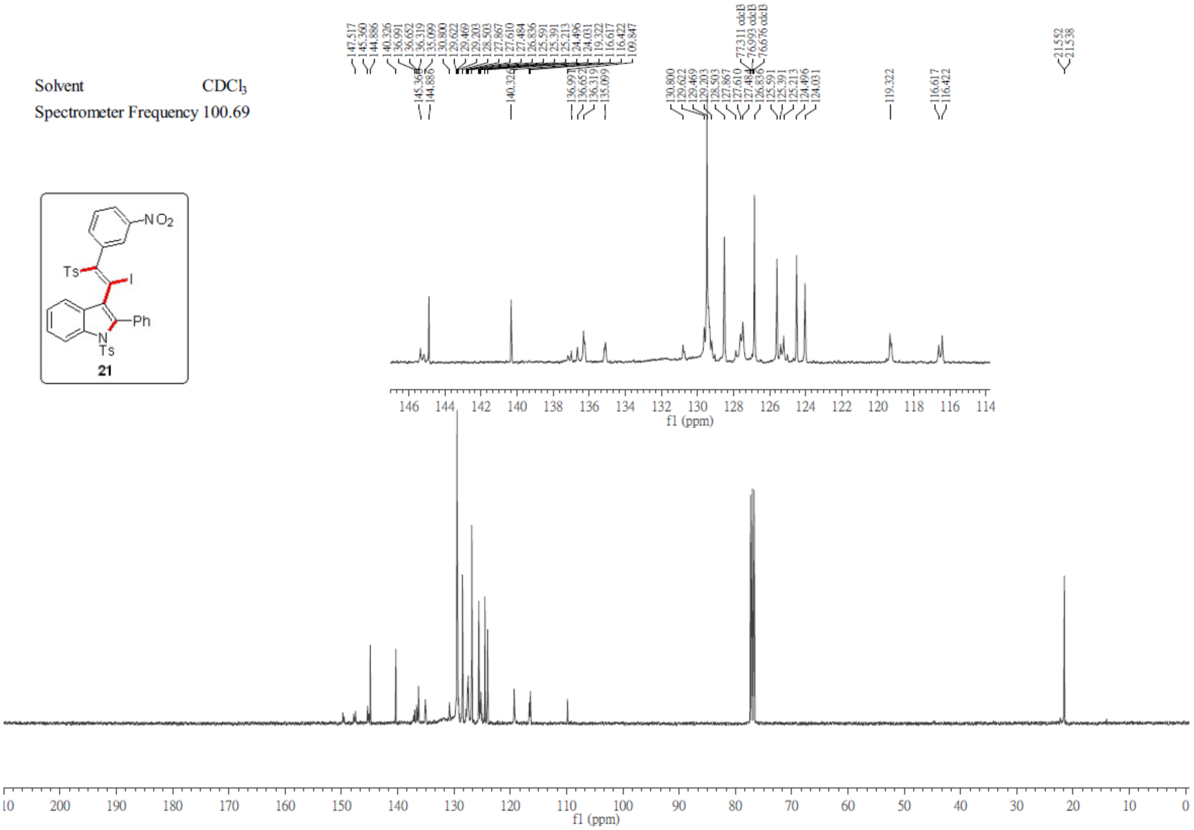

Supplementary Figure 108. ^{1}H (top) and ^{13}C (bottom) NMR spectra of compound **21**.

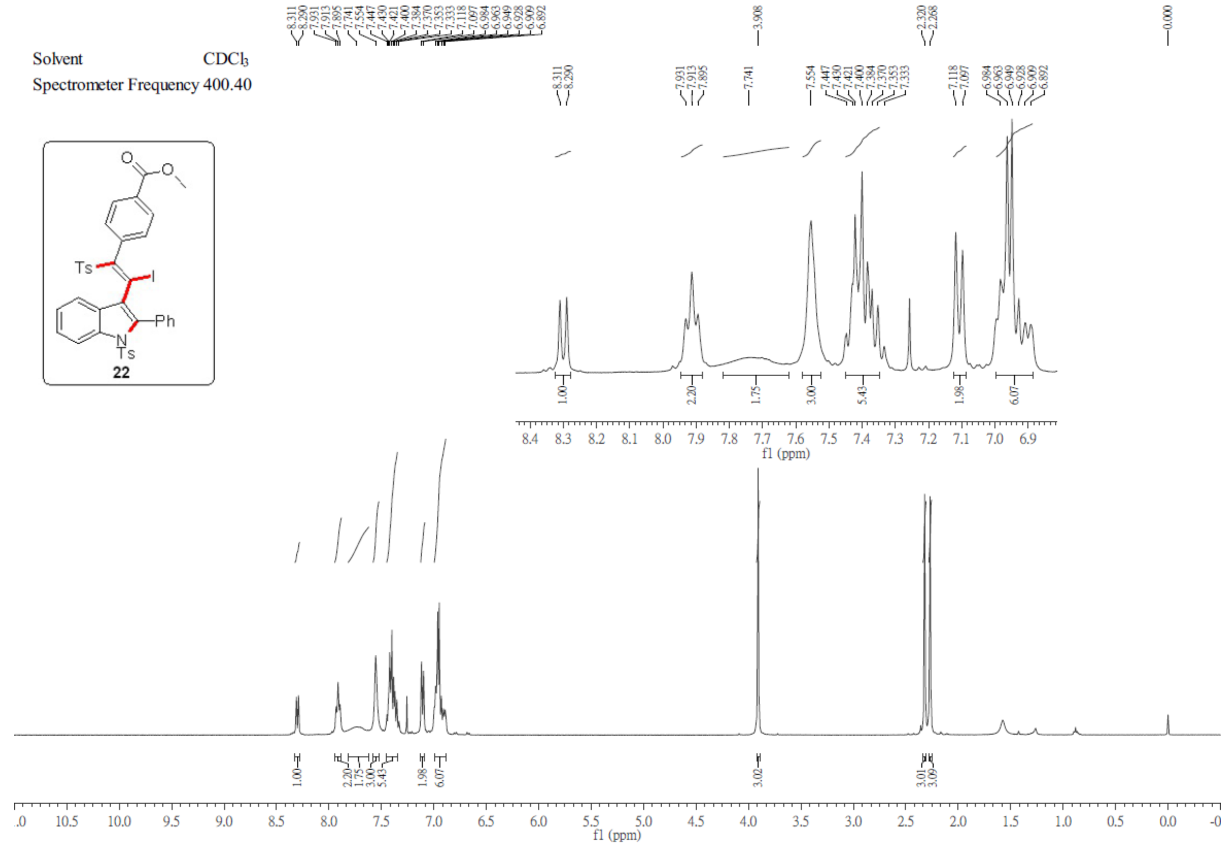

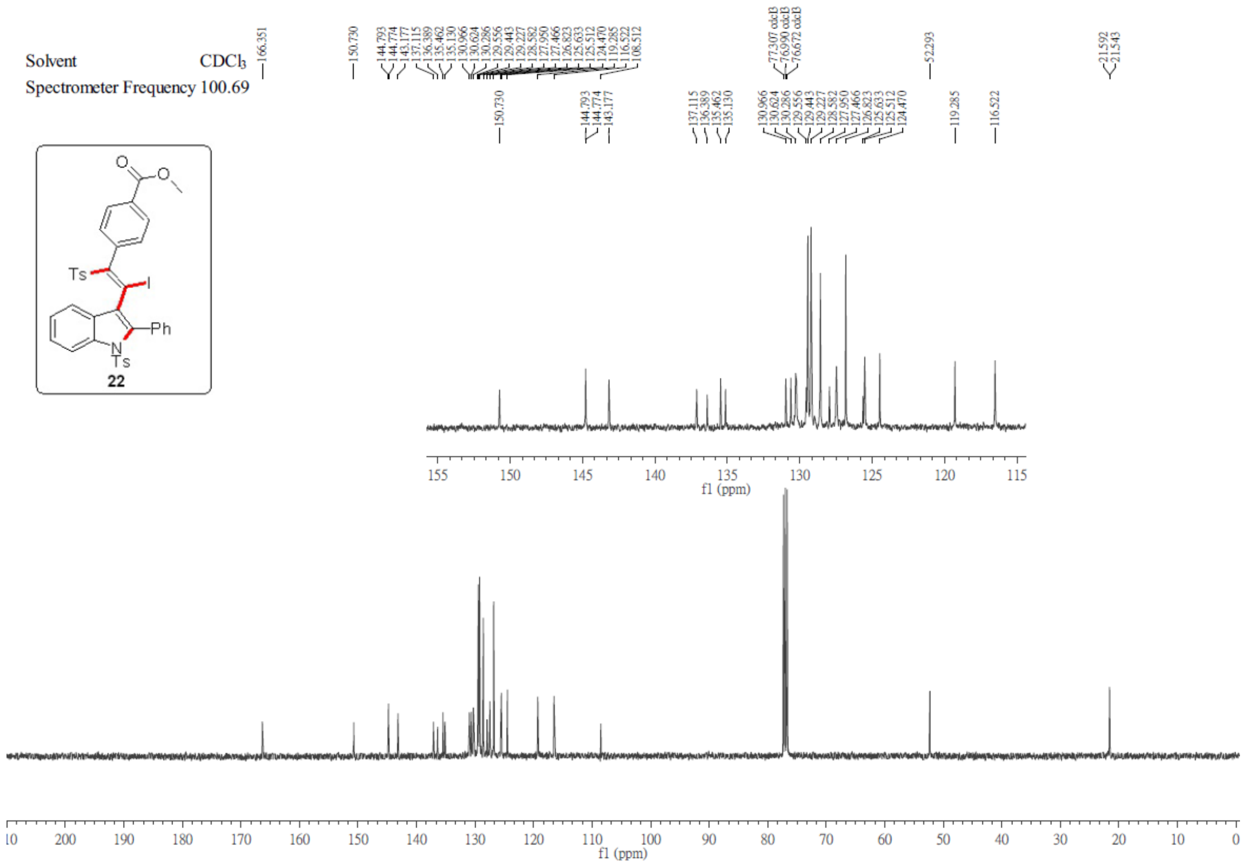

Supplementary Figure 109. ^{1}H (top) and ^{13}C (bottom) NMR spectra of compound **22**.

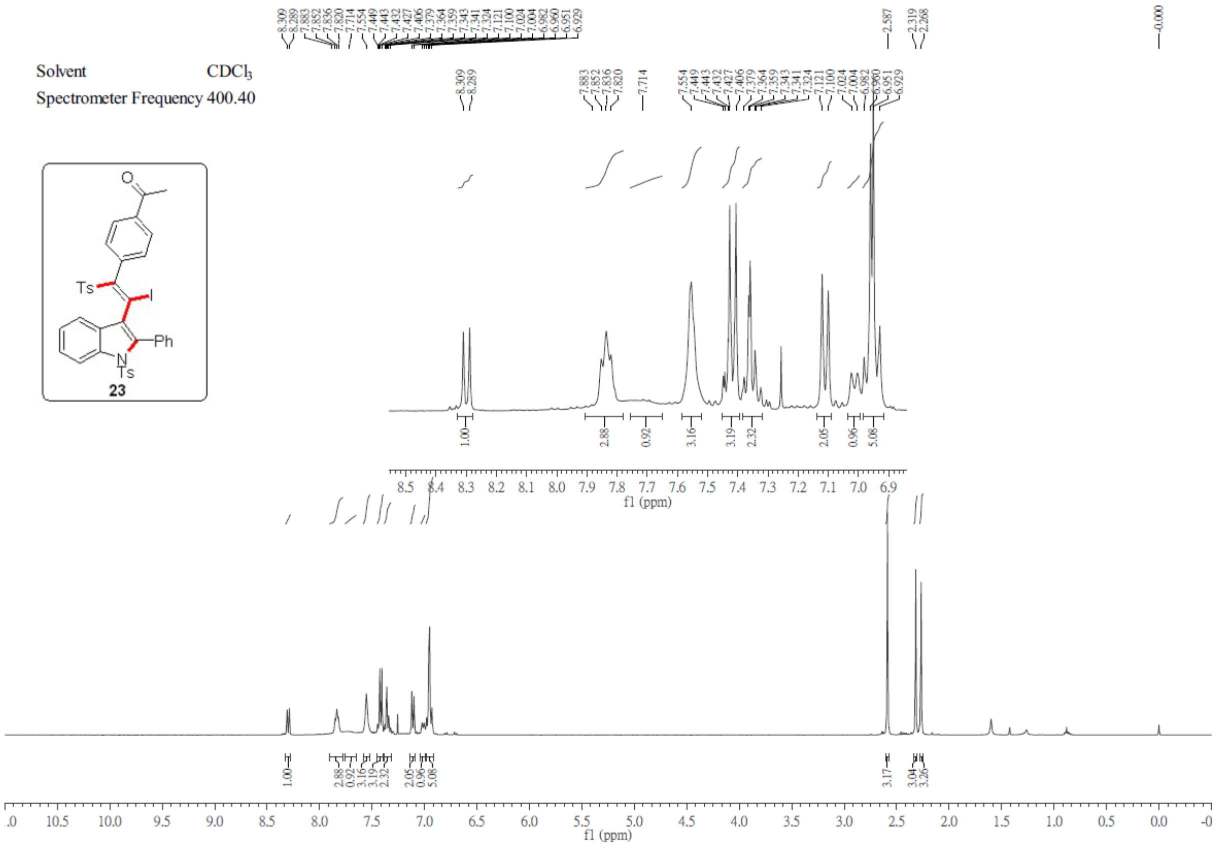

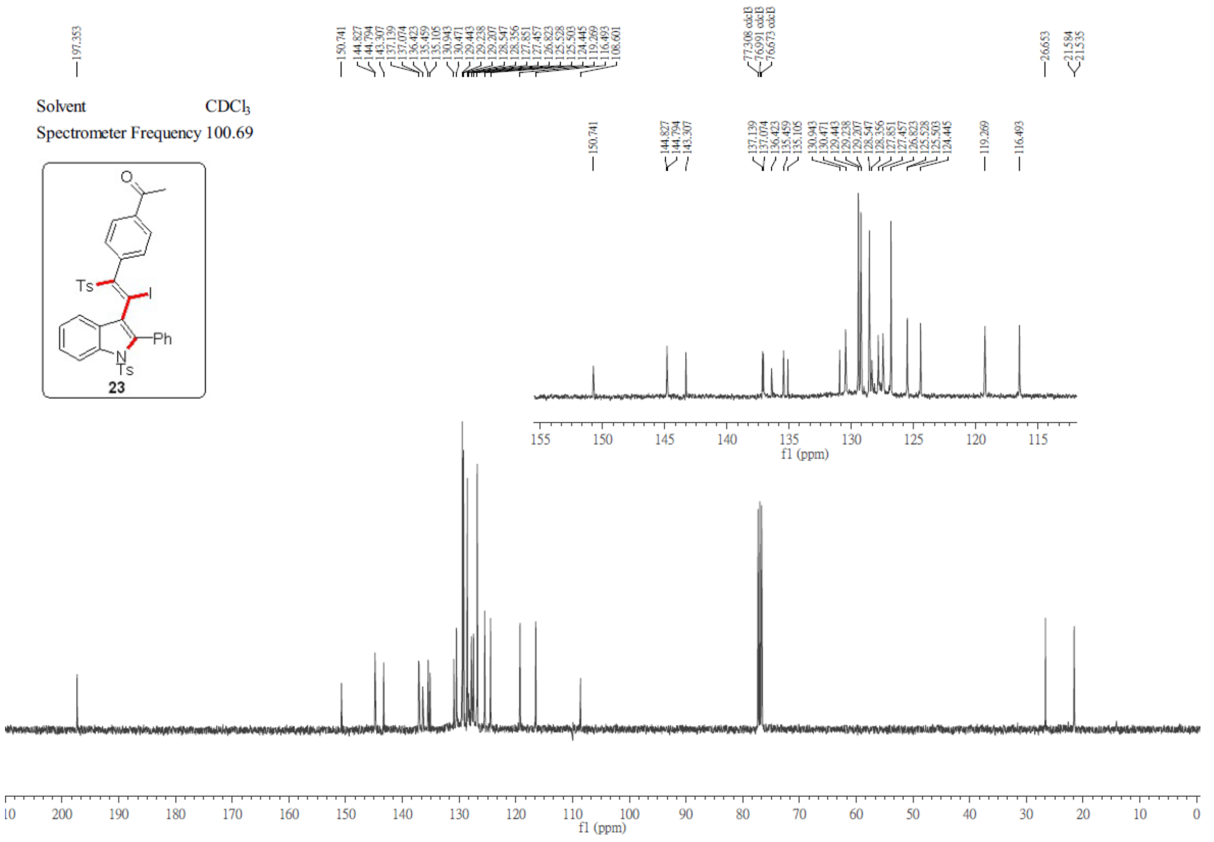

Supplementary Figure 110. ^{1}H (top) and ^{13}C (bottom) NMR spectra of compound **23**.

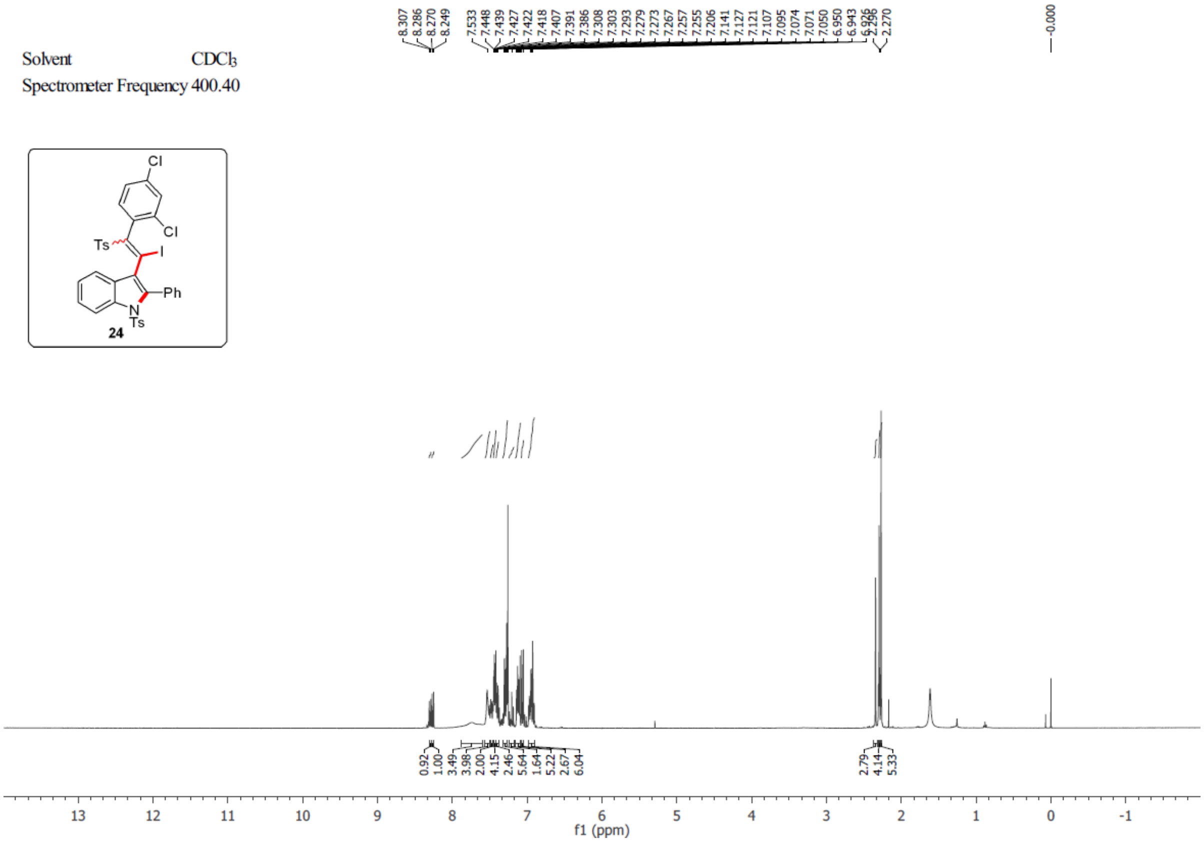

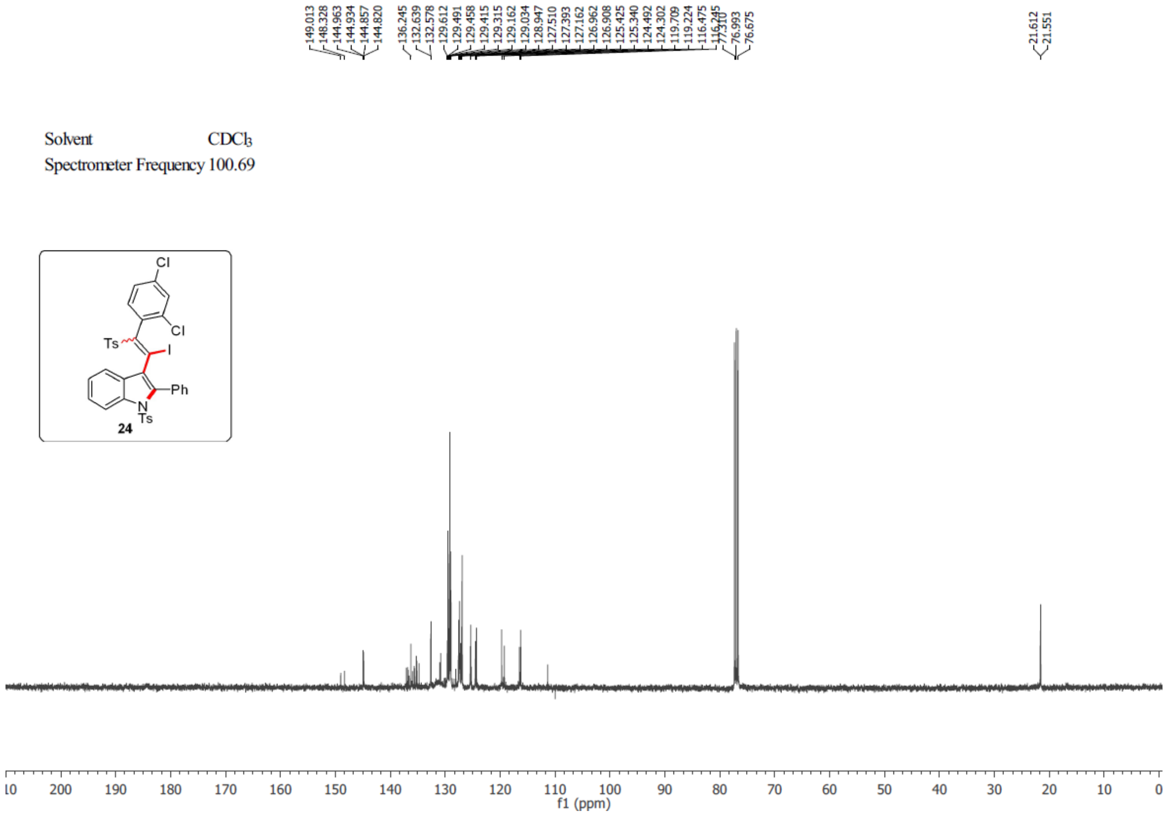

Supplementary Figure 111. ^{1}H (top) and ^{13}C (bottom) NMR spectra of compound **24**.

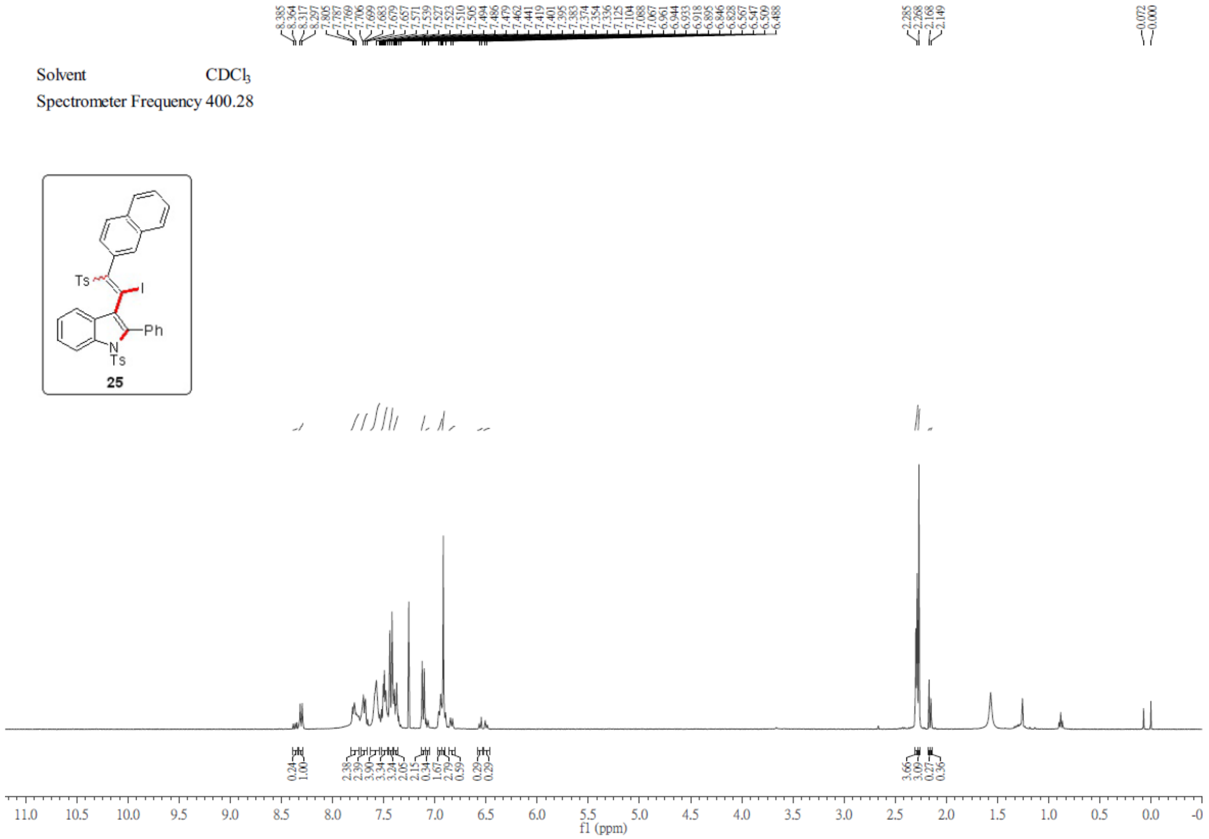

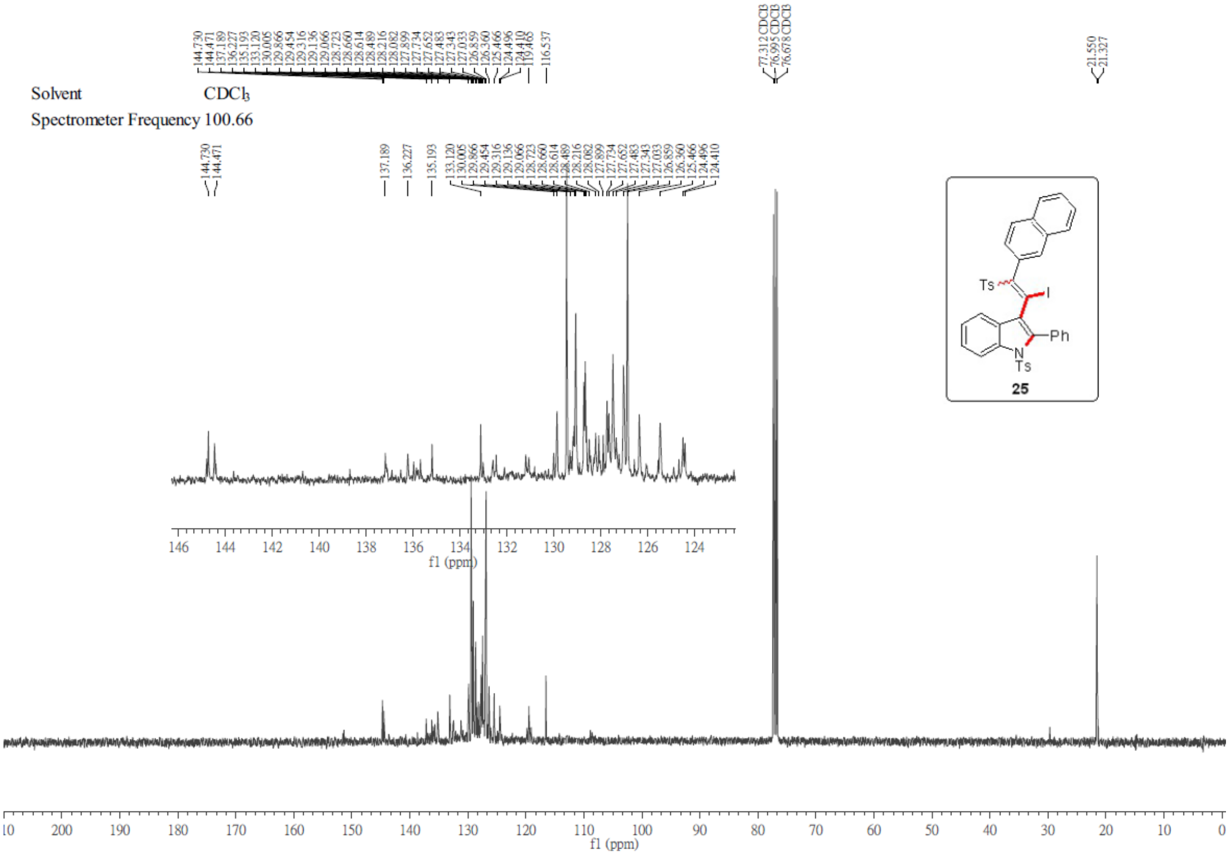

Supplementary Figure 112. ^{1}H (top) and ^{13}C (bottom) NMR spectra of compound **25**.

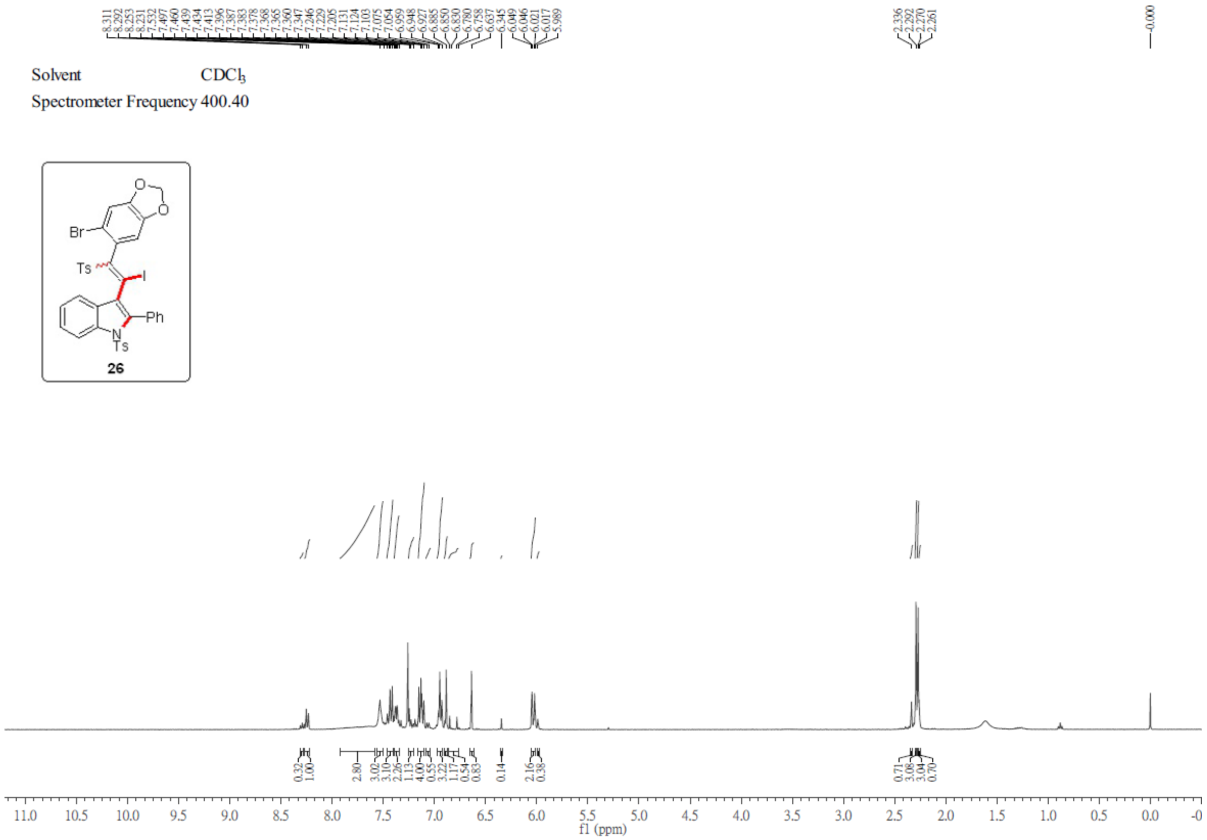

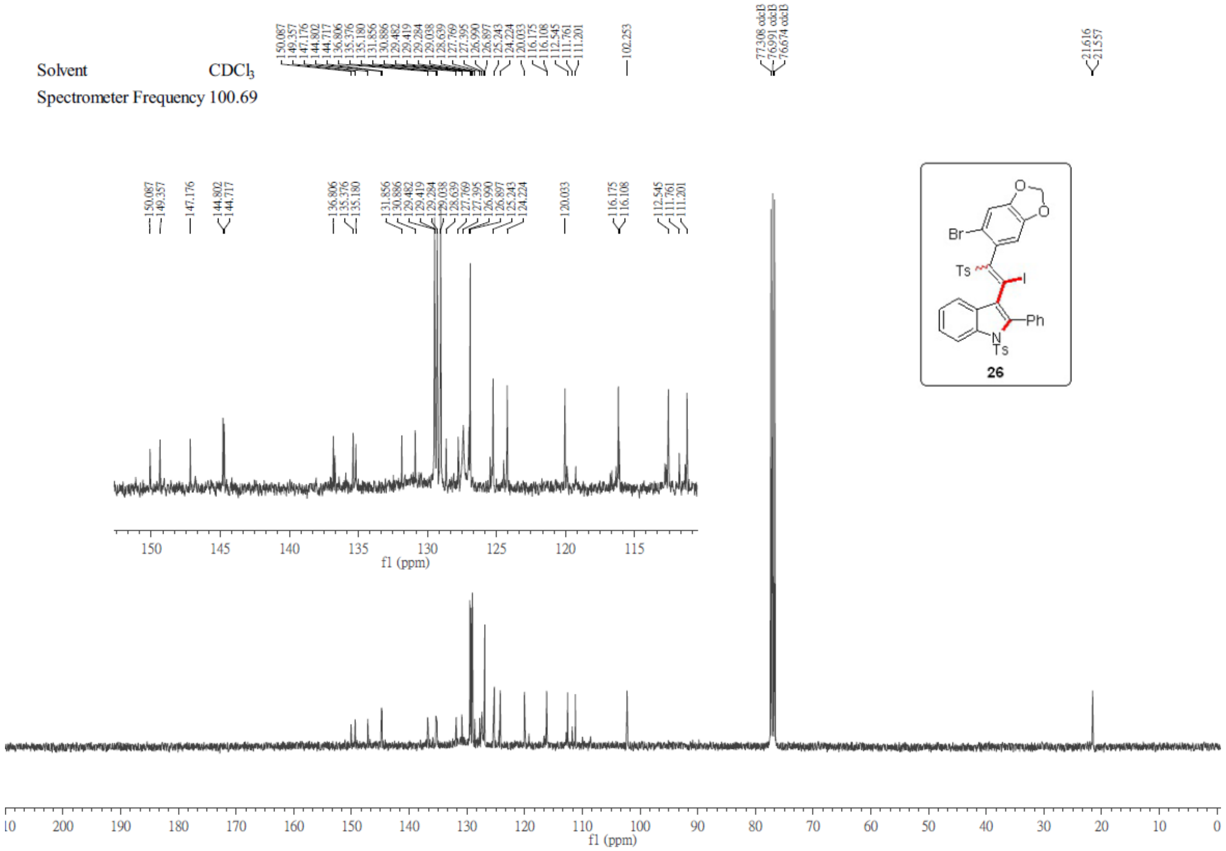

Supplementary Figure 113. ^{1}H (top) and ^{13}C (bottom) NMR spectra of compound **26**.

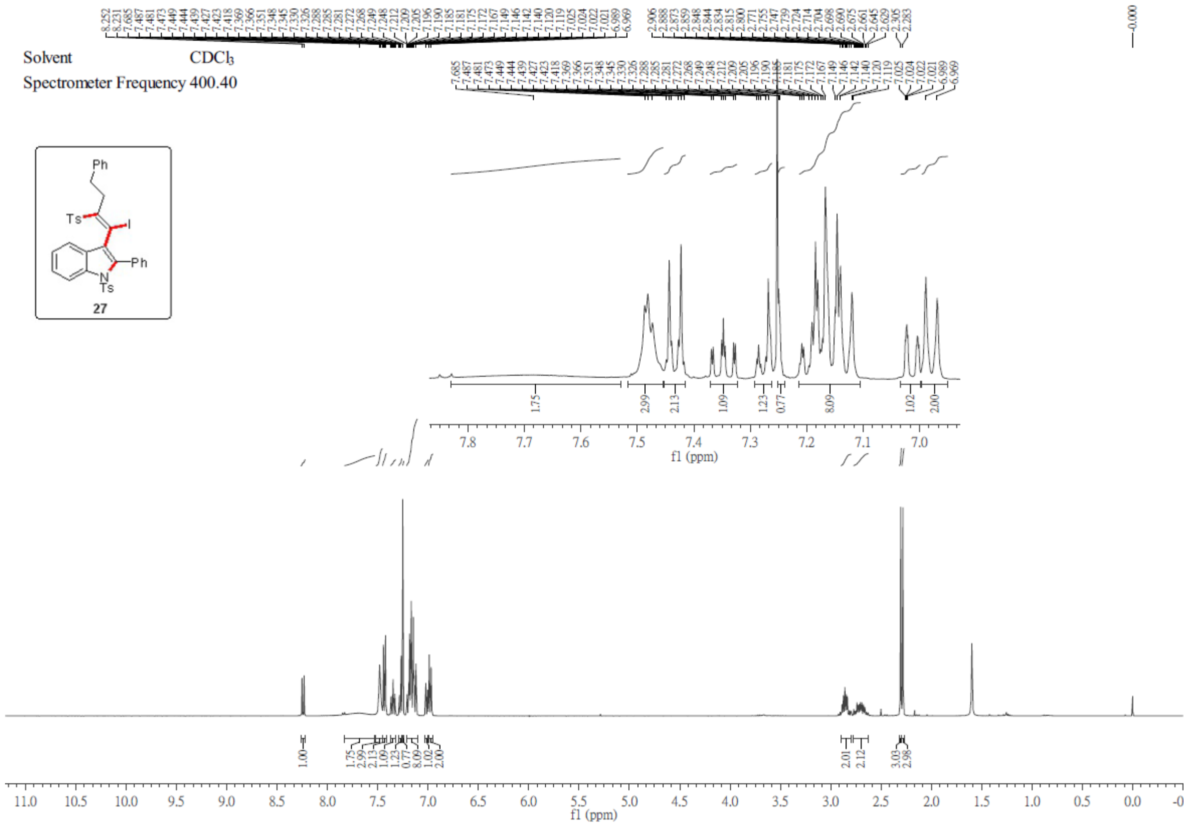

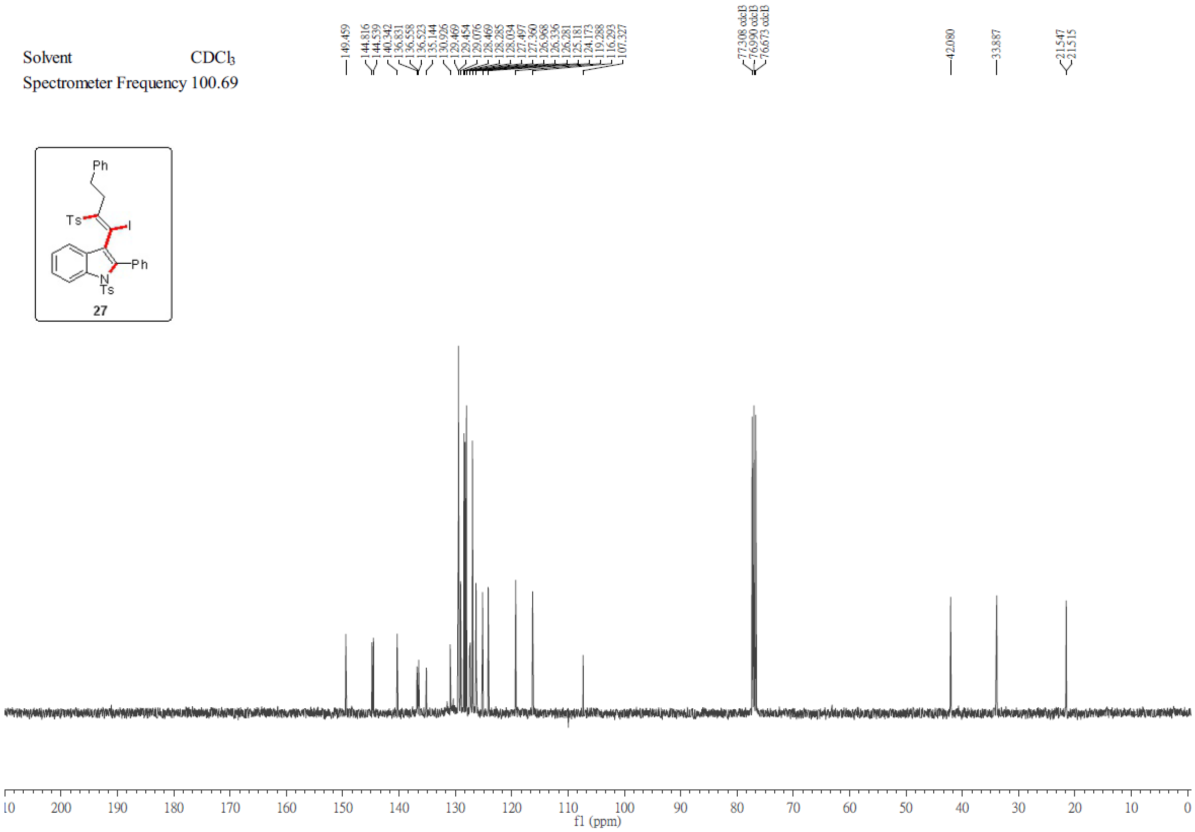

Supplementary Figure 114. ^{1}H (top) and ^{13}C (bottom) NMR spectra of compound **27**.

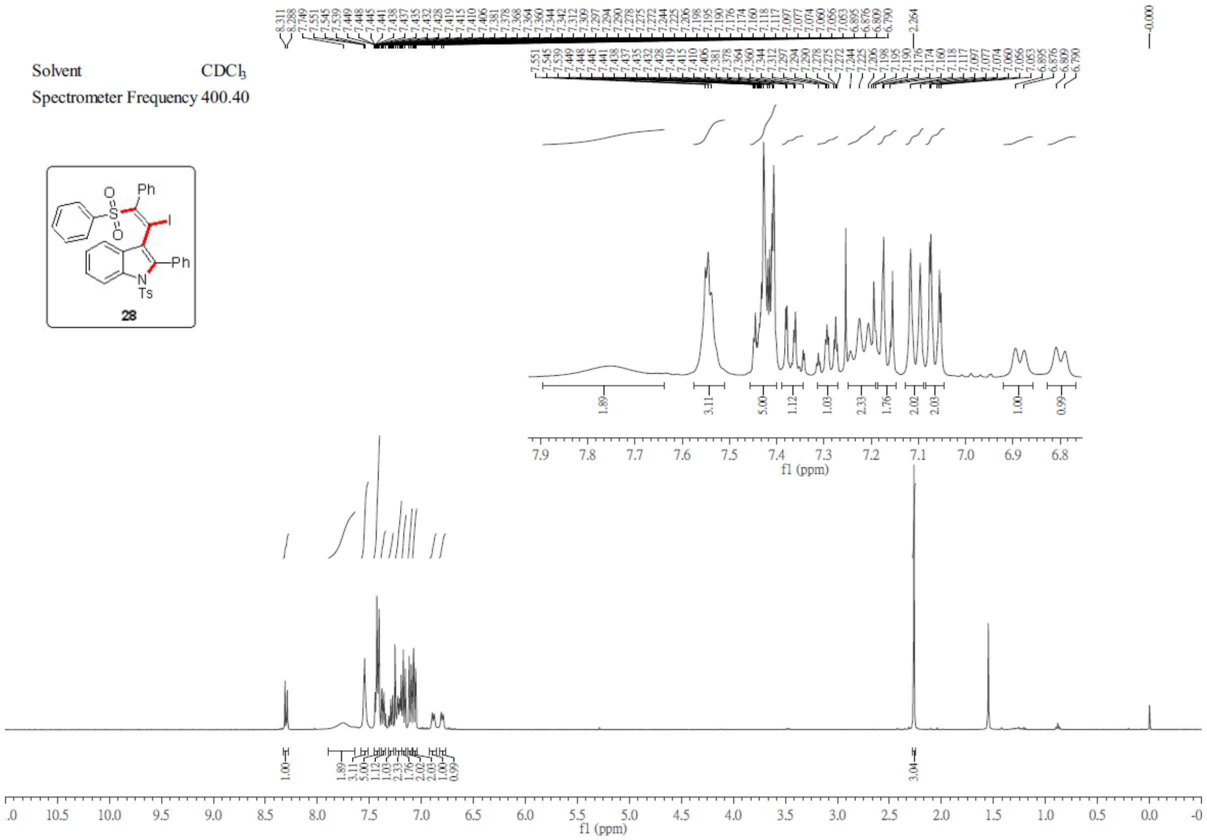

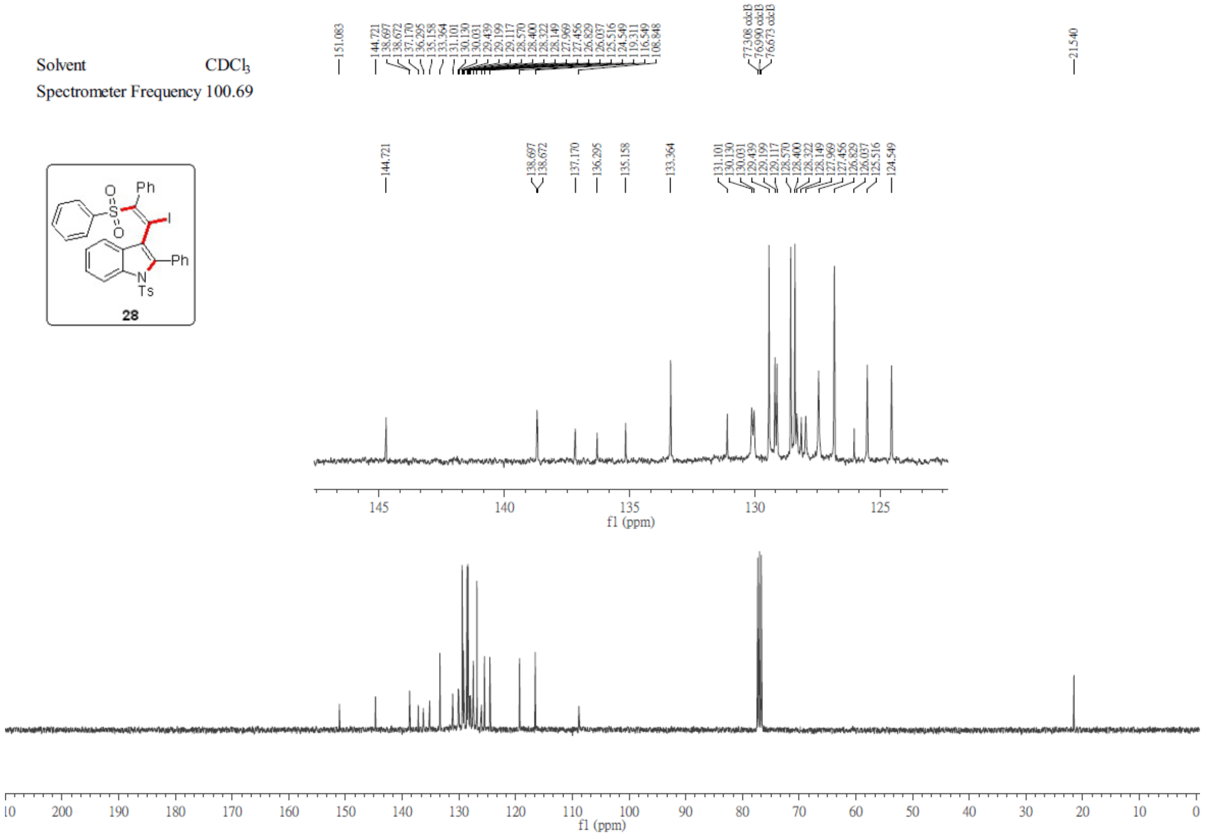

Supplementary Figure 115. ^{1}H (top) and ^{13}C (bottom) NMR spectra of compound **28**.

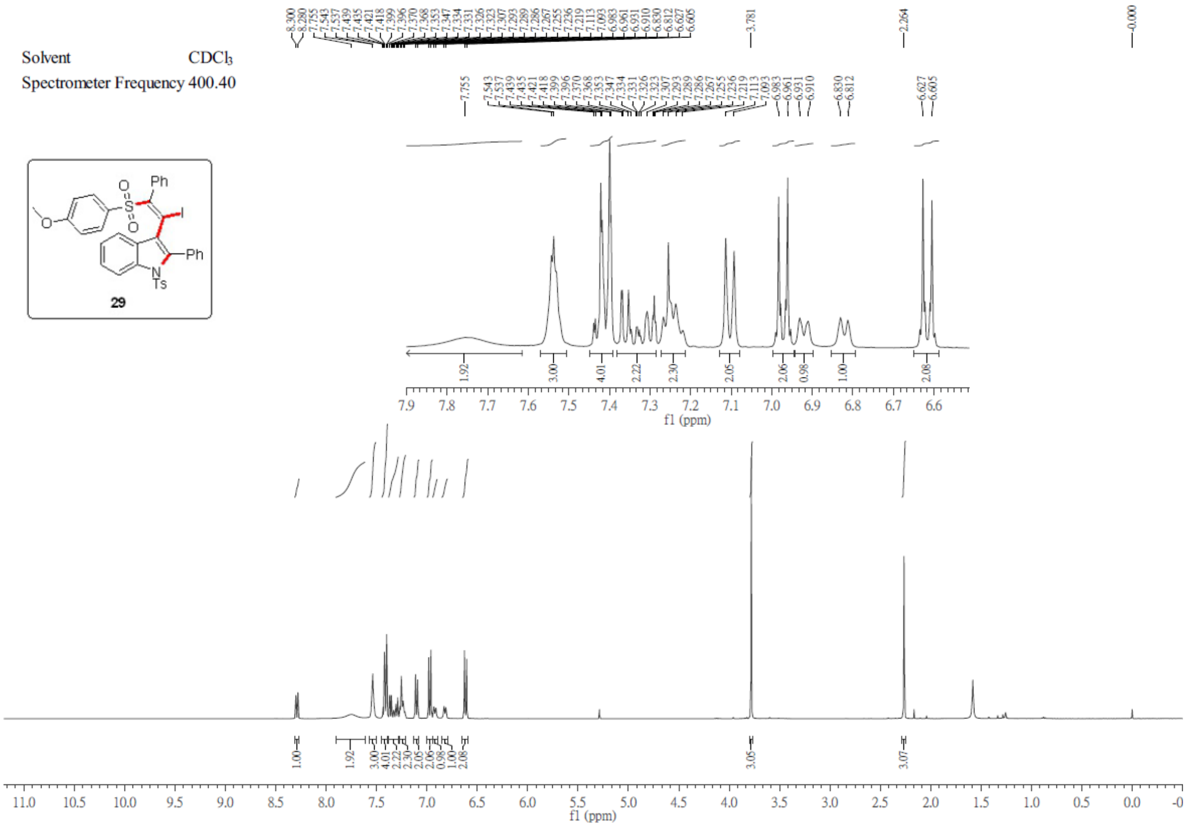

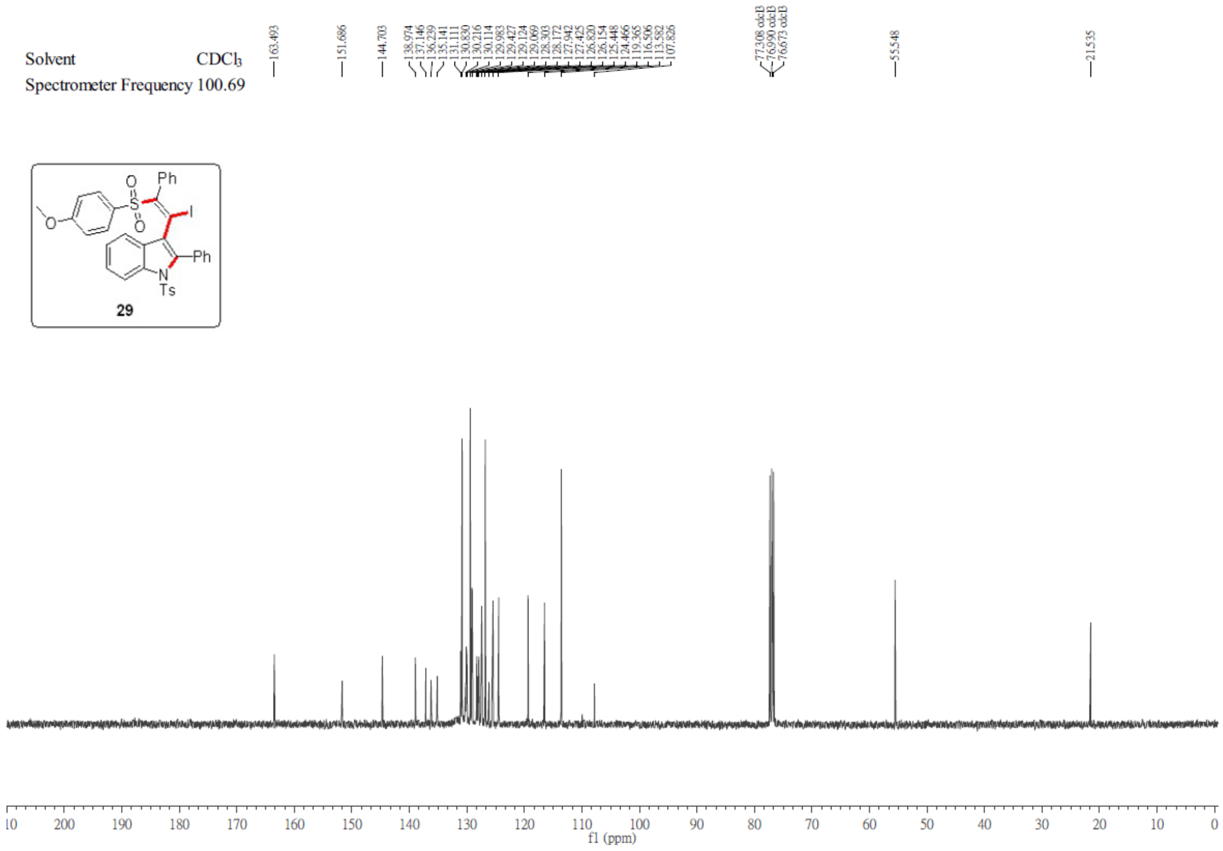

Supplementary Figure 116. ^{1}H (top) and ^{13}C (bottom) NMR spectra of compound **29**.

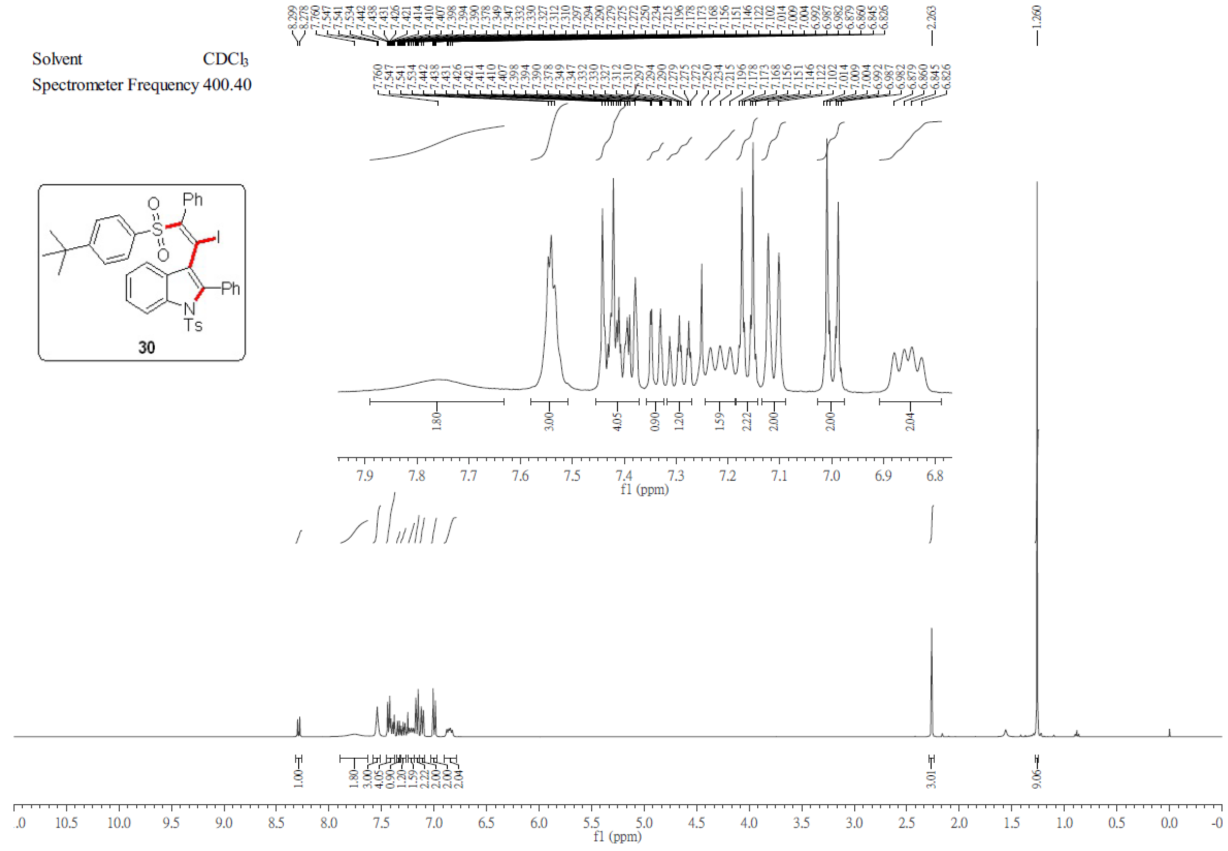

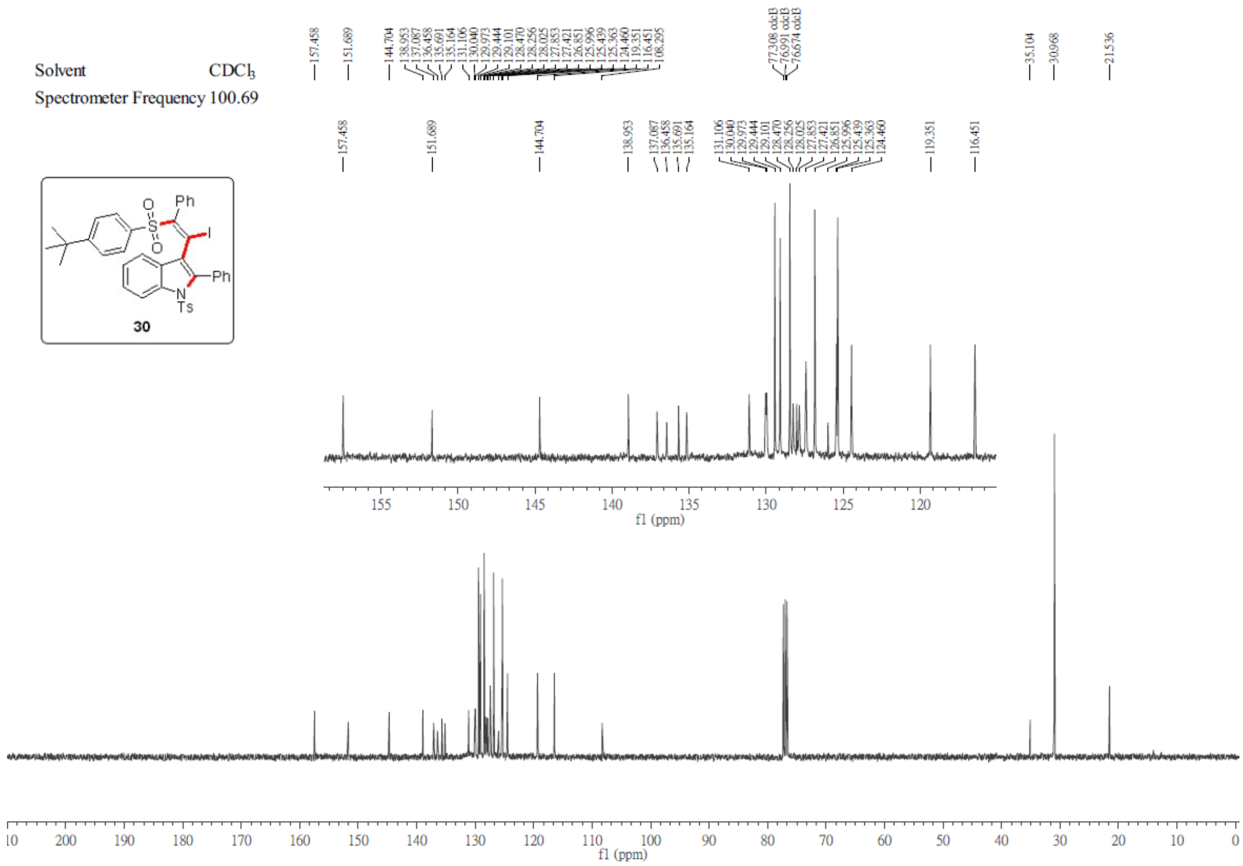

Supplementary Figure 117. ^{1}H (top) and ^{13}C (bottom) NMR spectra of compound **30**.

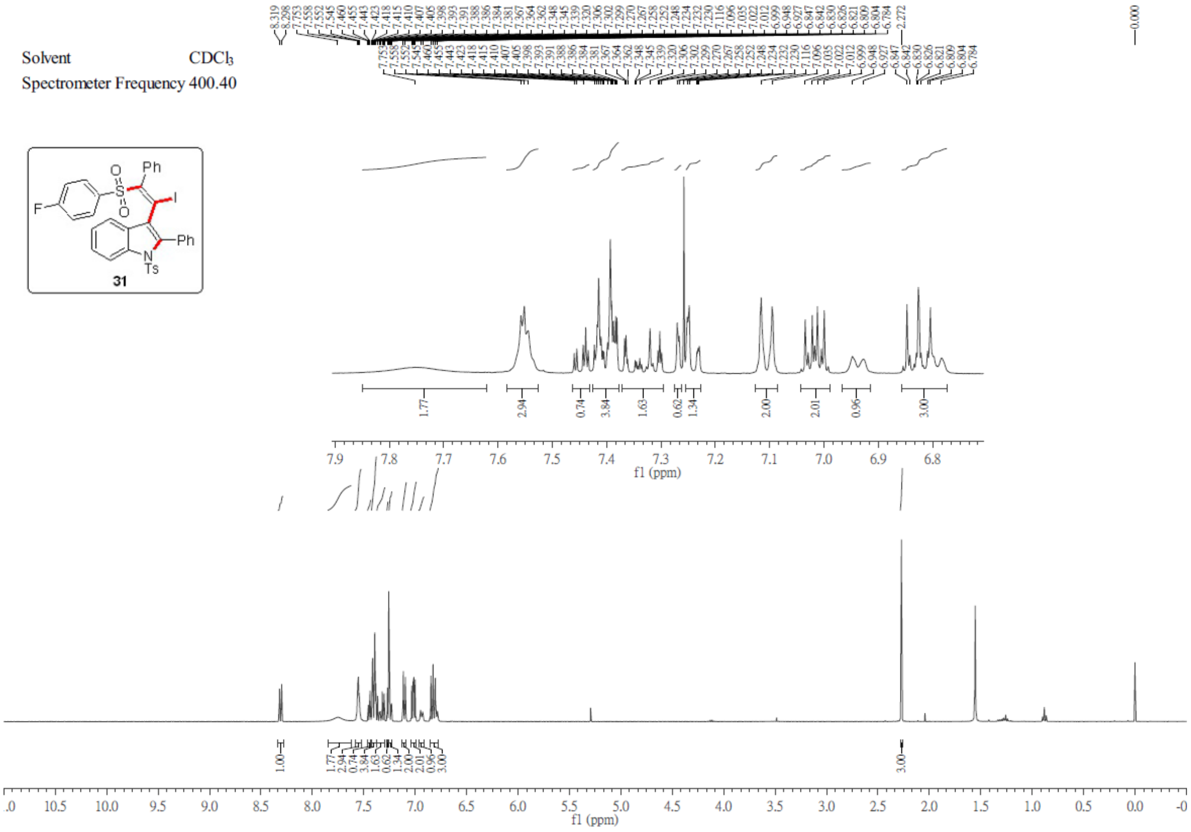

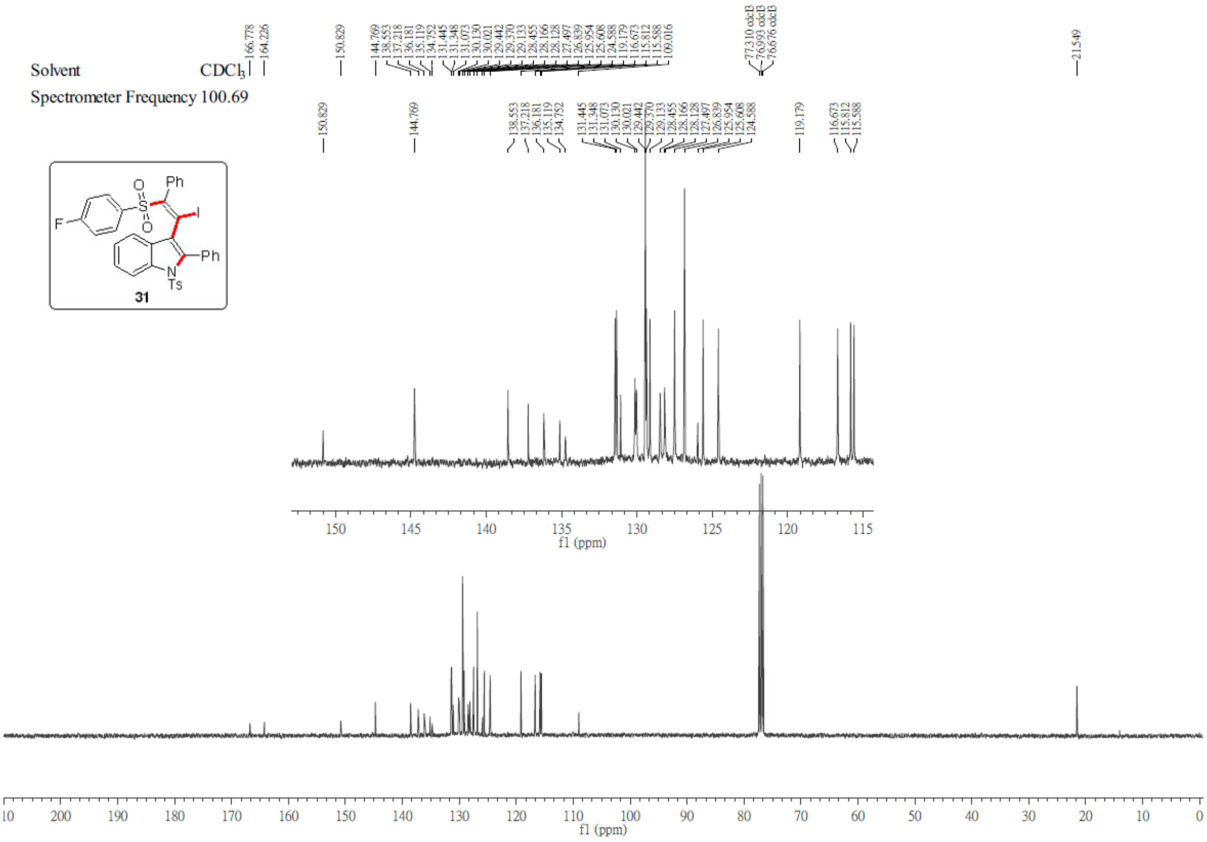

Supplementary Figure 118. ^{1}H (top) and ^{13}C (bottom) NMR spectra of compound **31**.

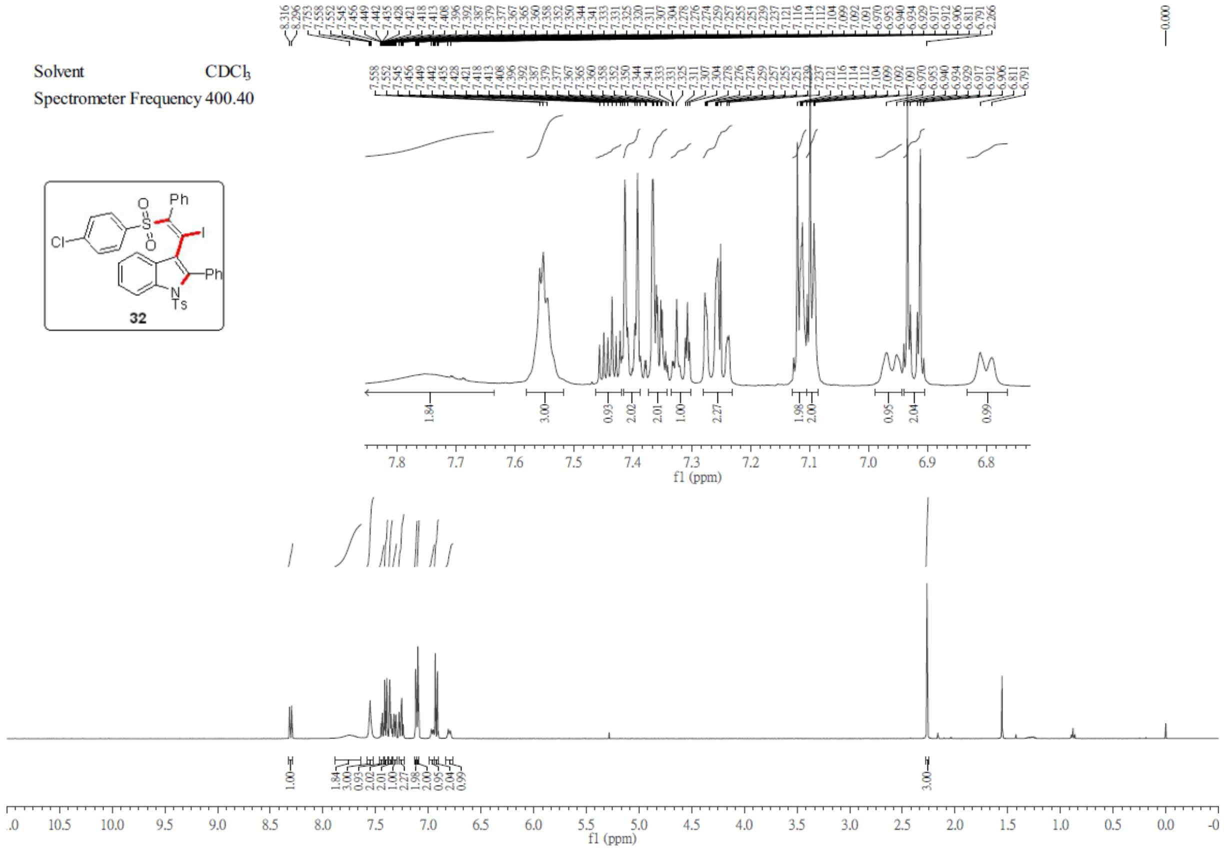

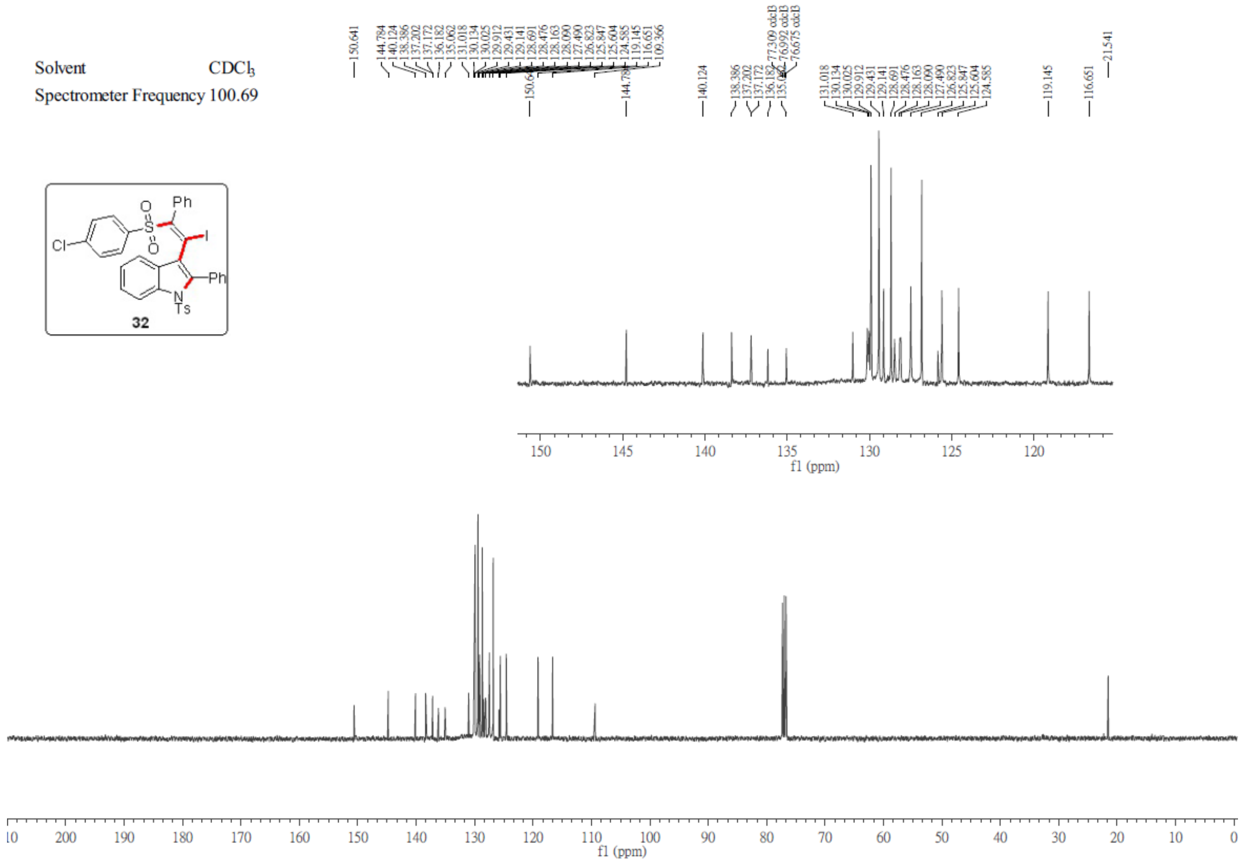

Supplementary Figure 119. ^{1}H (top) and ^{13}C (bottom) NMR spectra of compound **32**.

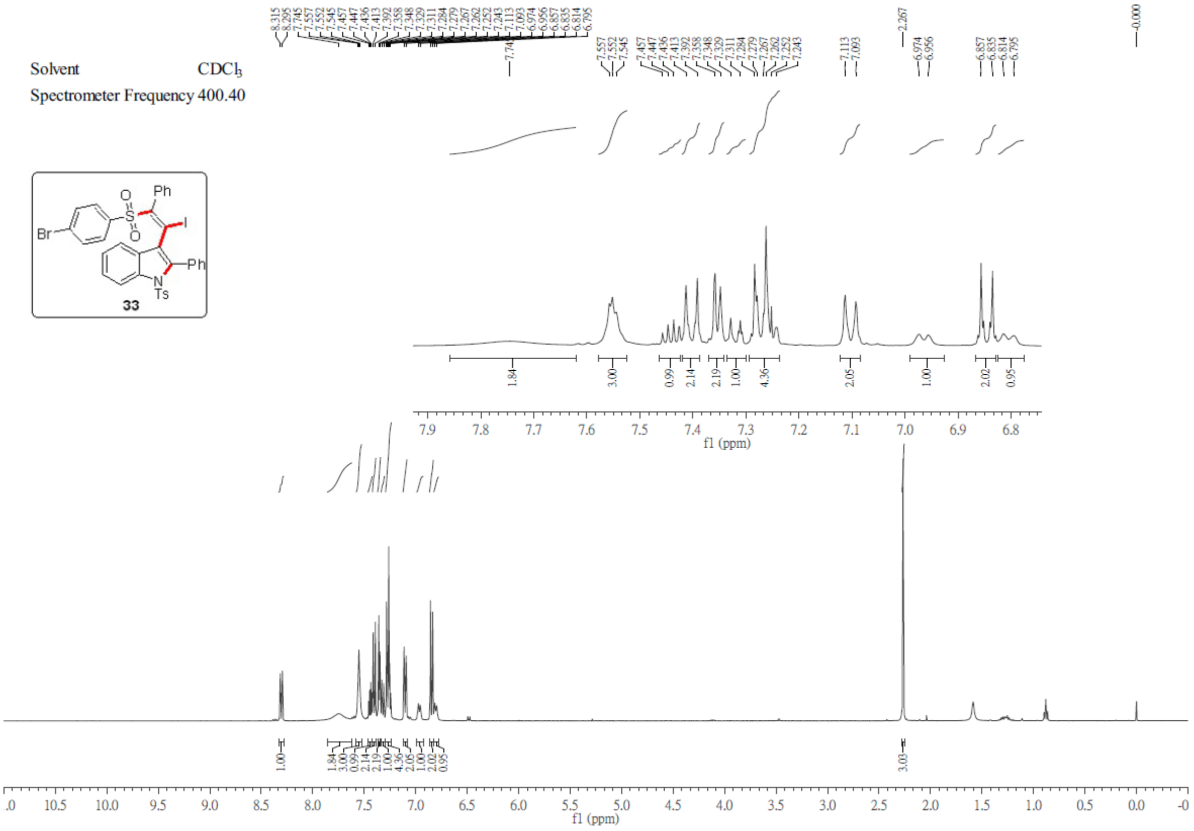

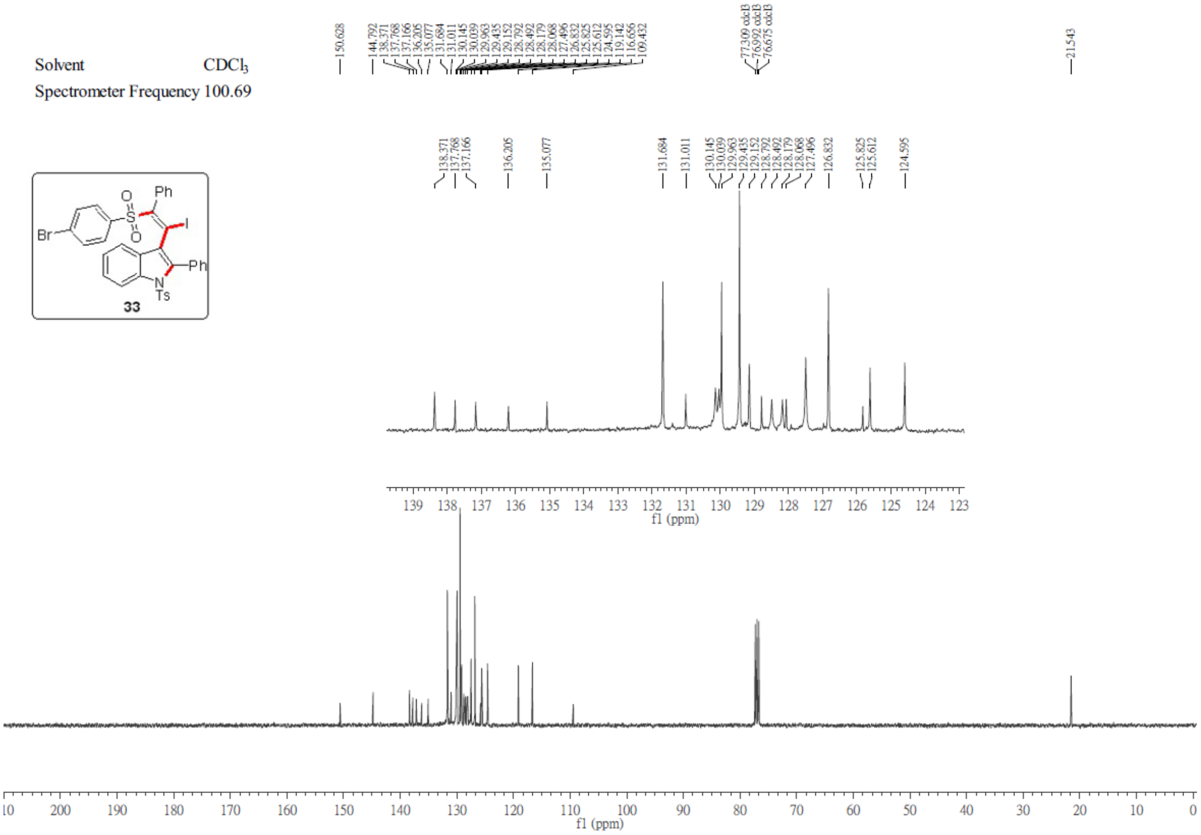

Supplementary Figure 120. ^{1}H (top) and ^{13}C (bottom) NMR spectra of compound **33**.

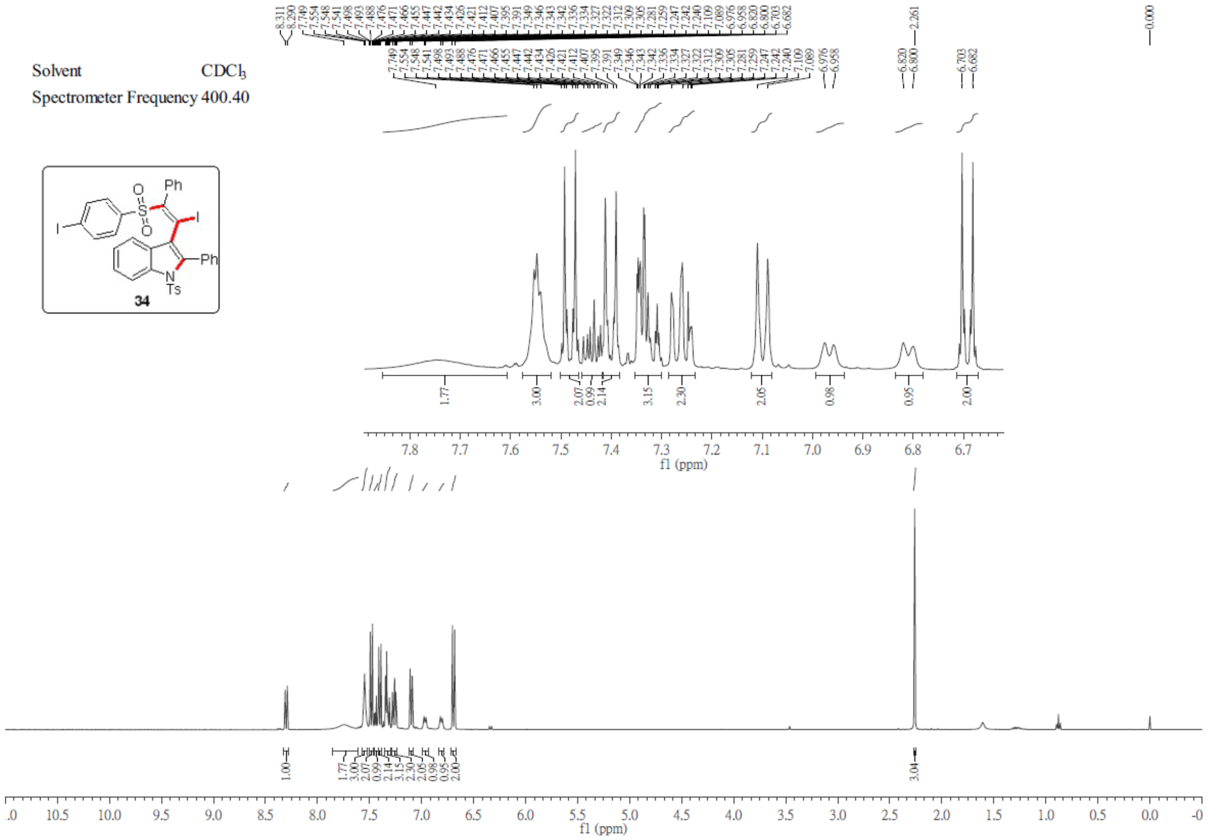

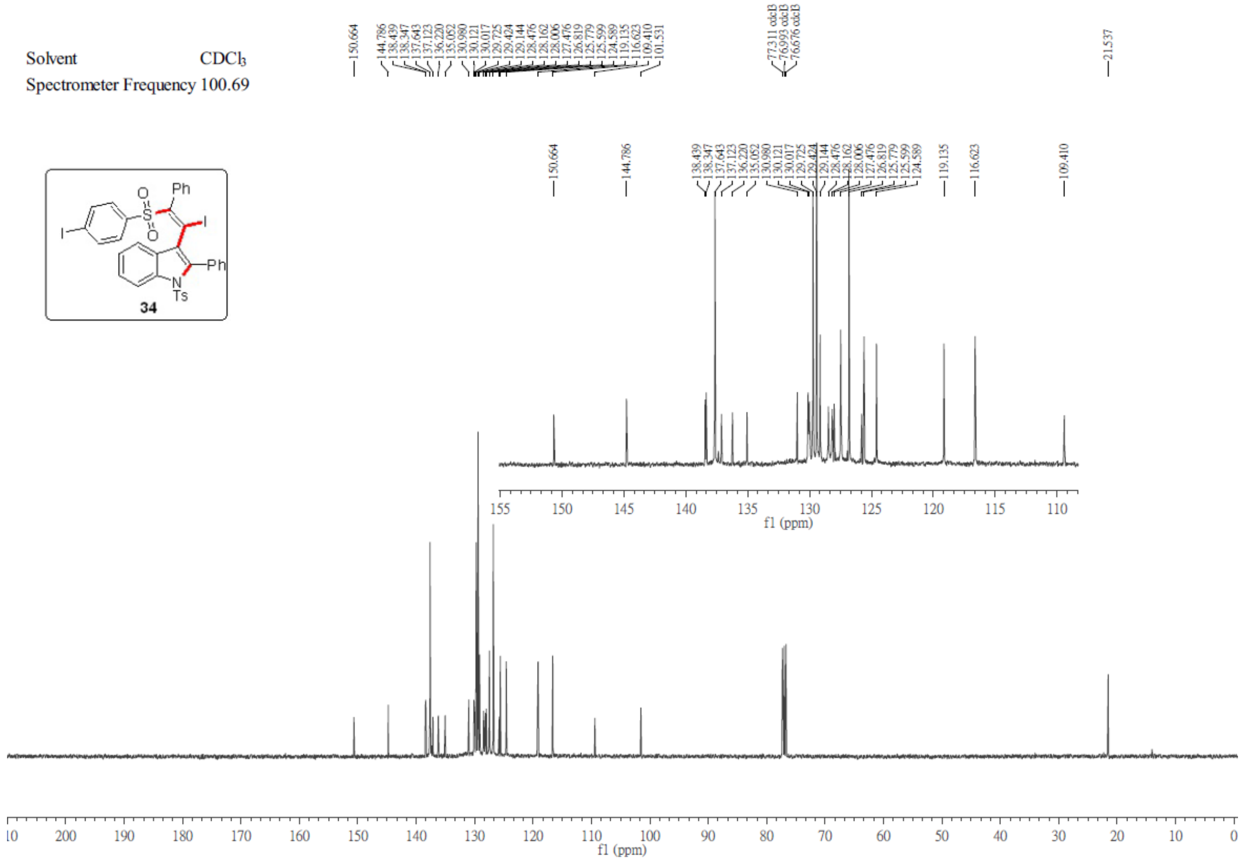

Supplementary Figure 121. ^{1}H (top) and ^{13}C (bottom) NMR spectra of compound **34**.

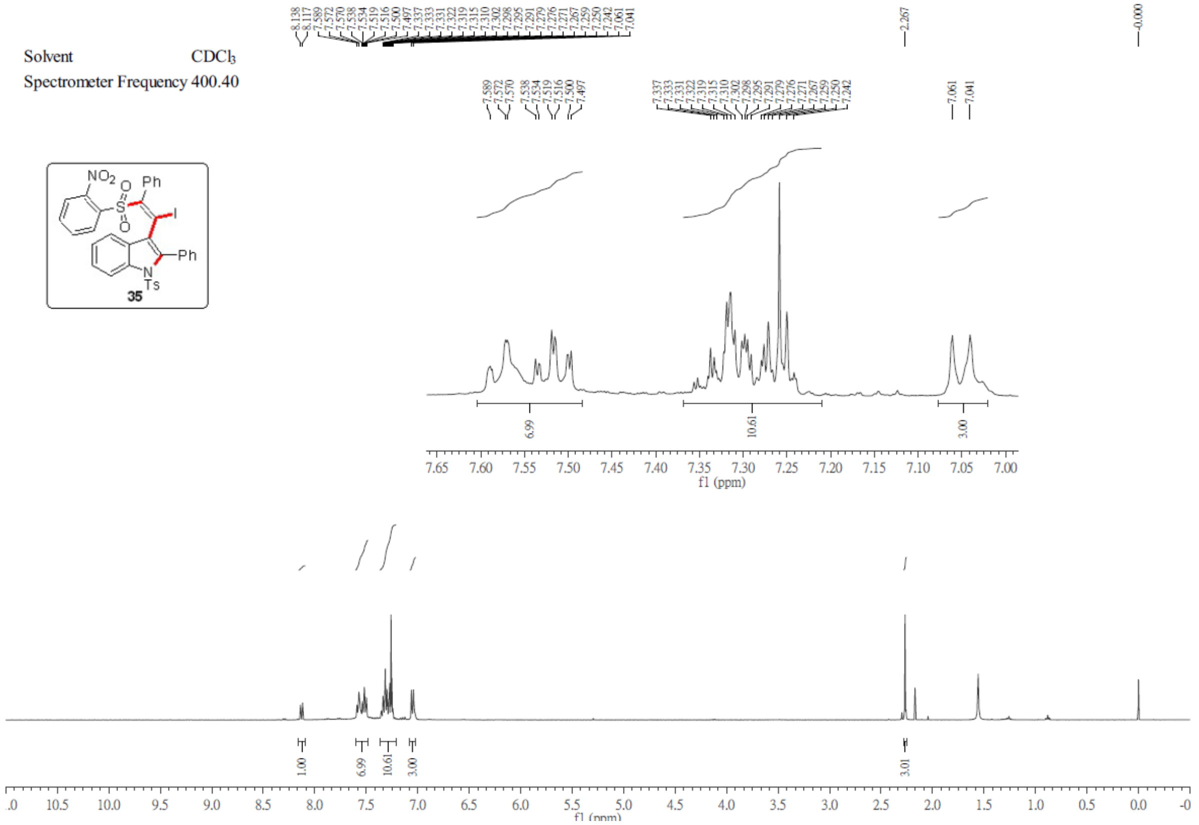

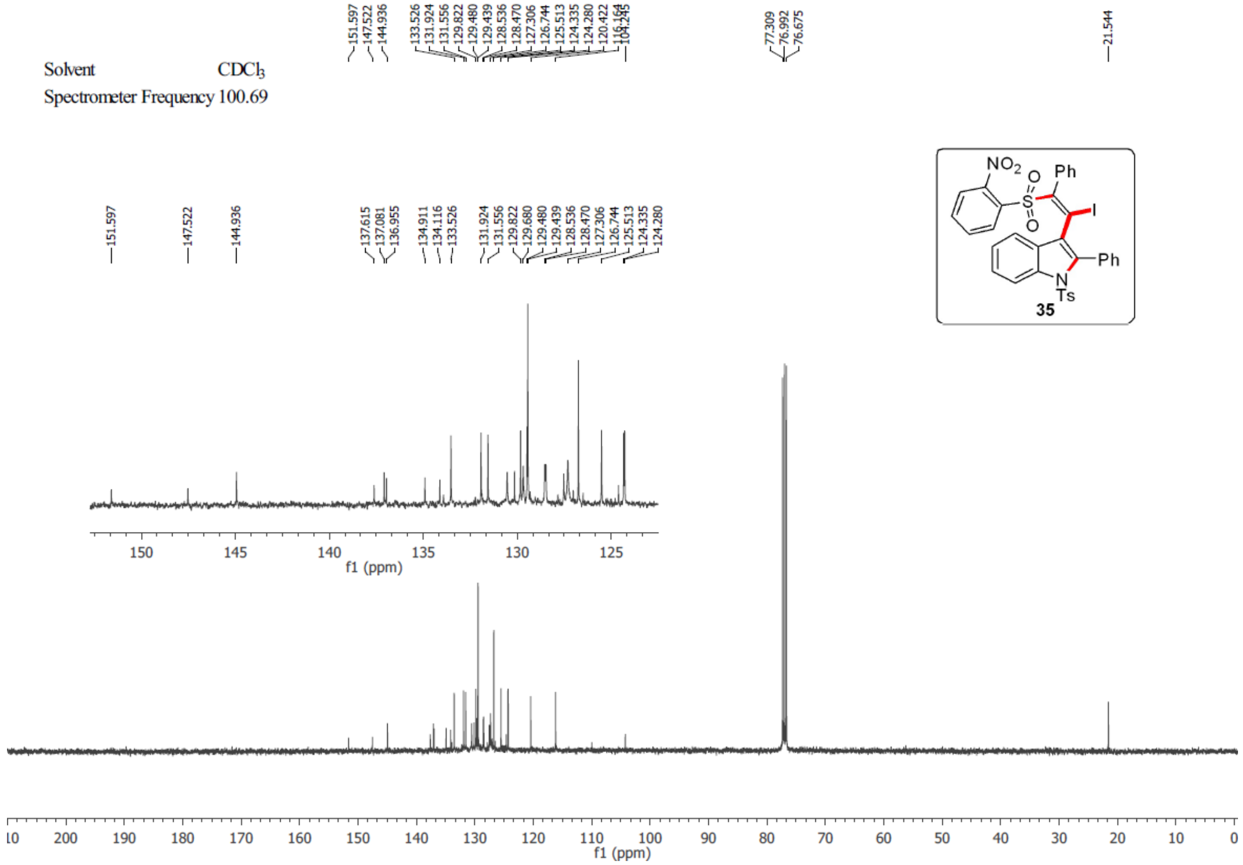

Supplementary Figure 122. ^{1}H (top) and ^{13}C (bottom) NMR spectra of compound **35**.

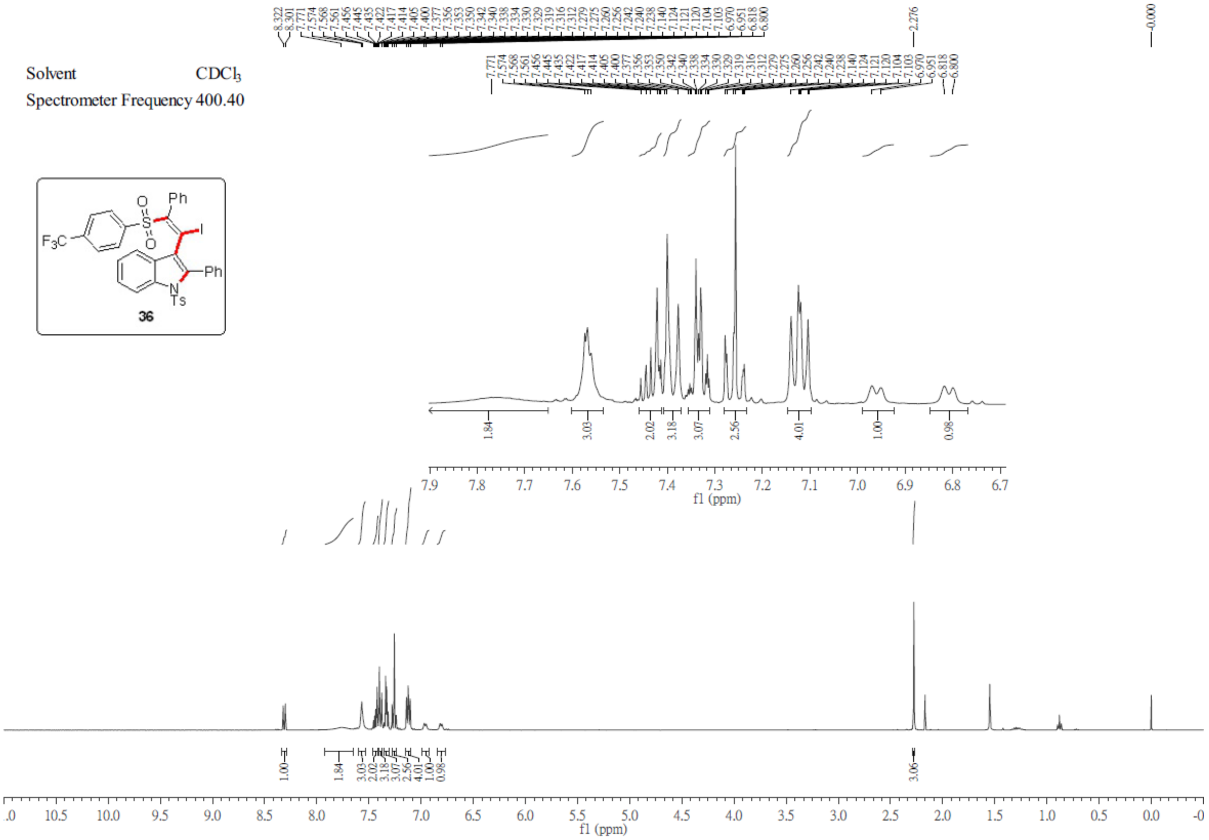

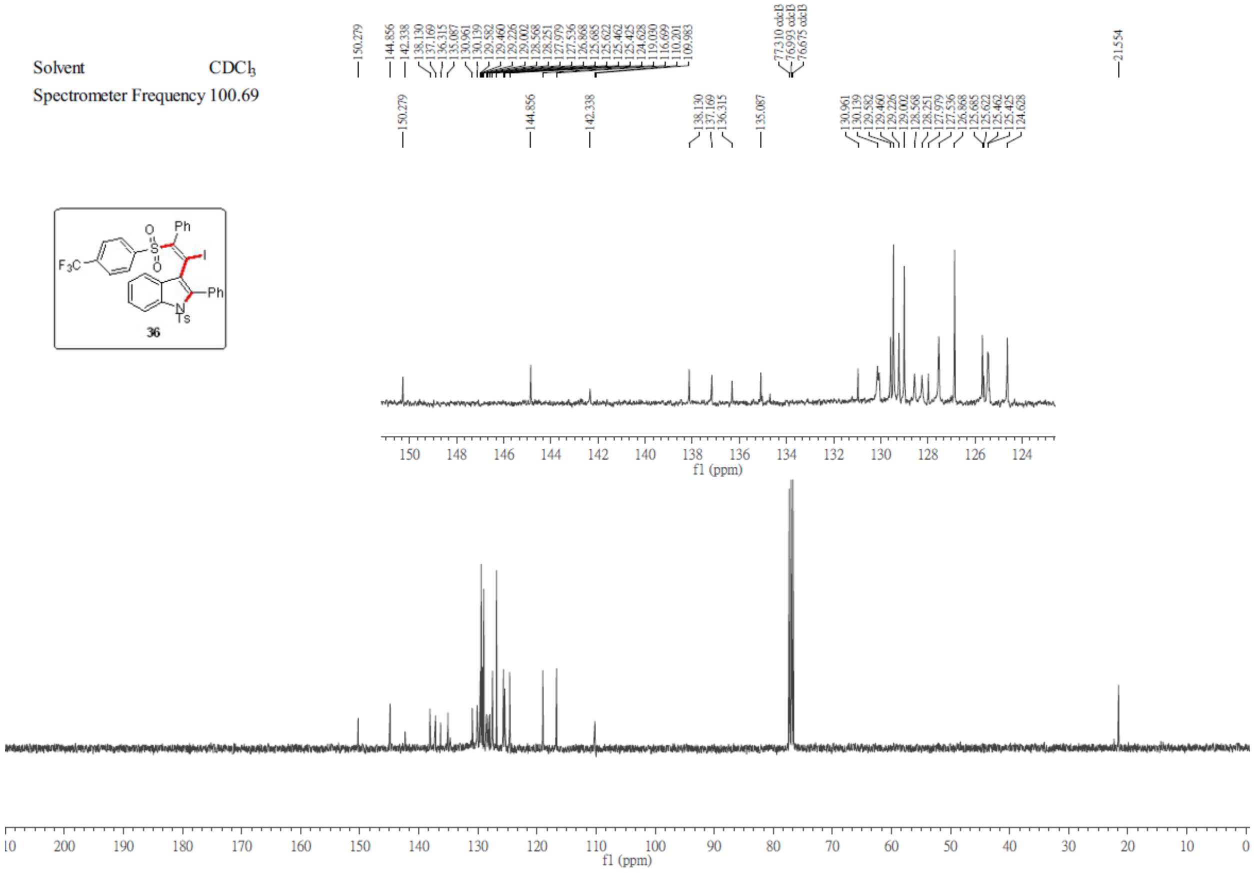

Supplementary Figure 123. ^{1}H (top) and ^{13}C (bottom) NMR spectra of compound **36**.

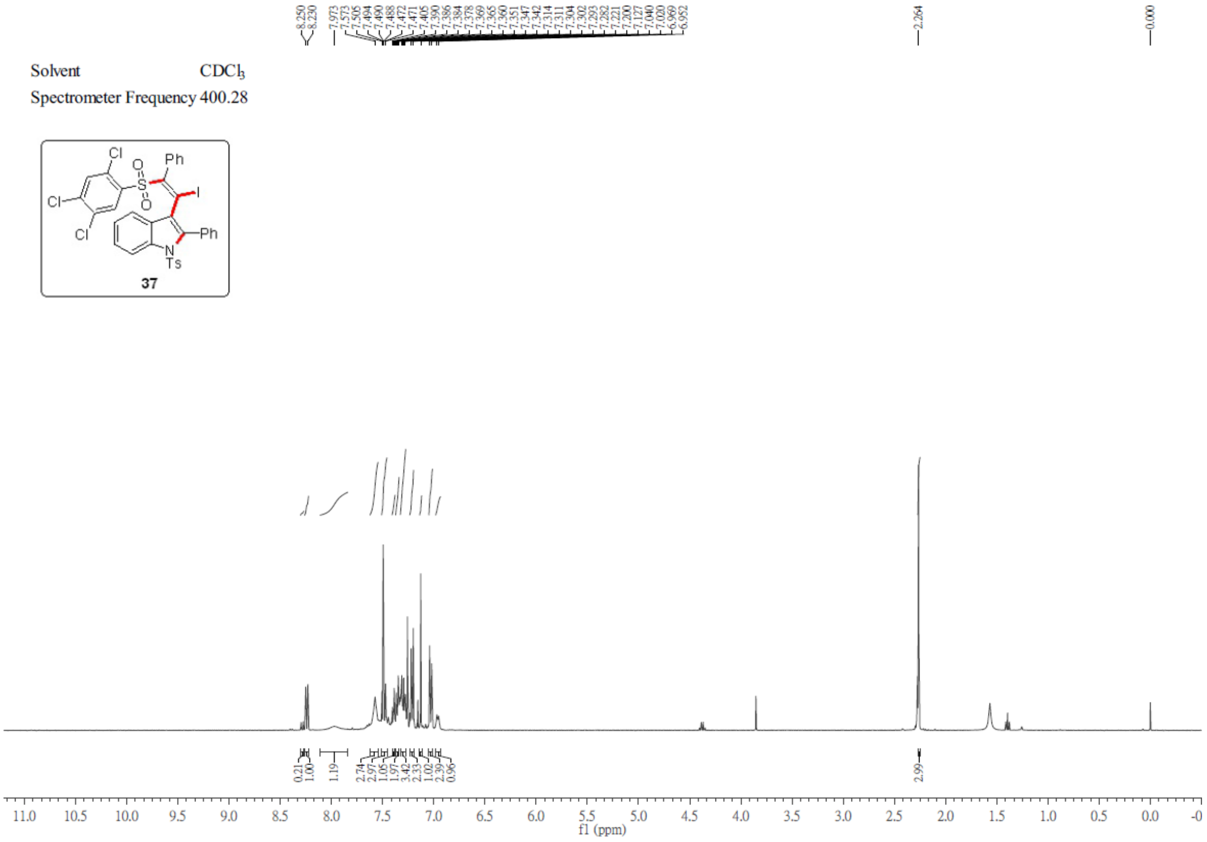

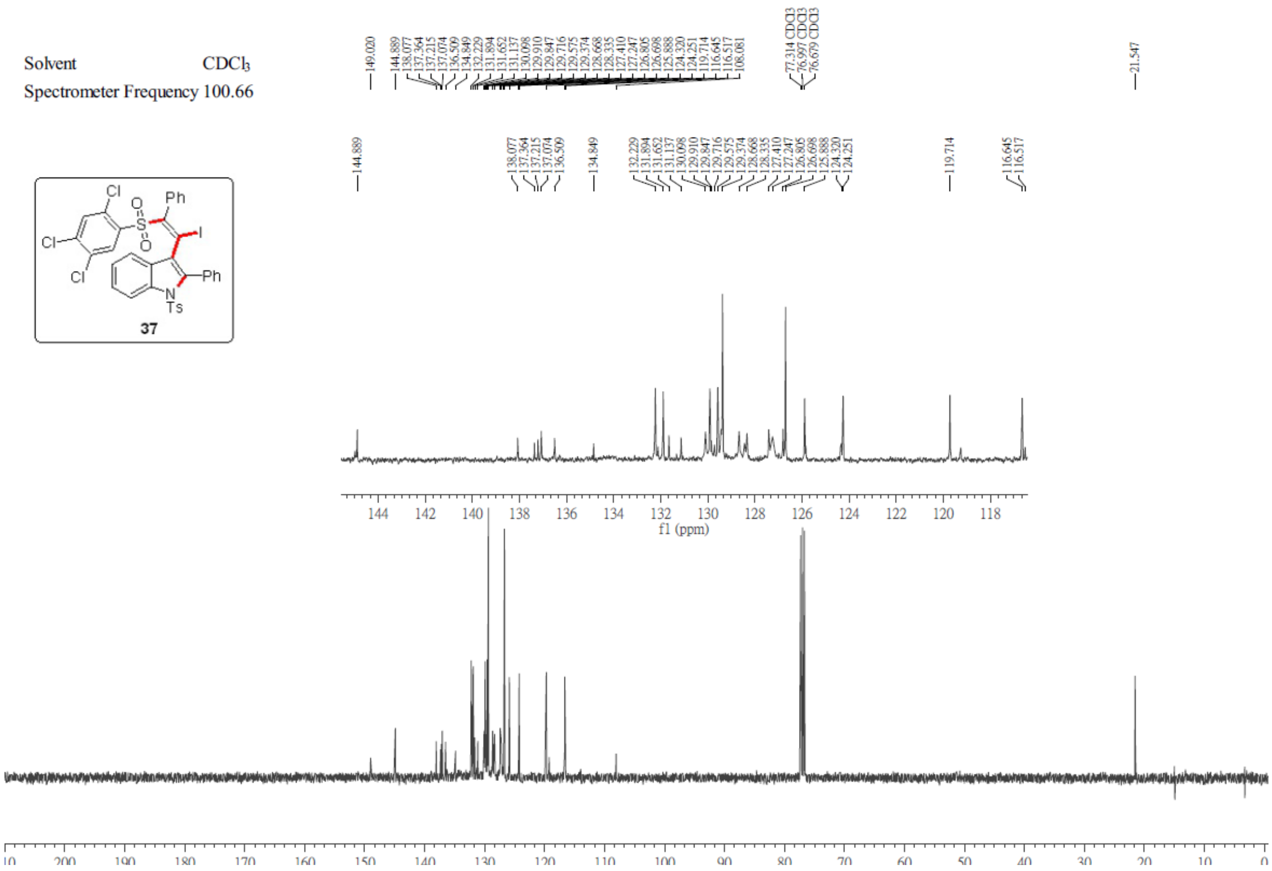

Supplementary Figure 124. ^{1}H (top) and ^{13}C (bottom) NMR spectra of compound **37**.

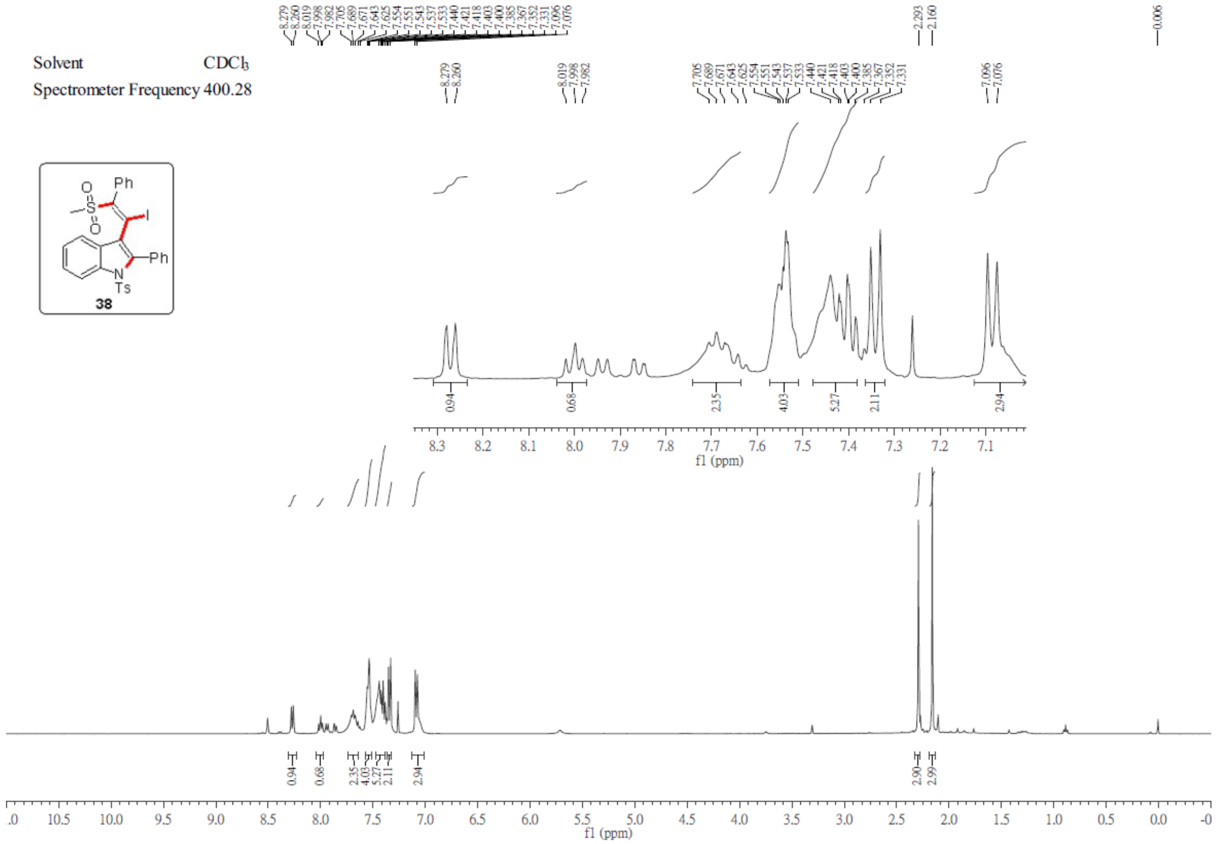

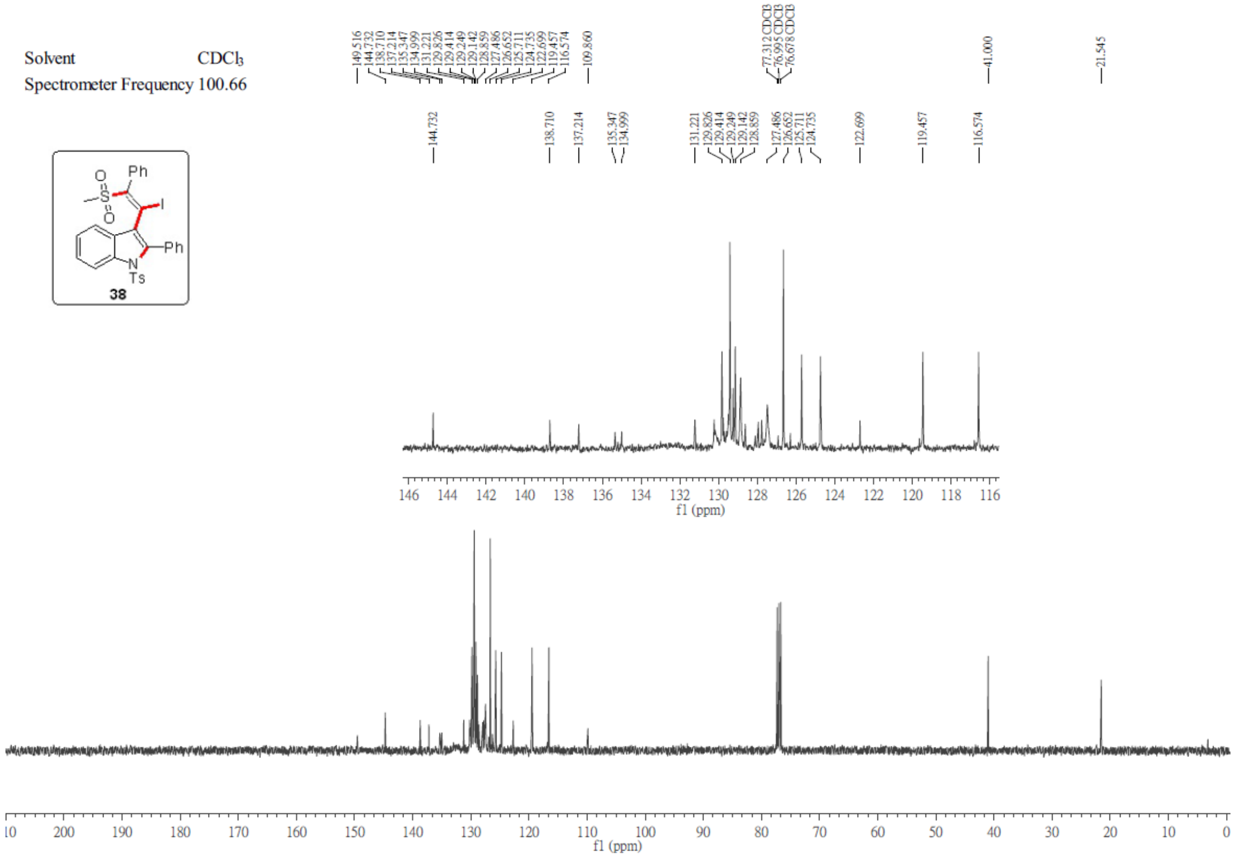

Supplementary Figure 125. ^{1}H (top) and ^{13}C (bottom) NMR spectra of compound **38**.

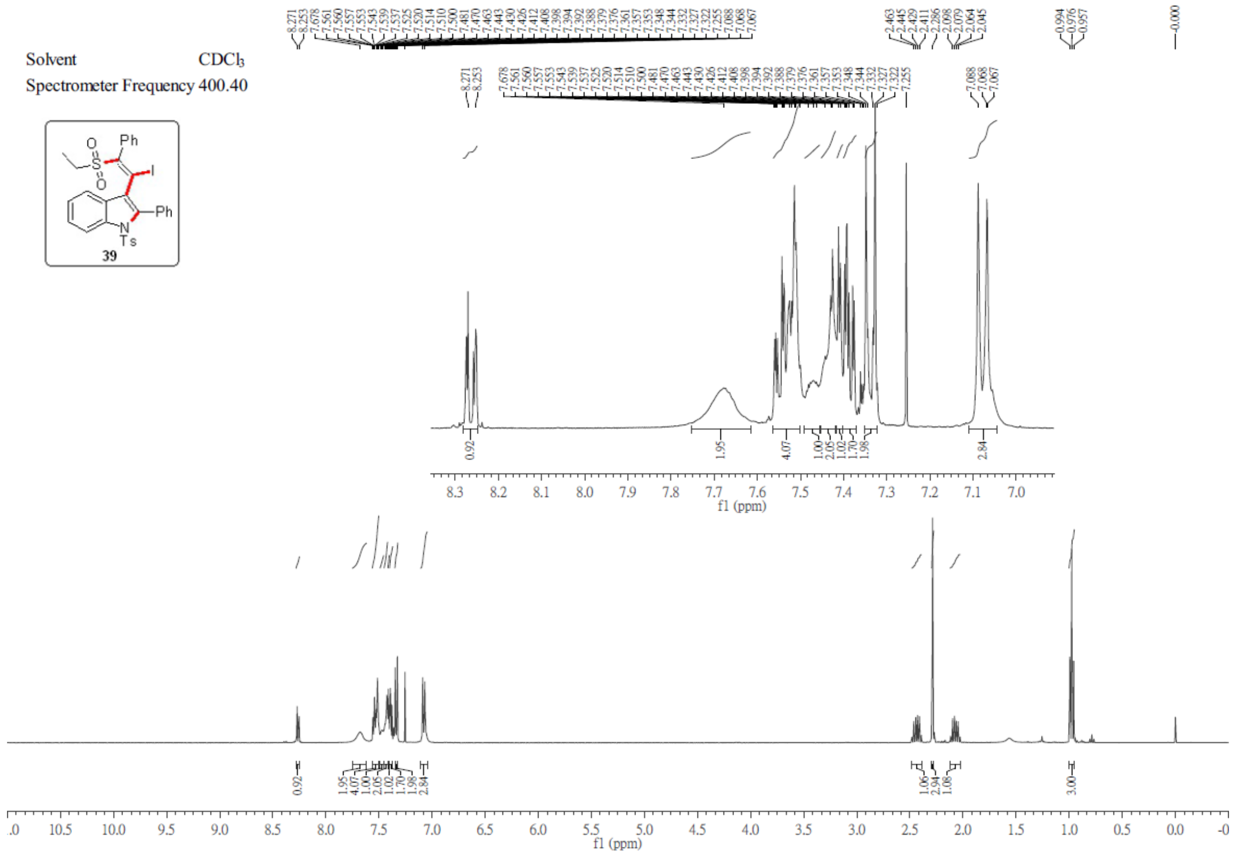

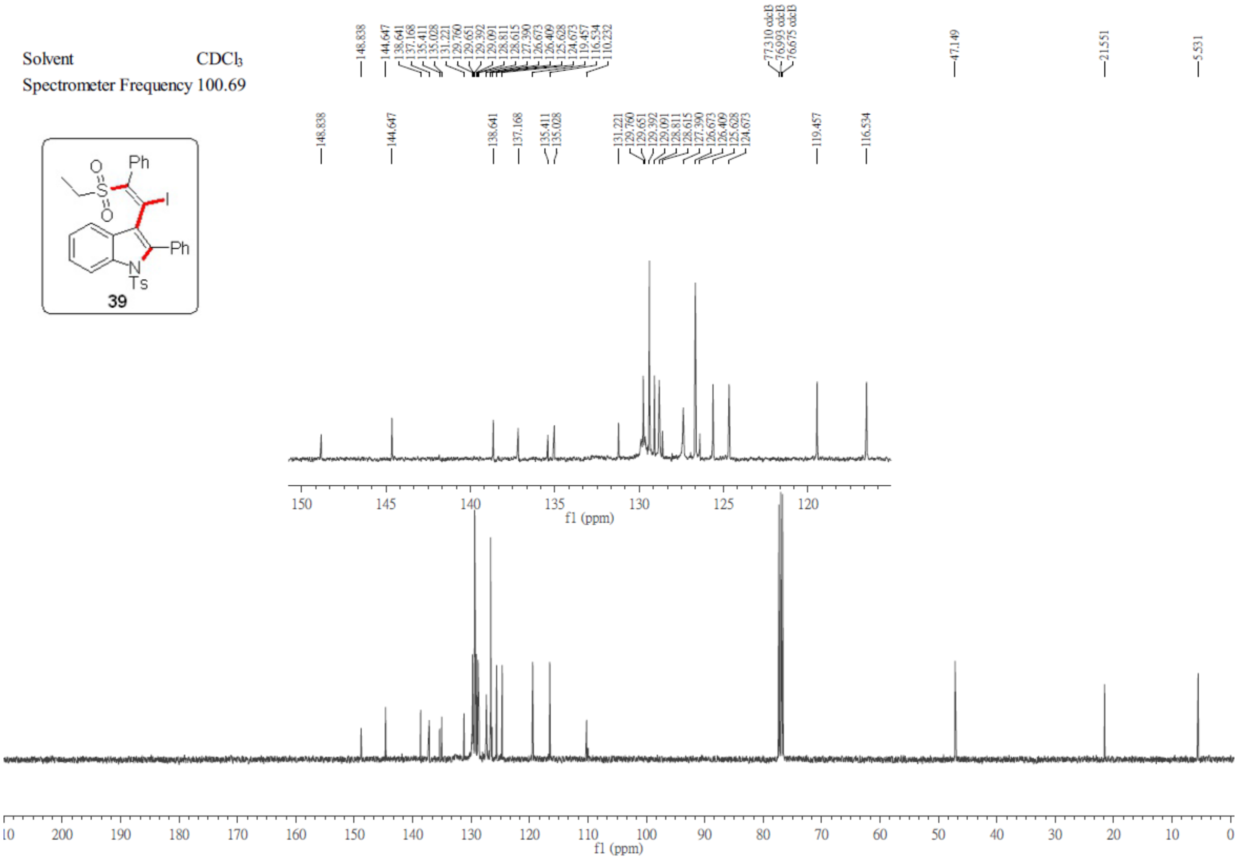

Supplementary Figure 126. ^{1}H (top) and ^{13}C (bottom) NMR spectra of compound **39**.

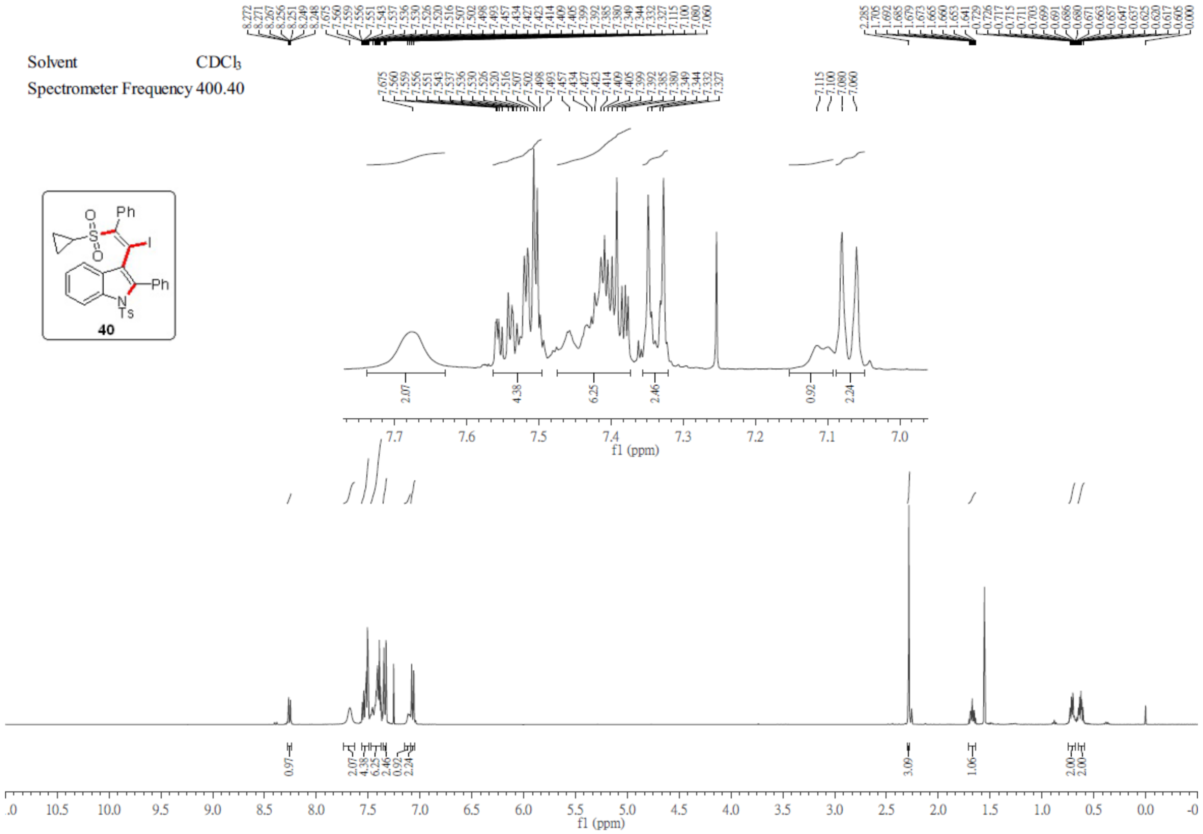

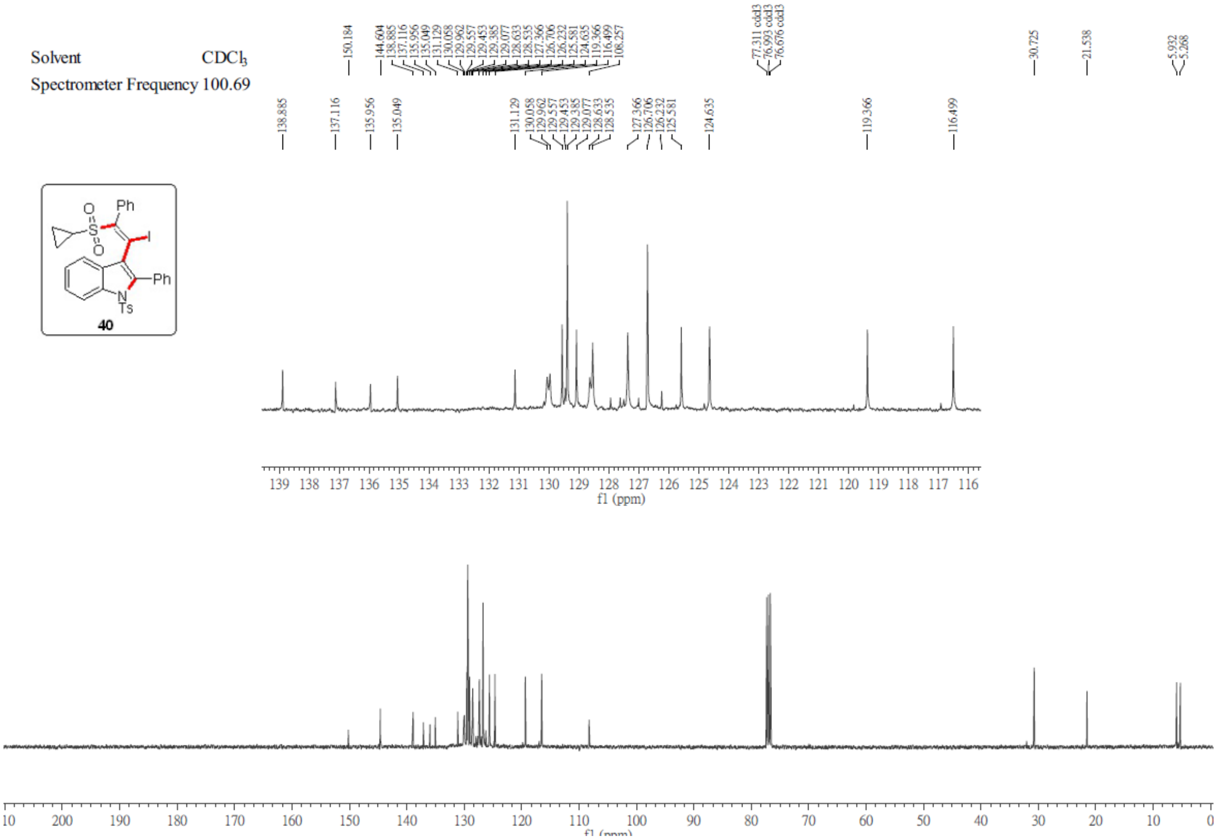

Supplementary Figure 127. ^{1}H (top) and ^{13}C (bottom) NMR spectra of compound **40**.

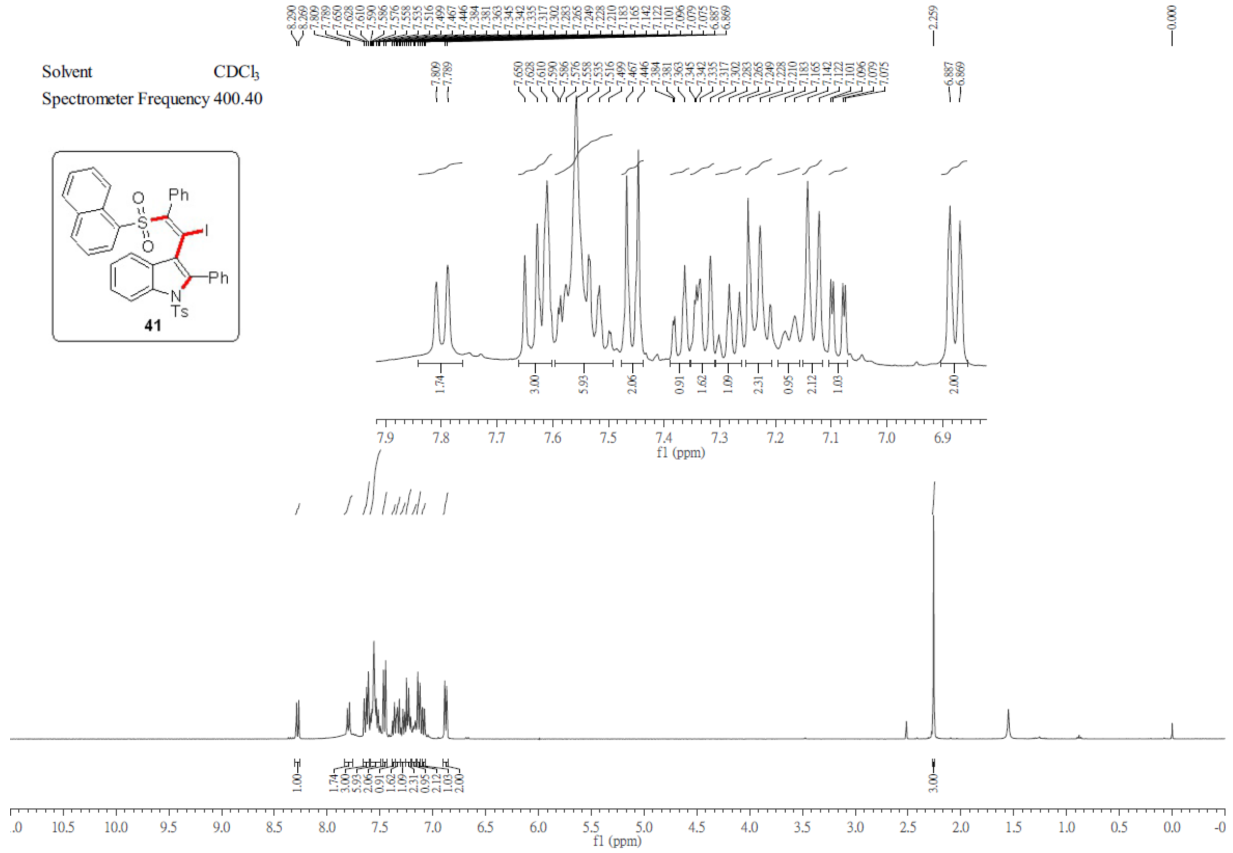

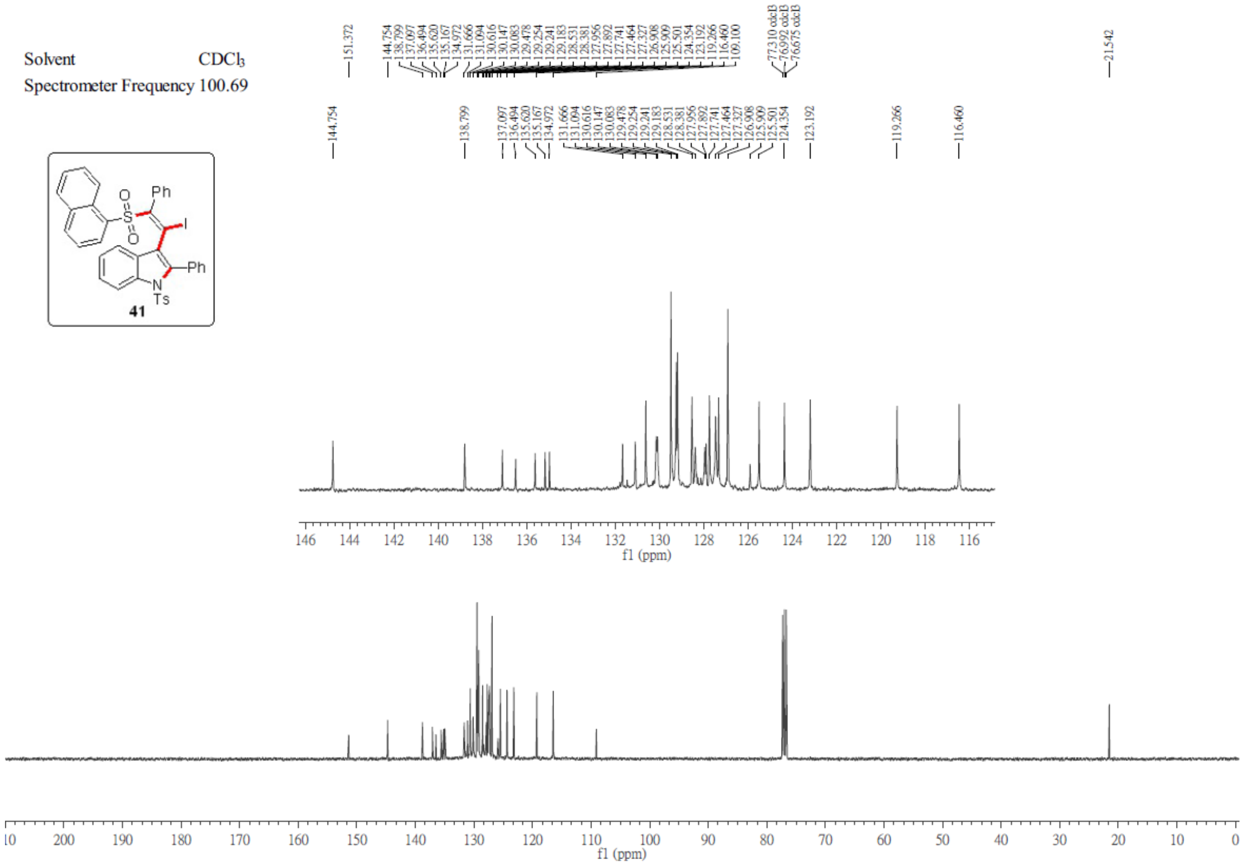

Supplementary Figure 128. ^{1}H (top) and ^{13}C (bottom) NMR spectra of compound **41**.

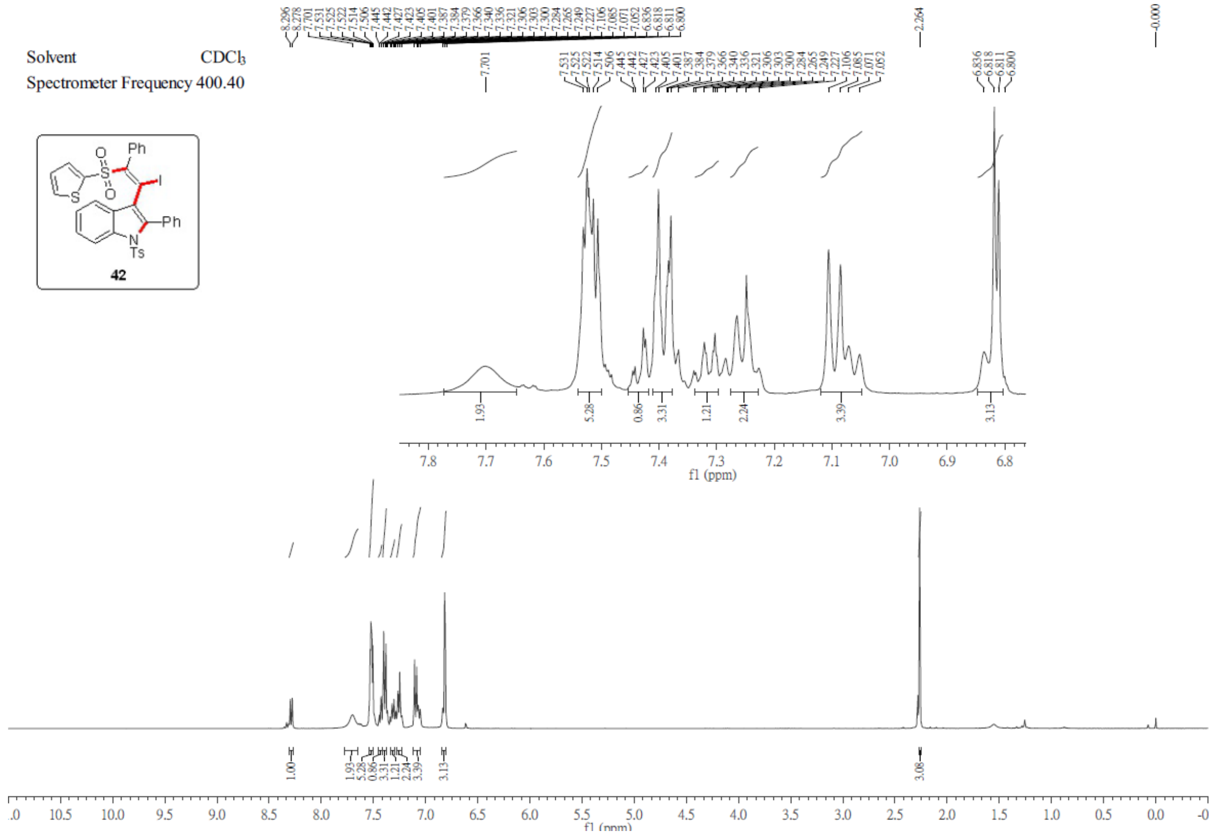

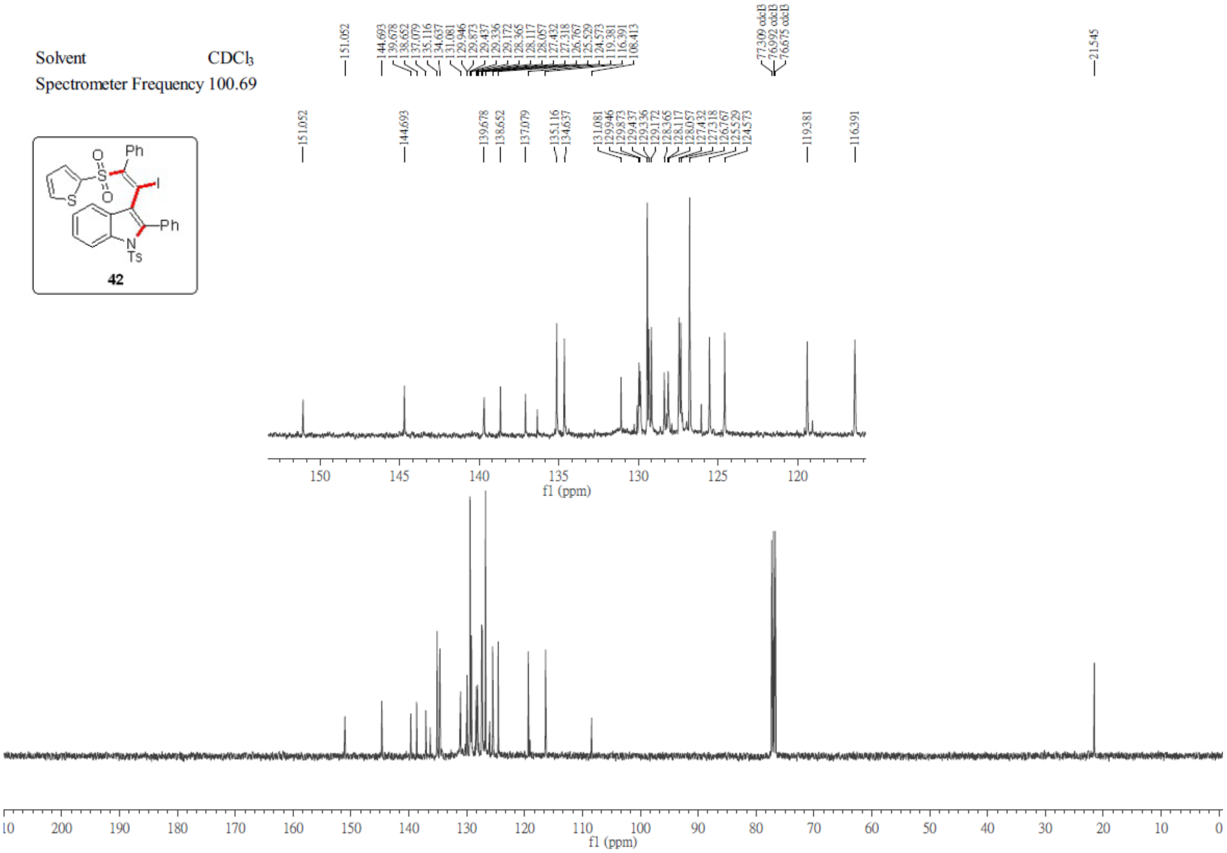

Supplementary Figure 129. ^{1}H (top) and ^{13}C (bottom) NMR spectra of compound **42**.

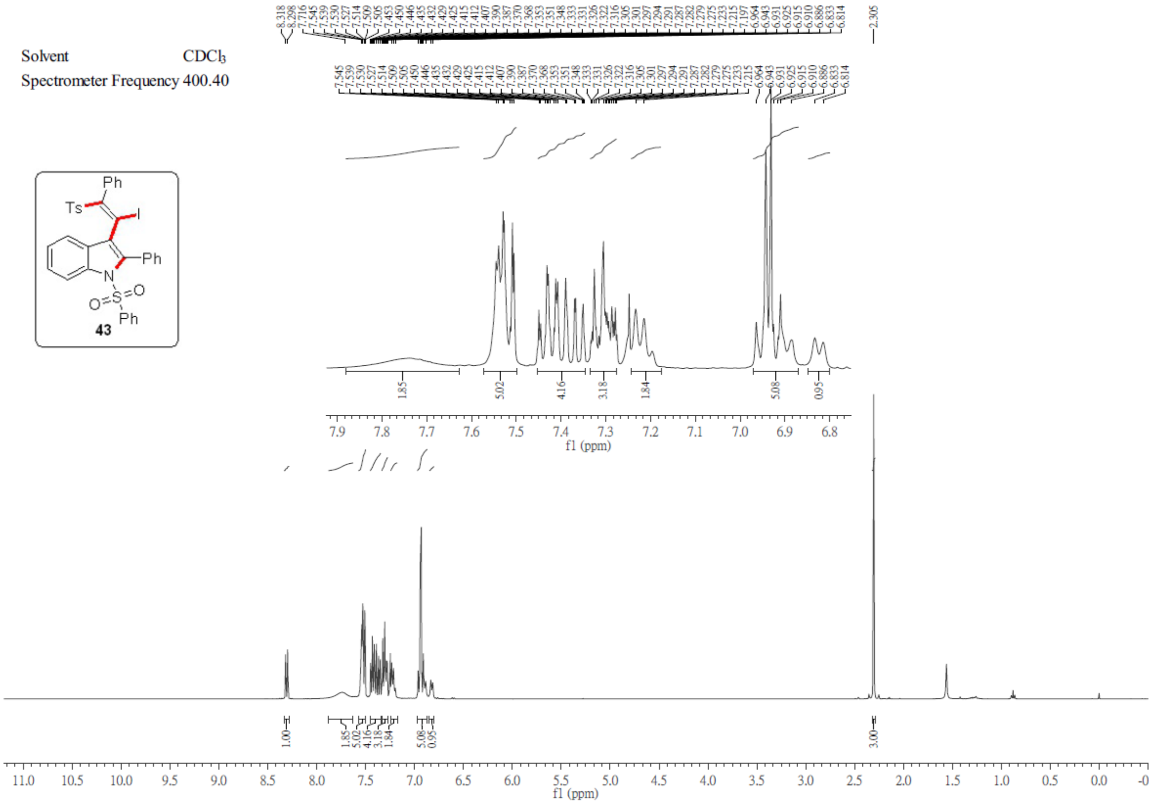

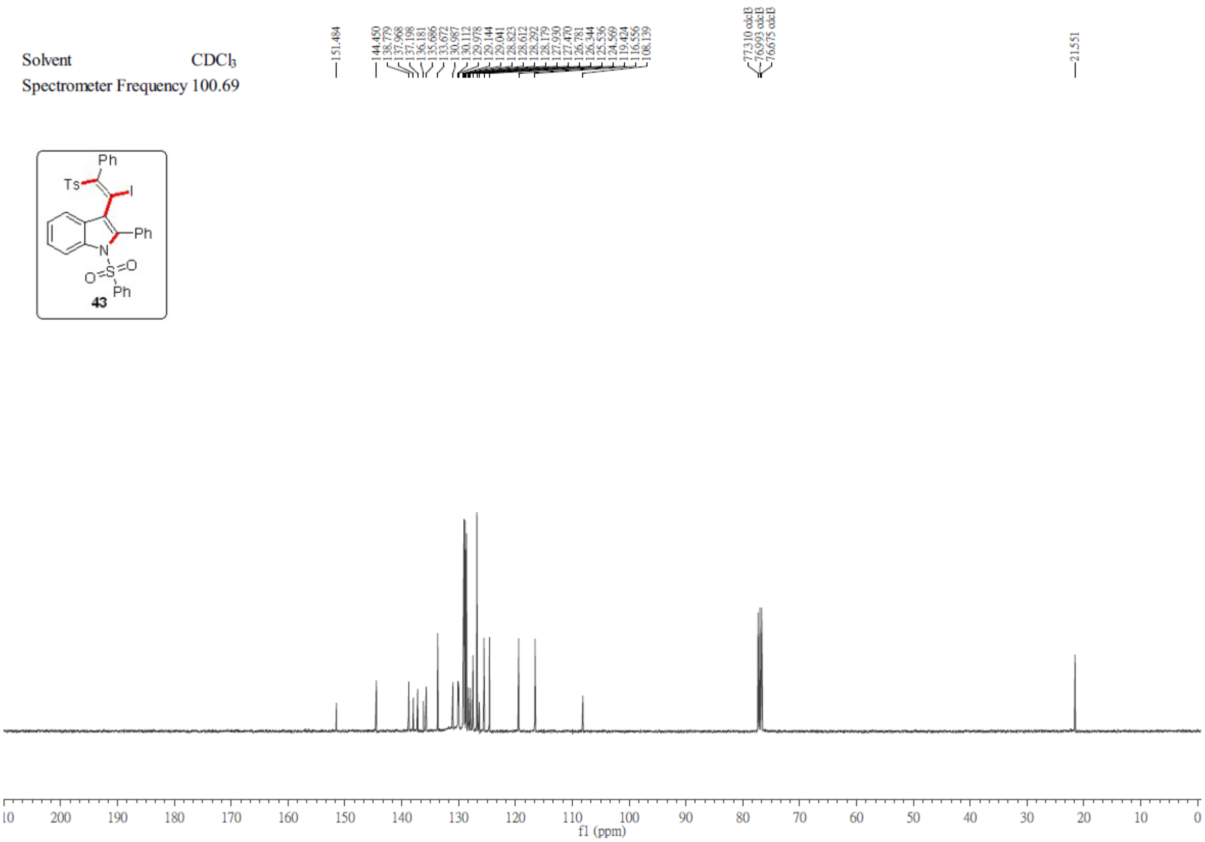

Supplementary Figure 130. ^{1}H (top) and ^{13}C (bottom) NMR spectra of compound **43**.

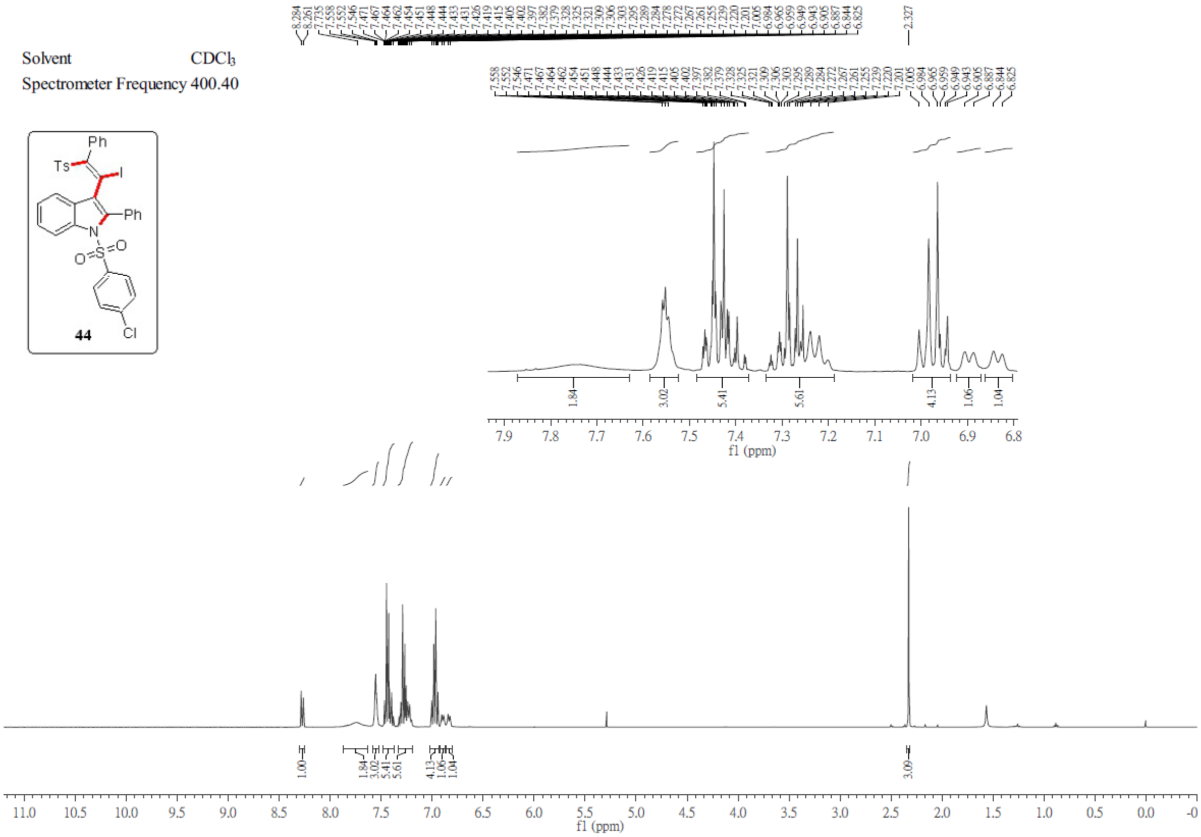

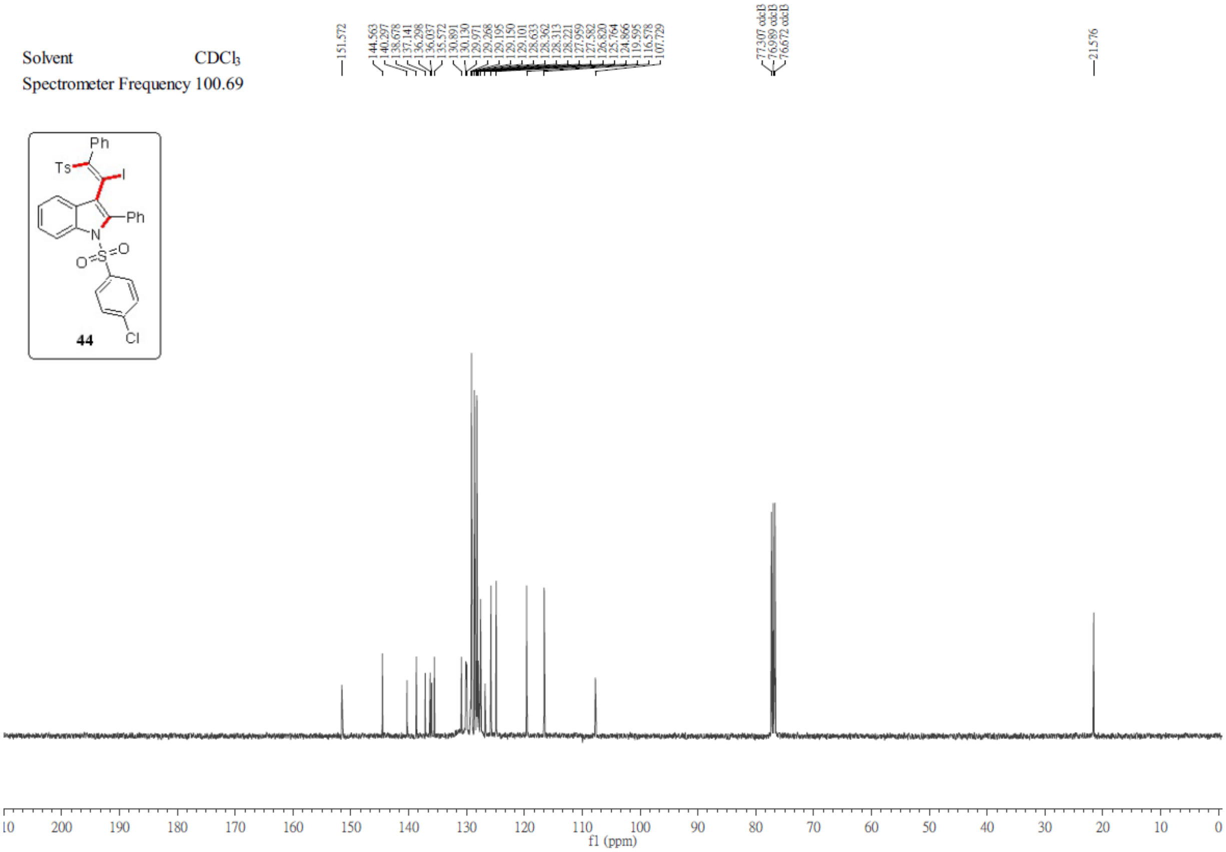

Supplementary Figure 131. ^{1}H (top) and ^{13}C (bottom) NMR spectra of compound **44**.

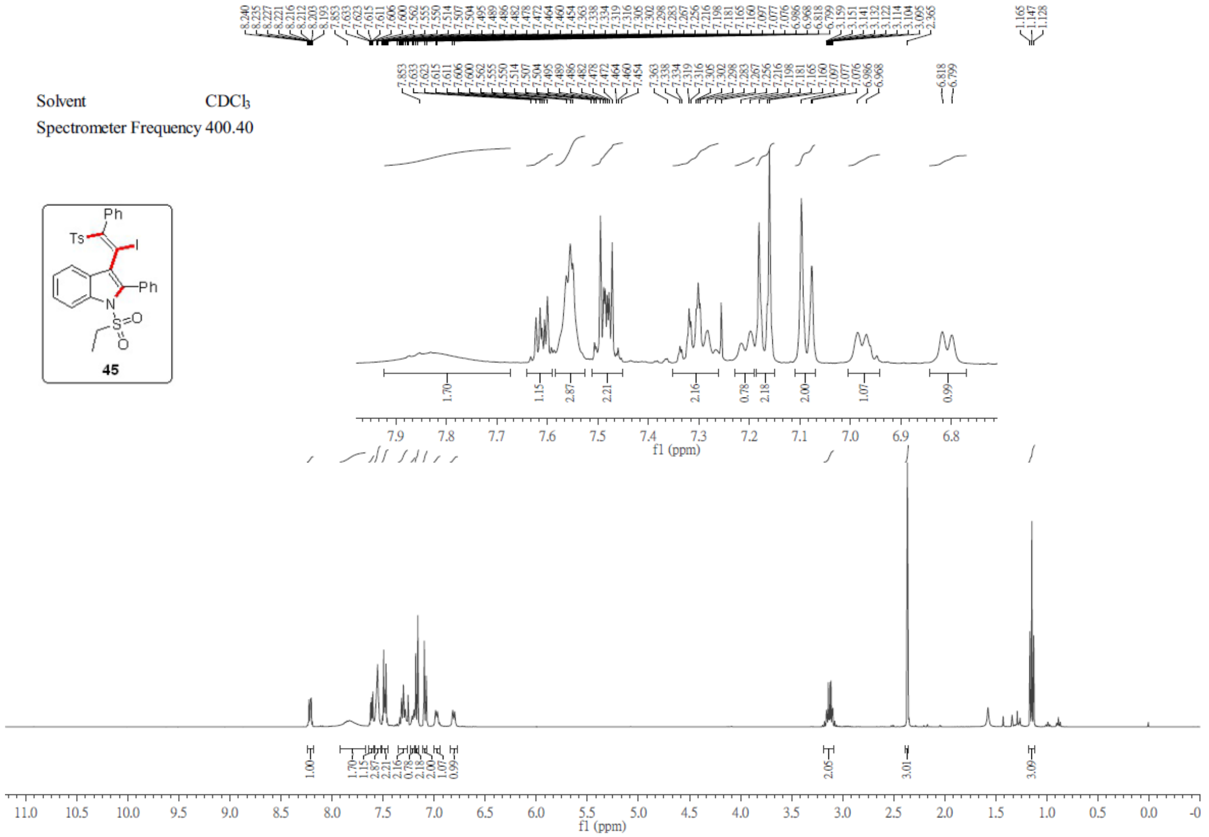

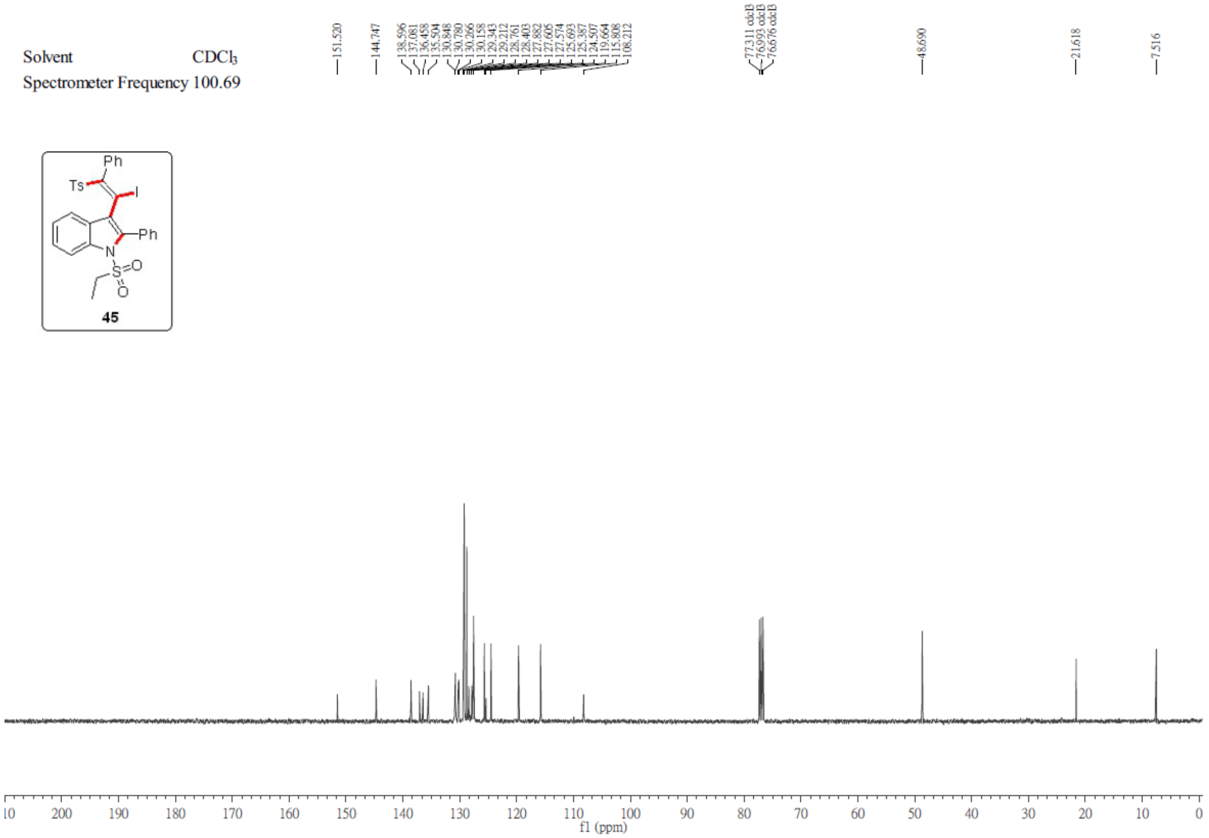

Supplementary Figure 132. ^{1}H (top) and ^{13}C (bottom) NMR spectra of compound **45**.

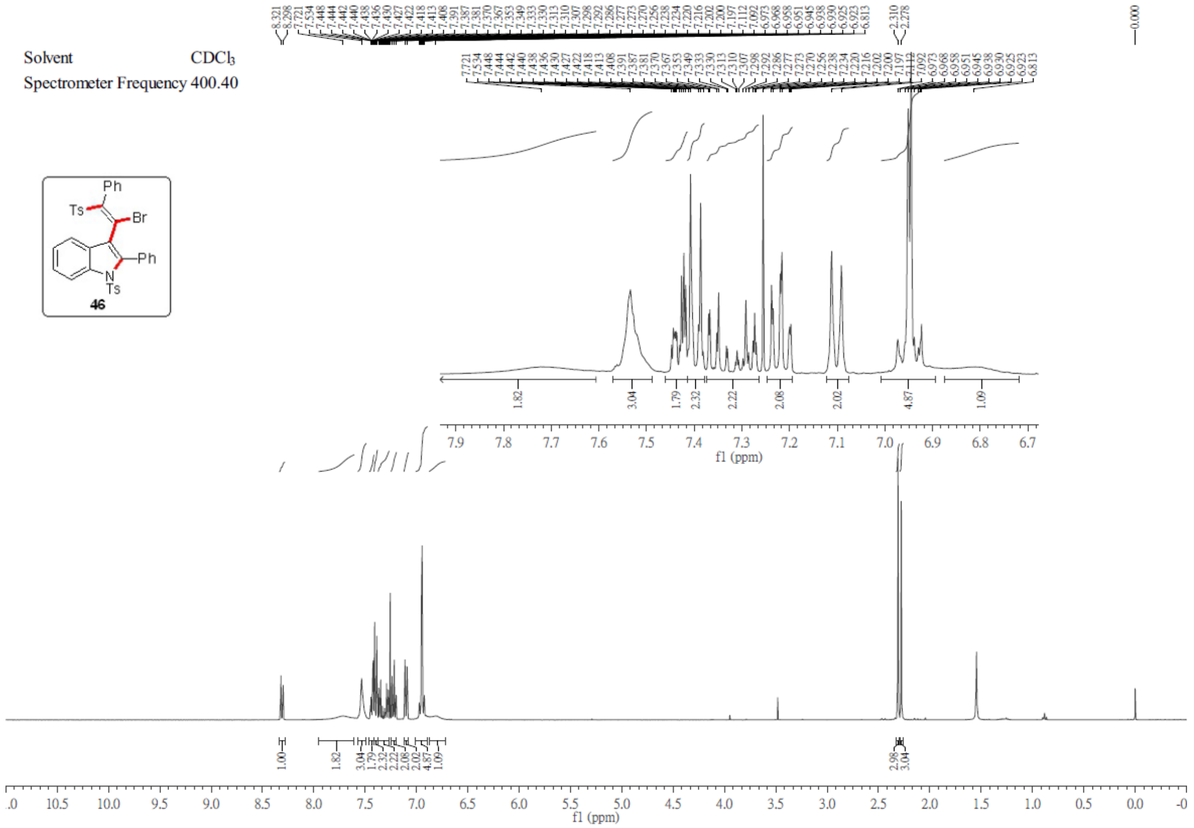

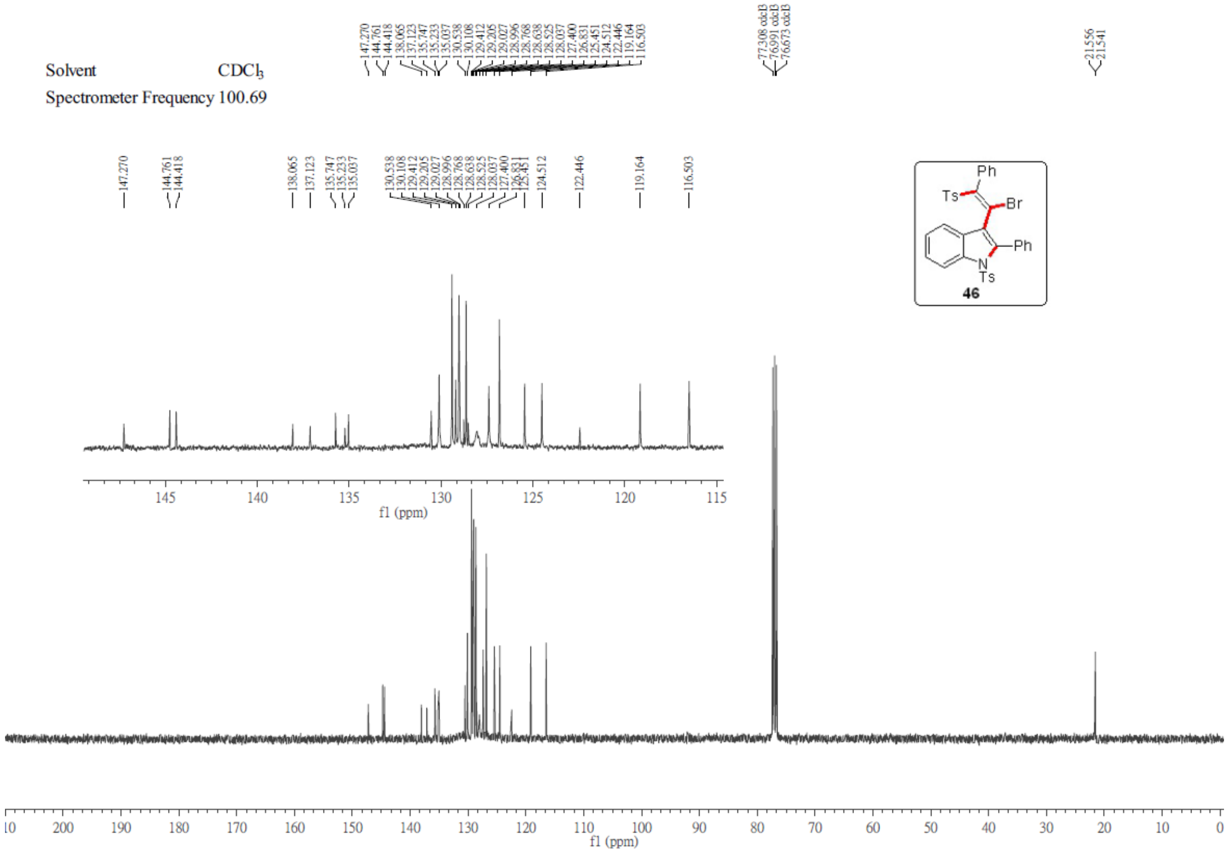

Supplementary Figure 133. ^{1}H (top) and ^{13}C (bottom) NMR spectra of compound **46**.

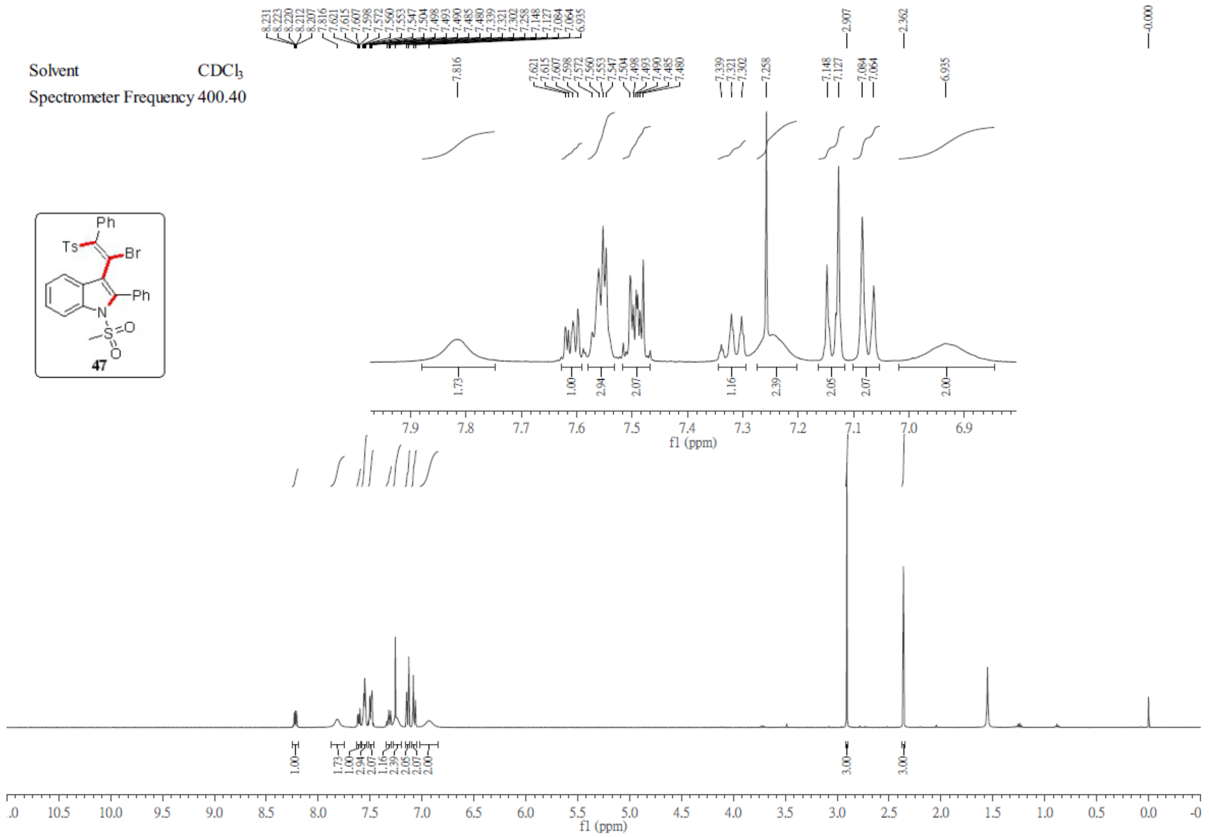

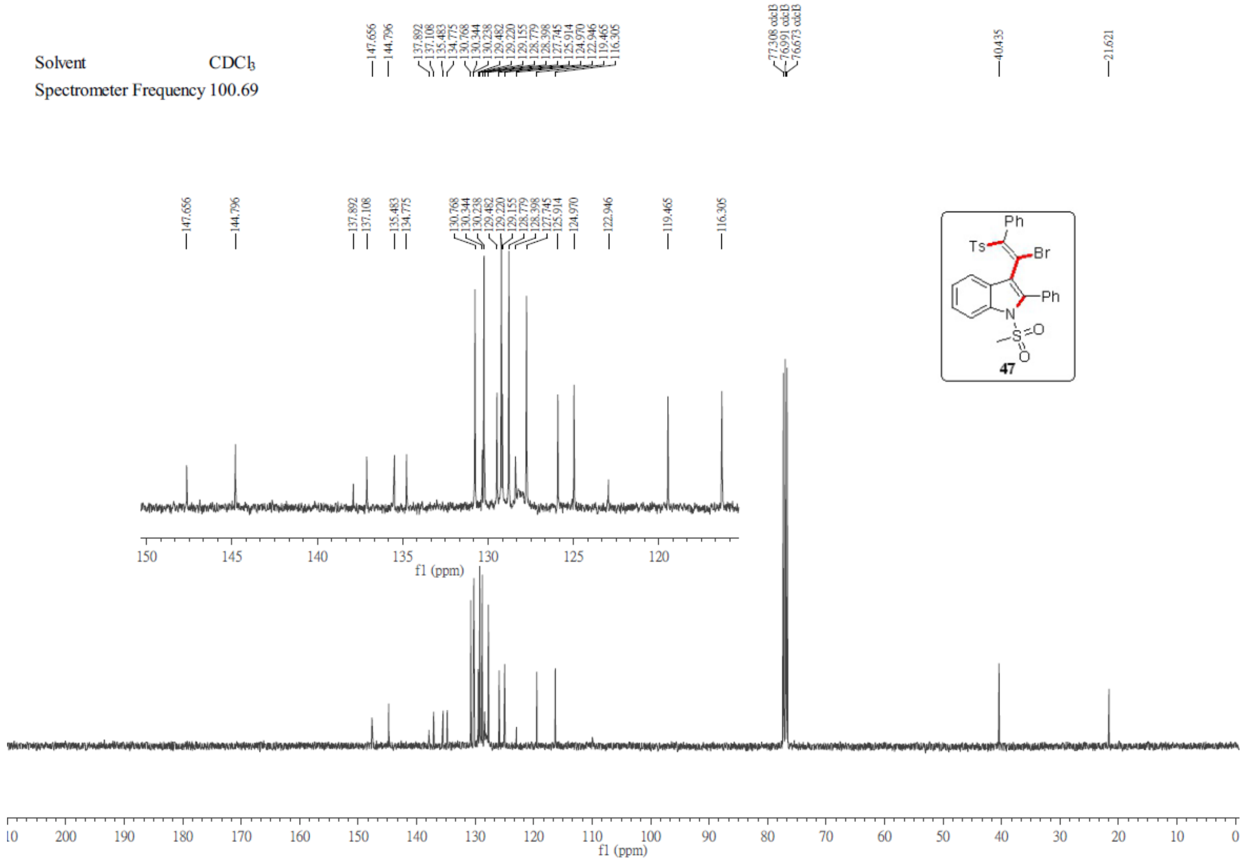

Supplementary Figure 134. ^{1}H (top) and ^{13}C (bottom) NMR spectra of compound **47**.

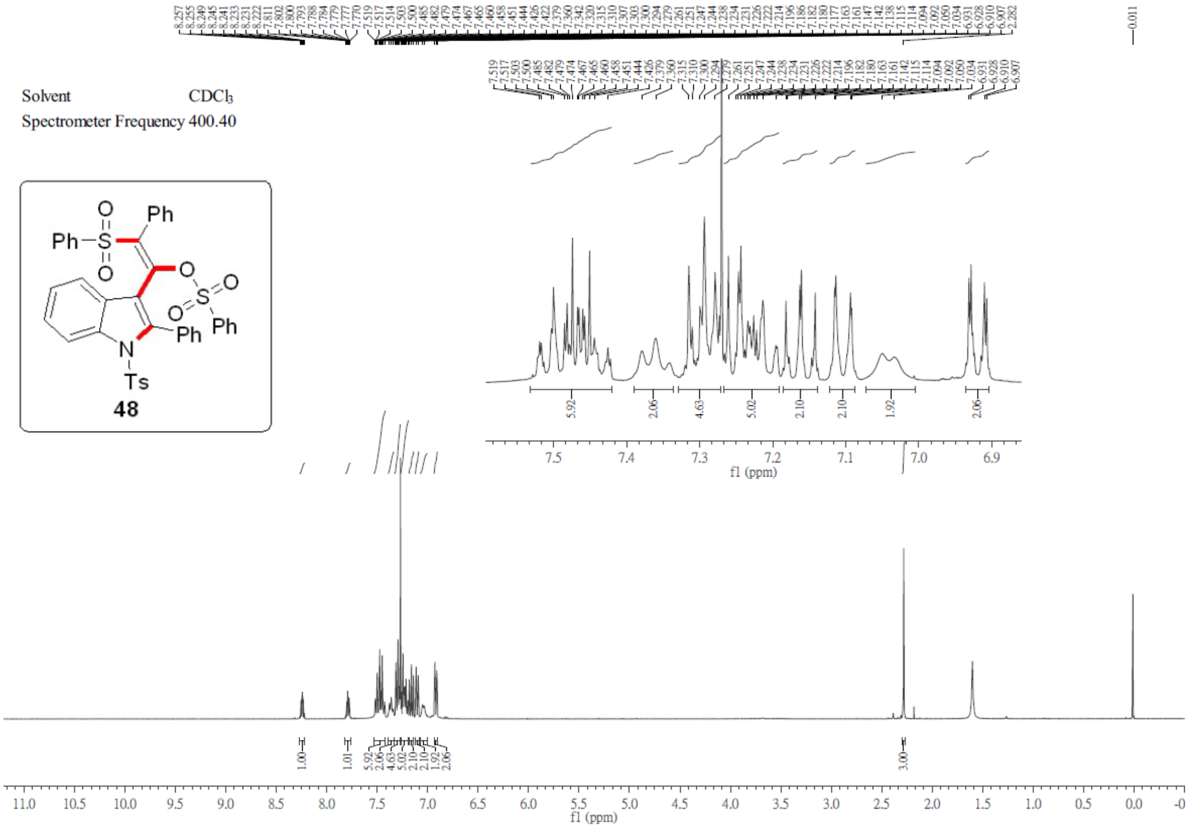

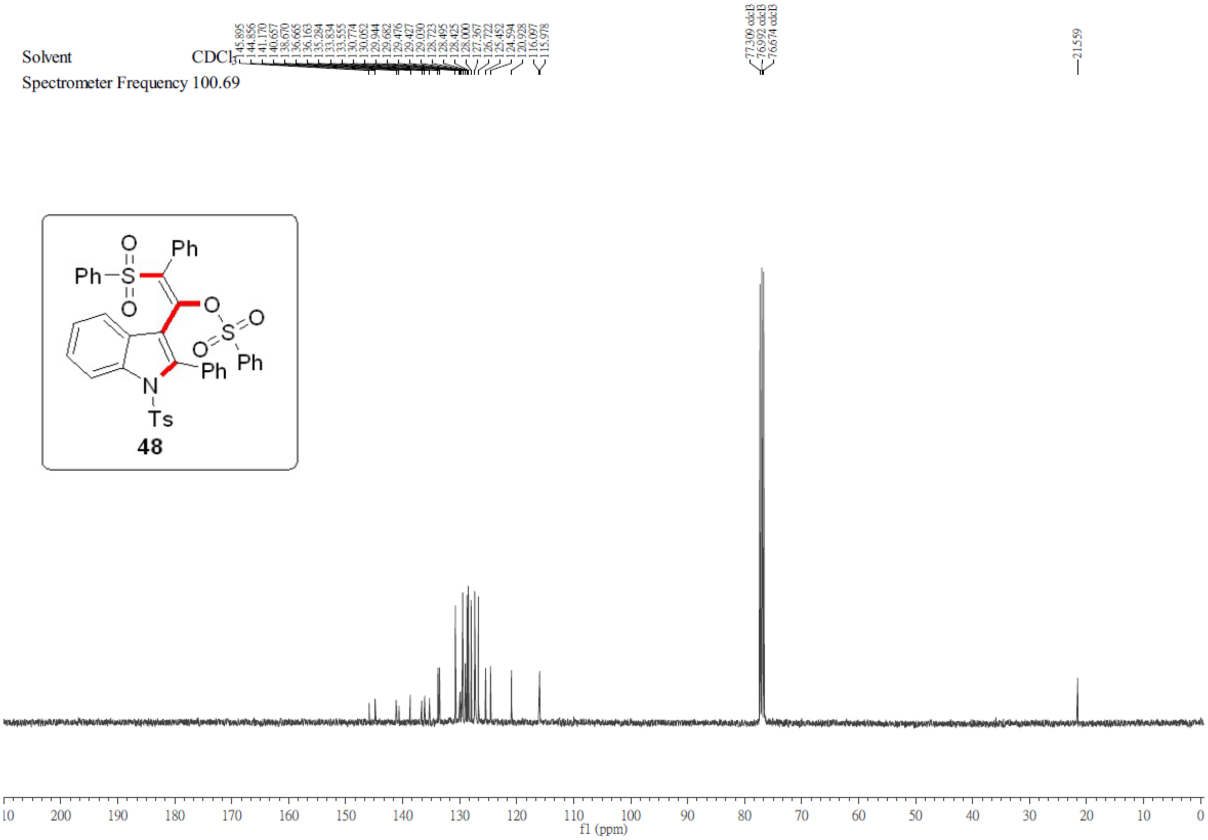

Supplementary Figure 135. ^{1}H (top) and ^{13}C (bottom) NMR spectra of compound **48**.

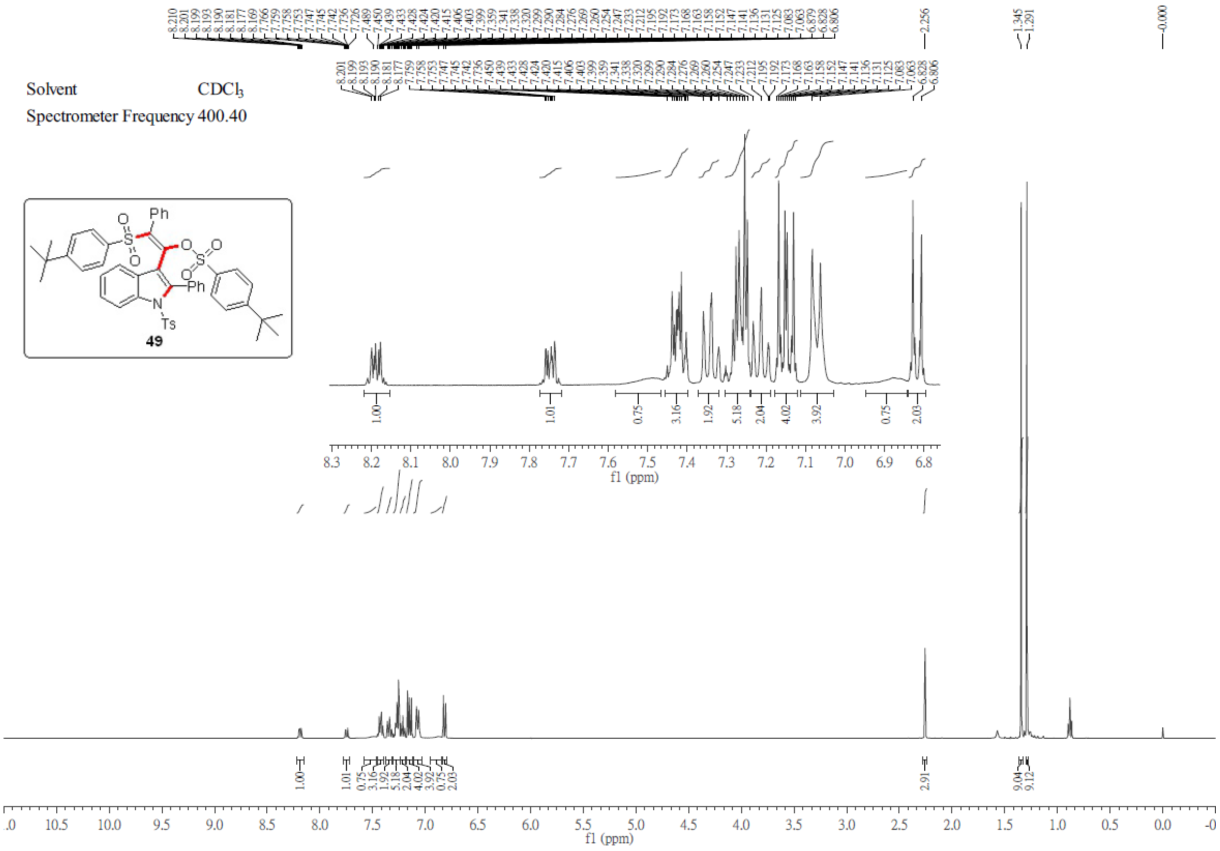

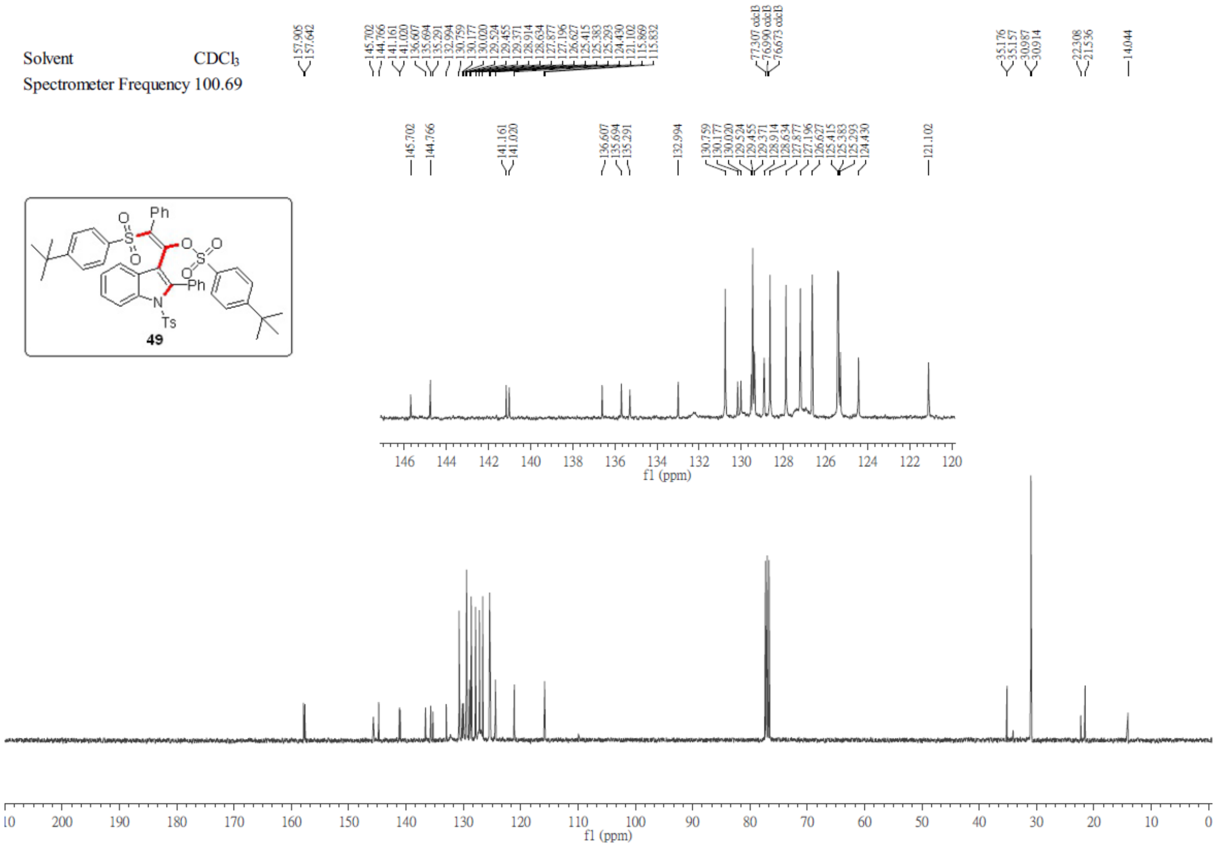

Supplementary Figure 136. ^{1}H (top) and ^{13}C (bottom) NMR spectra of compound **49**.

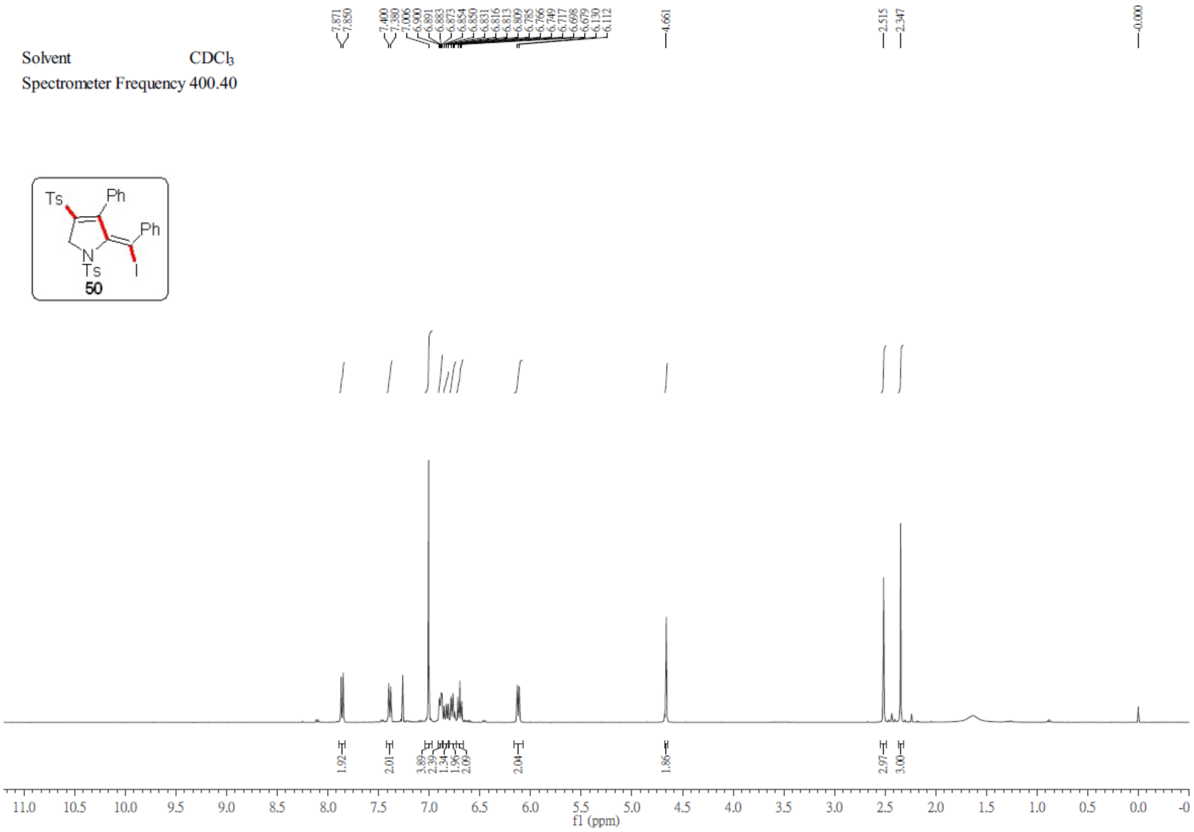

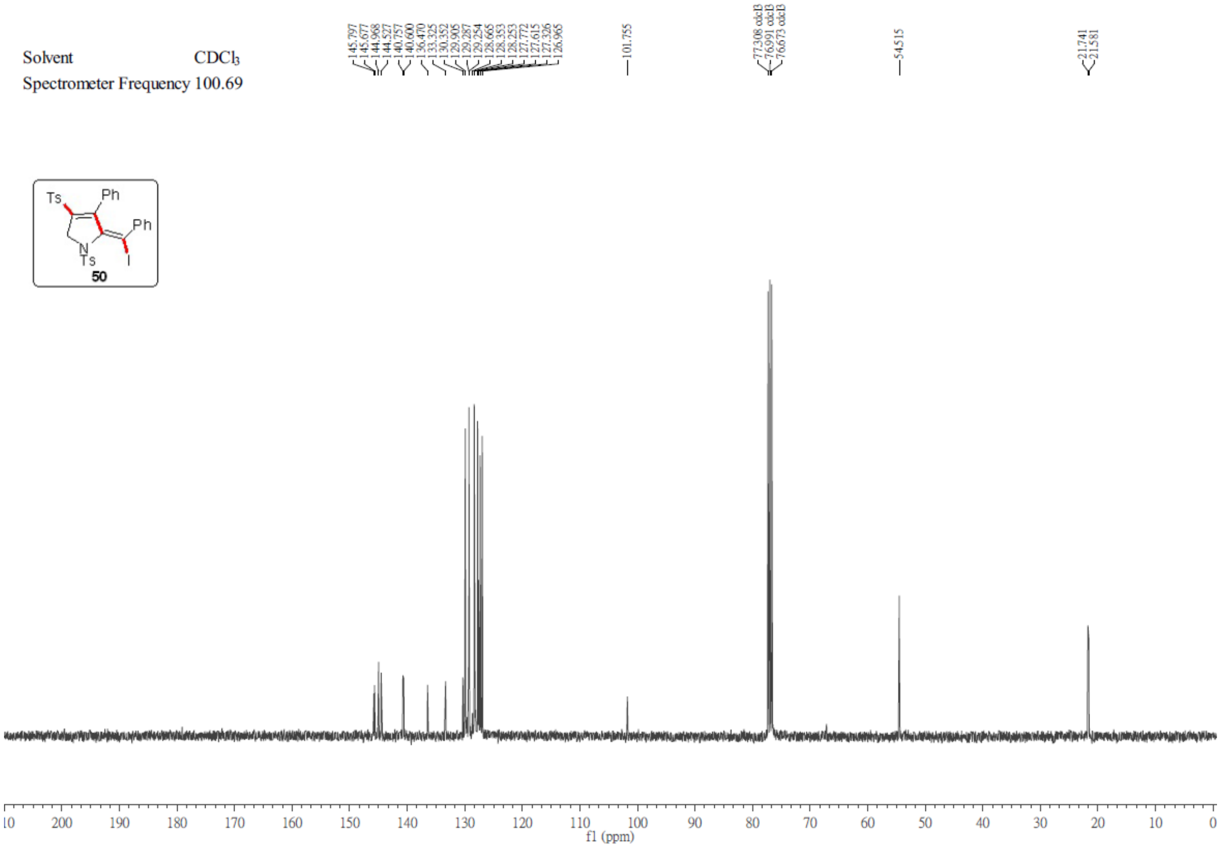

Supplementary Figure 137. ^{1}H (top) and ^{13}C (bottom) NMR spectra of compound **50**.

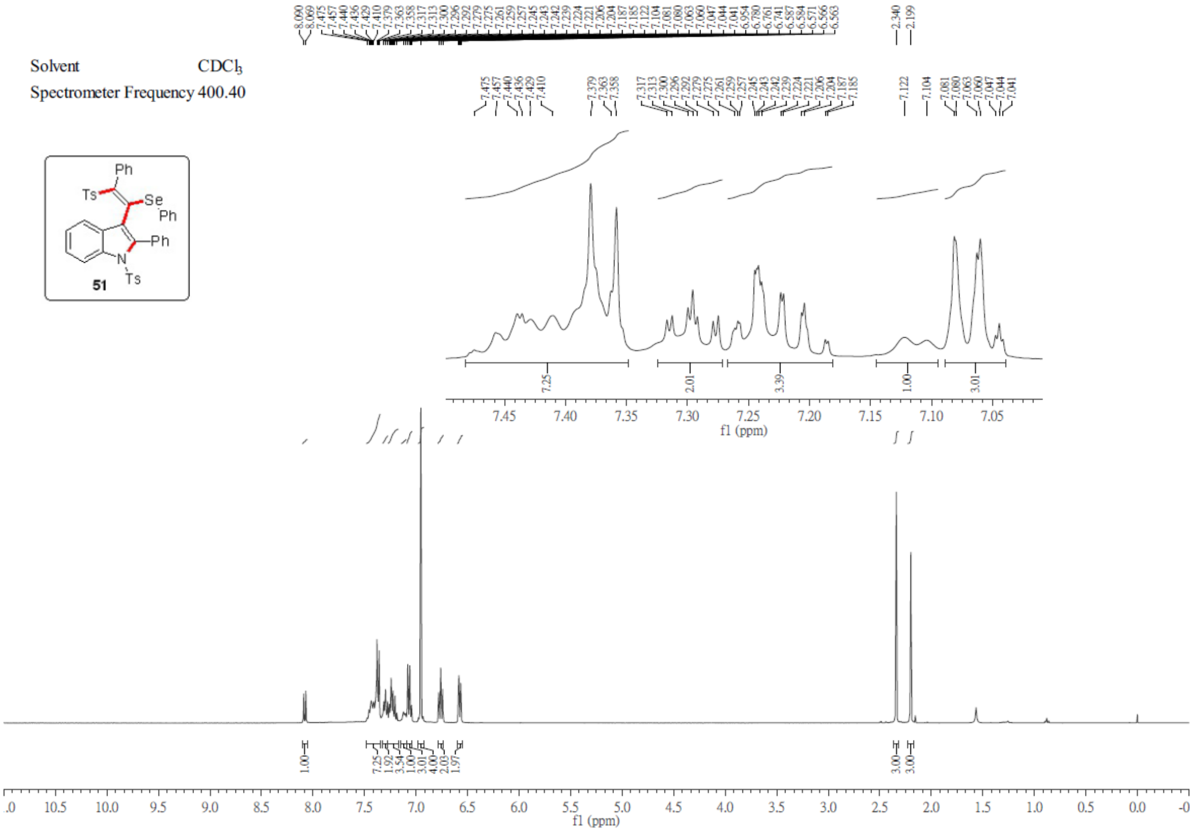

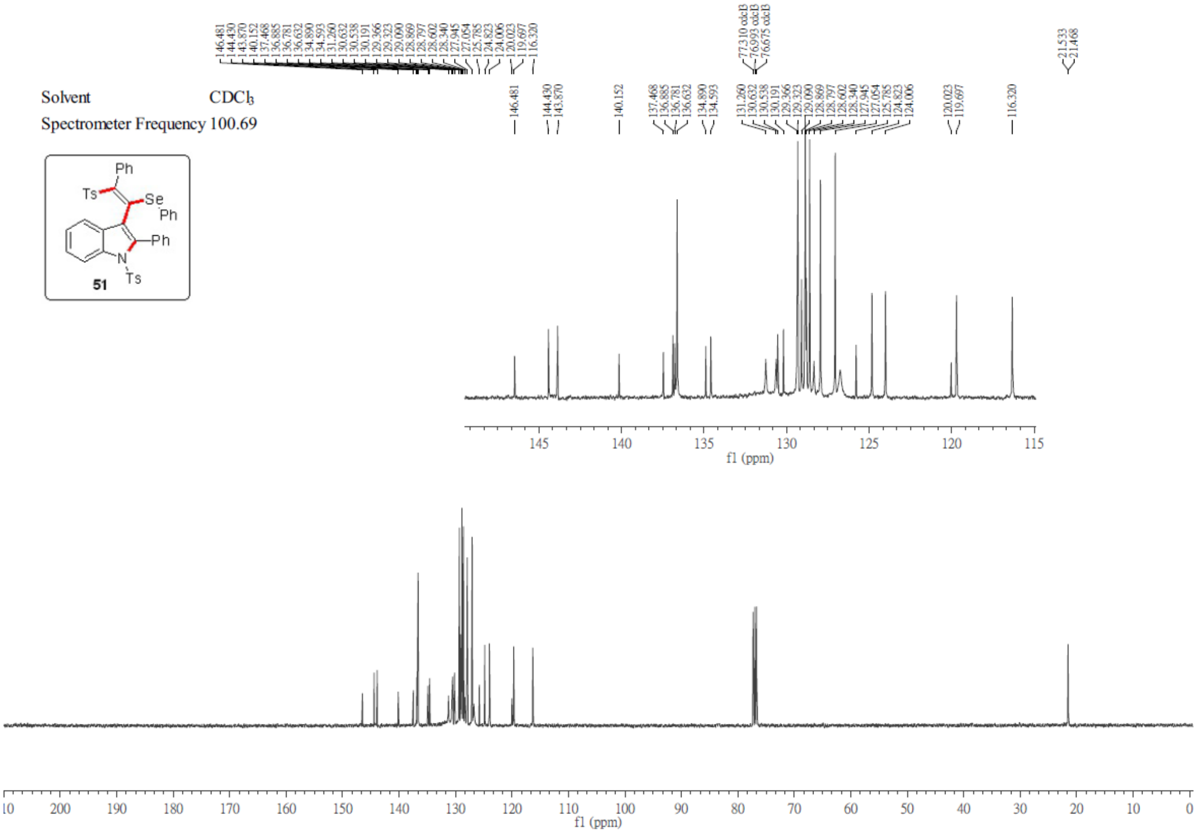

Supplementary Figure 138. ^{1}H (top) and ^{13}C (bottom) NMR spectra of compound **51**.

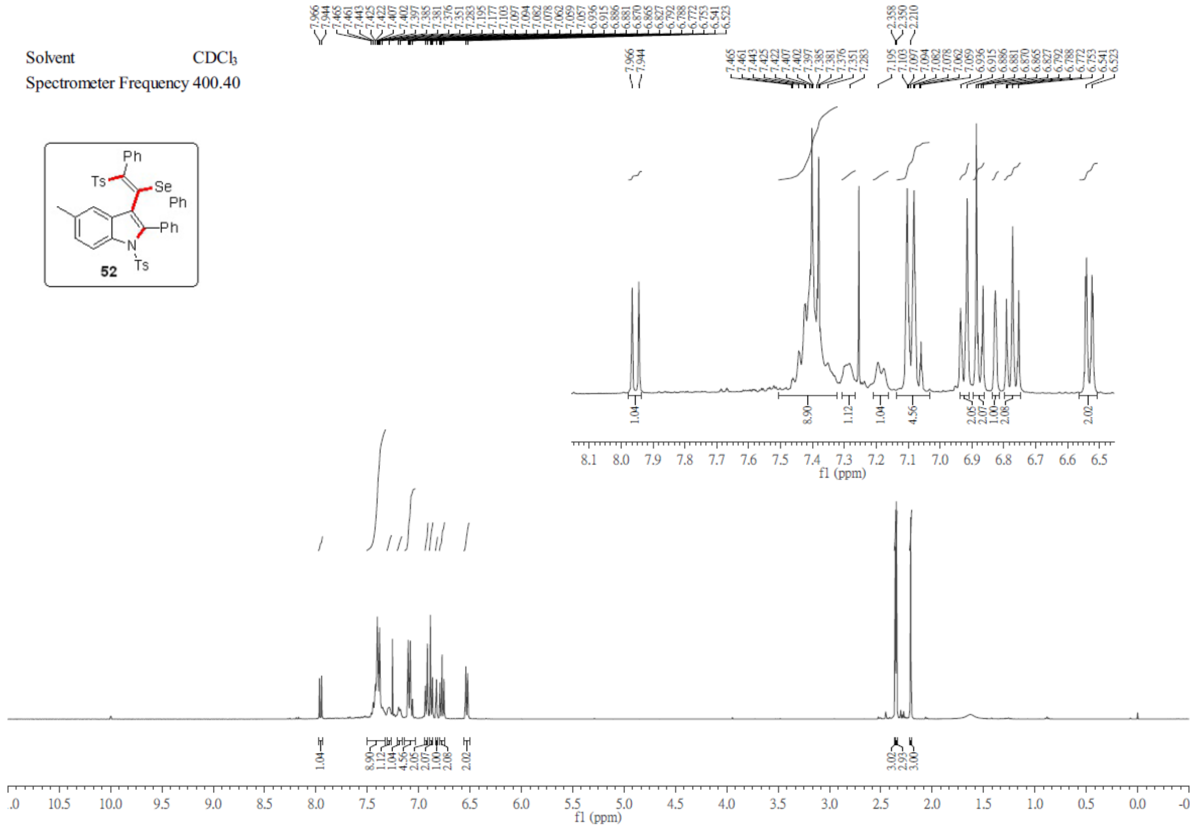

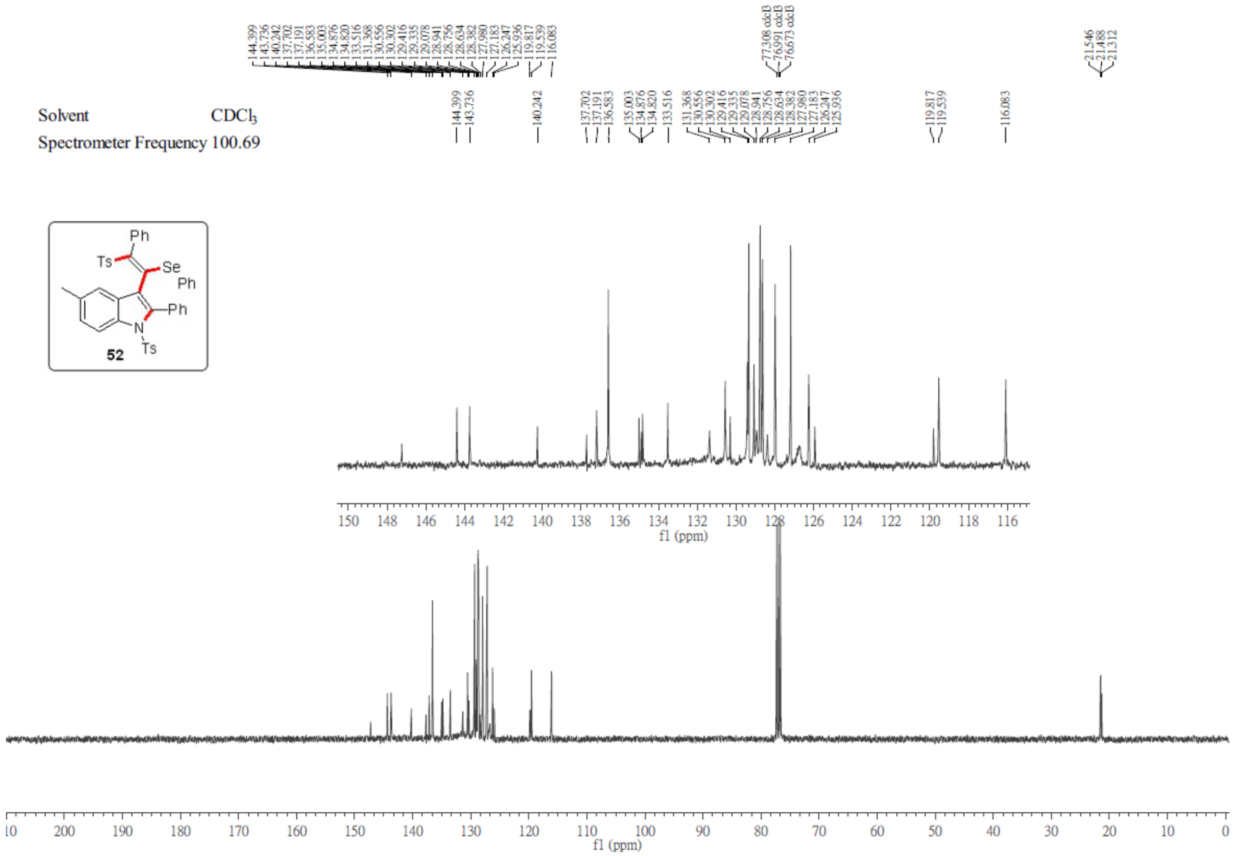

Supplementary Figure 139. ^{1}H (top) and ^{13}C (bottom) NMR spectra of compound **52**.

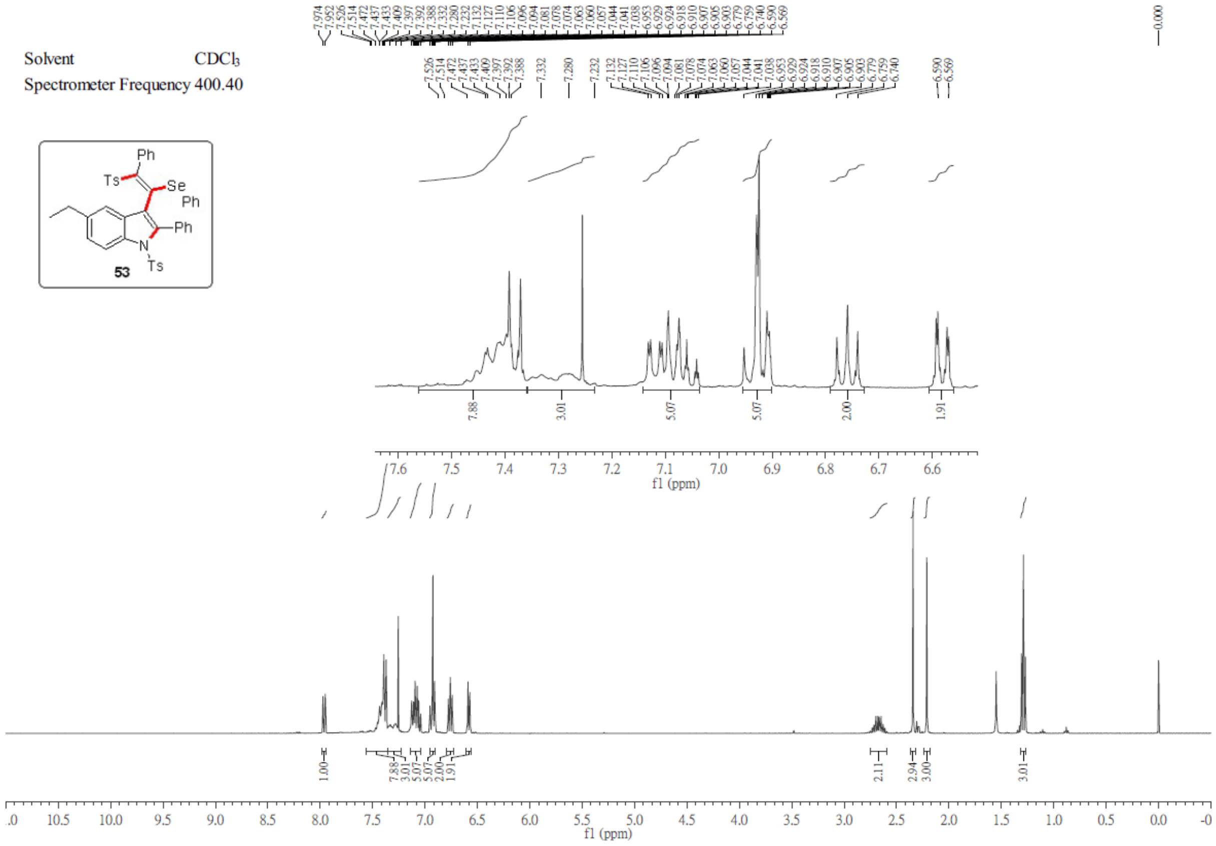

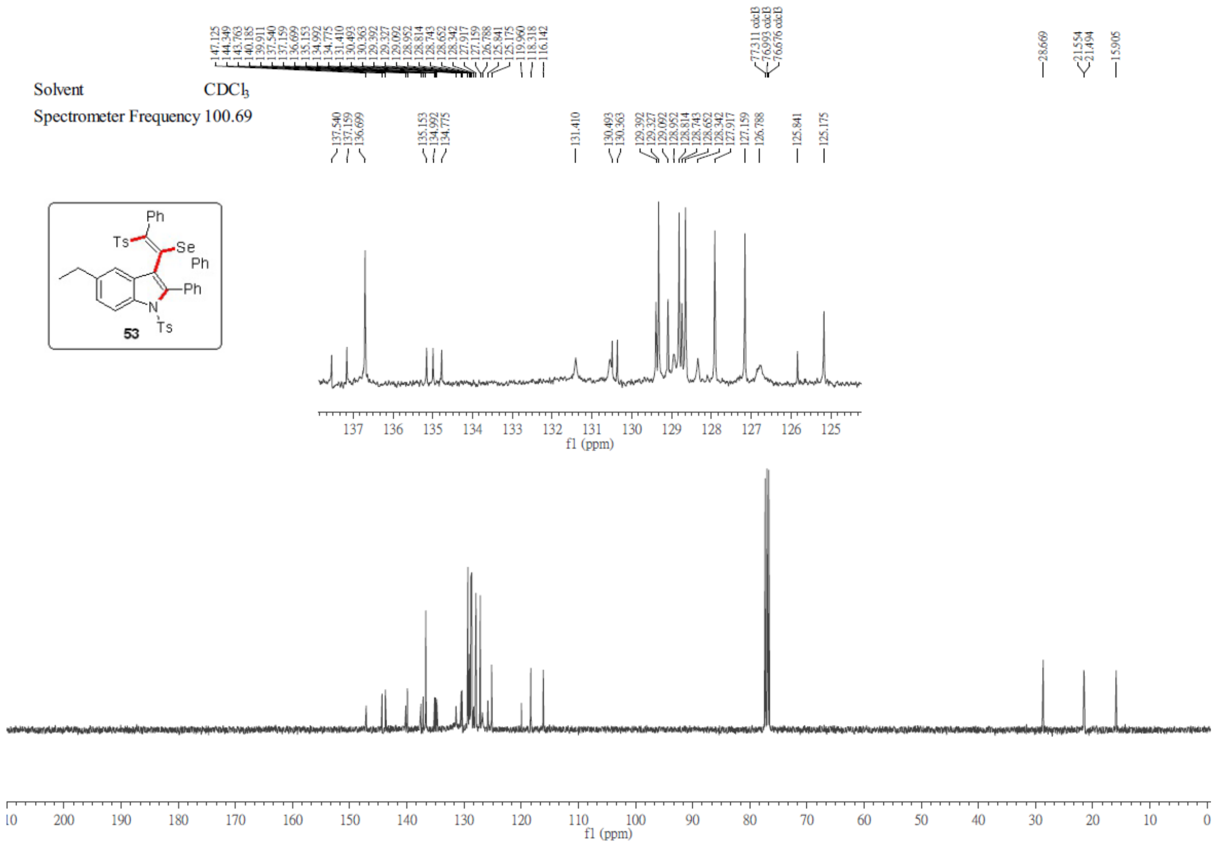

Supplementary Figure 140. ^{1}H (top) and ^{13}C (bottom) NMR spectra of compound **53**.

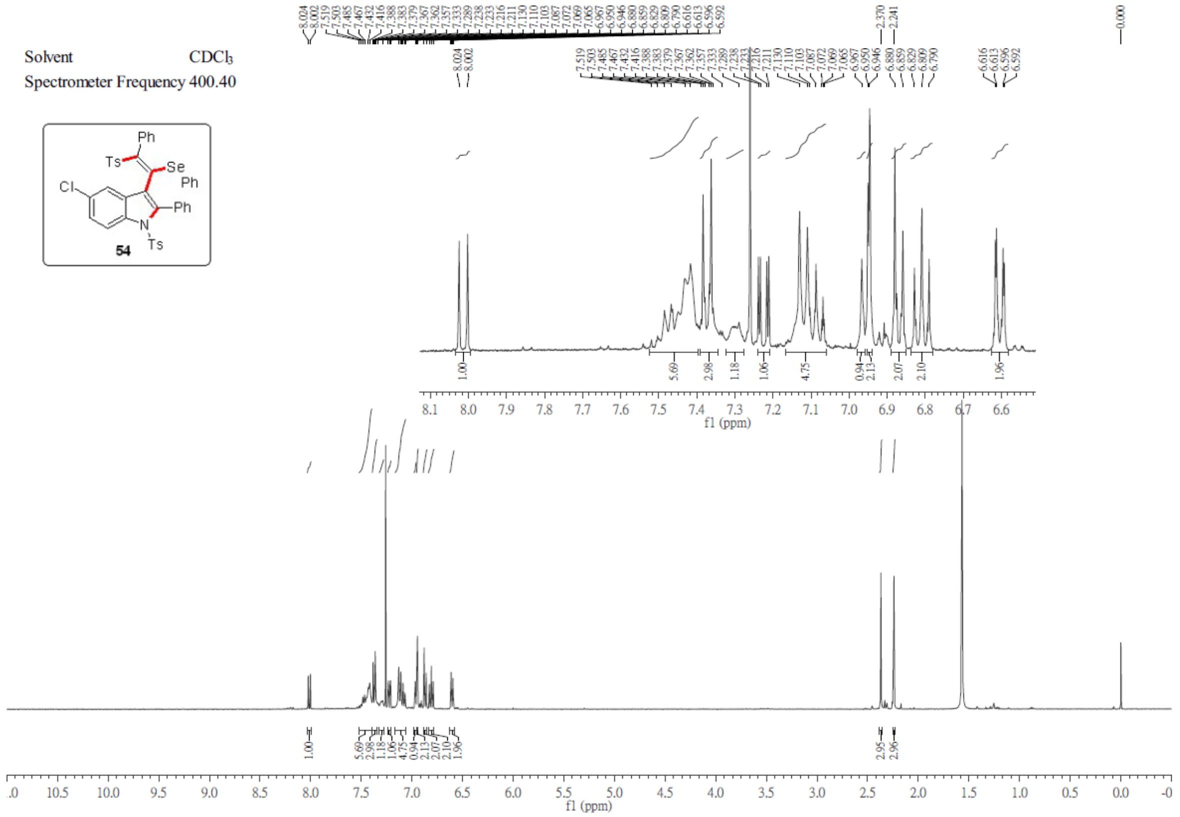

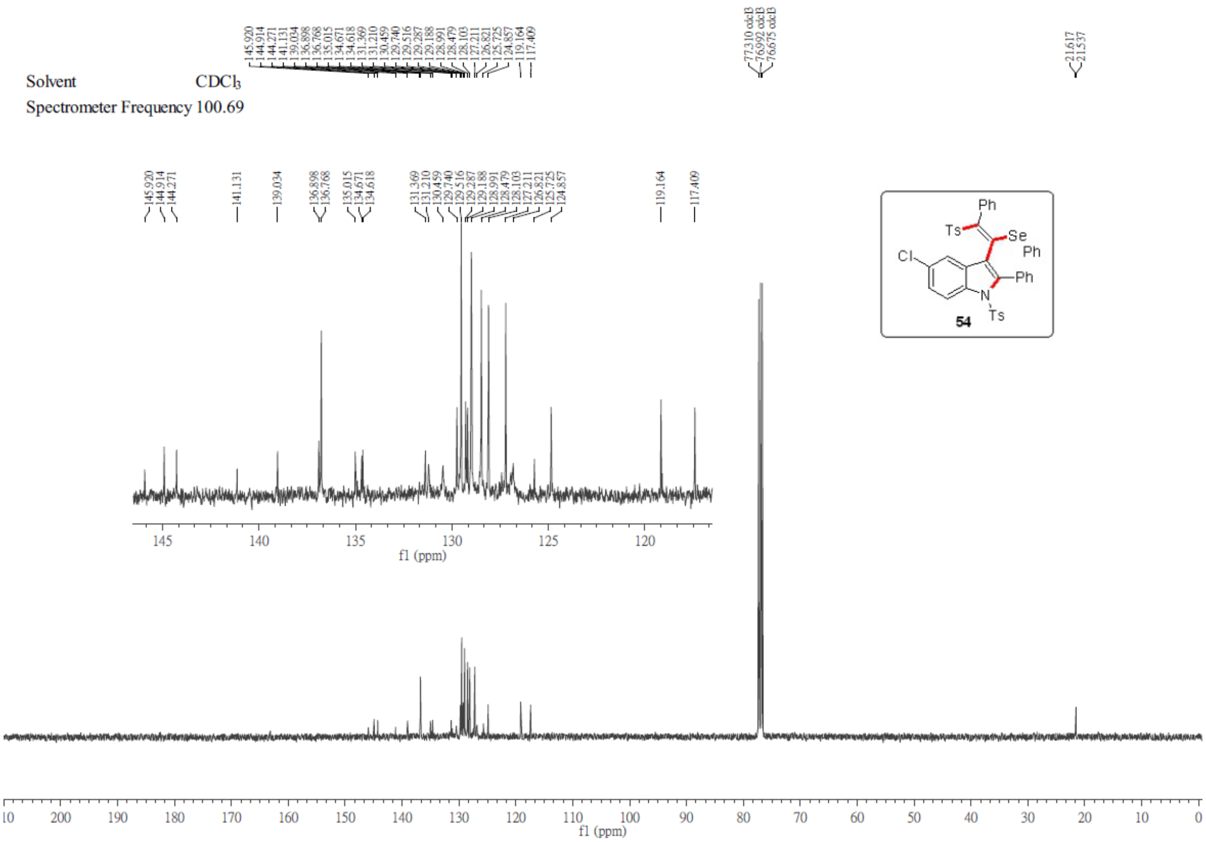

Supplementary Figure 141. ^{1}H (top) and ^{13}C (bottom) NMR spectra of compound **54**.

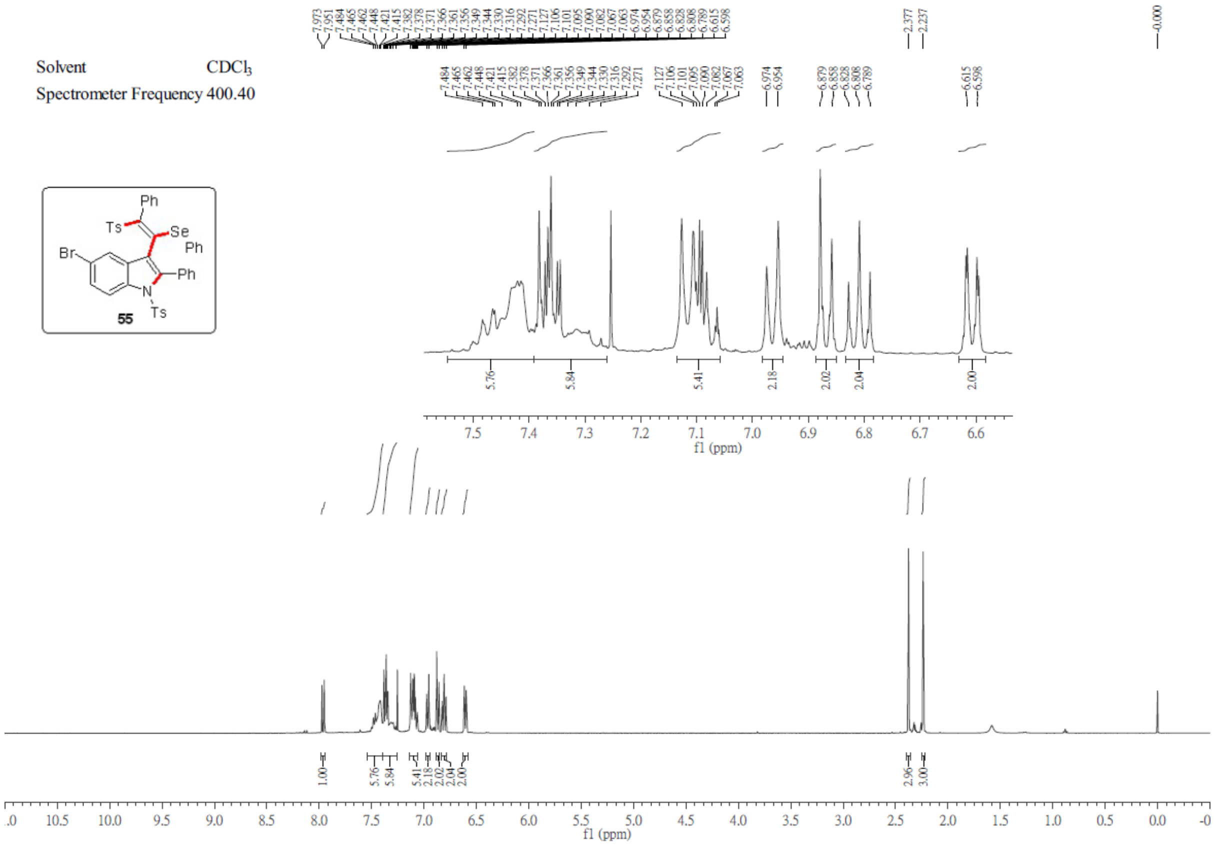

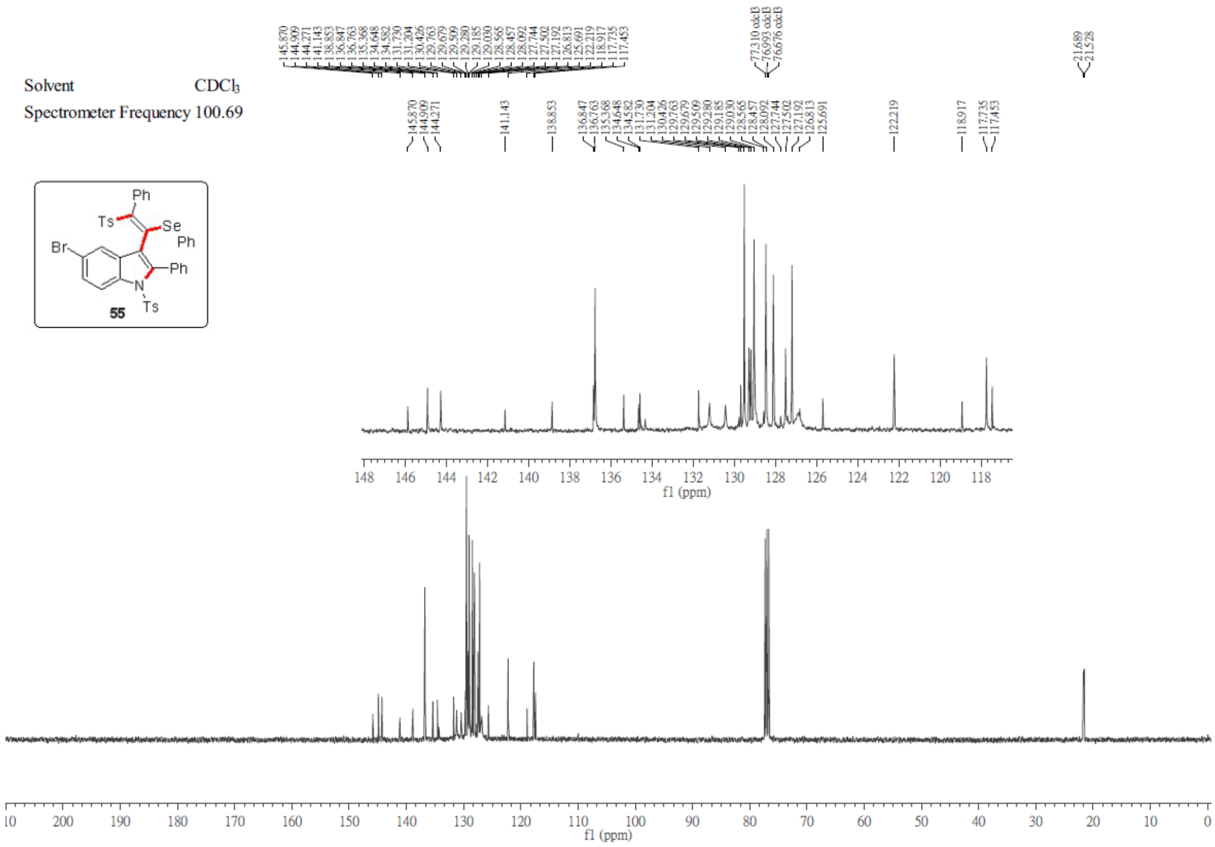

Supplementary Figure 142. ^{1}H (top) and ^{13}C (bottom) NMR spectra of compound **55**.

Solvent CDCl3
Spectrometer Frequency 400.40

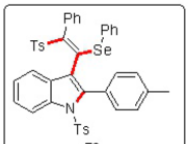

56

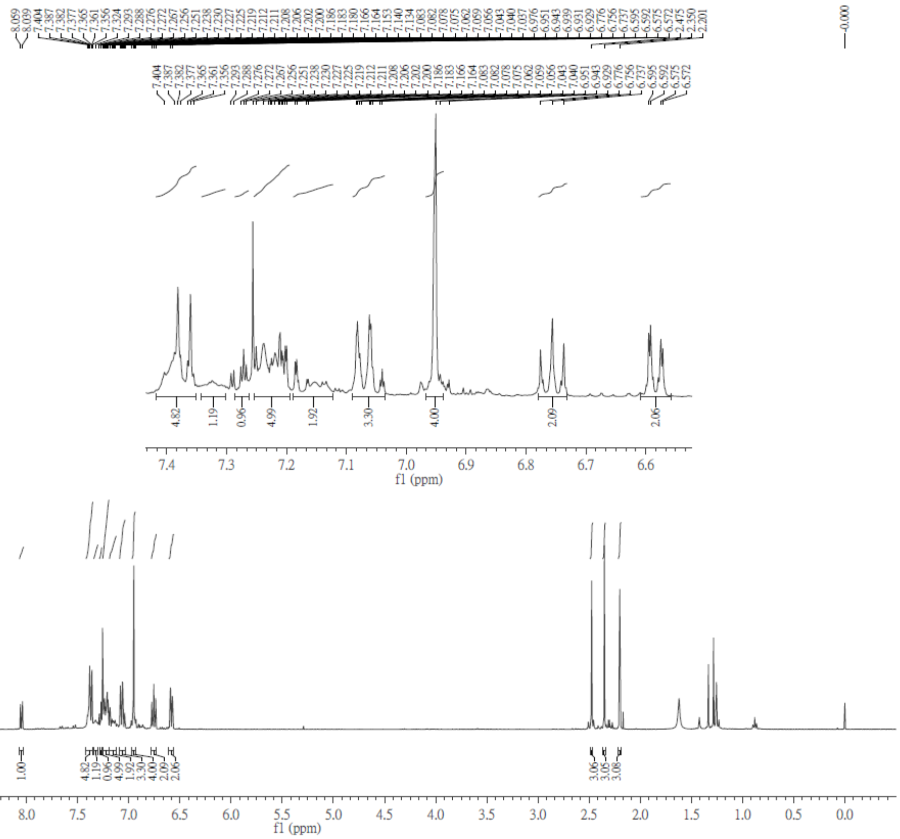

Solvent CDCl3
Spectrometer Frequency 100.69

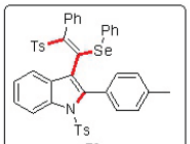

56

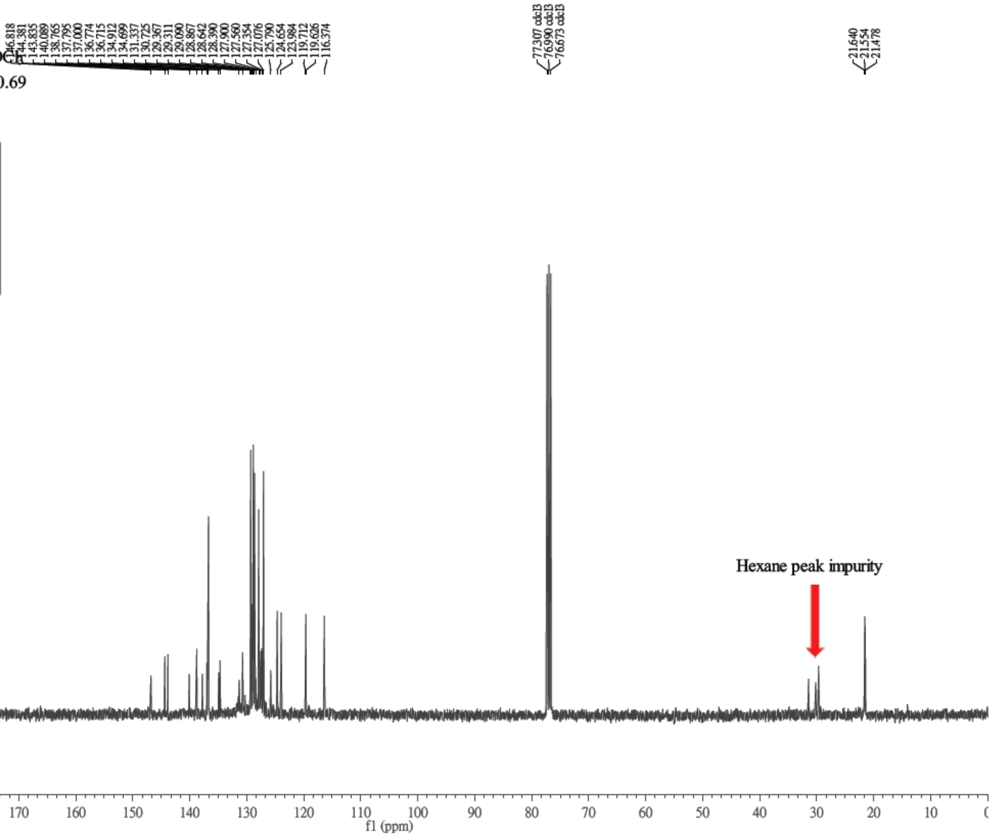

Supplementary Figure 143. ^{1}H (top) and ^{13}C (bottom) NMR spectra of compound **56**.

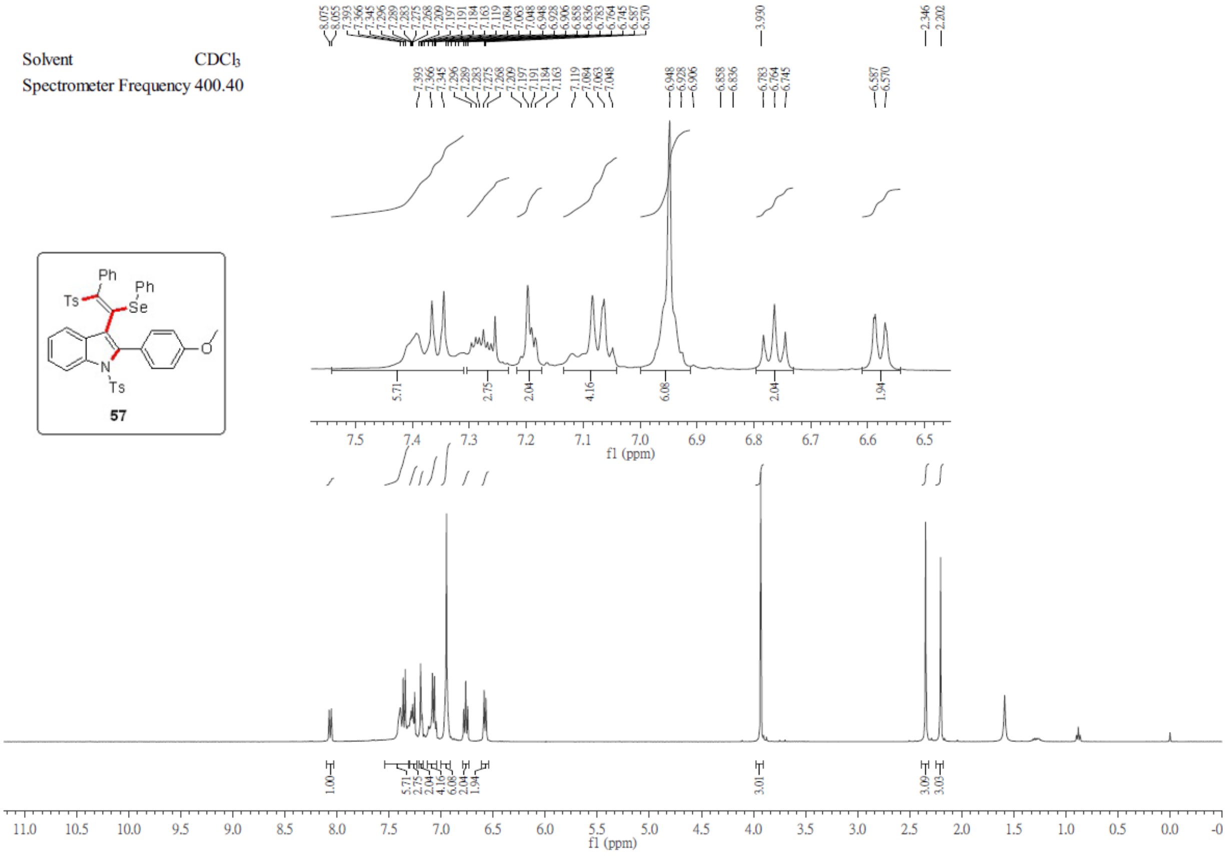

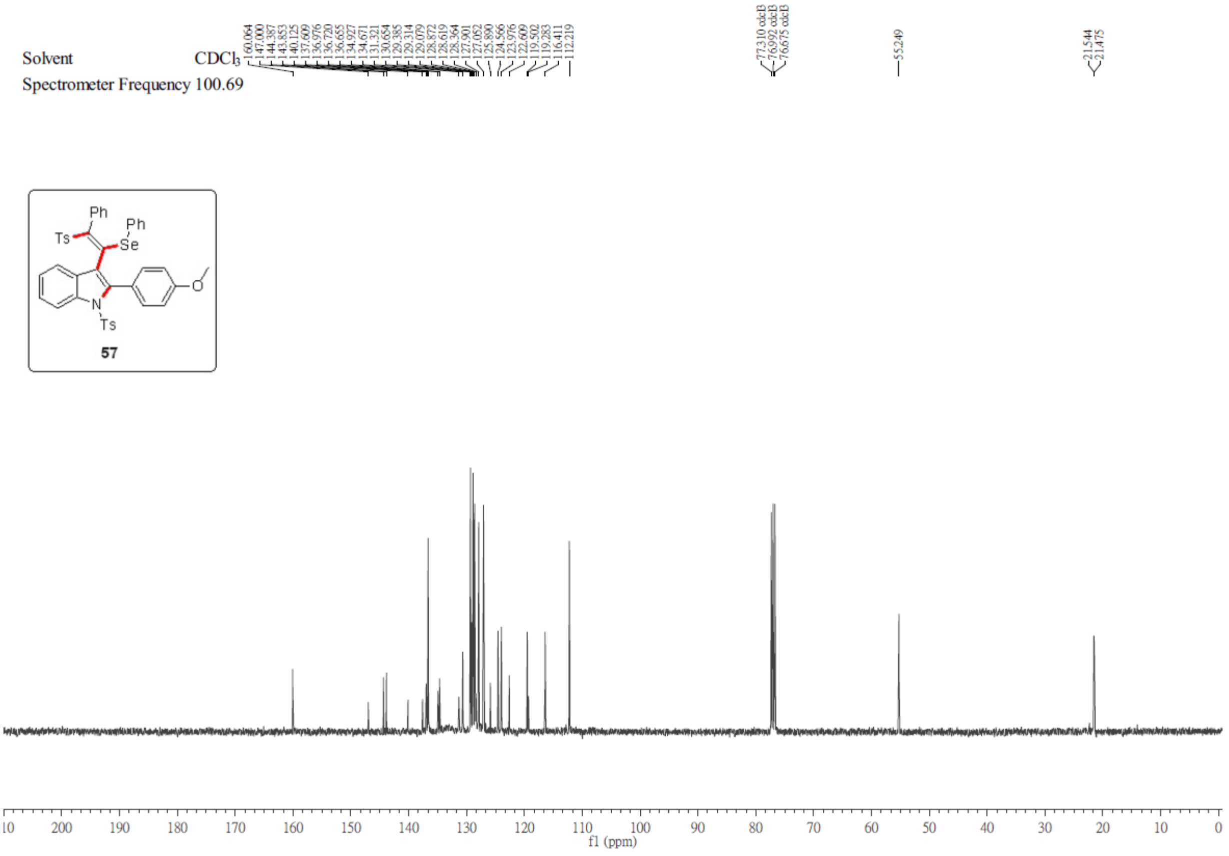

Supplementary Figure 144. ^{1}H (top) and ^{13}C (bottom) NMR spectra of compound **57**.

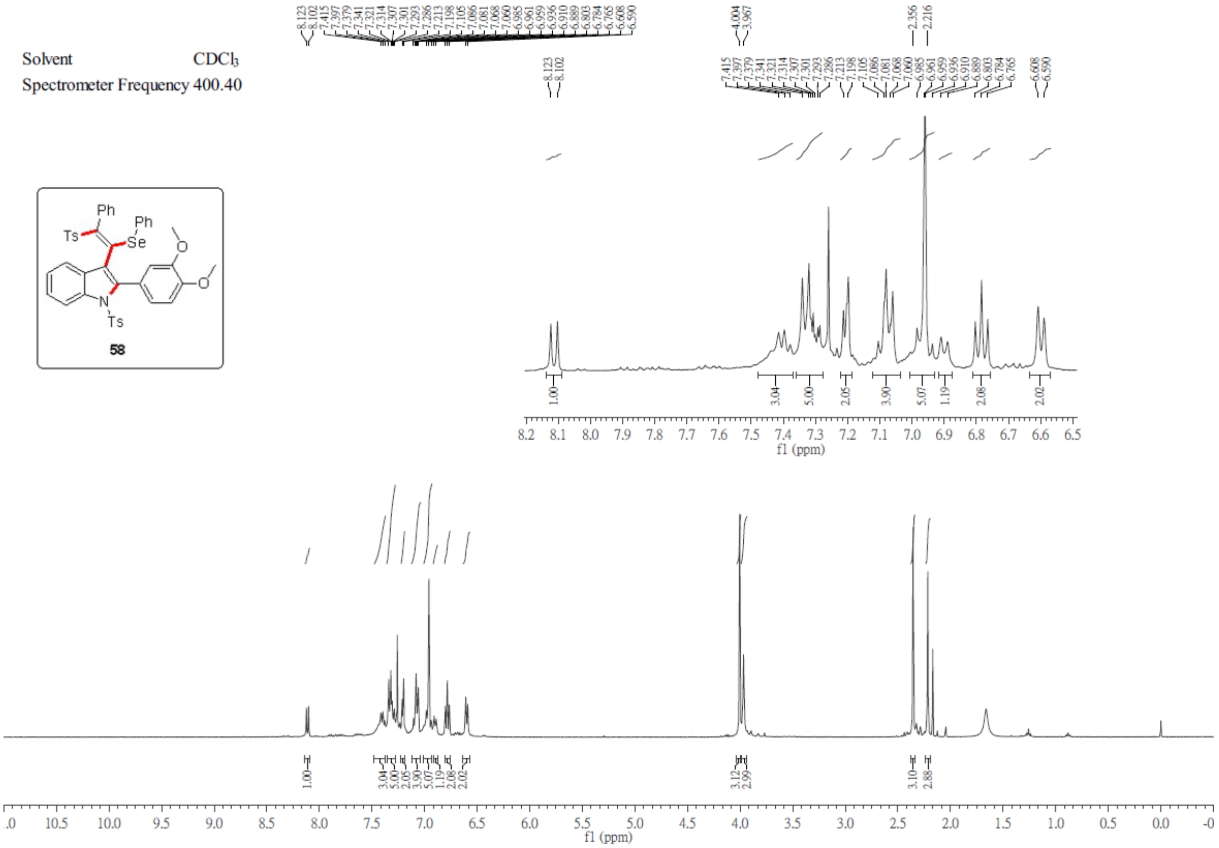

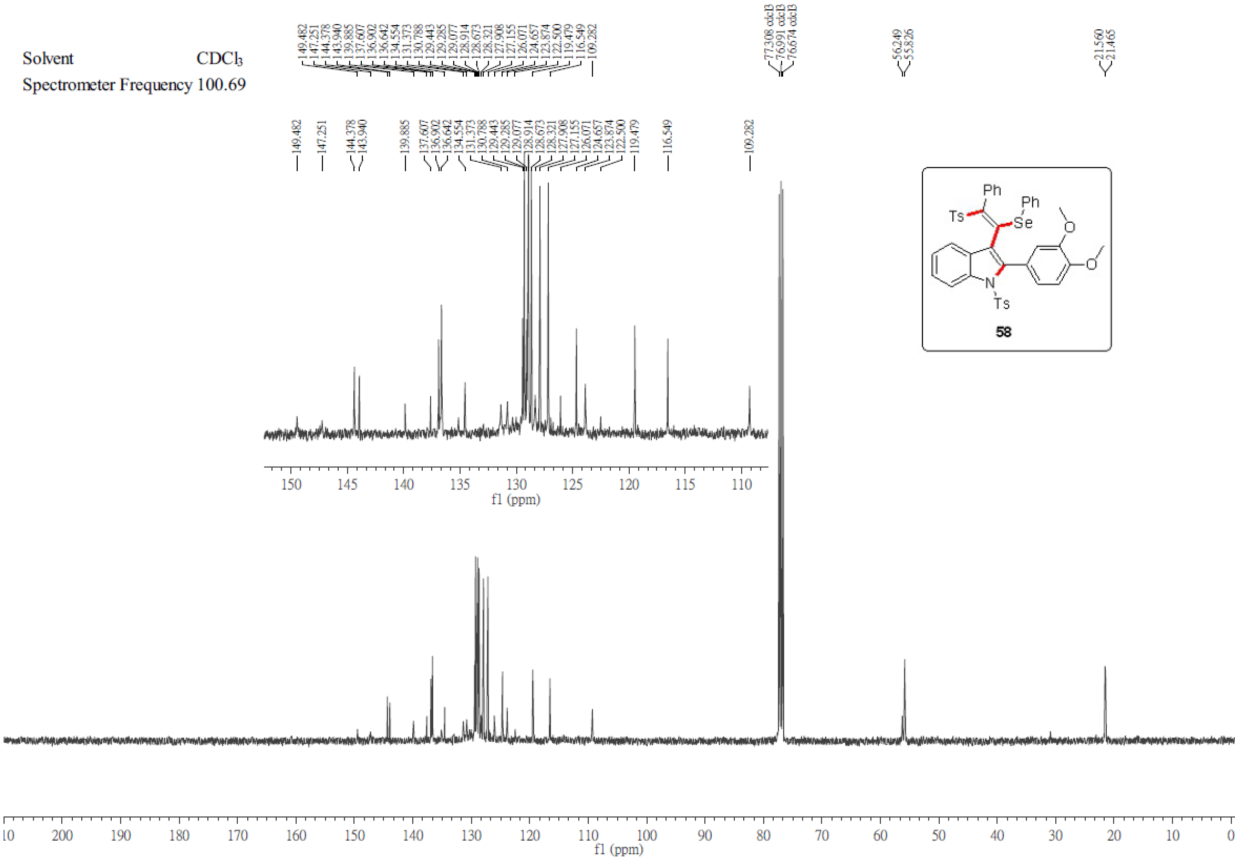

Supplementary Figure 145. ^{1}H (top) and ^{13}C (bottom) NMR spectra of compound **58**.

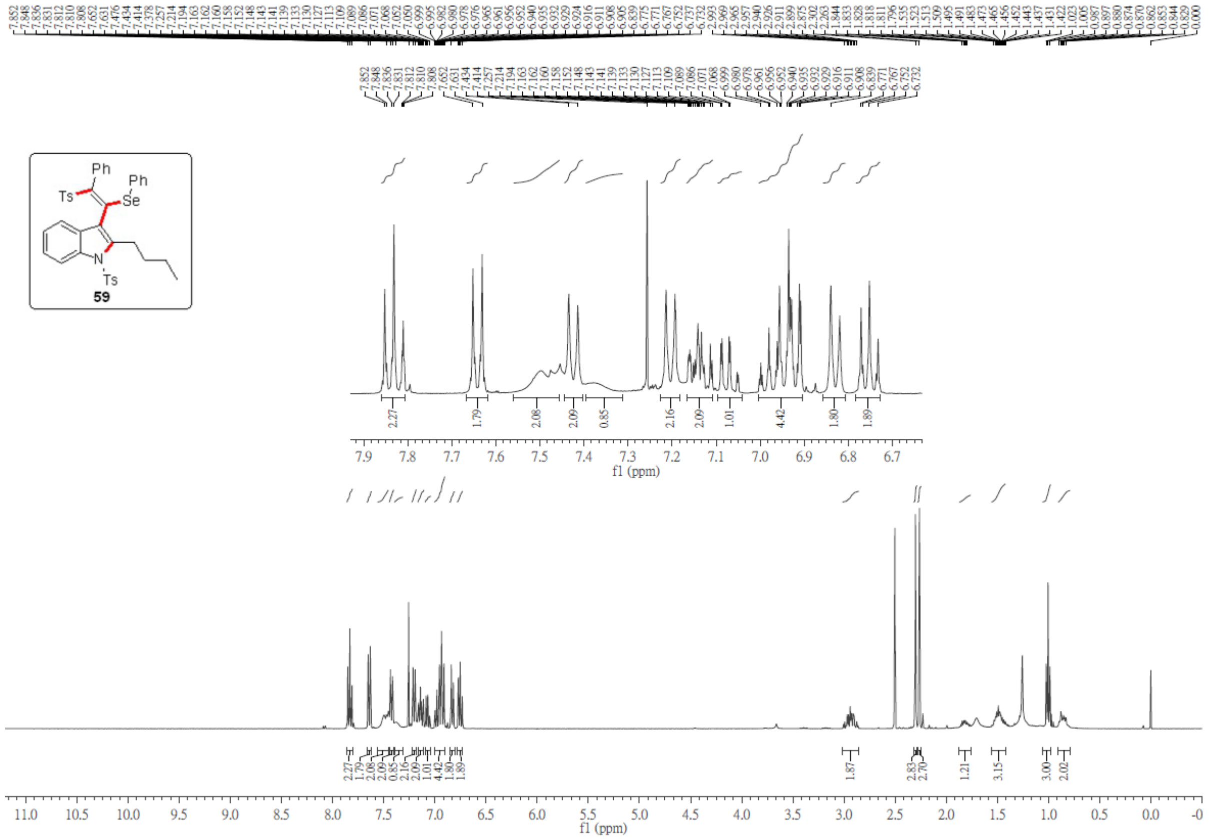

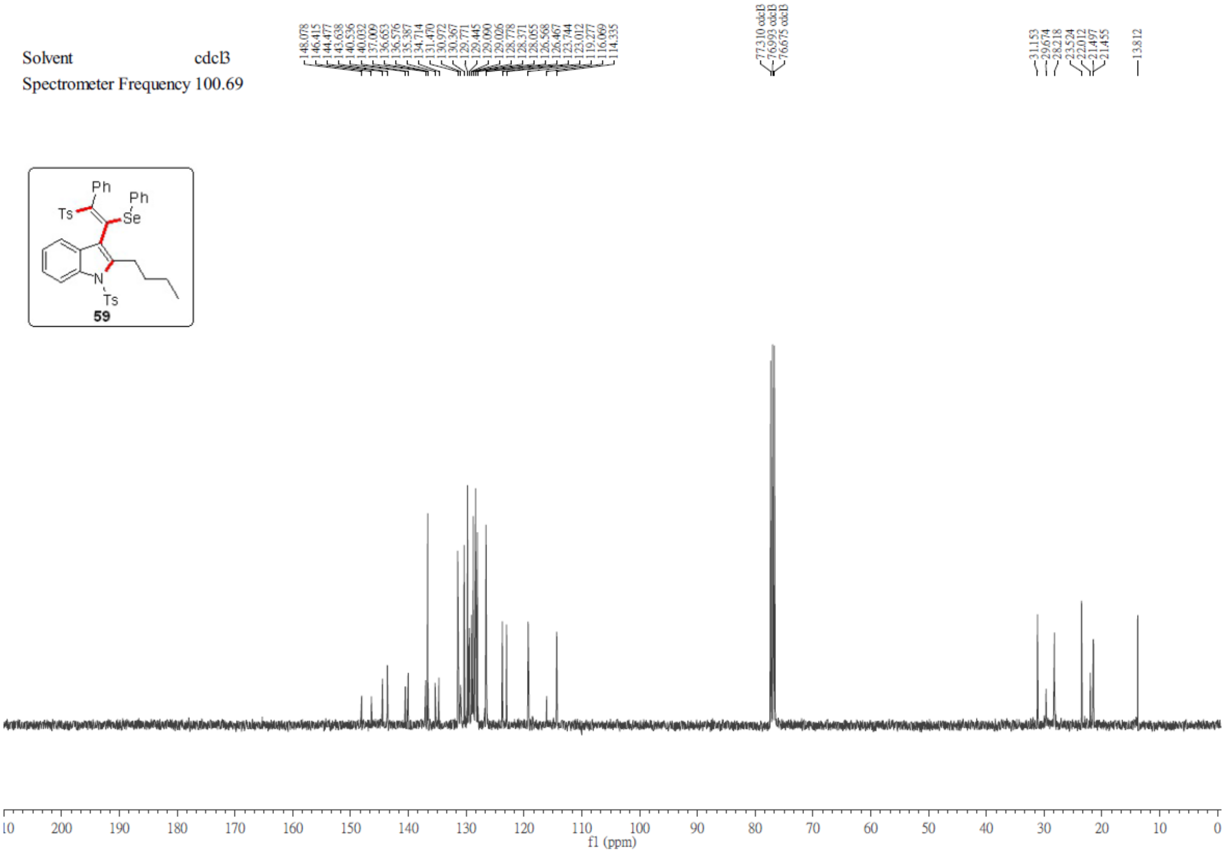

Supplementary Figure 146. ^{1}H (top) and ^{13}C (bottom) NMR spectra of compound **59**.

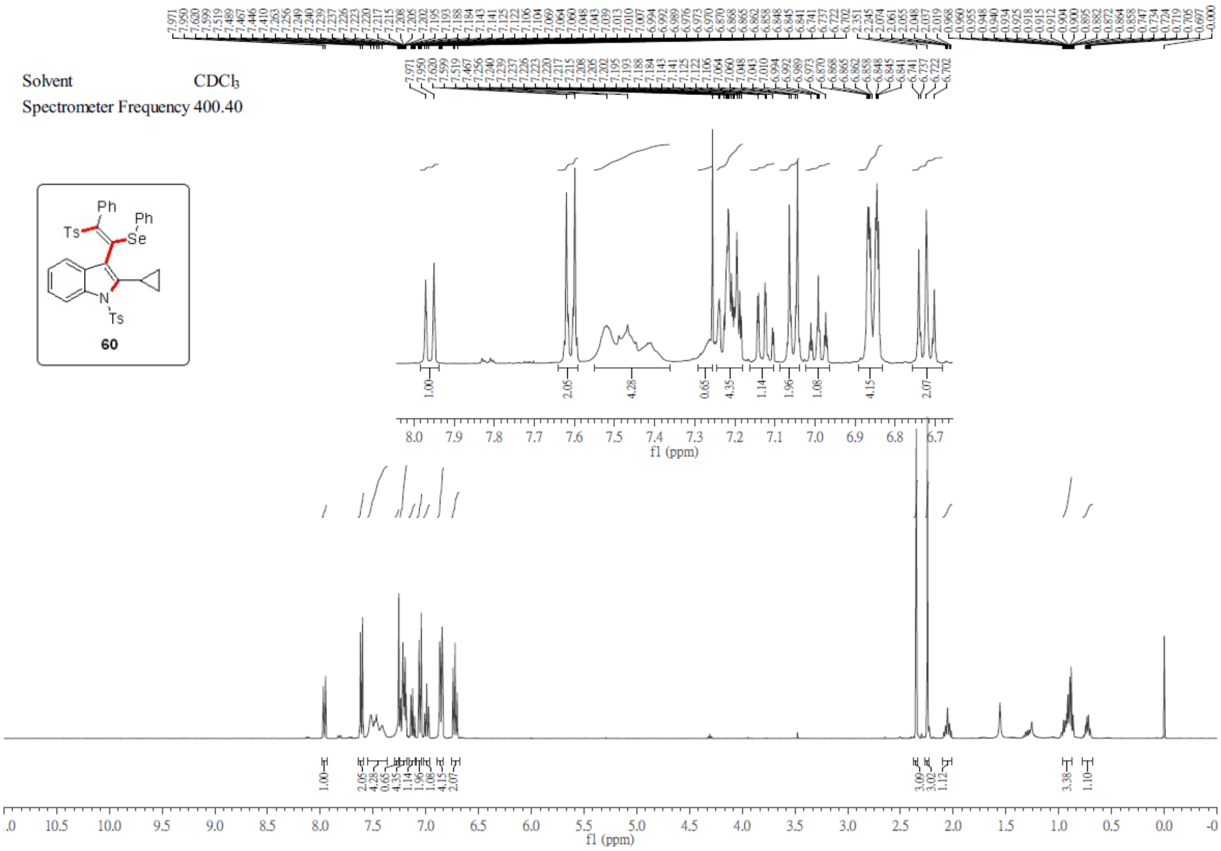

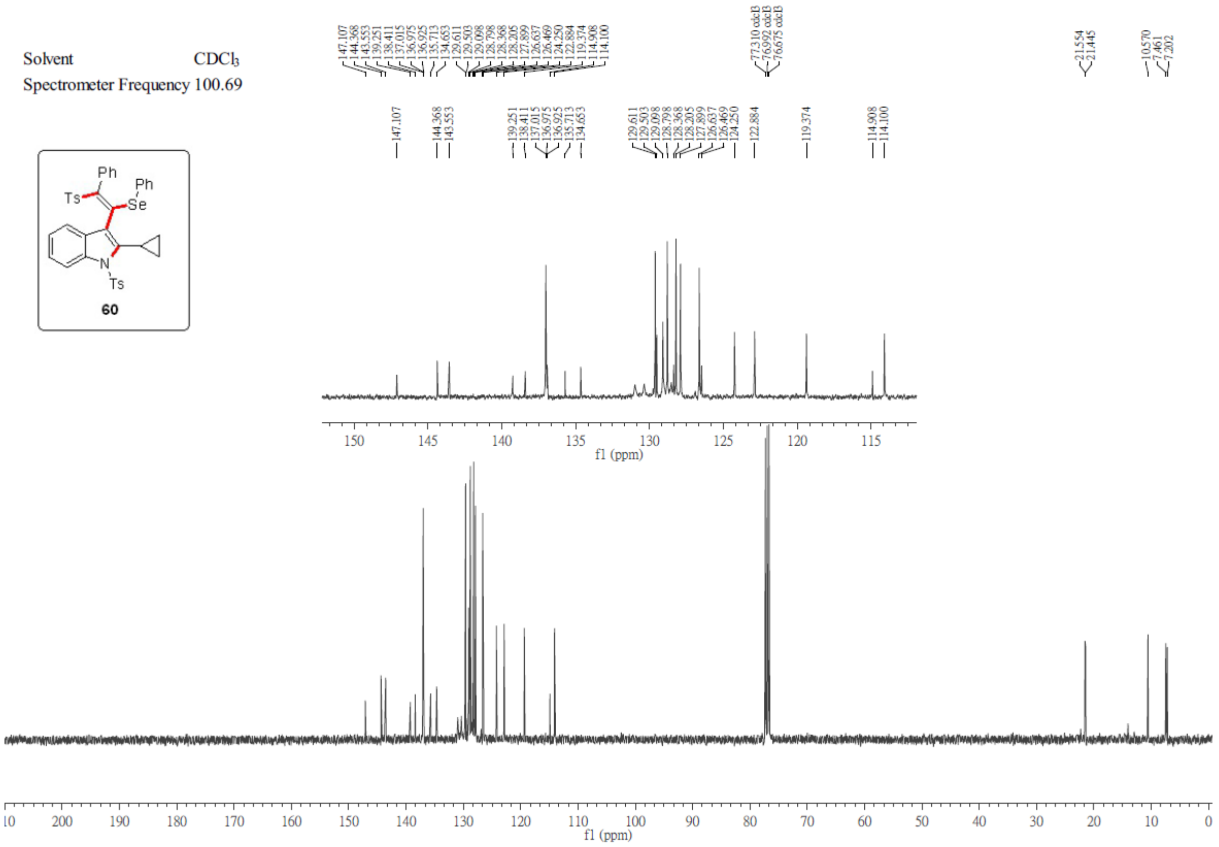

Supplementary Figure 147. ^{1}H (top) and ^{13}C (bottom) NMR spectra of compound **60**.

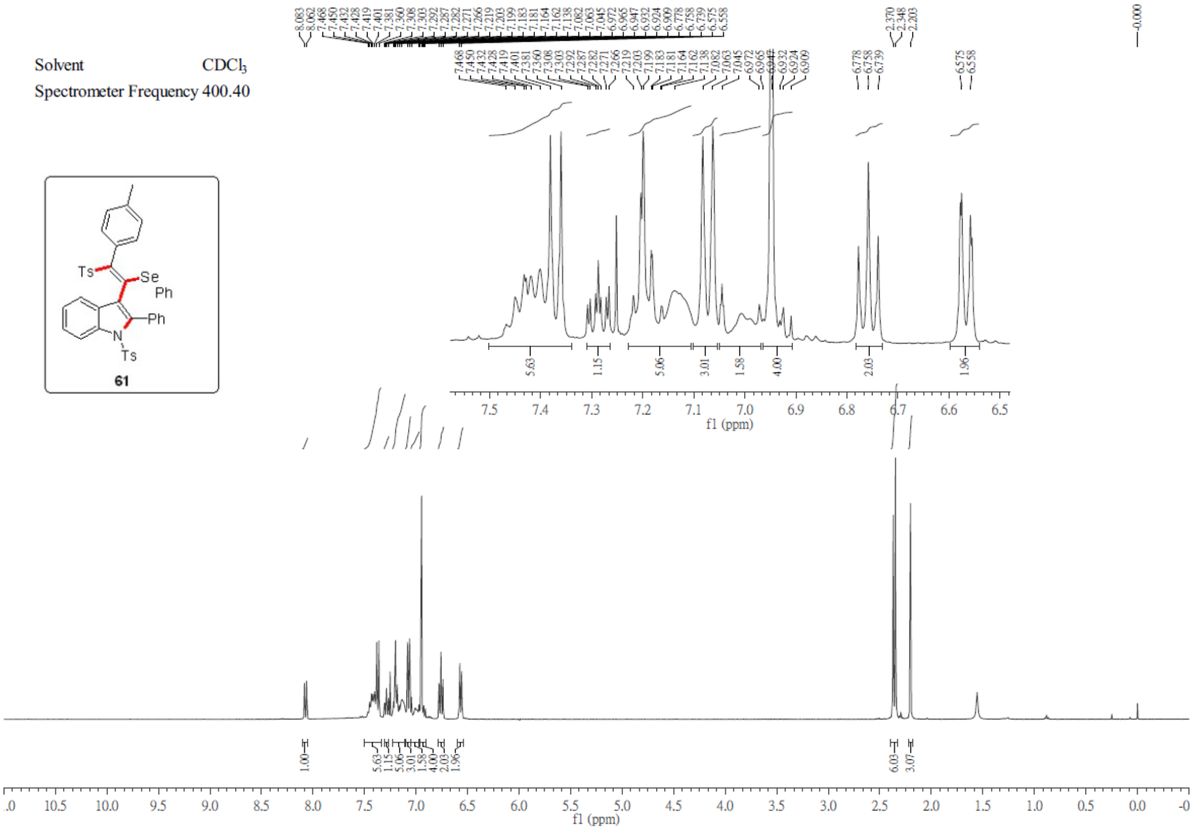

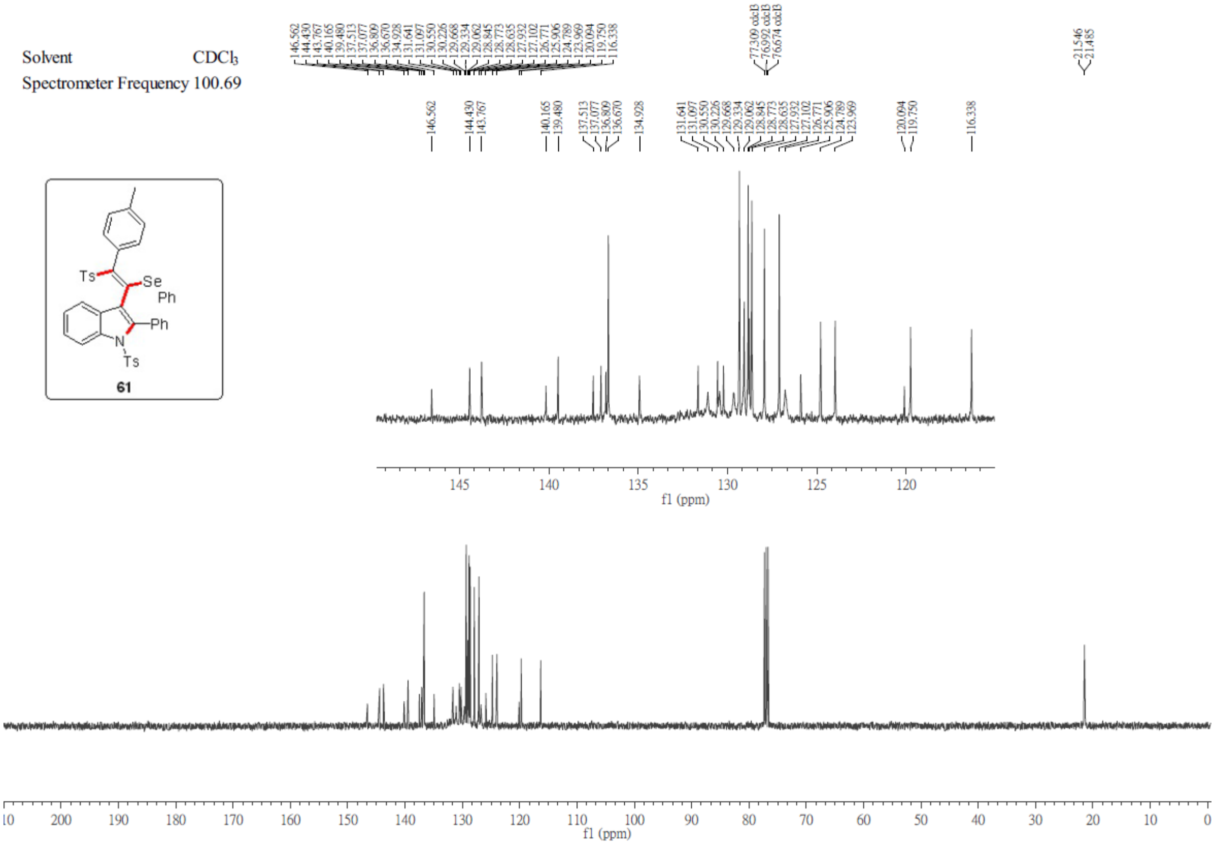

Supplementary Figure 148. ^{1}H (top) and ^{13}C (bottom) NMR spectra of compound **61**.

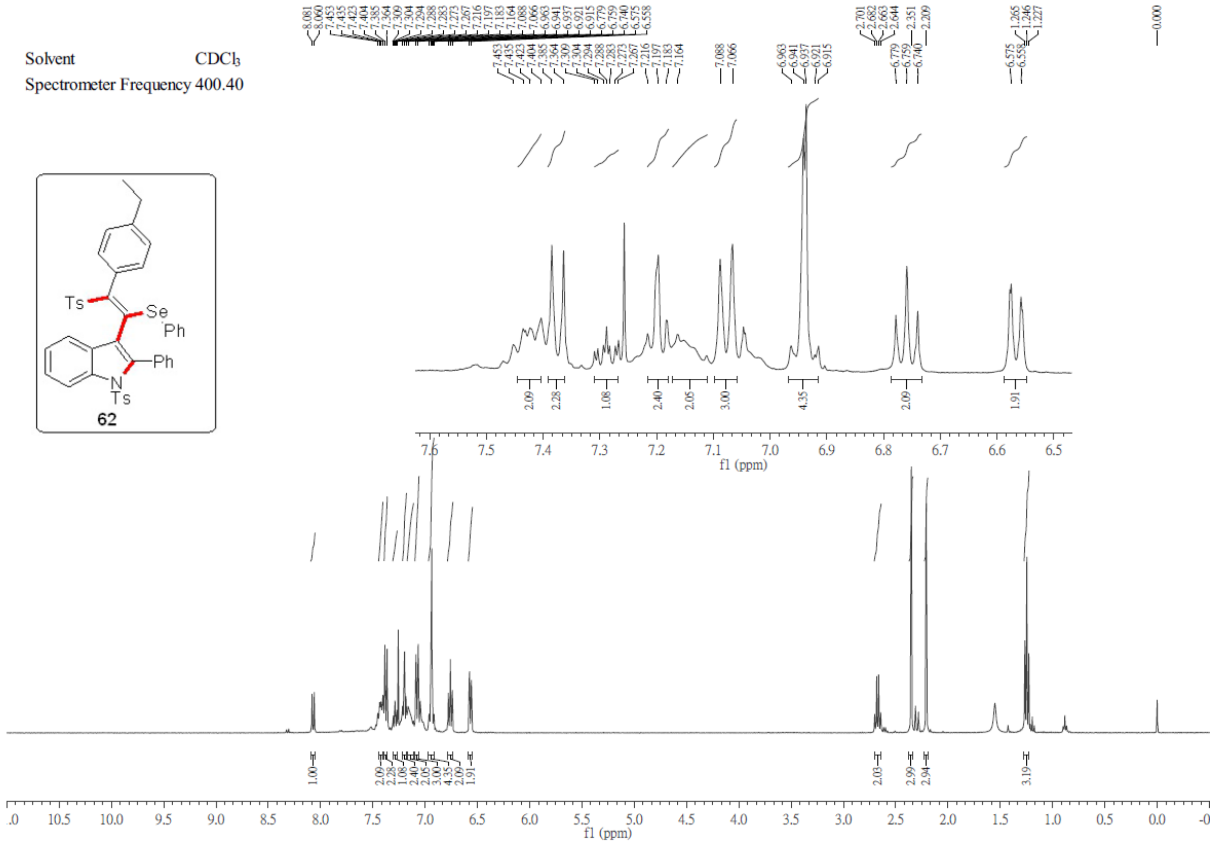

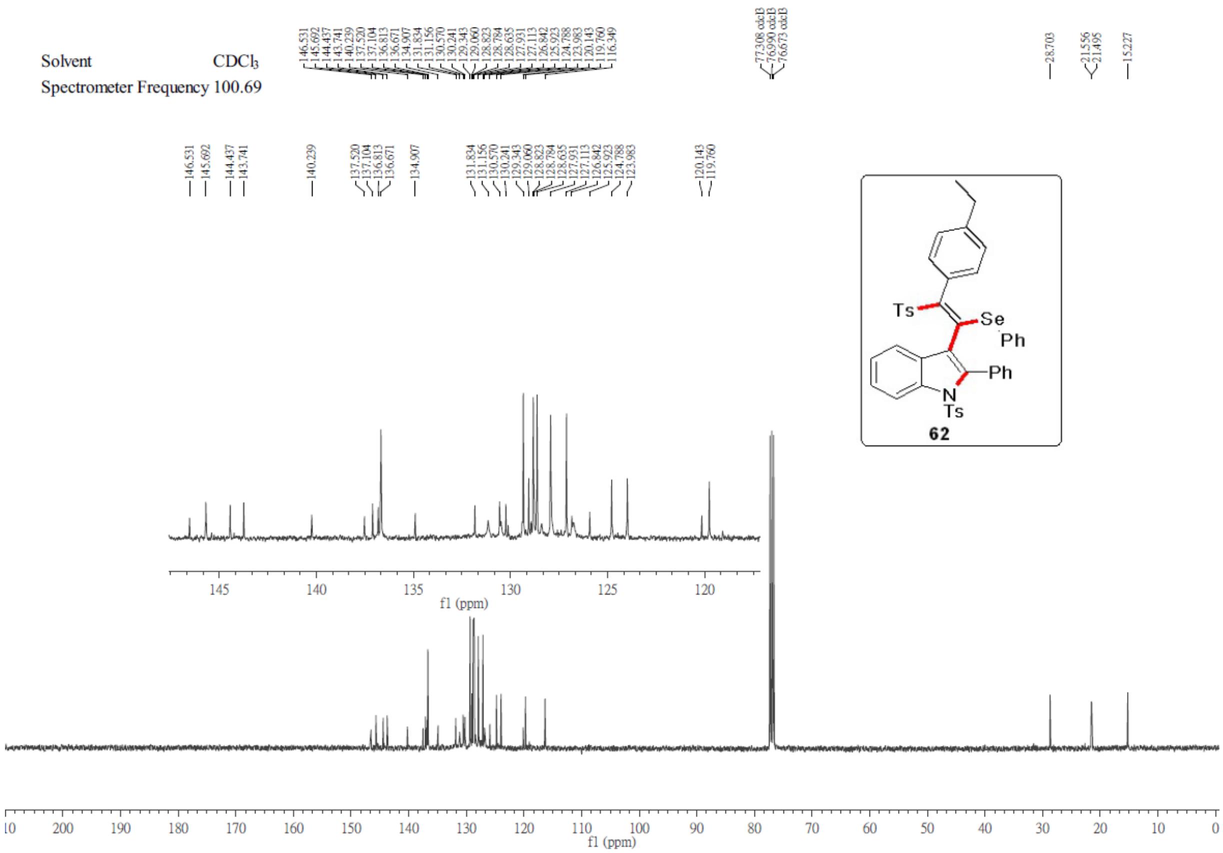

Supplementary Figure 149. ^{1}H (top) and ^{13}C (bottom) NMR spectra of compound **62**.

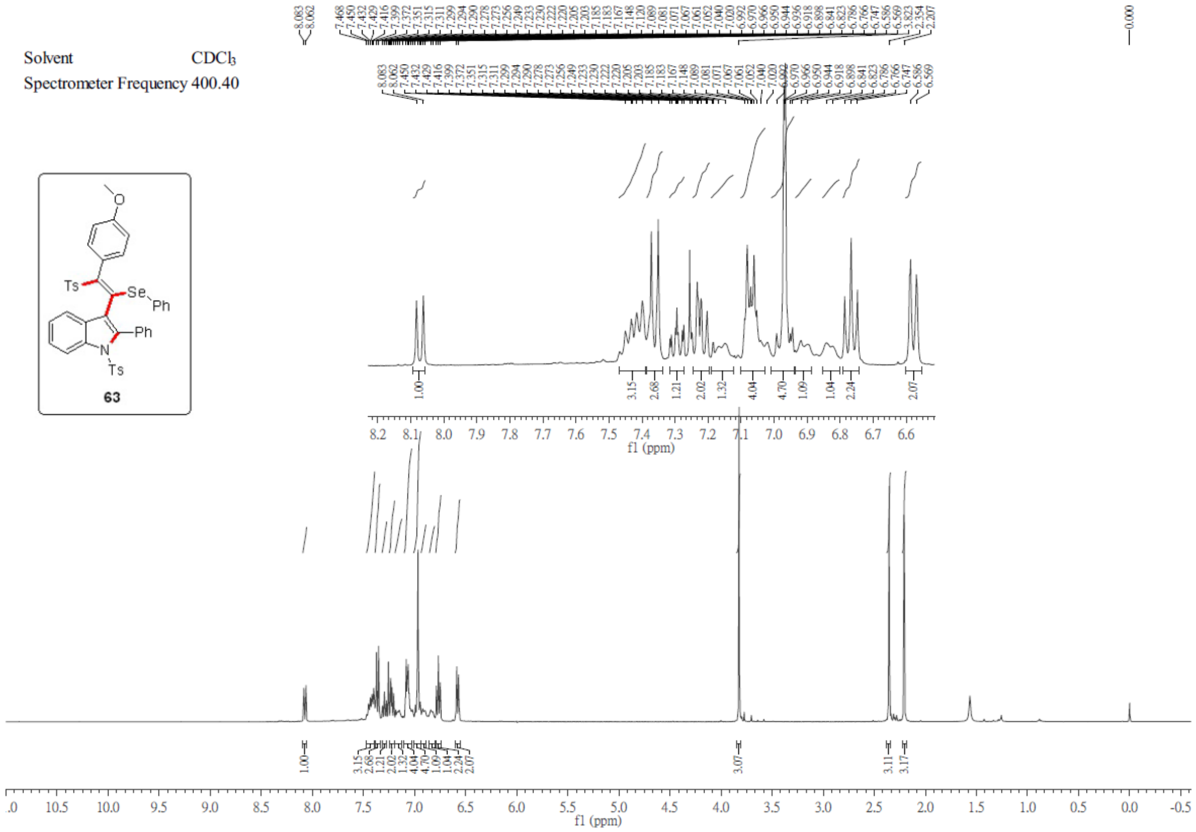

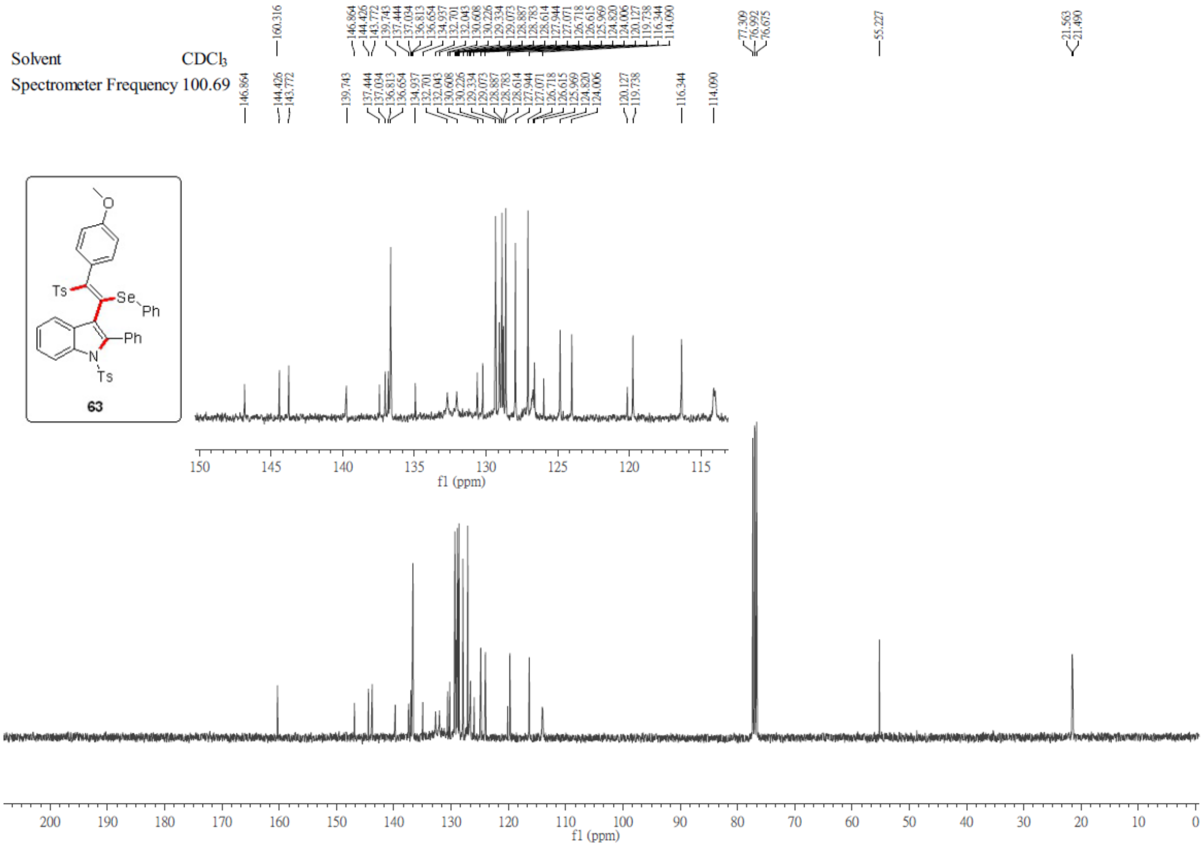

Supplementary Figure 150. ^{1}H (top) and ^{13}C (bottom) NMR spectra of compound **63**.

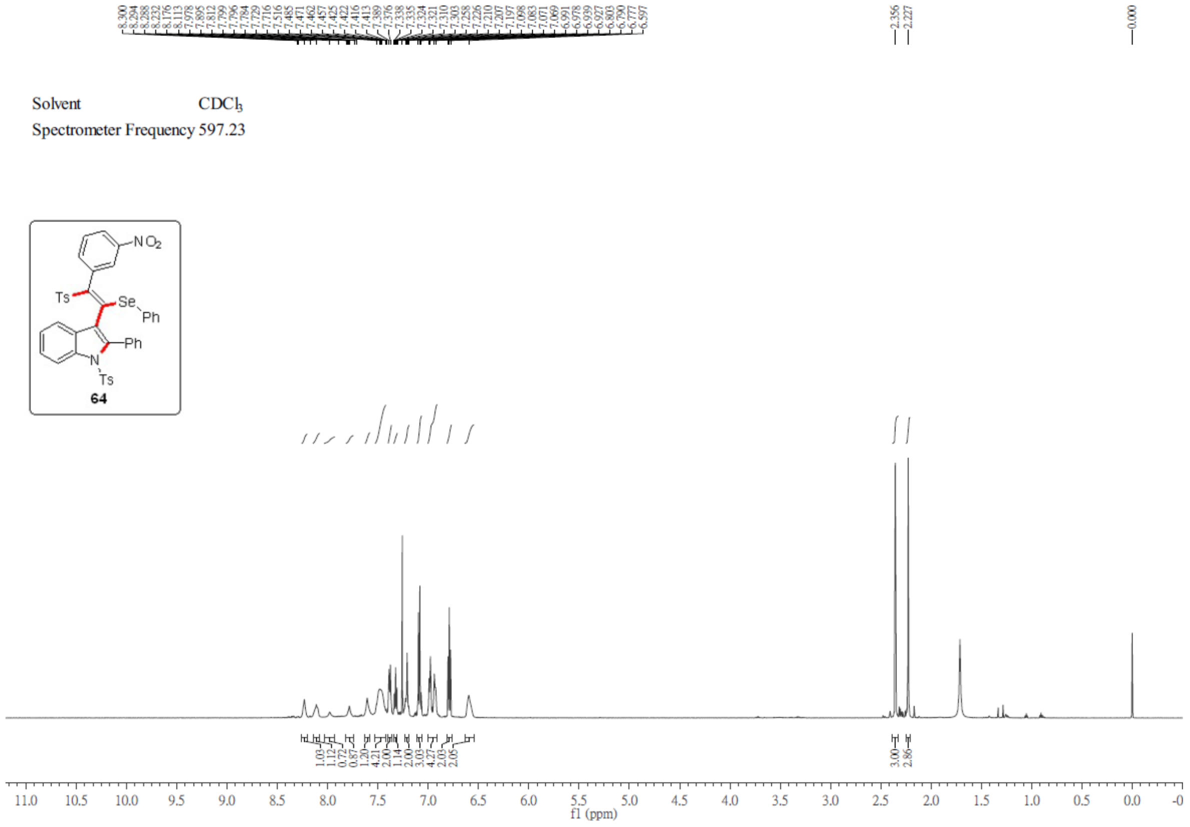

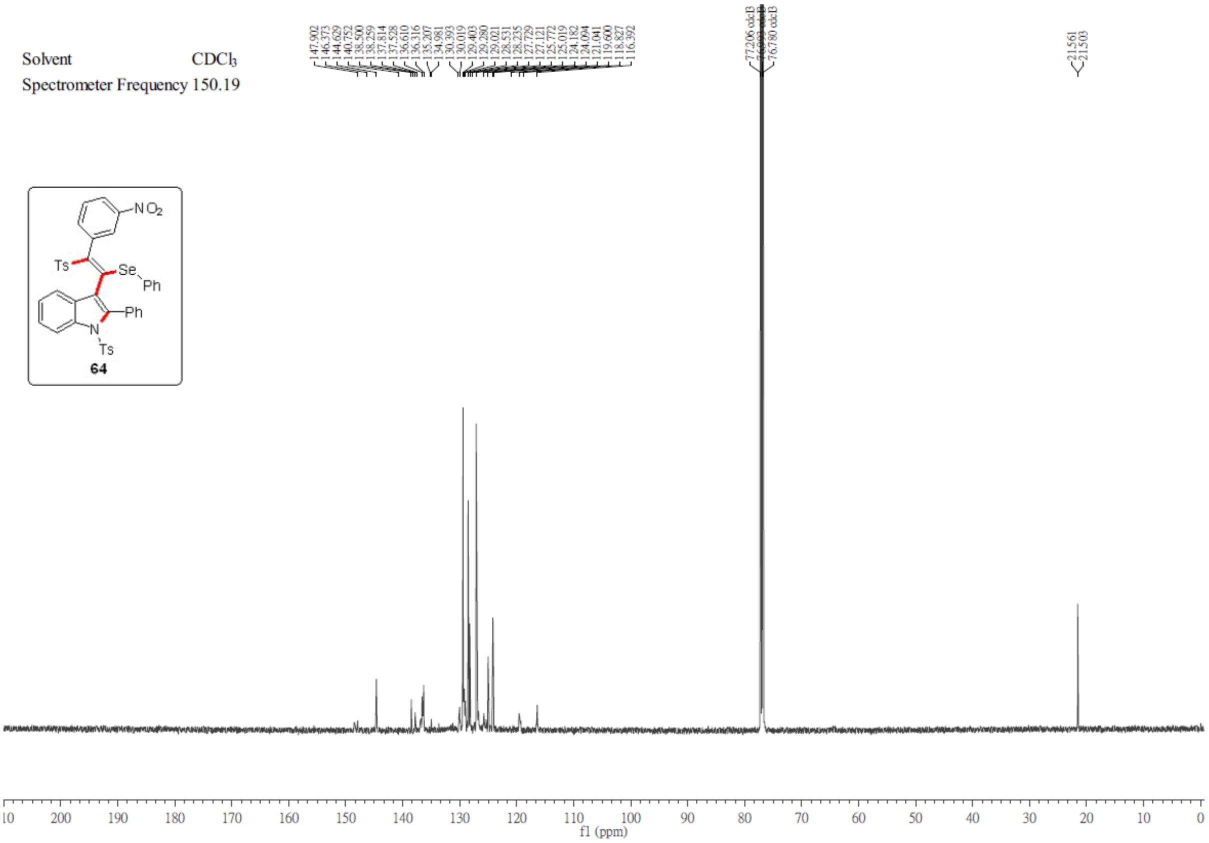

Supplementary Figure 151. ^{1}H (top) and ^{13}C (bottom) NMR spectra of compound **64**.

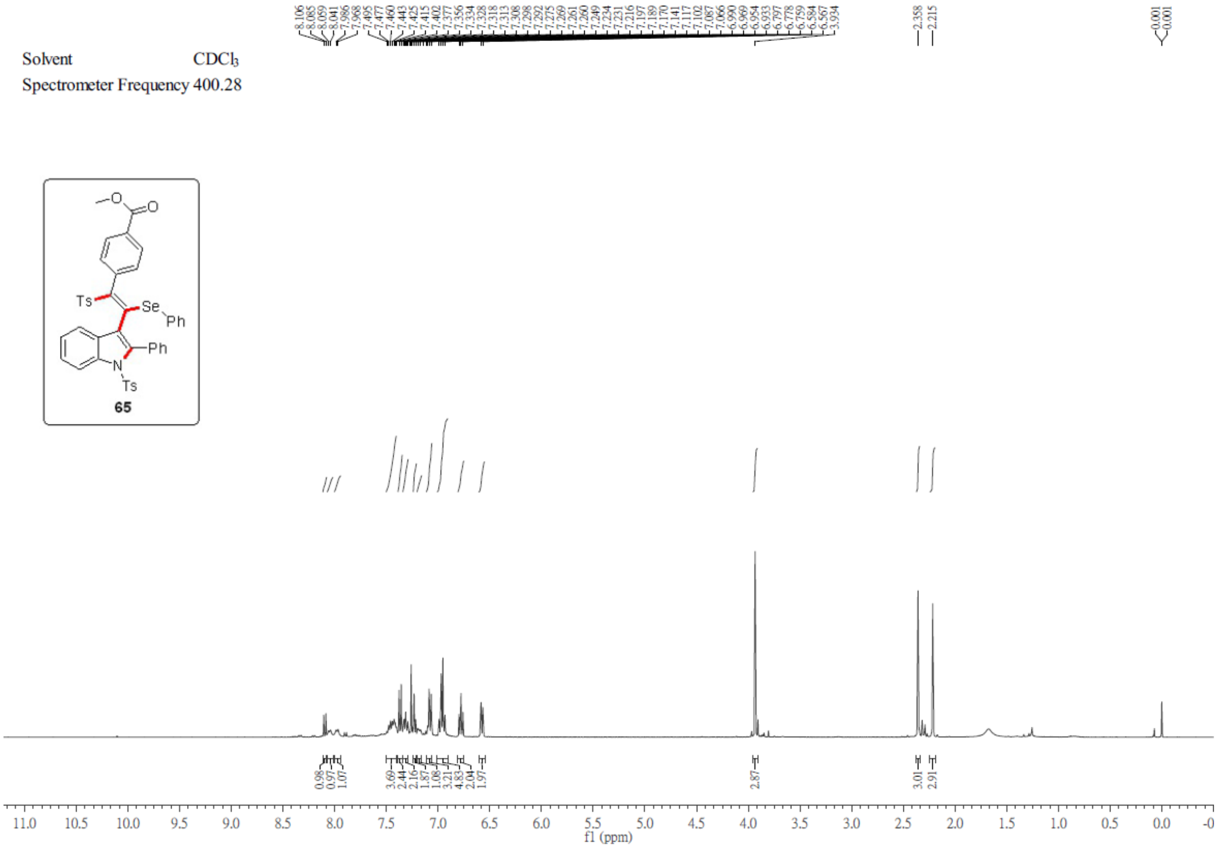

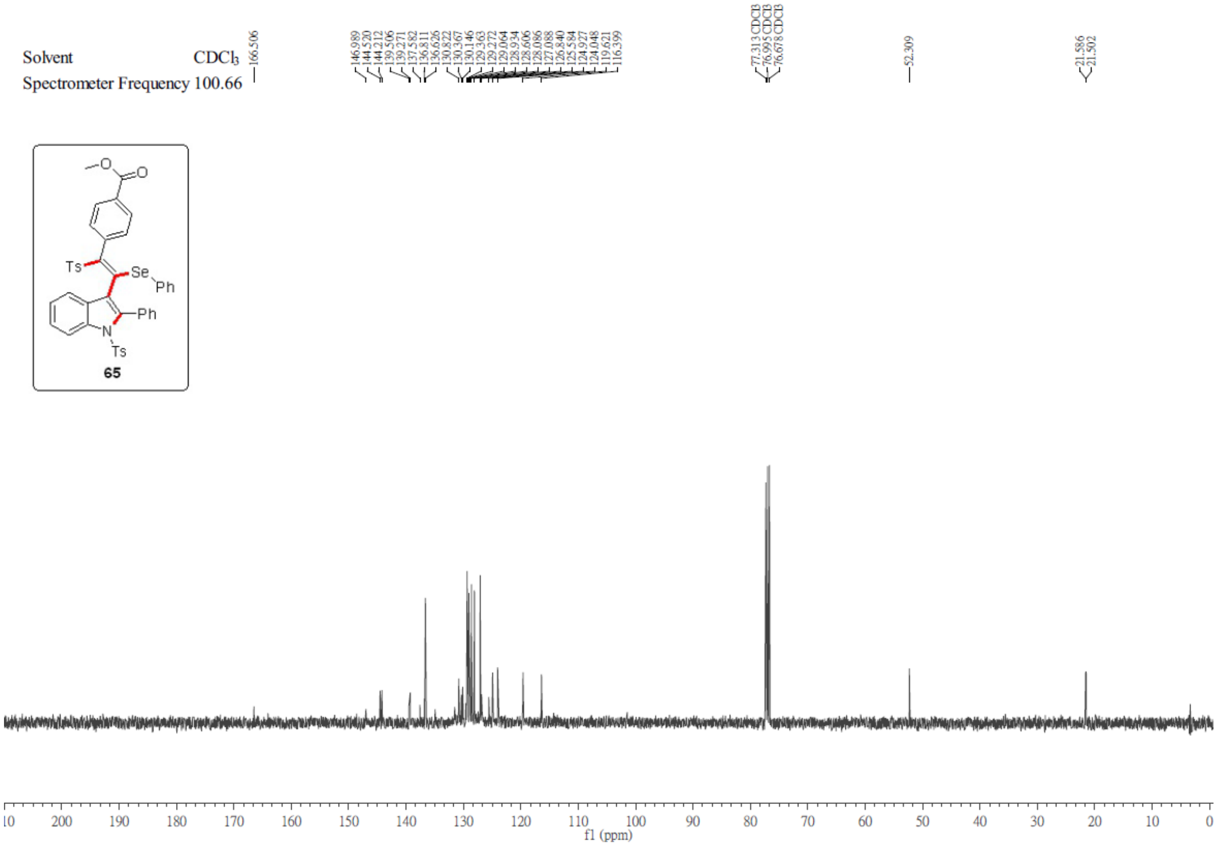

Supplementary Figure 152. ^{1}H (top) and ^{13}C (bottom) NMR spectra of compound **65**.

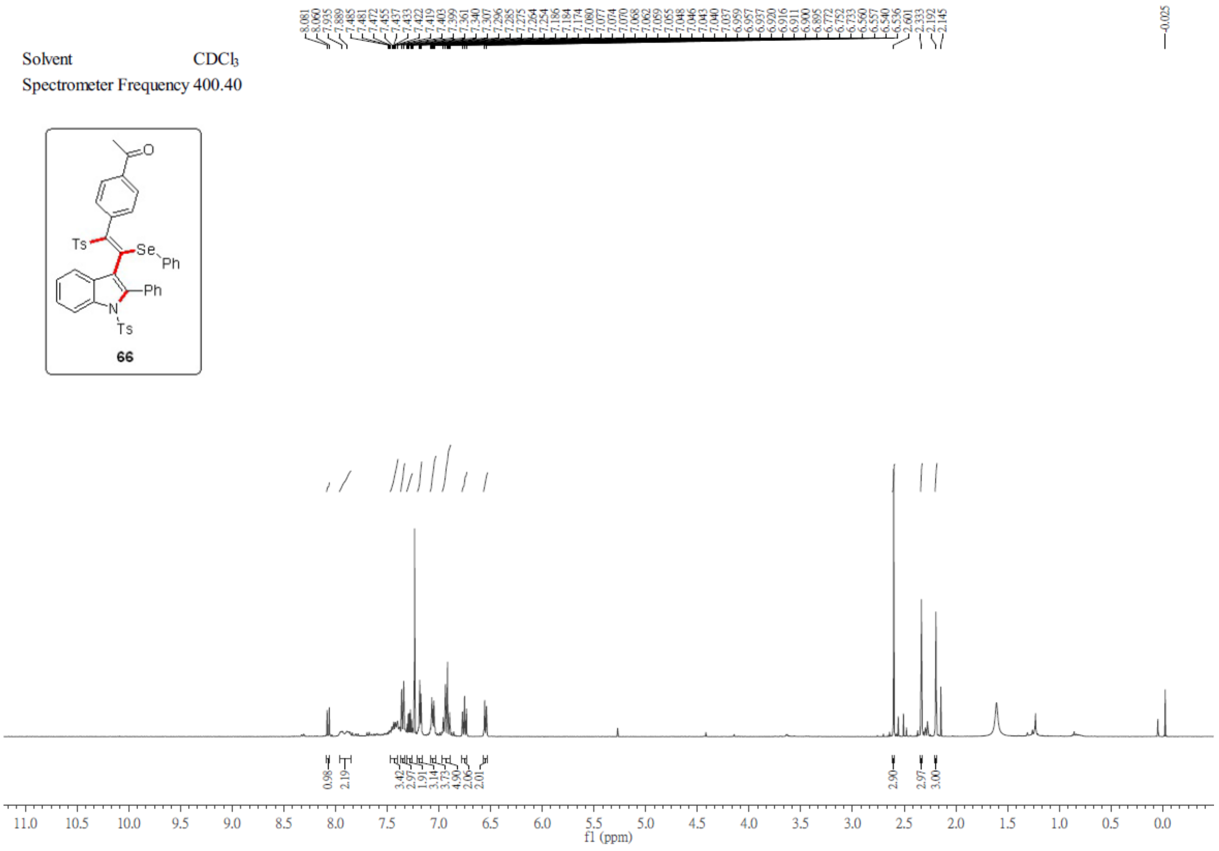

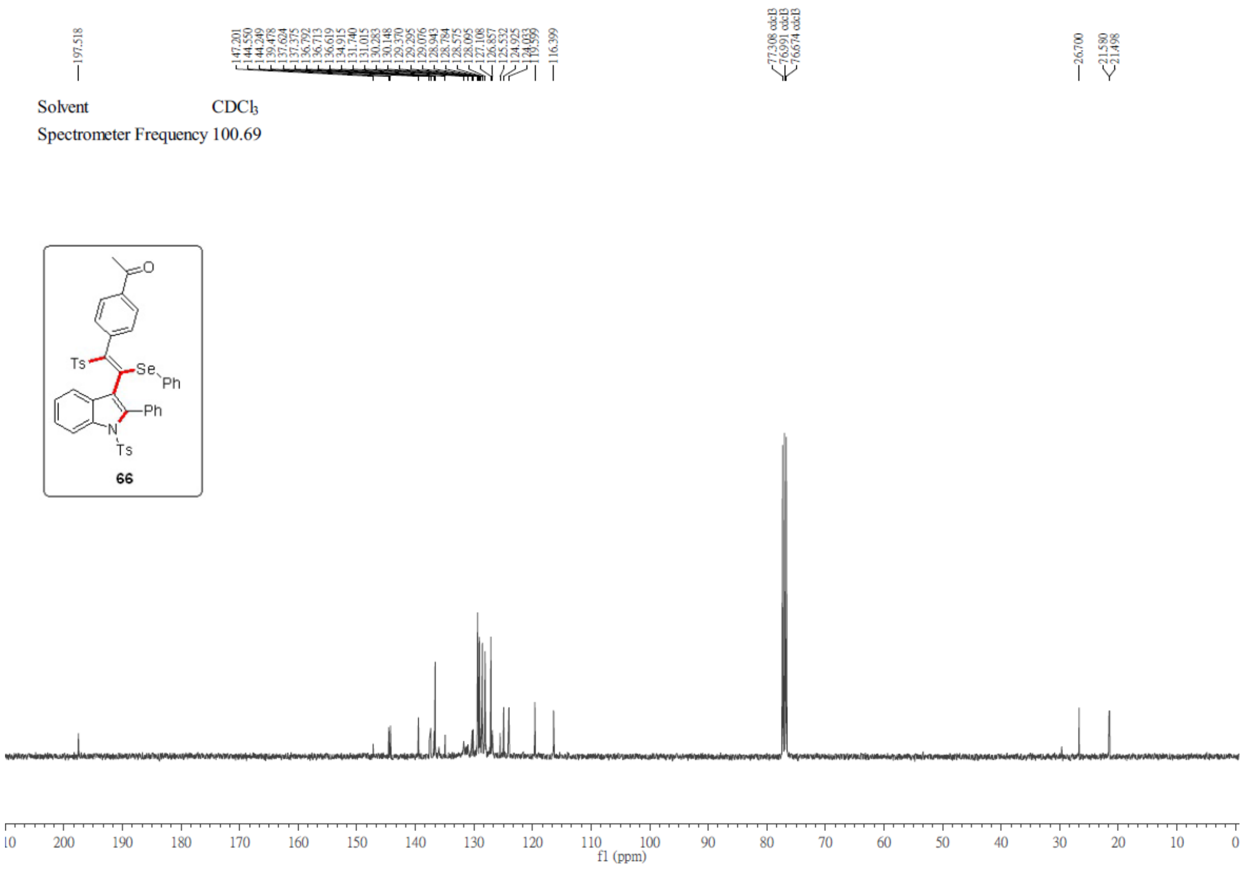

Supplementary Figure 153. ^{1}H (top) and ^{13}C (bottom) NMR spectra of compound **66**.

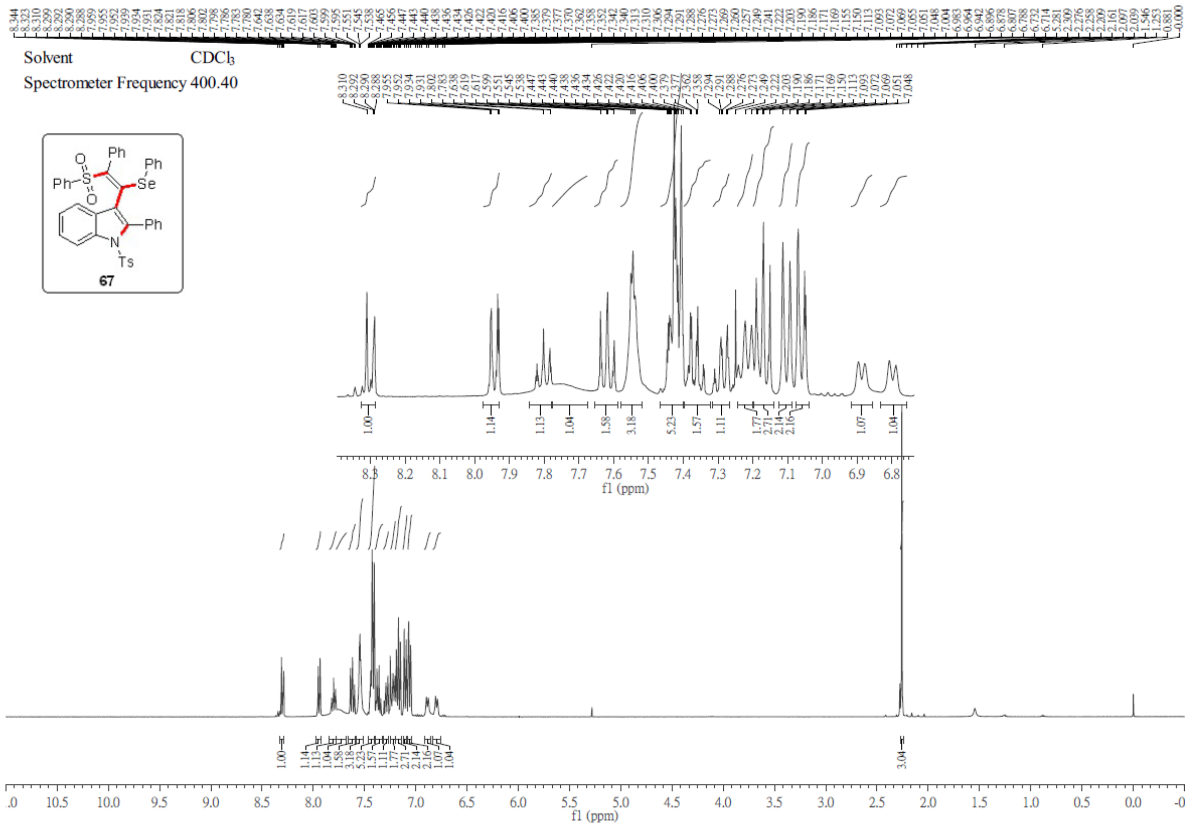

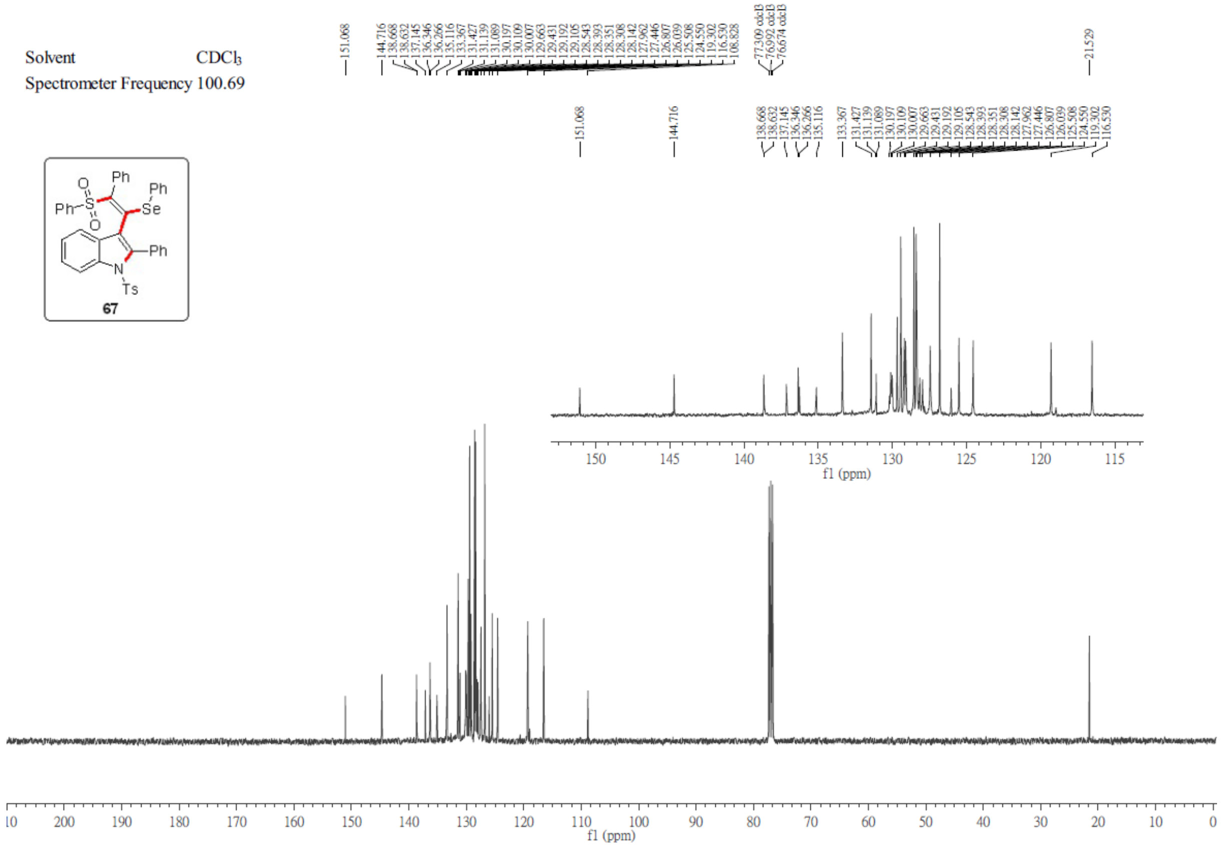

Supplementary Figure 154. ^{1}H (top) and ^{13}C (bottom) NMR spectra of compound **67**.

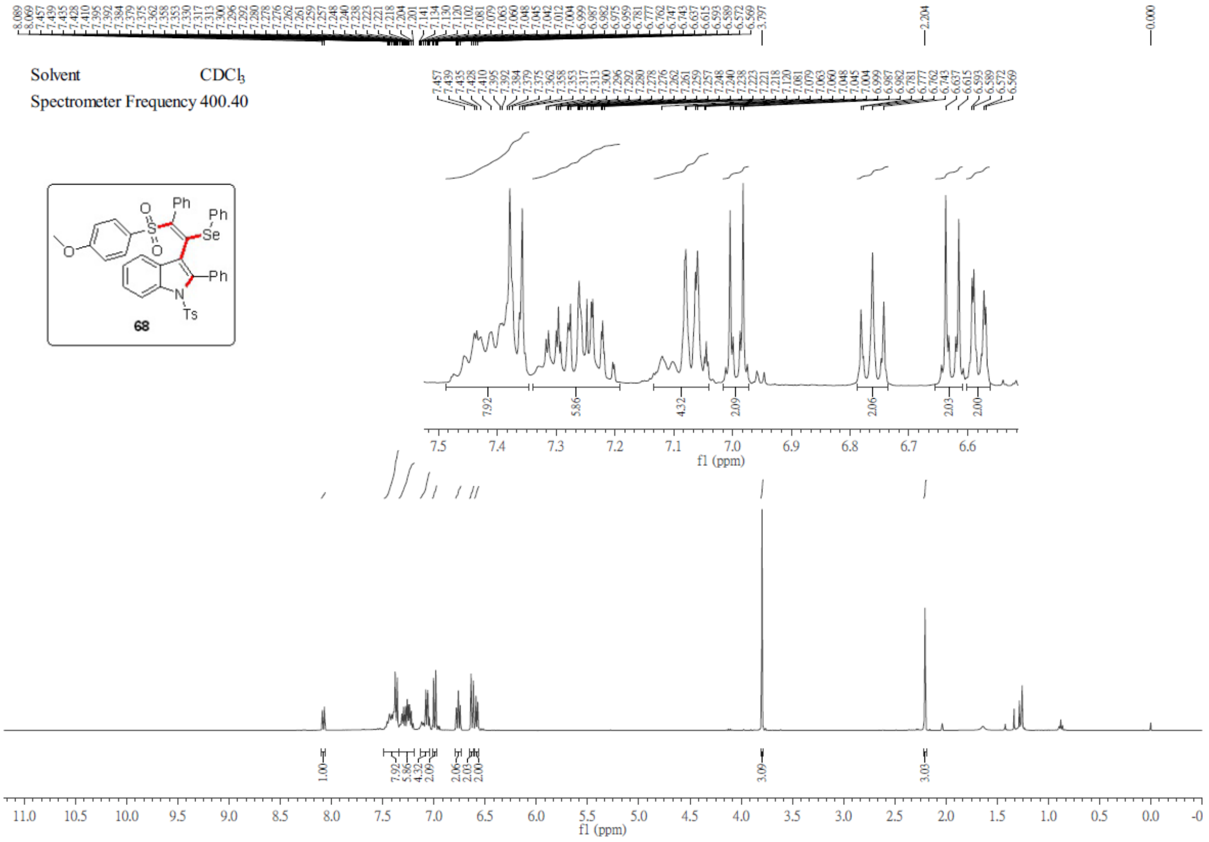

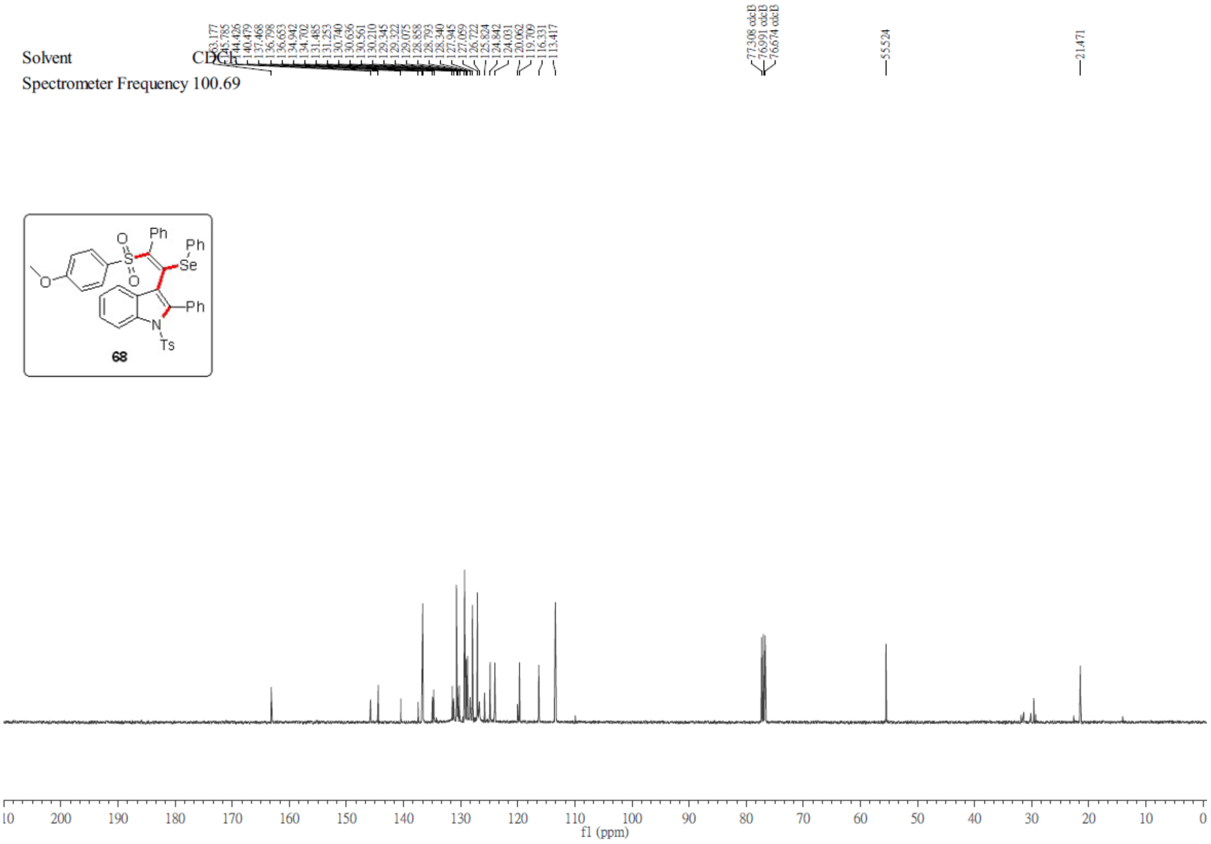

Supplementary Figure 155. ^{1}H (top) and ^{13}C (bottom) NMR spectra of compound **68**.

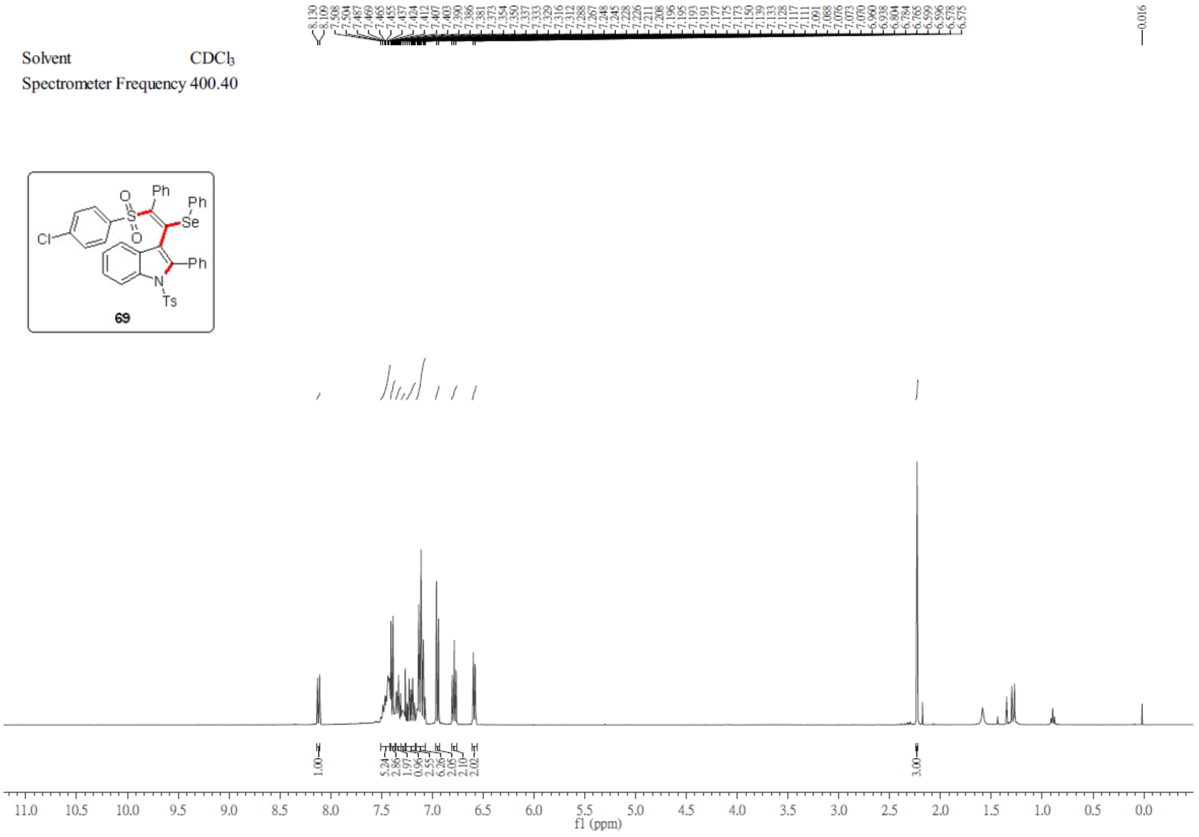

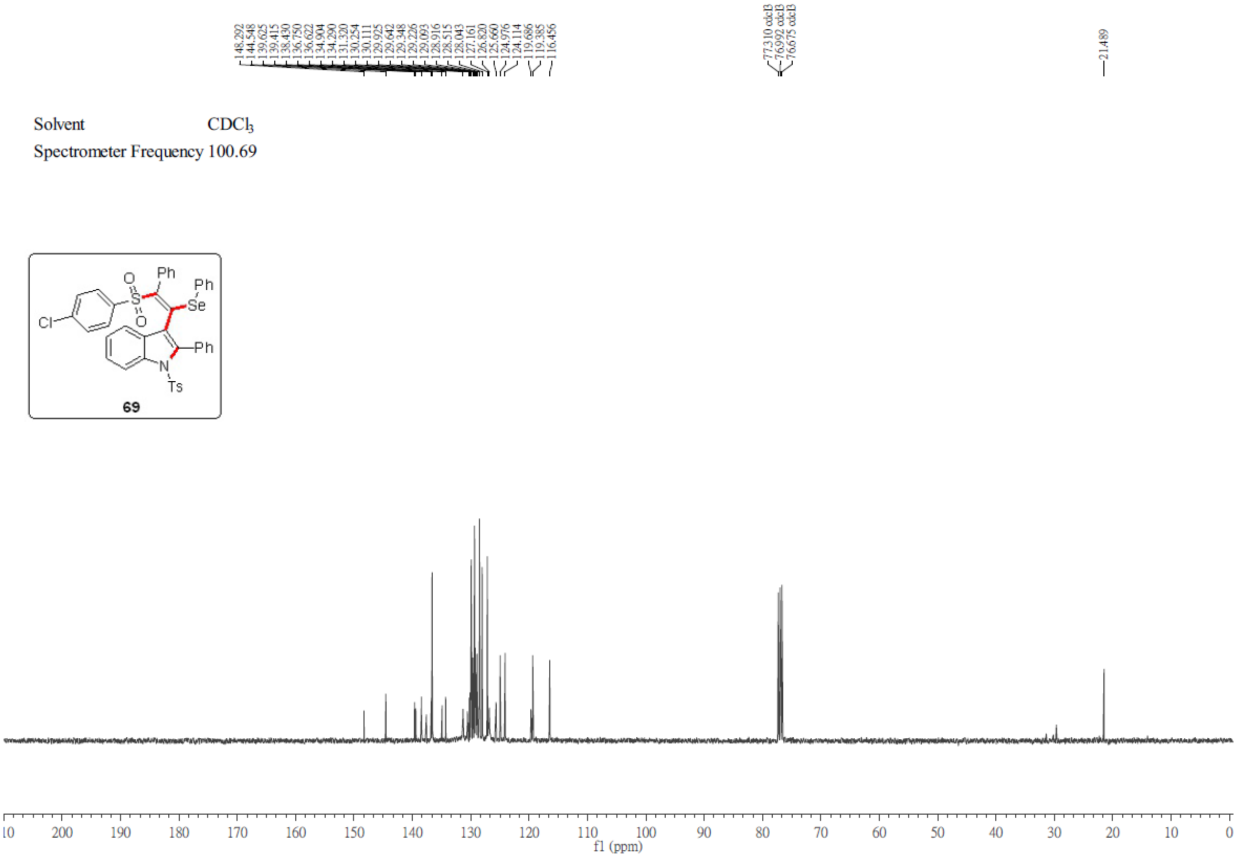

Supplementary Figure 156. ^{1}H (top) and ^{13}C (bottom) NMR spectra of compound **69**.

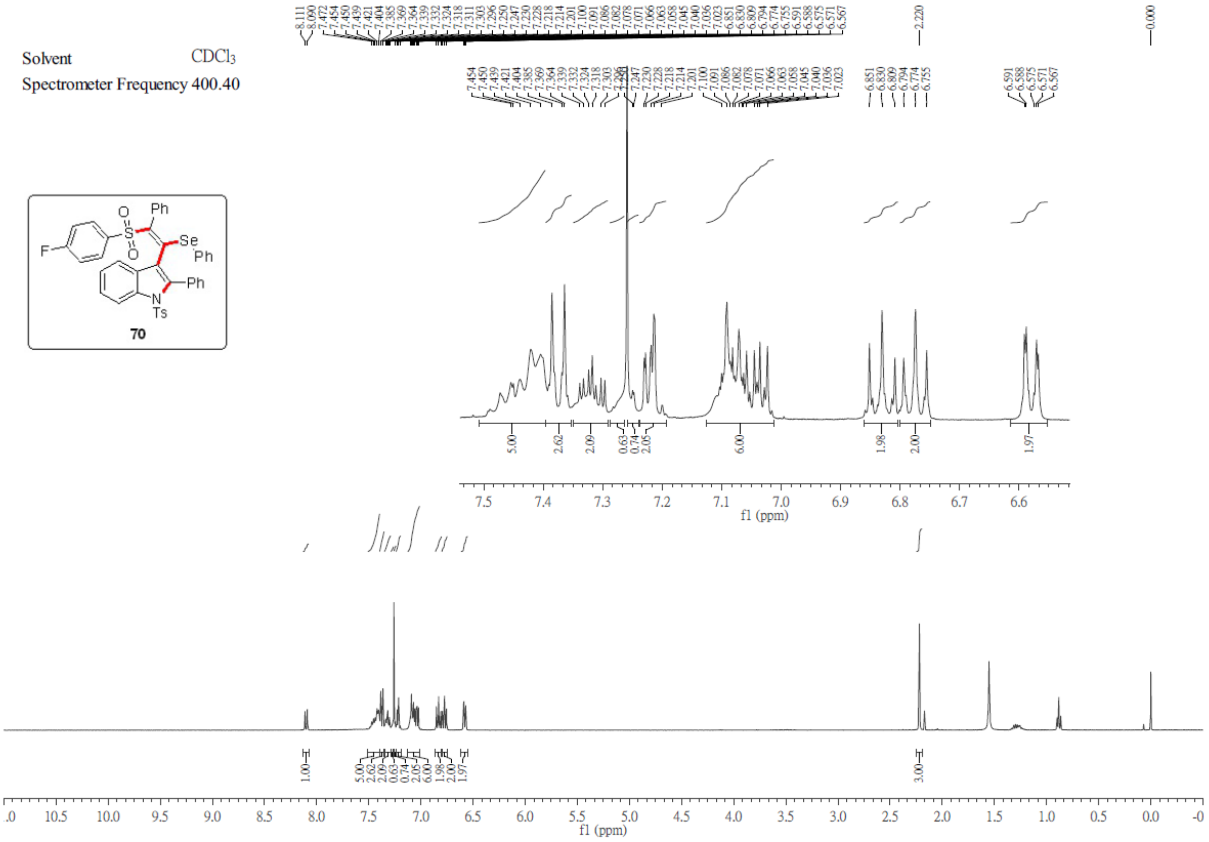

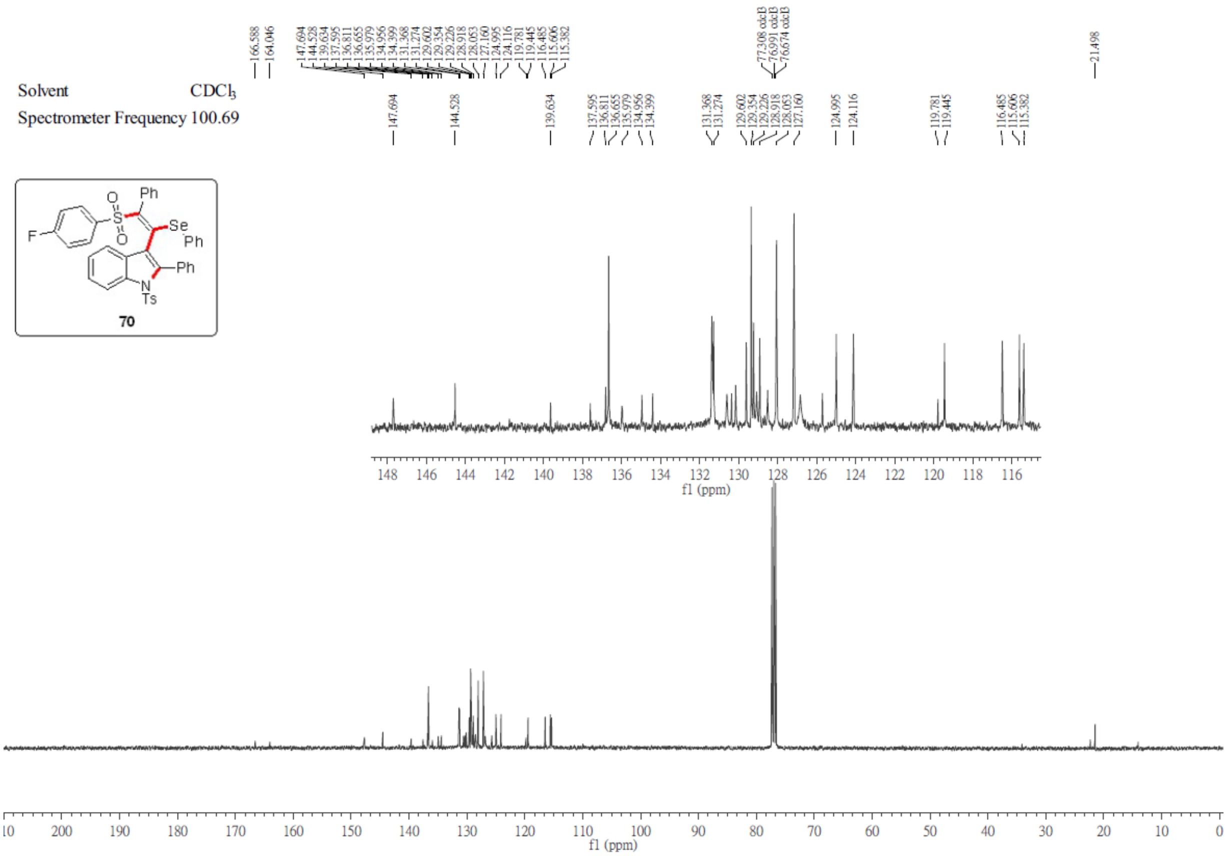

Supplementary Figure 157. ^{1}H (top) and ^{13}C (bottom) NMR spectra of compound **70**.

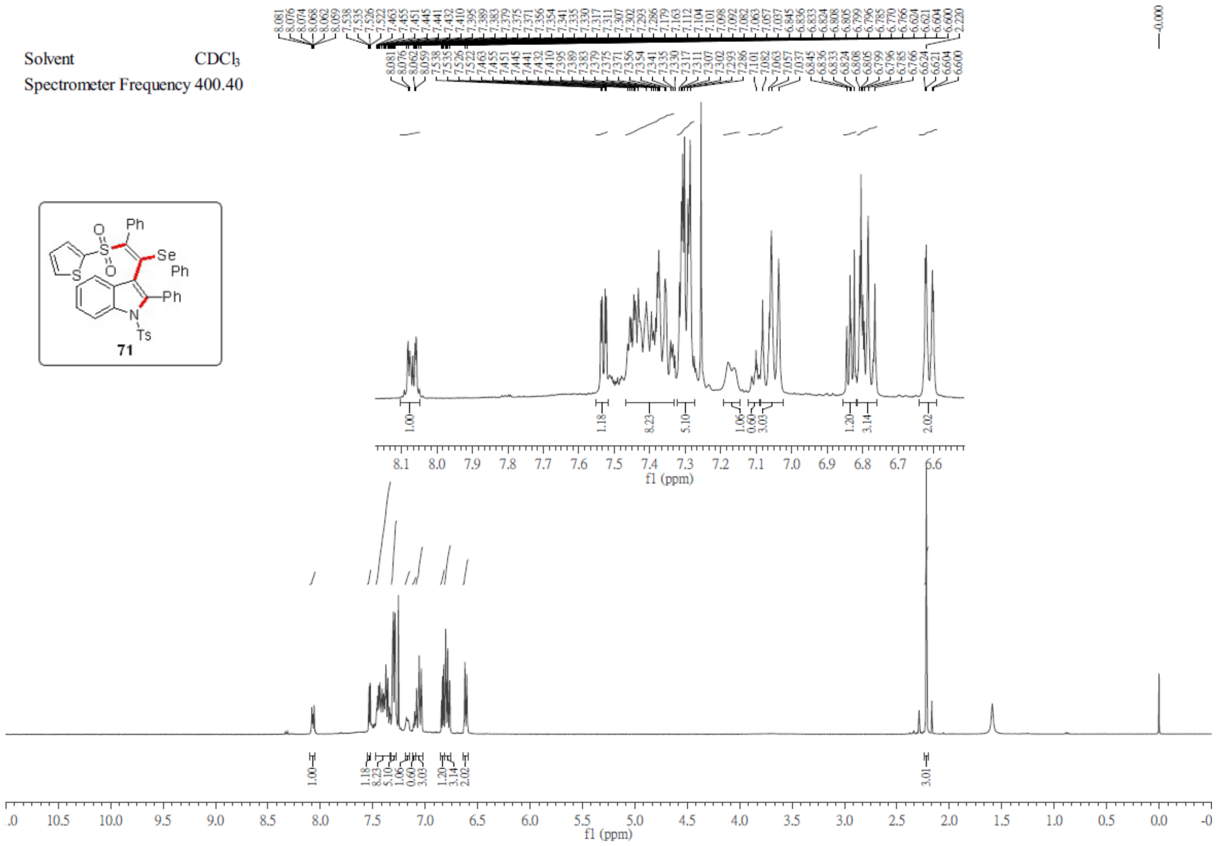

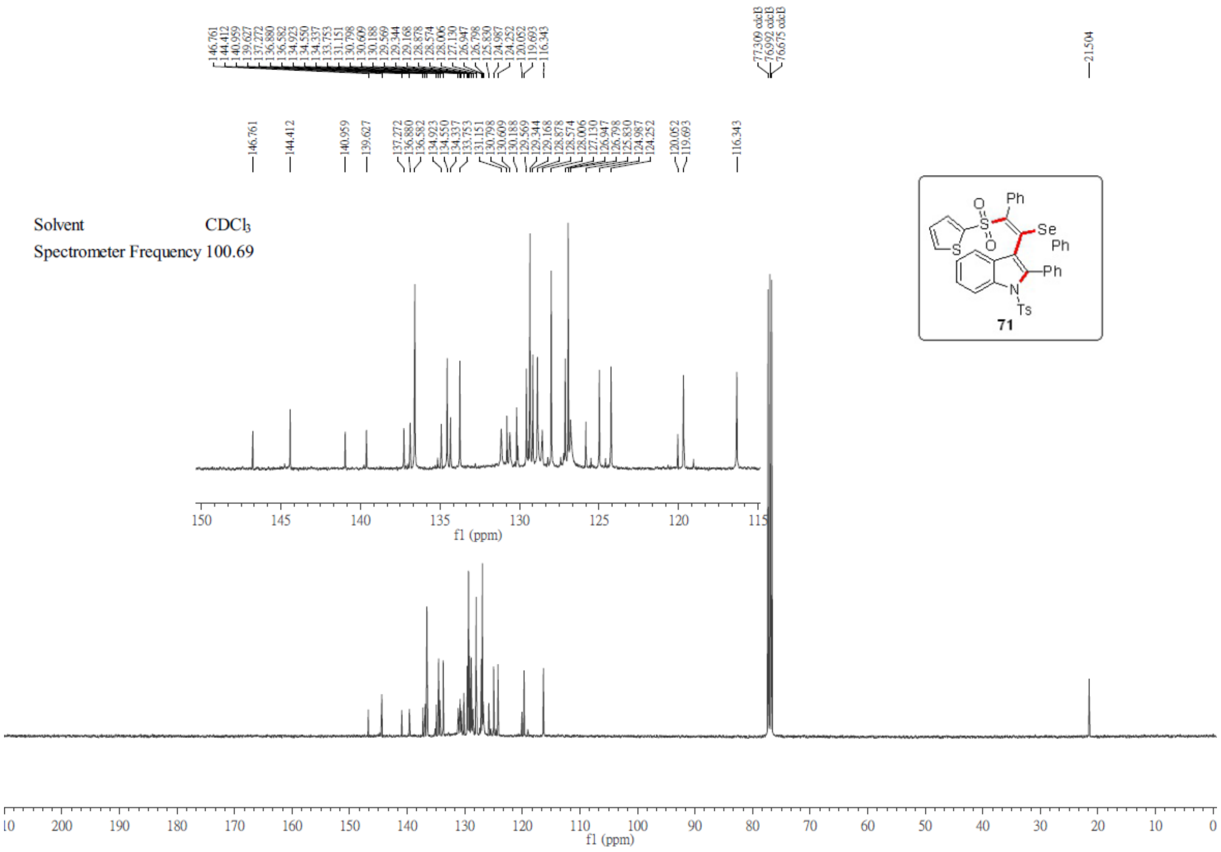

Supplementary Figure 158. ^{1}H (top) and ^{13}C (bottom) NMR spectra of compound **71**.

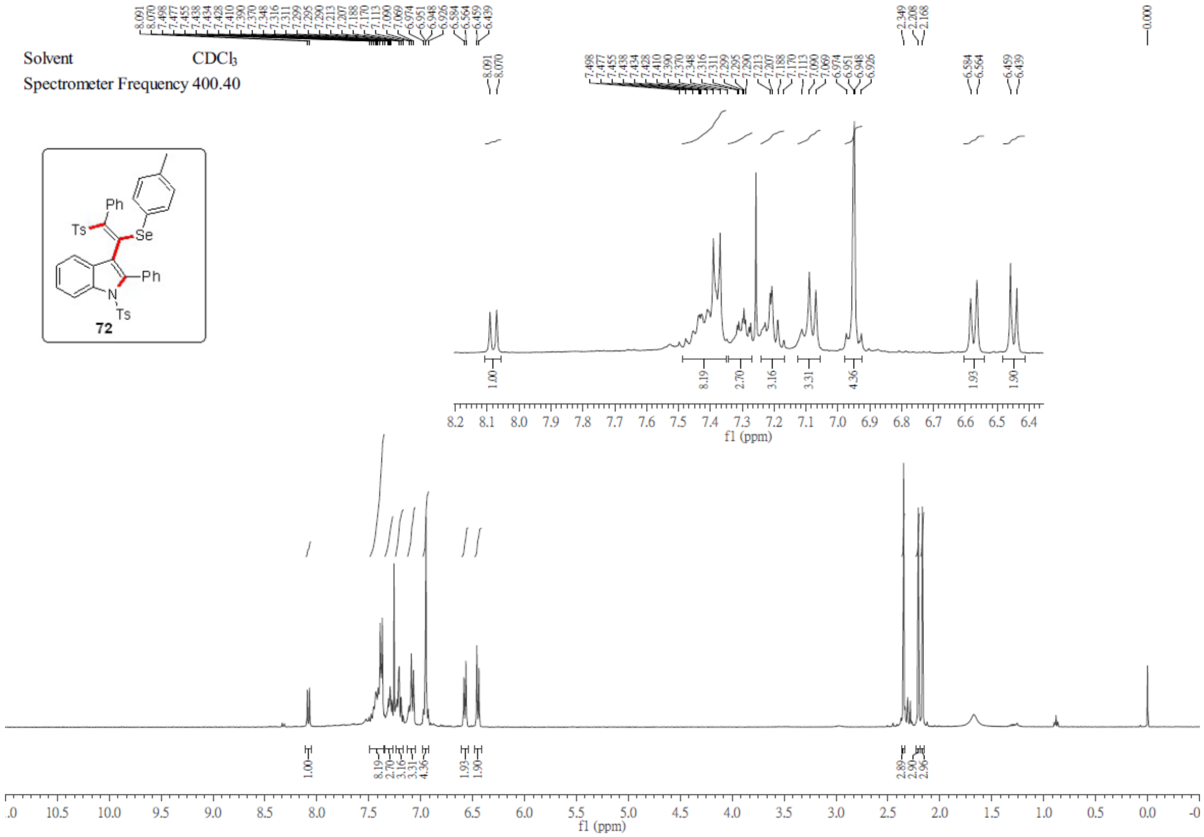

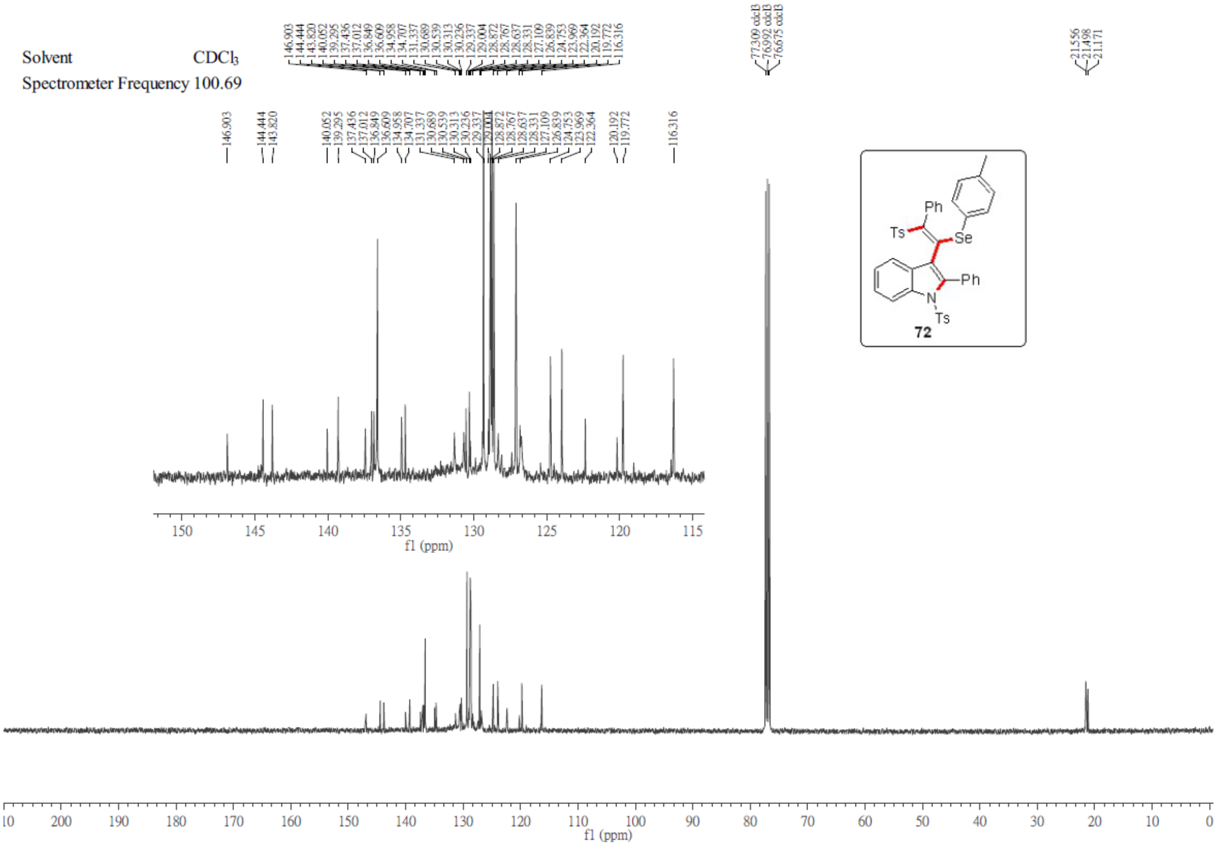

Supplementary Figure 159. ^{1}H (top) and ^{13}C (bottom) NMR spectra of compound **72**.

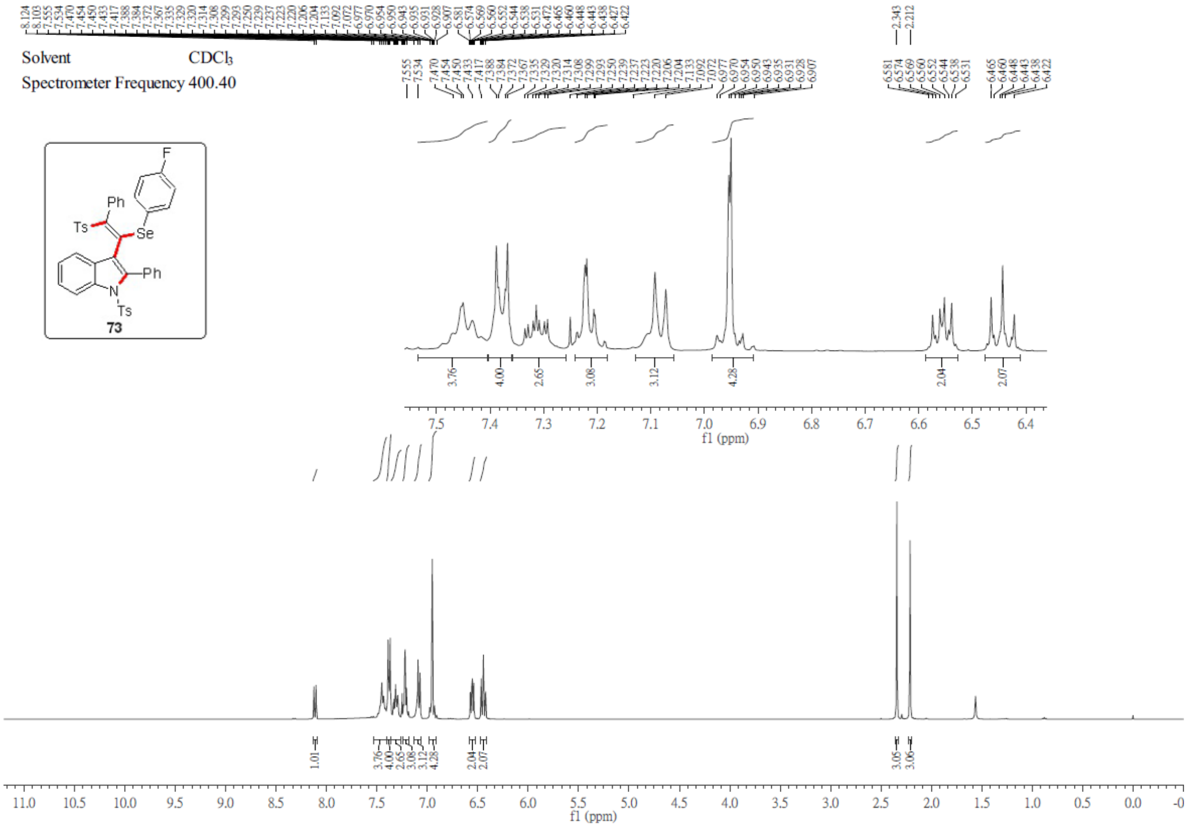

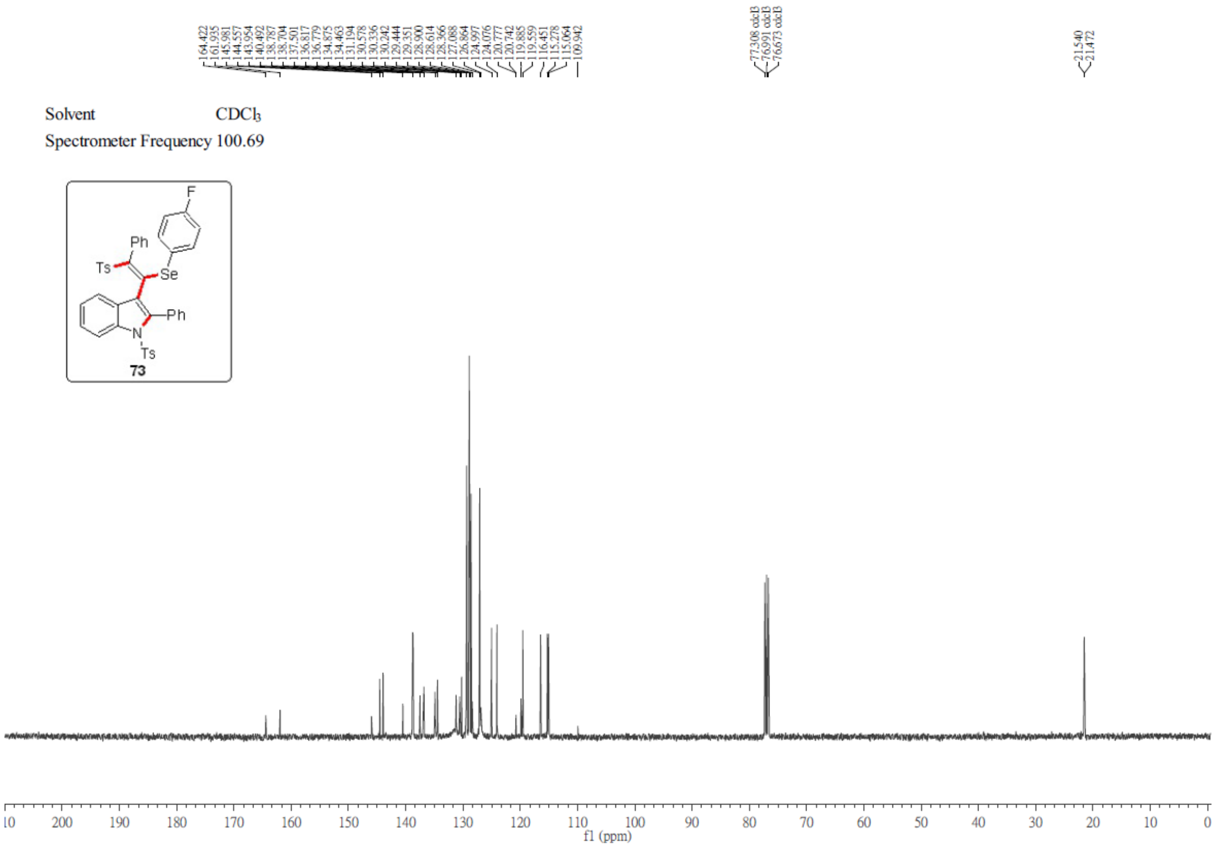

Supplementary Figure 160. ^{1}H (top) and ^{13}C (bottom) NMR spectra of compound **73**.

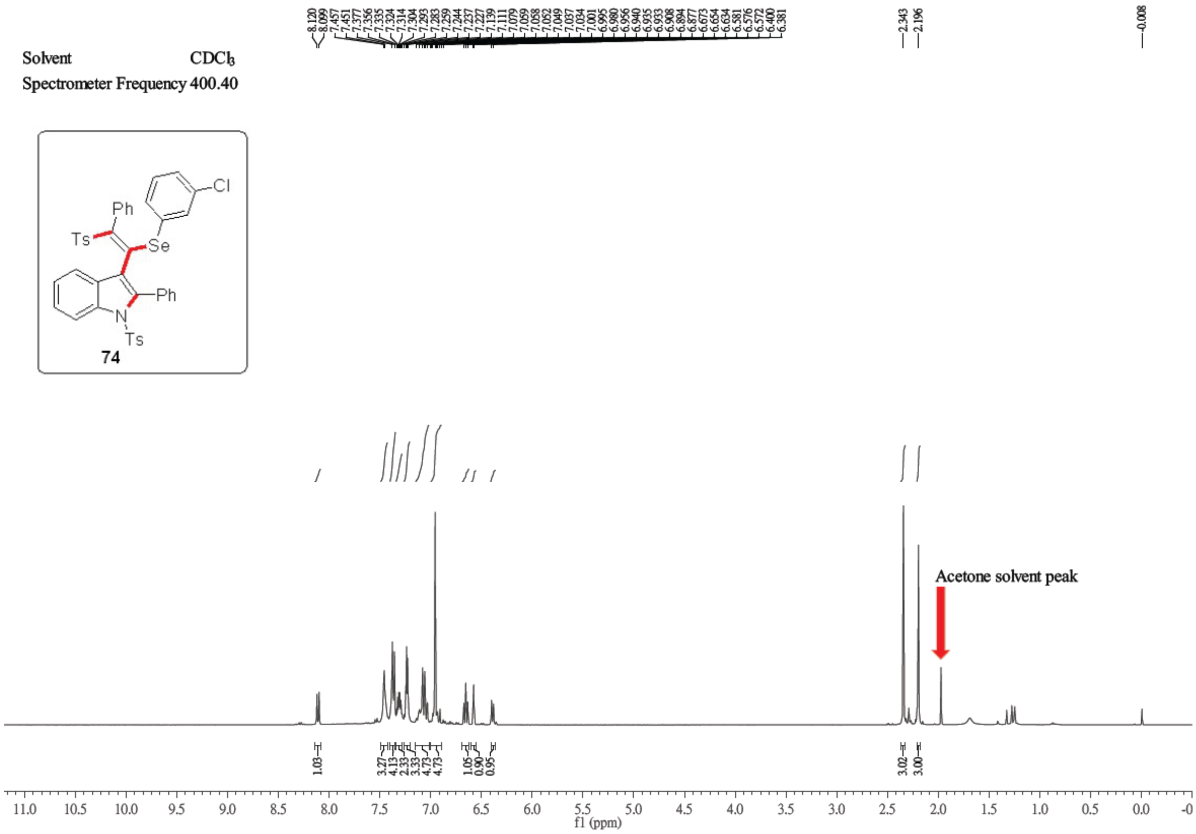

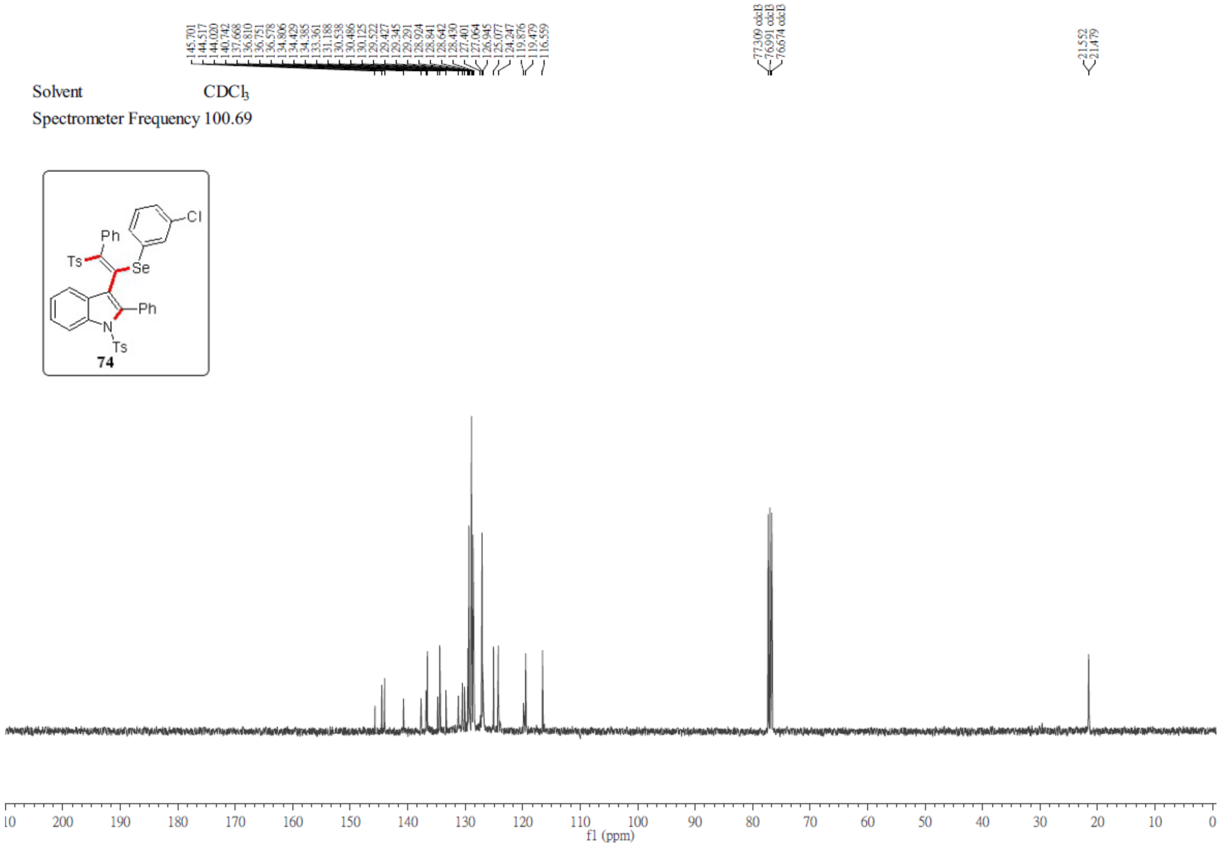

Supplementary Figure 161. ^{1}H (top) and ^{13}C (bottom) NMR spectra of compound **74**.

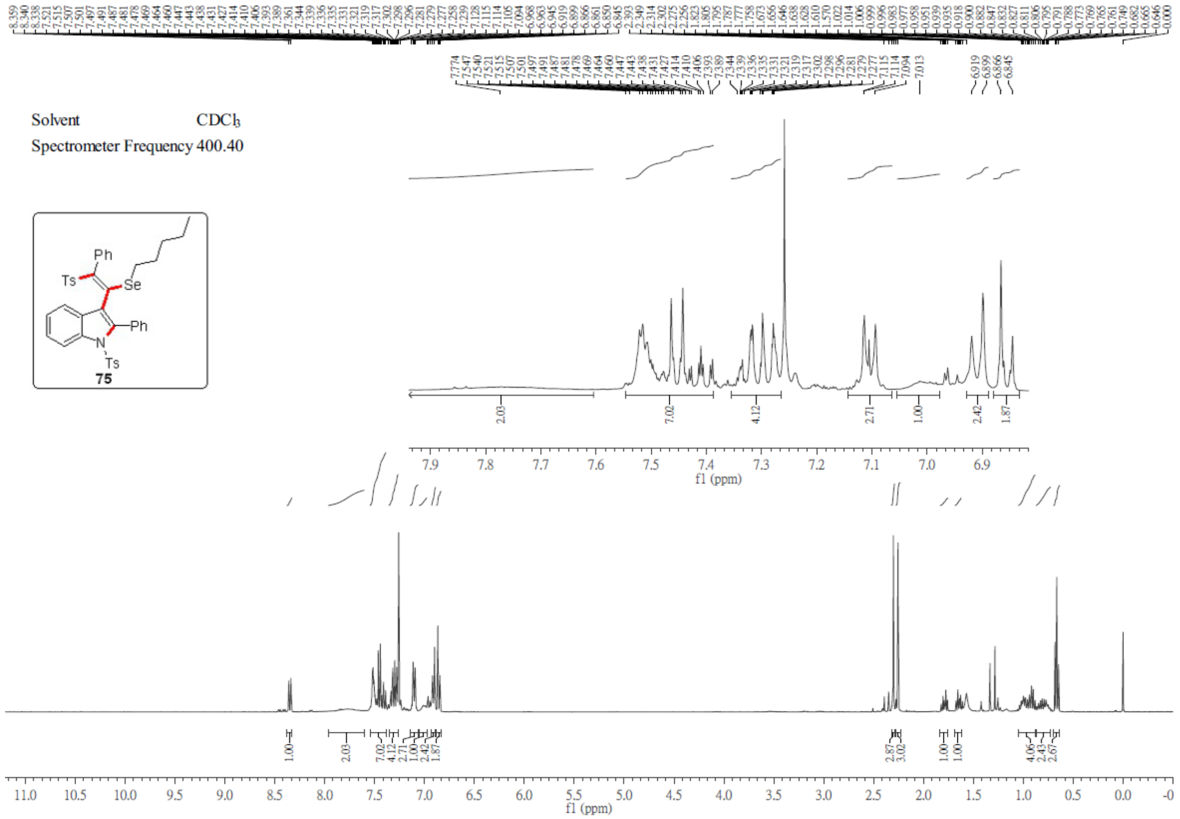

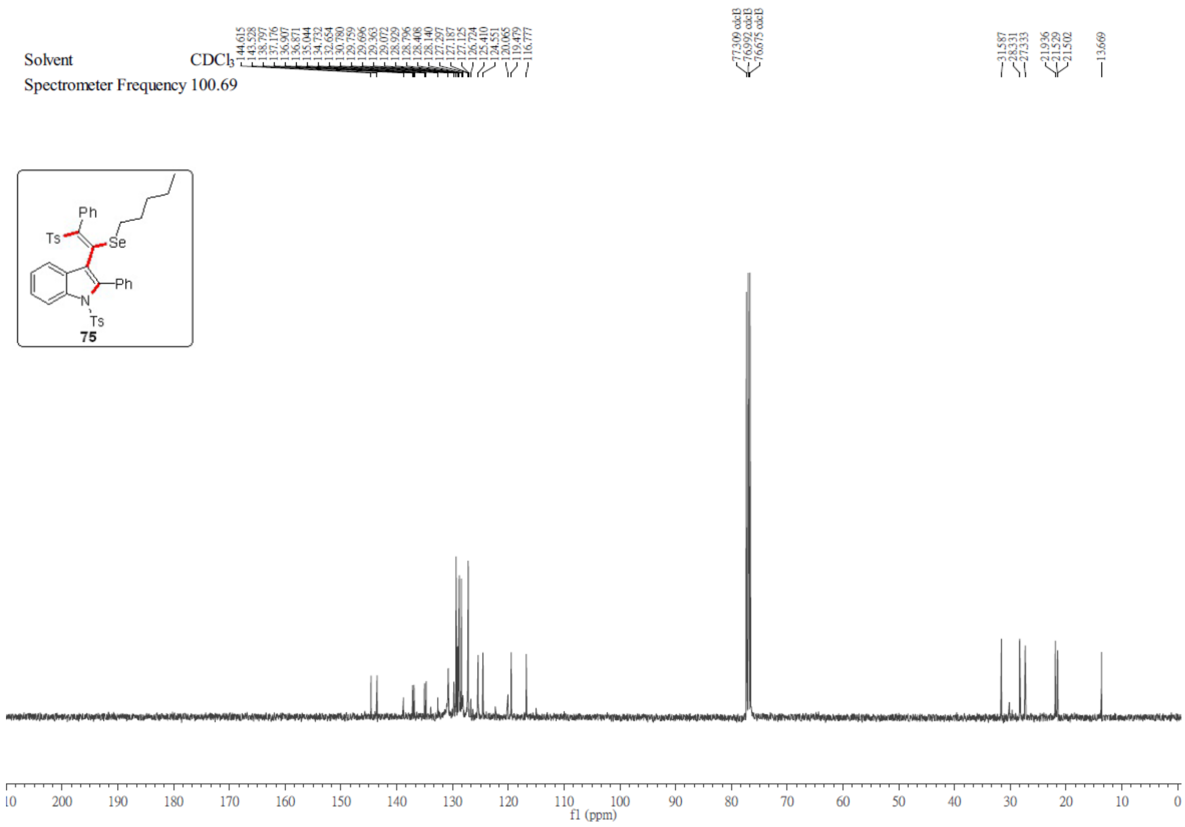

Supplementary Figure 162. ^{1}H (top) and ^{13}C (bottom) NMR spectra of compound **75**.

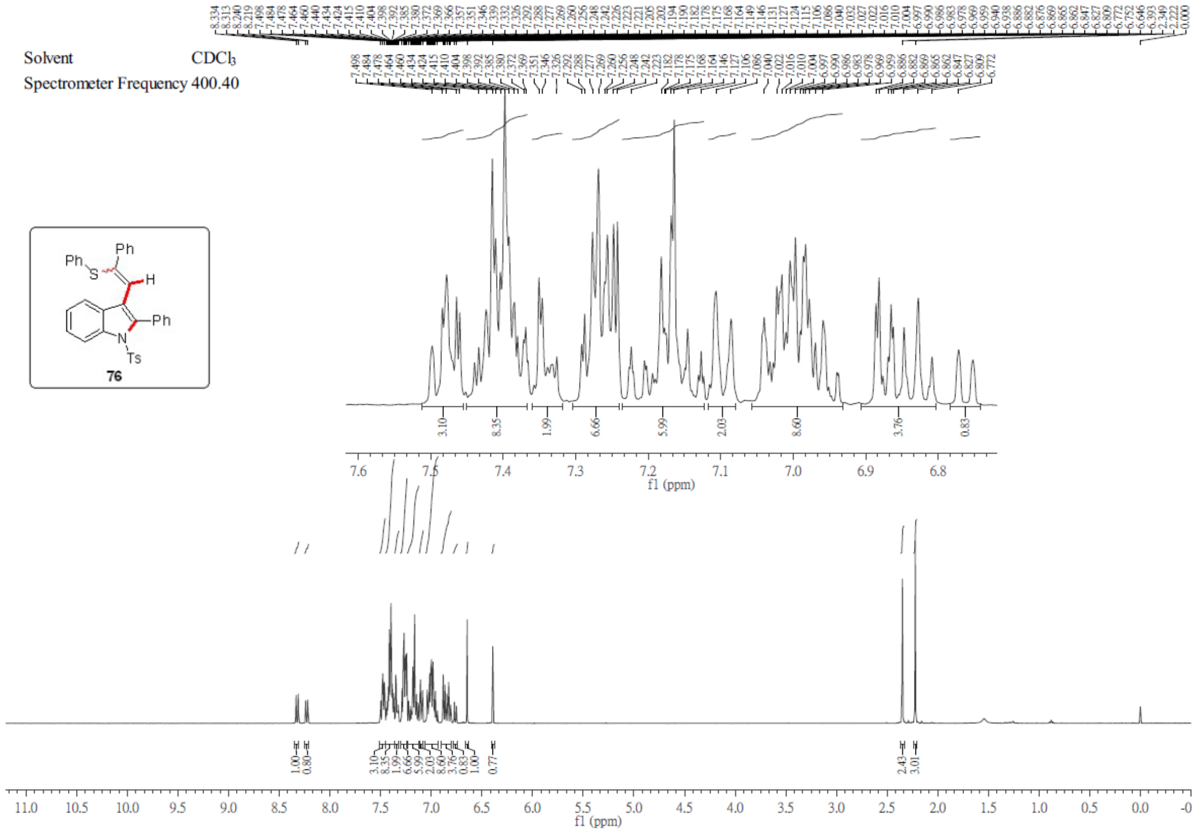

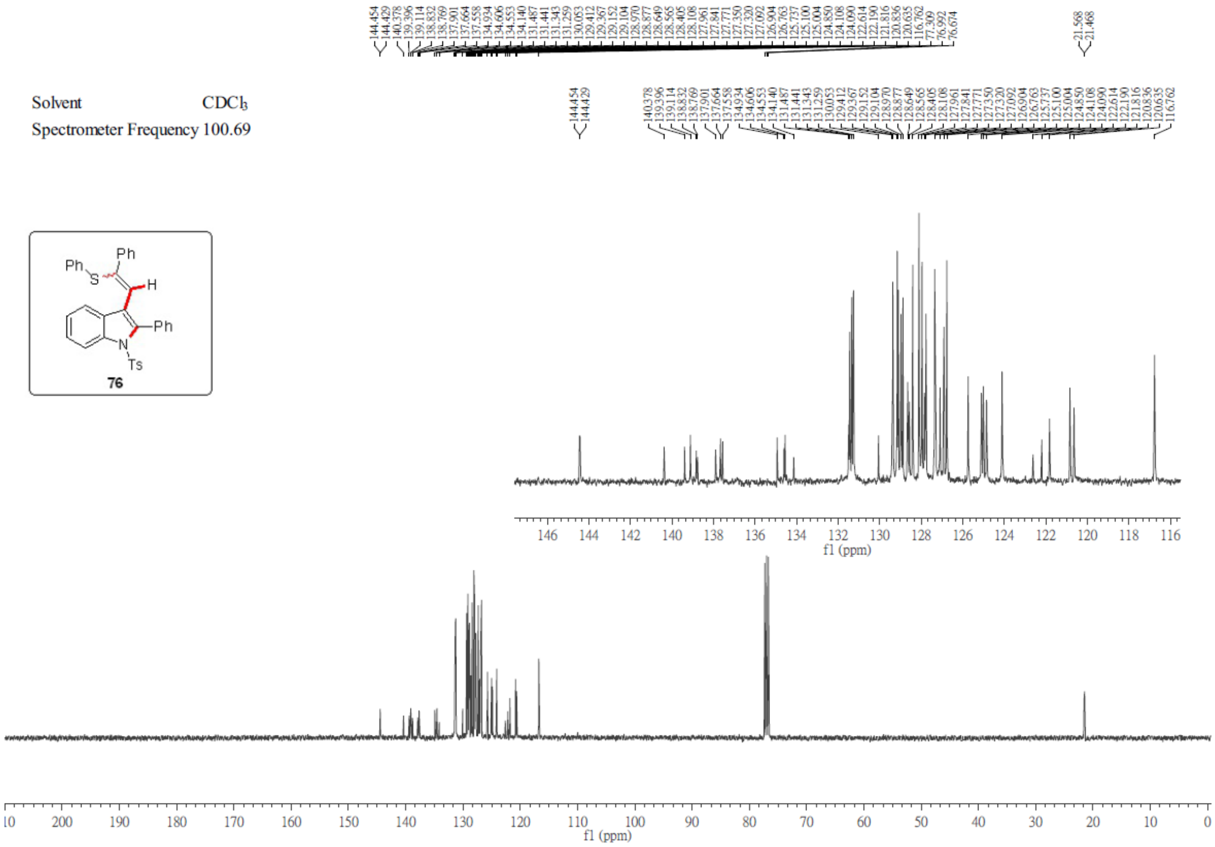

Supplementary Figure 163. ^{1}H (top) and ^{13}C (bottom) NMR spectra of compound **76**.

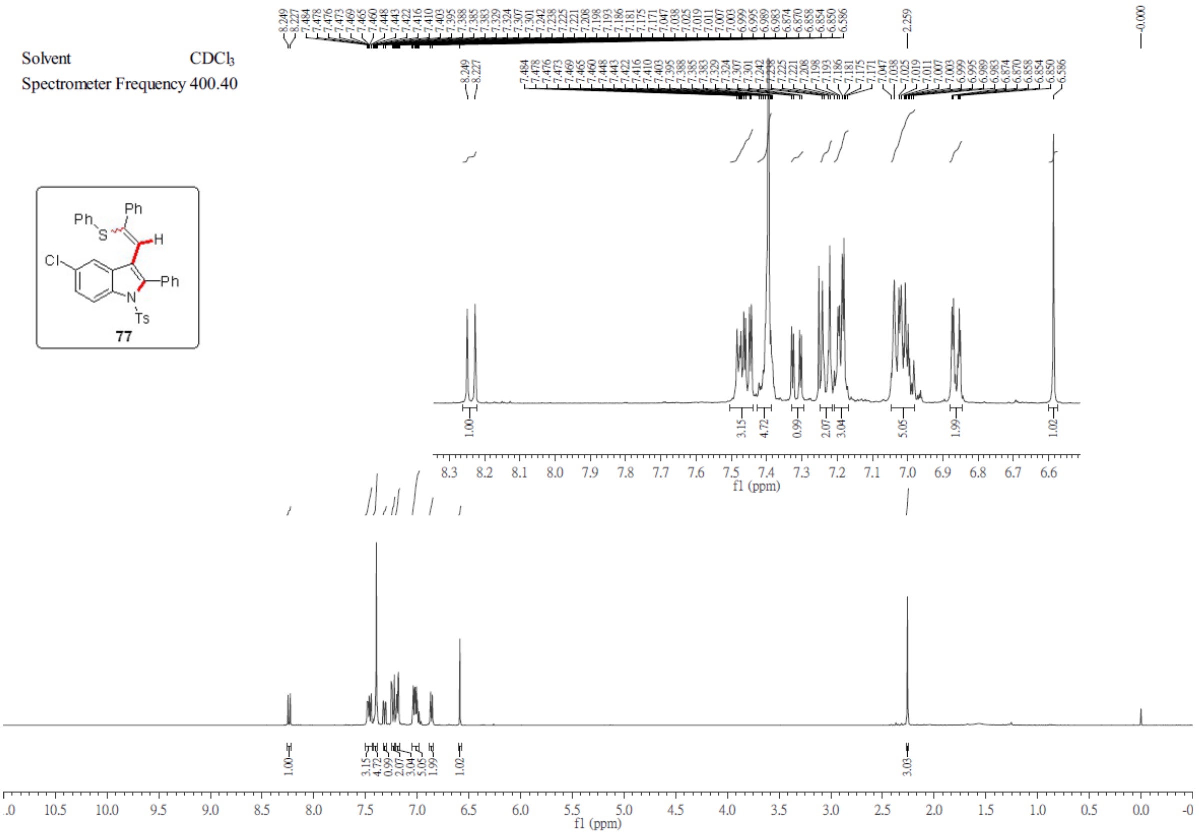

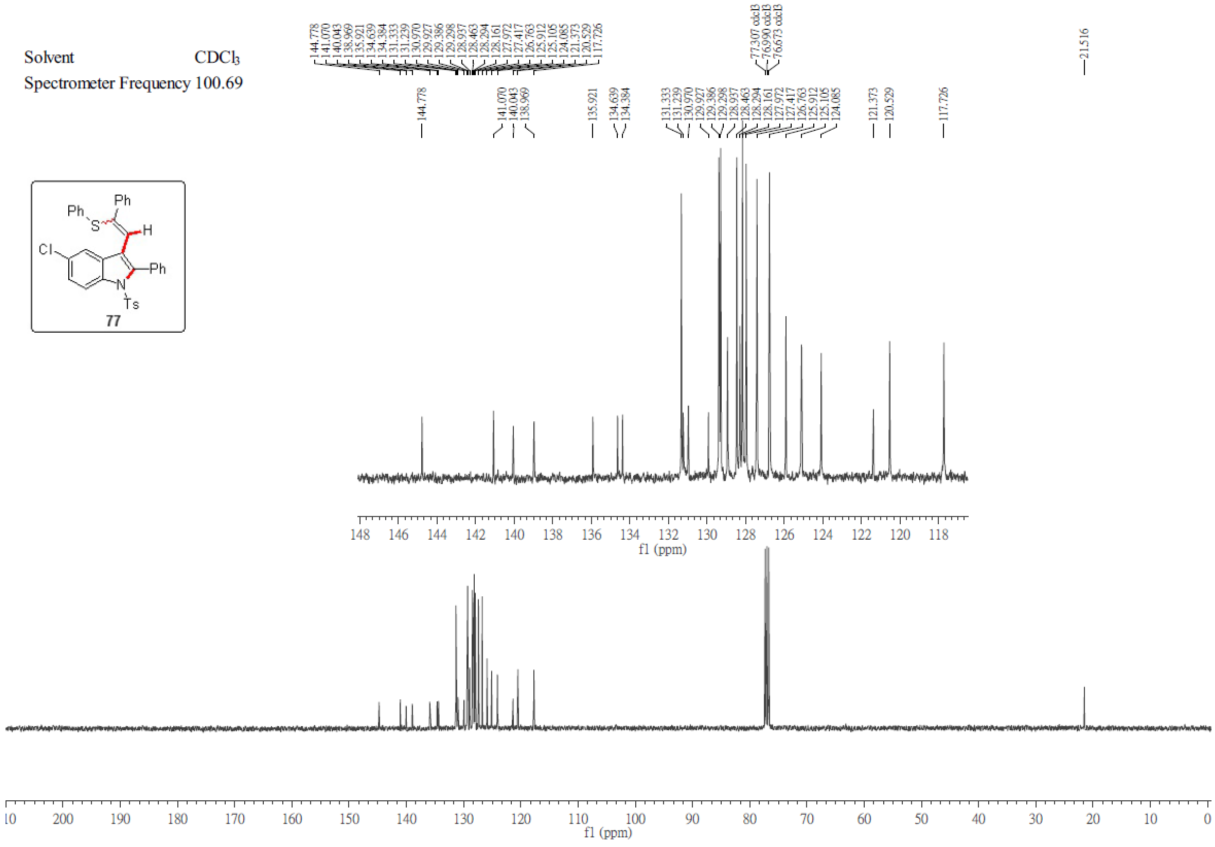

Supplementary Figure 164. ^{1}H (top) and ^{13}C (bottom) NMR spectra of compound **77**.

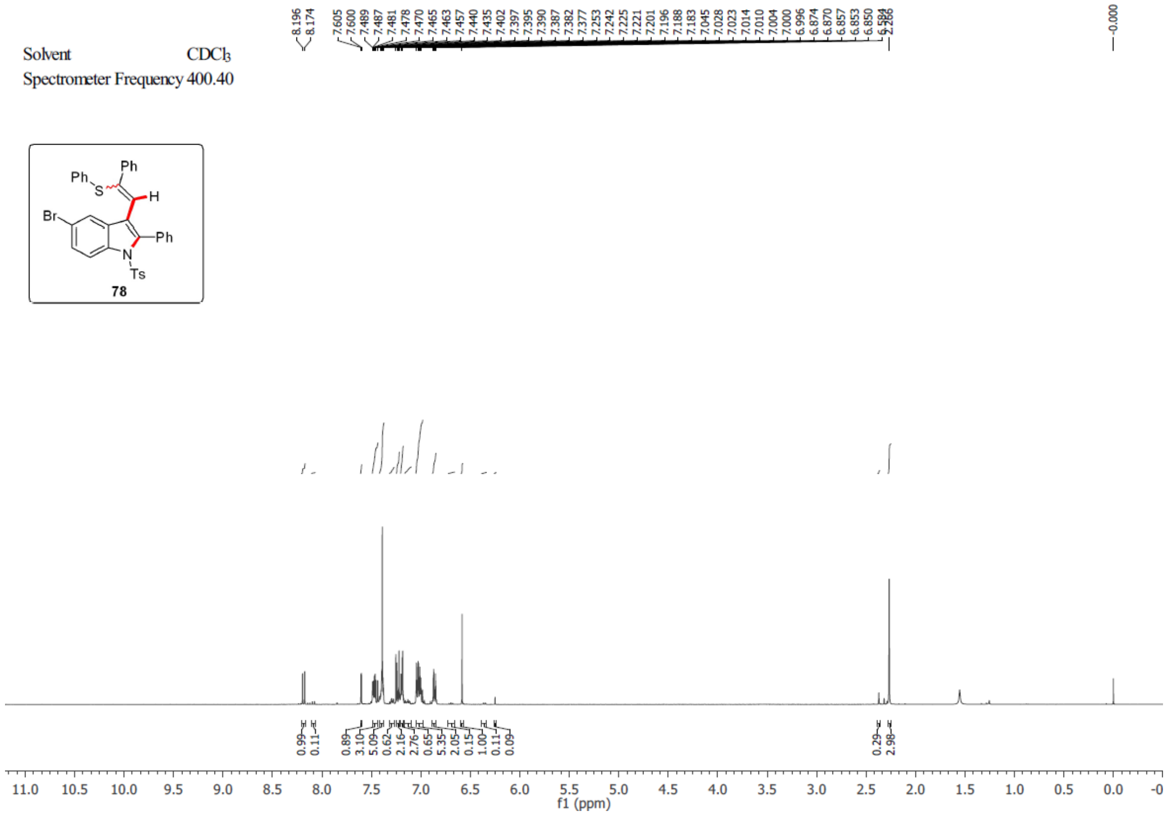

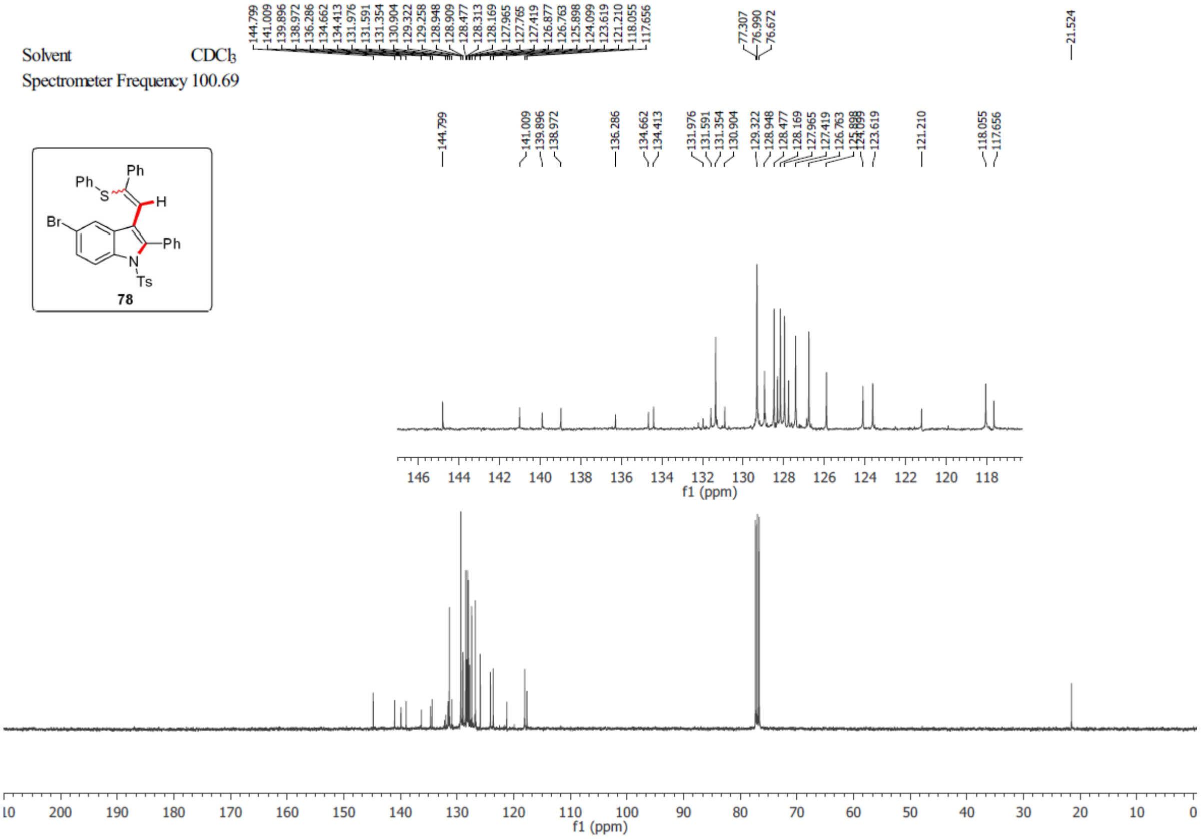

Supplementary Figure 165. ^{1}H (top) and ^{13}C (bottom) NMR spectra of compound **78**.

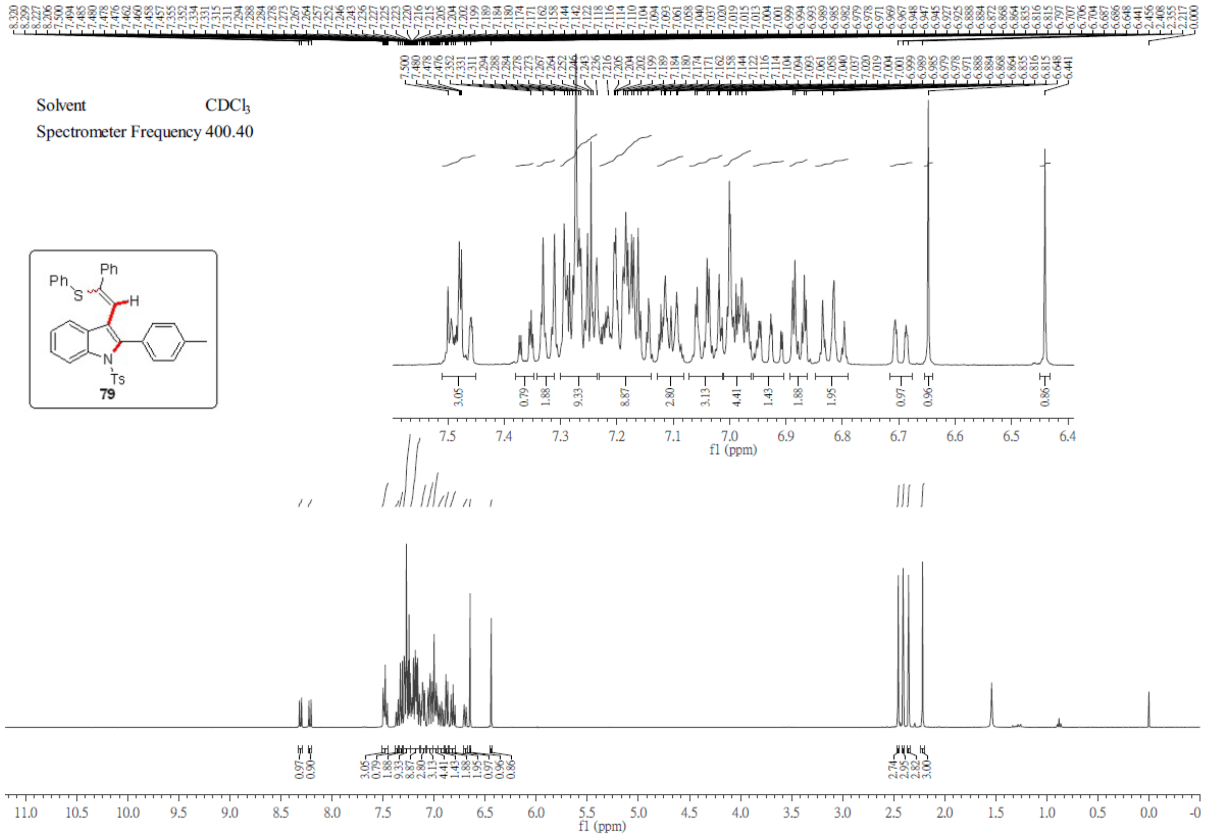

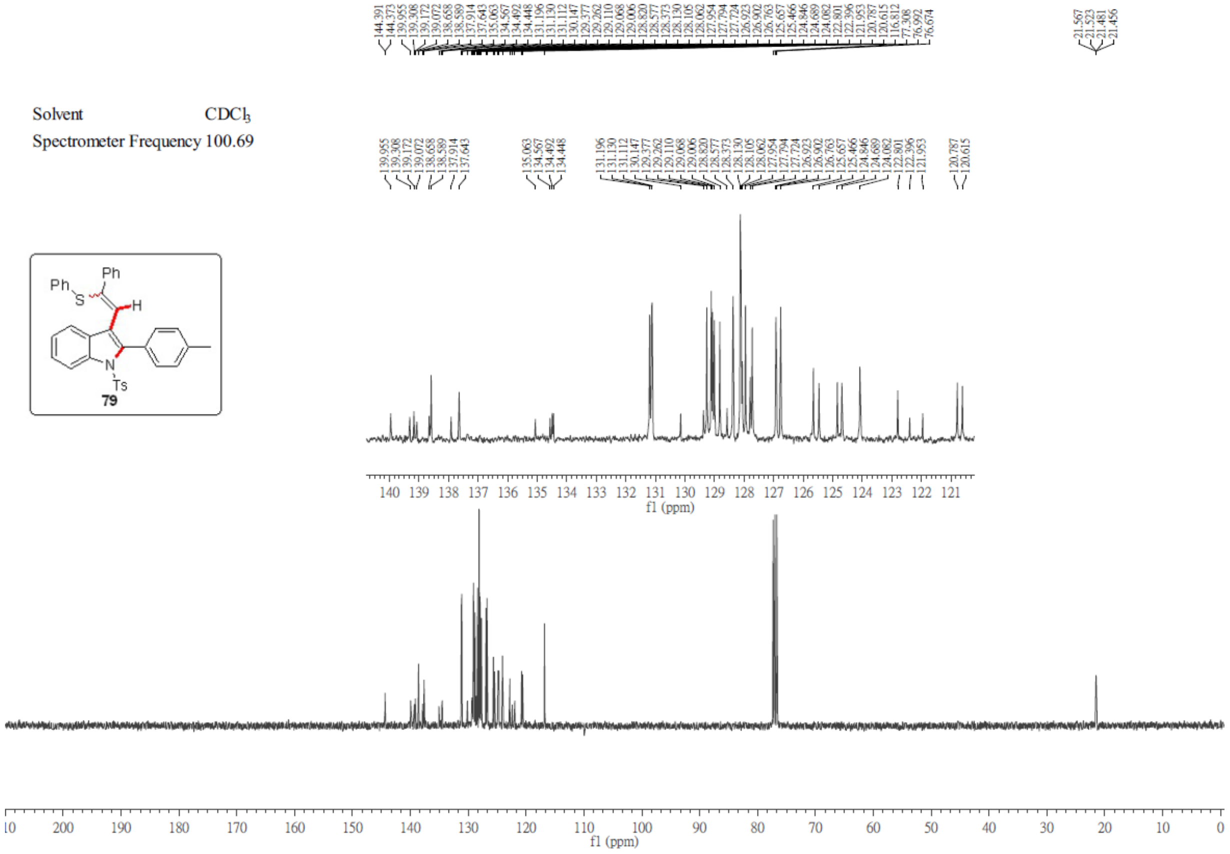

Supplementary Figure 166. ^{1}H (top) and ^{13}C (bottom) NMR spectra of compound **79**.

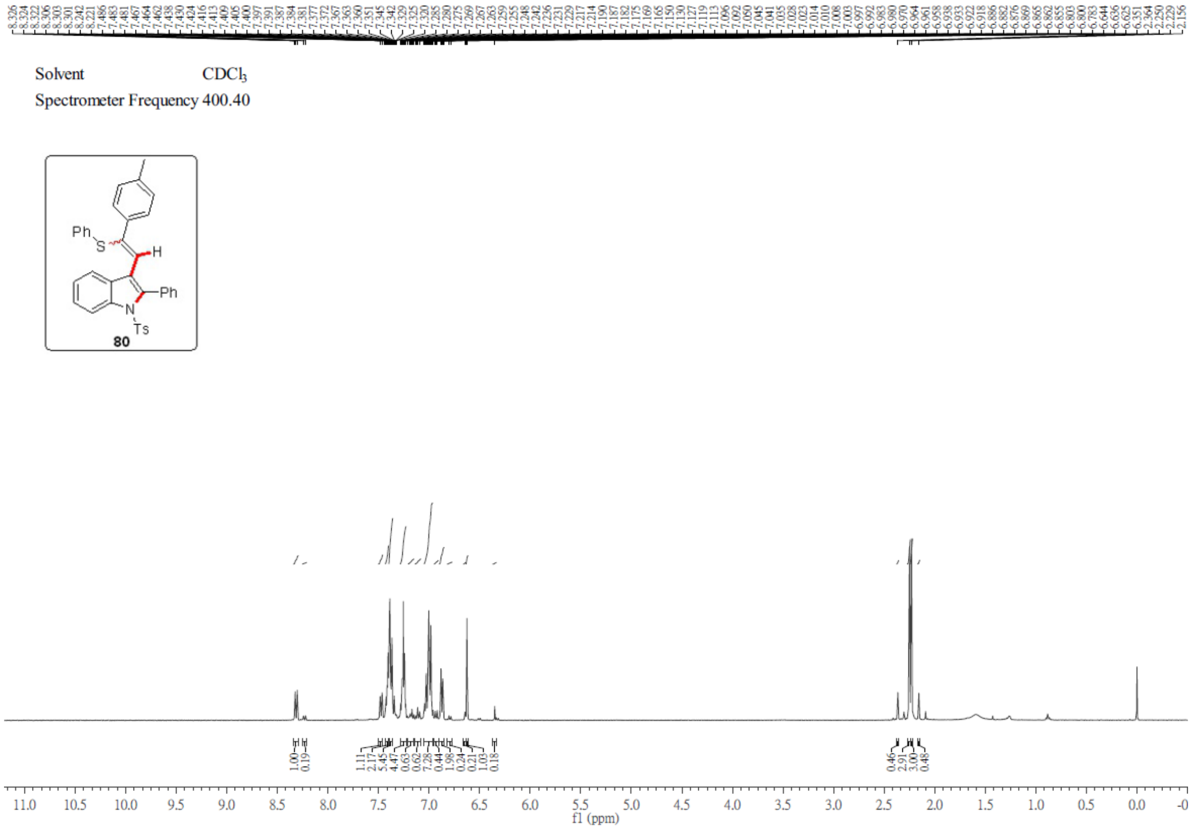

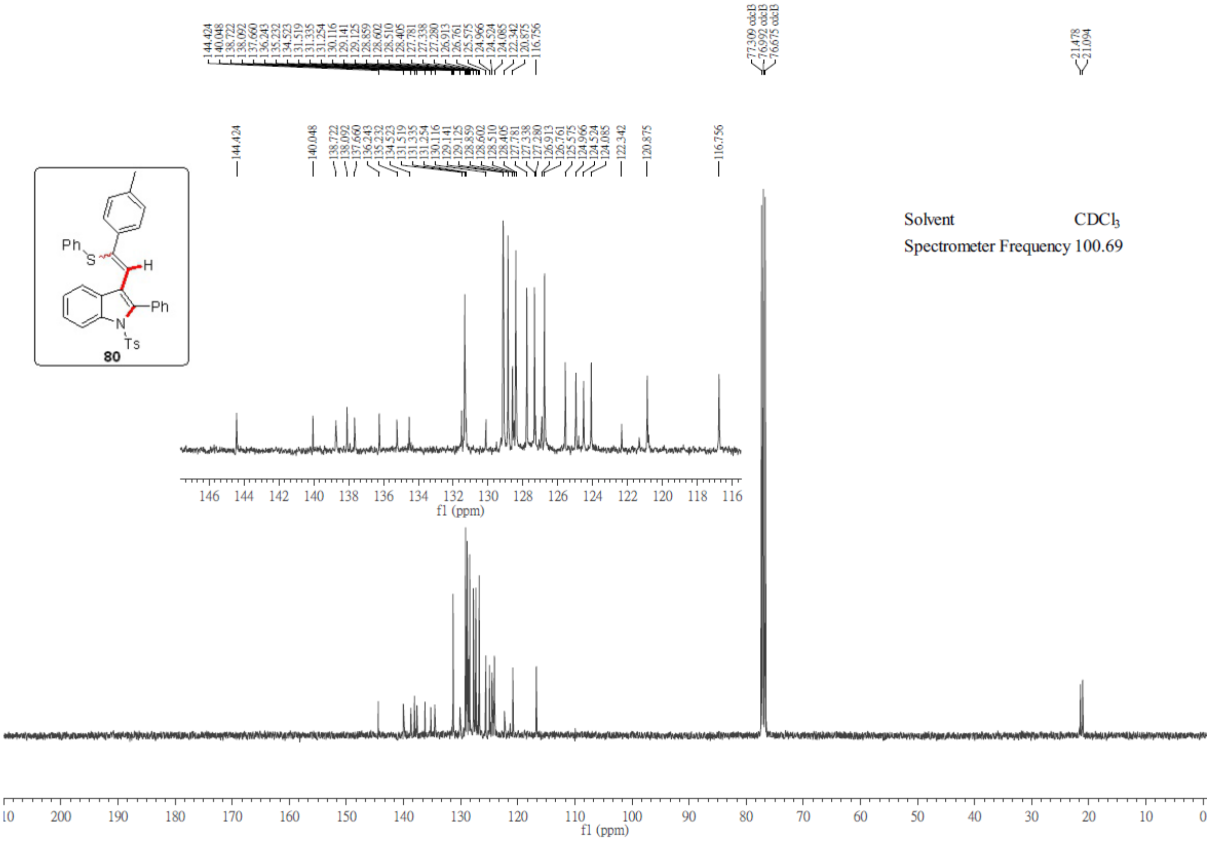

Supplementary Figure 167. ^{1}H (top) and ^{13}C (bottom) NMR spectra of compound **80**.

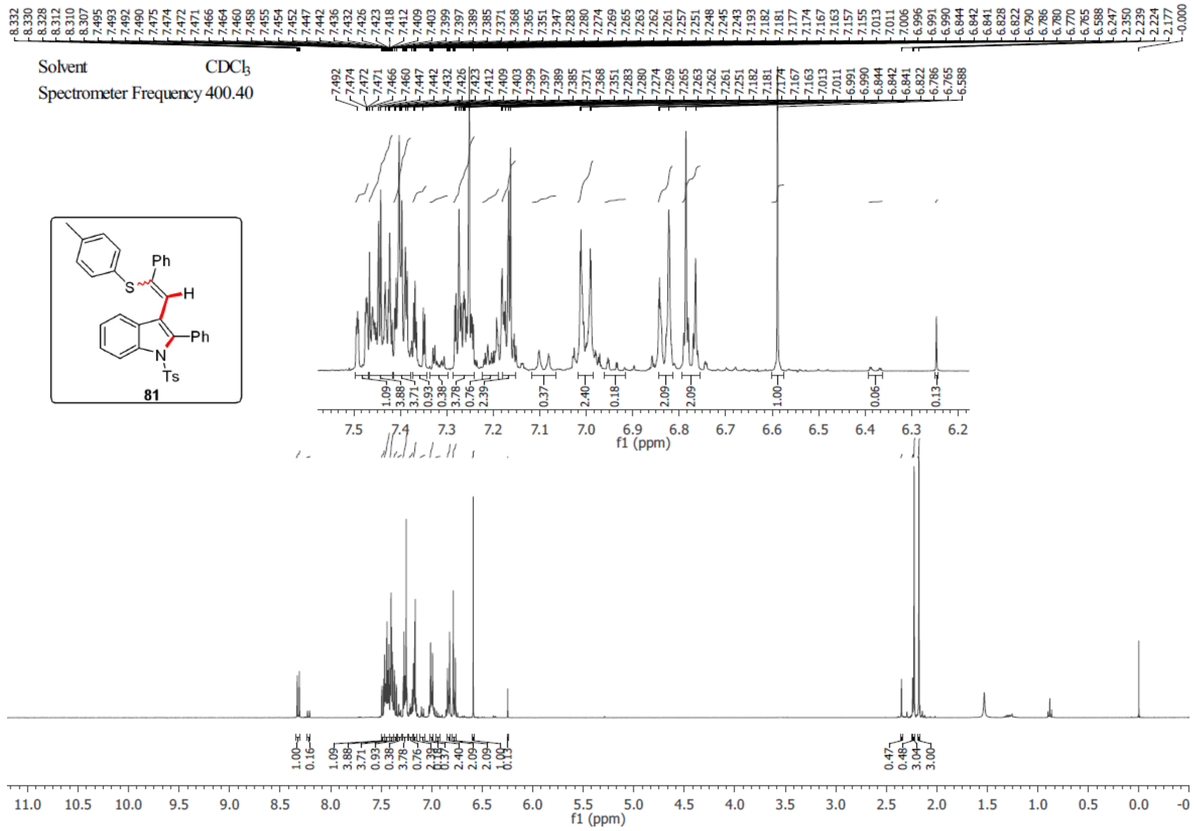

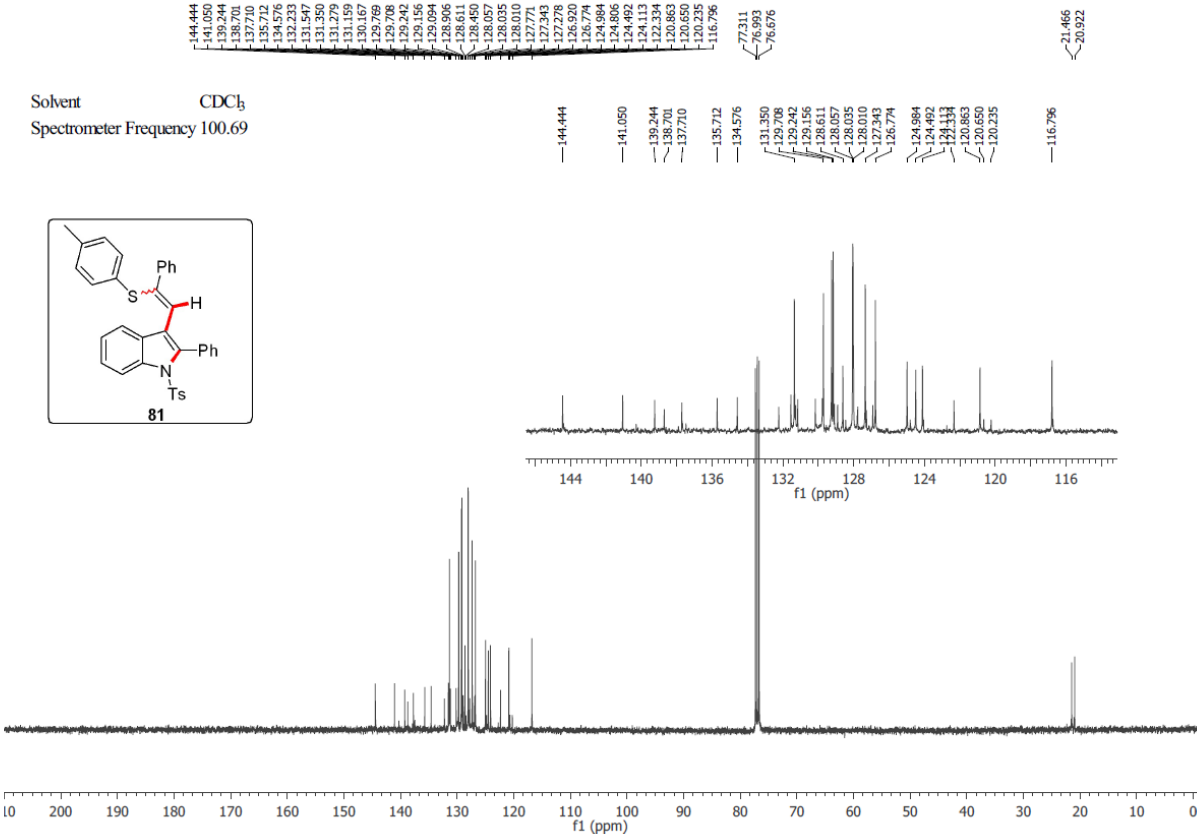

Supplementary Figure 168. ^{1}H (top) and ^{13}C (bottom) NMR spectra of compound **81**.

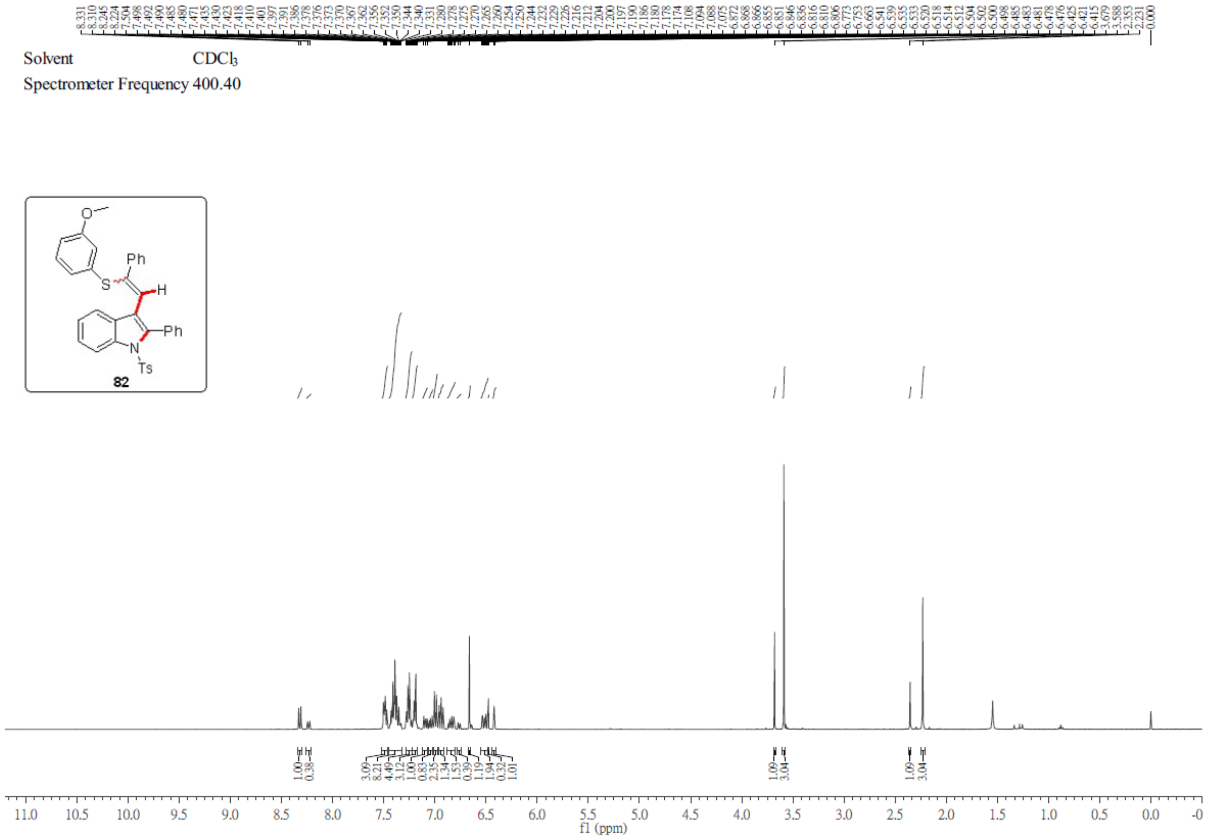

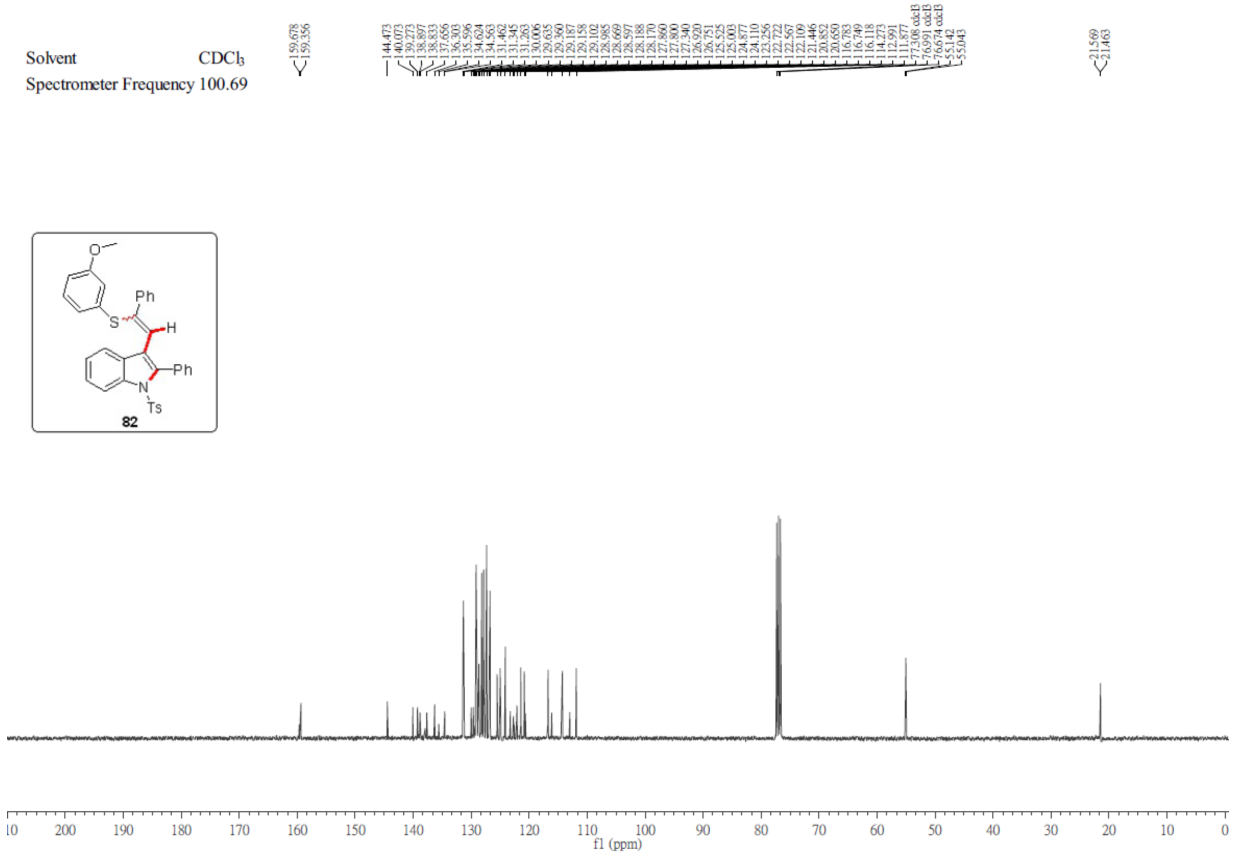

Supplementary Figure 169. ^{1}H (top) and ^{13}C (bottom) NMR spectra of compound **82**.

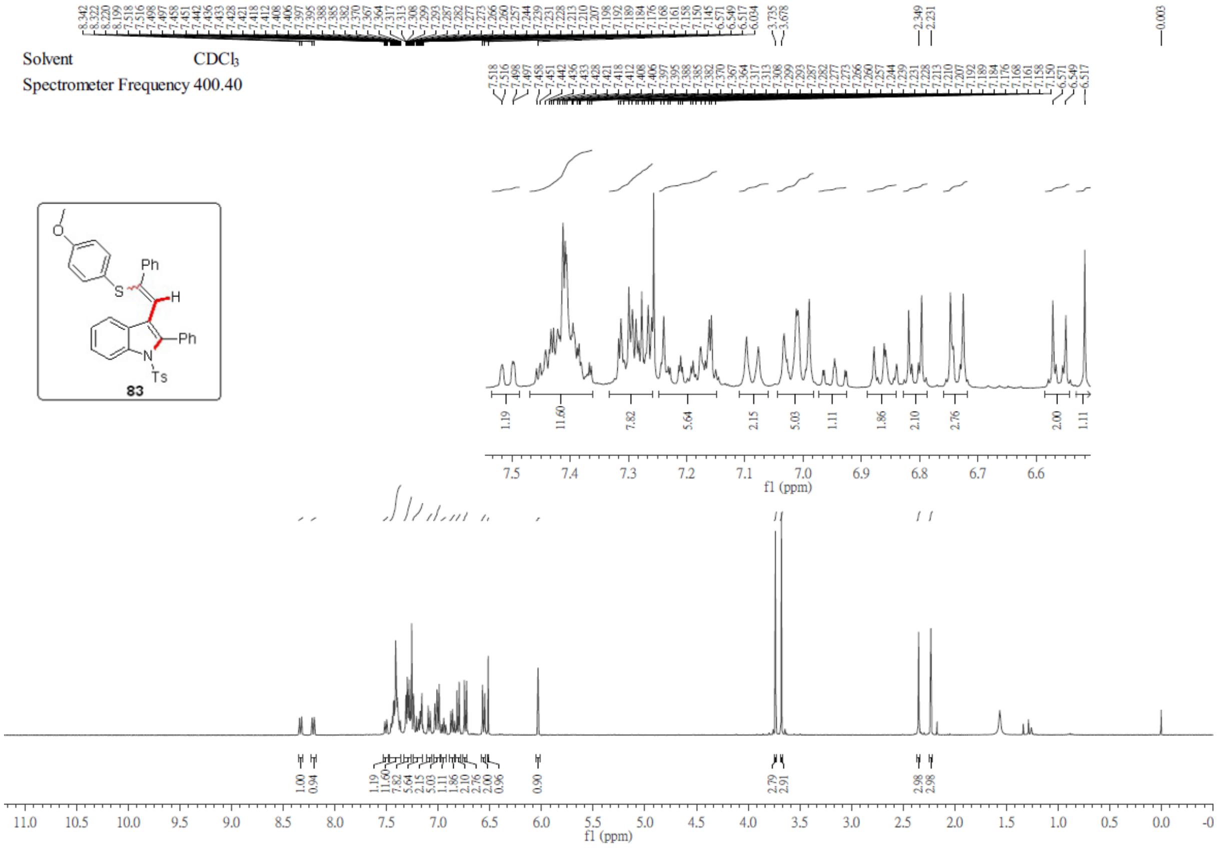

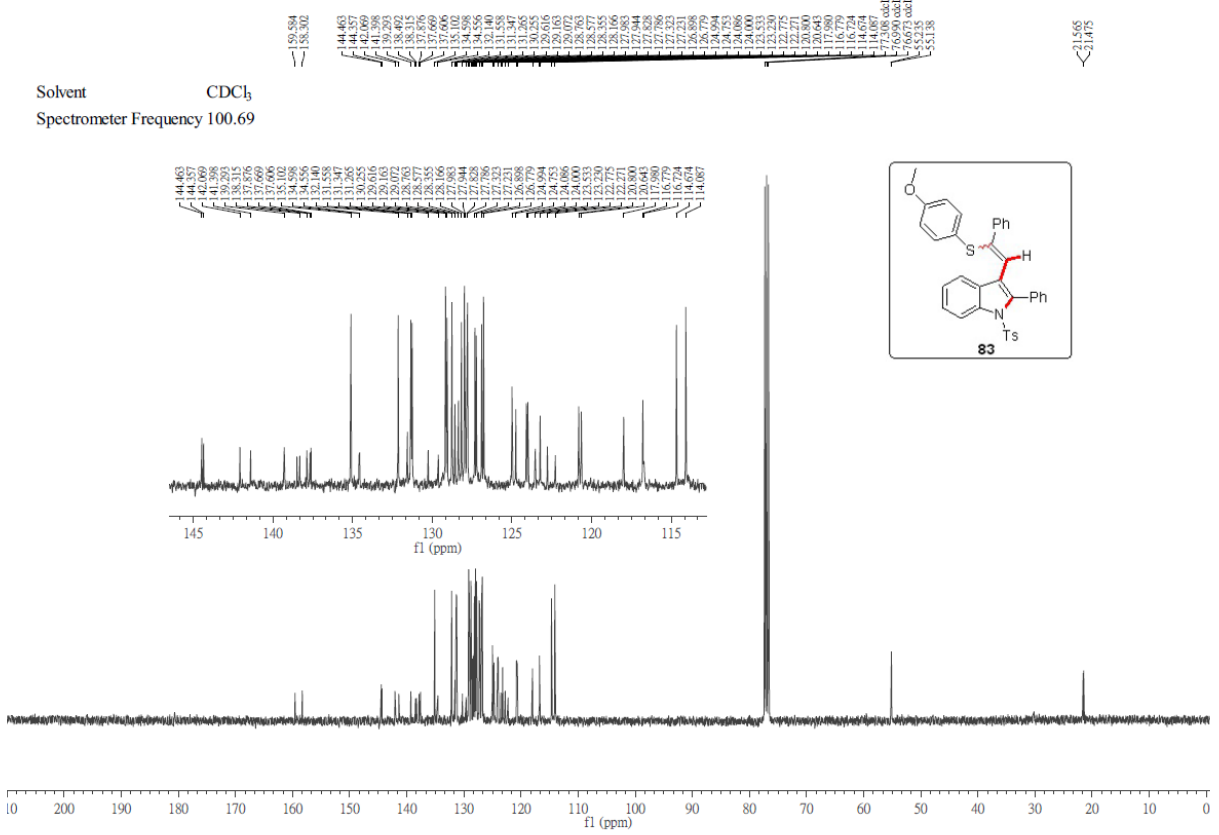

Supplementary Figure 170. ^{1}H (top) and ^{13}C (bottom) NMR spectra of compound **83**.

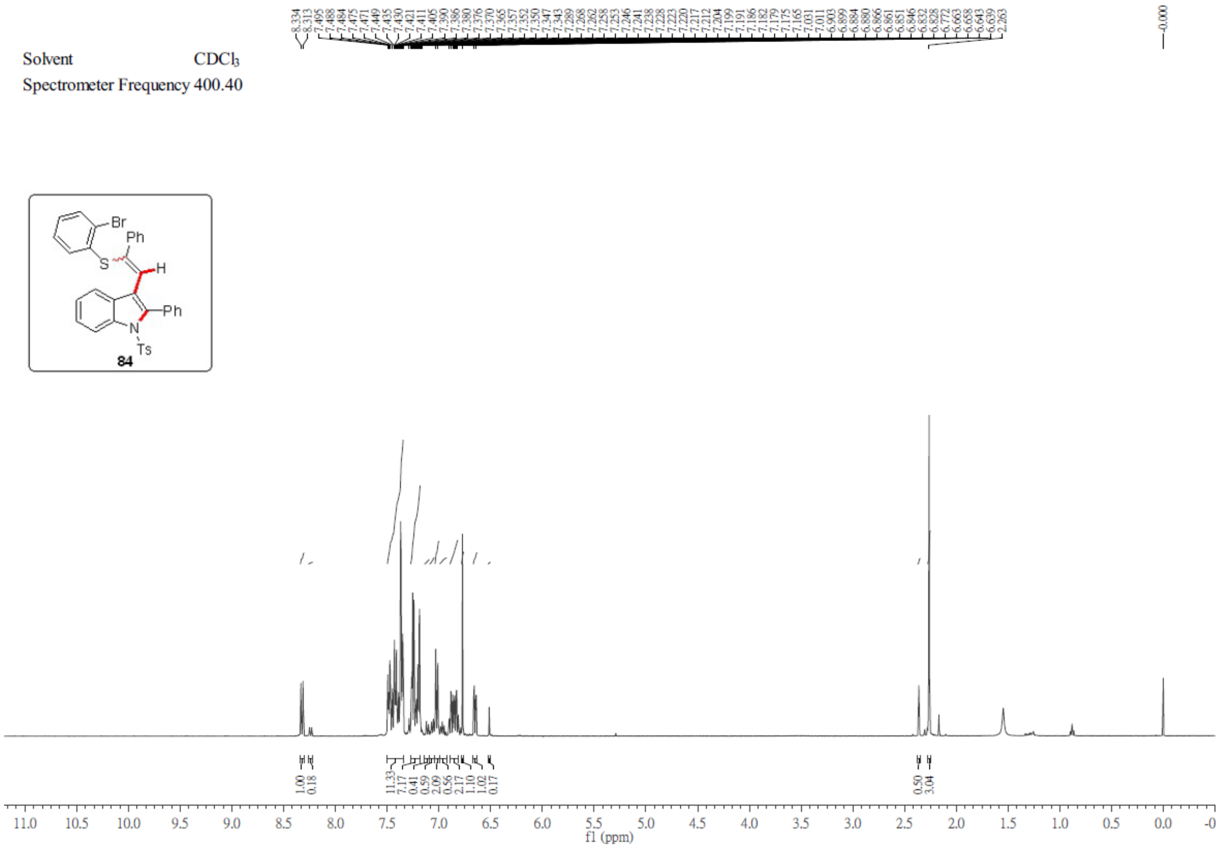

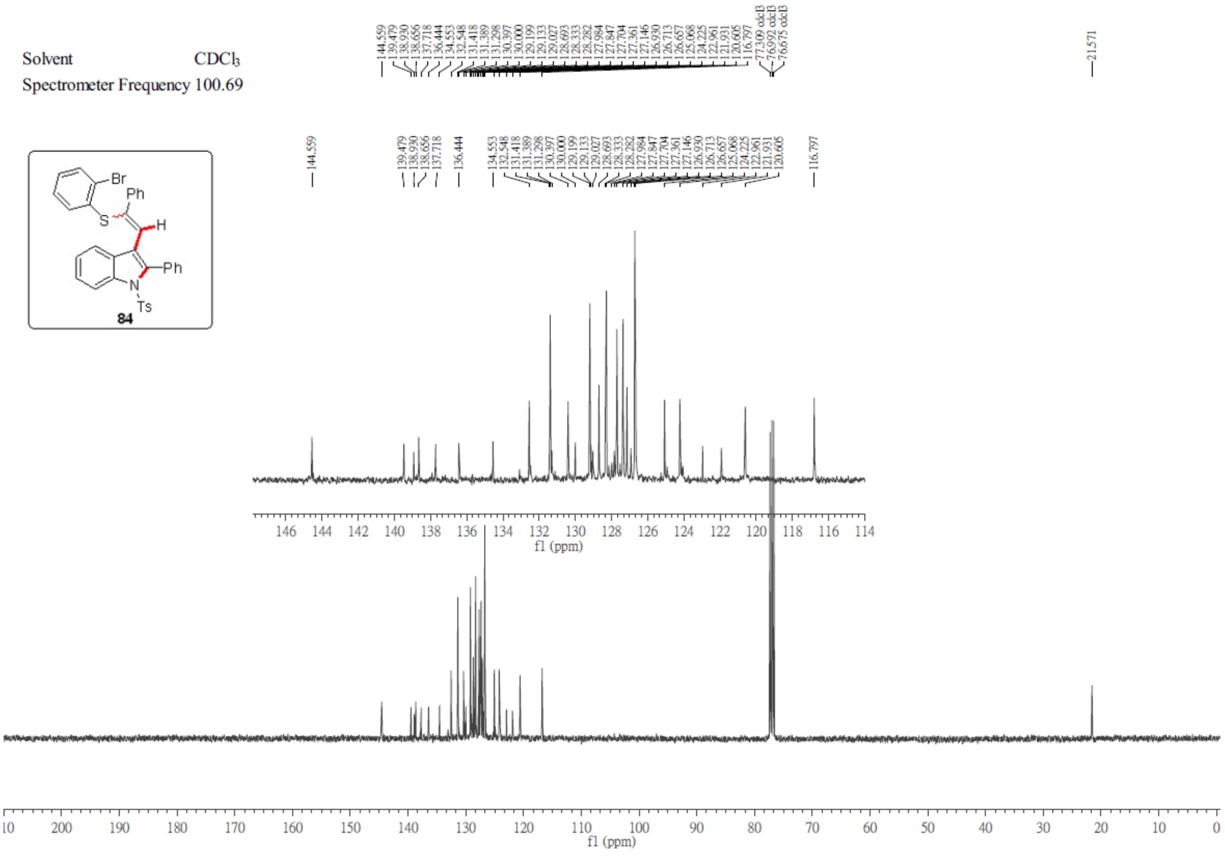

Supplementary Figure 171. ^{1}H (top) and ^{13}C (bottom) NMR spectra of compound **84**.

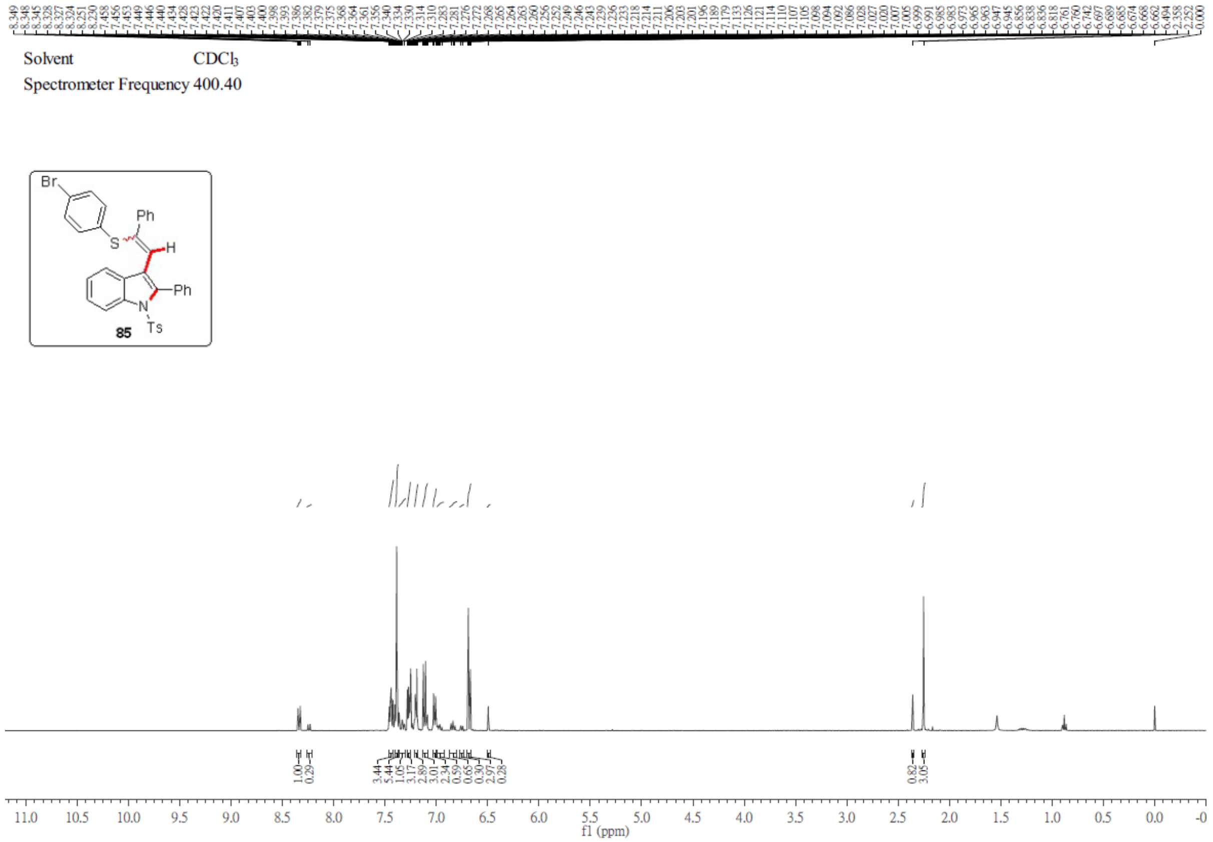

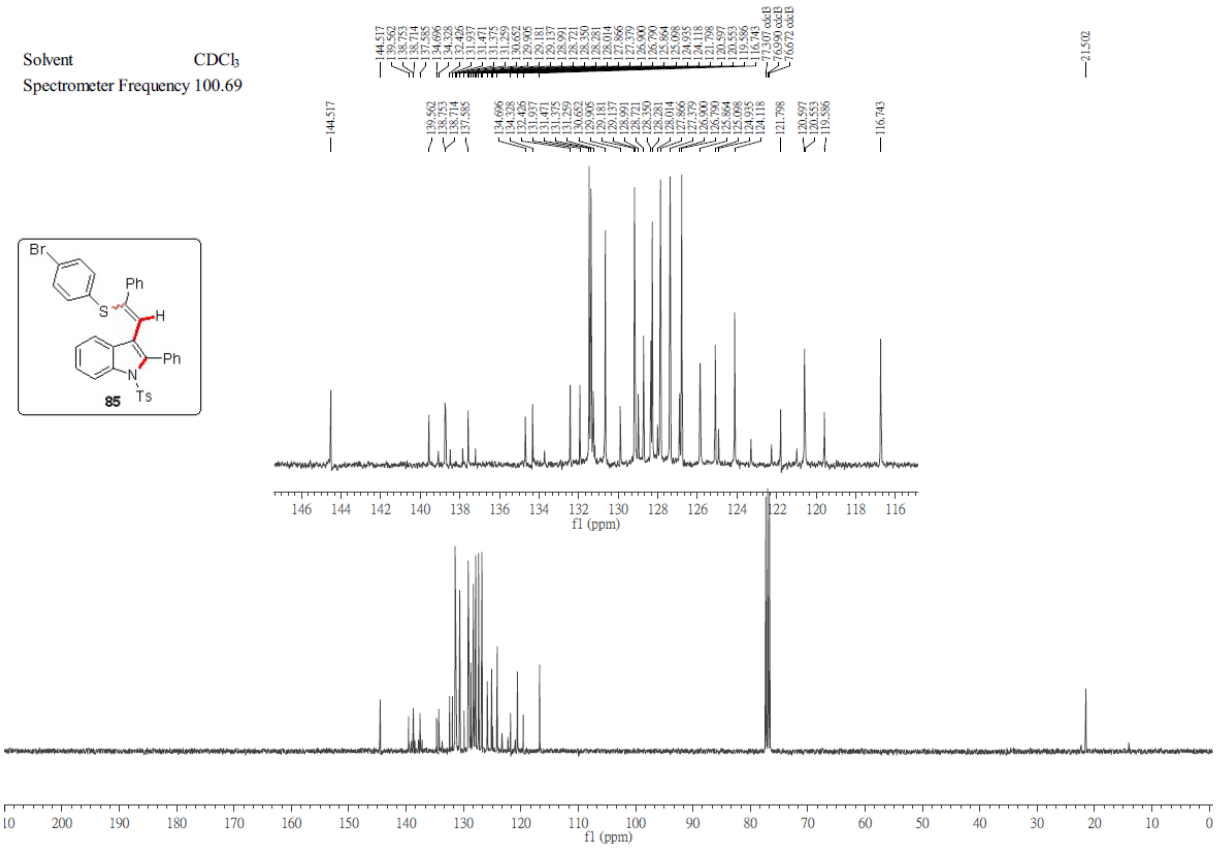

Supplementary Figure 172. ^{1}H (top) and ^{13}C (bottom) NMR spectra of compound **85**.

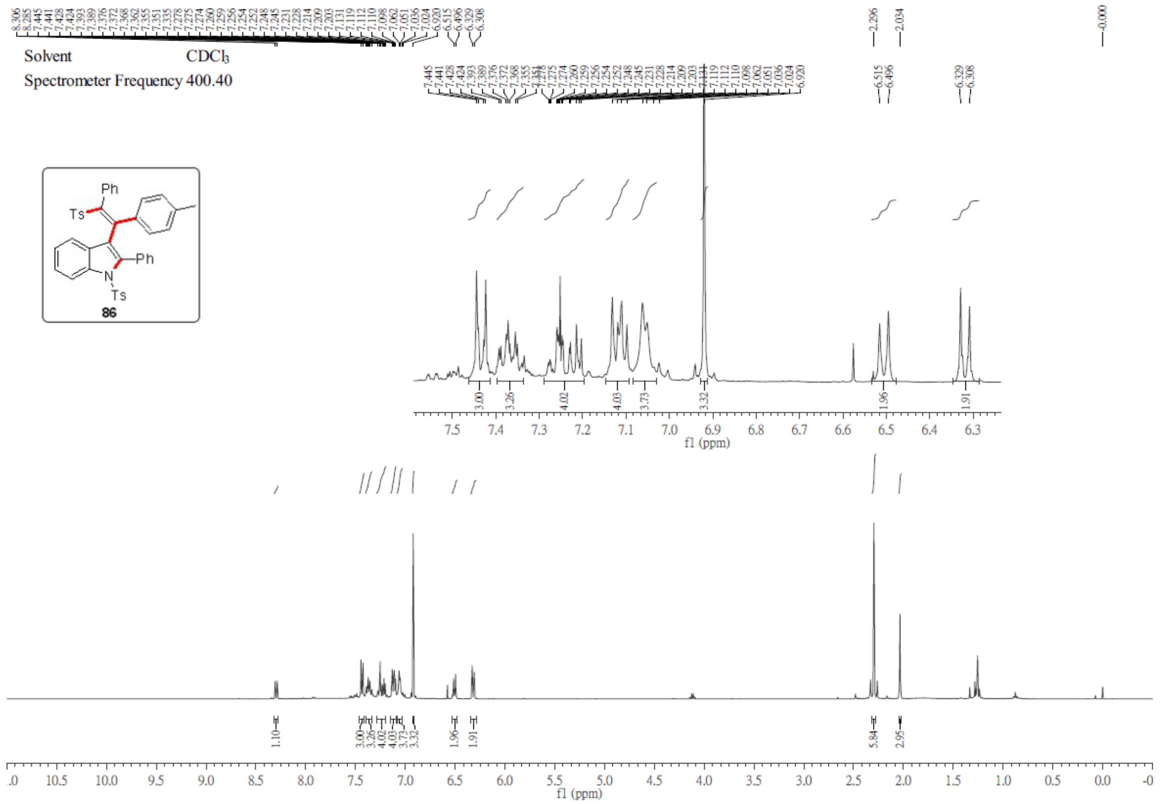

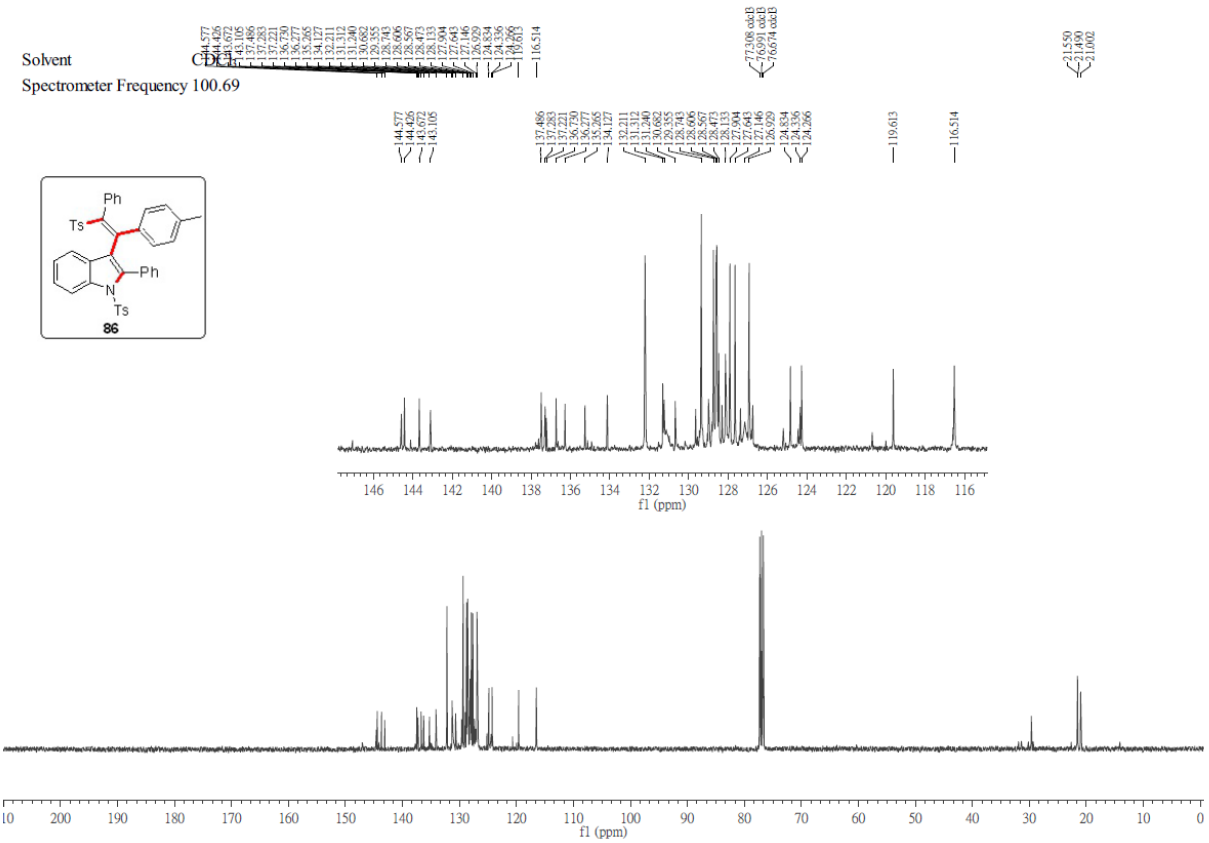

Supplementary Figure 173. ^{1}H (top) and ^{13}C (bottom) NMR spectra of compound **86**.

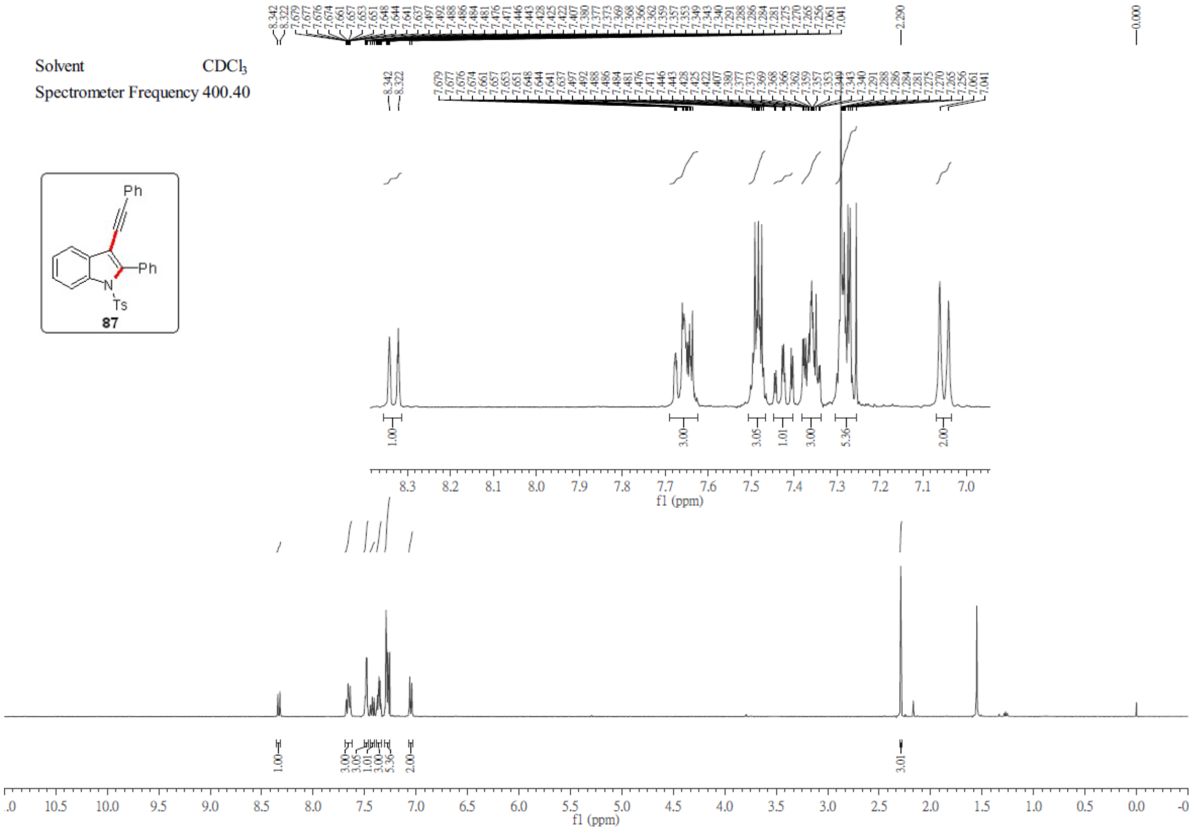

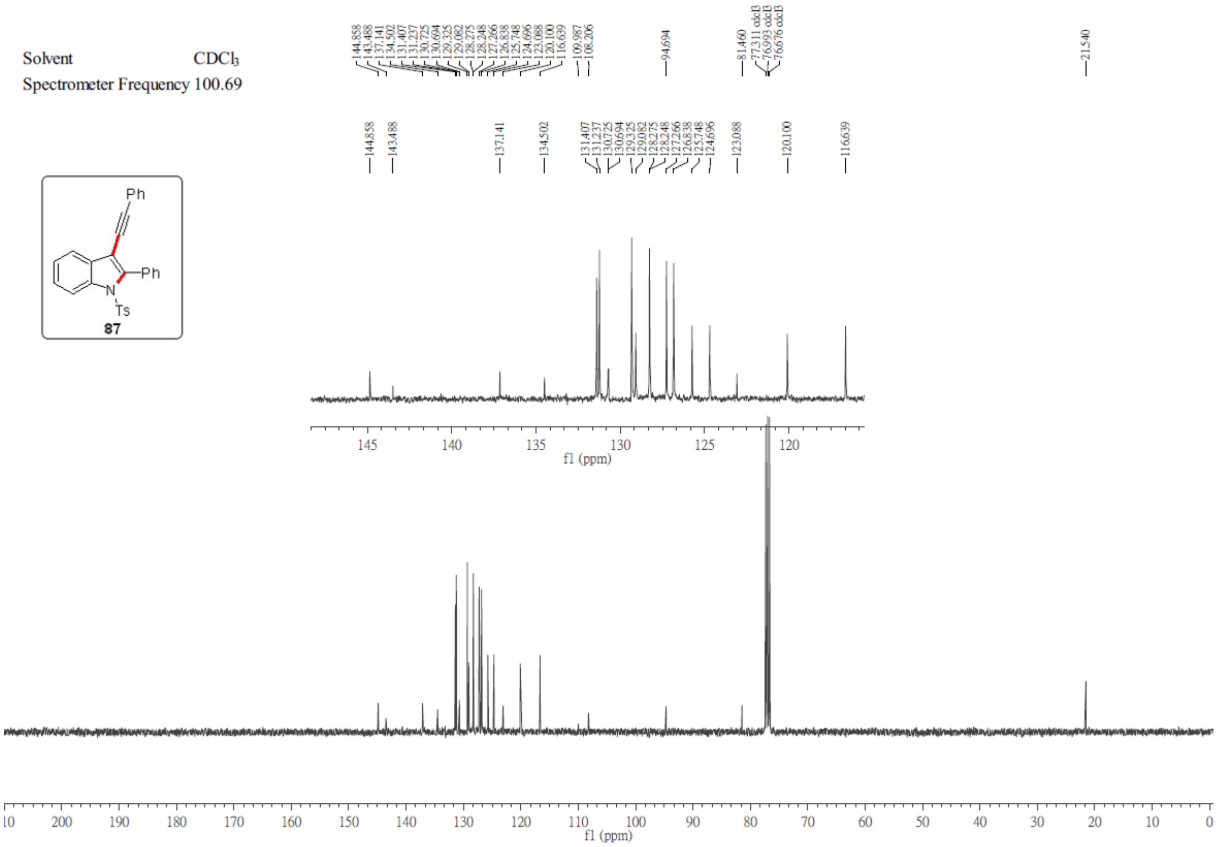

Supplementary Figure 174. ^{1}H (top) and ^{13}C (bottom) NMR spectra of compound **87**.

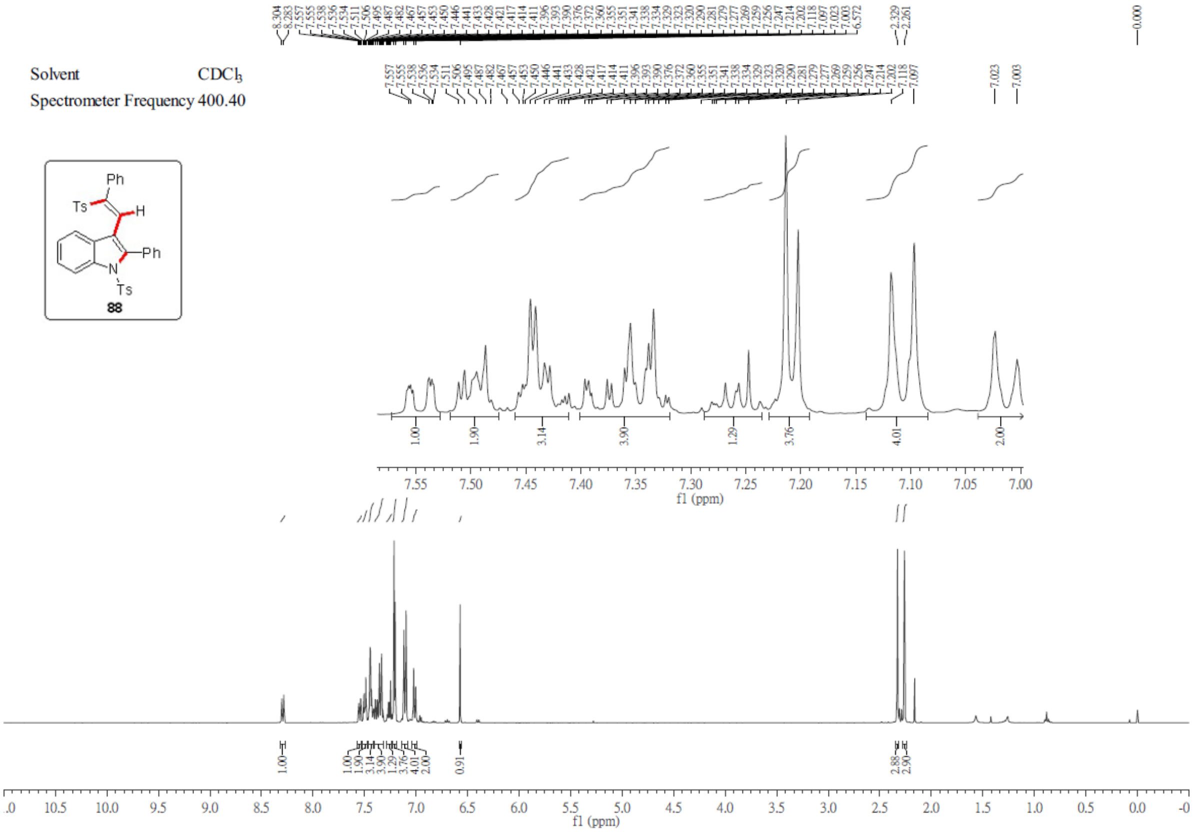

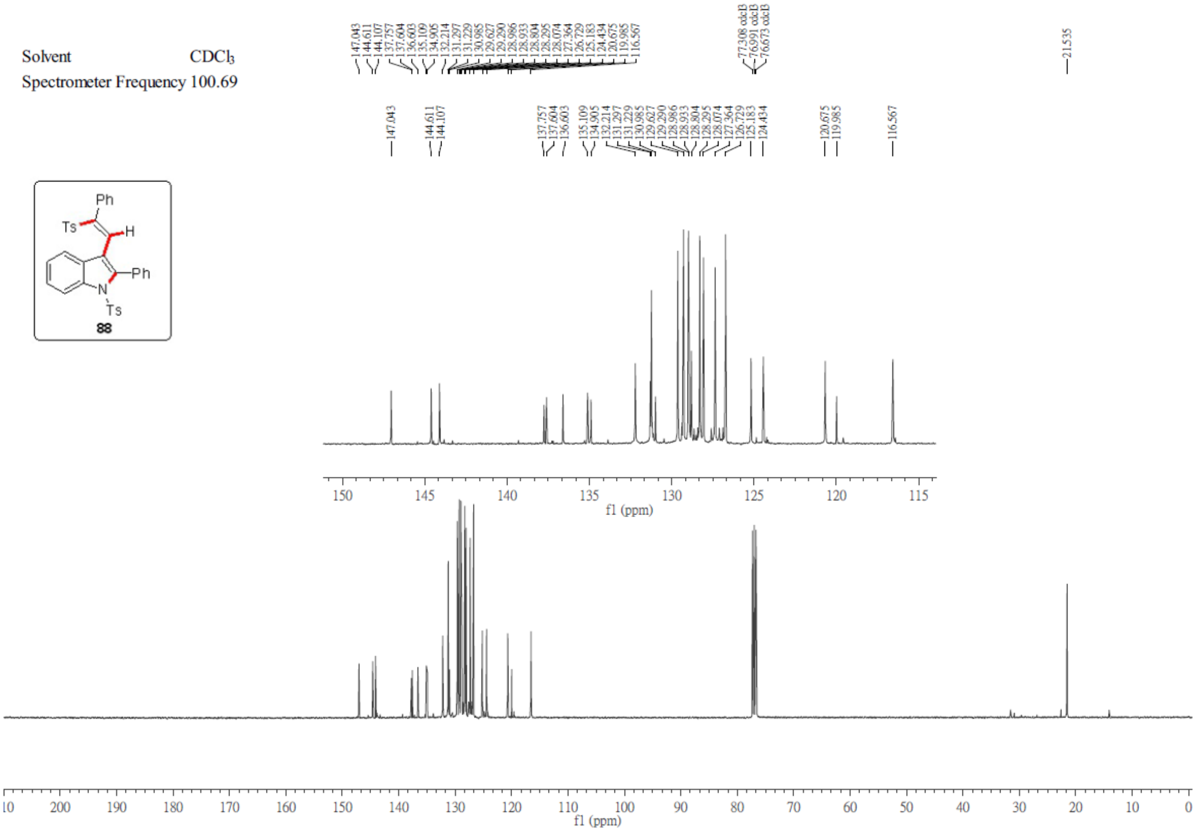

Supplementary Figure 175. ^{1}H (top) and ^{13}C (bottom) NMR spectra of compound **88**.

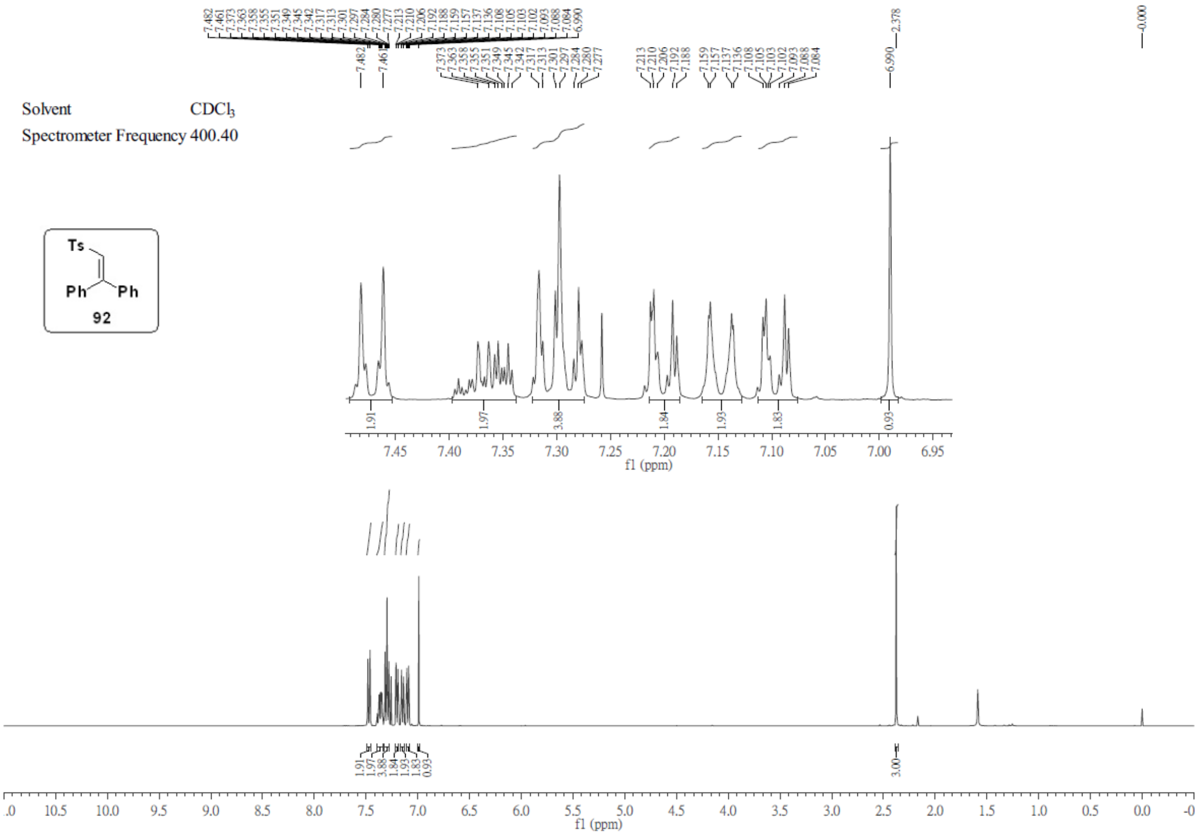

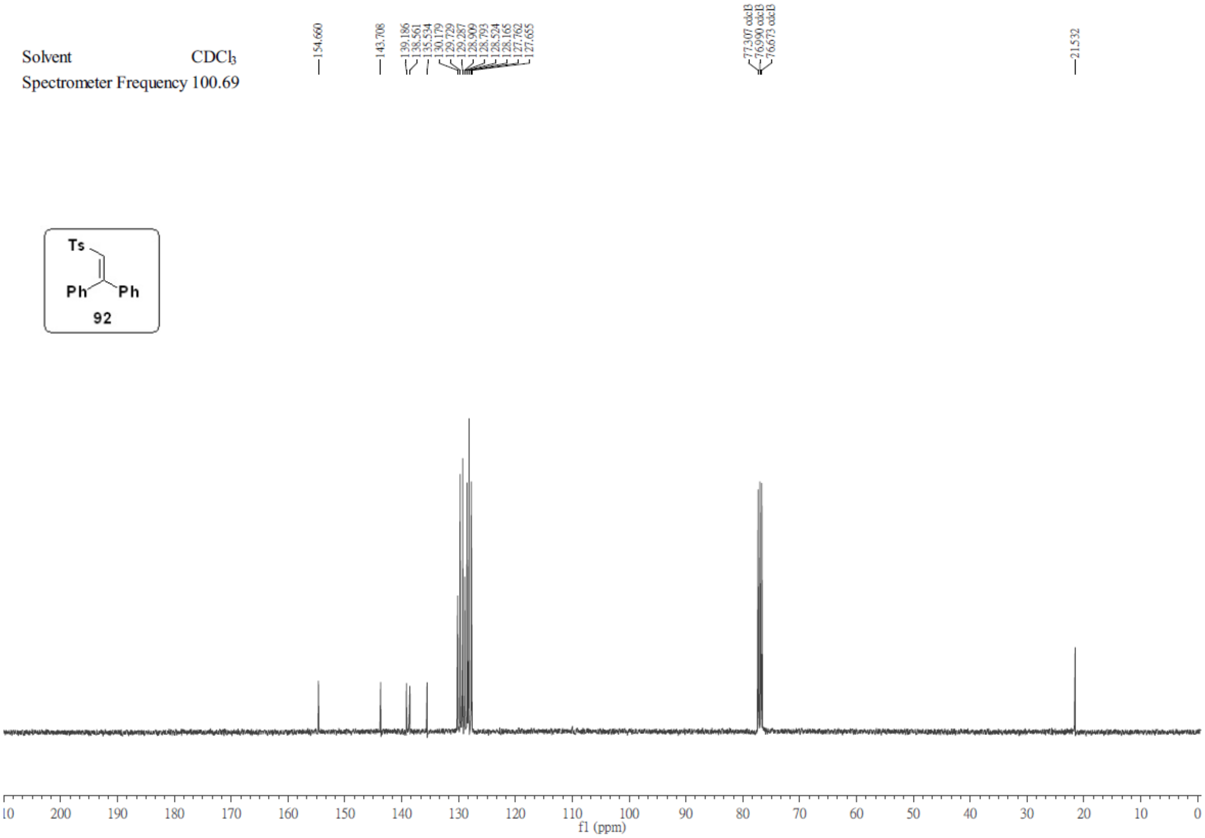

Supplementary Figure 176. ^{1}H (top) and ^{13}C (bottom) NMR spectra of compound **92**.

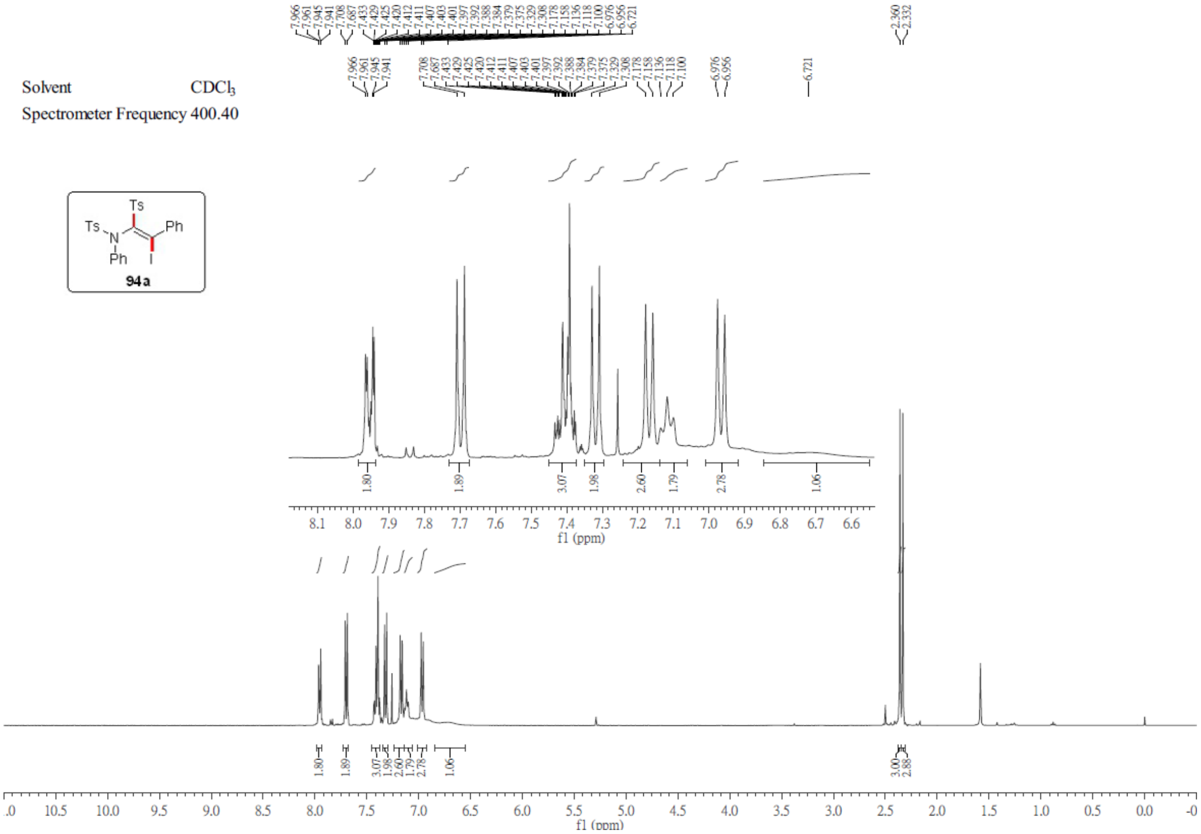

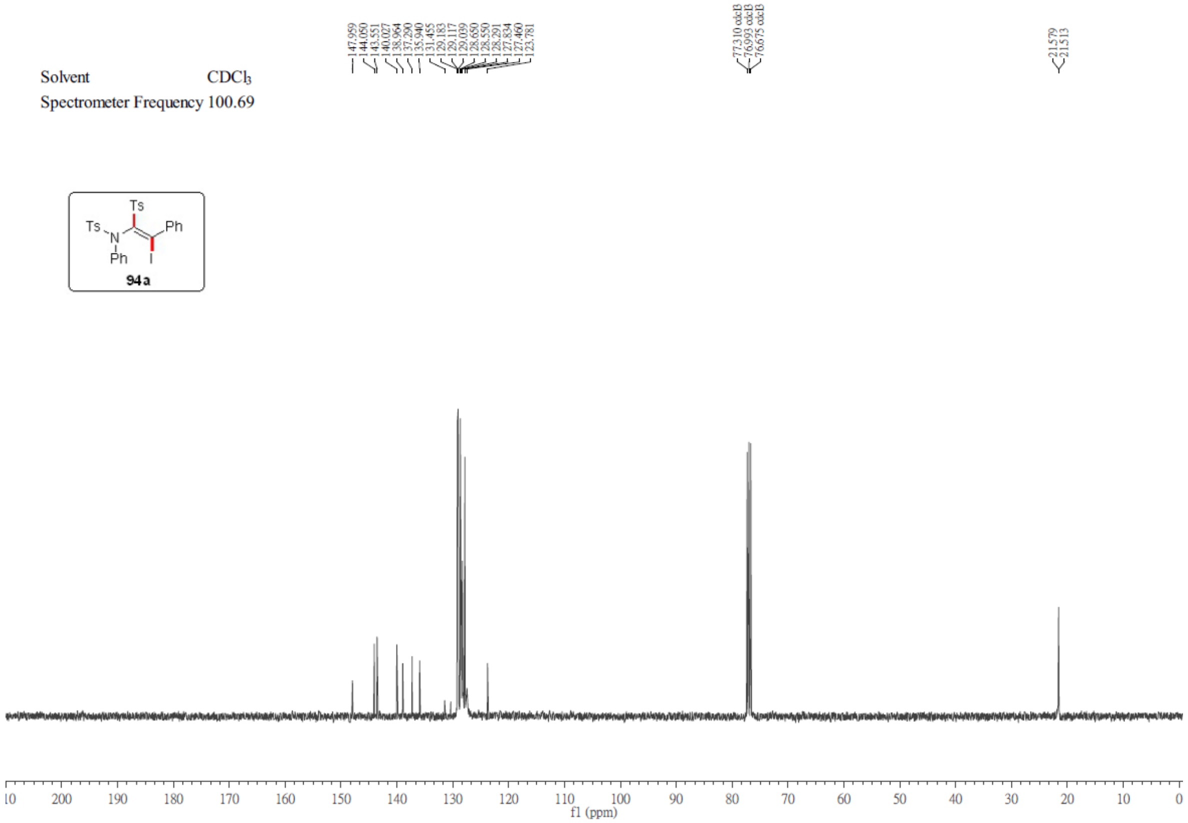

Supplementary Figure 177. ^{1}H (top) and ^{13}C (bottom) NMR spectra of compound **94a**.

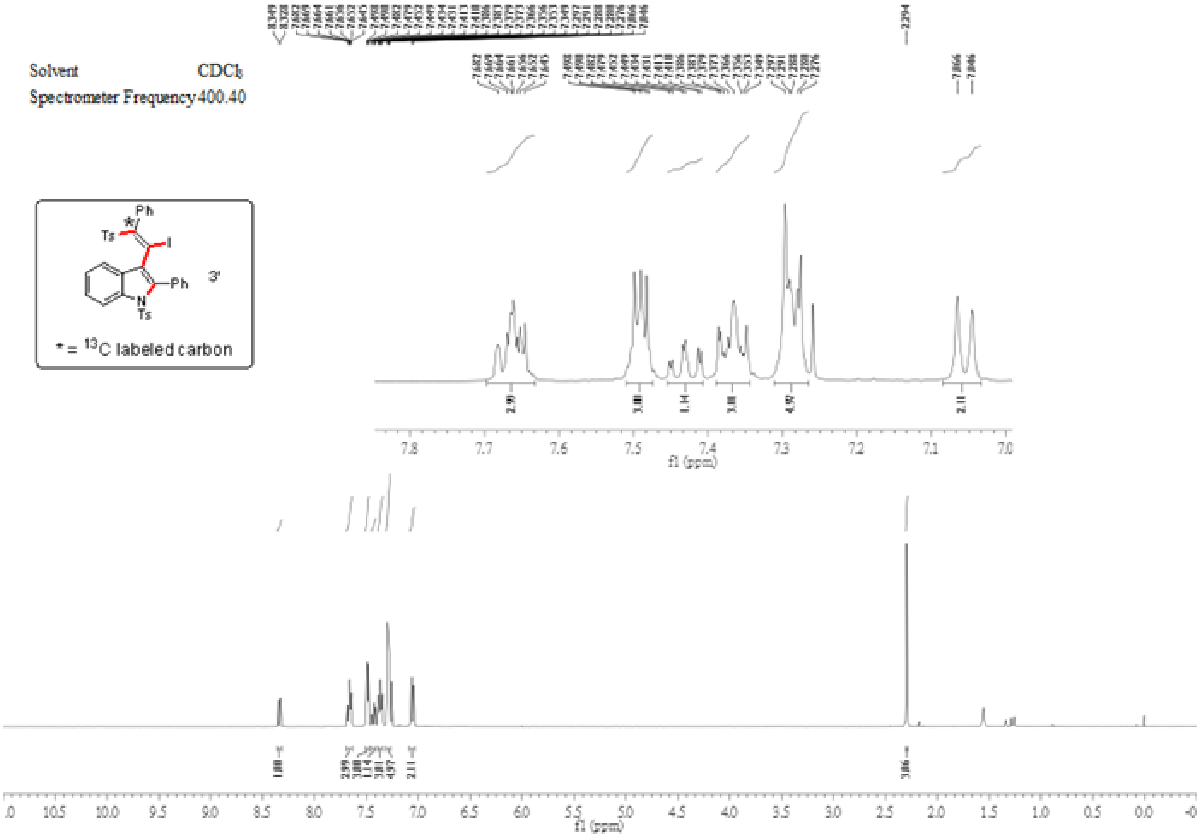

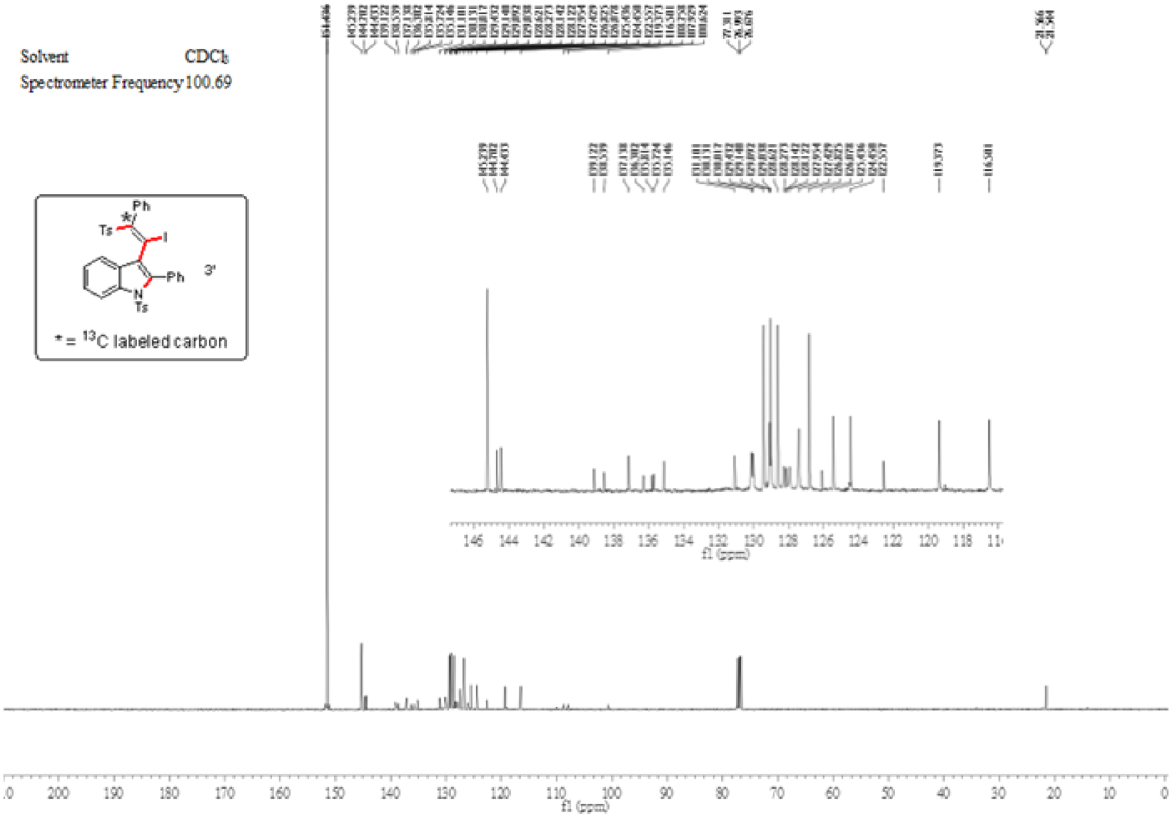

Supplementary Figure 178. ^{1}H (top) and ^{13}C (bottom) NMR spectra of compound **3’**.

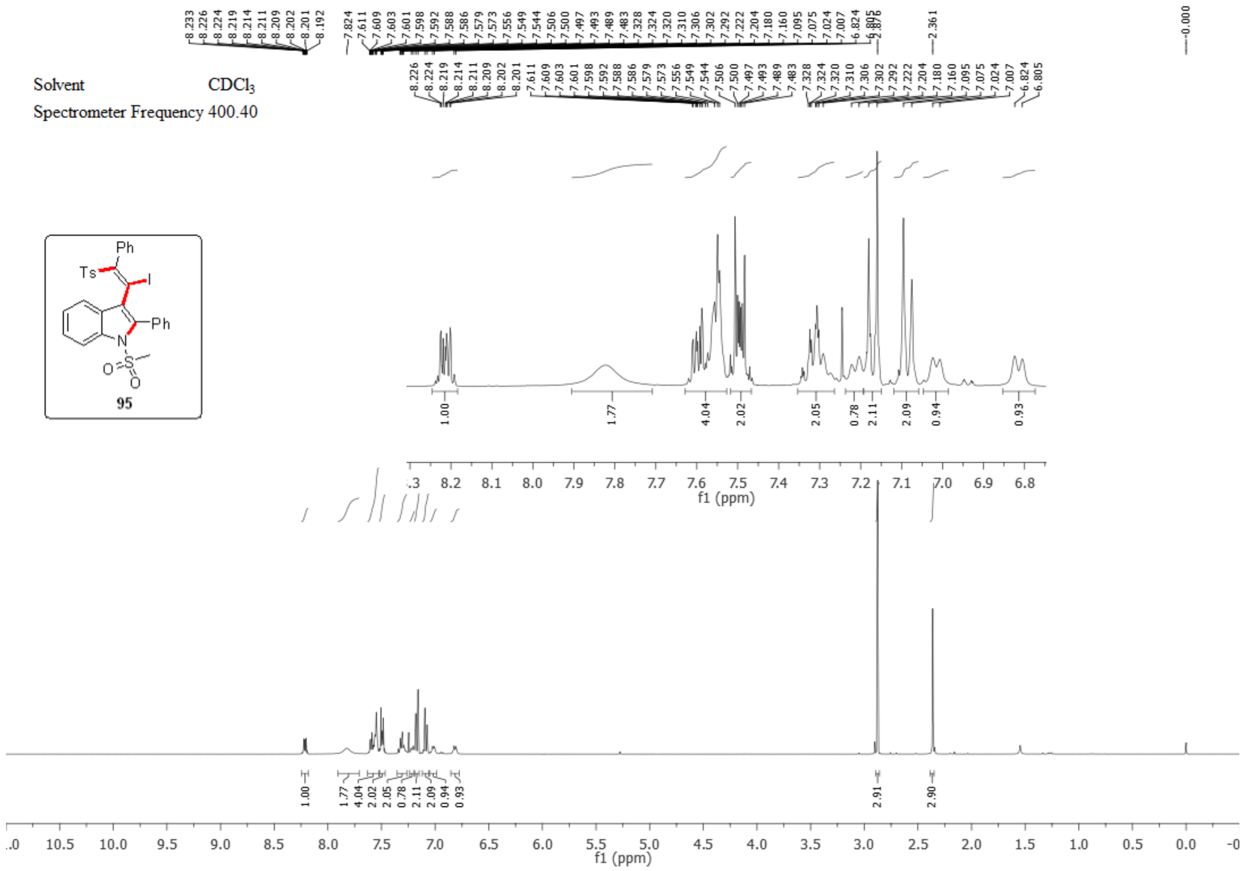

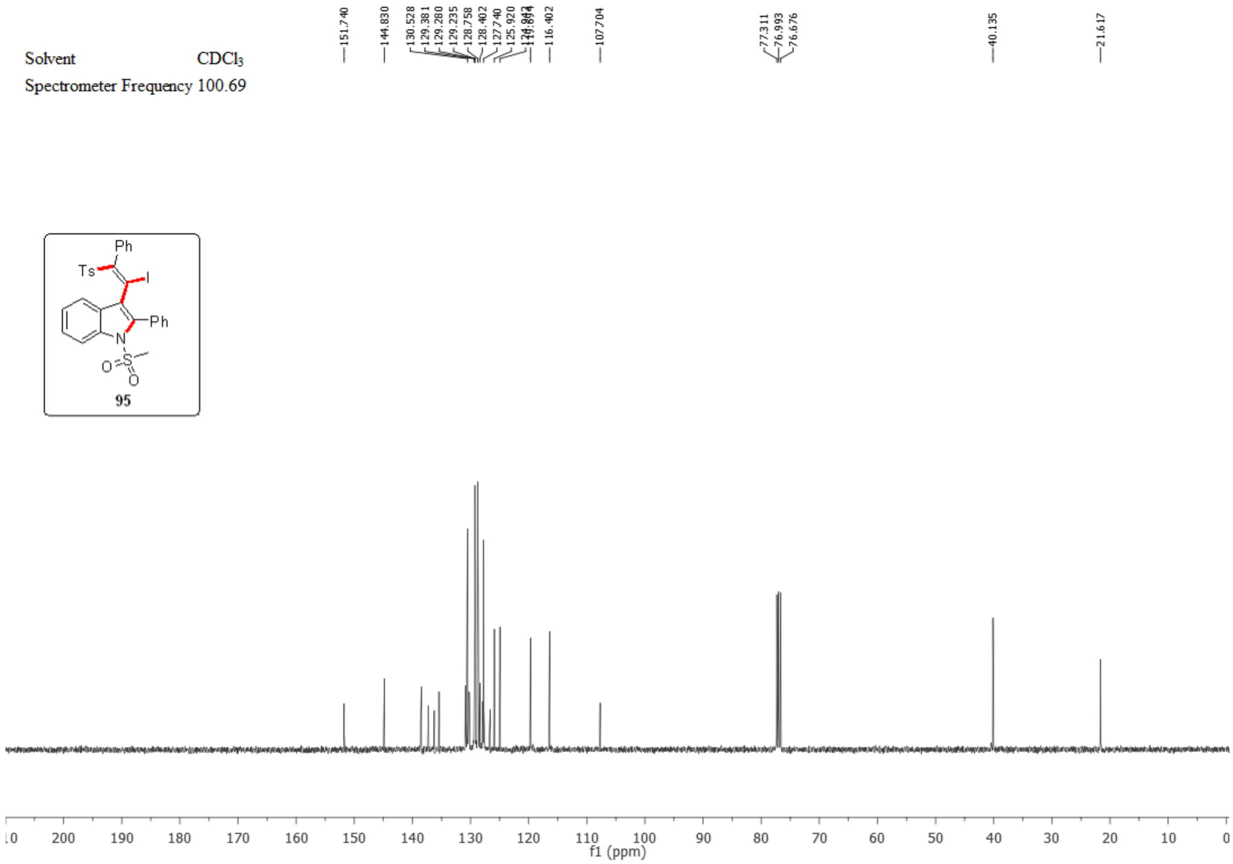

Supplementary Figure 179. ^{1}H (top) and ^{13}C (bottom) NMR spectra of compound **95**.

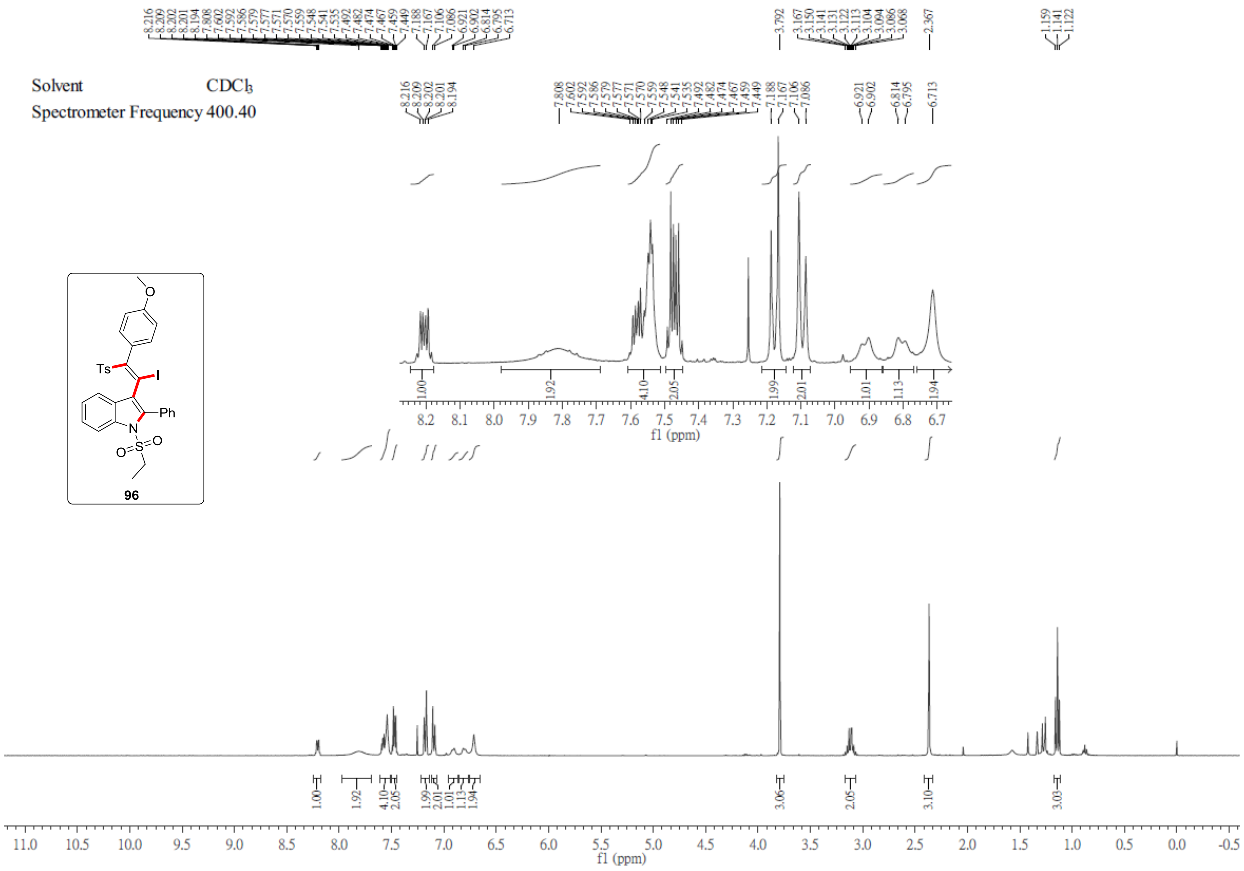

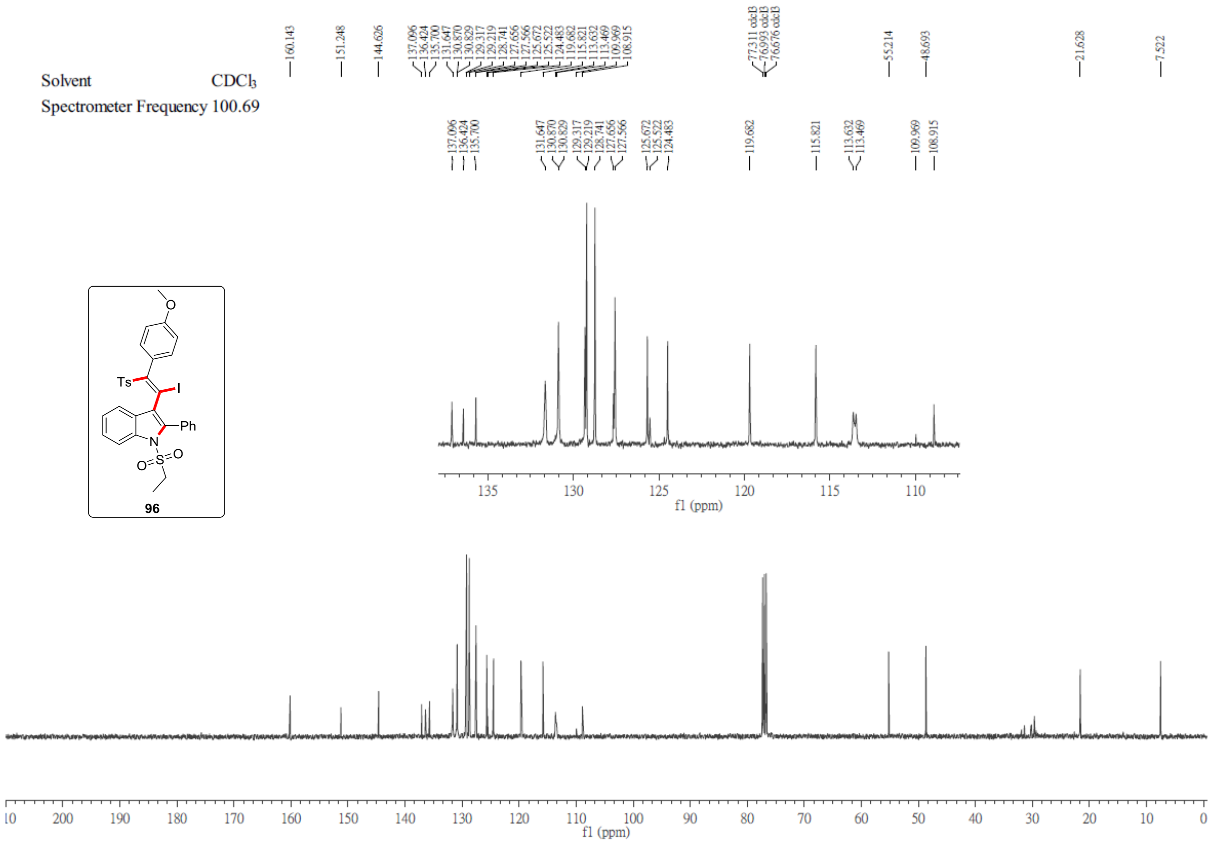

Supplementary Figure 180. ^{1}H (top) and ^{13}C (bottom) NMR spectra of compound **96**.

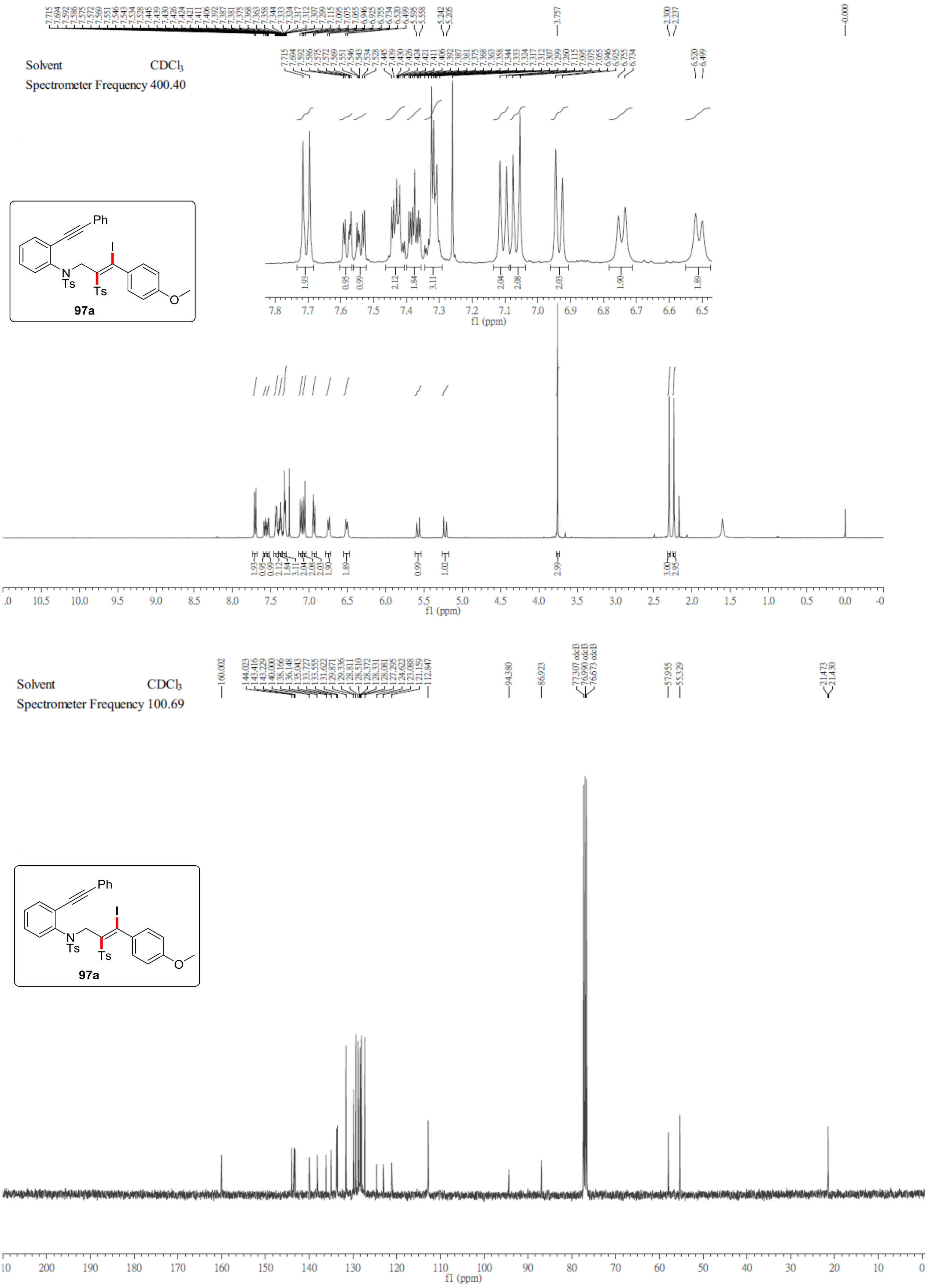

Supplementary Figure 181. ^{1}H (top) and ^{13}C (bottom) NMR spectra of compound **97a**.

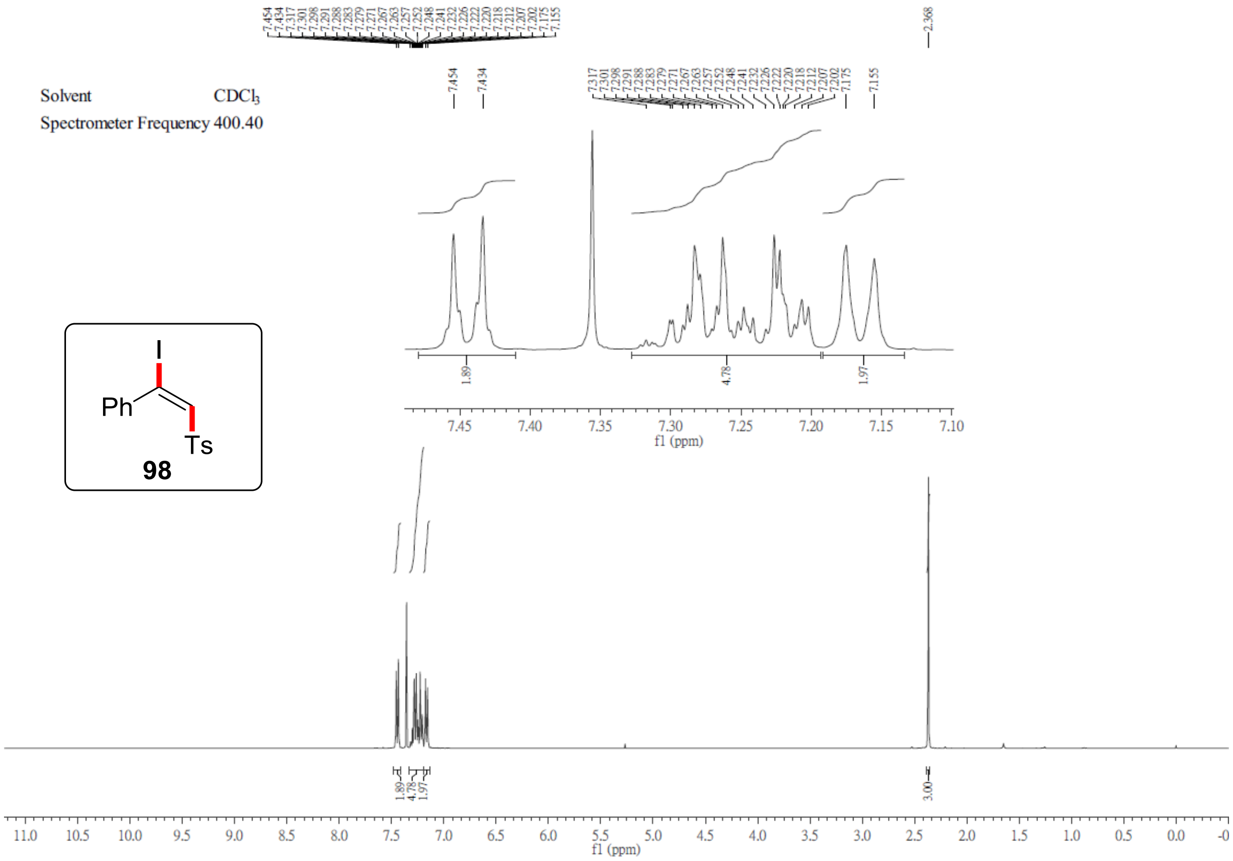

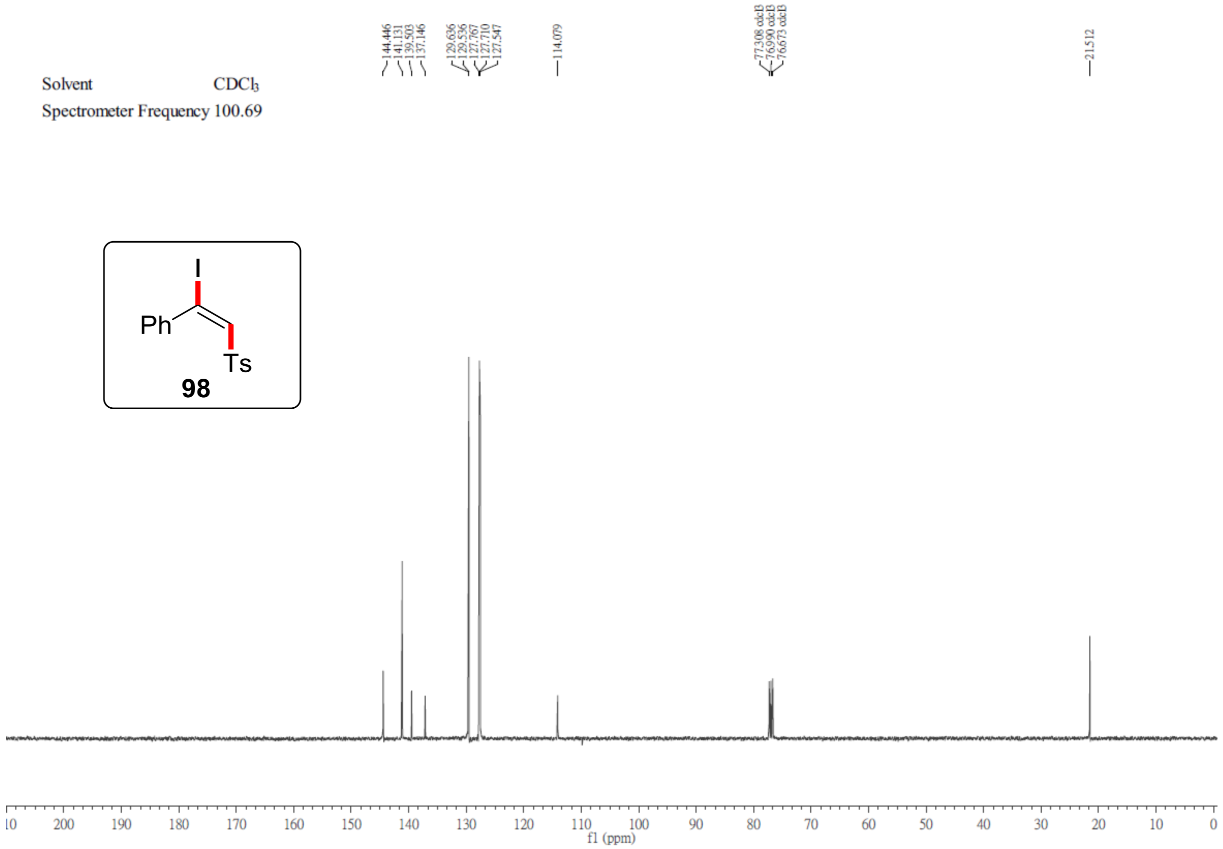

Supplementary Figure 182. ^{1}H (top) and ^{13}C (bottom) NMR spectra of compound **98**.

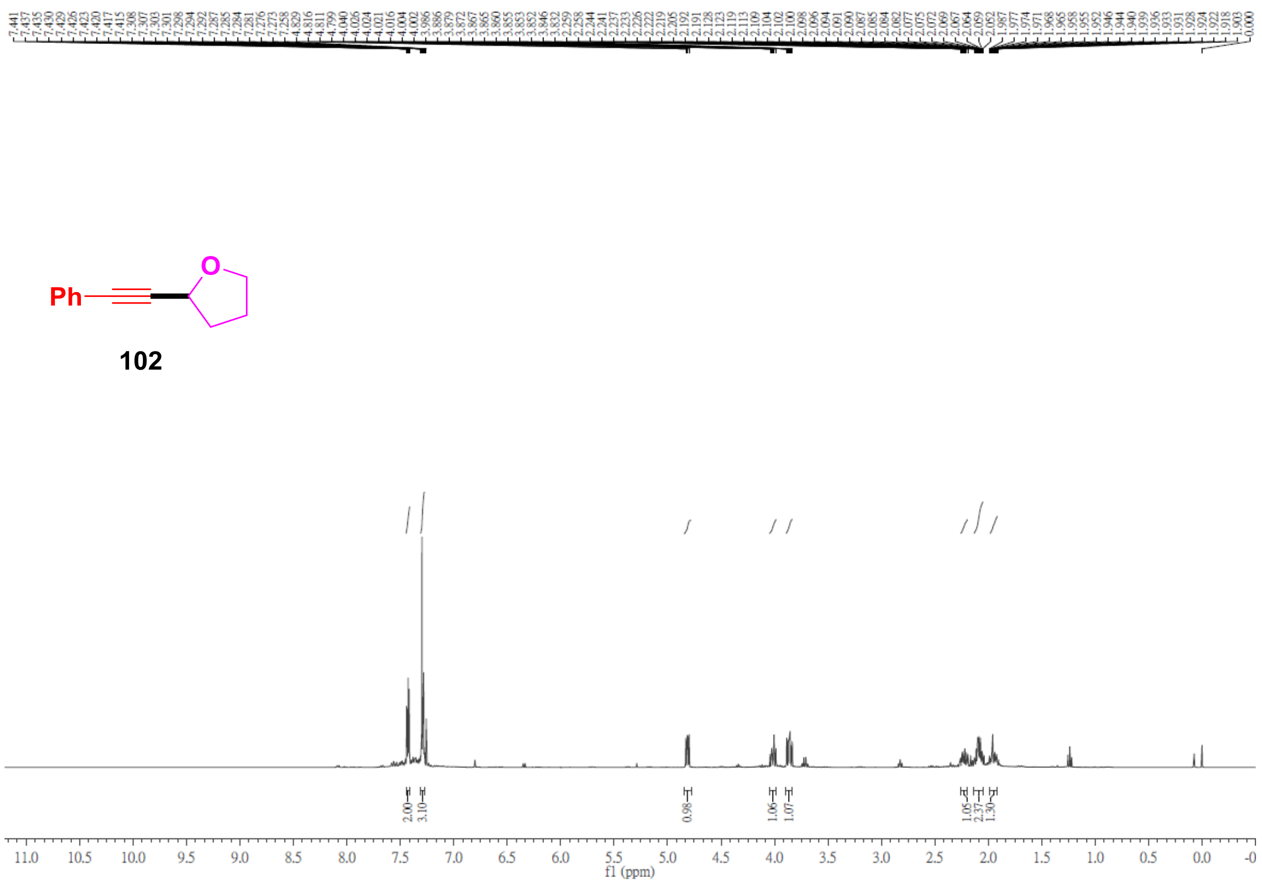

102

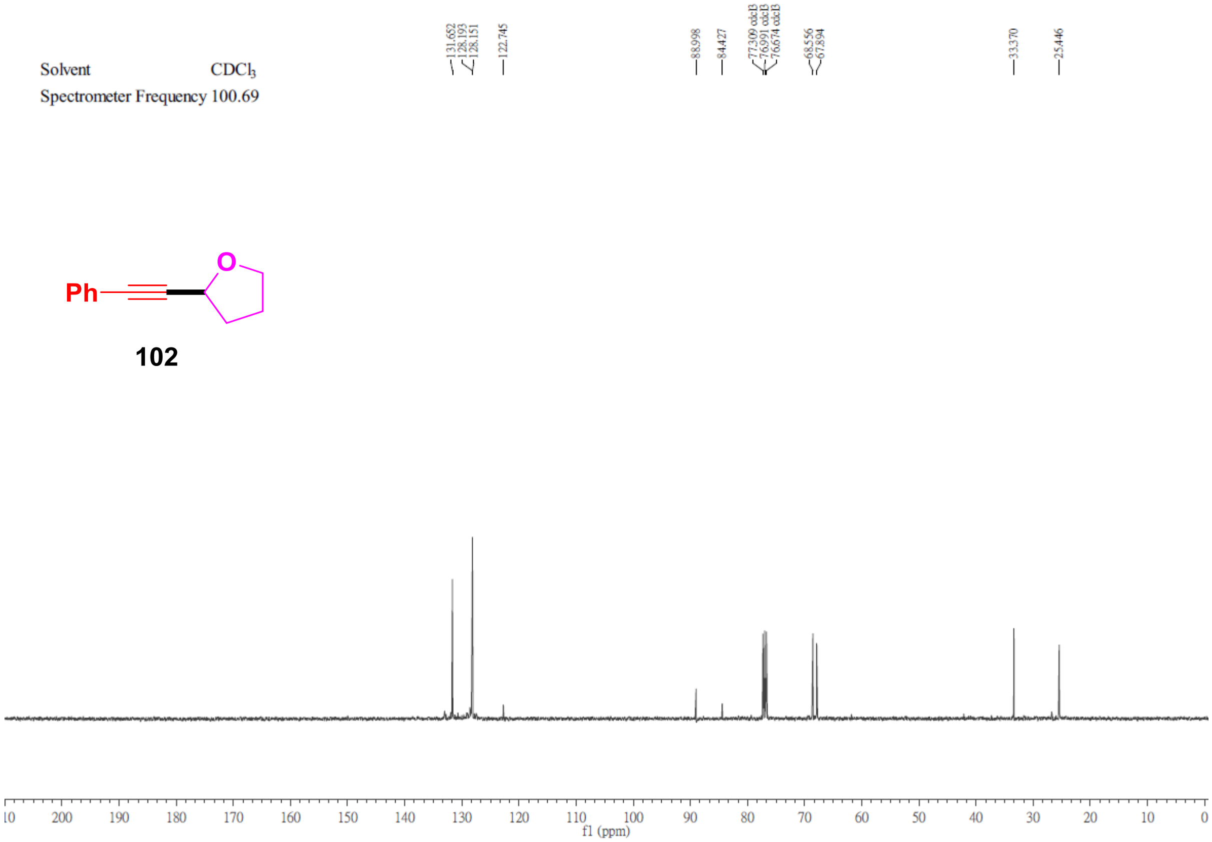

102

Supplementary Figure 183. ^{1}H (top) and ^{13}C (bottom) NMR spectra of compound **102**.

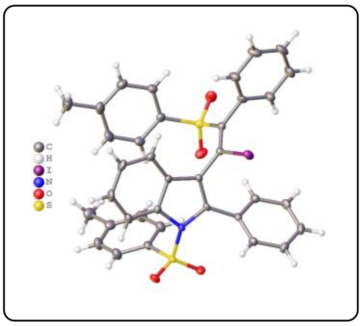

Crystal structure of compound 3

Crystal data and structure refinement for Compound 3

Identification code	K10904-JJW-E
Empirical formula	$C_{36}H_{28}INO_4S_2$
Formula weight	729.61
Temperature/K	113(2)
Crystal system	monoclinic
Space group	$P2_1/c$
a/Å	12.26690(6)
b/Å	10.76837(6)
c/Å	24.12539(13)
α/°	90
β/°	95.1660(5)
γ/°	90
Volume/Å ^{3}	3173.89(3)
Z	4
ρ_{calc}g/cm^3	1.527
μ/mm^{-1}	1.182
F(000)	1472.0
Crystal size/mm^3	0.25 × 0.25 × 0.25
Radiation	Mo Kα (λ = 0.71073)
2Θ range for data collection/°	4.964 to 54.086
Index ranges	-15 ≤ h ≤ 15, -13 ≤ k ≤ 13, -30 ≤ l ≤ 30
Reflections collected	206623
Independent reflections	6833 [R_{int} = 0.0412, R_{sigma} = 0.0122]
Data/restraints/parameters	6833/0/399
Goodness-of-fit on F^2	1.073
Final R indexes [I>=2σ (I)]	R_1 = 0.0209, wR_2 = 0.0526
Final R indexes [all data]	R_1 = 0.0222, wR_2 = 0.0532
Largest diff. peak/hole / e Å $^{-3}$	0.45/-0.44

Fractional Atomic Coordinates (×10^4) and Equivalent Isotropic Displacement Parameters (Å ^{2}×10^3) for compound 3. U_{eq} is defined as 1/3 of of the trace of the orthogonalised U_{IJ} tensor.

Atom	*x*	*y*	*z*	U(eq)
I1	3032.7(2)	5474.8(2)	1820.9(2)	18.96(4)

S1	5316.9(3)	3340.2(4)	4036.5(2)	15.42(8)
S2	1415.3(3)	1823.1(4)	2467.7(2)	16.33(8)
O1	6107.0(9)	2762.6(12)	3721.3(5)	19.8(3)
O2	5662.6(10)	4255.5(12)	4444.3(5)	22.2(3)
O3	2398.6(10)	1264.8(12)	2729.6(6)	24.4(3)
O4	726.2(11)	1093.2(12)	2079.0(6)	26.5(3)
N1	4412.7(11)	4048.8(13)	3581.1(5)	13.1(3)
C1	2765.1(17)	-668(2)	5104.4(9)	30.8(4)
C2	3390.5(15)	337.4(18)	4836.3(8)	23.3(4)
C3	3800.0(16)	1355(2)	5144.0(8)	28.2(4)
C4	4371.7(16)	2289.1(19)	4906.6(7)	25.2(4)
C5	4556.6(13)	2190.0(16)	4346.3(7)	17.4(3)
C6	4160.7(15)	1187.7(18)	4026.1(7)	22.9(4)
C7	3576.1(16)	272.3(18)	4274.7(8)	25.4(4)
C8	3474.7(13)	4631.1(15)	3770.5(7)	14.0(3)
C9	3321.0(14)	5169.6(17)	4282.3(7)	19.3(3)
C10	2322.3(15)	5755.9(18)	4325.8(7)	23.2(4)
C11	1511.1(14)	5805.7(17)	3882.0(8)	22.0(4)
C12	1662.5(13)	5245.6(16)	3379.4(7)	17.1(3)
C13	2653.8(13)	4634.3(15)	3329.0(7)	13.5(3)
C14	3085.4(12)	3996.2(15)	2869.8(6)	12.4(3)
C15	4141.5(13)	3667.4(15)	3019.4(6)	12.8(3)
C16	4931.1(13)	3242.9(15)	2635.1(6)	13.6(3)
C17	4611.8(13)	2314.8(16)	2248.8(7)	16.2(3)
C18	5261.1(14)	2026.0(16)	1825.3(7)	19.6(3)
C19	6243.9(14)	2639.9(16)	1786.6(7)	20.0(3)
C20	6572.8(14)	3553.1(16)	2171.3(7)	19.4(3)
C21	5921.2(13)	3858.8(16)	2592.9(7)	16.2(3)
C22	2533.6(12)	3974.8(15)	2302.5(6)	13.6(3)
C23	1772.3(13)	3185.4(15)	2087.0(7)	14.8(3)
C24	1204.8(13)	3283.5(16)	1517.4(7)	17.3(3)
C25	356.8(15)	4120.1(18)	1400.8(8)	24.1(4)
C26	-143.8(16)	4222.3(19)	862.7(8)	28.7(4)
C27	197.5(16)	3491.4(19)	442.5(8)	28.2(4)
C28	1037.5(17)	2650(2)	555.7(8)	28.9(4)
C29	1545.1(15)	2542.0(18)	1093.0(7)	23.6(4)
C30	630.9(13)	2339.4(16)	2995.6(7)	16.6(3)
C31	1082.5(14)	2317.4(17)	3542.7(7)	21.3(4)
C32	463.8(16)	2753.9(19)	3952.9(7)	25.9(4)
C33	-581.2(16)	3224.3(18)	3824.4(8)	24.8(4)
C34	-1024.6(14)	3218.2(18)	3271.4(8)	23.8(4)
C35	-428.2(14)	2775.0(17)	2855.0(7)	21.0(4)
C36	-1226.5(19)	3750(2)	4272.0(9)	39.5(5)

Anisotropic Displacement Parameters (Å ^{2}×10^3) for compound 3. The Anisotropic displacement factor exponent takes the form: -2π^2[h^{2}a*^{2}U$_{11}$+2hka*b*U$_{12}$+…].

Atom	U_{11}	U_{22}	U_{33}	U_{23}	U_{13}	U_{12}
I1	21.80(6)	18.63(7)	15.81(6)	3.22(4)	-1.90(4)	-4.03(4)
S1	14.09(17)	20.7(2)	10.83(17)	-0.41(15)	-2.52(14)	1.28(15)
S2	14.90(18)	13.61(19)	20.4(2)	-0.21(15)	1.00(15)	-1.83(15)
O1	15.6(5)	26.9(7)	16.6(6)	1.4(5)	0.1(4)	5.9(5)
O2	21.7(6)	29.5(7)	14.2(6)	-3.9(5)	-4.6(5)	-3.9(5)
O3	18.7(6)	19.2(6)	35.6(7)	8.2(5)	3.9(5)	4.1(5)
O4	26.7(7)	23.8(7)	29.2(7)	-9.2(6)	4.4(5)	-10.9(5)
N1	13.9(6)	16.0(7)	9.2(6)	-1.6(5)	-0.3(5)	2.3(5)
C1	30.8(10)	32.3(11)	29.2(10)	11.3(8)	1.4(8)	-1.2(8)
C2	20.2(8)	26.4(10)	23.3(9)	7.8(7)	0.5(7)	4.0(7)
C3	32.9(10)	35.8(11)	16.4(8)	3.0(8)	4.8(7)	-0.9(9)
C4	30.6(9)	28.9(10)	16.0(8)	-1.3(7)	1.6(7)	-1.7(8)
C5	17.8(8)	20.9(9)	13.1(7)	2.2(6)	-1.3(6)	3.2(7)
C6	27.5(9)	25.1(10)	15.8(8)	0.4(7)	0.7(7)	1.4(7)
C7	29.9(10)	21.8(9)	23.8(9)	-0.1(7)	-0.7(8)	-0.9(8)
C8	14.6(7)	13.8(8)	13.9(7)	-0.5(6)	3.2(6)	0.2(6)
C9	23.4(8)	22.0(9)	12.8(8)	-2.9(7)	2.9(6)	-0.9(7)
C10	29.6(9)	23.1(9)	18.5(8)	-5.3(7)	11.5(7)	0.7(7)
C11	19.8(8)	20.6(9)	27.2(9)	-2.2(7)	10.4(7)	3.0(7)
C12	14.6(7)	16.8(8)	20.2(8)	0.8(6)	2.6(6)	0.3(6)
C13	14.9(7)	13.5(8)	12.2(7)	-0.4(6)	2.5(6)	-1.3(6)
C14	14.4(7)	11.9(7)	10.9(7)	-0.7(6)	0.9(6)	-0.3(6)
C15	15.8(7)	12.7(7)	9.6(7)	-1.7(6)	-0.7(6)	-0.6(6)
C16	14.7(7)	14.8(8)	11.0(7)	0.4(6)	0.1(6)	3.7(6)
C17	15.9(7)	15.5(8)	17.1(8)	-1.5(6)	0.2(6)	1.3(6)
C18	25.3(8)	16.7(8)	16.8(8)	-4.4(7)	1.7(7)	4.1(7)
C19	25.2(9)	19.0(9)	17.0(8)	1.0(7)	8.3(7)	5.2(7)
C20	19.4(8)	17.1(8)	22.7(8)	2.5(7)	7.4(7)	0.3(7)
C21	17.5(8)	15.1(8)	15.8(8)	-2.0(6)	0.7(6)	1.1(6)
C22	13.6(7)	14.7(8)	12.6(7)	0.1(6)	0.7(6)	2.2(6)
C23	14.4(7)	14.7(8)	15.1(7)	-1.3(6)	0.8(6)	1.6(6)
C24	16.3(7)	18.0(8)	16.7(8)	-2.6(6)	-3.4(6)	-3.3(6)
C25	24.3(9)	24.1(9)	22.3(9)	-5.3(7)	-5.8(7)	4.1(7)
C26	28.6(10)	25.9(10)	29.1(10)	0.8(8)	-11.7(8)	4.4(8)
C27	33.9(10)	32.1(11)	16.5(8)	1.1(8)	-9.5(7)	-3.9(8)
C28	34.1(10)	35.7(11)	16.2(9)	-6.0(8)	-1.8(7)	1.6(9)
C29	22.9(9)	29.1(10)	17.7(8)	-4.4(7)	-3.1(7)	4.7(7)
C30	16.7(8)	15.2(8)	18.1(8)	2.0(6)	2.3(6)	-2.7(6)
C31	19.6(8)	23.2(9)	20.6(8)	7.3(7)	-1.3(7)	-1.7(7)
C32	30.7(10)	32.0(10)	14.9(8)	5.2(7)	0.9(7)	-5.2(8)
C33	28.8(9)	24.7(10)	22.1(9)	1.1(7)	9.6(7)	-5.6(8)

C34	17.9(8)	25.4(10)	28.2(9)	1.6(8)	3.1(7)	1.1(7)
C35	18.6(8)	25.1(9)	18.8(8)	1.2(7)	-1.6(6)	0.3(7)
C36	41.0(12)	49.3(14)	30.4(11)	-4.9(10)	15.8(9)	0.7(10)

Bond Lengths for compound 3

Atom	Atom	Length/Å	Atom	Atom	Length/Å
I1	C22	2.1124(16)	C14	C15	1.360(2)
S1	O1	1.4273(12)	C14	C22	1.471(2)
S1	O2	1.4297(13)	C15	C16	1.473(2)
S1	N1	1.6733(13)	C16	C17	1.398(2)
S1	C5	1.7572(18)	C16	C21	1.396(2)
S2	O3	1.4416(13)	C17	C18	1.386(2)
S2	O4	1.4383(13)	C18	C19	1.386(3)
S2	C23	1.8056(17)	C19	C20	1.387(3)
S2	C30	1.7544(17)	C20	C21	1.389(2)
N1	C8	1.421(2)	C22	C23	1.333(2)
N1	C15	1.4263(19)	C23	C24	1.487(2)
C1	C2	1.506(3)	C24	C25	1.386(2)
C2	C3	1.392(3)	C24	C29	1.392(2)
C2	C7	1.396(3)	C25	C26	1.390(3)
C3	C4	1.380(3)	C26	C27	1.378(3)
C4	C5	1.395(2)	C27	C28	1.381(3)
C5	C6	1.389(3)	C28	C29	1.391(2)
C6	C7	1.387(3)	C30	C31	1.385(2)
C8	C9	1.392(2)	C30	C35	1.394(2)
C8	C13	1.398(2)	C31	C32	1.383(3)
C9	C10	1.391(3)	C32	C33	1.387(3)
C10	C11	1.396(3)	C33	C34	1.395(3)
C11	C12	1.382(2)	C33	C36	1.506(3)
C12	C13	1.398(2)	C34	C35	1.380(3)
C13	C14	1.444(2)			

Bond Angles for compound 3

Atom	Atom	Atom	Angle/°	Atom	Atom	Atom	Angle/°
O1	S1	O2	119.56(8)	C15	C14	C22	126.18(14)
O1	S1	N1	106.90(7)	N1	C15	C16	125.25(13)
O1	S1	C5	109.35(8)	C14	C15	N1	108.20(13)
O2	S1	N1	105.99(7)	C14	C15	C16	125.35(14)
O2	S1	C5	109.21(8)	C17	C16	C15	118.74(14)
N1	S1	C5	104.77(7)	C21	C16	C15	121.64(14)
O3	S2	C23	109.27(7)	C21	C16	C17	118.98(14)
O3	S2	C30	107.81(8)	C18	C17	C16	120.44(15)

O4	S2	O3	118.51(8)	C19	C18	C17	120.25(16)
O4	S2	C23	105.52(8)	C18	C19	C20	119.71(15)
O4	S2	C30	108.54(8)	C19	C20	C21	120.41(16)
C30	S2	C23	106.61(8)	C20	C21	C16	120.20(15)
C8	N1	S1	119.87(11)	C14	C22	I1	111.58(11)
C8	N1	C15	107.82(12)	C23	C22	I1	120.03(12)
C15	N1	S1	125.21(11)	C23	C22	C14	128.33(15)
C3	C2	C1	120.68(17)	C22	C23	S2	120.87(13)
C3	C2	C7	118.35(17)	C22	C23	C24	124.63(15)
C7	C2	C1	120.97(18)	C24	C23	S2	114.39(12)
C4	C3	C2	121.67(17)	C25	C24	C23	120.94(15)
C3	C4	C5	118.66(18)	C25	C24	C29	119.54(16)
C4	C5	S1	119.81(14)	C29	C24	C23	119.51(15)
C6	C5	S1	118.91(13)	C24	C25	C26	120.11(17)
C6	C5	C4	121.27(17)	C27	C26	C25	120.28(18)
C7	C6	C5	118.71(16)	C26	C27	C28	119.99(17)
C6	C7	C2	121.33(18)	C27	C28	C29	120.18(18)
C9	C8	N1	130.31(15)	C28	C29	C24	119.90(17)
C9	C8	C13	121.92(15)	C31	C30	S2	119.21(13)
C13	C8	N1	107.73(13)	C31	C30	C35	121.45(16)
C10	C9	C8	116.53(16)	C35	C30	S2	119.34(13)
C9	C10	C11	122.12(16)	C32	C31	C30	118.56(16)
C12	C11	C10	120.90(16)	C31	C32	C33	121.32(17)
C11	C12	C13	117.95(16)	C32	C33	C34	119.03(17)
C8	C13	C14	107.13(14)	C32	C33	C36	120.79(18)
C12	C13	C8	120.50(15)	C34	C33	C36	120.18(18)
C12	C13	C14	132.28(15)	C35	C34	C33	120.74(17)
C13	C14	C22	123.46(14)	C34	C35	C30	118.87(16)
C15	C14	C13	109.05(14)				

Torsion Angles for compound 3.

A	B	C	D	Angle/°	A	B	C	D	Angle/°
I1	C22	C23	S2	174.69(8)	C9	C10	C11	C12	1.3(3)
I1	C22	C23	C24	-1.2(2)	C10	C11	C12	C13	-0.3(3)
S1	N1	C8	C9	28.7(2)	C11	C12	C13	C8	-2.1(2)
S1	N1	C8	C13	-153.45(12)	C11	C12	C13	C14	-178.02(17)
S1	N1	C15	C14	149.99(12)	C12	C13	C14	C15	173.85(17)
S1	N1	C15	C16	-42.0(2)	C12	C13	C14	C22	6.2(3)
S1	C5	C6	C7	178.99(14)	C13	C8	C9	C10	-2.5(3)
S2	C23	C24	C25	105.38(17)	C13	C14	C15	N1	1.61(18)
S2	C23	C24	C29	-75.93(18)	C13	C14	C15	C16	-166.35(15)
S2	C30	C31	C32	178.53(14)	C13	C14	C22	I1	89.99(16)

S2	C30	C35	C34	-177.83(14)	C13	C14	C22	C23	-87.2(2)
O1	S1	N1	C8	175.24(12)	C14	C15	C16	C17	-48.7(2)
O1	S1	N1	C15	28.39(15)	C14	C15	C16	C21	121.99(18)
O1	S1	C5	C4	133.40(14)	C14	C22	C23	S2	-8.3(2)
O1	S1	C5	C6	-45.90(16)	C14	C22	C23	C24	175.81(15)
O2	S1	N1	C8	-56.19(14)	C15	N1	C8	C9	-179.26(17)
O2	S1	N1	C15	156.96(13)	C15	N1	C8	C13	-1.44(17)
O2	S1	C5	C4	0.86(17)	C15	C14	C22	I1	-75.47(18)
O2	S1	C5	C6	-178.44(13)	C15	C14	C22	C23	107.3(2)
O3	S2	C23	C22	-41.86(16)	C15	C16	C17	C18	169.91(15)
O3	S2	C23	C24	134.43(12)	C15	C16	C21	C20	-170.42(15)
O3	S2	C30	C31	8.79(16)	C16	C17	C18	C19	1.2(3)
O3	S2	C30	C35	-171.90(14)	C17	C16	C21	C20	0.3(2)
O4	S2	C23	C22	-170.31(14)	C17	C18	C19	C20	-0.5(3)
O4	S2	C23	C24	5.98(14)	C18	C19	C20	C21	-0.3(3)
O4	S2	C30	C31	138.32(14)	C19	C20	C21	C16	0.4(3)
O4	S2	C30	C35	-42.37(17)	C21	C16	C17	C18	-1.0(2)
N1	S1	C5	C4	-112.32(15)	C22	C14	C15	N1	168.81(15)
N1	S1	C5	C6	68.38(15)	C22	C14	C15	C16	0.8(3)
N1	C8	C9	C10	175.10(17)	C22	C23	C24	C25	-78.5(2)
N1	C8	C13	C12	-174.50(14)	C22	C23	C24	C29	100.2(2)
N1	C8	C13	C14	2.37(18)	C23	S2	C30	C31	-108.45(15)
N1	C15	C16	C17	145.33(16)	C23	S2	C30	C35	70.87(15)
N1	C15	C16	C21	-44.0(2)	C23	C24	C25	C26	178.24(17)
C1	C2	C3	C4	-179.75(18)	C23	C24	C29	C28	-178.34(17)
C1	C2	C7	C6	-179.33(18)	C24	C25	C26	C27	0.2(3)
C2	C3	C4	C5	-1.2(3)	C25	C24	C29	C28	0.4(3)
C3	C2	C7	C6	0.5(3)	C25	C26	C27	C28	0.2(3)
C3	C4	C5	S1	-178.10(15)	C26	C27	C28	C29	-0.3(3)
C3	C4	C5	C6	1.2(3)	C27	C28	C29	C24	0.0(3)
C4	C5	C6	C7	-0.3(3)	C29	C24	C25	C26	-0.5(3)
C5	S1	N1	C8	59.26(14)	C30	S2	C23	C22	74.40(15)
C5	S1	N1	C15	-87.60(14)	C30	S2	C23	C24	-109.31(13)
C5	C6	C7	C2	-0.6(3)	C30	C31	C32	C33	-1.0(3)
C7	C2	C3	C4	0.4(3)	C31	C30	C35	C34	1.5(3)
C8	N1	C15	C14	-0.13(18)	C31	C32	C33	C34	2.0(3)

C8	N1	C15	C16	167.85(15)	C31	C32	C33	C36	-177.19(19)
C8	C9	C10	C11	0.1(3)	C32	C33	C34	C35	-1.3(3)
C8	C13	C14	C15	-2.51(18)	C33	C34	C35	C30	-0.4(3)
C8	C13	C14	C22	-170.13(15)	C35	C30	C31	C32	-0.8(3)
C9	C8	C13	C12	3.5(2)	C36	C33	C34	C35	177.93(19)
C9	C8	C13	C14	-179.58(15)					

Hydrogen Atom Coordinates (Å ×10^4) and Isotropic Displacement Parameters (Å^2×10^3) for compound 3.

**Atom**	***x***	***y***	***z***	**U(eq)**
H1A	2553.06	-1297.46	4834.18	46
H1B	2123.22	-318.2	5243.24	46
H1C	3220.49	-1029.09	5406.65	46
H3	3685.57	1406.33	5519.19	34
H4	4628.43	2971.05	5115.92	30
H6	4285.28	1131.77	3652.36	27
H7	3302.13	-398.54	4062.86	30
H9	3858.33	5138.86	4579.59	23
H10	2190.76	6126.83	4661.44	28
H11	859.62	6221.54	3925.12	26
H12	1121.51	5274.51	3083.72	21
H17	3959.47	1888.54	2276.21	19
H18	5036.08	1417.76	1566.19	24
H19	6681.2	2440.55	1503.91	24
H20	7233.96	3963.21	2146.79	23
H21	6145.28	4475.71	2847.91	19
H25	122.3	4614.02	1683.19	29
H26	-711.49	4786.21	785.91	34
H27	-137.95	3564.83	82.64	34
H28	1264.75	2154.77	272.12	35
H29	2110.84	1974.9	1168.45	28
H31	1786.72	2015.54	3632.2	26
H32	754.08	2731.76	4322.46	31
H34	-1730.25	3516.2	3182.21	29
H35	-727.57	2767.09	2486.93	25
H36A	-766.64	3801.01	4614.91	59
H36B	-1839.64	3220.36	4321.19	59
H36C	-1484.61	4565.07	4165.21	59

Experimental

Single crystals of $C_{36}H_{28}INO_4S_2$ **[K10904-JJW-E]** were **[]**. A suitable crystal was selected and **[]** on a **XtaLAB Pro II AFC12 (RINC): Kappa single** diffractometer. The crystal was kept at 113(2) K during data collection. Using Olex2 [1], the structure was solved with the ShelXT [2] structure solution

program using Intrinsic Phasing and refined with the ShelXL [3] refinement package using Least Squares minimisation.

1. Dolomanov, O.V., Bourhis, L.J., Gildea, R.J, Howard, J.A.K. & Puschmann, H. (2009), J. Appl. Cryst. 42, 339-341.
2. Sheldrick, G.M. (2015). Acta Cryst. A71, 3-8.
3. Sheldrick, G.M. (2015). Acta Cryst. C71, 3-8.

Crystal structure determination of [K10904-JJW-E]

Crystal Data for $C_{36}H_{28}INO_4S_2$ (M =729.61 g/mol): monoclinic, space group P2$_1$/c (no. 14), a = 12.26690(6) Å, b = 10.76837(6) Å, c = 24.12539(13) Å, β = 95.1660(5)°, V = 3173.89(3) Å^3, Z = 4, T = 113(2) K, μ(Mo Kα) = 1.182 mm^{-1}, $Dcalc$ = 1.527 g/cm^3, 206623 reflections measured (4.964° ≤ 2Θ ≤ 54.086°), 6833 unique (R_{int} = 0.0412, R_{sigma} = 0.0122) which were used in all calculations. The final R_1 was 0.0209 (I > 2σ(I)) and wR_2 was 0.0532 (all data).

Refinement model description

Number of restraints - 0, number of constraints - unknown.

Details:

```
1. Fixed Uiso
 At 1.2 times of:
  All C(H) groups
 At 1.5 times of:
  All C(H,H,H) groups
2.a Aromatic/amide H refined with riding coordinates:
 C3(H3), C4(H4), C6(H6), C7(H7), C9(H9), C10(H10), C11(H11), C12(H12),
 C17(H17), C18(H18), C19(H19), C20(H20), C21(H21), C25(H25), C26(H26),
C27(H27),
  C28(H28), C29(H29), C31(H31), C32(H32), C34(H34), C35(H35)
2.b Idealised Me refined as rotating group:
 C1(H1A,H1B,H1C), C36(H36A,H36B,H36C)
```

This report has been created with Olex2, compiled on 2019.04.23 svn.r3594 for Rigaku Oxford Diffraction. Please let us know if there are any errors or if you would like to have additional features.

checkCIF/PLATON report

Structure factors have been supplied for datablock(s) I

THIS REPORT IS FOR GUIDANCE ONLY. IF USED AS PART OF A REVIEW PROCEDURE FOR PUBLICATION, IT SHOULD NOT REPLACE THE EXPERTISE OF AN EXPERIENCED CRYSTALLOGRAPHIC REFEREE.

No syntax errors found. CIF dictionary Interpreting this report

Datablock: I

```
Bond precision:  C-C = 0.0024 A          Wavelength=0.71073

Cell:           a=10.5947(2)      b=13.5331(3)     c=13.8316(2)
                alpha=79.801(2)   beta=82.524(1)   gamma=73.496(2)
Temperature:    113 K

                Calculated                 Reported
Volume          1864.71(6)                 1864.71(6)
Space group     P -1                       P -1
Hall group      -P 1                       -P 1
Moiety formula  C43 H35 N O7 S3            C43 H35 N O7 S3
Sum formula     C43 H35 N O7 S3            C43 H35 N O7 S3
Mr              773.90                     773.90
Dx,g cm-3       1.378                      1.378
Z               2                          2
Mu (mm-1)       0.253                      0.253
F000            808.0                      808.0
F000'           809.10
h,k,lmax        13,17,17                   13,17,17
Nref            8198                       7774
Tmin,Tmax       0.941,0.951                0.552,1.000
Tmin'           0.939

Correction method= # Reported T Limits: Tmin=0.552 Tmax=1.000
AbsCorr = MULTI-SCAN

Data completeness= 0.948          Theta(max)= 27.037

R(reflections)= 0.0357( 6624)     wR2(reflections)= 0.0946( 7774)

S = 1.069                  Npar= 490
```

The following ALERTS were generated. Each ALERT has the format
test-name_ALERT_alert-type_alert-level.
Click on the hyperlinks for more details of the test.

```
Alert level C
PLAT761_ALERT_1_C CIF Contains no X-H Bonds ......................     Please Check
PLAT762_ALERT_1_C CIF Contains no X-Y-H or H-Y-H Angles ..........     Please Check
PLAT911_ALERT_3_C Missing FCF Refl Between Thmin & STh/L=    0.600         16 Report
```

```
Alert level G
PLAT910_ALERT_3_G Missing # of FCF Reflection(s) Below Theta(Min).          2 Note
PLAT912_ALERT_4_G Missing # of FCF Reflections Above STh/L=  0.600        406 Note
PLAT933_ALERT_2_G Number of OMIT Records in Embedded .res File ...          6 Note
PLAT941_ALERT_3_G Average HKL Measurement Multiplicity ...........        4.4 Low
PLAT978_ALERT_2_G Number C-C Bonds with Positive Residual Density.         14 Info
```

```
   0 ALERT level A = Most likely a serious problem - resolve or explain
   0 ALERT level B = A potentially serious problem, consider carefully
   3 ALERT level C = Check. Ensure it is not caused by an omission or oversight
   5 ALERT level G = General information/check it is not something unexpected

   2 ALERT type 1 CIF construction/syntax error, inconsistent or missing data
   2 ALERT type 2 Indicator that the structure model may be wrong or deficient
   3 ALERT type 3 Indicator that the structure quality may be low
   1 ALERT type 4 Improvement, methodology, query or suggestion
   0 ALERT type 5 Informative message, check
```

checkCIF publication errors

```
Alert level A
PUBL004_ALERT_1_A  The contact author's name and address are missing,
            _publ_contact_author_name and _publ_contact_author_address.
PUBL005_ALERT_1_A  _publ_contact_author_email, _publ_contact_author_fax and
            _publ_contact_author_phone are all missing.
            At least one of these should be present.
PUBL006_ALERT_1_A  _publ_requested_journal is missing
            e.g. 'Acta Crystallographica Section C'
PUBL008_ALERT_1_A  _publ_section_title is missing. Title of paper.
PUBL009_ALERT_1_A  _publ_author_name is missing. List of author(s) name(s).
PUBL010_ALERT_1_A  _publ_author_address is missing. Author(s) address(es).
PUBL012_ALERT_1_A  _publ_section_abstract is missing.
            Abstract of paper in English.
```

```
   7 ALERT level A = Data missing that is essential or data in wrong format
   0 ALERT level G = General alerts. Data that may be required is missing
```

Publication of your CIF

You should attempt to resolve as many as possible of the alerts in all categories. Often the minor alerts point to easily fixed oversights, errors and omissions in your CIF or refinement strategy, so attention to these fine details can be worthwhile. In order to resolve some of the more serious problems it may be necessary to carry out additional measurements or structure refinements. However, the nature of your study may justify the reported deviations from journal submission requirements and the more serious of these should be commented upon in the discussion or experimental section of a paper or in the "special_details" fields of the CIF. *checkCIF* was carefully designed to identify outliers and unusual parameters, but every test has its limitations and alerts that are not important in a particular case may appear. Conversely, the absence of alerts does not guarantee there are no aspects of the results needing attention. It is up to the individual to critically assess their own results and, if necessary, seek expert advice.

If level A alerts remain, which you believe to be justified deviations, and you intend to submit this CIF for publication in a journal, you should additionally insert an explanation in your CIF using the Validation Reply Form (VRF) below. This will allow your explanation to be considered as part of the review process.

Validation response form

Please find below a validation response form (VRF) that can be filled in and pasted into your CIF.

```
# start Validation Reply Form
_vrf_PUBL004_GLOBAL
;
PROBLEM: The contact author’s name and address are missing,
RESPONSE: ...
;
_vrf_PUBL005_GLOBAL
;
PROBLEM: _publ_contact_author_email, _publ_contact_author_fax and
RESPONSE: ...
;
_vrf_PUBL006_GLOBAL
;
PROBLEM: _publ_requested_journal is missing
RESPONSE: ...
;
_vrf_PUBL008_GLOBAL
;
PROBLEM: _publ_section_title is missing. Title of paper.
RESPONSE: ...
;
_vrf_PUBL009_GLOBAL
;
PROBLEM: _publ_author_name is missing. List of author(s) name(s).
RESPONSE: ...
;
_vrf_PUBL010_GLOBAL
;
PROBLEM: _publ_author_address is missing. Author(s) address(es).
RESPONSE: ...
;
_vrf_PUBL012_GLOBAL
;
```

```
PROBLEM: _publ_section_abstract is missing.
RESPONSE: ...
;
# end Validation Reply Form
```

If you wish to submit your CIF for publication in Acta Crystallographica Section C or E, you should upload your CIF via the web. If you wish to submit your CIF for publication in IUCrData you should upload your CIF via the web. If your CIF is to form part of a submission to another IUCr journal, you will be asked, either during electronic submission or by the Co-editor handling your paper, to upload your CIF via our web site.

PLATON version of 22/04/2020; check.def file version of 09/03/2020

Datablock I - ellipsoid plot

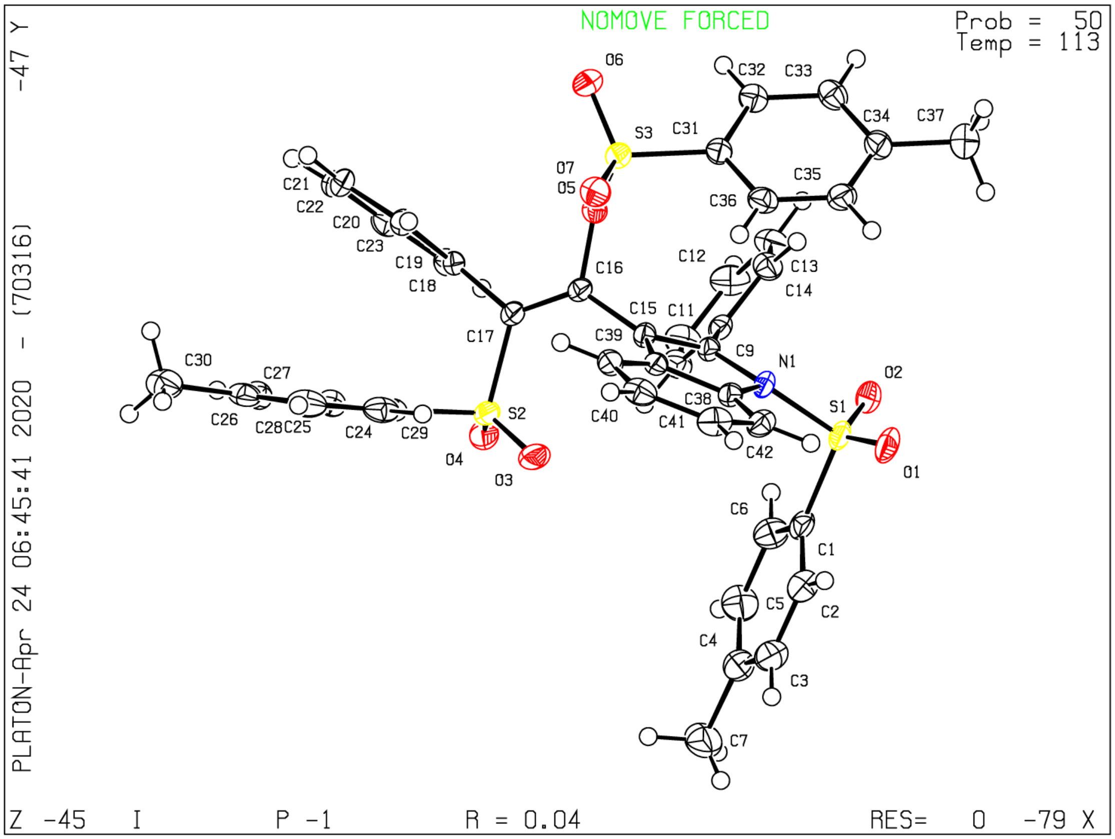

checkCIF/PLATON report

Structure factors have been supplied for datablock(s) I

THIS REPORT IS FOR GUIDANCE ONLY. IF USED AS PART OF A REVIEW PROCEDURE FOR PUBLICATION, IT SHOULD NOT REPLACE THE EXPERTISE OF AN EXPERIENCED CRYSTALLOGRAPHIC REFEREE.

No syntax errors found. CIF dictionary Interpreting this report

Datablock: I

```
Bond precision:  C-C = 0.0038 A          Wavelength=0.71073

Cell:         a=8.21319(13)      b=12.9858(2)      c=15.3869(3)
              alpha=66.1976(15)  beta=78.8697(14)  gamma=88.8876(13)
Temperature:  113 K

                Calculated                  Reported
Volume          1470.13(5)                  1470.13(4)
Space group     P -1                        P -1
Hall group      -P 1                        -P 1
Moiety formula  C33 H28 I N O4 S2           2(C33 H28 I N O4 S2)
Sum formula     C33 H28 I N O4 S2           C66 H56 I2 N2 O8 S4
Mr              693.58                      1387.16
Dx,g cm-3       1.567                       1.567
Z               2                           1
Mu (mm-1)       1.271                       1.271
F000            700.0                       700.0
F000'           699.81
h,k,lmax        9,15,18                     9,15,18
Nref            5179                        5152
Tmin,Tmax       0.859,0.881                 0.579,1.000
Tmin'           0.728

Correction method= # Reported T Limits: Tmin=0.579 Tmax=1.000
AbsCorr = MULTI-SCAN

Data completeness= 0.995          Theta(max)= 24.999

R(reflections)= 0.0242( 4674)     wR2(reflections)= 0.0568( 5152)

S = 1.049                  Npar= 372
```

The following ALERTS were generated. Each ALERT has the format
test-name_ALERT_alert-type_alert-level.
Click on the hyperlinks for more details of the test.

Alert level C

```
PLAT761_ALERT_1_C CIF Contains no X-H Bonds ......................     Please Check
PLAT762_ALERT_1_C CIF Contains no X-Y-H or H-Y-H Angles ..........     Please Check
PLAT911_ALERT_3_C Missing FCF Refl Between Thmin & STh/L=    0.595         25 Report
```

Alert level G

```
PLAT042_ALERT_1_G Calc. and Reported MoietyFormula Strings  Differ     Please Check
PLAT045_ALERT_1_G Calculated and Reported Z Differ by a Factor ...       2.00 Check
PLAT909_ALERT_3_G Percentage of I>2sig(I) Data at Theta(Max) Still        81% Note
PLAT910_ALERT_3_G Missing # of FCF Reflection(s) Below Theta(Min).          3 Note
PLAT933_ALERT_2_G Number of OMIT Records in Embedded .res File ...          3 Note
PLAT978_ALERT_2_G Number C-C Bonds with Positive Residual Density.         14 Info
```

```
   0 ALERT level A = Most likely a serious problem - resolve or explain
   0 ALERT level B = A potentially serious problem, consider carefully
   3 ALERT level C = Check. Ensure it is not caused by an omission or oversight
   6 ALERT level G = General information/check it is not something unexpected

   4 ALERT type 1 CIF construction/syntax error, inconsistent or missing data
   2 ALERT type 2 Indicator that the structure model may be wrong or deficient
   3 ALERT type 3 Indicator that the structure quality may be low
   0 ALERT type 4 Improvement, methodology, query or suggestion
   0 ALERT type 5 Informative message, check
```

checkCIF publication errors

Alert level A

```
PUBL004_ALERT_1_A  The contact author's name and address are missing,
            _publ_contact_author_name and _publ_contact_author_address.
PUBL005_ALERT_1_A  _publ_contact_author_email, _publ_contact_author_fax and
            _publ_contact_author_phone are all missing.
            At least one of these should be present.
PUBL006_ALERT_1_A  _publ_requested_journal is missing
            e.g. 'Acta Crystallographica Section C'
PUBL008_ALERT_1_A  _publ_section_title is missing. Title of paper.
PUBL009_ALERT_1_A  _publ_author_name is missing. List of author(s) name(s).
PUBL010_ALERT_1_A  _publ_author_address is missing. Author(s) address(es).
PUBL012_ALERT_1_A  _publ_section_abstract is missing.
            Abstract of paper in English.
```

```
   7 ALERT level A = Data missing that is essential or data in wrong format
   0 ALERT level G = General alerts. Data that may be required is missing
```

Publication of your CIF

You should attempt to resolve as many as possible of the alerts in all categories. Often the minor alerts point to easily fixed oversights, errors and omissions in your CIF or refinement strategy, so attention to these fine details can be worthwhile. In order to resolve some of the more serious problems it may be necessary to carry out additional measurements or structure refinements. However, the nature of your study may justify the reported deviations from journal submission requirements and the more serious of these should be commented upon in the discussion or experimental section of a paper or in the "special_details" fields of the CIF. *checkCIF* was carefully designed to identify outliers and unusual parameters, but every test has its limitations and alerts that are not important in a particular case may appear. Conversely, the absence of alerts does not guarantee there are no aspects of the results needing attention. It is up to the individual to critically assess their own results and, if necessary, seek expert advice.

If level A alerts remain, which you believe to be justified deviations, and you intend to submit this CIF for publication in a journal, you should additionally insert an explanation in your CIF using the Validation Reply Form (VRF) below. This will allow your explanation to be considered as part of the review process.

Validation response form

Please find below a validation response form (VRF) that can be filled in and pasted into your CIF.

```
# start Validation Reply Form
_vrf_PUBL004_GLOBAL
;
PROBLEM: The contact author's name and address are missing,
RESPONSE: ...
;
_vrf_PUBL005_GLOBAL
;
PROBLEM: _publ_contact_author_email, _publ_contact_author_fax and
RESPONSE: ...
;
_vrf_PUBL006_GLOBAL
;
PROBLEM: _publ_requested_journal is missing
RESPONSE: ...
;
_vrf_PUBL008_GLOBAL
;
PROBLEM: _publ_section_title is missing. Title of paper.
RESPONSE: ...
;
_vrf_PUBL009_GLOBAL
;
PROBLEM: _publ_author_name is missing. List of author(s) name(s).
RESPONSE: ...
;
_vrf_PUBL010_GLOBAL
;
PROBLEM: _publ_author_address is missing. Author(s) address(es).
RESPONSE: ...
;
_vrf_PUBL012_GLOBAL
;
```

```
PROBLEM: _publ_section_abstract is missing.
RESPONSE: ...
;
# end Validation Reply Form
```

If you wish to submit your CIF for publication in Acta Crystallographica Section C or E, you should upload your CIF via the web. If you wish to submit your CIF for publication in IUCrData you should upload your CIF via the web. If your CIF is to form part of a submission to another IUCr journal, you will be asked, either during electronic submission or by the Co-editor handling your paper, to upload your CIF via our web site.

PLATON version of 10/08/2020; check.def file version of 06/08/2020

Datablock I - ellipsoid plot

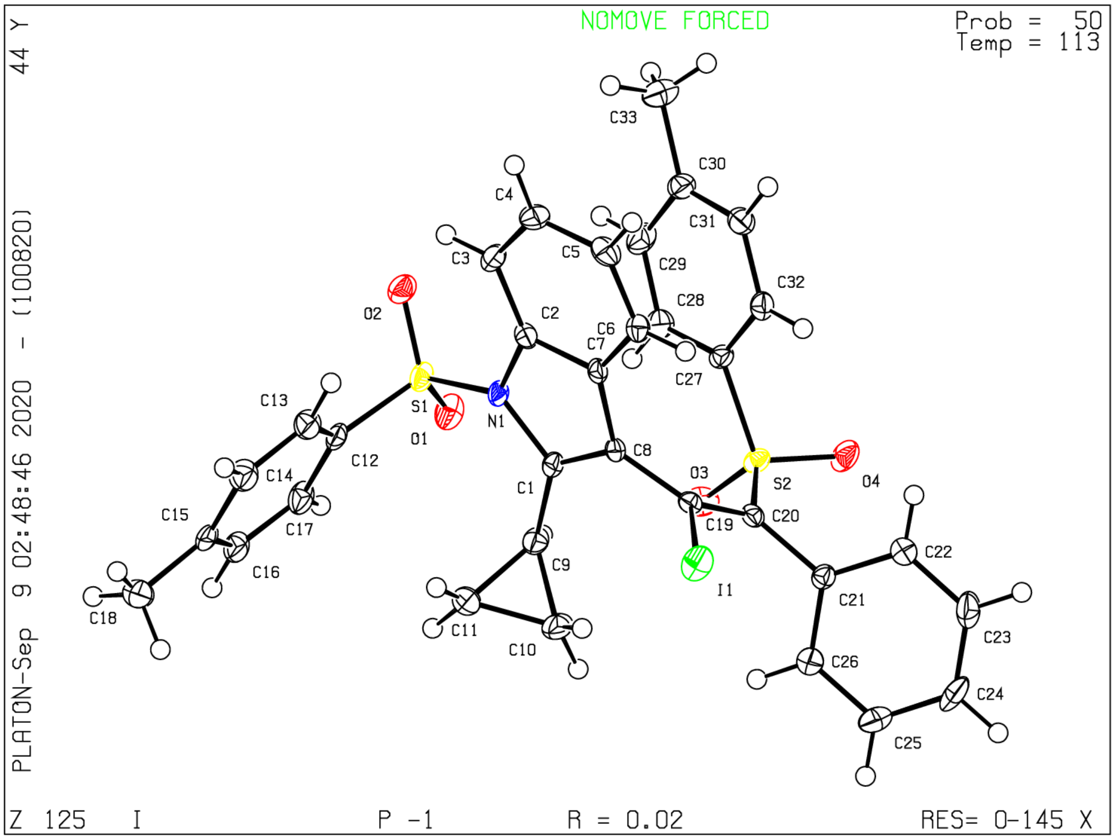

checkCIF/PLATON report

You have not supplied any structure factors. As a result the full set of tests cannot be run.

THIS REPORT IS FOR GUIDANCE ONLY. IF USED AS PART OF A REVIEW PROCEDURE FOR PUBLICATION, IT SHOULD NOT REPLACE THE EXPERTISE OF AN EXPERIENCED CRYSTALLOGRAPHIC REFEREE.

No syntax errors found.   CIF dictionary   Interpreting this report

Datablock: I

```
Bond precision:  C-C = 0.0031 A          Wavelength=0.71073

Cell:           a=11.2616(1)       b=11.6601(2)      c=13.2822(2)
                alpha=101.418(1)   beta=113.463(1)   gamma=106.594(1)
Temperature:    113 K
```

	Calculated	Reported
Volume	1433.67(4)	1433.67(4)
Space group	P -1	P -1
Hall group	-P 1	-P 1
Moiety formula	C31 H26 I N O4 S2	0.33(C31 H26 I N O4 S2)
Sum formula	C31 H26 I N O4 S2	C10.33 H8.67 I0.33 N0.33 O1.33 S0.67
Mr	667.55	222.52
Dx,g cm-3	1.546	1.546
Z	2	6
Mu (mm-1)	1.300	1.300
F000	672.0	672.0
F000’	671.80	
h,k,lmax	13,13,15	13,13,15
Nref	5050	5042
Tmin,Tmax	0.633,0.723	0.547,1.000
Tmin’	0.589	

```
Correction method= # Reported T Limits: Tmin=0.547 Tmax=1.000
AbsCorr = MULTI-SCAN

Data completeness= 0.998          Theta(max)= 24.999

R(reflections)= 0.0205( 4728)     wR2(reflections)= 0.0534( 5042)

S = 1.047                  Npar= 354
```

```
The following ALERTS were generated. Each ALERT has the format
       test-name_ALERT_alert-type_alert-level.
Click on the hyperlinks for more details of the test.
```

Alert level C

```
PLAT761_ALERT_1_C CIF Contains no X-H Bonds ......................     Please Check
PLAT762_ALERT_1_C CIF Contains no X-Y-H or H-Y-H Angles ..........     Please Check
```

Alert level G

```
FORMU01_ALERT_1_G  There is a discrepancy between the atom counts in the
            _chemical_formula_sum and _chemical_formula_moiety. This is
            usually due to the moiety formula being in the wrong format.
            Atom count from _chemical_formula_sum:   C10.33 H8.67 I0.33 N0.33 O1.3
            Atom count from _chemical_formula_moiety:C10.23 H8.58 I0.33 N0.33 O1.3
CELLZ01_ALERT_1_G Difference between formula and atom_site contents detected.
CELLZ01_ALERT_1_G ALERT: check formula stoichiometry or atom site occupancies.
           From the CIF: _cell_formula_units_Z    6
           From the CIF: _chemical_formula_sum  C10.33 H8.67 I0.33 N0.33 O1.33 S0.
           TEST: Compare cell contents of formula and atom_site data

           atom    Z*formula  cif sites diff
           C         61.98     62.00   -0.02
           H         52.02     52.00    0.02
           I          1.98      2.00   -0.02
           N          1.98      2.00   -0.02
           O          7.98      8.00   -0.02
           S          4.02      4.00    0.02
PLAT003_ALERT_2_G Number of Uiso or Uij Restrained non-H Atoms ...         39 Report
PLAT005_ALERT_5_G No Embedded Refinement Details Found  in the CIF     Please Do !
PLAT042_ALERT_1_G Calc. and Reported Moiety Formula Strings Differ     Please Check
PLAT045_ALERT_1_G Calculated and Reported Z Differ by a Factor ...       0.33 Check
PLAT154_ALERT_1_G The s.u.'s on the Cell Angles are Equal ..(Note)      0.001 Degree
PLAT860_ALERT_3_G Number of Least-Squares Restraints ............       1134 Note
```

```
   0 ALERT level A = Most likely a serious problem - resolve or explain
   0 ALERT level B = A potentially serious problem, consider carefully
   2 ALERT level C = Check. Ensure it is not caused by an omission or oversight
   9 ALERT level G = General information/check it is not something unexpected

   8 ALERT type 1 CIF construction/syntax error, inconsistent or missing data
   1 ALERT type 2 Indicator that the structure model may be wrong or deficient
   1 ALERT type 3 Indicator that the structure quality may be low
   0 ALERT type 4 Improvement, methodology, query or suggestion
   1 ALERT type 5 Informative message, check
```

---

checkCIF publication errors

Alert level A

```
PUBL004_ALERT_1_A  The contact author's name and address are missing,
            _publ_contact_author_name and _publ_contact_author_address.
PUBL005_ALERT_1_A  _publ_contact_author_email, _publ_contact_author_fax and
            _publ_contact_author_phone are all missing.
            At least one of these should be present.
PUBL006_ALERT_1_A  _publ_requested_journal is missing
```

```
           e.g. ’Acta Crystallographica Section C’
PUBL008_ALERT_1_A  _publ_section_title is missing. Title of paper.
PUBL009_ALERT_1_A  _publ_author_name is missing. List of author(s) name(s).
PUBL010_ALERT_1_A  _publ_author_address is missing. Author(s) address(es).
PUBL012_ALERT_1_A  _publ_section_abstract is missing.
           Abstract of paper in English.
```

```
   7 ALERT level A = Data missing that is essential or data in wrong format
   0 ALERT level G = General alerts. Data that may be required is missing
```

Publication of your CIF

You should attempt to resolve as many as possible of the alerts in all categories. Often the minor alerts point to easily fixed oversights, errors and omissions in your CIF or refinement strategy, so attention to these fine details can be worthwhile. In order to resolve some of the more serious problems it may be necessary to carry out additional measurements or structure refinements. However, the nature of your study may justify the reported deviations from journal submission requirements and the more serious of these should be commented upon in the discussion or experimental section of a paper or in the "special_details" fields of the CIF. *checkCIF* was carefully designed to identify outliers and unusual parameters, but every test has its limitations and alerts that are not important in a particular case may appear. Conversely, the absence of alerts does not guarantee there are no aspects of the results needing attention. It is up to the individual to critically assess their own results and, if necessary, seek expert advice.

If level A alerts remain, which you believe to be justified deviations, and you intend to submit this CIF for publication in a journal, you should additionally insert an explanation in your CIF using the Validation Reply Form (VRF) below. This will allow your explanation to be considered as part of the review process.

Validation response form

Please find below a validation response form (VRF) that can be filled in and pasted into your CIF.

```
# start Validation Reply Form
_vrf_PUBL004_GLOBAL
;
PROBLEM: The contact author’s name and address are missing,
RESPONSE: ...
;
_vrf_PUBL005_GLOBAL
;
PROBLEM: _publ_contact_author_email, _publ_contact_author_fax and
RESPONSE: ...
;
_vrf_PUBL006_GLOBAL
;
PROBLEM: _publ_requested_journal is missing
RESPONSE: ...
;
_vrf_PUBL008_GLOBAL
;
PROBLEM: _publ_section_title is missing. Title of paper.
RESPONSE: ...
;
_vrf_PUBL009_GLOBAL
```

```
;
PROBLEM: _publ_author_name is missing. List of author(s) name(s).
RESPONSE: ...
;
_vrf_PUBL010_GLOBAL
;
PROBLEM: _publ_author_address is missing. Author(s) address(es).
RESPONSE: ...
;
_vrf_PUBL012_GLOBAL
;
PROBLEM: _publ_section_abstract is missing.
RESPONSE: ...
;
# end Validation Reply Form
```

If you wish to submit your CIF for publication in Acta Crystallographica Section C or E, you should upload your CIF via the web. If you wish to submit your CIF for publication in IUCrData you should upload your CIF via the web. If your CIF is to form part of a submission to another IUCr journal, you will be asked, either during electronic submission or by the Co-editor handling your paper, to upload your CIF via our web site.

PLATON version of 13/07/2021; check.def file version of 13/07/2021

Datablock I - ellipsoid plot

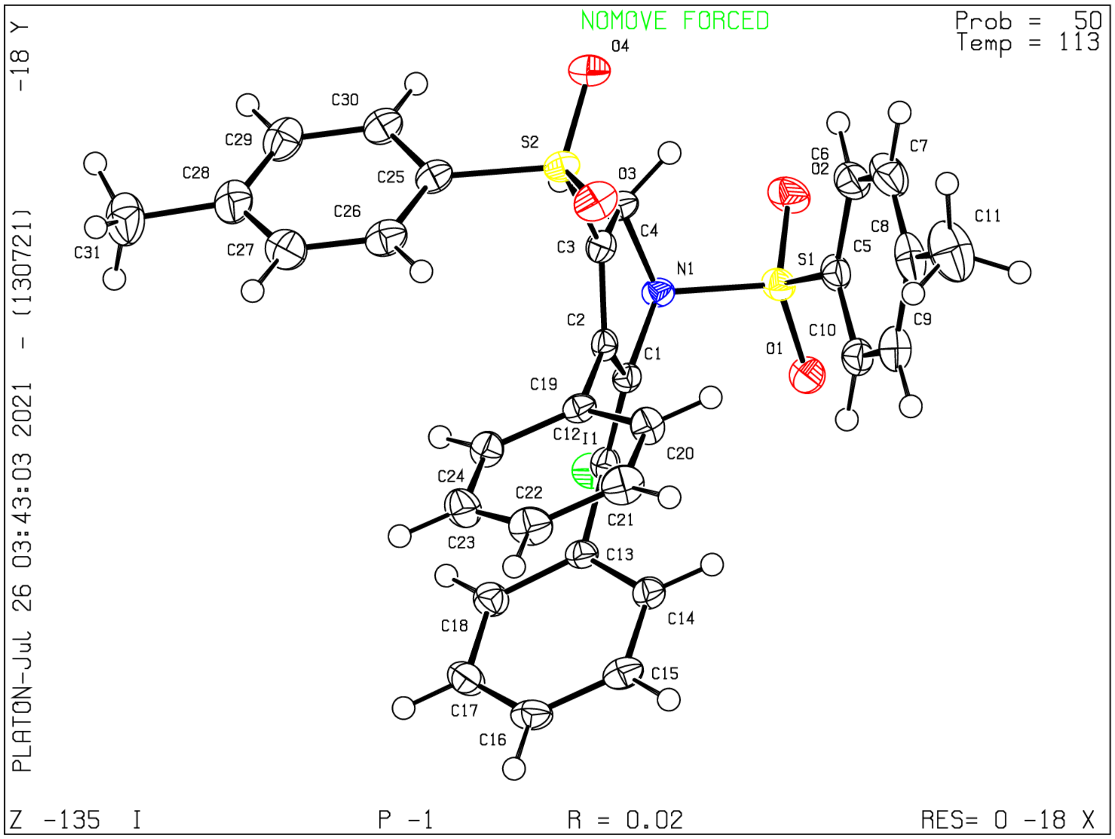

checkCIF/PLATON report

Structure factors have been supplied for datablock(s) I

THIS REPORT IS FOR GUIDANCE ONLY. IF USED AS PART OF A REVIEW PROCEDURE FOR PUBLICATION, IT SHOULD NOT REPLACE THE EXPERTISE OF AN EXPERIENCED CRYSTALLOGRAPHIC REFEREE.

No syntax errors found. CIF dictionary Interpreting this report

Datablock: I

```
Bond precision:  C-C = 0.0032 A          Wavelength=0.71073

Cell:            a=17.2341(2)     b=12.08534(15)     c=16.8038(3)
                 alpha=90         beta=90            gamma=90
Temperature:     113 K

                Calculated                 Reported
Volume          3499.90(9)                 3499.90(8)
Space group     P n a 21                   P n a 21
Hall group      P 2c -2n                   P 2c -2n
Moiety formula  C42 H33 N O4 S2 Se         C42 H33 N O4 S2 Se
Sum formula     C42 H33 N O4 S2 Se         C42 H33 N O4 S2 Se
Mr              758.77                     758.83
Dx,g cm-3       1.440                      1.440
Z               4                          4
Mu (mm-1)       1.237                      1.237
F000            1560.0                     1561.3
F000'           1561.19
h,k,lmax        20,14,19                   20,14,19
Nref            6168[ 3200]                5932
Tmin,Tmax       0.781,0.831                0.455,1.000
Tmin'           0.781

Correction method= # Reported T Limits: Tmin=0.455 Tmax=1.000
AbsCorr = MULTI-SCAN

Data completeness= 1.85/0.96      Theta(max)= 25.000

R(reflections)= 0.0234( 5600)     wR2(reflections)= 0.0526( 5932)

S = 1.045                   Npar= 453
```

The following ALERTS were generated. Each ALERT has the format
test-name_ALERT_alert-type_alert-level.
Click on the hyperlinks for more details of the test.

Alert level C

```
PLAT029_ALERT_3_C _diffrn_measured_fraction_theta_full value Low .      0.962 Why?
PLAT090_ALERT_3_C Poor Data / Parameter Ratio (Zmax > 18) ........       7.06 Note
```

Alert level G

```
PLAT003_ALERT_2_G Number of Uiso or Uij Restrained non-H Atoms ...          2 Report
PLAT073_ALERT_1_G H-atoms ref, but _hydrogen_treatment Reported as     constr Check
PLAT186_ALERT_4_G The CIF-Embedded .res File Contains ISOR Records          1 Report
PLAT860_ALERT_3_G Number of Least-Squares Restraints .............         13 Note
PLAT909_ALERT_3_G Percentage of I>2sig(I) Data at Theta(Max) Still        86% Note
PLAT978_ALERT_2_G Number C-C Bonds with Positive Residual Density.          6 Info
PLAT982_ALERT_1_G The Se-f'=   -0.0811 Deviates from  IT-value =      -0.0929 Check
PLAT983_ALERT_1_G The  S-f"=    0.1244 Deviates from  IT-Value =       0.1234 Check
PLAT983_ALERT_1_G The Se-f"=    2.3083 Deviates from  IT-Value =       2.2259 Check
```

```
   0 ALERT level A = Most likely a serious problem - resolve or explain
   0 ALERT level B = A potentially serious problem, consider carefully
   2 ALERT level C = Check. Ensure it is not caused by an omission or oversight
   9 ALERT level G = General information/check it is not something unexpected

   4 ALERT type 1 CIF construction/syntax error, inconsistent or missing data
   2 ALERT type 2 Indicator that the structure model may be wrong or deficient
   4 ALERT type 3 Indicator that the structure quality may be low
   1 ALERT type 4 Improvement, methodology, query or suggestion
   0 ALERT type 5 Informative message, check
```

checkCIF publication errors

Alert level A

```
PUBL004_ALERT_1_A  The contact author's name and address are missing,
            _publ_contact_author_name and _publ_contact_author_address.
PUBL005_ALERT_1_A  _publ_contact_author_email, _publ_contact_author_fax and
            _publ_contact_author_phone are all missing.
            At least one of these should be present.
PUBL006_ALERT_1_A  _publ_requested_journal is missing
            e.g. 'Acta Crystallographica Section C'
PUBL008_ALERT_1_A  _publ_section_title is missing. Title of paper.
PUBL009_ALERT_1_A  _publ_author_name is missing. List of author(s) name(s).
PUBL010_ALERT_1_A  _publ_author_address is missing. Author(s) address(es).
PUBL012_ALERT_1_A  _publ_section_abstract is missing.
            Abstract of paper in English.
```

```
   7 ALERT level A = Data missing that is essential or data in wrong format
   0 ALERT level G = General alerts. Data that may be required is missing
```

Publication of your CIF

You should attempt to resolve as many as possible of the alerts in all categories. Often the minor alerts point to easily fixed oversights, errors and omissions in your CIF or refinement strategy, so attention to these fine details can be worthwhile. In order to resolve some of the more serious problems it may be necessary to carry out additional measurements or structure refinements. However, the nature of your study may justify the reported deviations from journal submission requirements and the more serious of these should be commented upon in the discussion or experimental section of a paper or in the "special_details" fields of the CIF. *checkCIF* was carefully designed to identify outliers and unusual parameters, but every test has its limitations and alerts that are not important in a particular case may appear. Conversely, the absence of alerts does not guarantee there are no aspects of the results needing attention. It is up to the individual to critically assess their own results and, if necessary, seek expert advice.

If level A alerts remain, which you believe to be justified deviations, and you intend to submit this CIF for publication in a journal, you should additionally insert an explanation in your CIF using the Validation Reply Form (VRF) below. This will allow your explanation to be considered as part of the review process.

Validation response form

Please find below a validation response form (VRF) that can be filled in and pasted into your CIF.

```
# start Validation Reply Form
_vrf_PUBL004_GLOBAL
;
PROBLEM: The contact author’s name and address are missing,
RESPONSE: ...
;
_vrf_PUBL005_GLOBAL
;
PROBLEM: _publ_contact_author_email, _publ_contact_author_fax and
RESPONSE: ...
;
_vrf_PUBL006_GLOBAL
;
PROBLEM: _publ_requested_journal is missing
RESPONSE: ...
;
_vrf_PUBL008_GLOBAL
;
PROBLEM: _publ_section_title is missing. Title of paper.
RESPONSE: ...
;
_vrf_PUBL009_GLOBAL
;
PROBLEM: _publ_author_name is missing. List of author(s) name(s).
RESPONSE: ...
;
_vrf_PUBL010_GLOBAL
;
PROBLEM: _publ_author_address is missing. Author(s) address(es).
RESPONSE: ...
;
_vrf_PUBL012_GLOBAL
;
```

```
PROBLEM: _publ_section_abstract is missing.
RESPONSE: ...
;
# end Validation Reply Form
```

If you wish to submit your CIF for publication in Acta Crystallographica Section C or E, you should upload your CIF via the web. If you wish to submit your CIF for publication in IUCrData you should upload your CIF via the web. If your CIF is to form part of a submission to another IUCr journal, you will be asked, either during electronic submission or by the Co-editor handling your paper, to upload your CIF via our web site.

PLATON version of 22/12/2019; check.def file version of 13/12/2019

Datablock I - ellipsoid plot

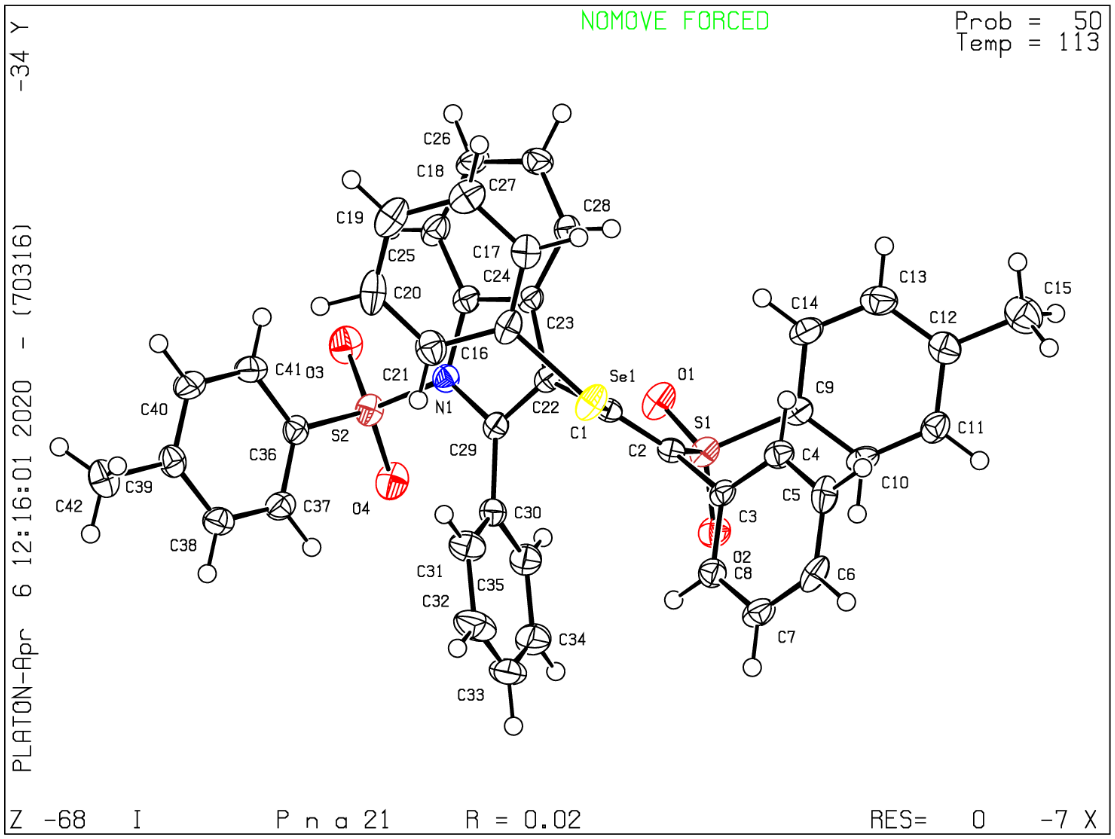

checkCIF/PLATON report

Structure factors have been supplied for datablock(s) I

THIS REPORT IS FOR GUIDANCE ONLY. IF USED AS PART OF A REVIEW PROCEDURE FOR PUBLICATION, IT SHOULD NOT REPLACE THE EXPERTISE OF AN EXPERIENCED CRYSTALLOGRAPHIC REFEREE.

No syntax errors found.   CIF dictionary   Interpreting this report

Datablock: I

```
Bond precision:  C-C = 0.0025 A          Wavelength=0.71073

Cell:           a=9.4299(3)         b=12.8979(4)      c=13.6741(4)
                alpha=102.414(3)    beta=107.900(3)   gamma=111.196(3)
Temperature:    113 K

                  Calculated                Reported
Volume            1372.27(10)               1372.27(8)
Space group       P -1                      P -1
Hall group        -P 1                      -P 1
Moiety formula    C35 H27 N O2 S2           C35 H27 N O2 S2
Sum formula       C35 H27 N O2 S2           C35 H27 N O2 S2
Mr                557.70                    557.69
Dx,g cm-3         1.350                     1.350
Z                 2                         2
Mu (mm-1)         0.229                     0.229
F000              584.0                     584.0
F000'             584.71
h,k,lmax          12,16,17                  11,16,17
Nref              6004                      5745
Tmin,Tmax         0.955,0.966               0.656,1.000
Tmin'             0.955

Correction method= # Reported T Limits: Tmin=0.656 Tmax=1.000
AbsCorr = MULTI-SCAN

Data completeness= 0.957          Theta(max)= 27.012

R(reflections)= 0.0354( 4884)     wR2(reflections)= 0.0865( 5745)

S = 1.048                   Npar= 362
```

The following ALERTS were generated. Each ALERT has the format
test-name_ALERT_alert-type_alert-level.
Click on the hyperlinks for more details of the test.

Alert level C

```
PLAT906_ALERT_3_C Large K Value in the Analysis of Variance ......      2.228 Check
PLAT911_ALERT_3_C Missing FCF Refl Between Thmin & STh/L=    0.600         13 Report
```

Alert level G

```
PLAT152_ALERT_1_G The Supplied and Calc. Volume s.u. Differ by ...          2 Units
PLAT154_ALERT_1_G The s.u.'s on the Cell Angles are Equal ..(Note)      0.003 Degree
PLAT910_ALERT_3_G Missing # of FCF Reflection(s) Below Theta(Min).          2 Note
PLAT912_ALERT_4_G Missing # of FCF Reflections Above STh/L=  0.600        238 Note
PLAT933_ALERT_2_G Number of OMIT Records in Embedded .res File ...         10 Note
PLAT978_ALERT_2_G Number C-C Bonds with Positive Residual Density.         15 Info
PLAT992_ALERT_5_G Repd & Actual _reflns_number_gt Values Differ by          1 Check
```

```
   0 ALERT level A = Most likely a serious problem - resolve or explain
   0 ALERT level B = A potentially serious problem, consider carefully
   2 ALERT level C = Check. Ensure it is not caused by an omission or oversight
   7 ALERT level G = General information/check it is not something unexpected

   2 ALERT type 1 CIF construction/syntax error, inconsistent or missing data
   2 ALERT type 2 Indicator that the structure model may be wrong or deficient
   3 ALERT type 3 Indicator that the structure quality may be low
   1 ALERT type 4 Improvement, methodology, query or suggestion
   1 ALERT type 5 Informative message, check
```

checkCIF publication errors

Alert level A

```
PUBL004_ALERT_1_A  The contact author's name and address are missing,
            _publ_contact_author_name and _publ_contact_author_address.
PUBL005_ALERT_1_A  _publ_contact_author_email, _publ_contact_author_fax and
            _publ_contact_author_phone are all missing.
            At least one of these should be present.
PUBL006_ALERT_1_A  _publ_requested_journal is missing
            e.g. 'Acta Crystallographica Section C'
PUBL008_ALERT_1_A  _publ_section_title is missing. Title of paper.
PUBL009_ALERT_1_A  _publ_author_name is missing. List of author(s) name(s).
PUBL010_ALERT_1_A  _publ_author_address is missing. Author(s) address(es).
PUBL012_ALERT_1_A  _publ_section_abstract is missing.
            Abstract of paper in English.
```

```
   7 ALERT level A = Data missing that is essential or data in wrong format
   0 ALERT level G = General alerts. Data that may be required is missing
```

Publication of your CIF

You should attempt to resolve as many as possible of the alerts in all categories. Often the minor alerts point to easily fixed oversights, errors and omissions in your CIF or refinement strategy, so attention to these fine details can be worthwhile. In order to resolve some of the more serious problems it may be necessary to carry out additional measurements or structure refinements. However, the nature of your study may justify the reported deviations from journal submission requirements and the more serious of these should be commented upon in the discussion or experimental section of a paper or in the "special_details" fields of the CIF. *checkCIF* was carefully designed to identify outliers and unusual parameters, but every test has its limitations and alerts that are not important in a particular case may appear. Conversely, the absence of alerts does not guarantee there are no aspects of the results needing attention. It is up to the individual to critically assess their own results and, if necessary, seek expert advice.

If level A alerts remain, which you believe to be justified deviations, and you intend to submit this CIF for publication in a journal, you should additionally insert an explanation in your CIF using the Validation Reply Form (VRF) below. This will allow your explanation to be considered as part of the review process.

Validation response form

Please find below a validation response form (VRF) that can be filled in and pasted into your CIF.

```
# start Validation Reply Form
_vrf_PUBL004_GLOBAL
;
PROBLEM: The contact author's name and address are missing,
RESPONSE: ...
;
_vrf_PUBL005_GLOBAL
;
PROBLEM: _publ_contact_author_email, _publ_contact_author_fax and
RESPONSE: ...
;
_vrf_PUBL006_GLOBAL
;
PROBLEM: _publ_requested_journal is missing
RESPONSE: ...
;
_vrf_PUBL008_GLOBAL
;
PROBLEM: _publ_section_title is missing. Title of paper.
RESPONSE: ...
;
_vrf_PUBL009_GLOBAL
;
PROBLEM: _publ_author_name is missing. List of author(s) name(s).
RESPONSE: ...
;
_vrf_PUBL010_GLOBAL
;
PROBLEM: _publ_author_address is missing. Author(s) address(es).
RESPONSE: ...
;
_vrf_PUBL012_GLOBAL
;
```

```
PROBLEM: _publ_section_abstract is missing.
RESPONSE: ...
;
# end Validation Reply Form
```

If you wish to submit your CIF for publication in Acta Crystallographica Section C or E, you should upload your CIF via the web. If you wish to submit your CIF for publication in IUCrData you should upload your CIF via the web. If your CIF is to form part of a submission to another IUCr journal, you will be asked, either during electronic submission or by the Co-editor handling your paper, to upload your CIF via our web site.

PLATON version of 22/12/2019; check.def file version of 13/12/2019

Datablock I - ellipsoid plot

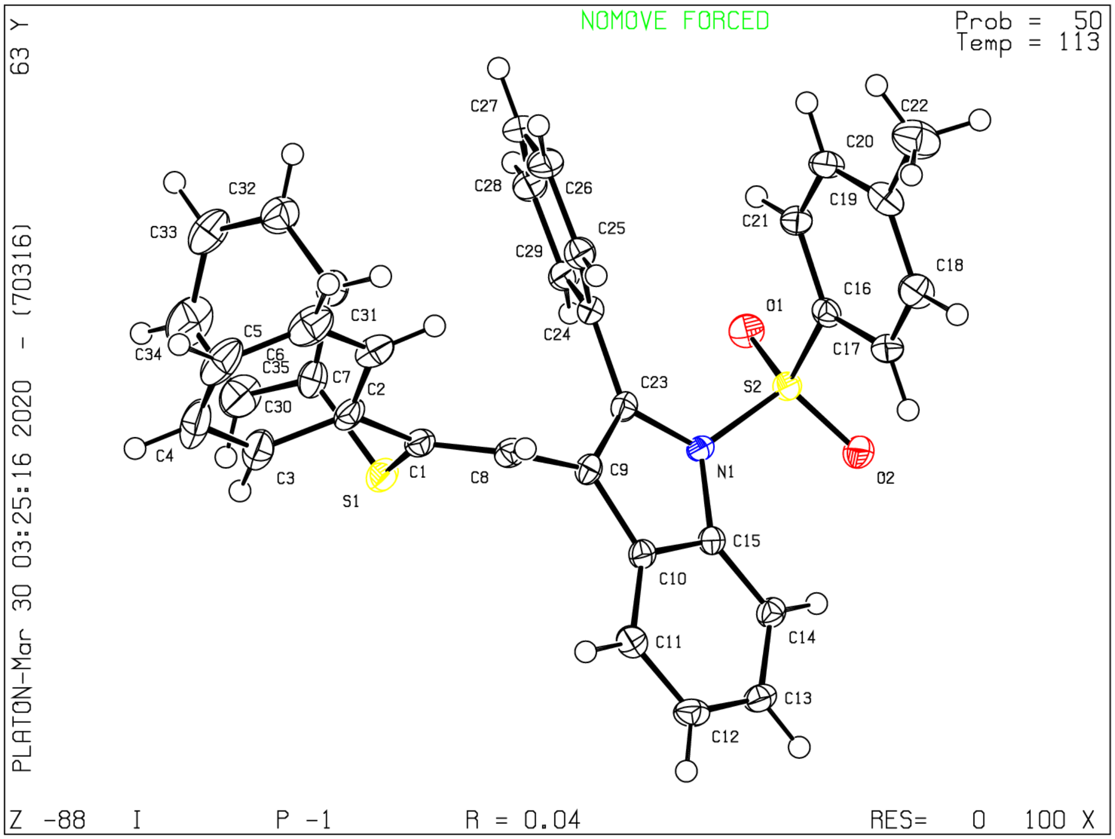

checkCIF/PLATON report

Structure factors have been supplied for datablock(s) I

THIS REPORT IS FOR GUIDANCE ONLY. IF USED AS PART OF A REVIEW PROCEDURE FOR PUBLICATION, IT SHOULD NOT REPLACE THE EXPERTISE OF AN EXPERIENCED CRYSTALLOGRAPHIC REFEREE.

No syntax errors found. CIF dictionary Interpreting this report

Datablock: I

```
Bond precision:  C-C = 0.0084 A          Wavelength=0.71073

Cell:          a=9.2544(2)       b=9.7531(2)      c=30.4790(9)
               alpha=89.135(2)   beta=88.150(2)   gamma=74.562(2)
Temperature:   113 K
```

	Calculated	Reported
Volume	2650.31(11)	2650.31(11)
Space group	P -1	P -1
Hall group	-P 1	-P 1
Moiety formula	C28 H24 I N O4 S2 [+ solvent]	2(C28 H24 I N O4 S2)
Sum formula	C28 H24 I N O4 S2 [+ solvent]	C56 H48 I2 N2 O8 S4
Mr	629.50	1259.00
Dx,g cm-3	1.578	1.578
Z	4	2
Mu (mm-1)	1.401	1.401
F000	1264.0	1264.0
F000’	1263.57	
h,k,lmax	11,11,36	11,11,36
Nref	9304	9290
Tmin,Tmax	0.663,0.756	0.307,1.000
Tmin’	0.565	

```
Correction method= # Reported T Limits: Tmin=0.307 Tmax=1.000
AbsCorr = MULTI-SCAN

Data completeness= 0.998          Theta(max)= 24.998

R(reflections)= 0.0532( 8370)     wR2(reflections)= 0.1063( 9290)

S = 1.274                   Npar= 653
```

```
The following ALERTS were generated. Each ALERT has the format
       test-name_ALERT_alert-type_alert-level.
Click on the hyperlinks for more details of the test.
```

Alert level C

```
PLAT342_ALERT_3_C Low Bond Precision on  C-C Bonds ...............    0.00837 Ang.
PLAT761_ALERT_1_C CIF Contains no X-H Bonds ......................     Please Check
PLAT762_ALERT_1_C CIF Contains no X-Y-H or H-Y-H Angles ..........     Please Check
PLAT906_ALERT_3_C Large K Value in the Analysis of Variance ......      6.976 Check
PLAT911_ALERT_3_C Missing FCF Refl Between Thmin & STh/L=    0.595         14 Report
PLAT977_ALERT_2_C Check Negative Difference Density on H11              -0.39 eA-3
```

Alert level G

```
PLAT042_ALERT_1_G Calc. and Reported MoietyFormula Strings  Differ     Please Check
PLAT045_ALERT_1_G Calculated and Reported Z Differ by a Factor ...       2.00 Check
PLAT083_ALERT_2_G SHELXL Second Parameter in WGHT  Unusually Large      15.10 Why ?
PLAT154_ALERT_1_G The s.u.'s on the Cell Angles are Equal ..(Note)      0.002 Degree
PLAT431_ALERT_2_G Short Inter HL..A Contact  I1       ..O2       .       2.97 Ang.
                                                 1-x,-y,2-z  =      2_657 Check
PLAT605_ALERT_4_G Largest Solvent Accessible VOID in the Structure         22 A**3
PLAT909_ALERT_3_G Percentage of I>2sig(I) Data at Theta(Max) Still        75% Note
PLAT910_ALERT_3_G Missing # of FCF Reflection(s) Below Theta(Min).          2 Note
PLAT933_ALERT_2_G Number of OMIT Records in Embedded .res File ...          5 Note
PLAT978_ALERT_2_G Number C-C Bonds with Positive Residual Density.          1 Info
```

```
   0 ALERT level A = Most likely a serious problem - resolve or explain
   0 ALERT level B = A potentially serious problem, consider carefully
   6 ALERT level C = Check. Ensure it is not caused by an omission or oversight
  10 ALERT level G = General information/check it is not something unexpected

   5 ALERT type 1 CIF construction/syntax error, inconsistent or missing data
   5 ALERT type 2 Indicator that the structure model may be wrong or deficient
   5 ALERT type 3 Indicator that the structure quality may be low
   1 ALERT type 4 Improvement, methodology, query or suggestion
   0 ALERT type 5 Informative message, check
```

---

checkCIF publication errors

Alert level A

```
PUBL004_ALERT_1_A  The contact author's name and address are missing,
            _publ_contact_author_name and _publ_contact_author_address.
PUBL005_ALERT_1_A  _publ_contact_author_email, _publ_contact_author_fax and
            _publ_contact_author_phone are all missing.
            At least one of these should be present.
PUBL006_ALERT_1_A  _publ_requested_journal is missing
            e.g. 'Acta Crystallographica Section C'
PUBL008_ALERT_1_A  _publ_section_title is missing. Title of paper.
PUBL009_ALERT_1_A  _publ_author_name is missing. List of author(s) name(s).
PUBL010_ALERT_1_A  _publ_author_address is missing. Author(s) address(es).
PUBL012_ALERT_1_A  _publ_section_abstract is missing.
            Abstract of paper in English.
```

```
   7 ALERT level A = Data missing that is essential or data in wrong format
   0 ALERT level G = General alerts. Data that may be required is missing
```

Publication of your CIF

You should attempt to resolve as many as possible of the alerts in all categories. Often the minor alerts point to easily fixed oversights, errors and omissions in your CIF or refinement strategy, so attention to these fine details can be worthwhile. In order to resolve some of the more serious problems it may be necessary to carry out additional measurements or structure refinements. However, the nature of your study may justify the reported deviations from journal submission requirements and the more serious of these should be commented upon in the discussion or experimental section of a paper or in the "special_details" fields of the CIF. *checkCIF* was carefully designed to identify outliers and unusual parameters, but every test has its limitations and alerts that are not important in a particular case may appear. Conversely, the absence of alerts does not guarantee there are no aspects of the results needing attention. It is up to the individual to critically assess their own results and, if necessary, seek expert advice.

If level A alerts remain, which you believe to be justified deviations, and you intend to submit this CIF for publication in a journal, you should additionally insert an explanation in your CIF using the Validation Reply Form (VRF) below. This will allow your explanation to be considered as part of the review process.

Validation response form

Please find below a validation response form (VRF) that can be filled in and pasted into your CIF.

```
# start Validation Reply Form
_vrf_PUBL004_GLOBAL
;
PROBLEM: The contact author’s name and address are missing,
RESPONSE: ...
;
_vrf_PUBL005_GLOBAL
;
PROBLEM: _publ_contact_author_email, _publ_contact_author_fax and
RESPONSE: ...
;
_vrf_PUBL006_GLOBAL
;
PROBLEM: _publ_requested_journal is missing
RESPONSE: ...
;
_vrf_PUBL008_GLOBAL
;
PROBLEM: _publ_section_title is missing. Title of paper.
RESPONSE: ...
;
_vrf_PUBL009_GLOBAL
;
PROBLEM: _publ_author_name is missing. List of author(s) name(s).
RESPONSE: ...
;
_vrf_PUBL010_GLOBAL
;
PROBLEM: _publ_author_address is missing. Author(s) address(es).
RESPONSE: ...
;
_vrf_PUBL012_GLOBAL
```

```
;
PROBLEM: _publ_section_abstract is missing.
RESPONSE: ...
;
# end Validation Reply Form
```

If you wish to submit your CIF for publication in Acta Crystallographica Section C or E, you should upload your CIF via the web. If you wish to submit your CIF for publication in IUCrData you should upload your CIF via the web. If your CIF is to form part of a submission to another IUCr journal, you will be asked, either during electronic submission or by the Co-editor handling your paper, to upload your CIF via our web site.

PLATON version of 18/09/2020; check.def file version of 20/08/2020

Datablock I - ellipsoid plot

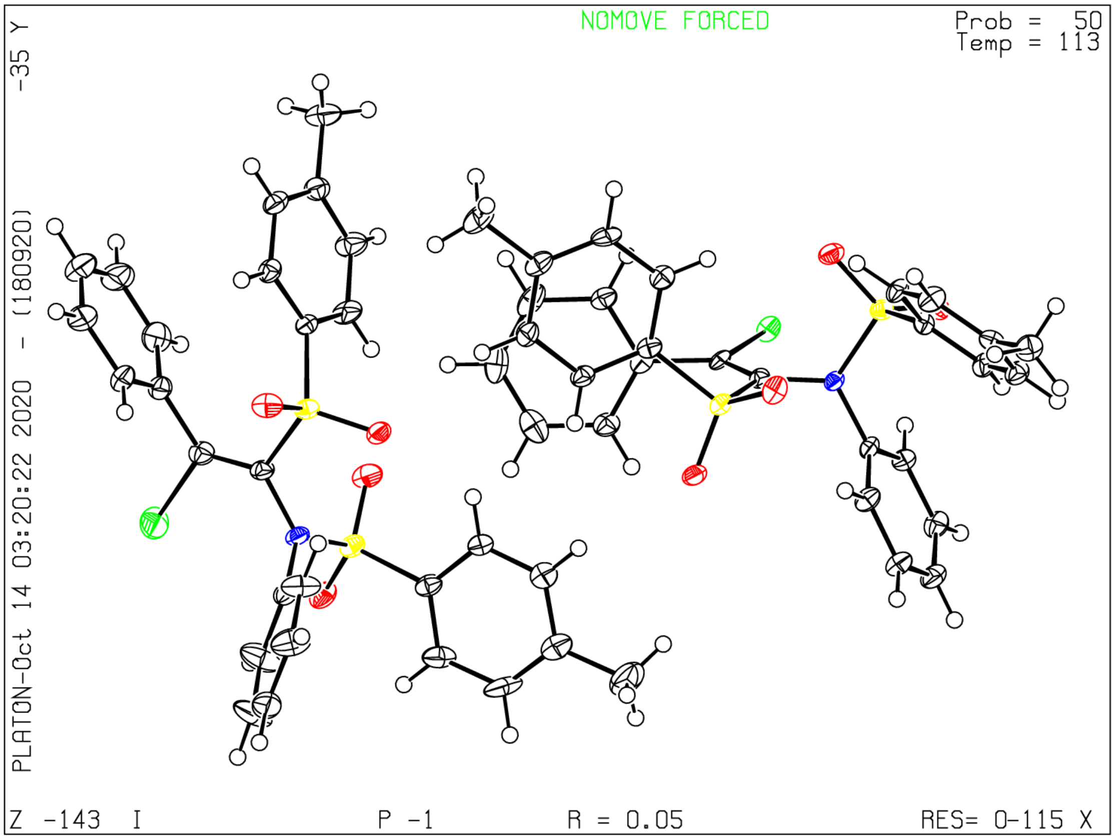

checkCIF/PLATON report

Structure factors have been supplied for datablock(s) I

THIS REPORT IS FOR GUIDANCE ONLY. IF USED AS PART OF A REVIEW PROCEDURE FOR PUBLICATION, IT SHOULD NOT REPLACE THE EXPERTISE OF AN EXPERIENCED CRYSTALLOGRAPHIC REFEREE.

No syntax errors found. CIF dictionary Interpreting this report

Datablock: I

```
Bond precision:  C-C = 0.0038 A          Wavelength=0.71073

Cell:           a=9.9501(1)       b=13.7256(2)     c=20.3493(3)
                alpha=86.067(1)   beta=84.880(1)   gamma=87.521(1)
Temperature:    113 K

                 Calculated                Reported
Volume           2759.59(6)                2759.59(6)
Space group      P -1                      P -1
Hall group       -P 1                      -P 1
Moiety formula   C30 H24 I N O4 S2         2(C30 H24 I N O4 S2)
Sum formula      C30 H24 I N O4 S2         C60 H48 I2 N2 O8 S4
Mr               653.52                    1307.04
Dx,g cm-3        1.573                     1.573
Z                4                         2
Mu (mm-1)        1.349                     1.349
F000             1312.0                    1312.0
F000'            1311.59
h,k,lmax         11,16,24                  11,16,24
Nref             9727                      9687
Tmin,Tmax        0.630,0.667               0.268,1.000
Tmin'            0.617

Correction method= # Reported T Limits: Tmin=0.268 Tmax=1.000
AbsCorr = MULTI-SCAN

Data completeness= 0.996          Theta(max)= 24.998

R(reflections)= 0.0269( 8913)     wR2(reflections)= 0.0716( 9687)

S = 1.032                 Npar= 689
```

The following ALERTS were generated. Each ALERT has the format
test-name_ALERT_alert-type_alert-level.
Click on the hyperlinks for more details of the test.

Alert level C

```
PLAT911_ALERT_3_C Missing FCF Refl Between Thmin & STh/L=    0.595         38 Report
PLAT918_ALERT_3_C Reflection(s) with I(obs) much Smaller I(calc) .          1 Check
```

Alert level G

```
PLAT003_ALERT_2_G Number of Uiso or Uij Restrained non-H Atoms ...         74 Report
PLAT042_ALERT_1_G Calc. and Reported MoietyFormula Strings  Differ     Please Check
PLAT045_ALERT_1_G Calculated and Reported Z Differ by a Factor ...       2.00 Check
PLAT154_ALERT_1_G The s.u.'s on the Cell Angles are Equal ..(Note)      0.001 Degree
PLAT178_ALERT_4_G The CIF-Embedded .res File Contains SIMU Records          1 Report
PLAT431_ALERT_2_G Short Inter HL..A Contact  I2       ..O5       .       3.19 Ang.
                                               1-x,-y,1-z  =      2_656 Check
PLAT860_ALERT_3_G Number of Least-Squares Restraints ............       2082 Note
PLAT909_ALERT_3_G Percentage of I>2sig(I) Data at Theta(Max) Still        83% Note
PLAT910_ALERT_3_G Missing # of FCF Reflection(s) Below Theta(Min).          2 Note
PLAT933_ALERT_2_G Number of OMIT Records in Embedded .res File ...         31 Note
PLAT978_ALERT_2_G Number C-C Bonds with Positive Residual Density.          7 Info
```

0 **ALERT level A** = Most likely a serious problem - resolve or explain
0 **ALERT level B** = A potentially serious problem, consider carefully
2 **ALERT level C** = Check. Ensure it is not caused by an omission or oversight
11 **ALERT level G** = General information/check it is not something unexpected

3 ALERT type 1 CIF construction/syntax error, inconsistent or missing data
4 ALERT type 2 Indicator that the structure model may be wrong or deficient
5 ALERT type 3 Indicator that the structure quality may be low
1 ALERT type 4 Improvement, methodology, query or suggestion
0 ALERT type 5 Informative message, check

checkCIF publication errors

Alert level A

```
PUBL004_ALERT_1_A  The contact author's name and address are missing,
            _publ_contact_author_name and _publ_contact_author_address.
PUBL005_ALERT_1_A  _publ_contact_author_email, _publ_contact_author_fax and
            _publ_contact_author_phone are all missing.
            At least one of these should be present.
PUBL006_ALERT_1_A  _publ_requested_journal is missing
            e.g. 'Acta Crystallographica Section C'
PUBL008_ALERT_1_A  _publ_section_title is missing. Title of paper.
PUBL009_ALERT_1_A  _publ_author_name is missing. List of author(s) name(s).
PUBL010_ALERT_1_A  _publ_author_address is missing. Author(s) address(es).
PUBL012_ALERT_1_A  _publ_section_abstract is missing.
            Abstract of paper in English.
```

7 **ALERT level A** = Data missing that is essential or data in wrong format
0 **ALERT level G** = General alerts. Data that may be required is missing

Publication of your CIF

You should attempt to resolve as many as possible of the alerts in all categories. Often the minor alerts point to easily fixed oversights, errors and omissions in your CIF or refinement strategy, so attention to these fine details can be worthwhile. In order to resolve some of the more serious problems it may be necessary to carry out additional measurements or structure refinements. However, the nature of your study may justify the reported deviations from journal submission requirements and the more serious of these should be commented upon in the discussion or experimental section of a paper or in the "special_details" fields of the CIF. *checkCIF* was carefully designed to identify outliers and unusual parameters, but every test has its limitations and alerts that are not important in a particular case may appear. Conversely, the absence of alerts does not guarantee there are no aspects of the results needing attention. It is up to the individual to critically assess their own results and, if necessary, seek expert advice.

If level A alerts remain, which you believe to be justified deviations, and you intend to submit this CIF for publication in a journal, you should additionally insert an explanation in your CIF using the Validation Reply Form (VRF) below. This will allow your explanation to be considered as part of the review process.

Validation response form

Please find below a validation response form (VRF) that can be filled in and pasted into your CIF.

```
# start Validation Reply Form
_vrf_PUBL004_GLOBAL
;
PROBLEM: The contact author’s name and address are missing,
RESPONSE: ...
;
_vrf_PUBL005_GLOBAL
;
PROBLEM: _publ_contact_author_email, _publ_contact_author_fax and
RESPONSE: ...
;
_vrf_PUBL006_GLOBAL
;
PROBLEM: _publ_requested_journal is missing
RESPONSE: ...
;
_vrf_PUBL008_GLOBAL
;
PROBLEM: _publ_section_title is missing. Title of paper.
RESPONSE: ...
;
_vrf_PUBL009_GLOBAL
;
PROBLEM: _publ_author_name is missing. List of author(s) name(s).
RESPONSE: ...
;
_vrf_PUBL010_GLOBAL
;
PROBLEM: _publ_author_address is missing. Author(s) address(es).
RESPONSE: ...
;
_vrf_PUBL012_GLOBAL
;
```

```
PROBLEM: _publ_section_abstract is missing.
RESPONSE: ...
;
# end Validation Reply Form
```

If you wish to submit your CIF for publication in Acta Crystallographica Section C or E, you should upload your CIF via the web. If you wish to submit your CIF for publication in IUCrData you should upload your CIF via the web. If your CIF is to form part of a submission to another IUCr journal, you will be asked, either during electronic submission or by the Co-editor handling your paper, to upload your CIF via our web site.

PLATON version of 18/09/2020; check.def file version of 20/08/2020

Datablock I - ellipsoid plot

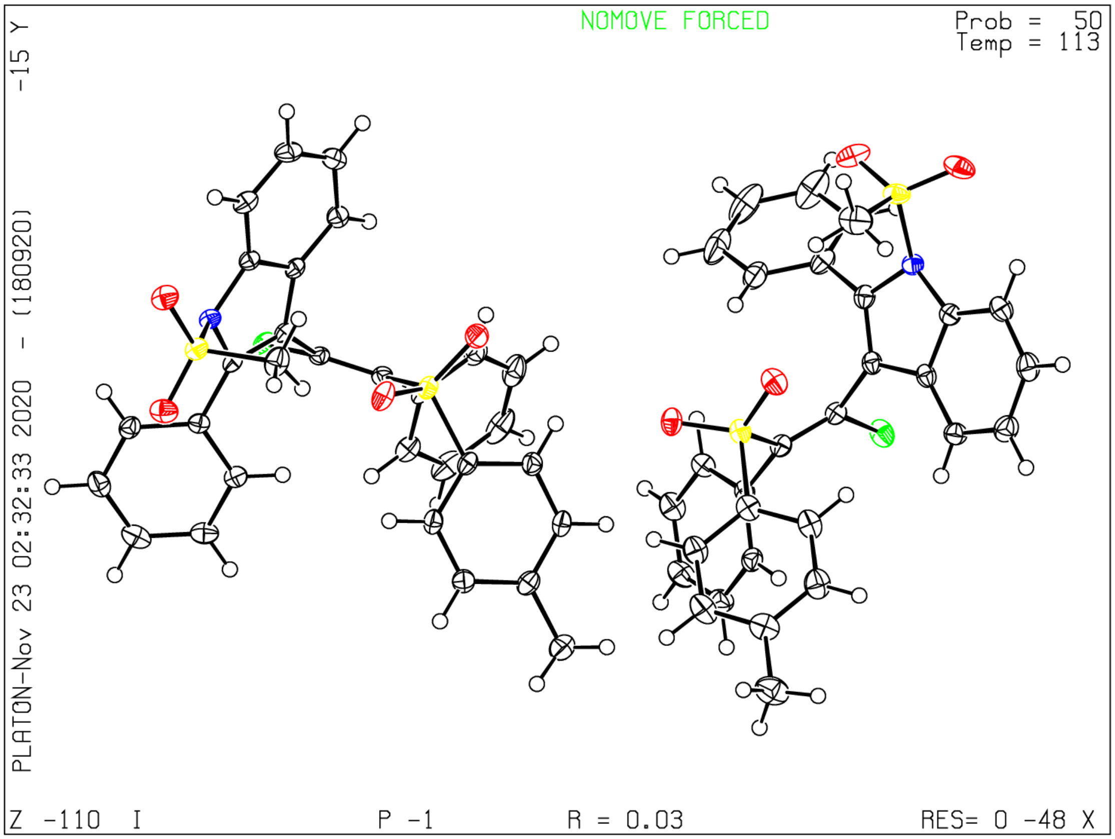

checkCIF/PLATON report

Structure factors have been supplied for datablock(s) I

THIS REPORT IS FOR GUIDANCE ONLY. IF USED AS PART OF A REVIEW PROCEDURE FOR PUBLICATION, IT SHOULD NOT REPLACE THE EXPERTISE OF AN EXPERIENCED CRYSTALLOGRAPHIC REFEREE.

No syntax errors found. CIF dictionary Interpreting this report

Datablock: I

```
Bond precision:  C-C = 0.0032 A          Wavelength=0.71073

Cell:           a=9.2709(1)        b=11.0543(1)      c=33.4440(3)
                alpha=82.428(1)    beta=87.103(1)    gamma=87.095(1)
Temperature:    113 K

                 Calculated                 Reported
Volume           3389.87(6)                 3389.87(6)
Space group      P -1                       P -1
Hall group       -P 1                       -P 1
Moiety formula   C38 H32 I N O5 S2          2(C38 H32 I N O5 S2)
Sum formula      C38 H32 I N O5 S2          C76 H64 I2 N2 O10 S4
Mr               773.67                     1547.33
Dx,g cm-3        1.516                      1.516
Z                4                          2
Mu (mm-1)        1.113                      1.113
F000             1568.0                     1568.0
F000'            1567.70
h,k,lmax         11,14,42                   11,14,42
Nref             14875                      14161
Tmin,Tmax        0.766,0.800                0.558,1.000
Tmin'            0.757

Correction method= # Reported T Limits: Tmin=0.558 Tmax=1.000
AbsCorr = MULTI-SCAN

Data completeness= 0.952          Theta(max)= 27.076

R(reflections)= 0.0285( 12283)    wR2(reflections)= 0.0670( 14161)

S = 1.060                   Npar= 853
```

The following ALERTS were generated. Each ALERT has the format
test-name_ALERT_alert-type_alert-level.
Click on the hyperlinks for more details of the test.

Alert level B

```
PLAT434_ALERT_2_B Short Inter HL..HL Contact I2       ..I2              3.43 Ang.
                                               1-x,1-y,1-z  =      2_666 Check
```

Alert level C

```
PLAT911_ALERT_3_C Missing FCF Refl Between Thmin & STh/L=    0.600         62 Report
```

Alert level G

```
PLAT002_ALERT_2_G Number of Distance or Angle Restraints on AtSite          2 Note
PLAT042_ALERT_1_G Calc. and Reported MoietyFormula Strings  Differ     Please Check
PLAT045_ALERT_1_G Calculated and Reported Z Differ by a Factor ...       2.00 Check
PLAT154_ALERT_1_G The s.u.'s on the Cell Angles are Equal ..(Note)      0.001 Degree
PLAT172_ALERT_4_G The CIF-Embedded .res File Contains DFIX Records          1 Report
PLAT371_ALERT_2_G Long   C(sp2)-C(sp1) Bond  C23      - C24      .       1.43 Ang.
PLAT371_ALERT_2_G Long   C(sp2)-C(sp1) Bond  C25      - C26      .       1.43 Ang.
PLAT371_ALERT_2_G Long   C(sp2)-C(sp1) Bond  C61      - C62      .       1.44 Ang.
PLAT371_ALERT_2_G Long   C(sp2)-C(sp1) Bond  C63      - C64      .       1.44 Ang.
PLAT860_ALERT_3_G Number of Least-Squares Restraints .............          1 Note
PLAT910_ALERT_3_G Missing # of FCF Reflection(s) Below Theta(Min).          2 Note
PLAT912_ALERT_4_G Missing # of FCF Reflections Above STh/L=  0.600        652 Note
PLAT933_ALERT_2_G Number of OMIT Records in Embedded .res File ...         50 Note
PLAT978_ALERT_2_G Number C-C Bonds with Positive Residual Density.          8 Info
```

```
   0 ALERT level A = Most likely a serious problem - resolve or explain
   1 ALERT level B = A potentially serious problem, consider carefully
   1 ALERT level C = Check. Ensure it is not caused by an omission or oversight
  14 ALERT level G = General information/check it is not something unexpected

   3 ALERT type 1 CIF construction/syntax error, inconsistent or missing data
   8 ALERT type 2 Indicator that the structure model may be wrong or deficient
   3 ALERT type 3 Indicator that the structure quality may be low
   2 ALERT type 4 Improvement, methodology, query or suggestion
   0 ALERT type 5 Informative message, check
```

checkCIF publication errors

Alert level A

```
PUBL004_ALERT_1_A  The contact author's name and address are missing,
            _publ_contact_author_name and _publ_contact_author_address.
PUBL005_ALERT_1_A  _publ_contact_author_email, _publ_contact_author_fax and
            _publ_contact_author_phone are all missing.
            At least one of these should be present.
PUBL006_ALERT_1_A  _publ_requested_journal is missing
            e.g. 'Acta Crystallographica Section C'
PUBL008_ALERT_1_A  _publ_section_title is missing. Title of paper.
PUBL009_ALERT_1_A  _publ_author_name is missing. List of author(s) name(s).
PUBL010_ALERT_1_A  _publ_author_address is missing. Author(s) address(es).
PUBL012_ALERT_1_A  _publ_section_abstract is missing.
            Abstract of paper in English.
```

```
   7 ALERT level A = Data missing that is essential or data in wrong format
   0 ALERT level G = General alerts. Data that may be required is missing
```

Publication of your CIF

You should attempt to resolve as many as possible of the alerts in all categories. Often the minor alerts point to easily fixed oversights, errors and omissions in your CIF or refinement strategy, so attention to these fine details can be worthwhile. In order to resolve some of the more serious problems it may be necessary to carry out additional measurements or structure refinements. However, the nature of your study may justify the reported deviations from journal submission requirements and the more serious of these should be commented upon in the discussion or experimental section of a paper or in the "special_details" fields of the CIF. *checkCIF* was carefully designed to identify outliers and unusual parameters, but every test has its limitations and alerts that are not important in a particular case may appear. Conversely, the absence of alerts does not guarantee there are no aspects of the results needing attention. It is up to the individual to critically assess their own results and, if necessary, seek expert advice.

If level A alerts remain, which you believe to be justified deviations, and you intend to submit this CIF for publication in a journal, you should additionally insert an explanation in your CIF using the Validation Reply Form (VRF) below. This will allow your explanation to be considered as part of the review process.

Validation response form

Please find below a validation response form (VRF) that can be filled in and pasted into your CIF.

```
# start Validation Reply Form
_vrf_PUBL004_GLOBAL
;
PROBLEM: The contact author’s name and address are missing,
RESPONSE: ...
;
_vrf_PUBL005_GLOBAL
;
PROBLEM: _publ_contact_author_email, _publ_contact_author_fax and
RESPONSE: ...
;
_vrf_PUBL006_GLOBAL
;
PROBLEM: _publ_requested_journal is missing
RESPONSE: ...
;
_vrf_PUBL008_GLOBAL
;
PROBLEM: _publ_section_title is missing. Title of paper.
RESPONSE: ...
;
_vrf_PUBL009_GLOBAL
;
PROBLEM: _publ_author_name is missing. List of author(s) name(s).
RESPONSE: ...
;
_vrf_PUBL010_GLOBAL
;
PROBLEM: _publ_author_address is missing. Author(s) address(es).
RESPONSE: ...
;
_vrf_PUBL012_GLOBAL
;
```

```
PROBLEM: _publ_section_abstract is missing.
RESPONSE: ...
;
# end Validation Reply Form
```

If you wish to submit your CIF for publication in Acta Crystallographica Section C or E, you should upload your CIF via the web. If you wish to submit your CIF for publication in IUCrData you should upload your CIF via the web. If your CIF is to form part of a submission to another IUCr journal, you will be asked, either during electronic submission or by the Co-editor handling your paper, to upload your CIF via our web site.

PLATON version of 05/12/2020; check.def file version of 05/12/2020

Datablock I - ellipsoid plot

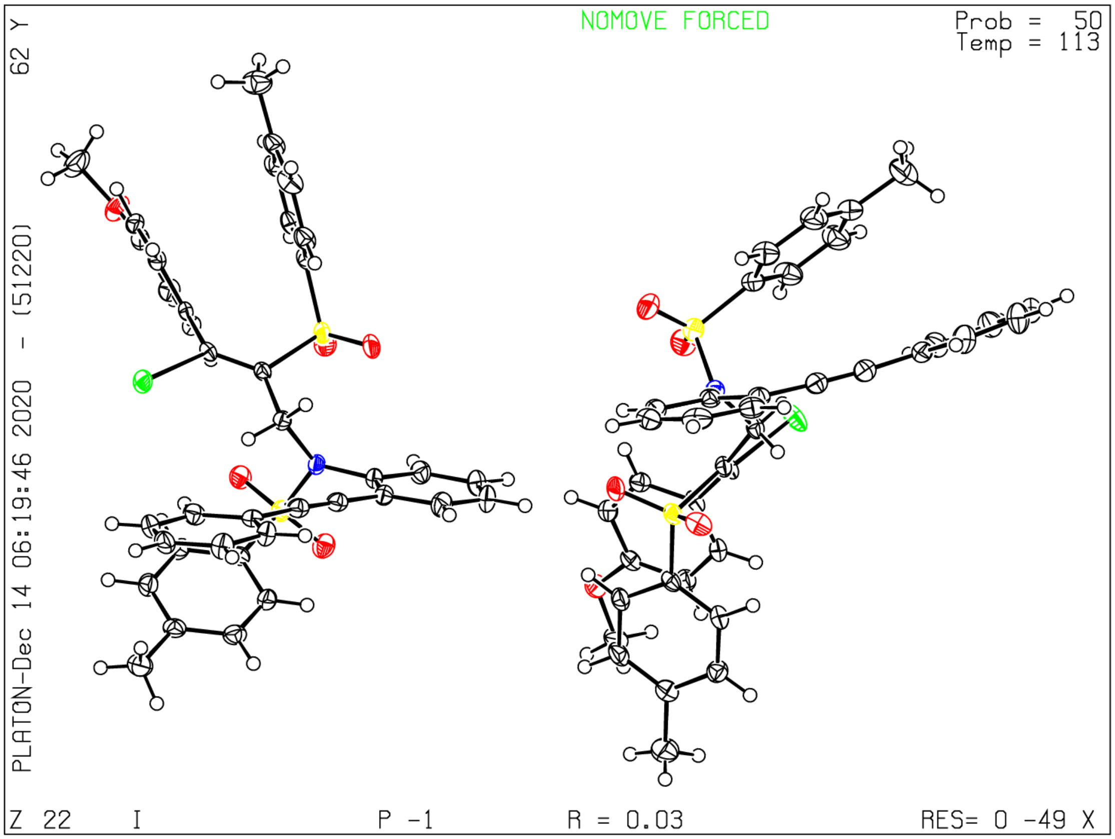

3. Supplementary References

1. Xu, W., Wang, G., Xie, X. & Liu, Y., Gold (I)-Catalyzed Formal Intramolecular Dehydro-Diels-Alder Reaction of Ynamide-ynes: Synthesis of Functionalized Benzo[b]carbazoles. *Org. Lett.* **20**, 3273-3277 (2018).
2. Dutta, S., Mallick, R. K., Prasad, R., Gandon, V. & Sahoo, A. K. Alkyne Versus Ynamide Reactivity: Regioselective Radical Cyclization of Yne-Ynamides. *Angew. Chem., Int. Ed*. **58**, 2289-2294 (2019).
3. Dutta, S., Prabagar, B., Vanjari, R., Gandon, V. & Sahoo, A. K. An unconventional sulfur-to-selenium-to-carbon radical transfer: chemo-and regioselective cyclization of yne-ynamides. *Green Chem*. **22**, 1113-1118 (2020).
4. Pi, R., Zhou, M.-B., Yang, Y., Gao, C., Song, R.-J. & Li, J.-H. Rhodium(III)-catalyzed oxidative bicyclization of 4-arylbut-3-yn-1-amines with internal alkynes through C–H functionalization. *Chem. Commun*. **51**, 13550-13553 (2015).
5. Mutra, M. R., Kudale, V. S., Li, J., Tsai, W.-H. & Wang, J.-J. Alkene versus alkyne reactivity in unactivated 1,6-enynes: regio- and chemoselective radical cyclization with chalcogens under metal- and oxidant-free conditions. *Green Chem.* **22**, 2288-2300 (2020).
6. Lu, N., Zhang, Z., Ma, N., Wu, C., Zhang, G., Lin, Q. & Liu, T., Copper-Catalyzed Difunctionalization of Allenes with Sulfonyl Iodides Leading to (*E*)−α-Iodomethyl Vinylsulfones. *Org. Lett*. **20**, 4318−4322 (2018).
7. Gorsche, C., Koch, T., Moszner, N. & Liska, R. Exploring the benefits of β-allyl sulfones for more homogeneous dimethacrylate photopolymer networks. *Polym. Chem*. **6**, 2038-2047 (2015).
8. Zhang, G., Fan, Q., Wang, H., Zhao, Y. & Ding, C. $NaHSO_3$-Mediated Direct Synthesis of Sulfinic Esters from Sulfonyl Hydrazides under Transition-Metal-Free Conditions. *Adv. Synth. Catal*. **363**, 833-837 (2021).
9. Kirihara, M., Naito, S., Nishimura, Y., Ishizuka, Y., Iwai, T., Takeuchi, H., Ogata, T., Hanai, H., Kinoshita, Y., Kishida, M., Yamazaki, K., Noguchi, T. & Yamashoji, S. Oxidation of disulfides with electrophilic halogenating reagents: concise methods for preparation of thiosulfonates and sulfonyl halides. Tetrahedron **70**, 2464-2471 (2014).
